# Supplementary material for: An organometallic approach to the synthesis of heteropolycyclic compounds from benzenes
Source: Nat Commun. 2025 Aug 13;16:7504. doi: 10.1038/s41467-025-62835-2 (PMC12350676; doi:10.1038/s41467-025-62835-2)
Supplement: Supplementary file 1 — Supplementary Information [file 41467_2025_62835_MOESM1_ESM.pdf]

Supplementary Information

An organometallic approach to the synthesis of heteropolycyclic compounds from benzene

Authors: Paolo Siano[1], Louis A. Diment[1], Daniel J. Siela[1], Megan N. Ericson[1], Matt McGraw[1], Benjamin F. Livaudais,[1] Diane A. Dickie[1], and W. Dean Harman [1]*

Affiliation: [1]Department of Chemistry, University of Virginia; Charlottesville, VA 22904 U.S.A.

*Corresponding author. Email: wdh5z@virginia.edu

Table of Contents

NMR Spectra: 2

Supplementary Methods: 188

DFT Analysis: 245

Crystallographic Data: 246

Supplementary References: 253

Compounds **1, 2, 3, 4** and **82** have been previously reported.[1] Previously unreported SC-XRD data determined for compounds **2**, and **3** can be found in the Crystallographic Data section of this SI.

NMR Spectra:

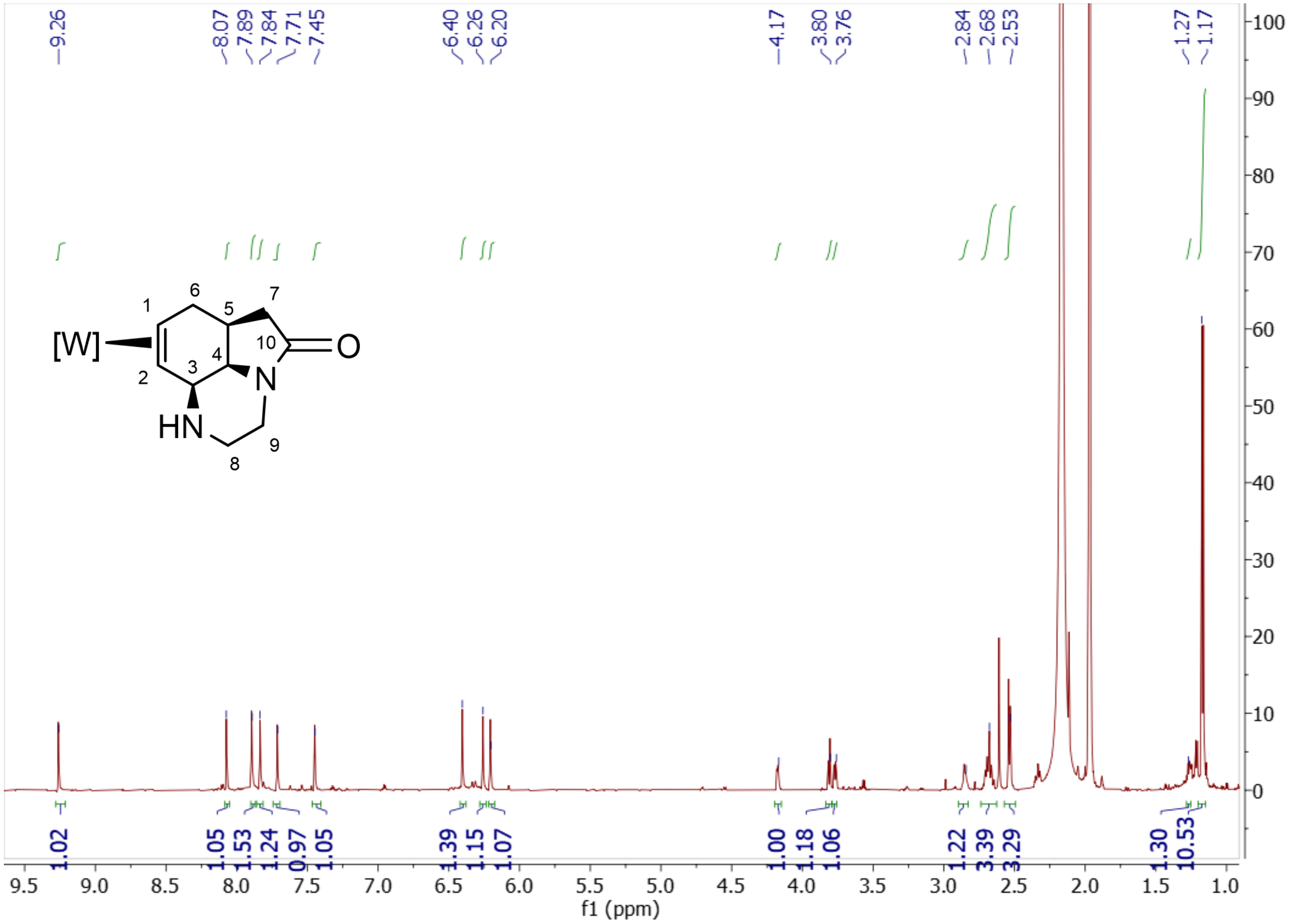

Supplementary Fig. 1: ^{1}H-NMR (CD3CN) of Compound **5**.

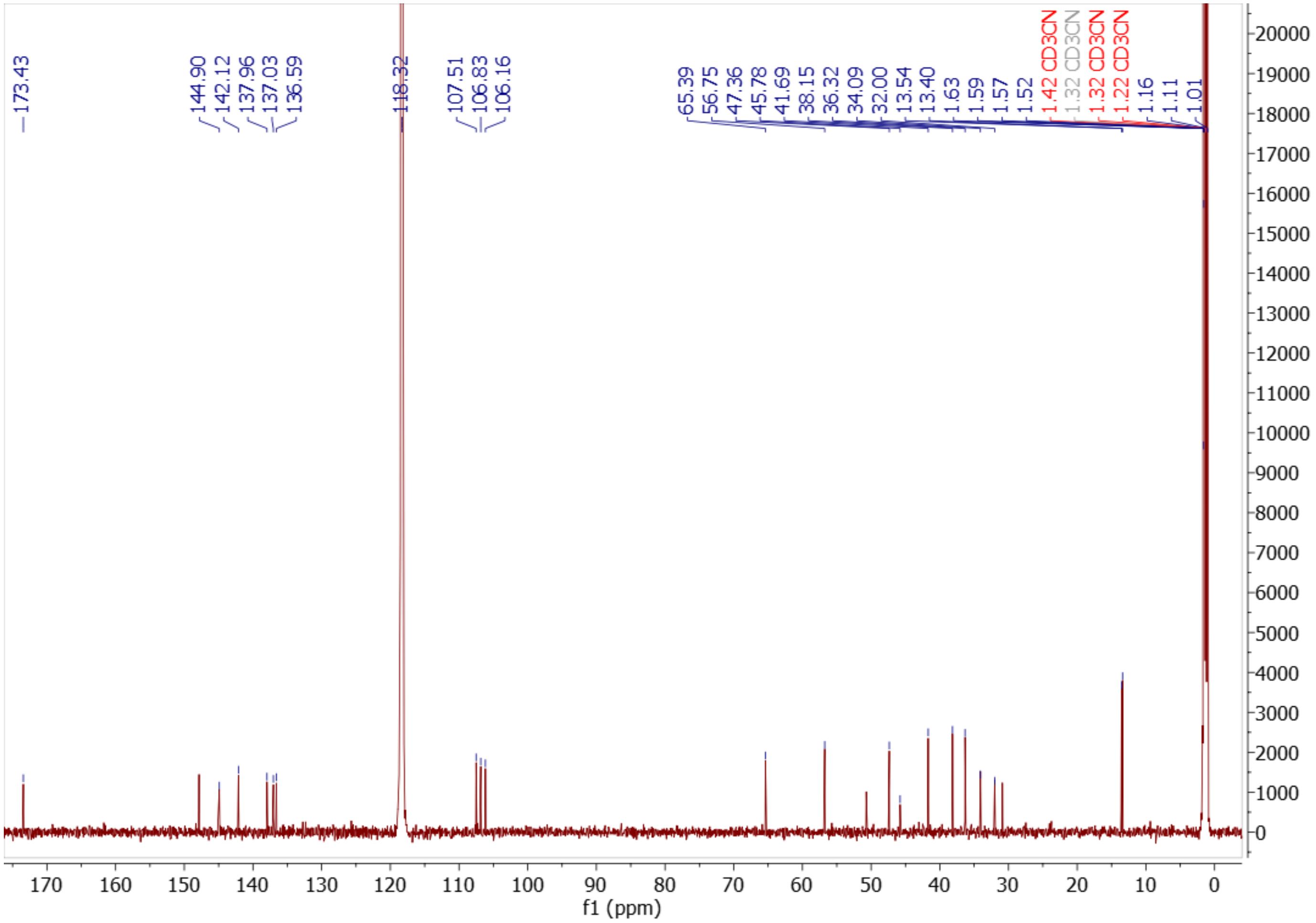

Supplementary Fig. 2: ^{13}C-NMR (CD3CN) of Compound **5**.

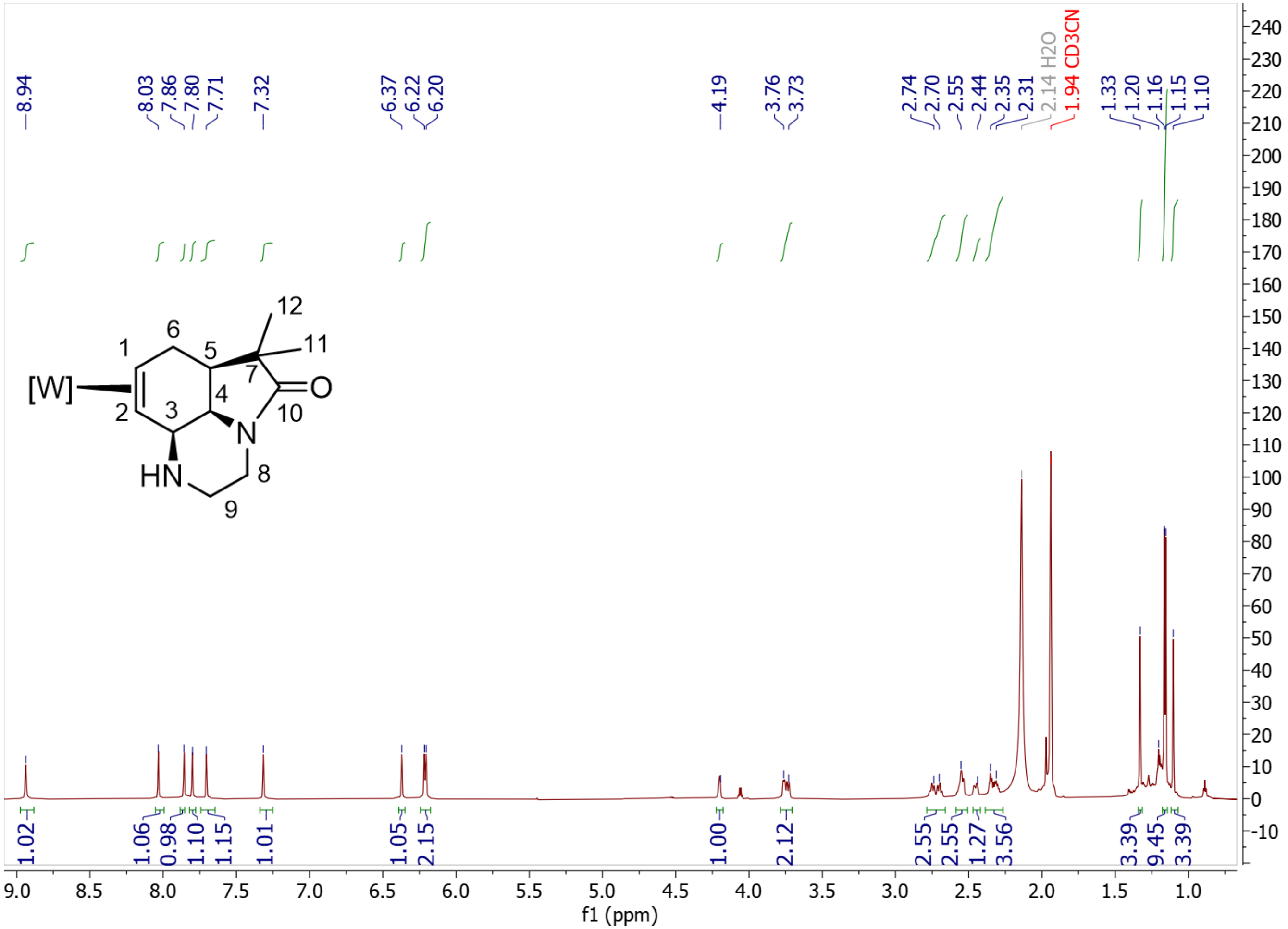

Supplementary Fig. 3: ^{1}H-NMR (CD3CN) of Compound **6**.

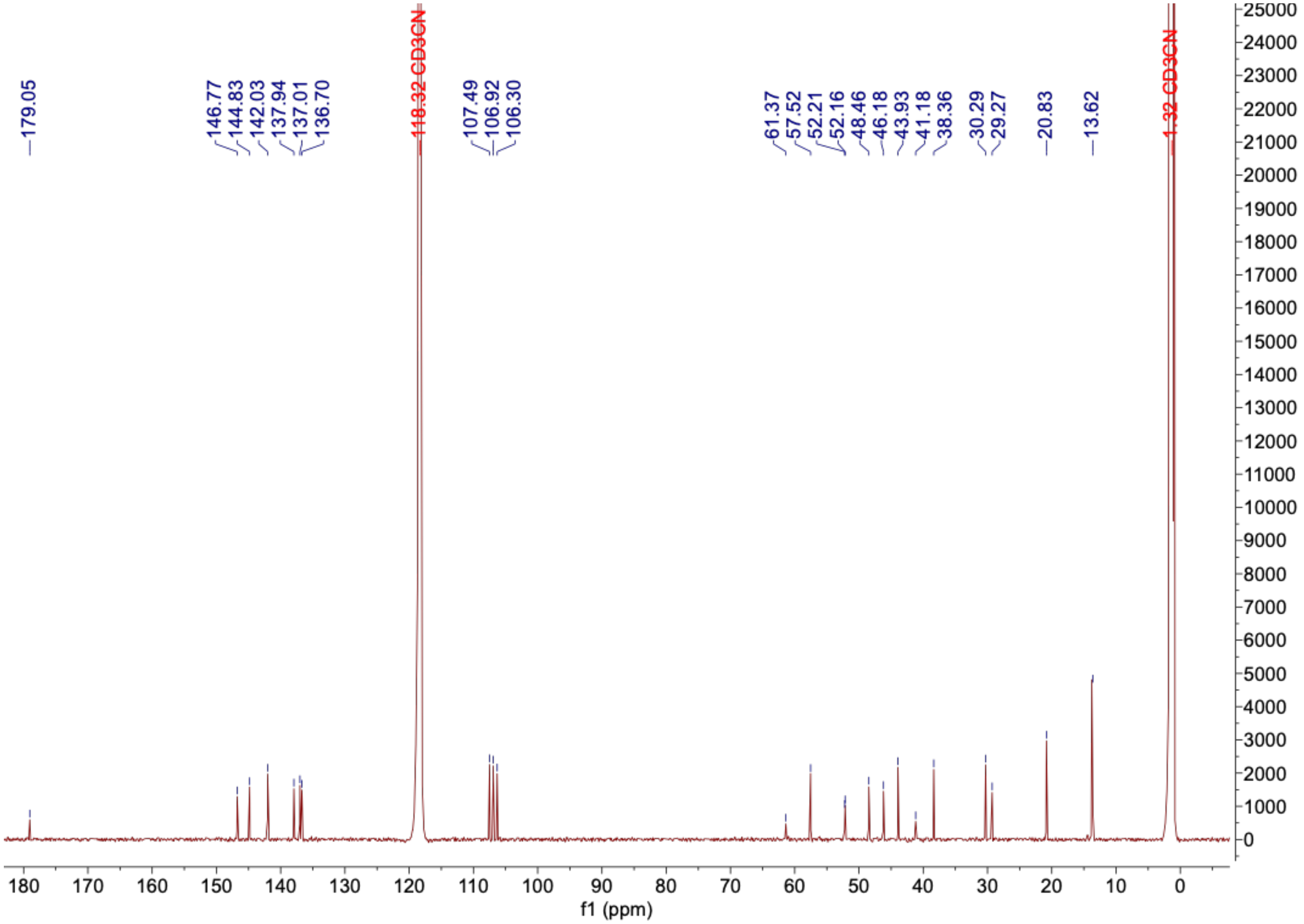

Supplementary Fig. 4: ^{13}C-NMR (CD3CN) of Compound **7**.

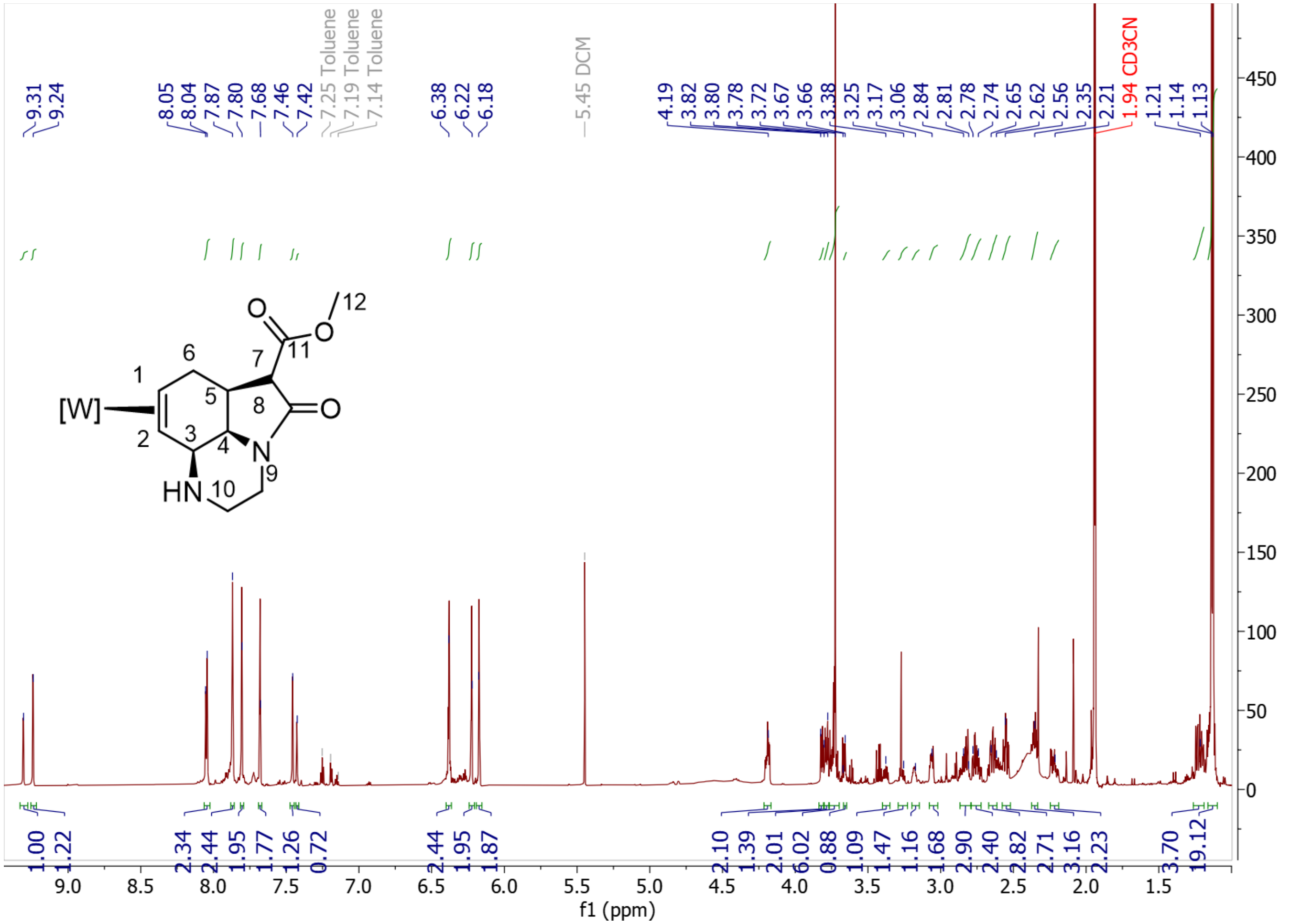

Supplementary Fig. 5: ^{1}H-NMR (CD3CN) of Compound **7**.
2 epimers are observed in the NMR spectra.

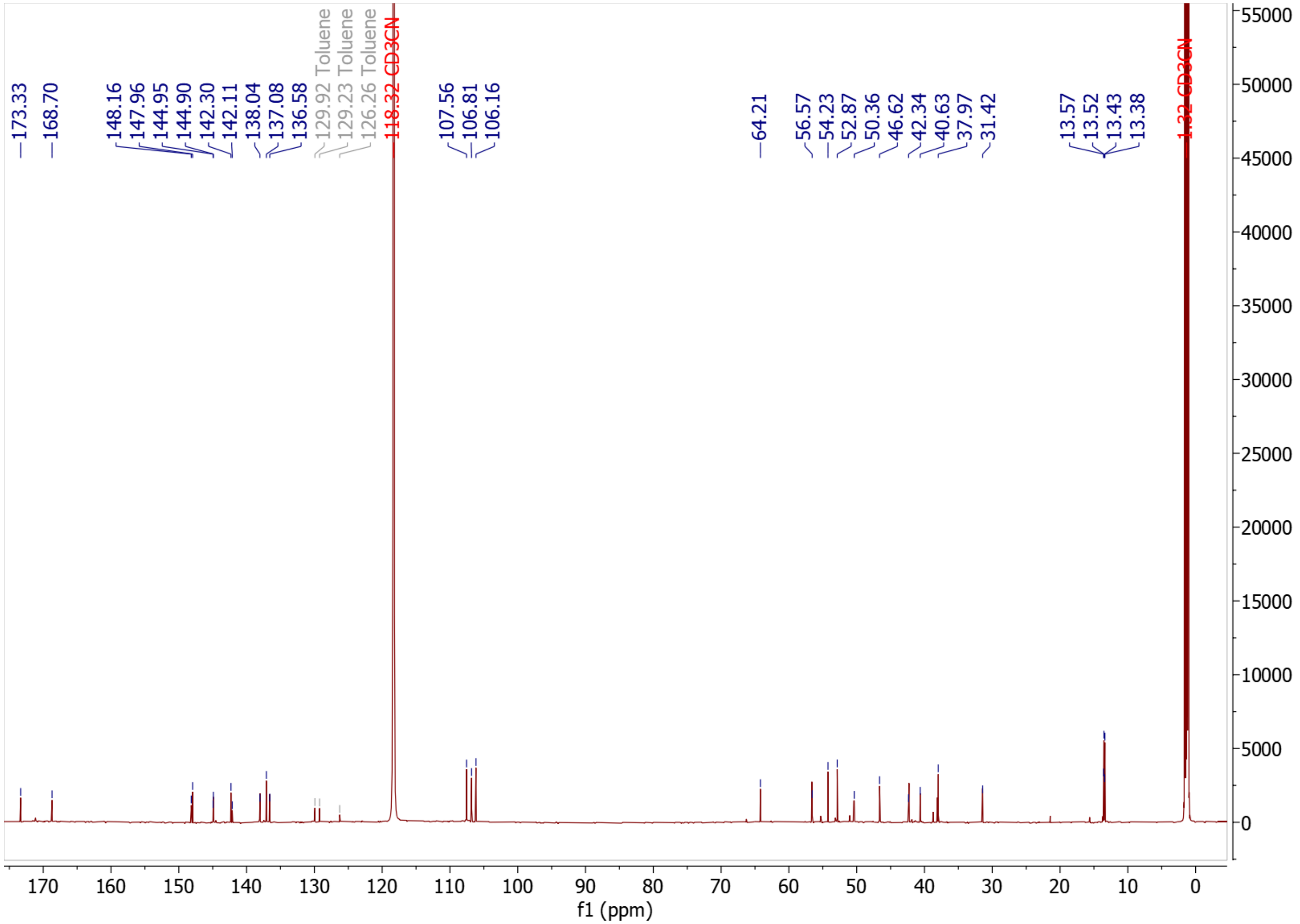

Supplementary Fig. 6: ^{13}C-NMR (CD3CN) of Compound **7**.
2 epimers are observed in the NMR spectra.

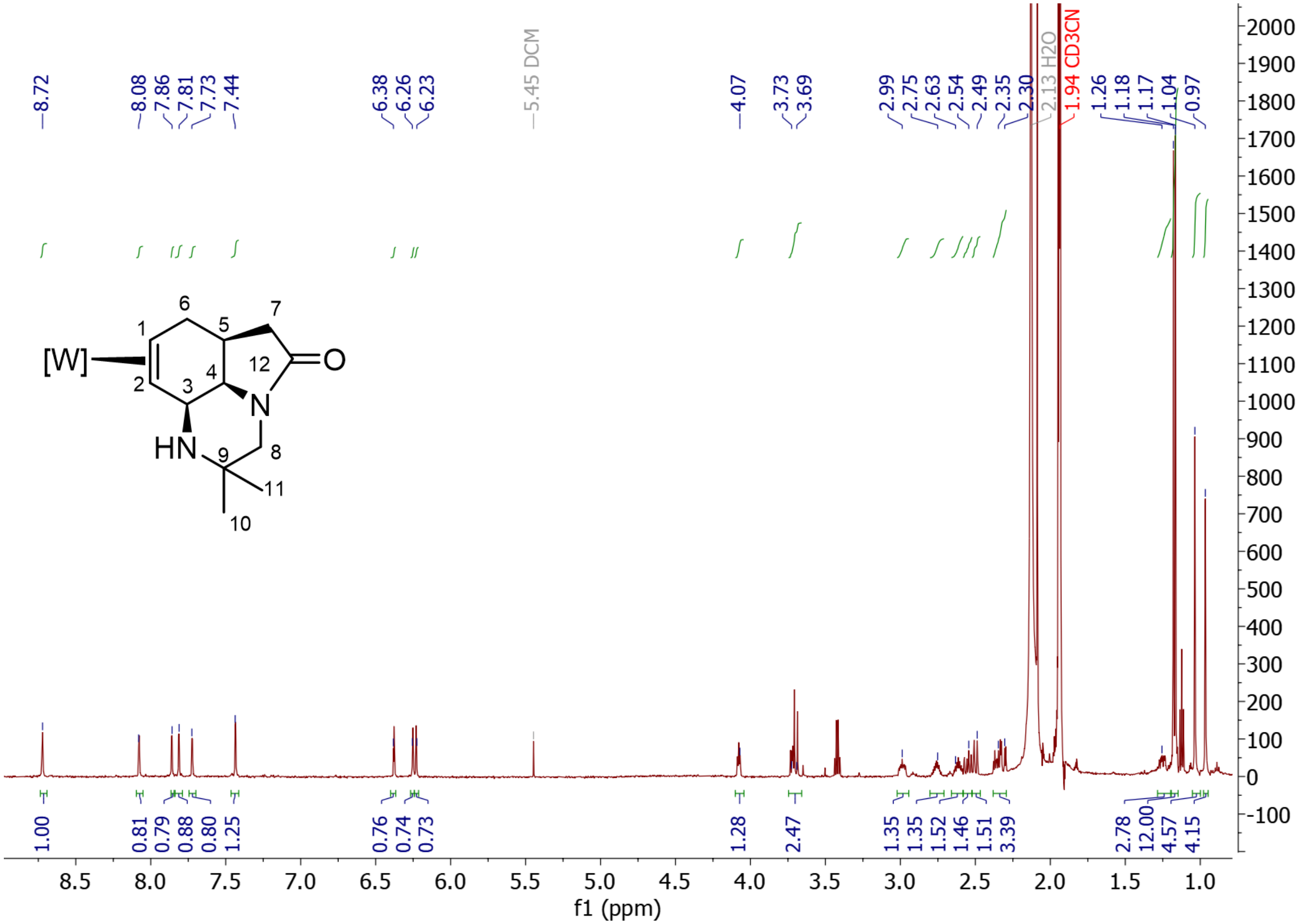

Supplementary Fig. 7: ^{1}H-NMR (CD3CN) of Compound **8**.

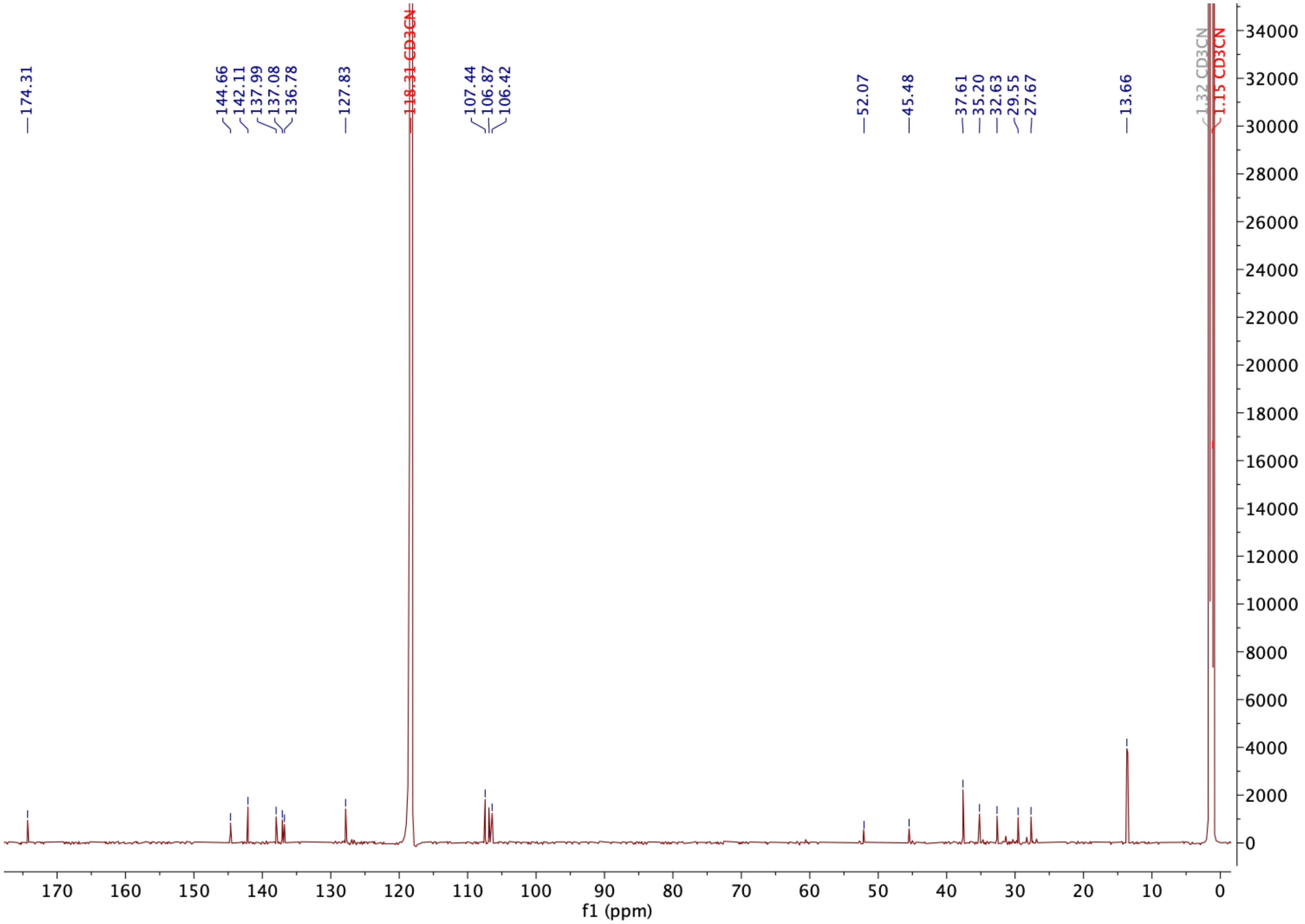

Supplementary Fig. 8: ^{13}C-NMR (CD3CN) of Compound **8**.

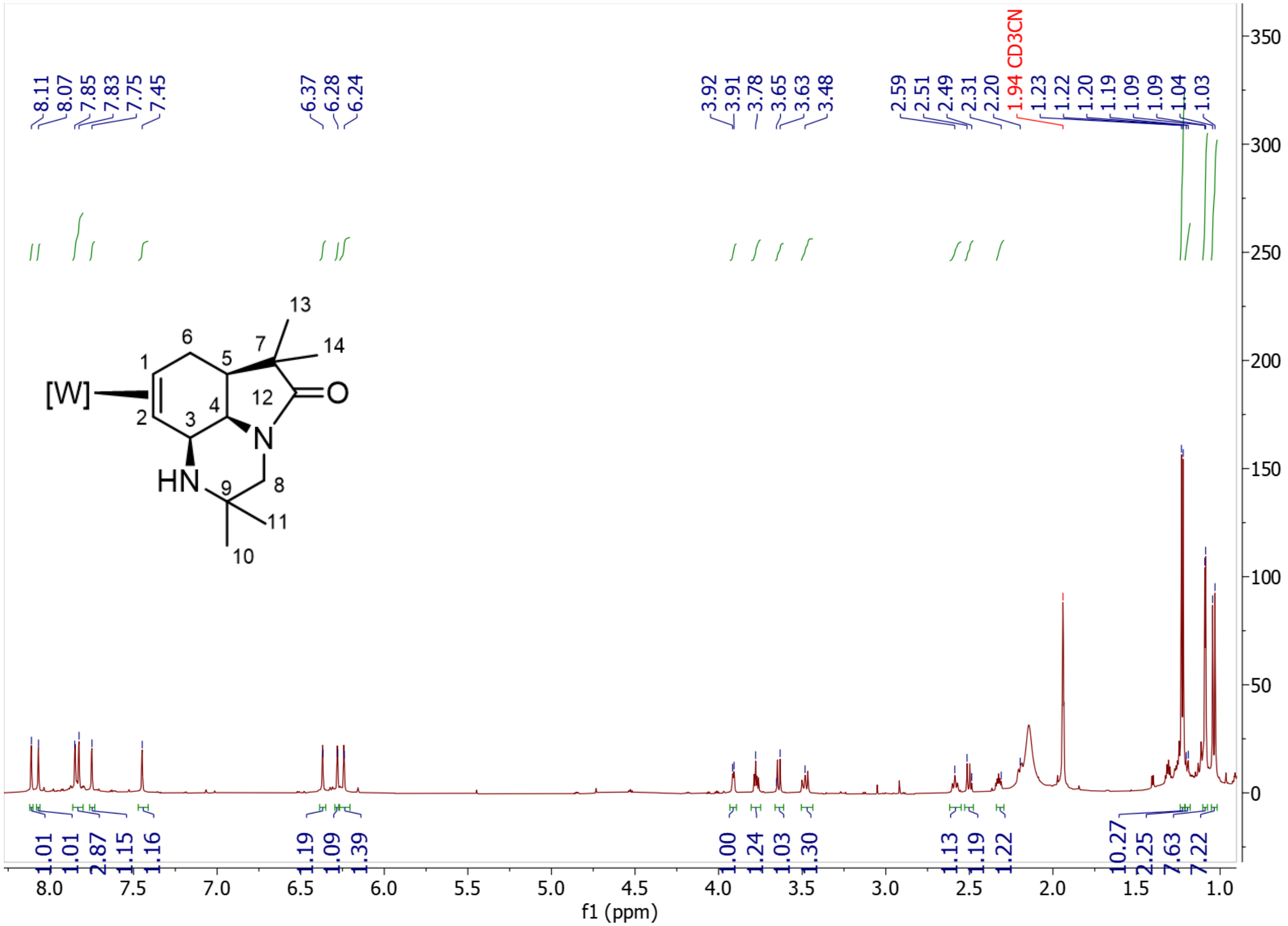

Supplementary Fig. 9: ^{1}H-NMR (CD3CN) of Compound **9**.

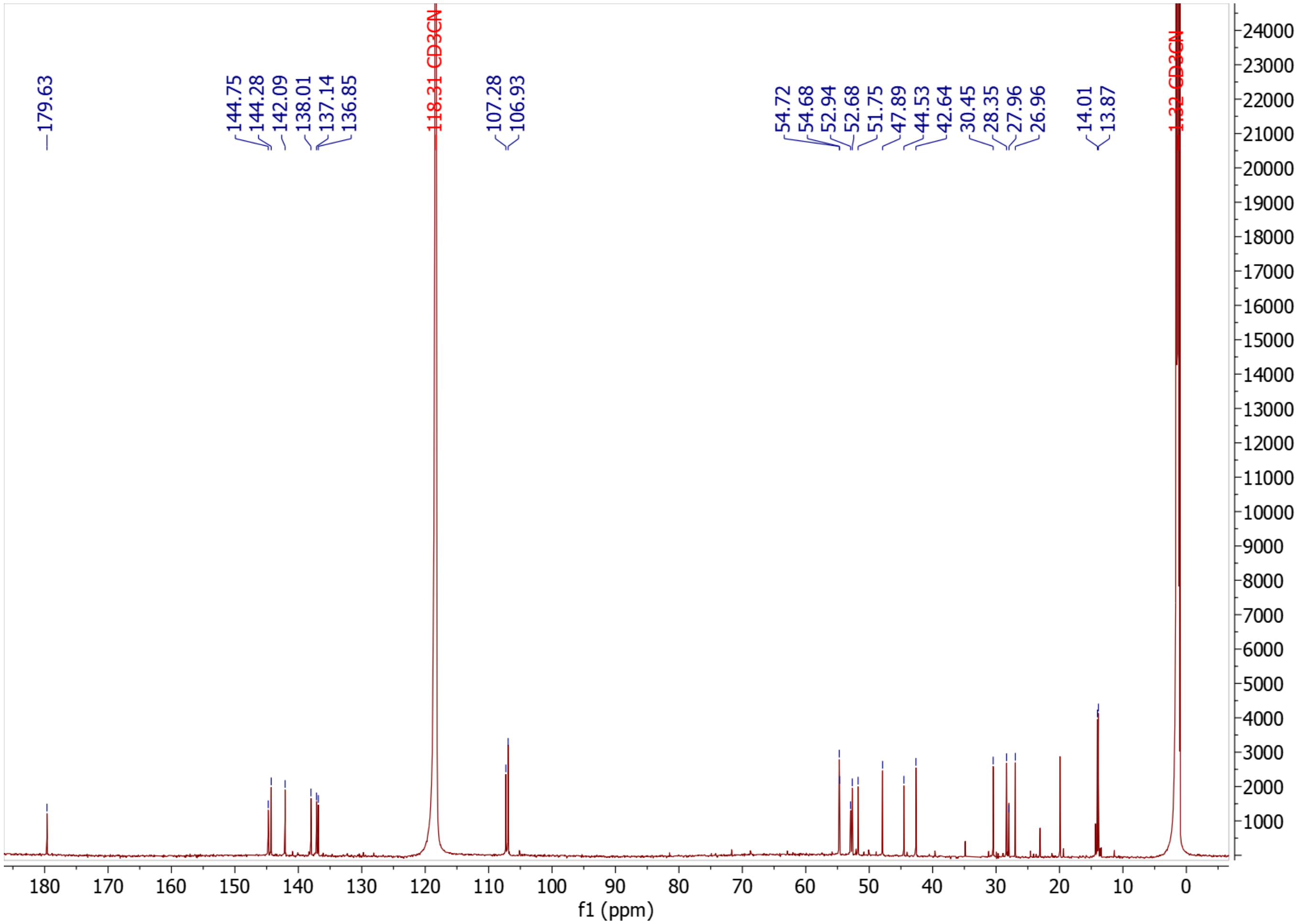

Supplementary Fig. 10: ^{13}C-NMR (CD3CN) of Compound **9**.

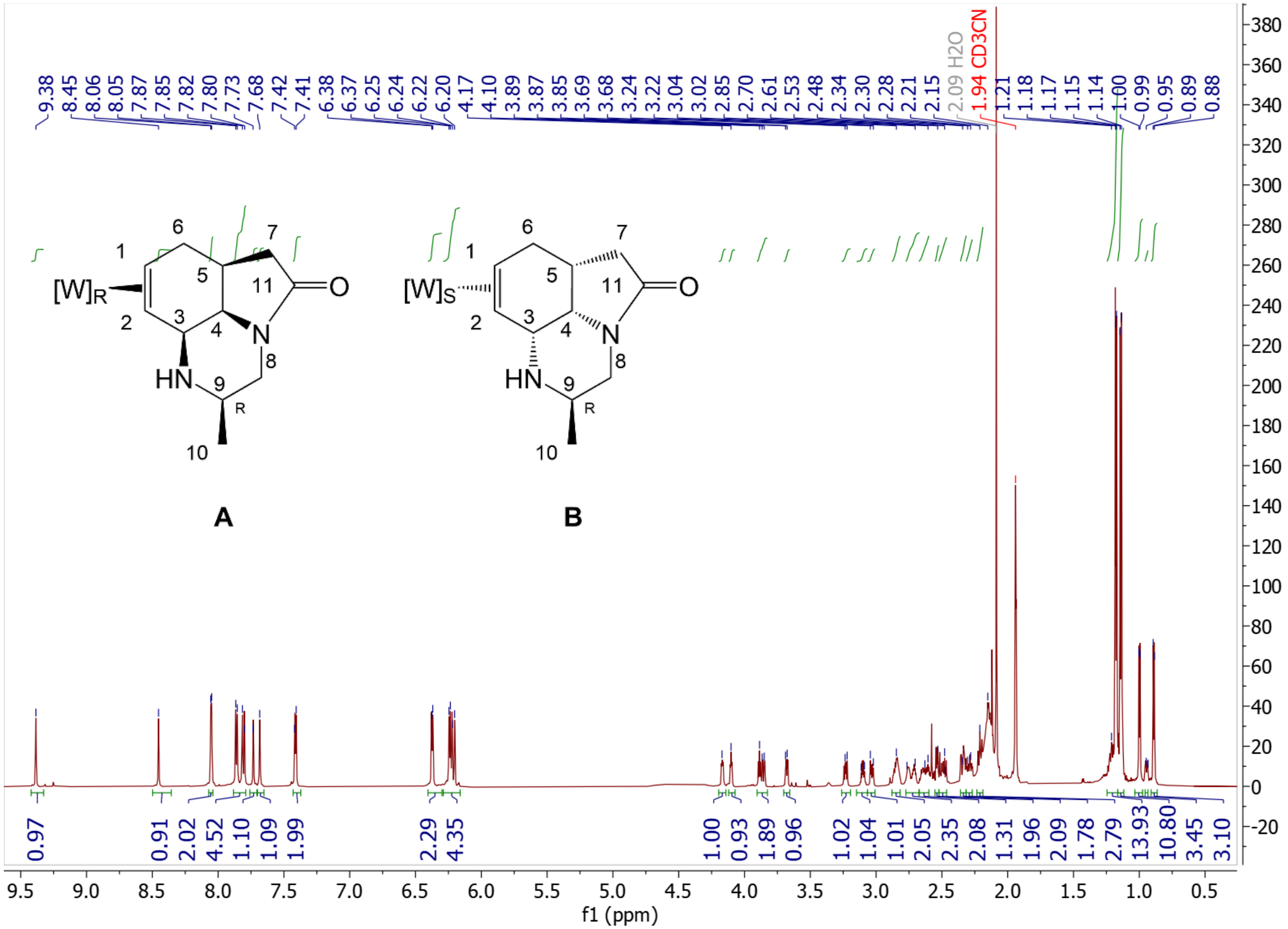

Supplementary Fig. 11: ^{1}H-NMR (CD3CN) of Compound **10**.

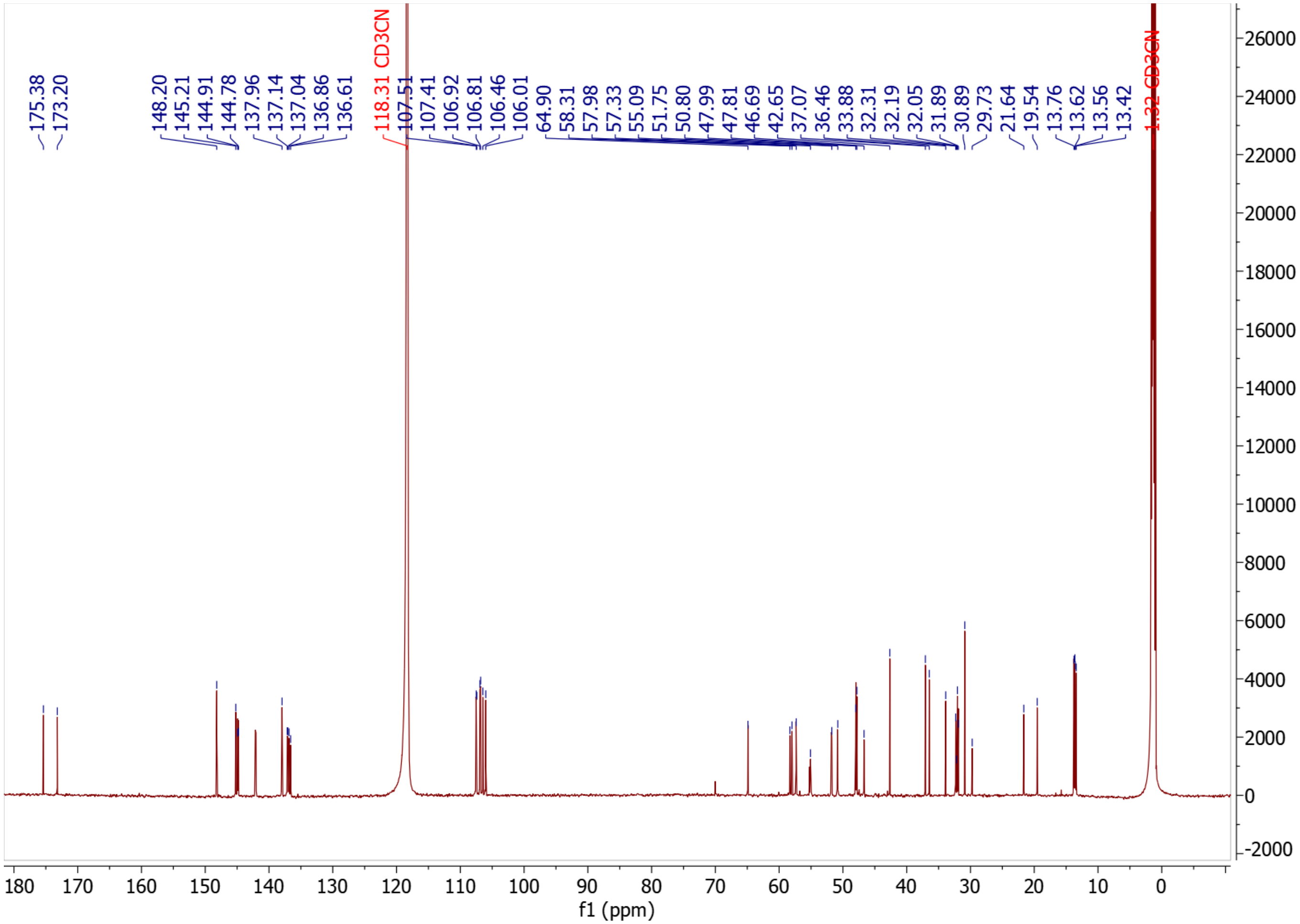

Supplementary Fig. 12: ^{13}C-NMR (CD3CN) of Compound **10**.

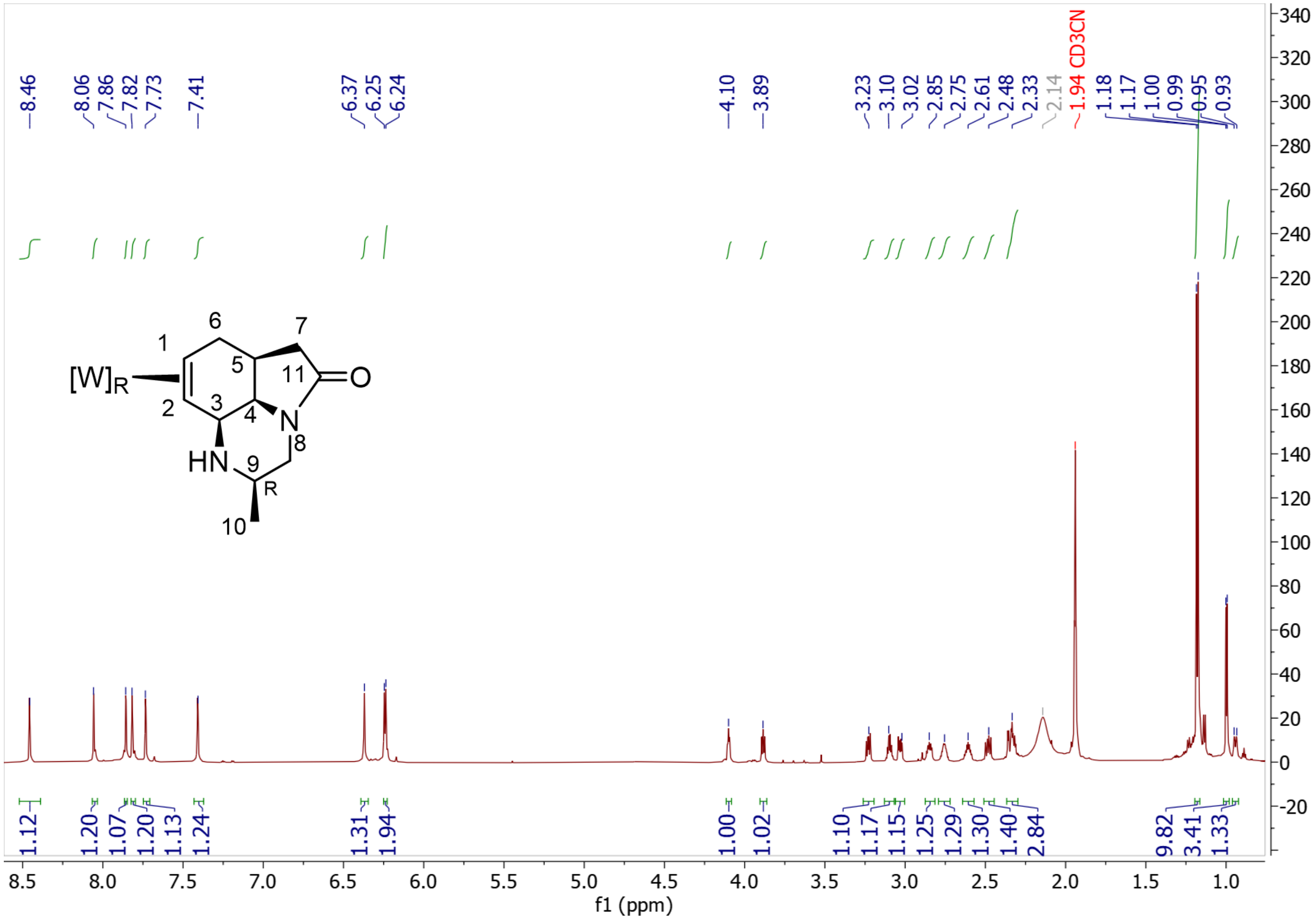

Supplementary Fig. 13: ^{1}H-NMR (CD3CN) of Compound **11**.

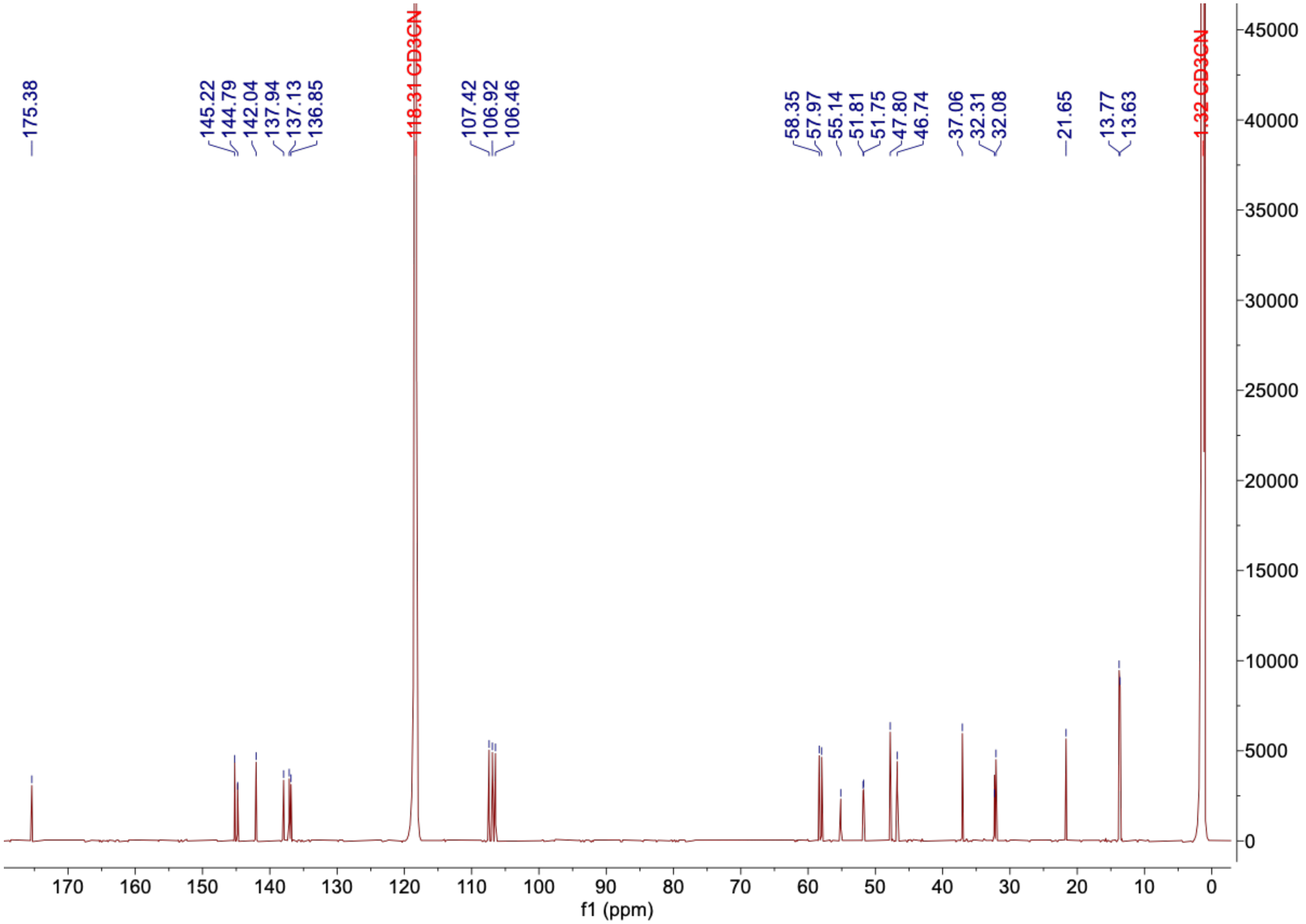

Supplementary Fig. 14: ^{13}C-NMR (CD3CN) of Compound **11**.

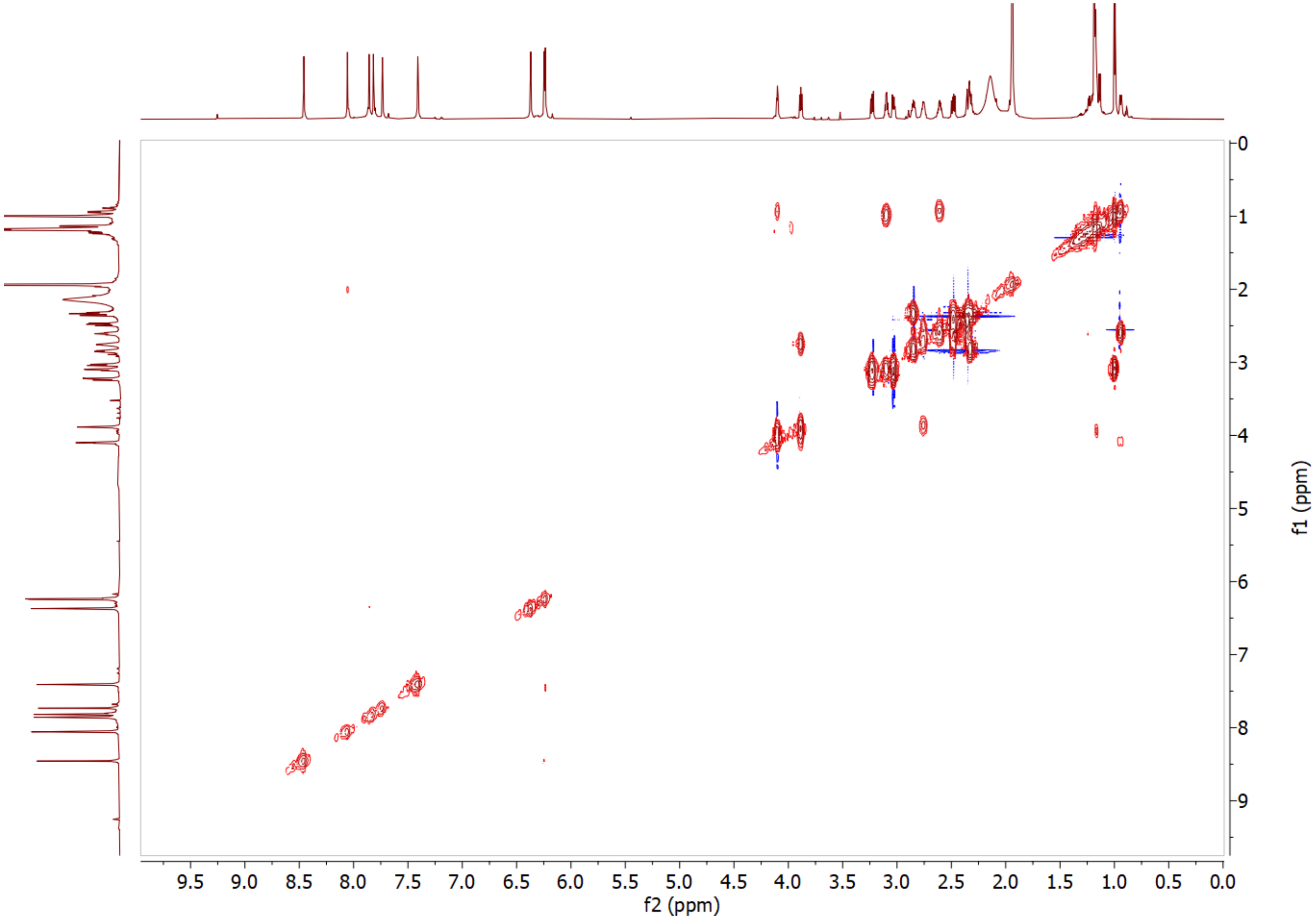

Supplementary Fig. 15: ^{1}H-^{1}H COSY (CD3CN) of Compound **11**.

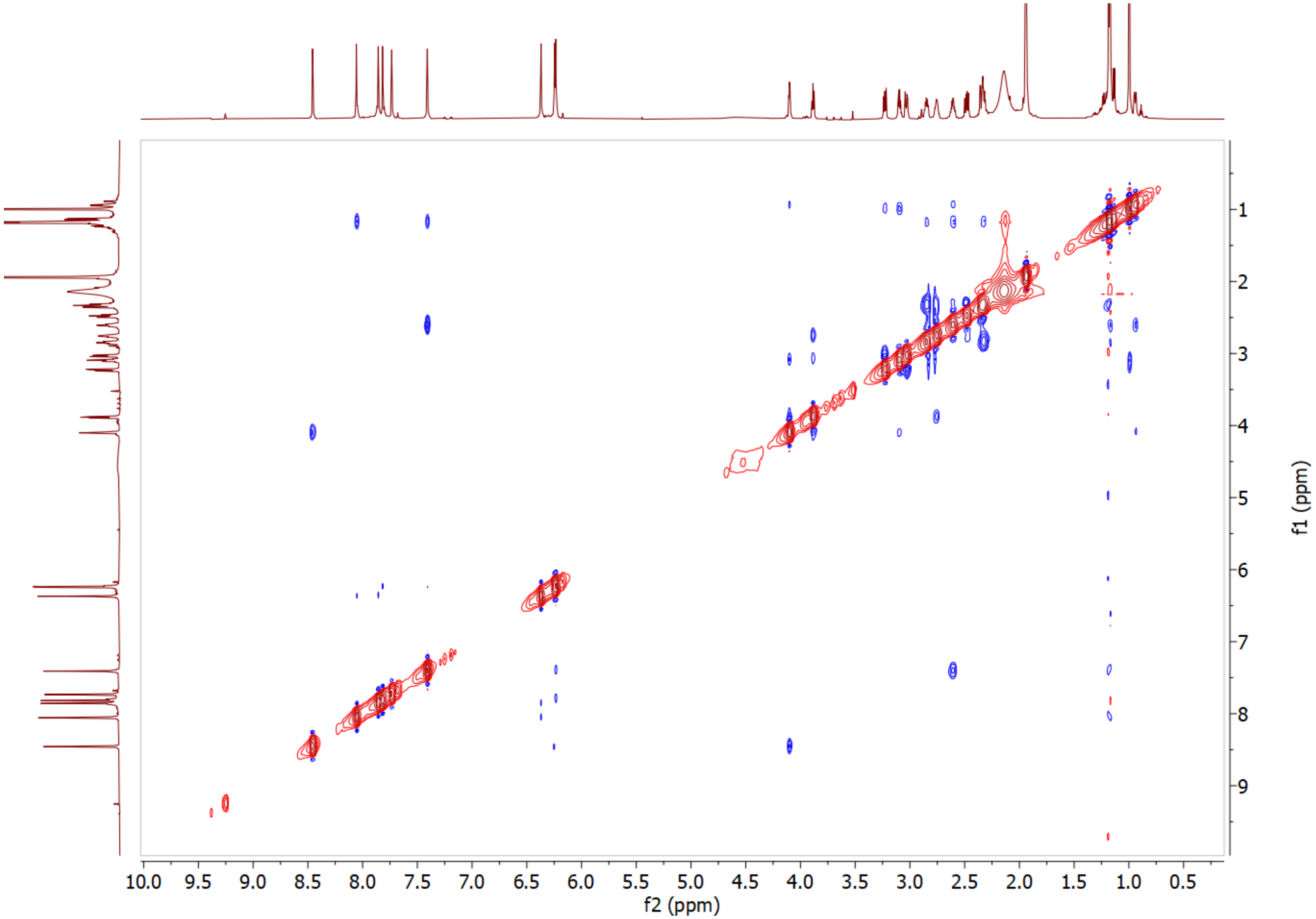

Supplementary Fig. 16: ^{1}H-^{1}H NOESY (CD3CN) of Compound **11**.

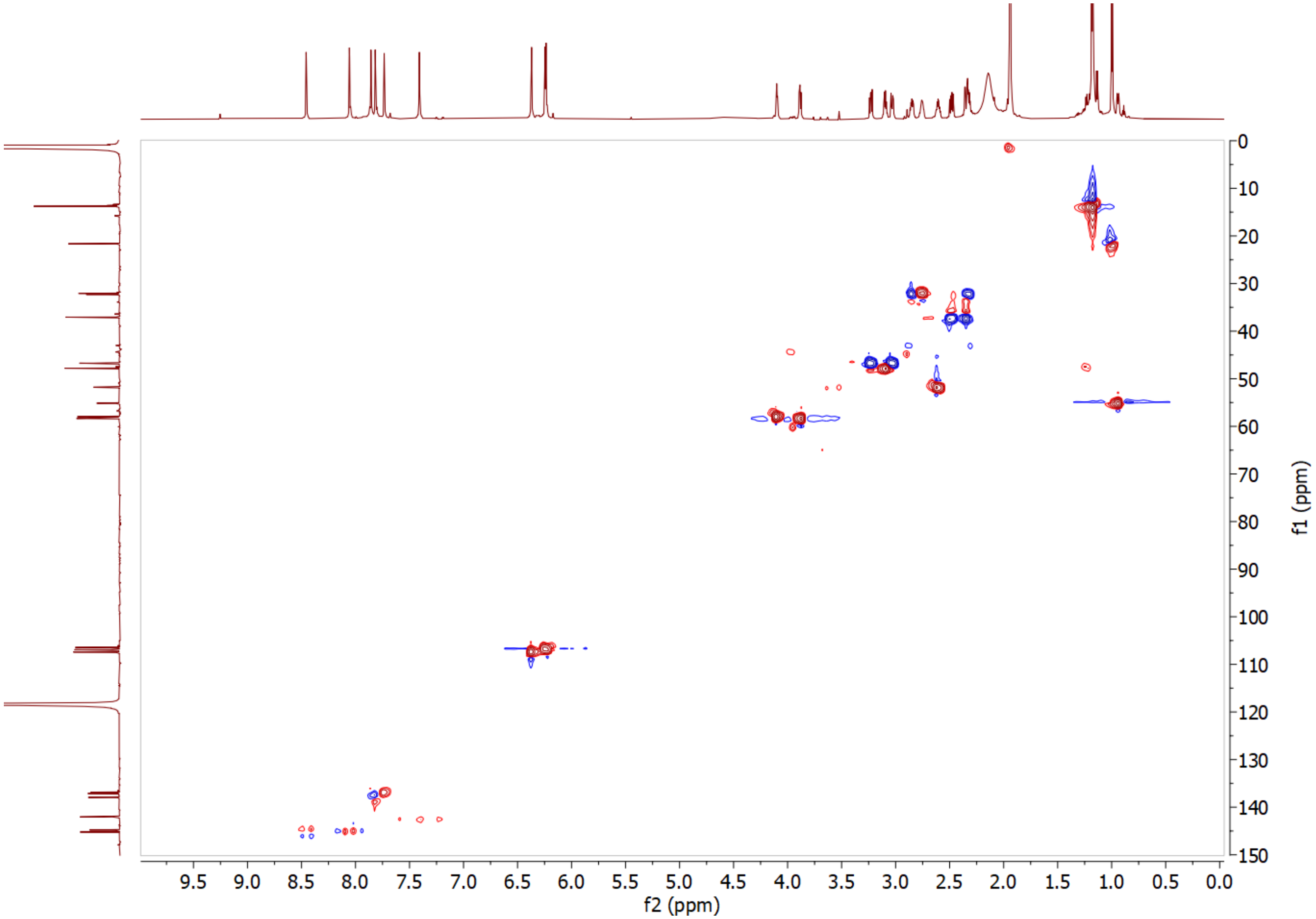

Supplementary Fig. 17: ^{1}H-^{13}C HSQC (CD3CN) of Compound **11**.

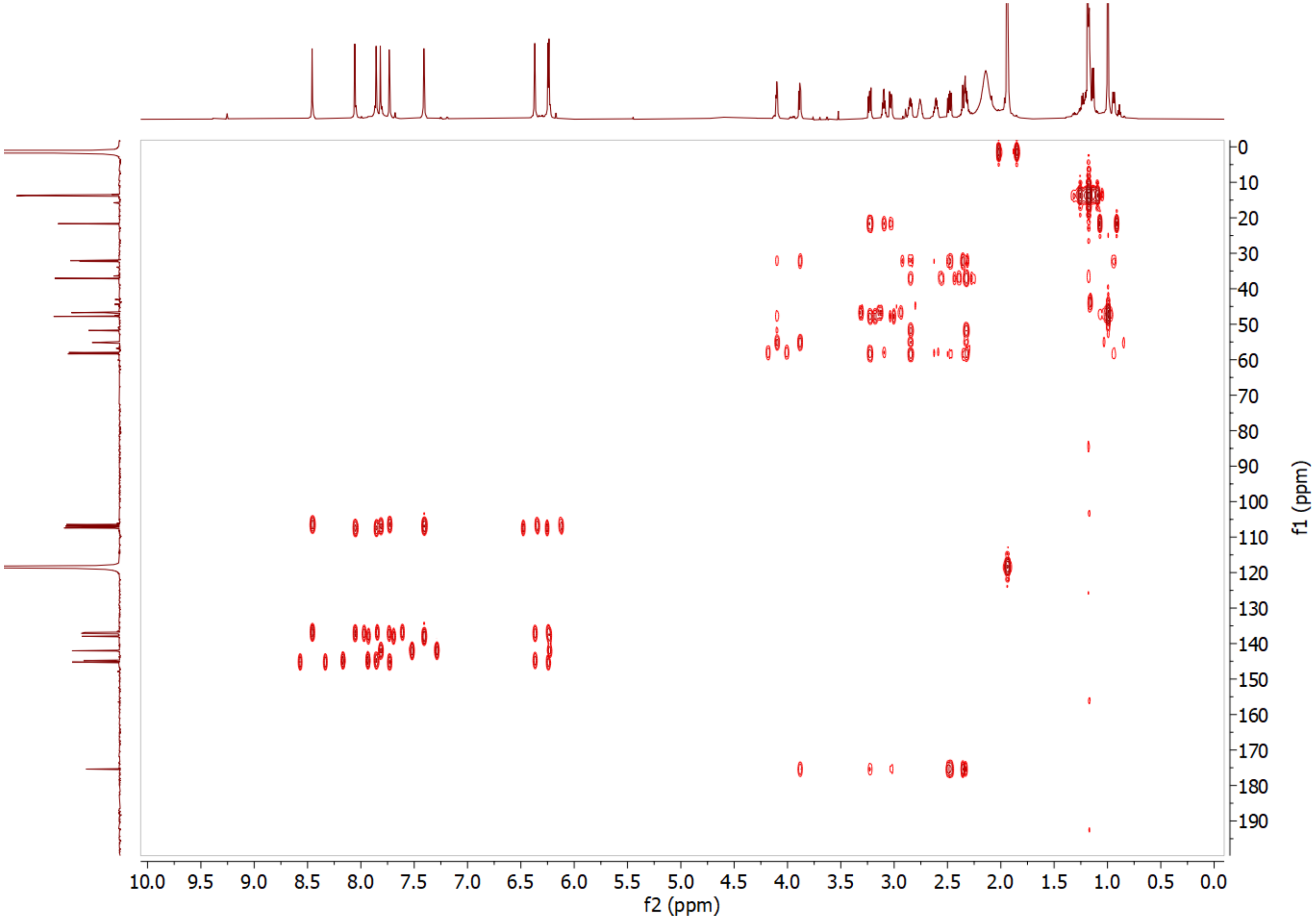

Supplementary Fig. 18: ^{1}H-^{13}C HMBC (CD3CN) of Compound **11**.

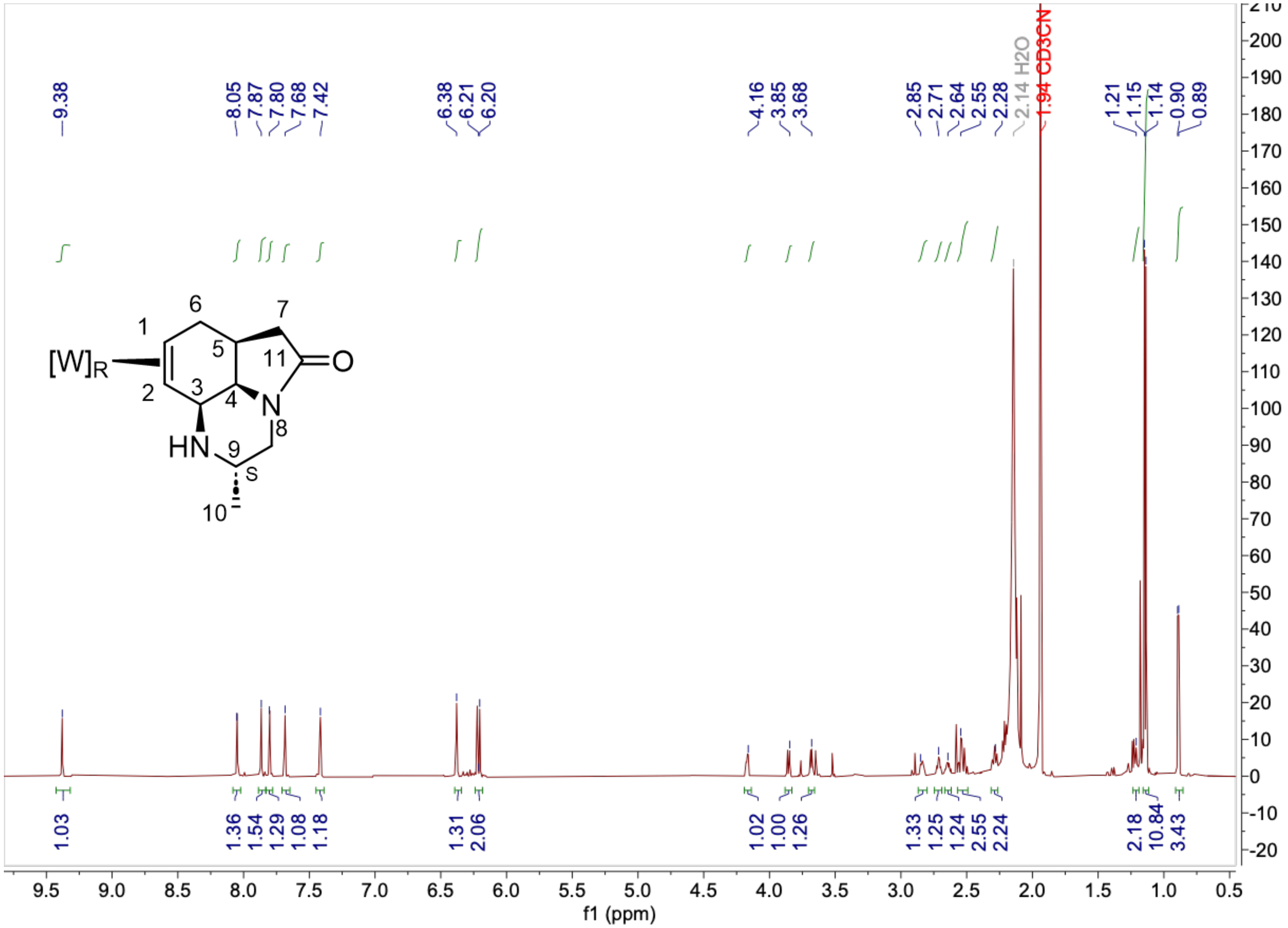

Supplementary Fig. 19: ^{1}H-NMR (CD3CN) of Compound **12**.

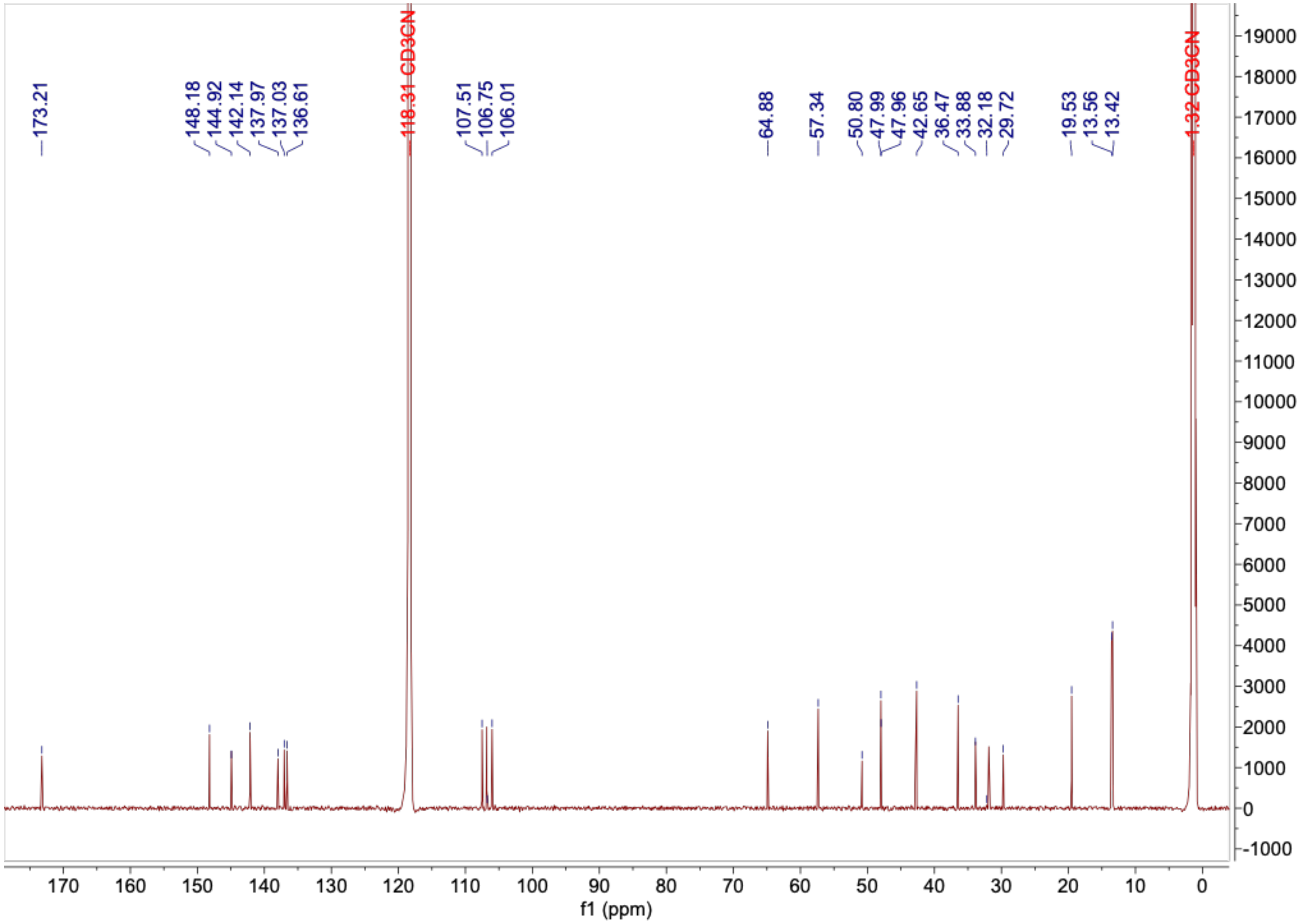

Supplementary Fig. 20: ^{13}C-NMR (CD3CN) of Compound **12**.

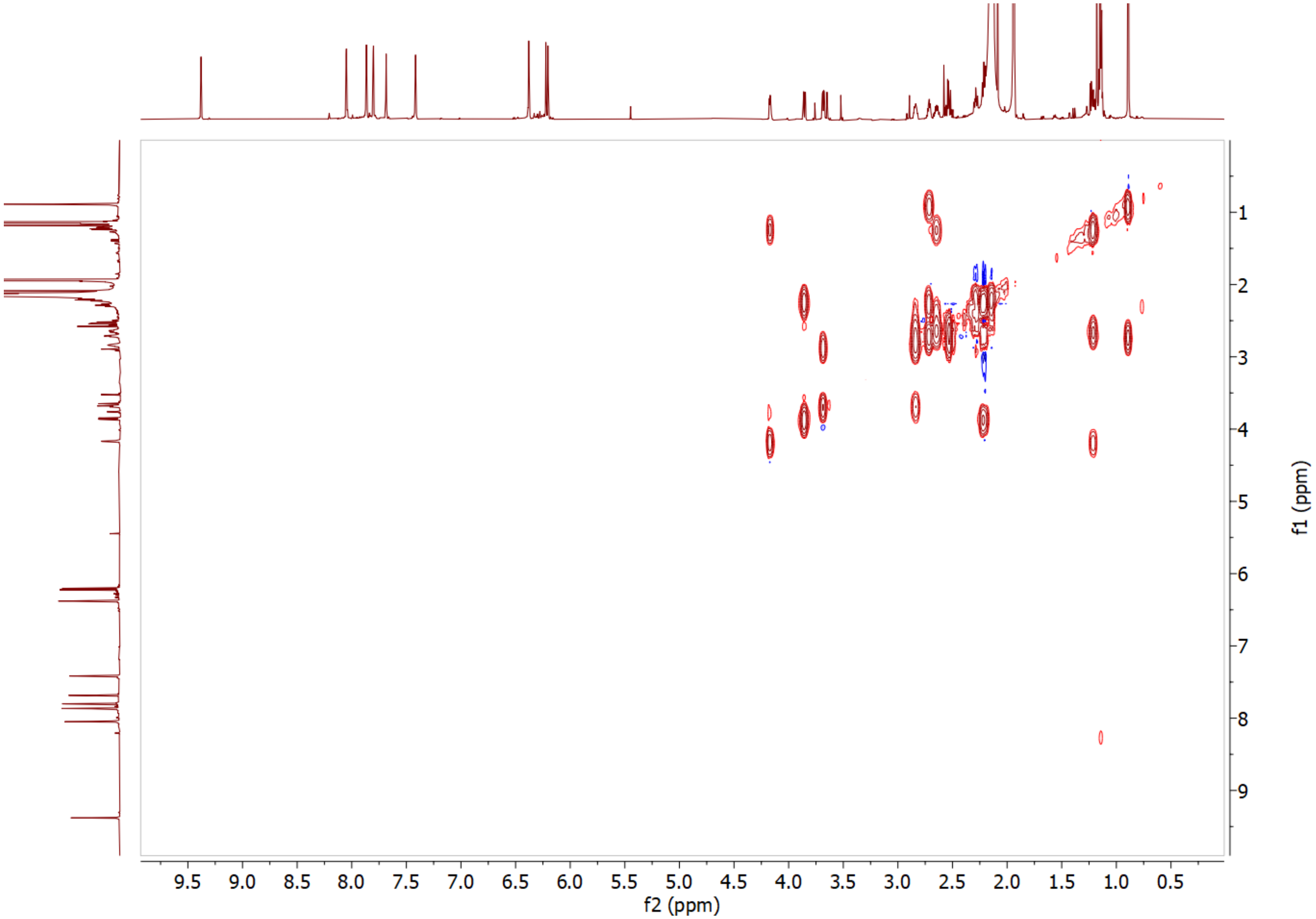

Supplementary Fig. 21: ^{1}H-^{1}H COSY (CD3CN) of Compound **12**.

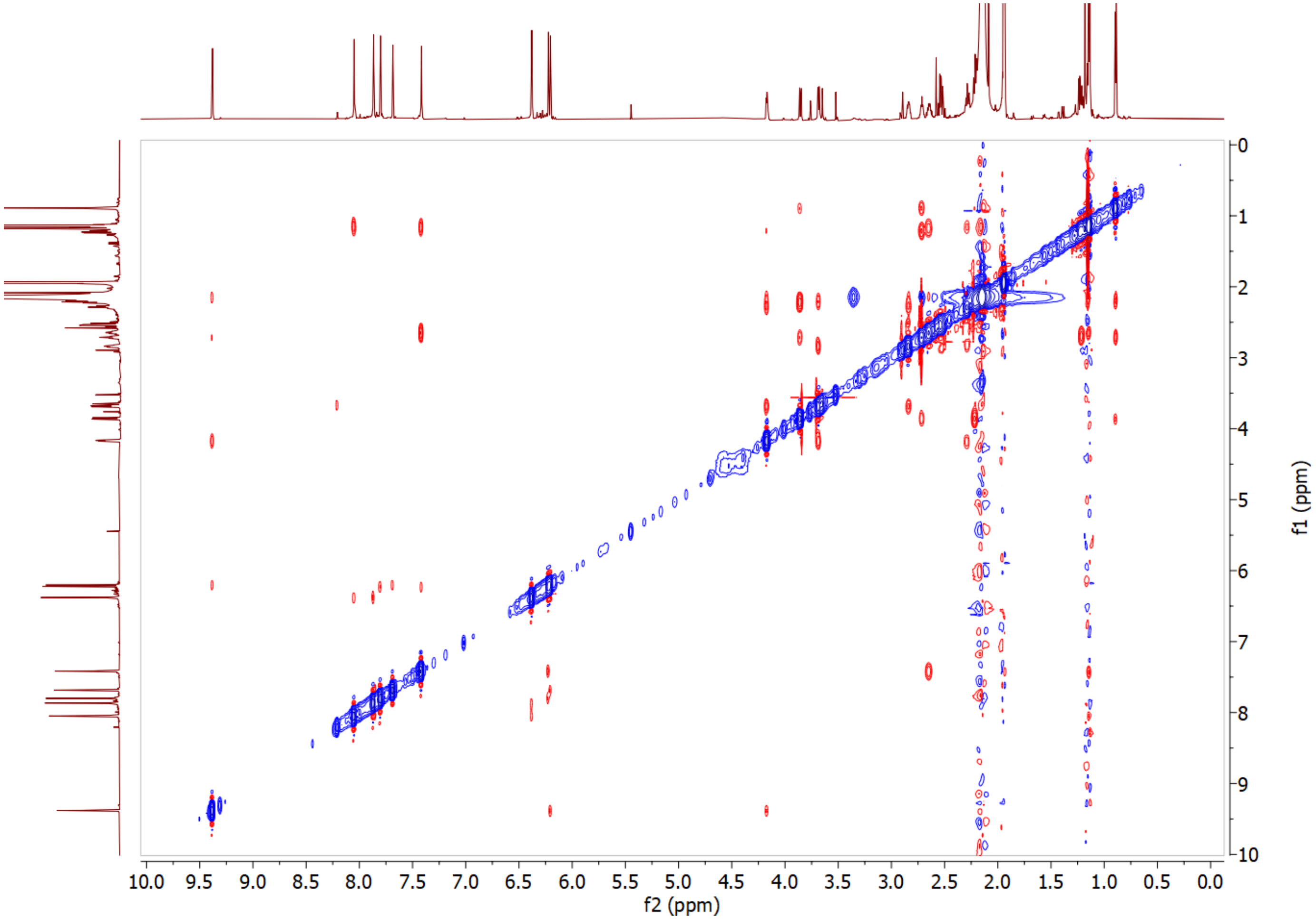

Supplementary Fig. 22: ^{1}H-^{1}H NOESY (CD3CN) of Compound **12**.

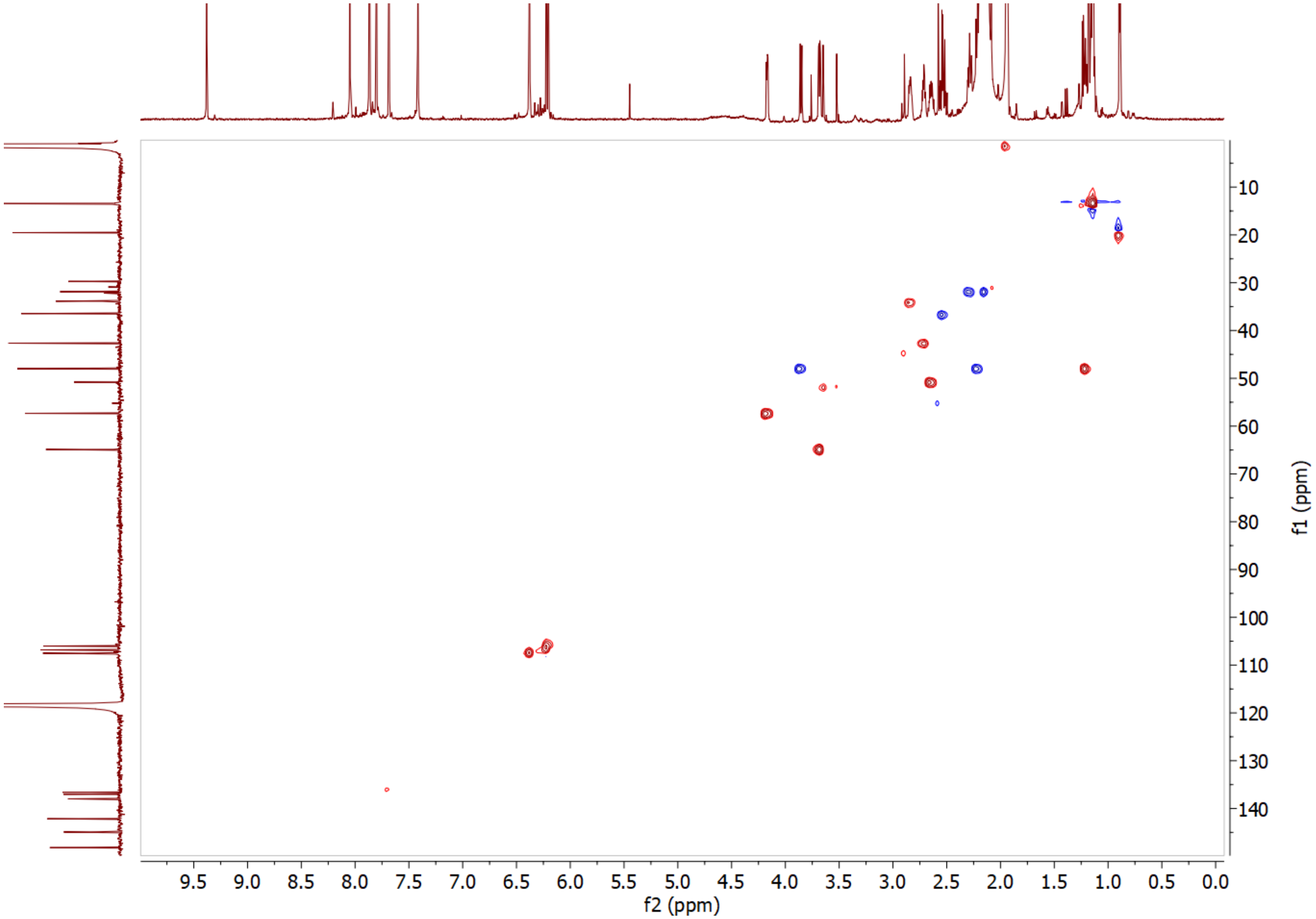

Supplementary Fig. 23: ^{1}H-^{13}C HSQC (CD3CN) of Compound **12**.

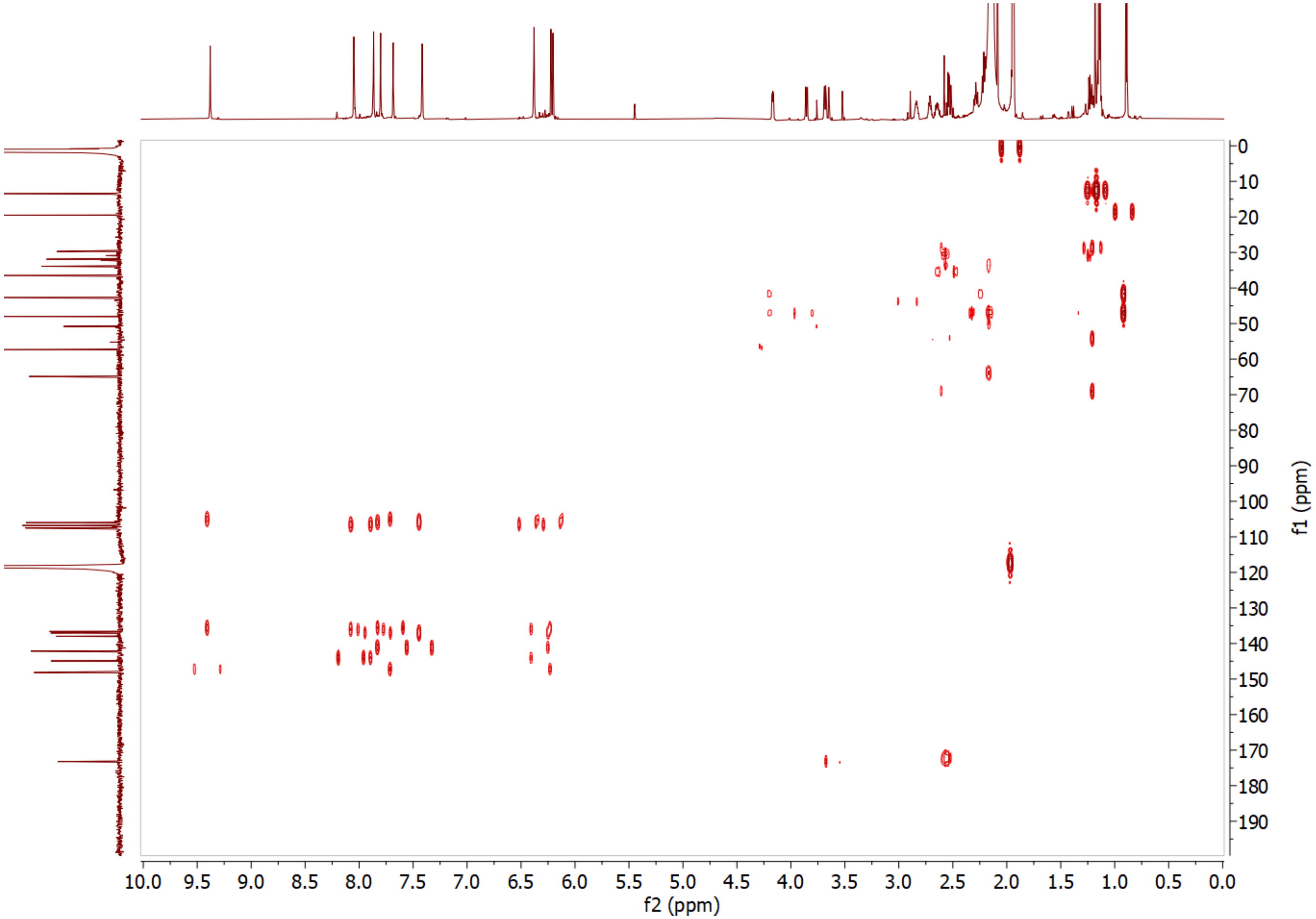

Supplementary Fig. 24: ^{1}H-^{13}C HMBC (CD3CN) of Compound **12**.

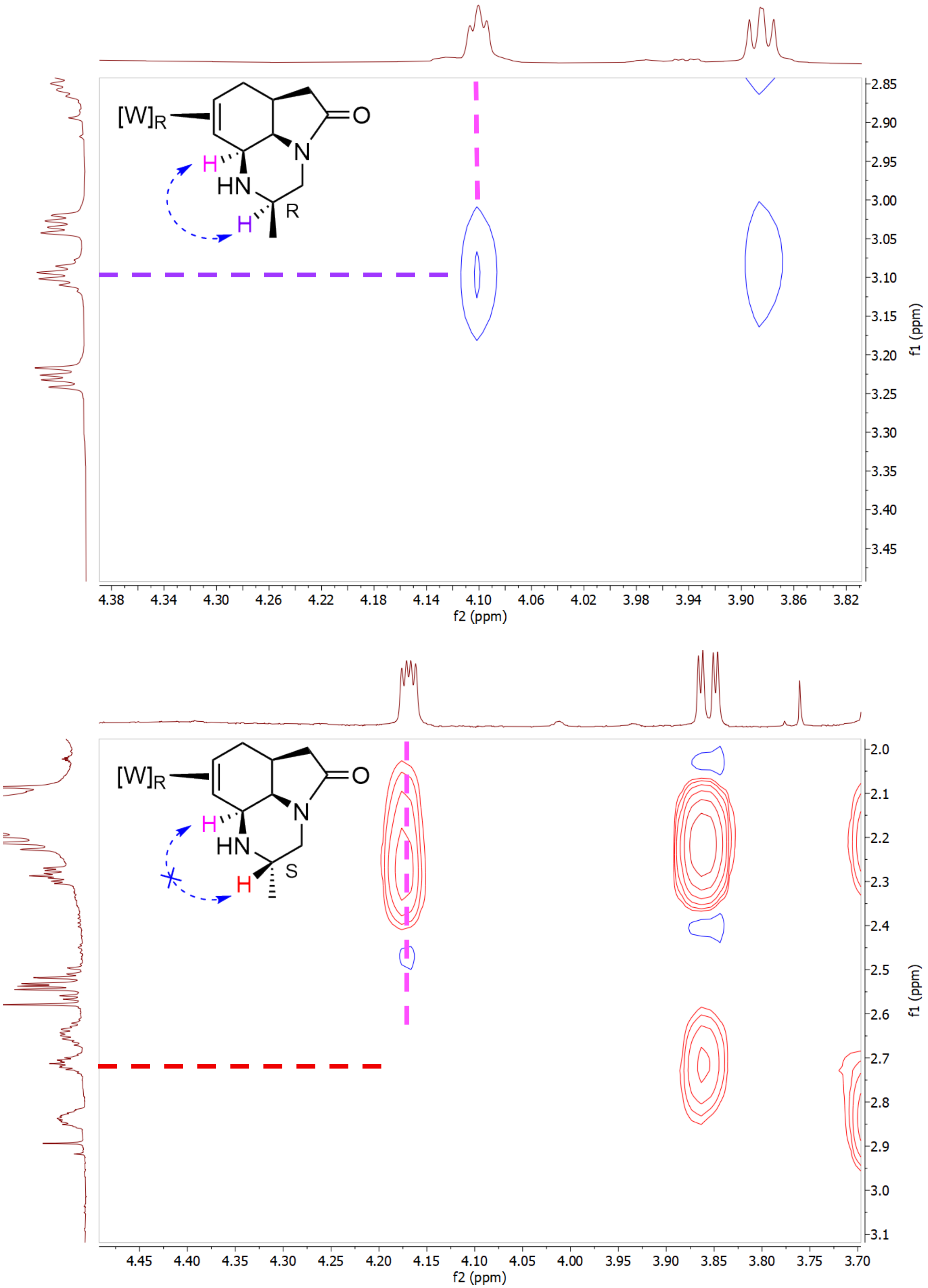

Supplementary Fig. 25: Comparison of Key NOESY interaction (CD3CN) of Compound **11** and **12**.

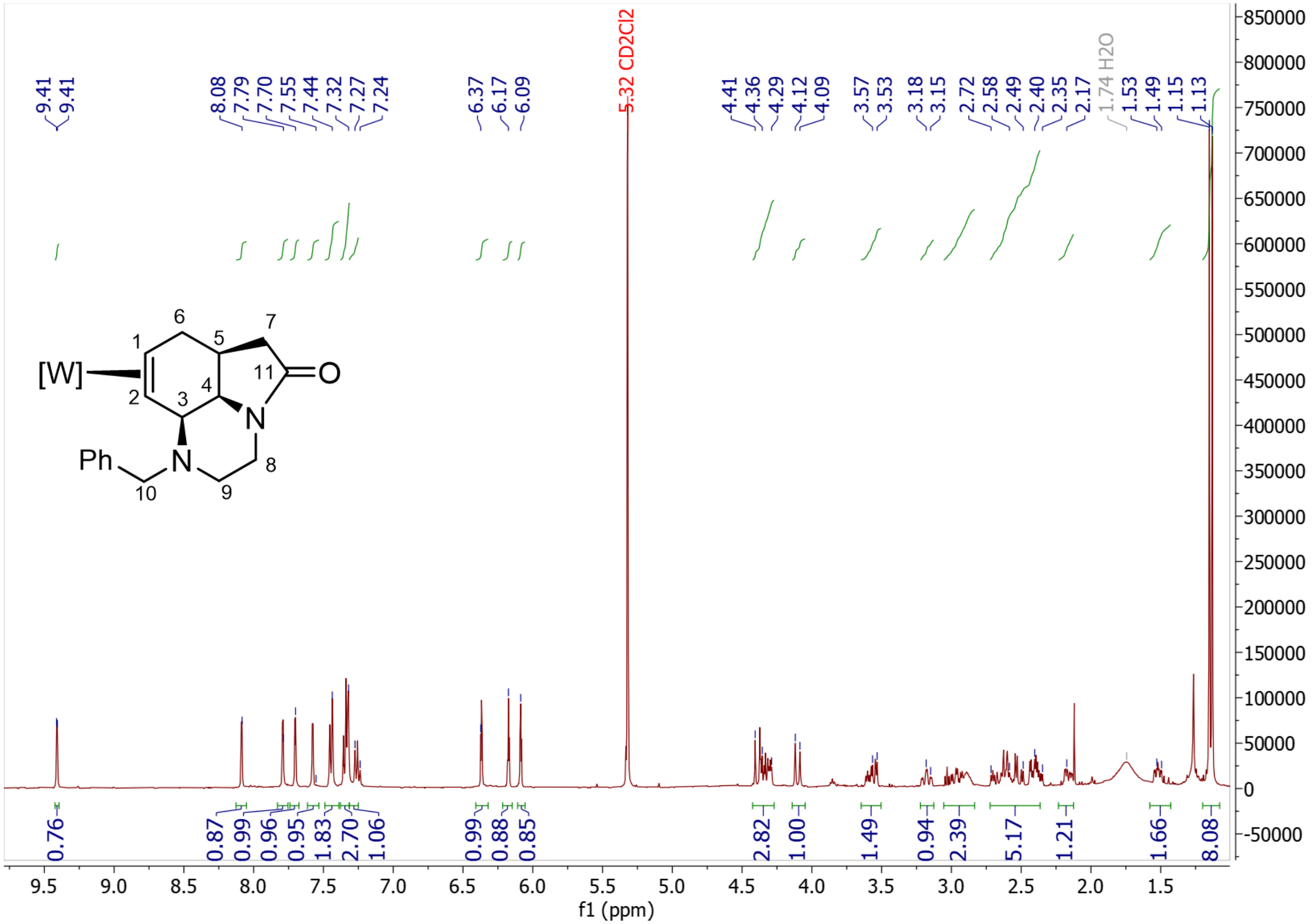

Supplementary Fig. 26: ^{1}H-NMR (CD_2Cl_2) of Compound **13**.

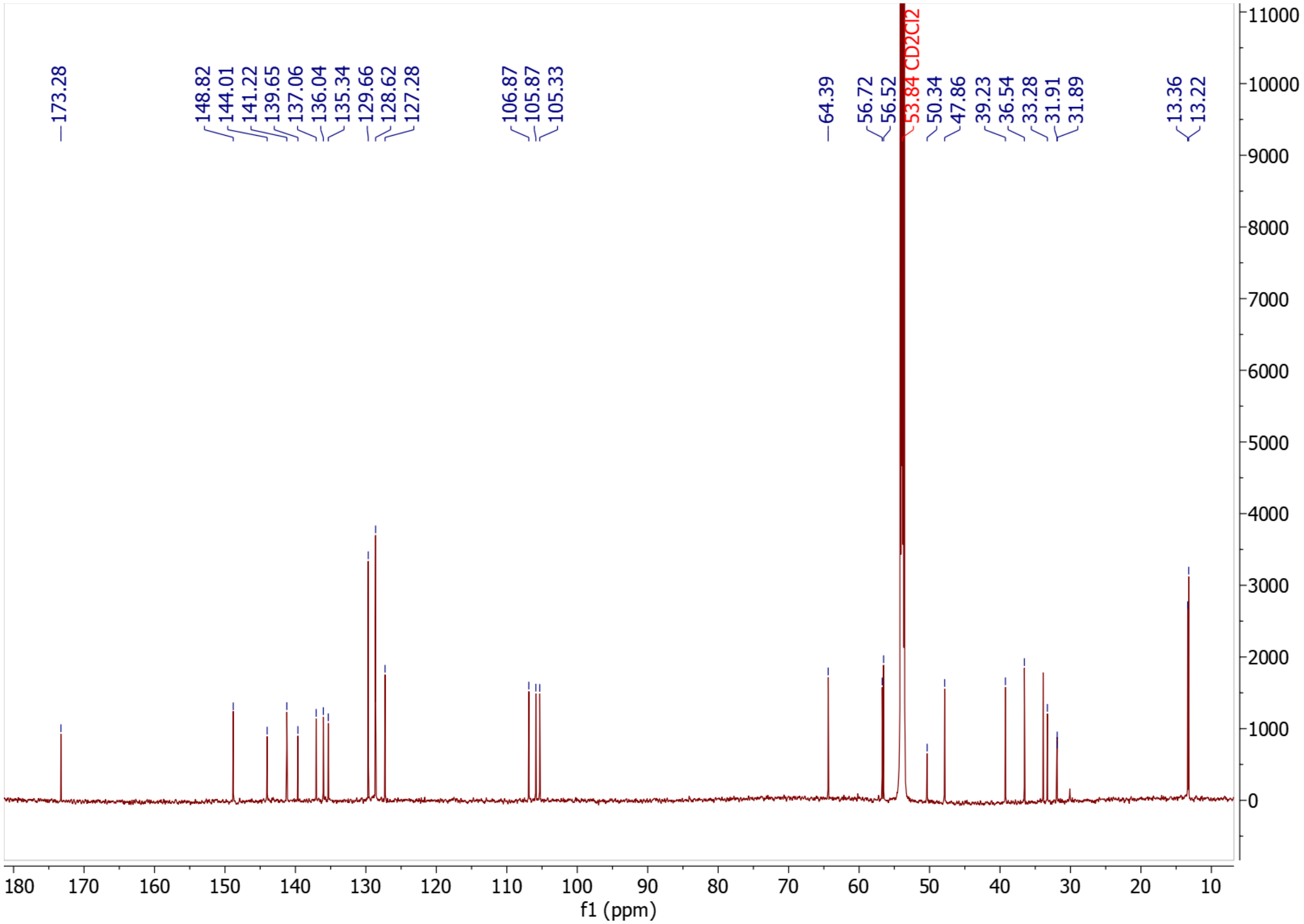

Supplementary Fig. 27: ^{13}C-NMR (CD3CN) of Compound **13**.

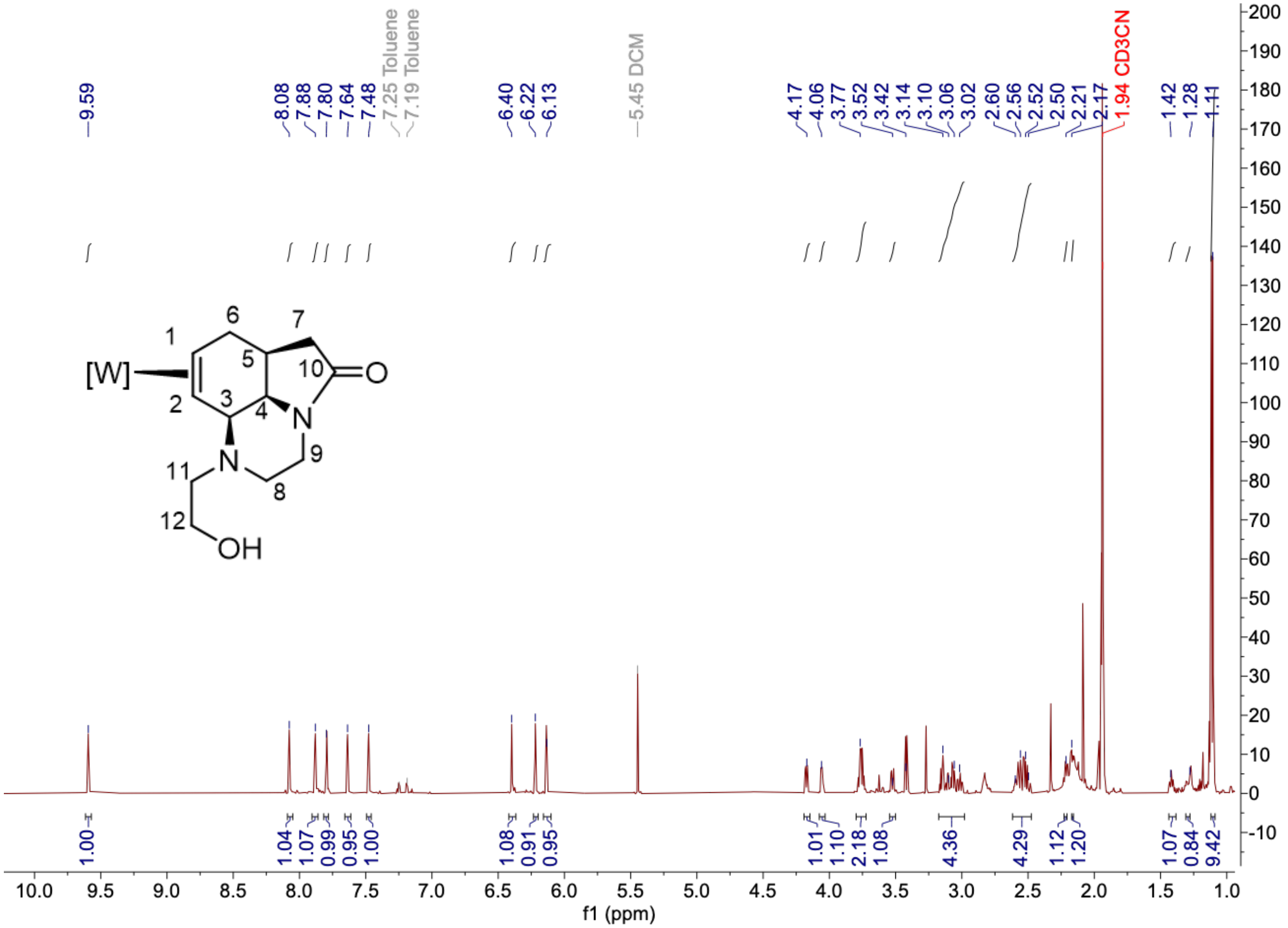

Supplementary Fig. 28: ^{1}H-NMR (CD3CN) of Compound **14**.

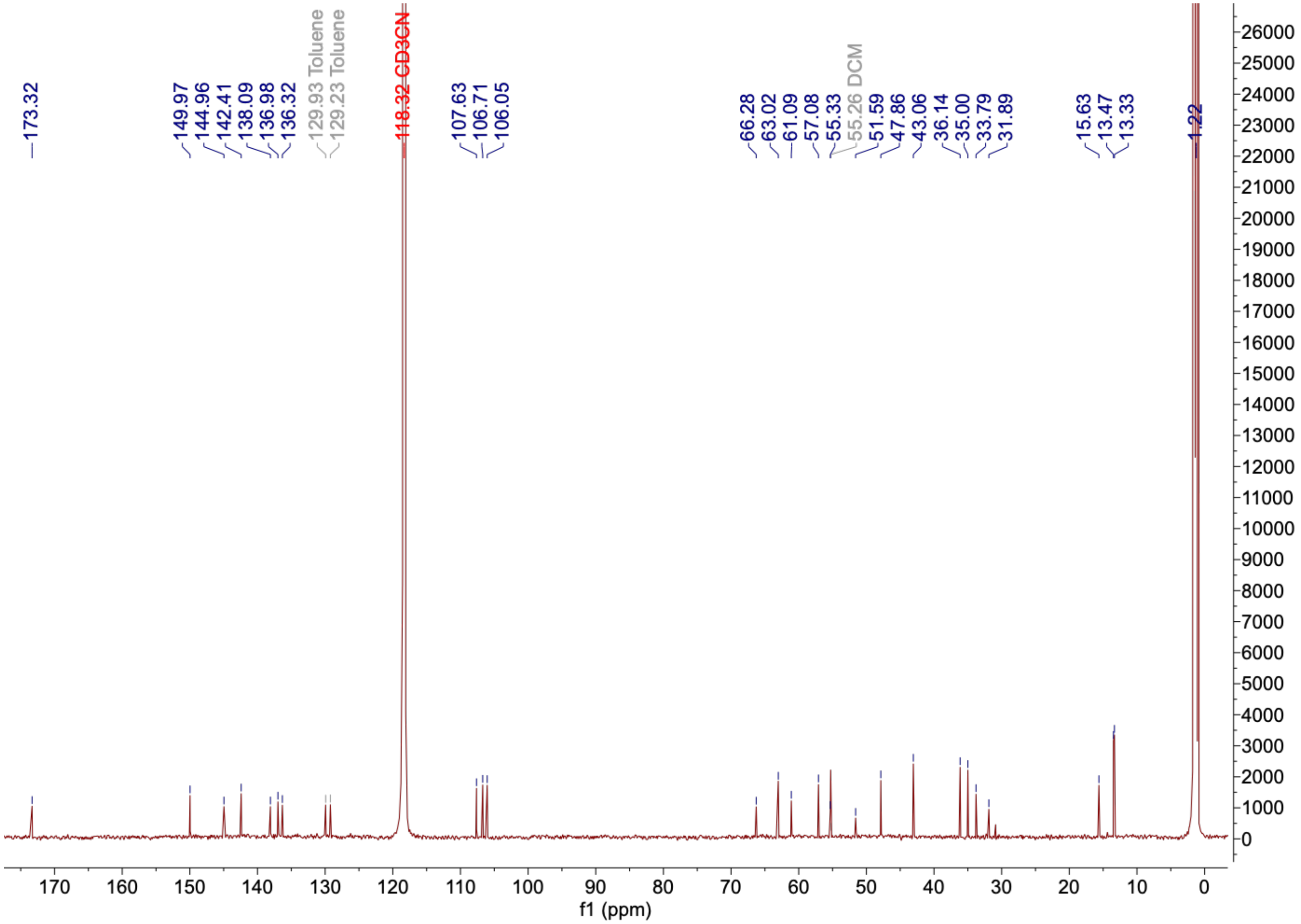

Supplementary Fig. 29: ^{13}C-NMR (CD3CN) of Compound **14**.

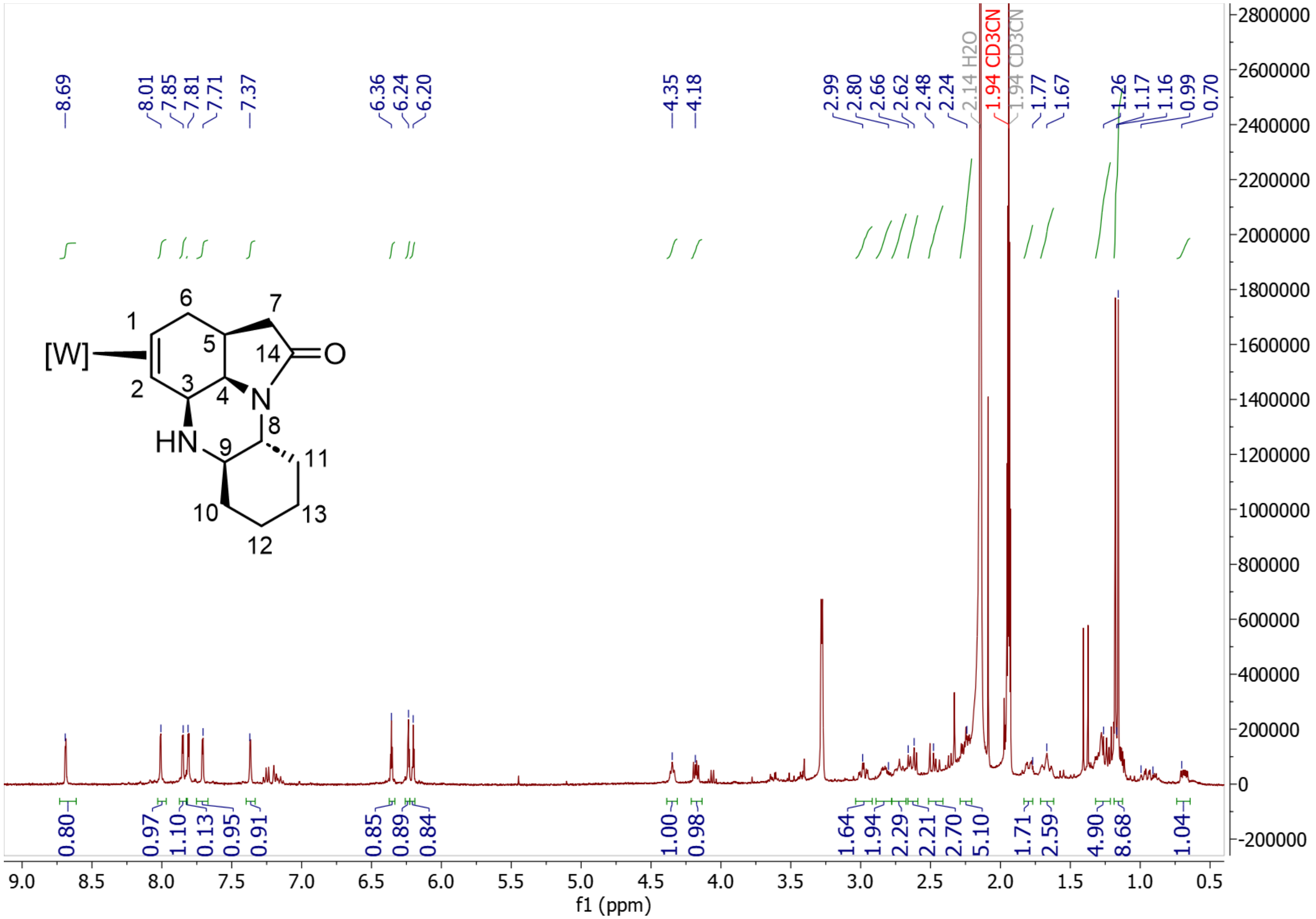

Supplementary Fig. 30: ^{1}H-NMR (CD3CN) of Compound **15**.

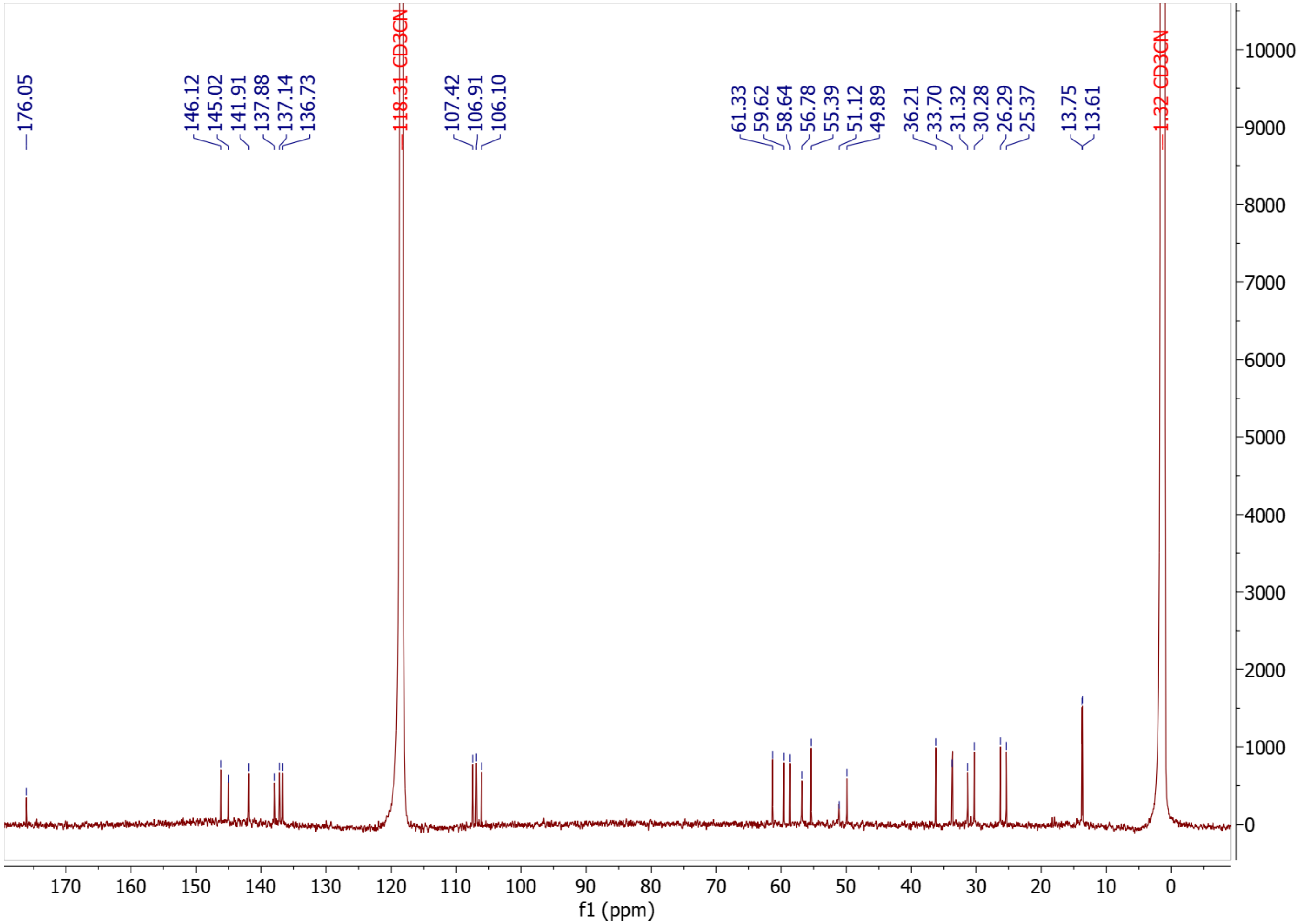

Supplementary Fig. 31: ^{13}C-NMR (CD3CN) of Compound **15**.

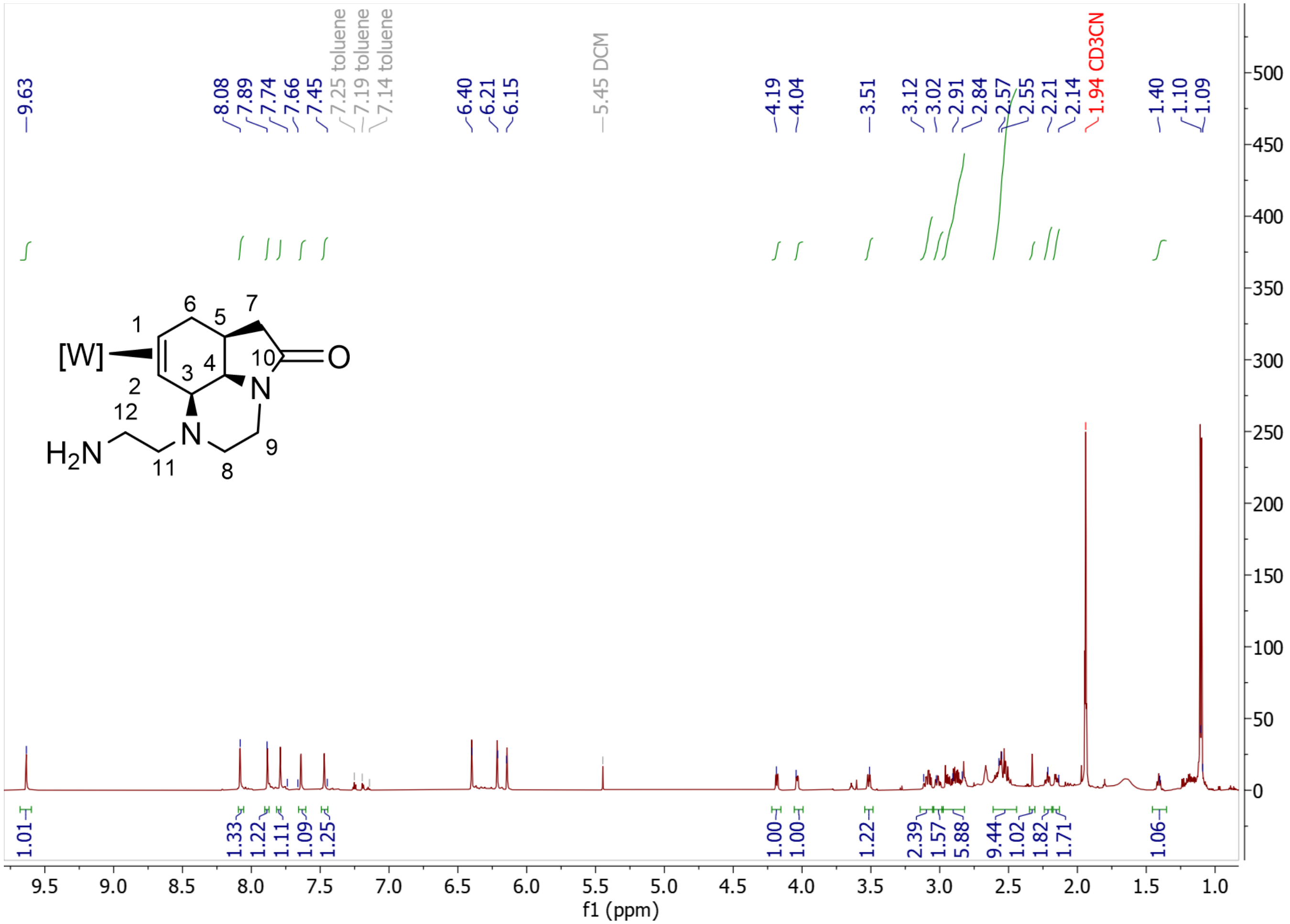

Supplementary Fig. 32: ^{1}H-NMR (CD3CN) of Compound **16**.

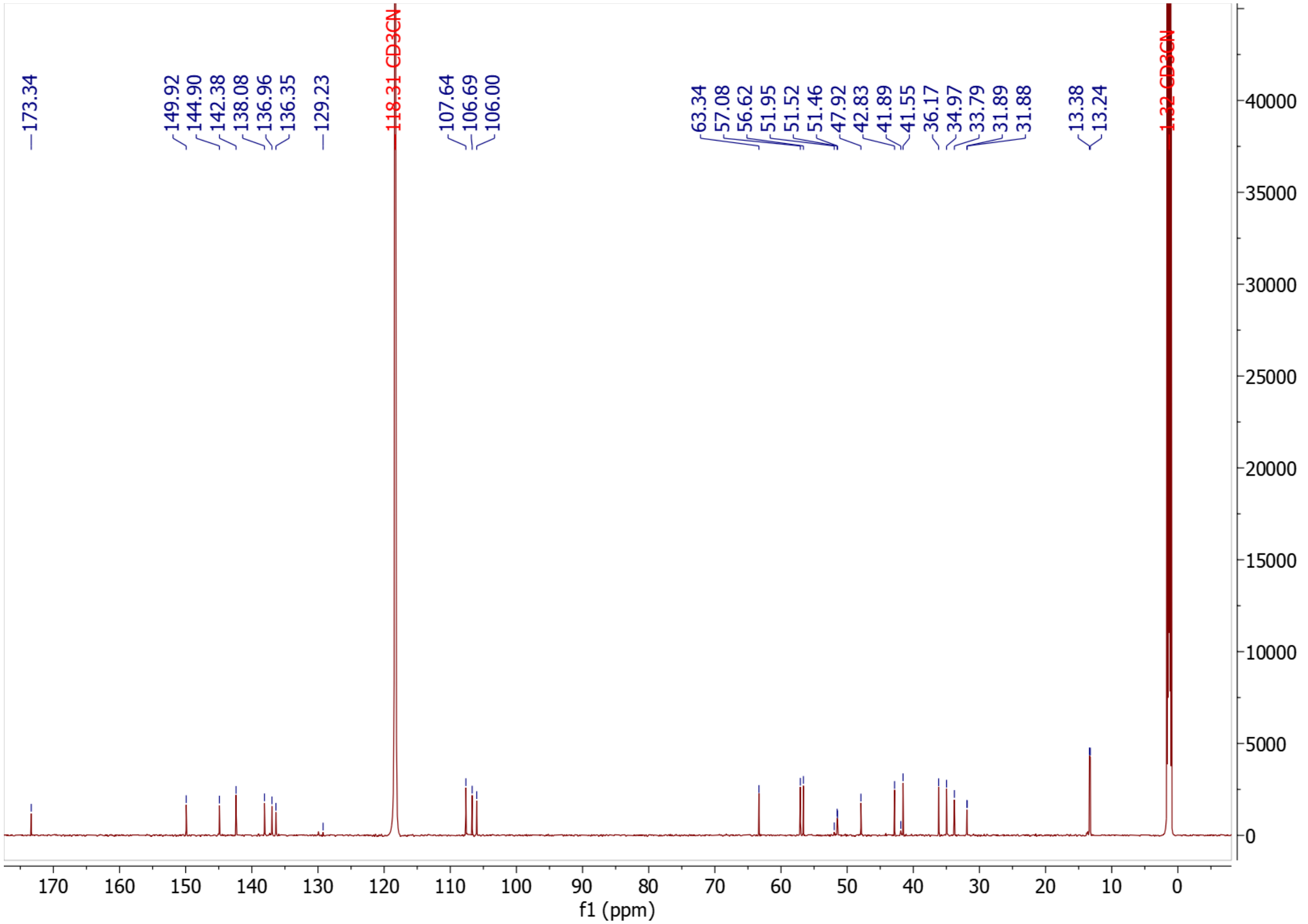

Supplementary Fig. 33: ^{13}C-NMR (CD3CN) of Compound **16**.

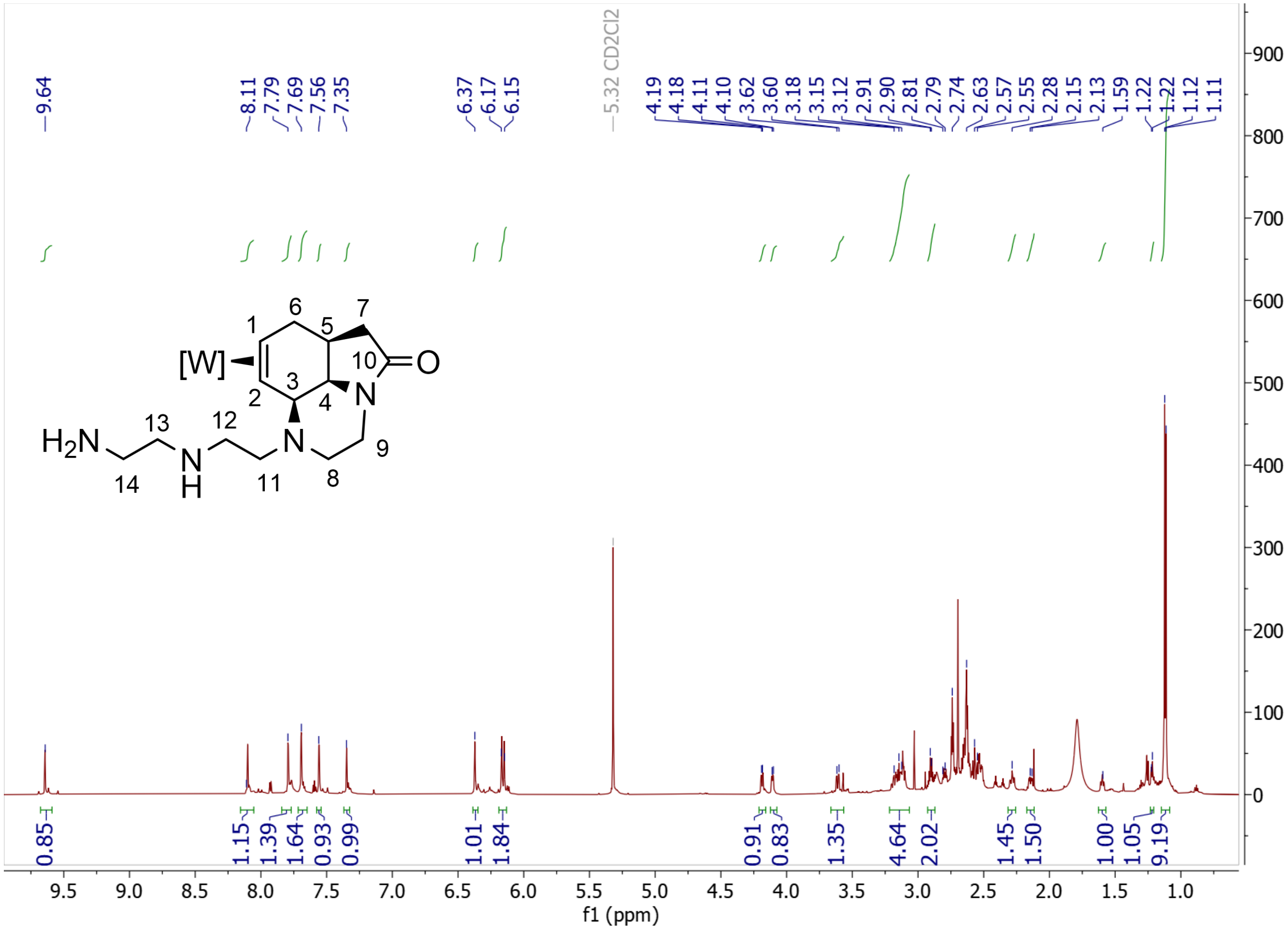

Supplementary Fig. 34: ^{1}H-NMR (CD_2Cl_2) of Compound **17**.

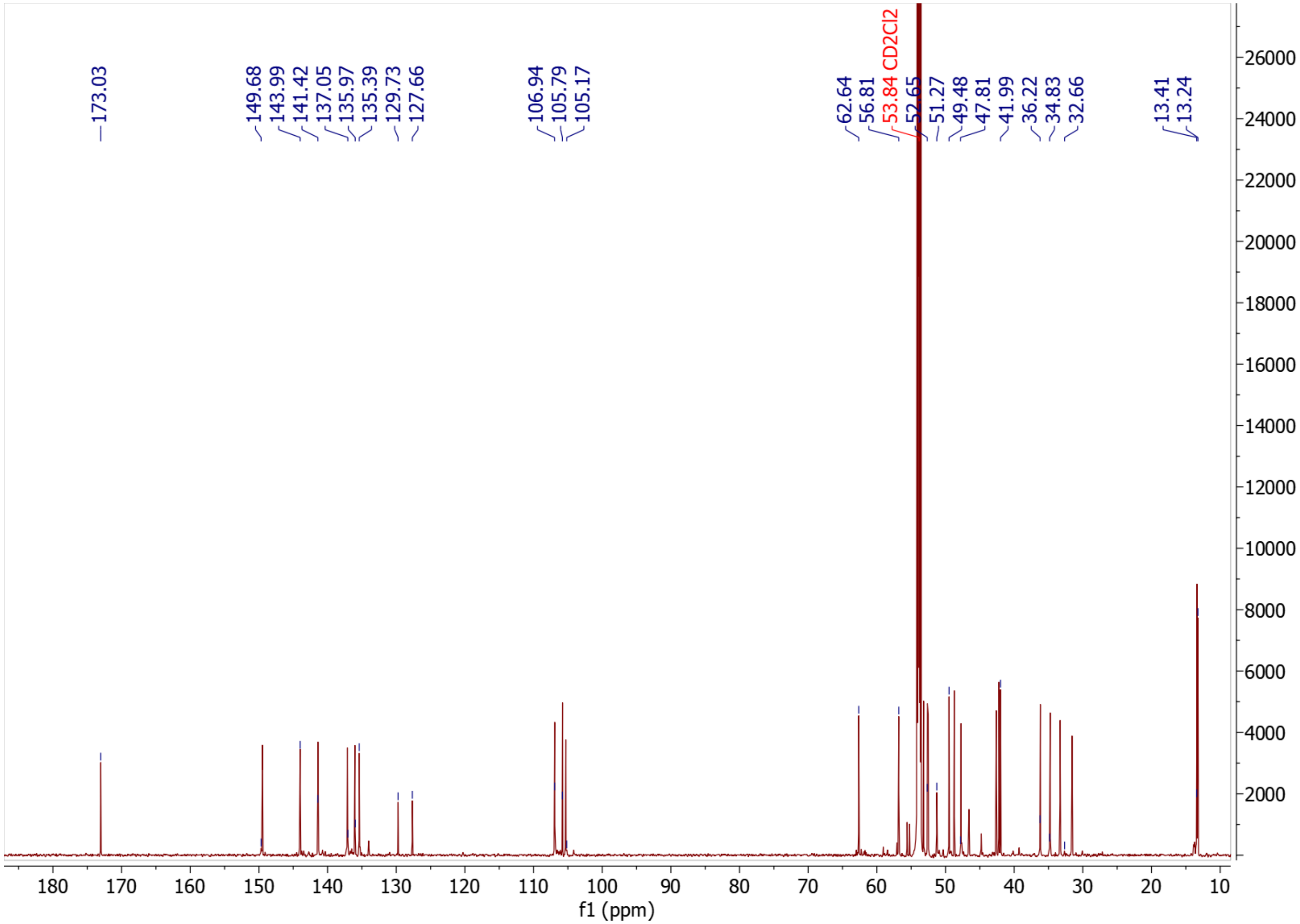

Supplementary Fig. 35: ^{13}C-NMR (CD3CN) of Compound **17**.

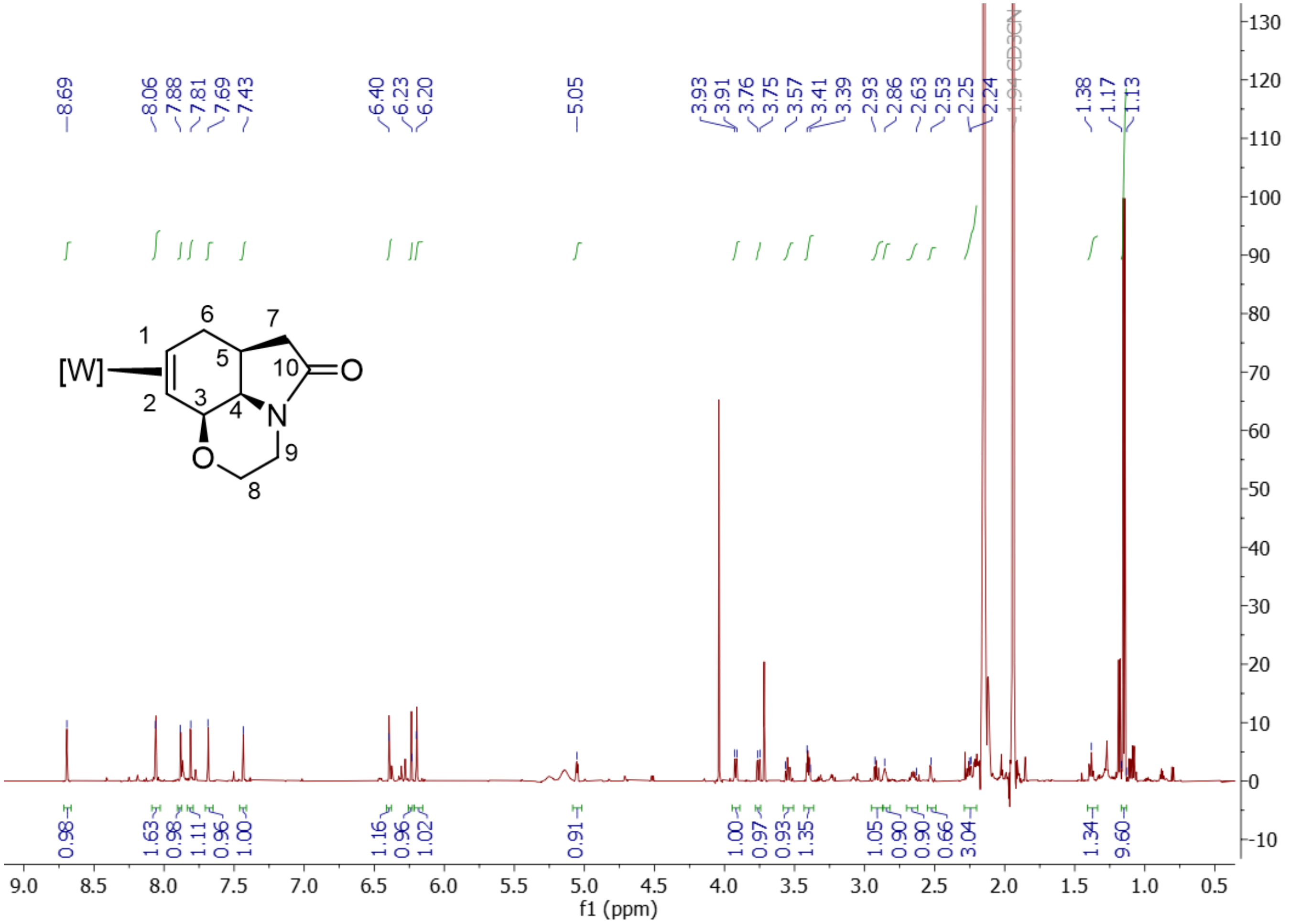

Supplementary Fig. 36: ^{1}H-NMR (CD_3CN) of Compound **18**.

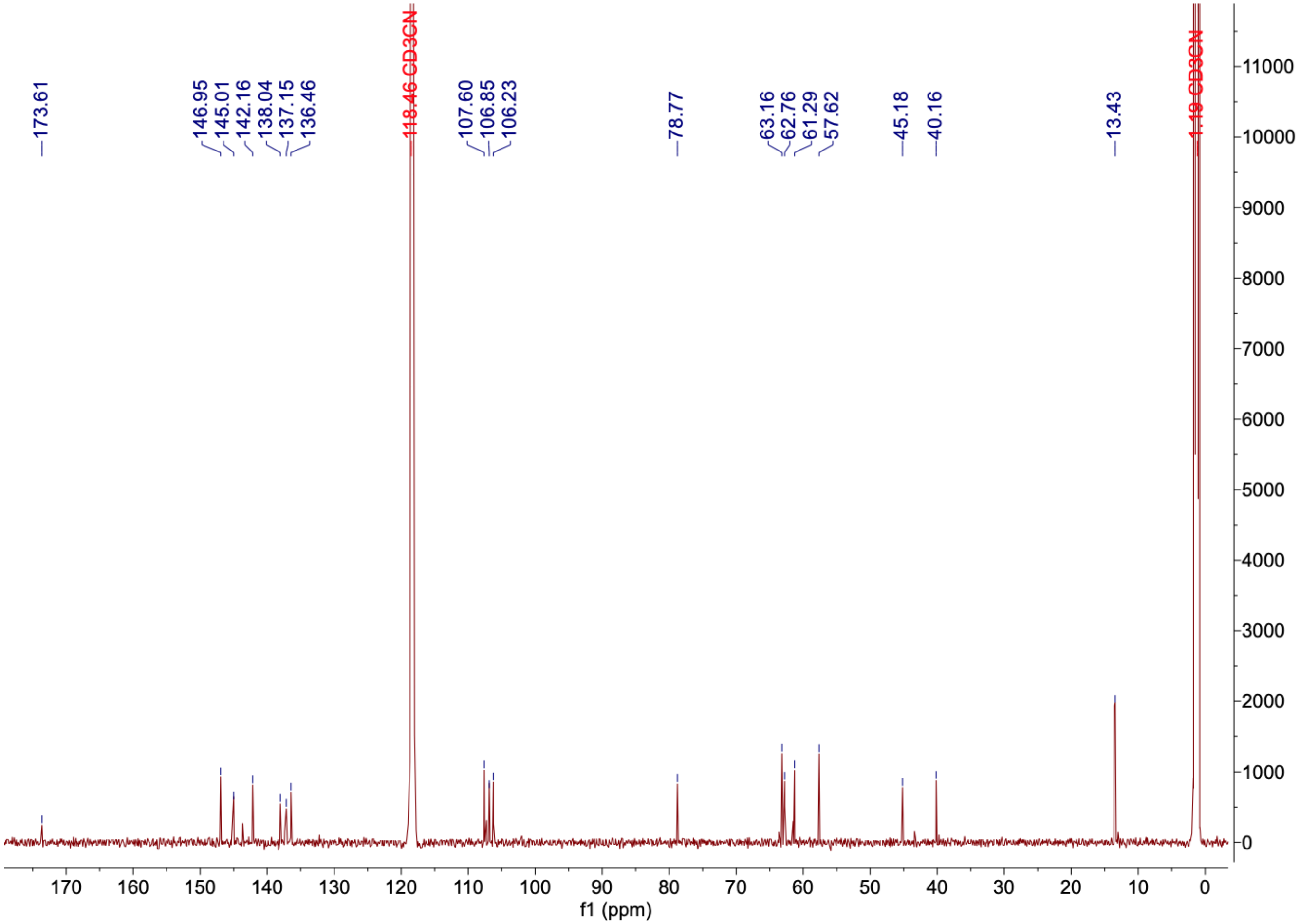

Supplementary Fig. 37: ^{13}C-NMR (CD_3CN) of Compound **18**.

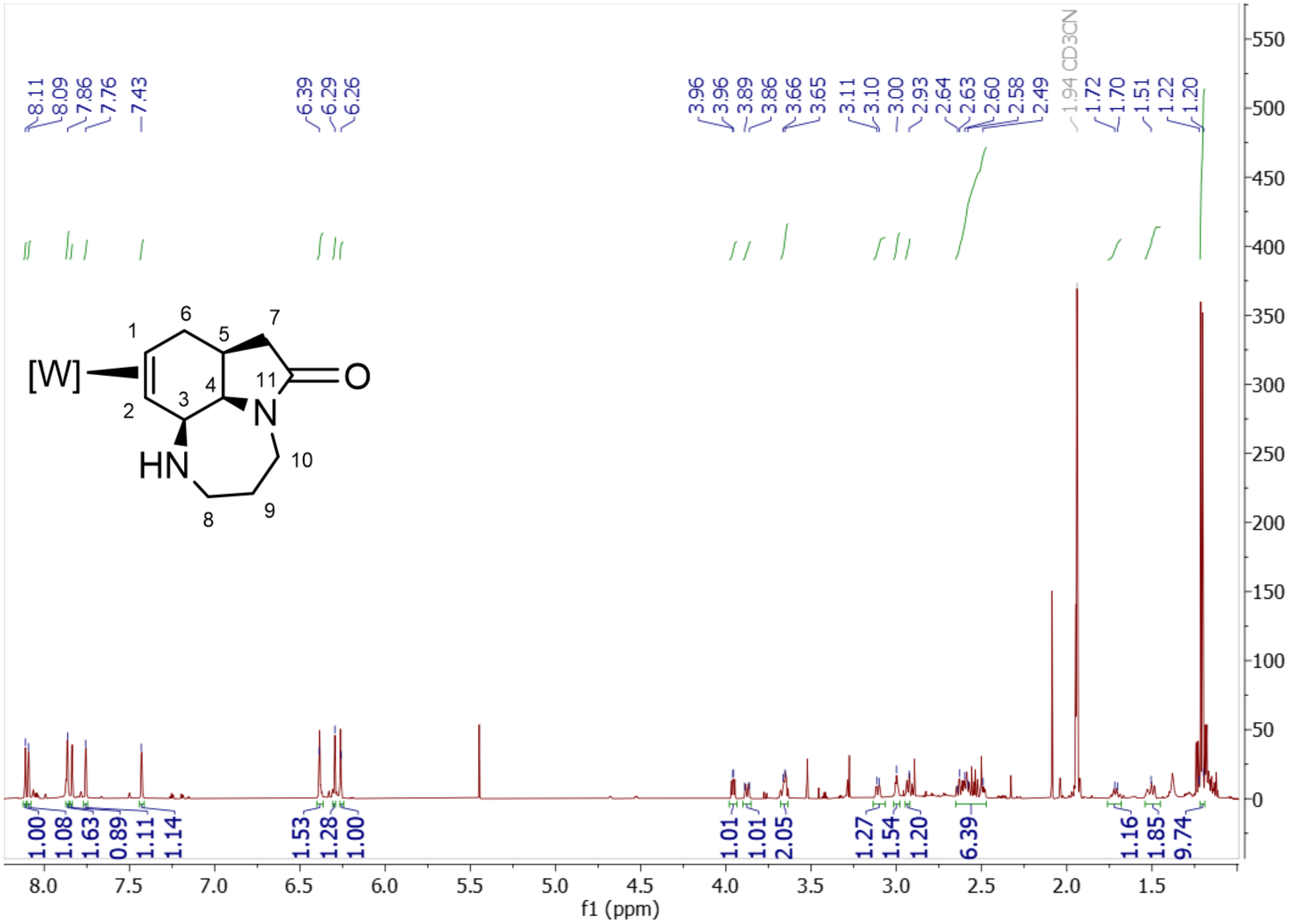

Supplementary Fig. 38: ^{1}H-NMR (CD_3CN) of Compound **19**.

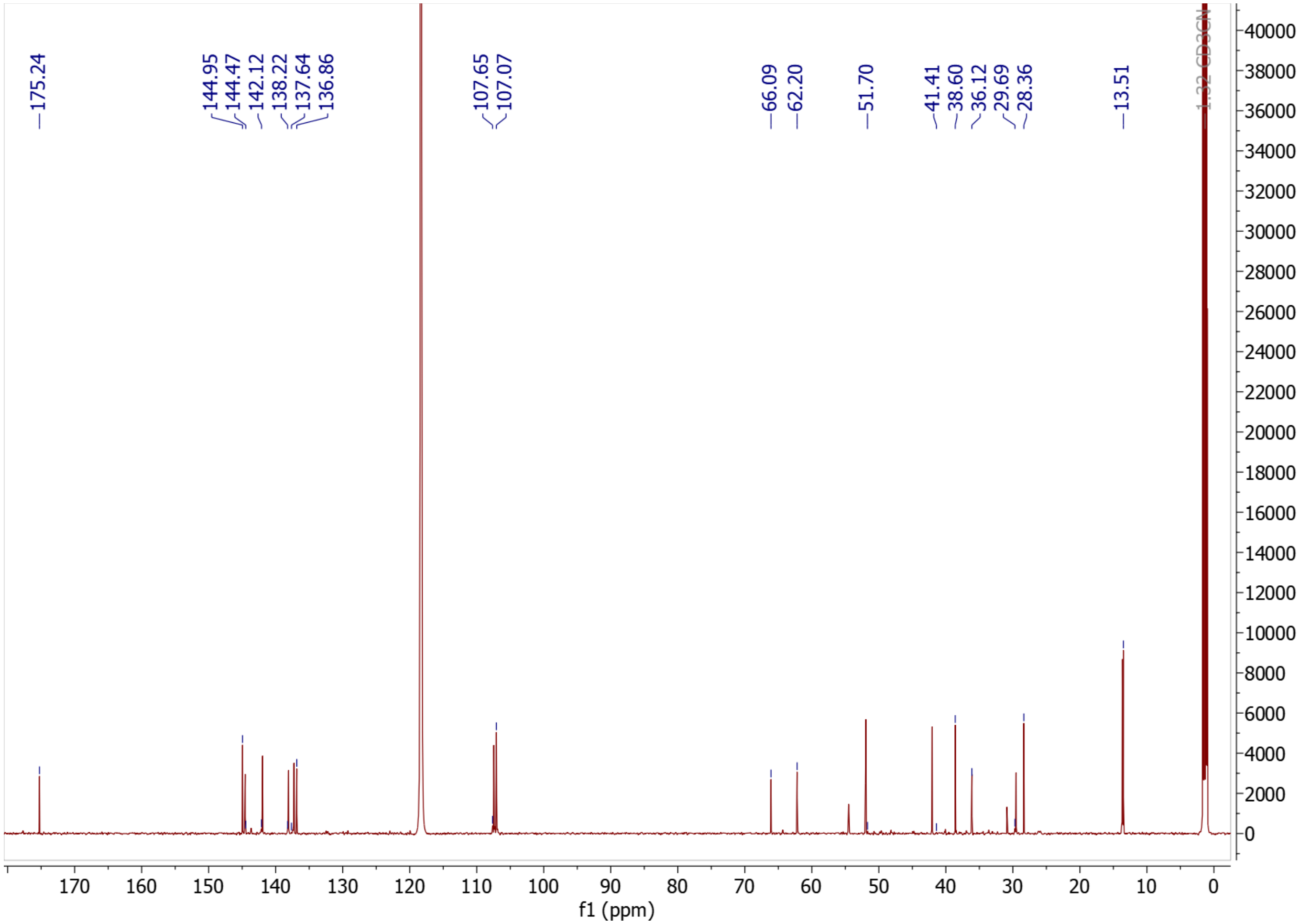

Supplementary Fig. 39: ^{13}C-NMR (CD_3CN) of Compound **19**.

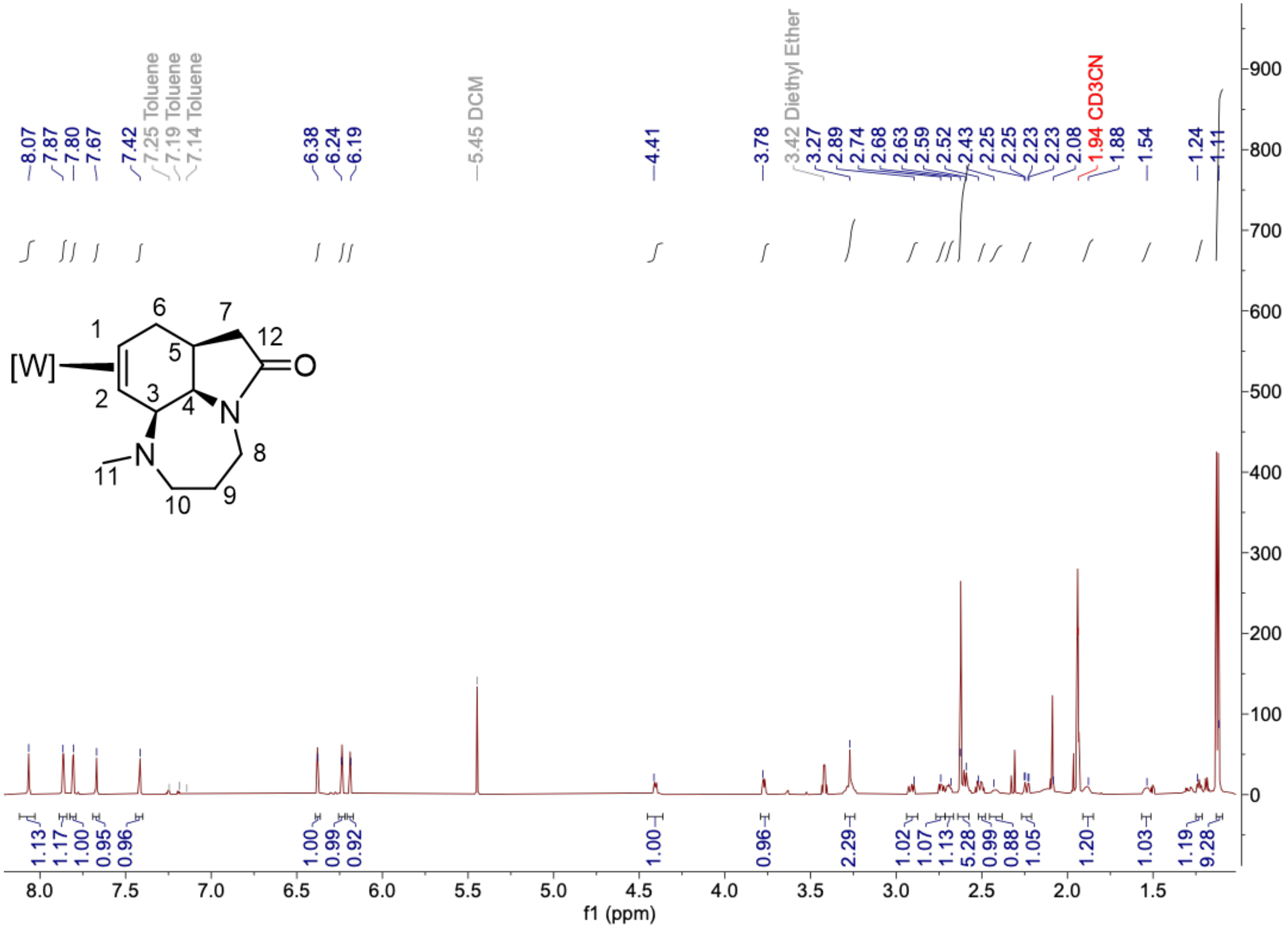

Supplementary Fig. 40: ^{1}H-NMR (CD_3CN) of Compound **20**.

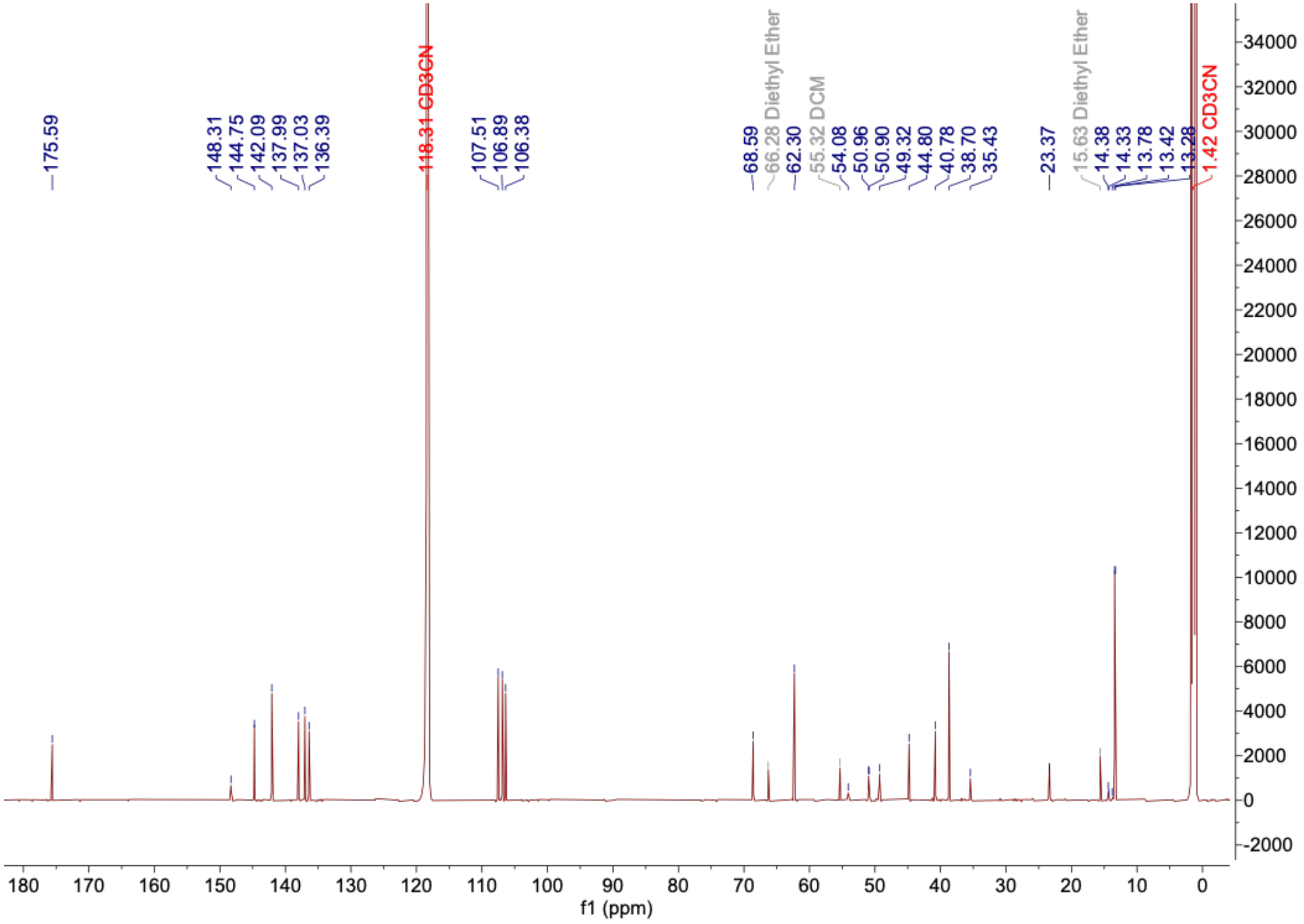

Supplementary Fig. 41: ^{13}C-NMR (CD_3CN) of Compound **20**.

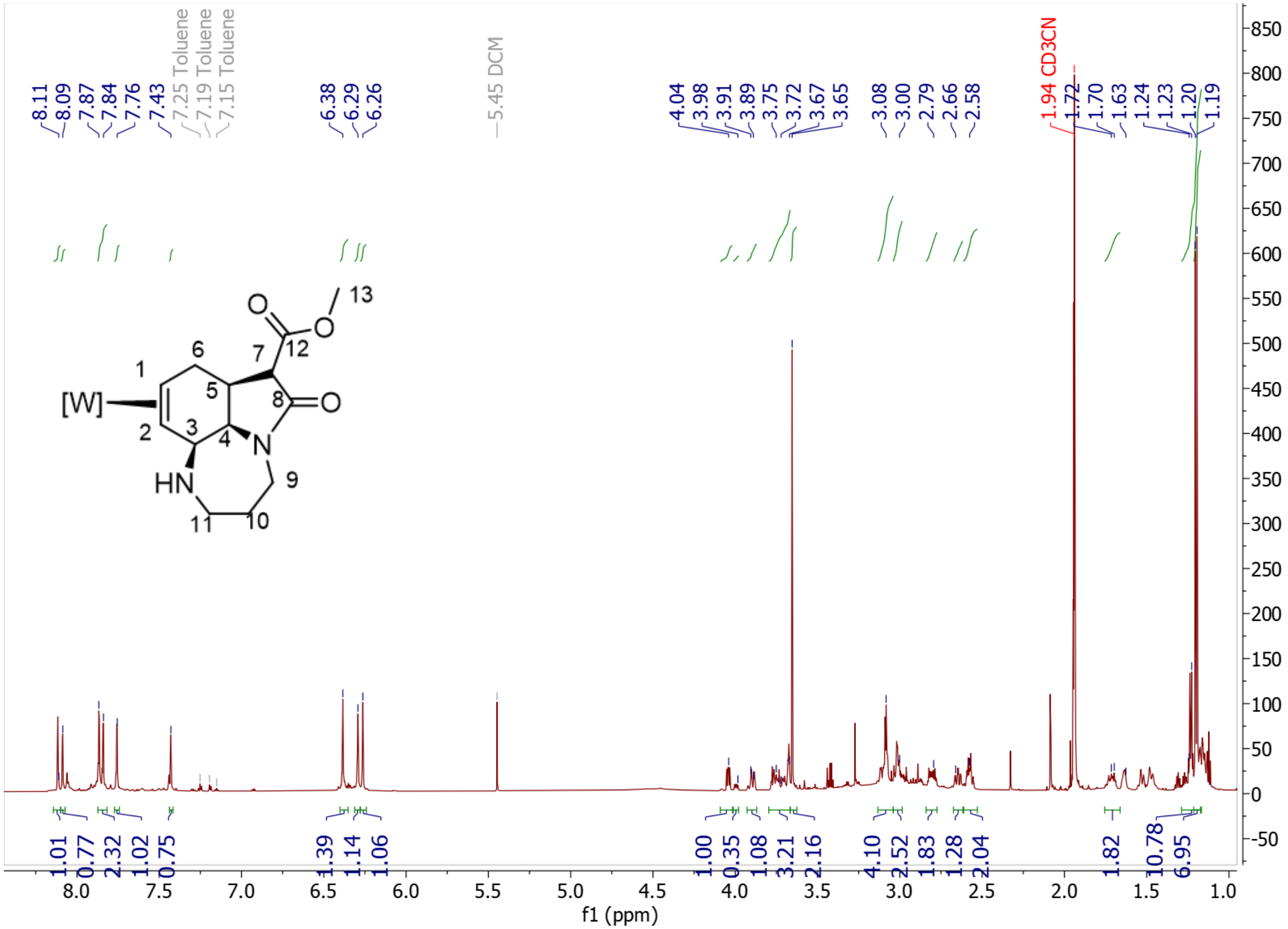

Supplementary Fig. 42: ^{1}H-NMR (CD_3CN) of Compound **21**.
2 epimers are observed in the NMR spectra.

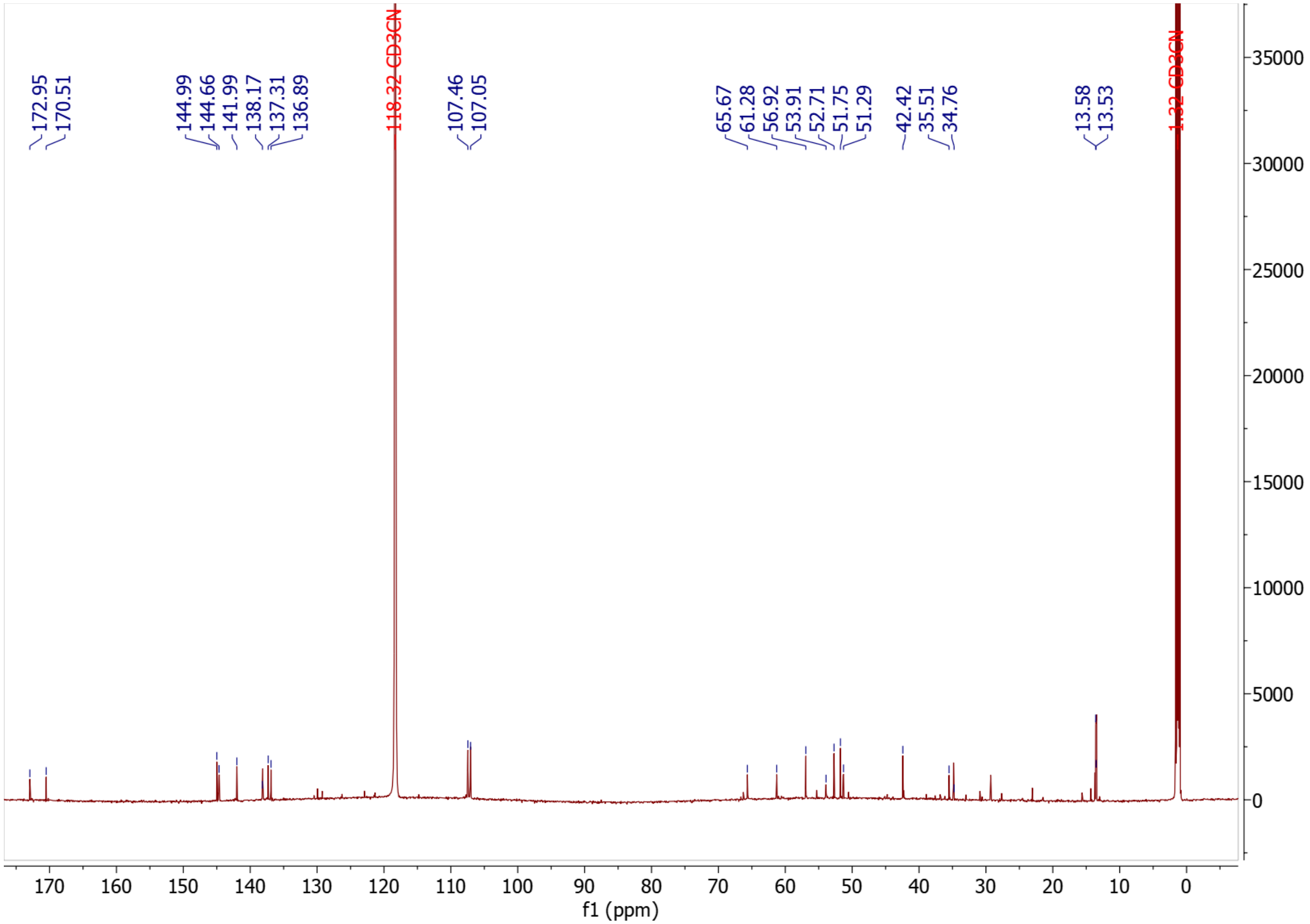

Supplementary Fig. 43: ^{13}C-NMR (CD_3CN) of Compound **21**.
2 epimers are observed in the NMR spectra.

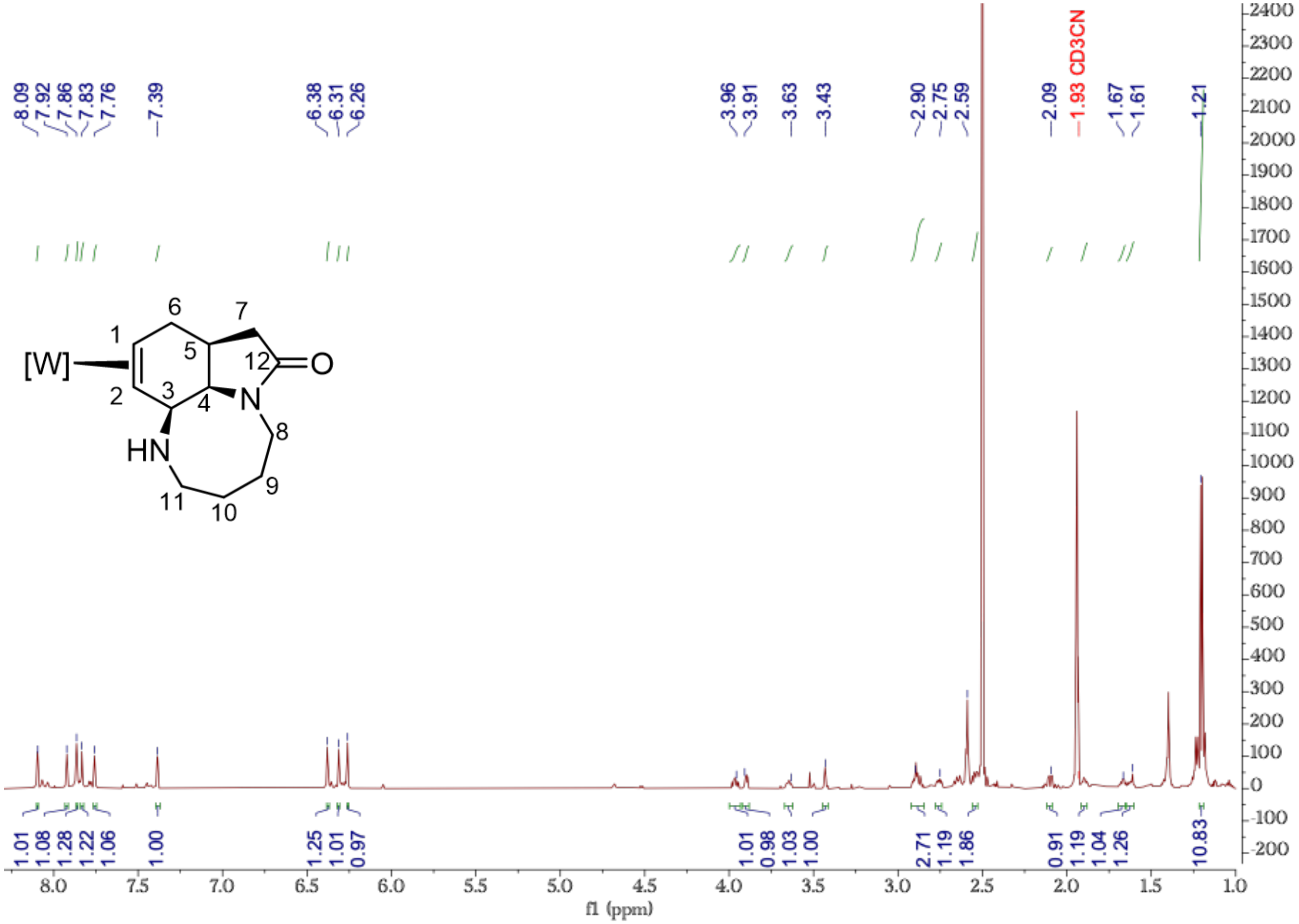

Supplementary Fig. 44: ^{1}H-NMR (CD_3CN) of Compound **22**.

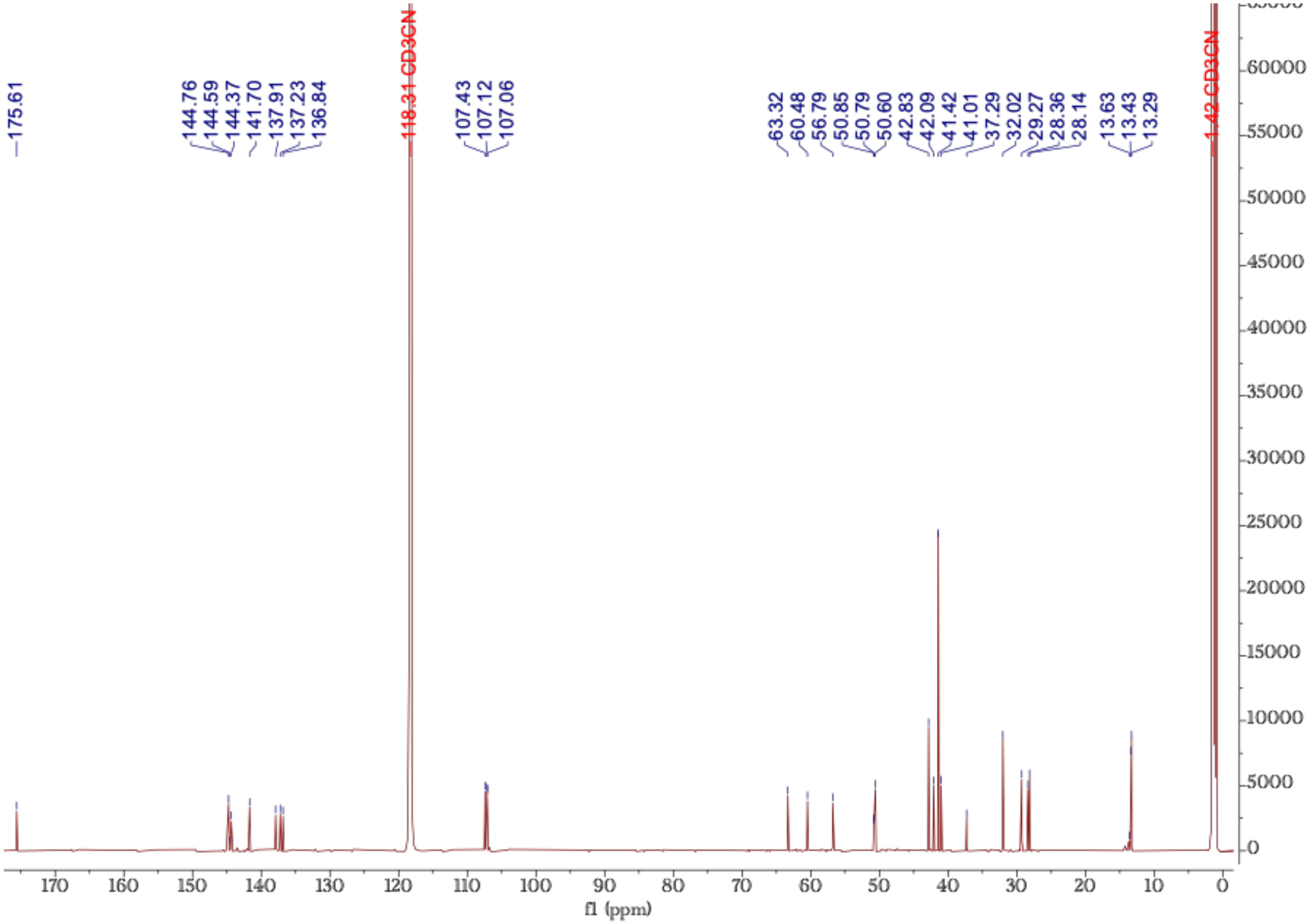

Supplementary Fig. 45: ^{13}C-NMR (CD_3CN) of Compound **22**.

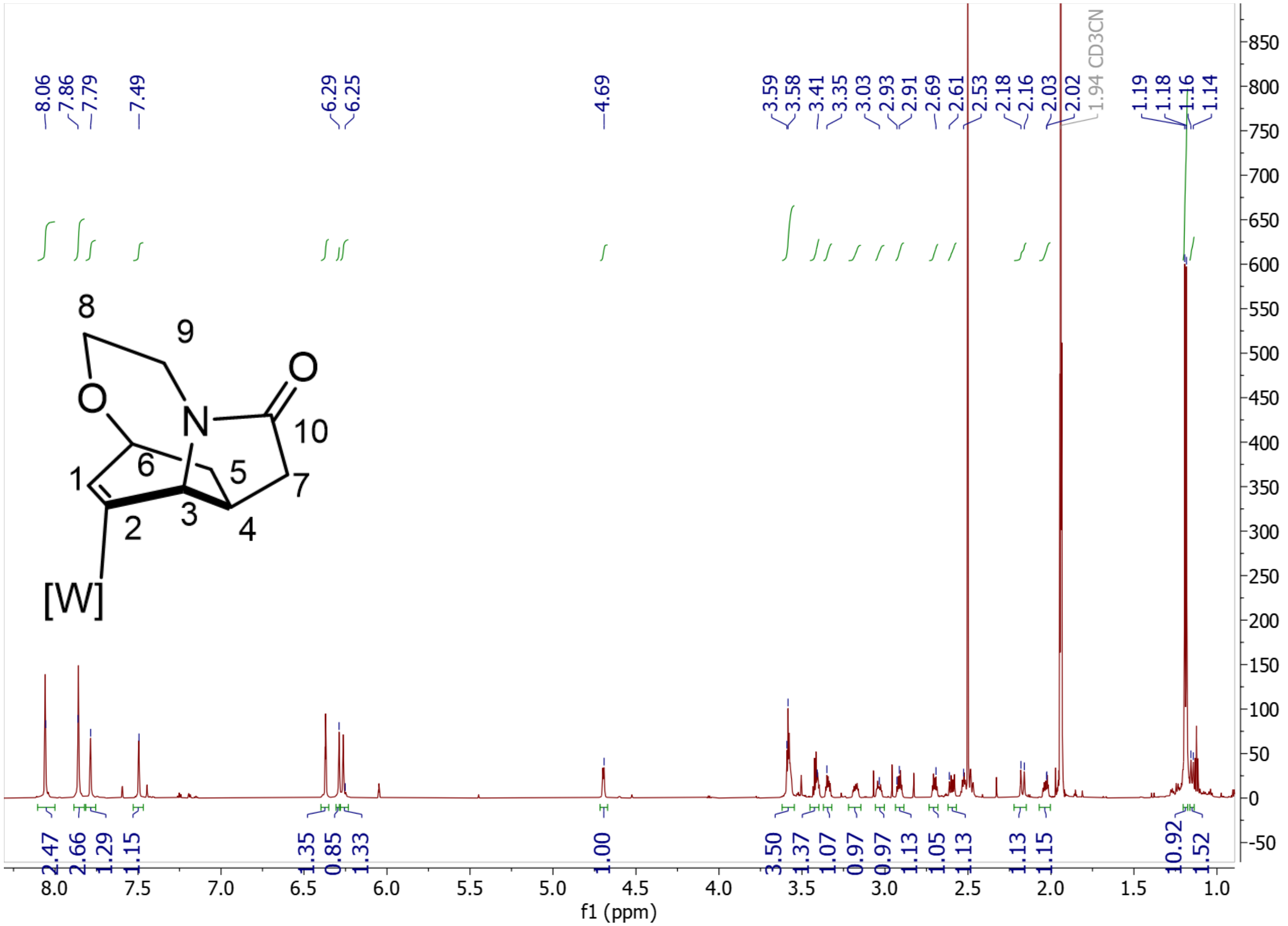

Supplementary Fig. 46: ^{1}H-NMR (CD_3CN) of Compound **23**.

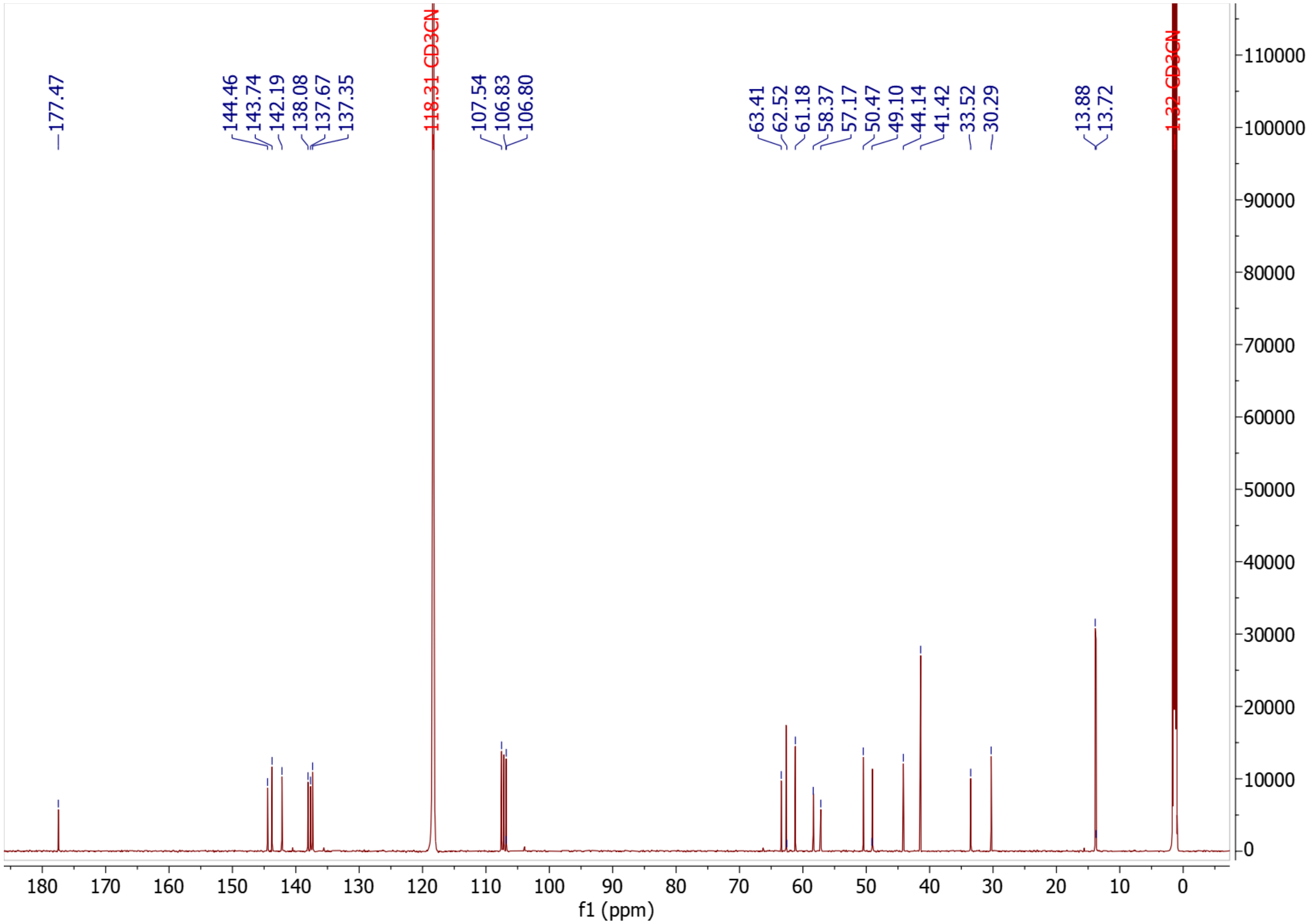

Supplementary Fig. 47: ^{13}C-NMR (CD_3CN) of Compound **23**.

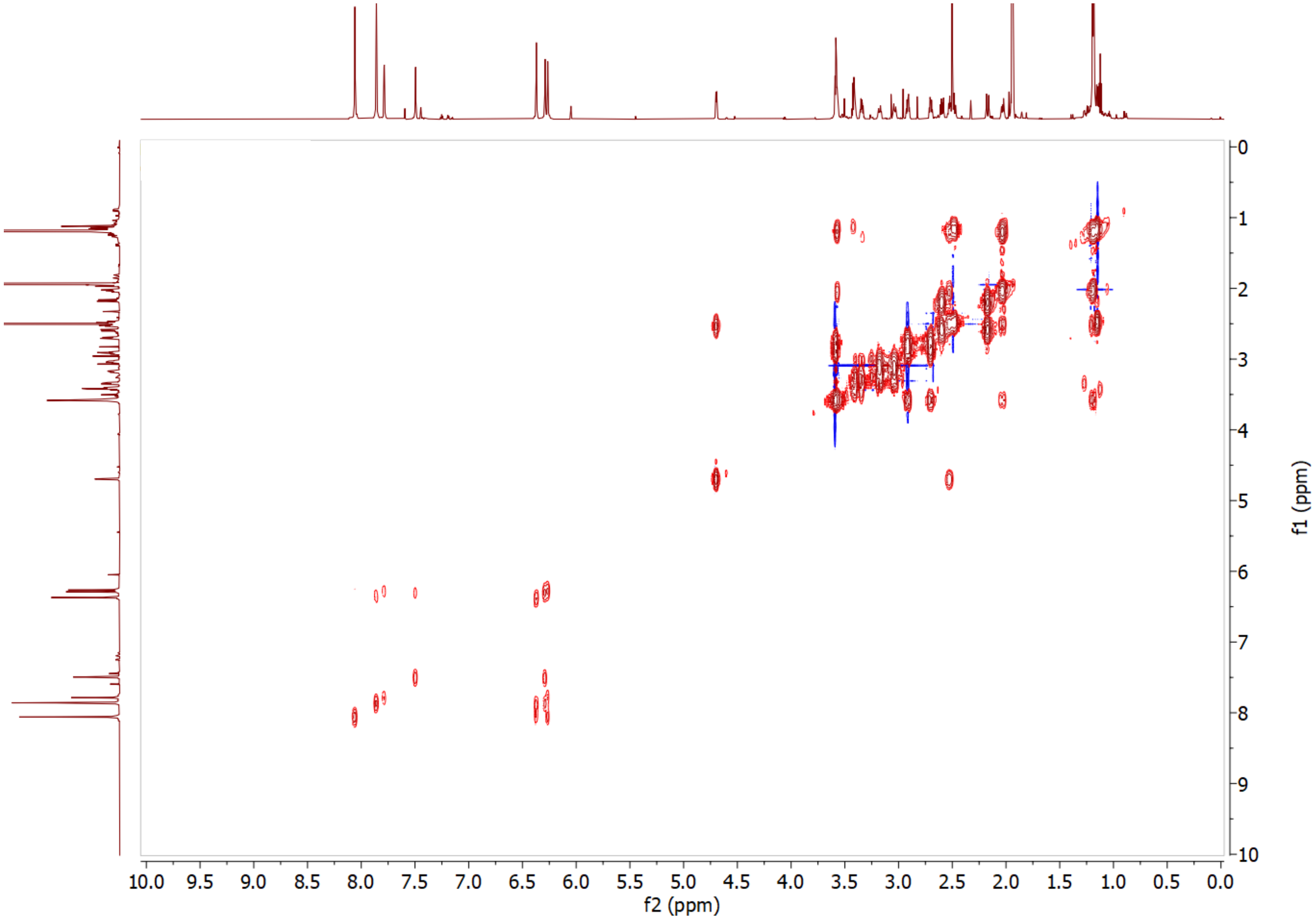

Supplementary Fig. 48: ^{1}H-^{1}H COSY (CD_3CN) of Compound **23**.

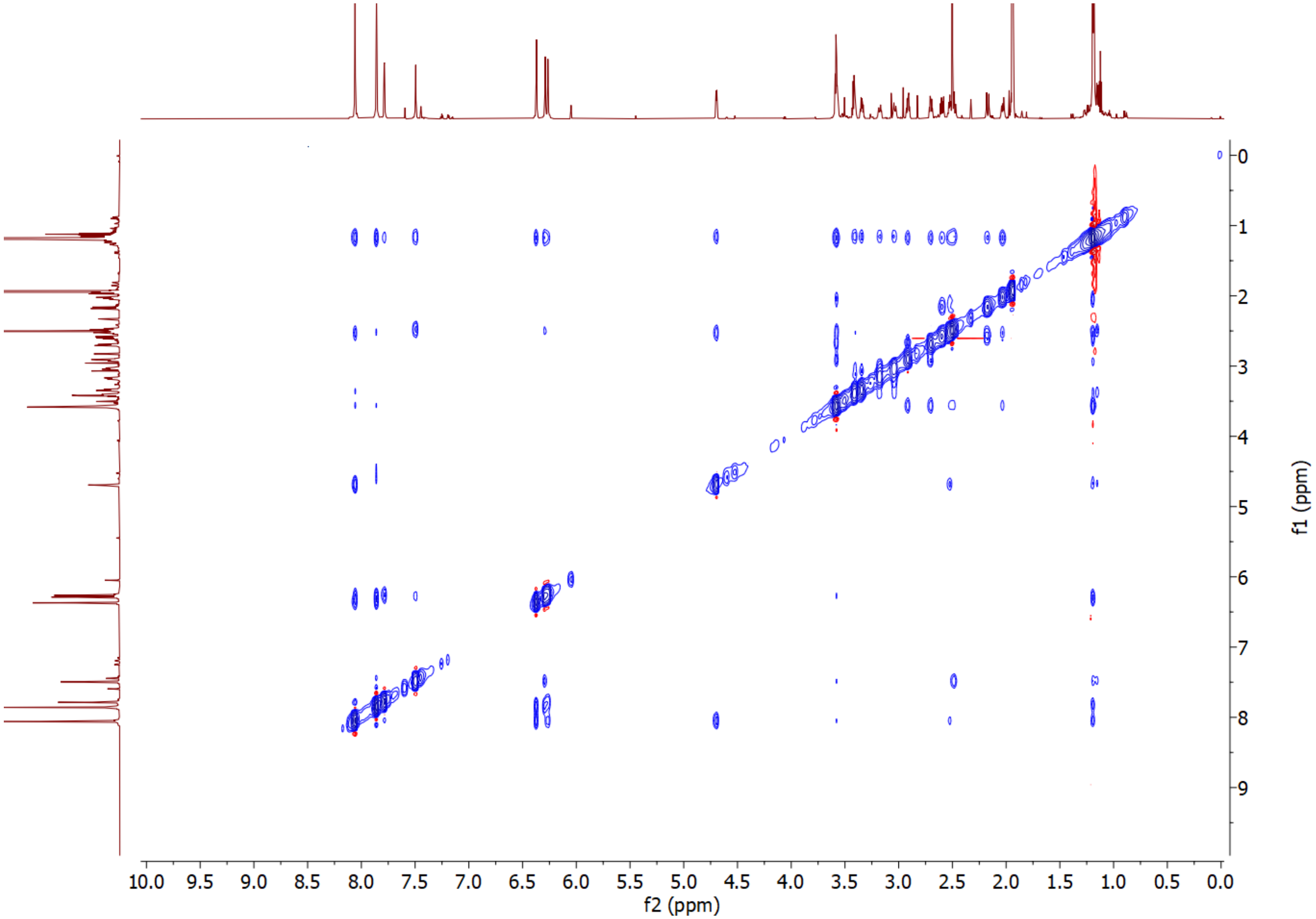

Supplementary Fig. 49: ^{1}H-^{1}H NOESY (CD_3CN) of Compound **23**.

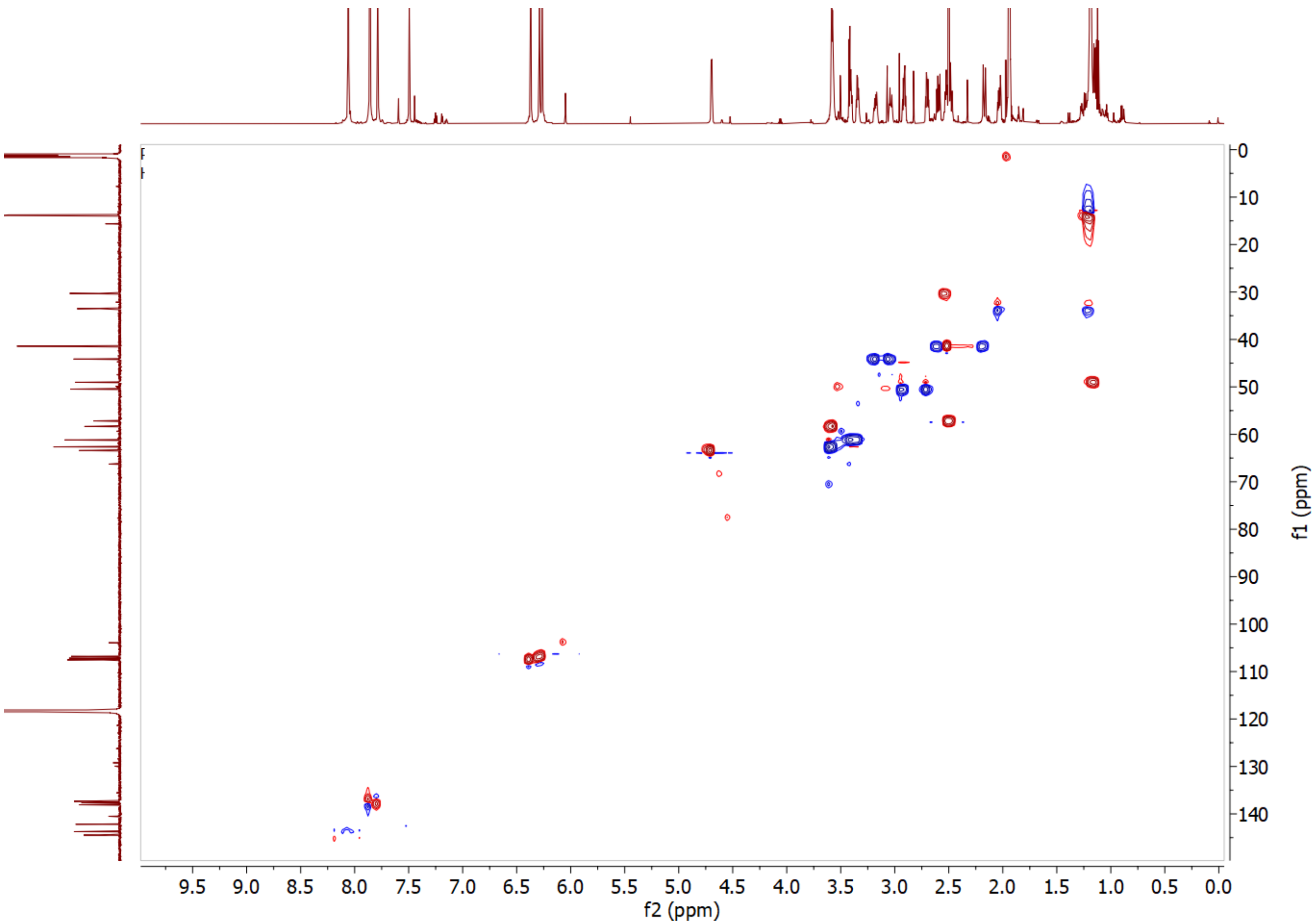

Supplementary Fig. 50: ^{1}H-^{13}C HSQC (CD_3CN) of Compound **23**.

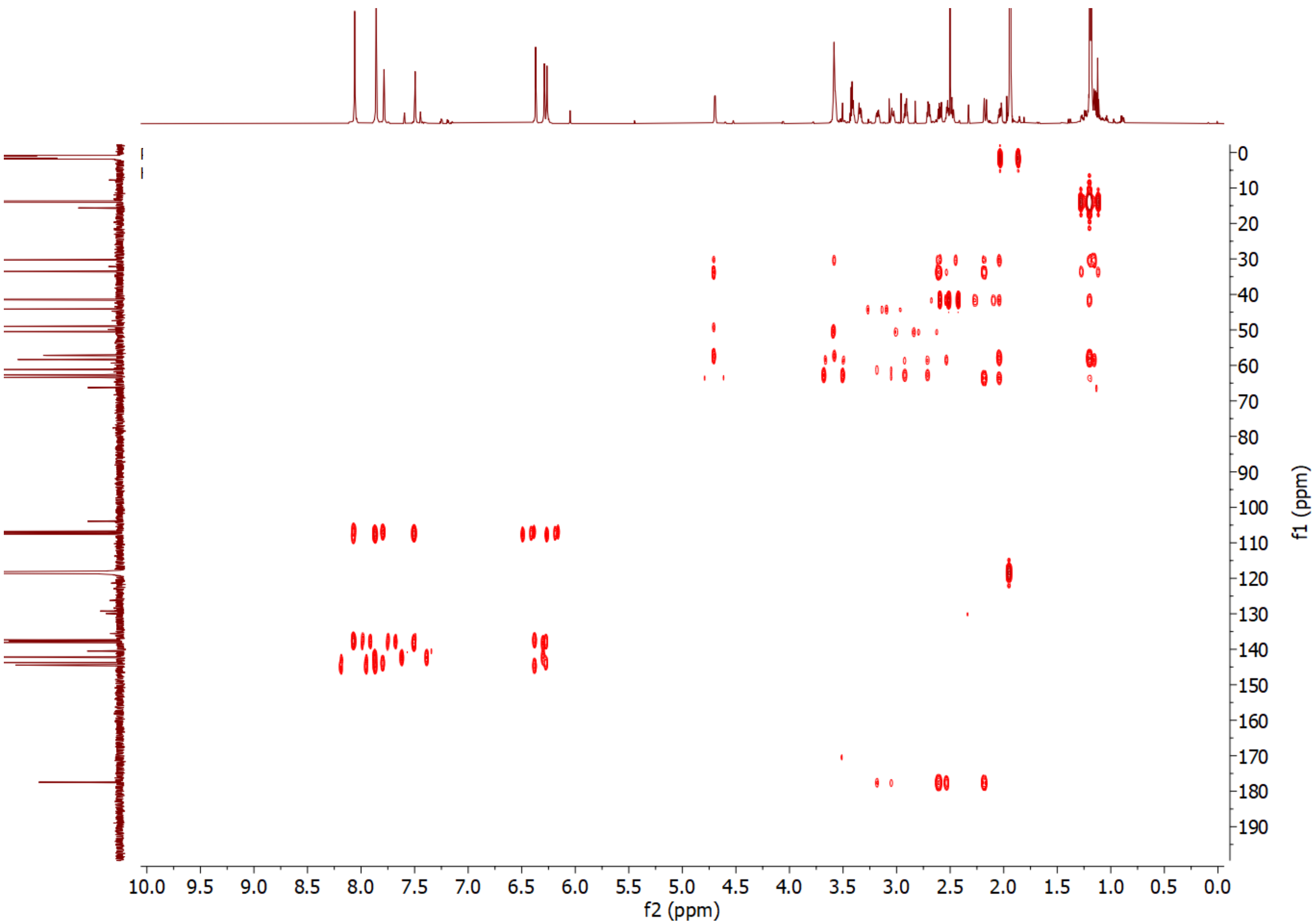

Supplementary Fig. 51: ^{1}H-^{13}C HMBC (CD_3CN) of Compound **23**.

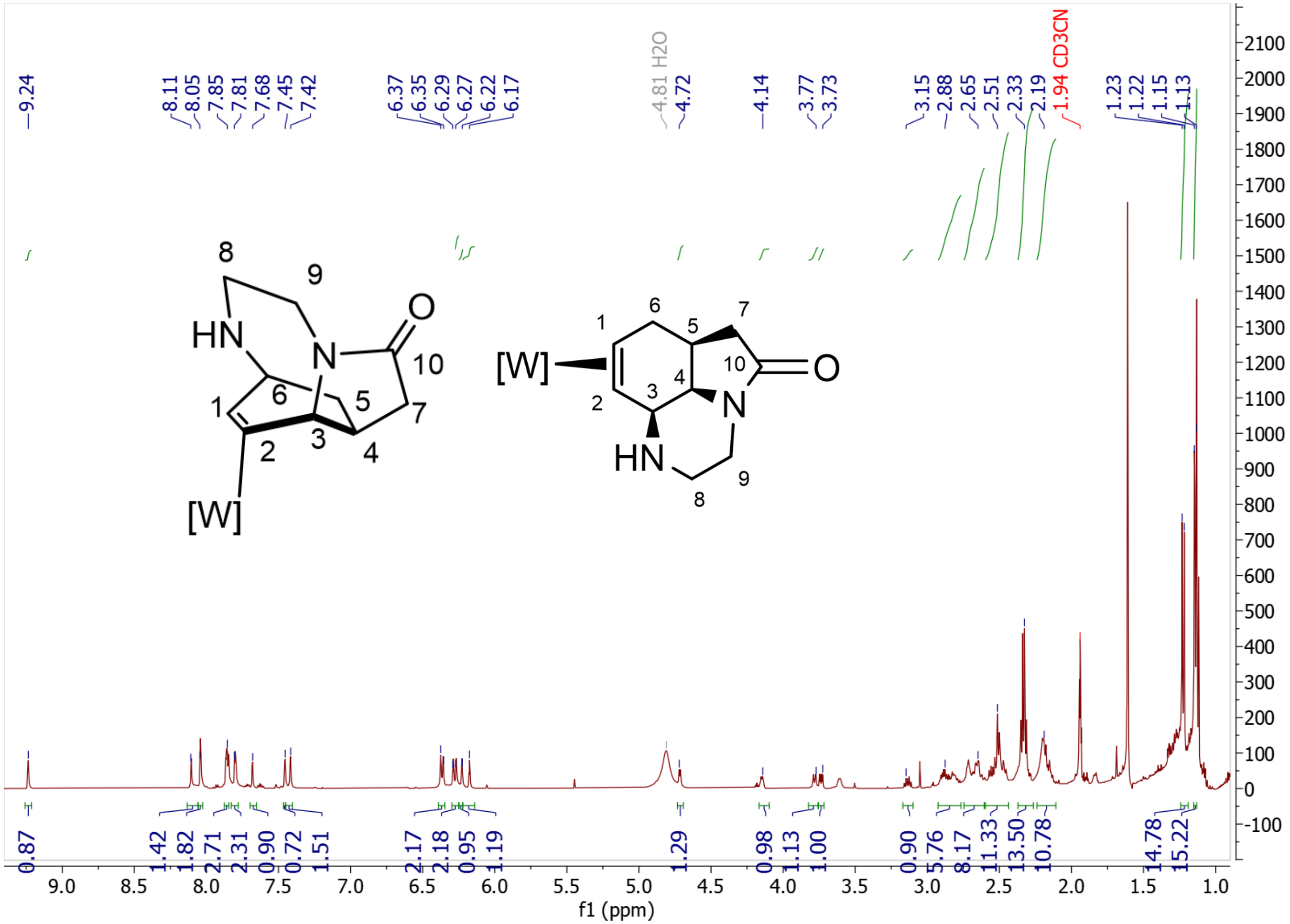

Supplementary Fig. 52: ^{1}H-NMR (CD_3CN) of Compound **24** and **5**.

Different reaction and separation conditions were employed to purify 24, but the mixture never resolved to a single isomer. Multiple crystallization experiments were attempted. However, none proved successful in isolating 24.

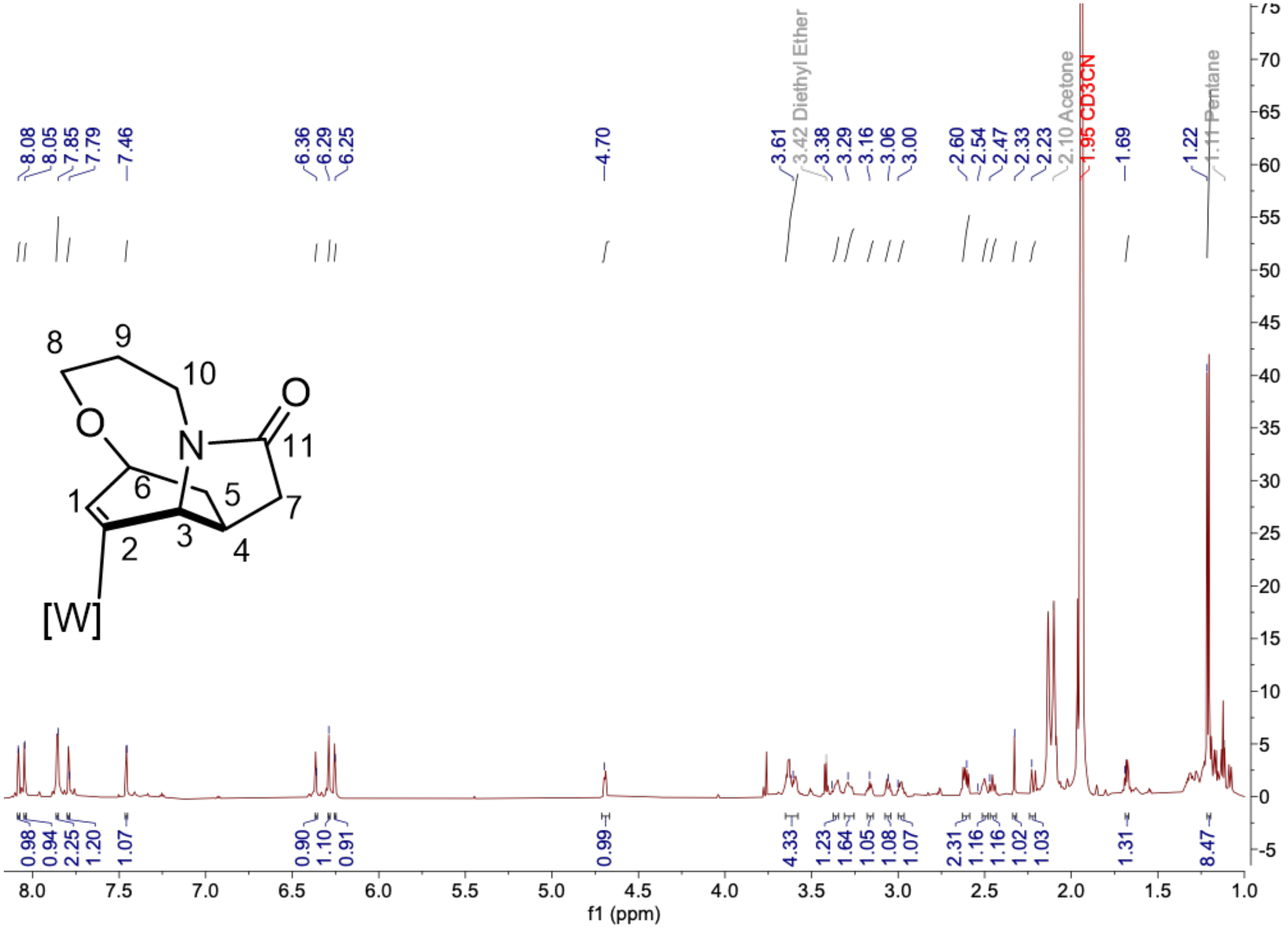

Supplementary Fig. 53: ^{1}H-NMR (CD_3CN) of Compound **25**.

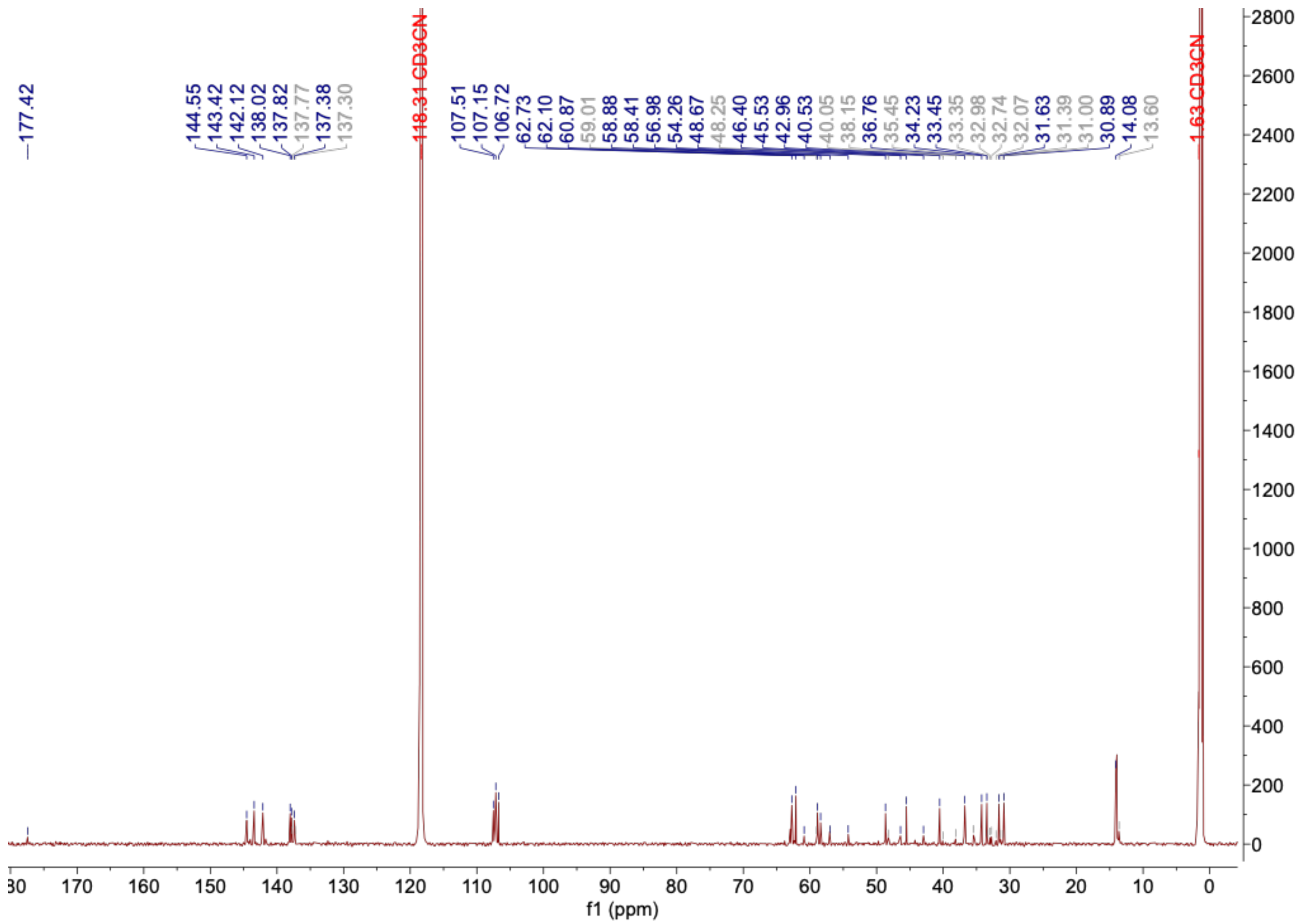

Supplementary Fig. 54: ^{13}C-NMR (CD_3CN) of Compound **25**.

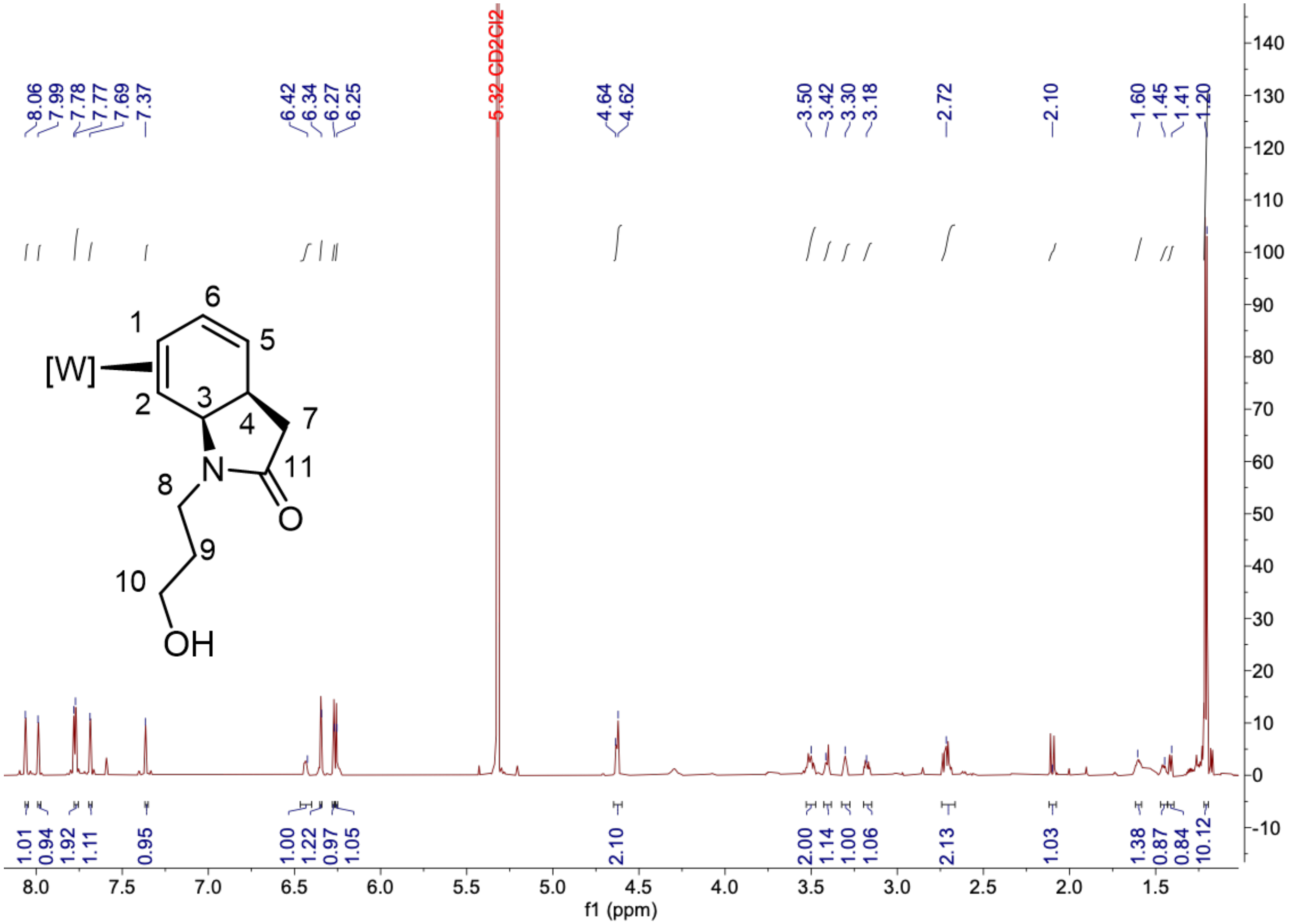

Supplementary Fig. 55: ^{1}H-NMR (CD_2Cl_2) of Compound **26**.

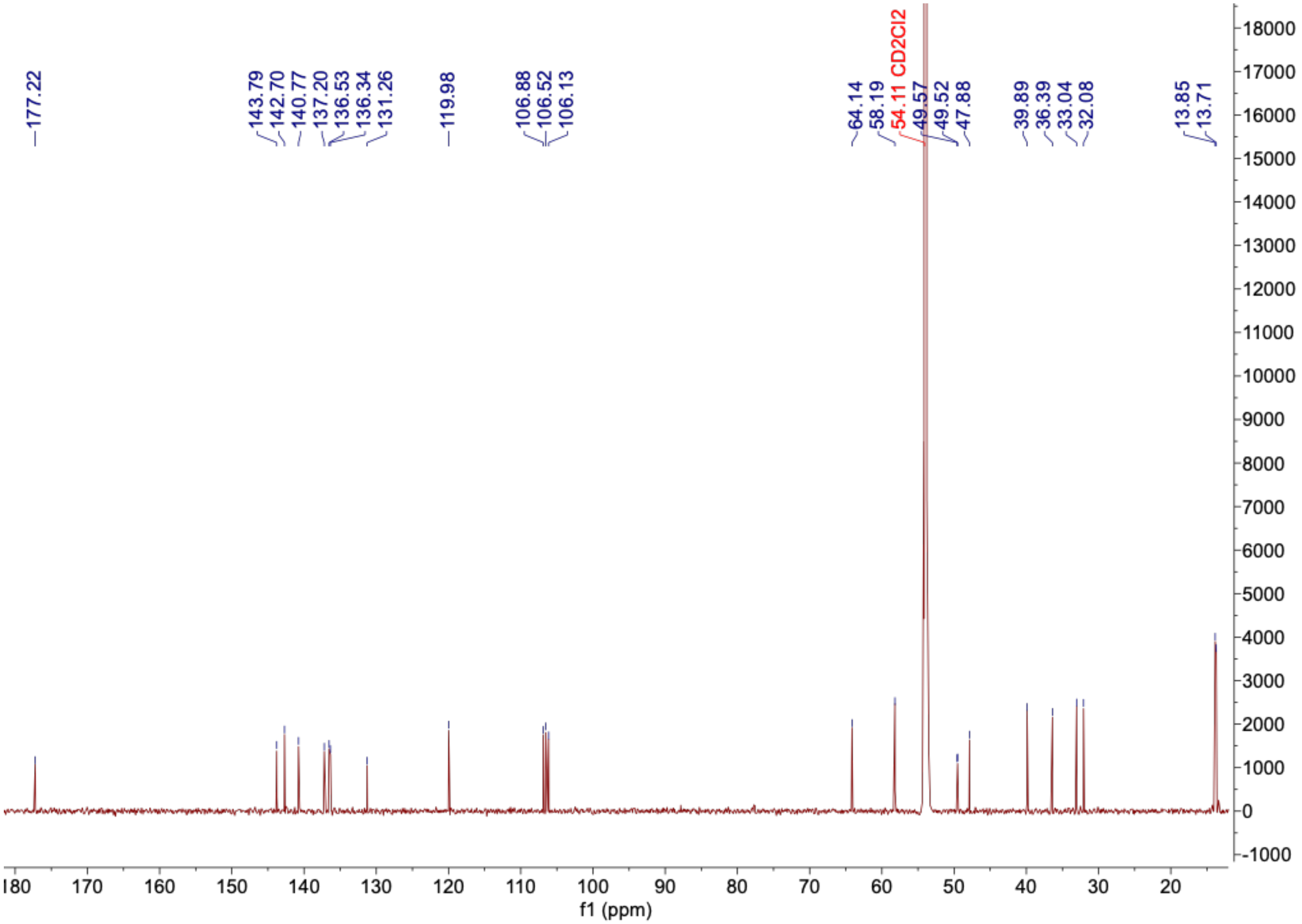

Supplementary Fig. 56: ^{13}C-NMR (CD_2Cl) of Compound **26**.

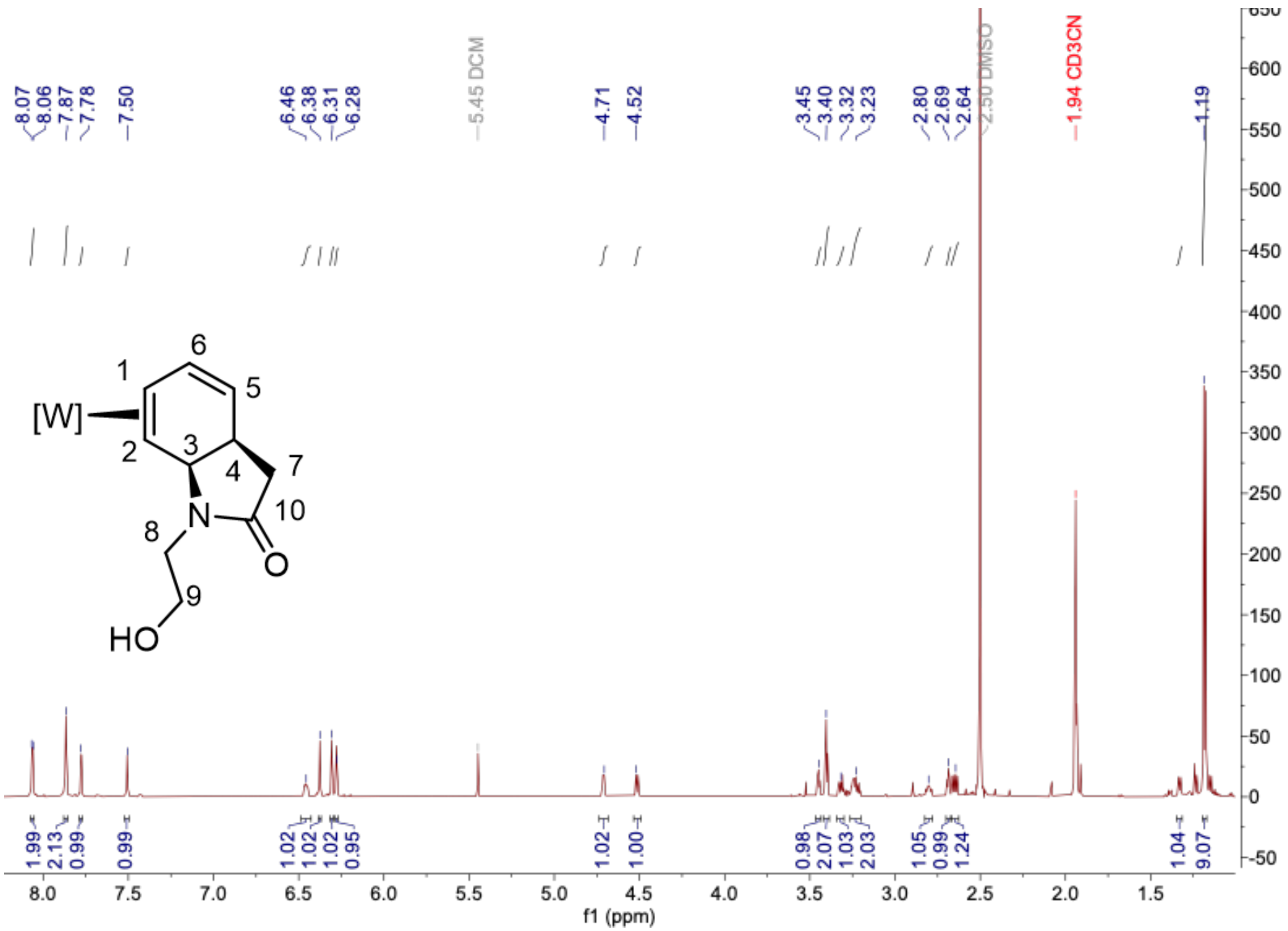

Supplementary Fig. 57: ^{1}H-NMR (CD_3CN) of Compound **27**.

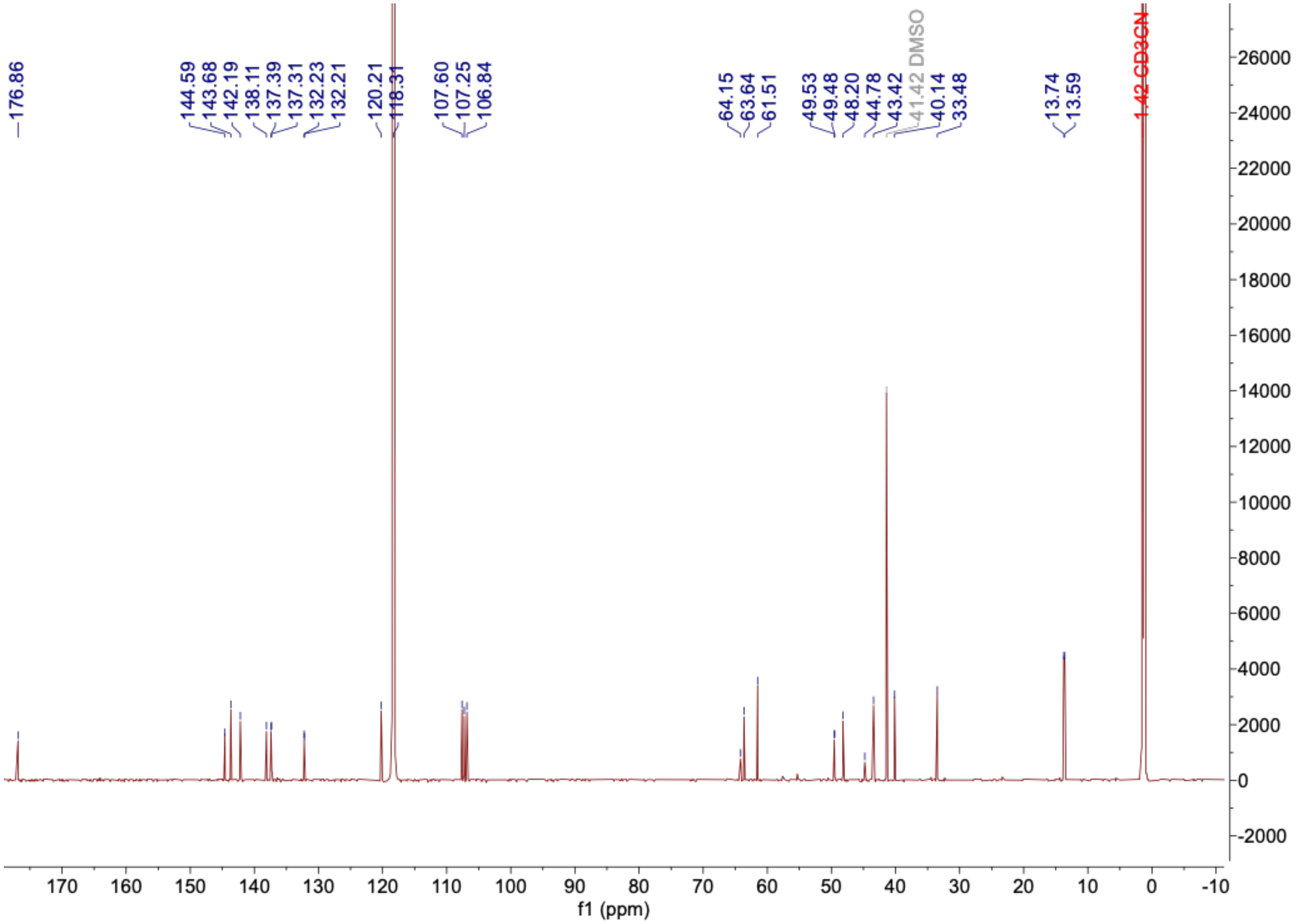

Supplementary Fig. 58: ^{13}C-NMR (CD_3CN) of Compound **27**.

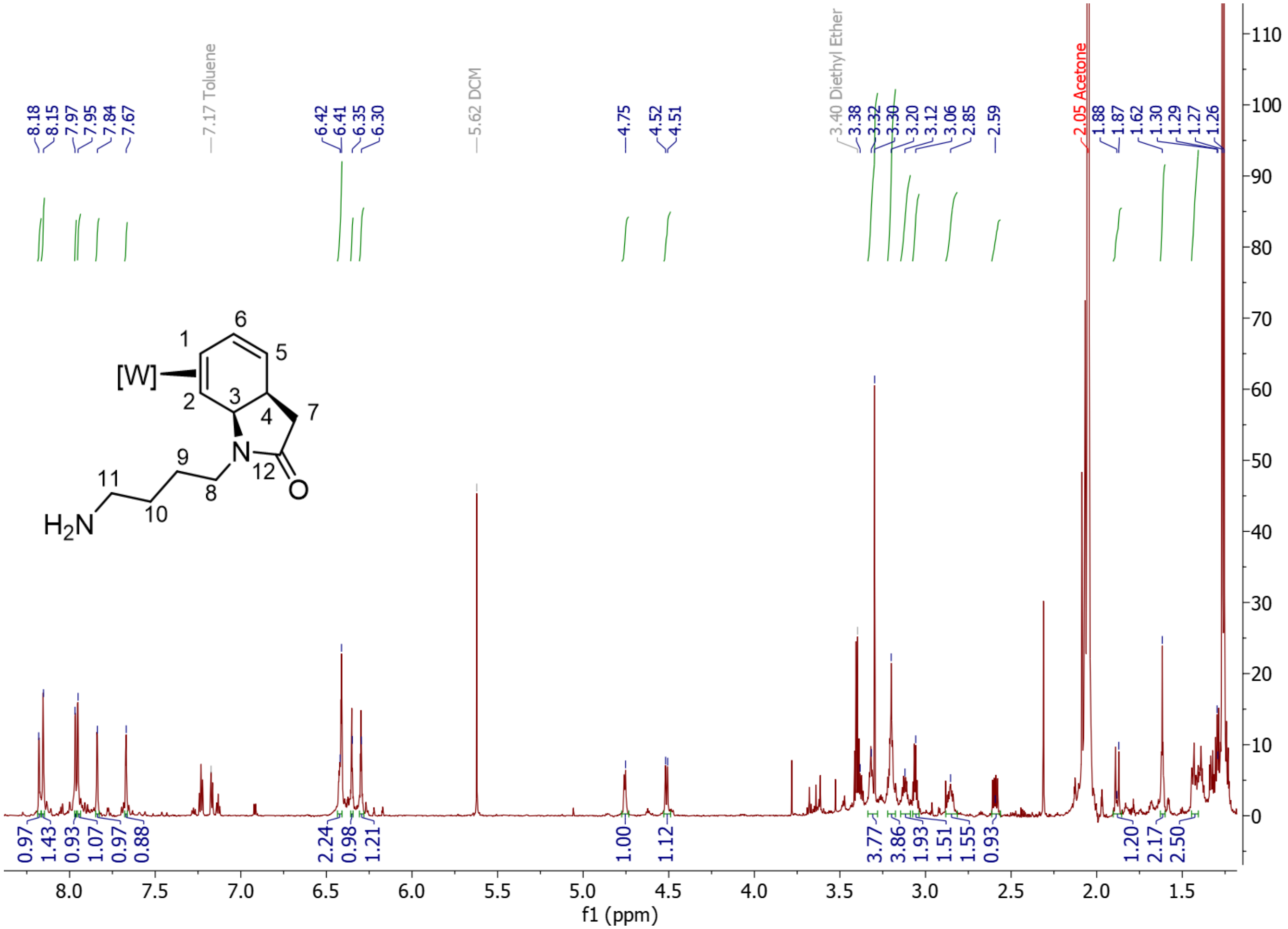

Supplementary Fig. 59: ^{1}H-NMR ($(CD_3)_2CO$) of Compound **28**.

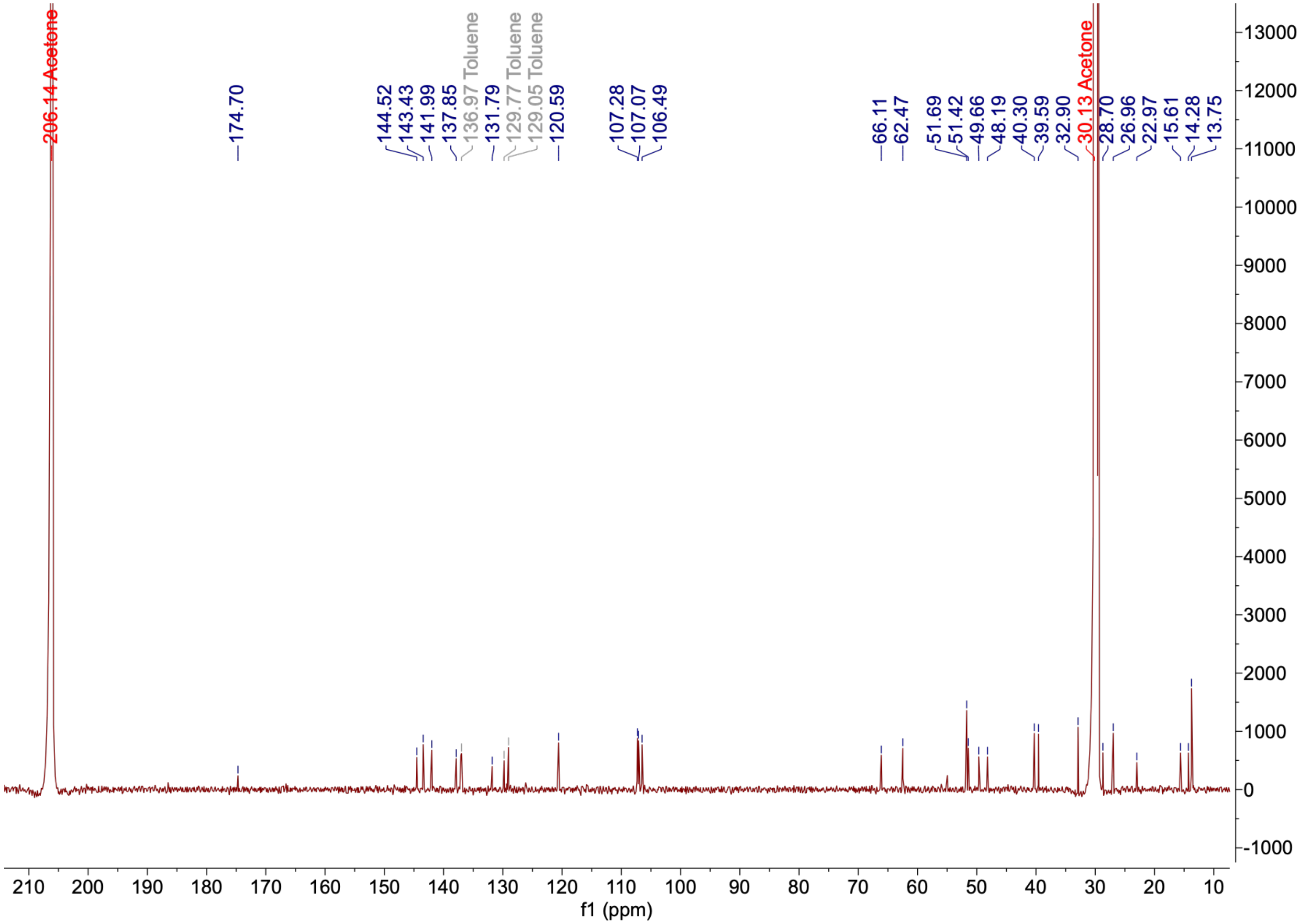

Supplementary Fig. 60: ^{13}C-NMR ($(CD_3)_2CO$) of Compound **28**.

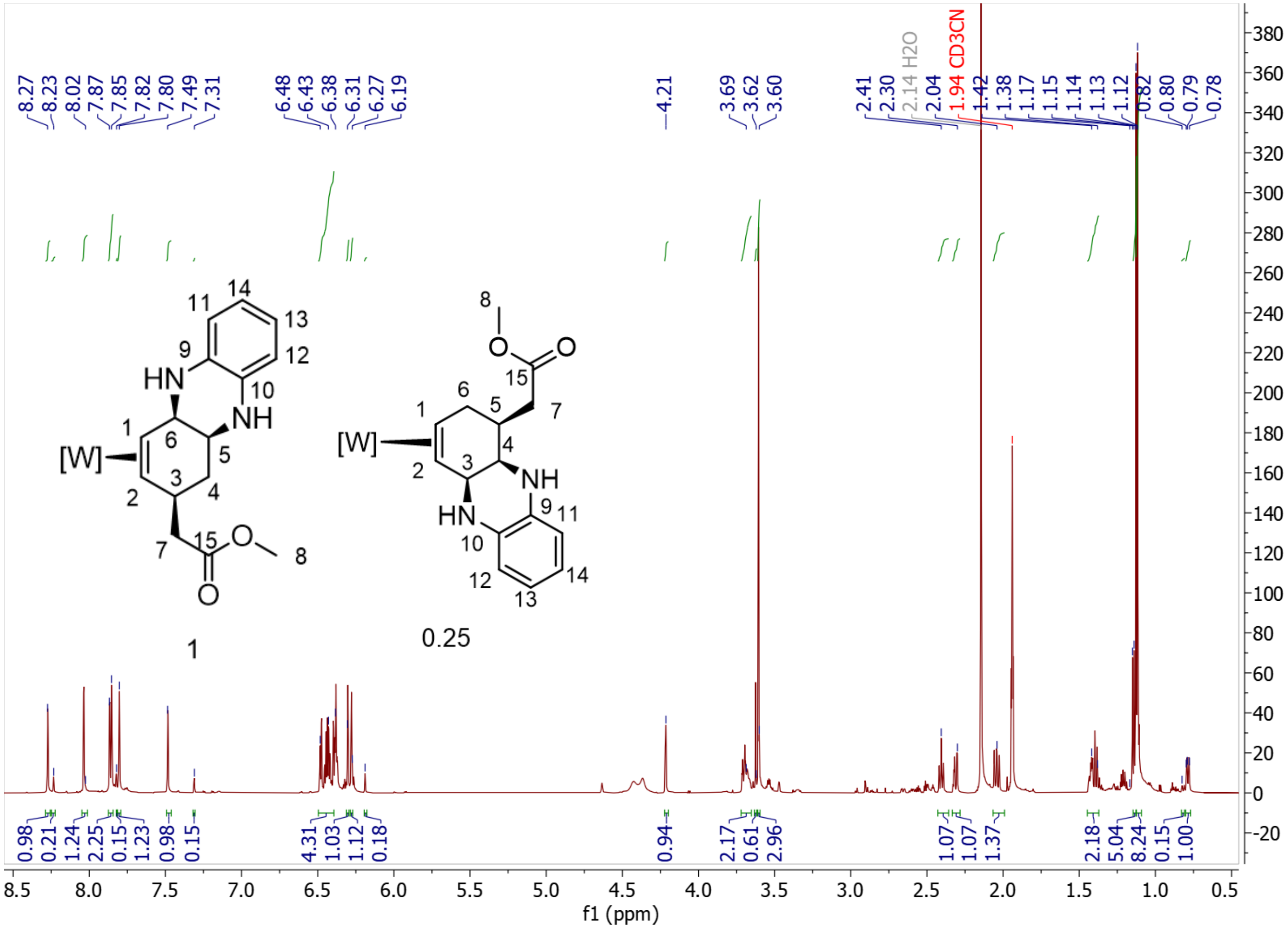

Supplementary Fig. 61: ^{1}H-NMR (CD_3CN) of Compound **29** and **31**.

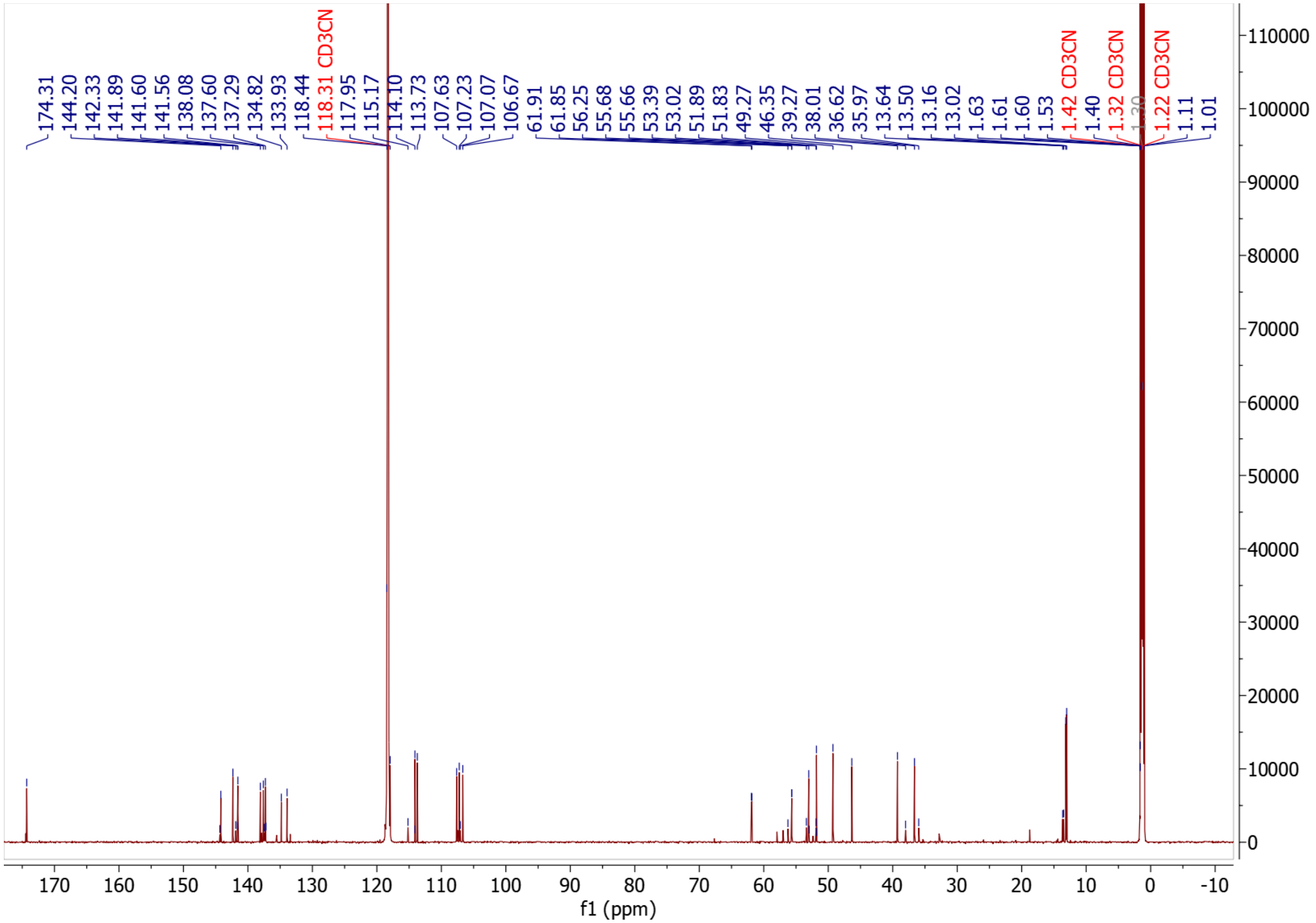

Supplementary Fig. 62: ^{13}C-NMR (CD_3CN) of Compound **29** and **31**.

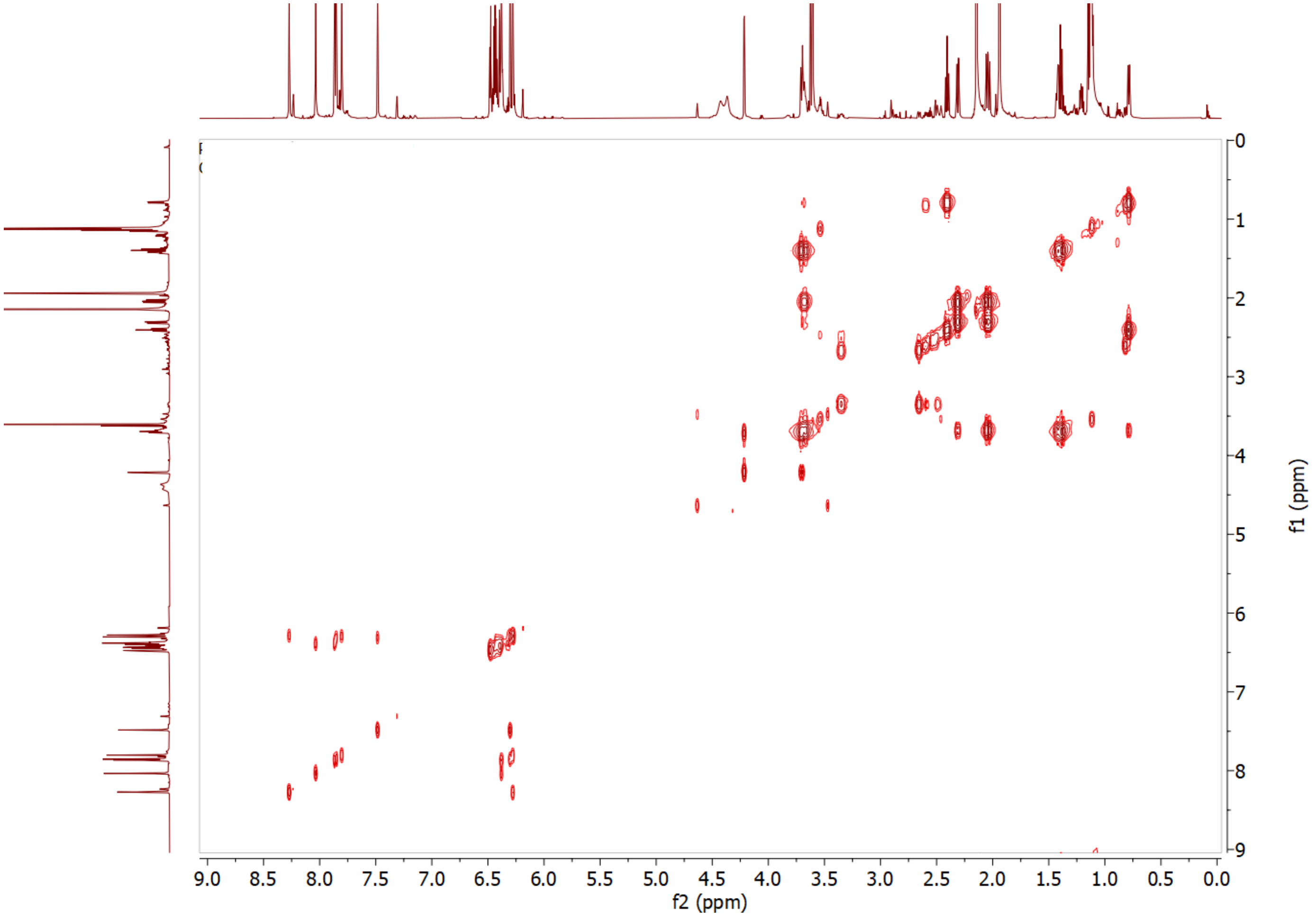

Supplementary Fig. 63: ^{1}H-^{1}H COSY (CD_3CN) of Compound **29** and **31**.

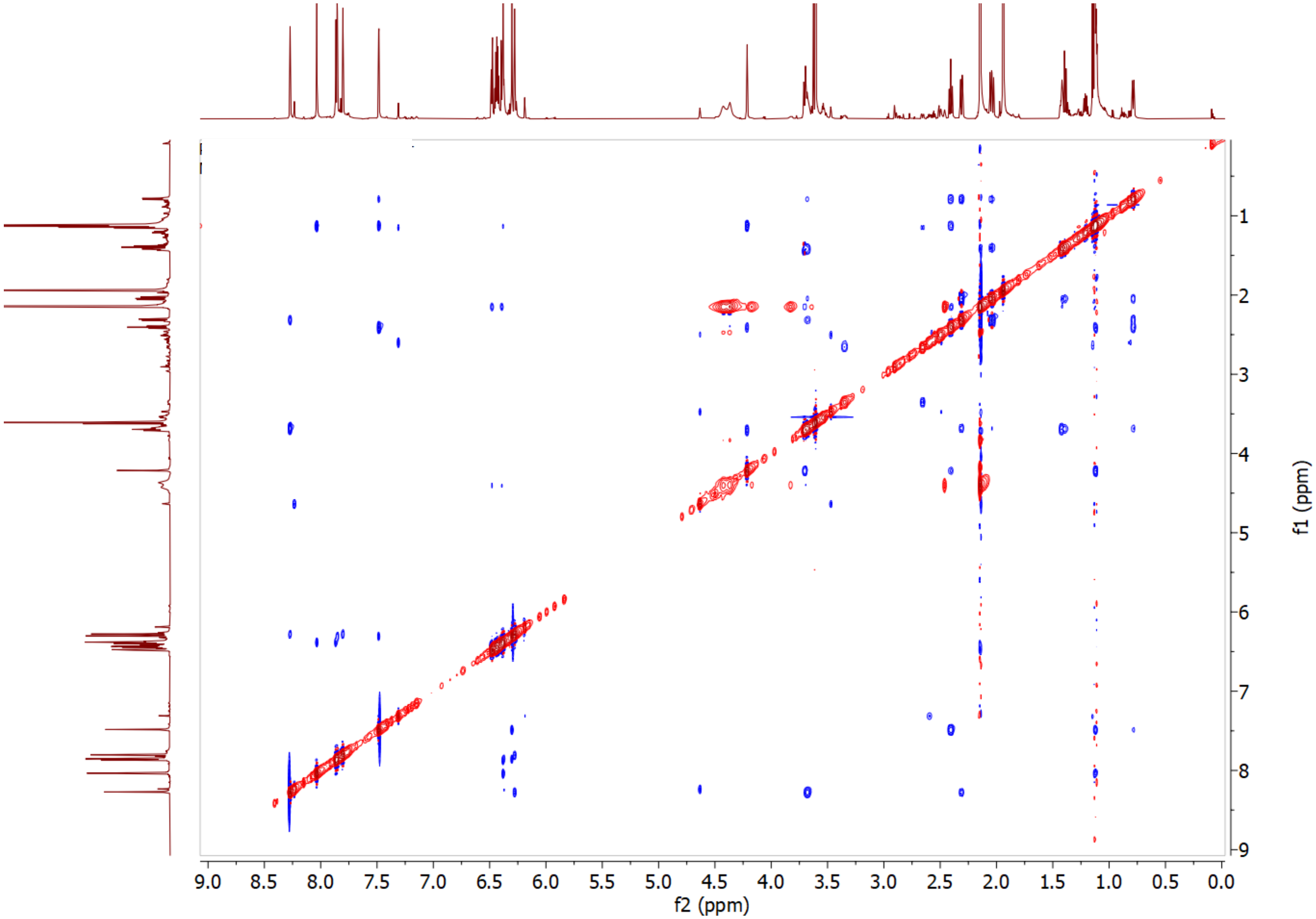

Supplementary Fig. 64: ^{1}H-^{1}H NOESY (CD_3CN) of Compound **29** and **31**.

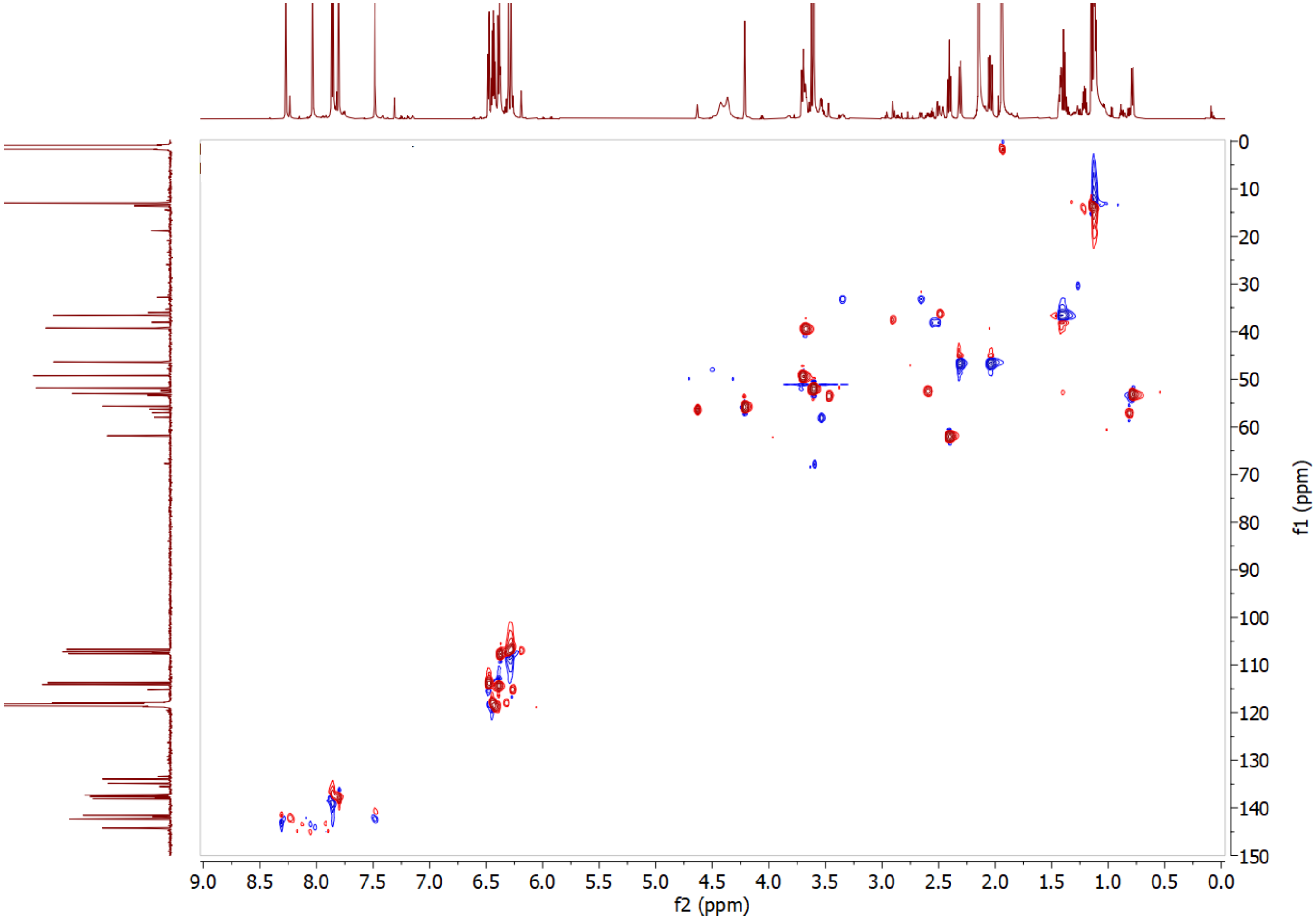

Supplementary Fig. 65: ^{1}H-^{13}C HSQC (CD_3CN) of Compound **29** and **31**.

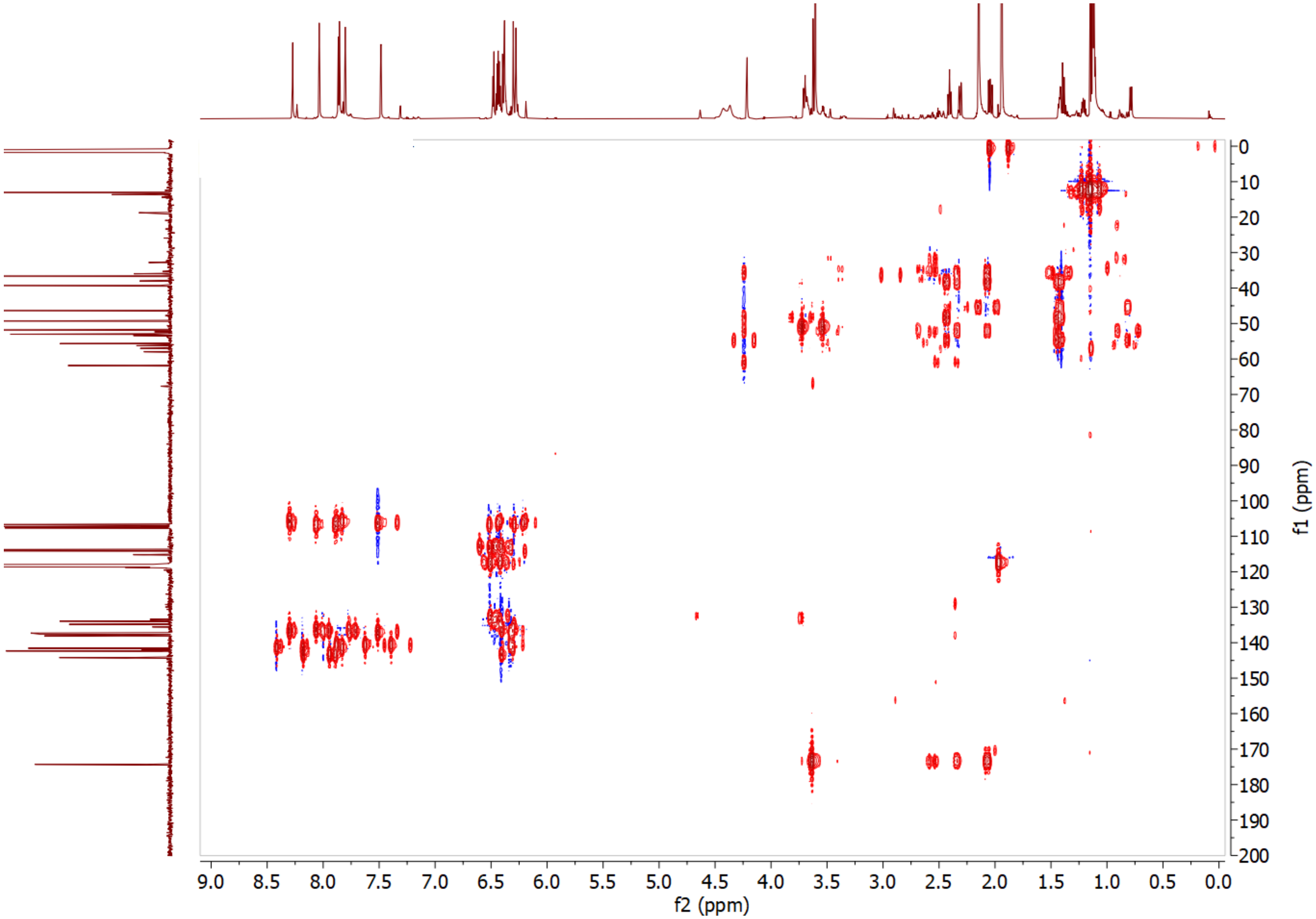

Supplementary Fig. 66: ^{1}H-^{13}C HMBC (CD_3CN) of Compound **29** and **31**.

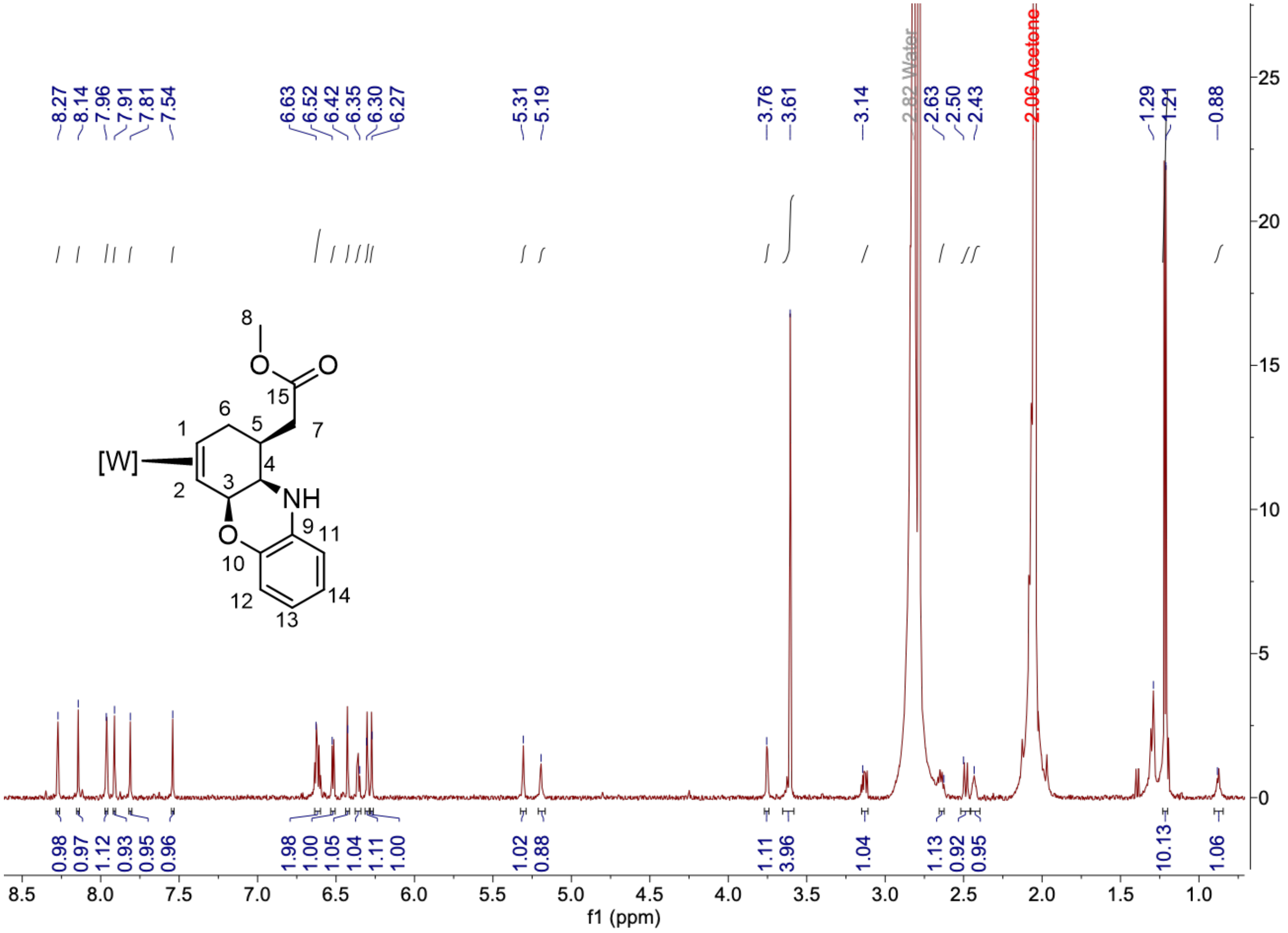

Supplementary Fig. 67: ^{1}H-NMR ($(CD_3)_2CO$) of Compound **30**.

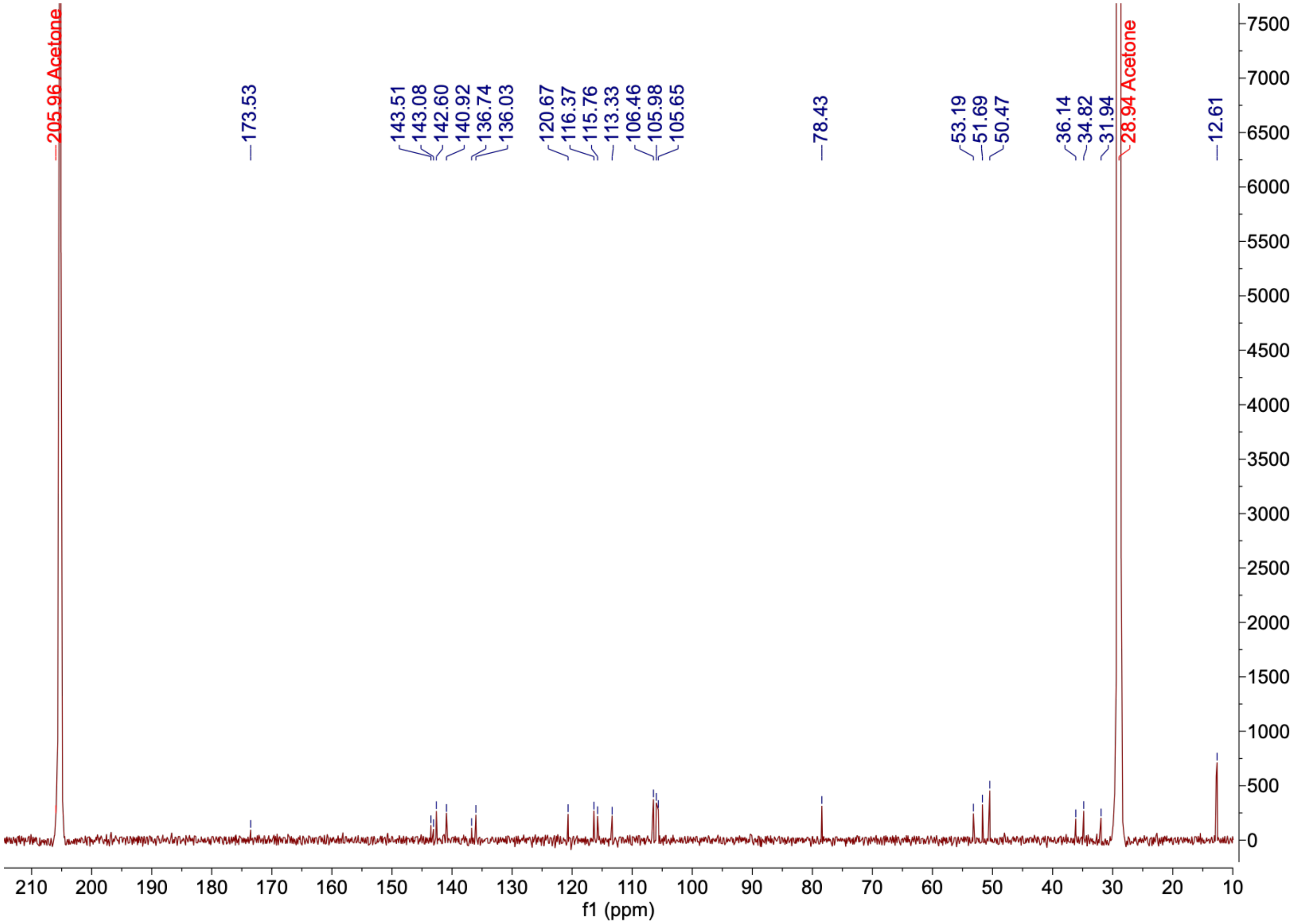

Supplementary Fig. 68: ^{13}C-NMR ($(CD_3)_2CO$) of Compound **30**.

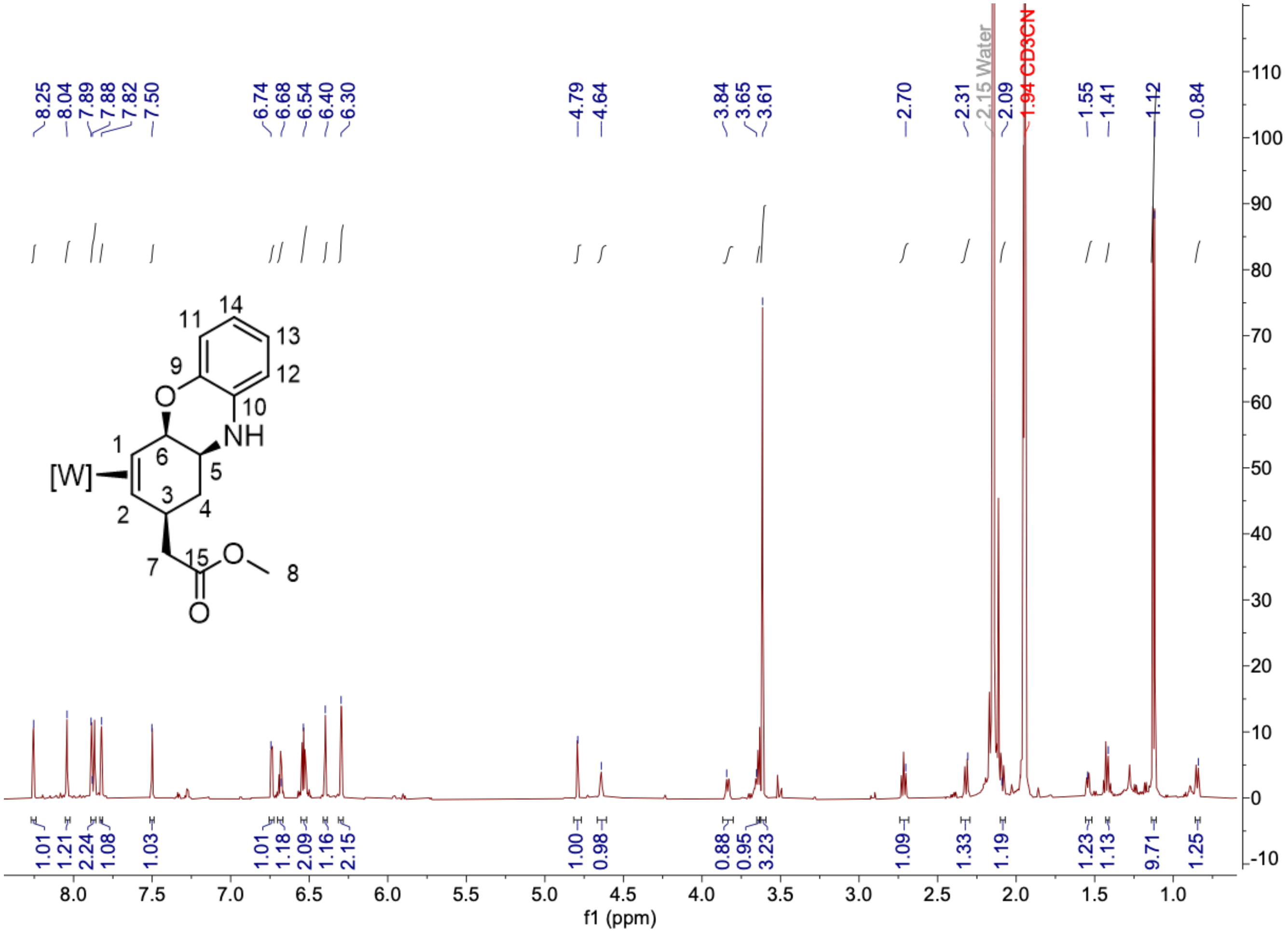

Supplementary Fig. 69: ^{1}H-NMR (CD_3CN) of Compound **32**.

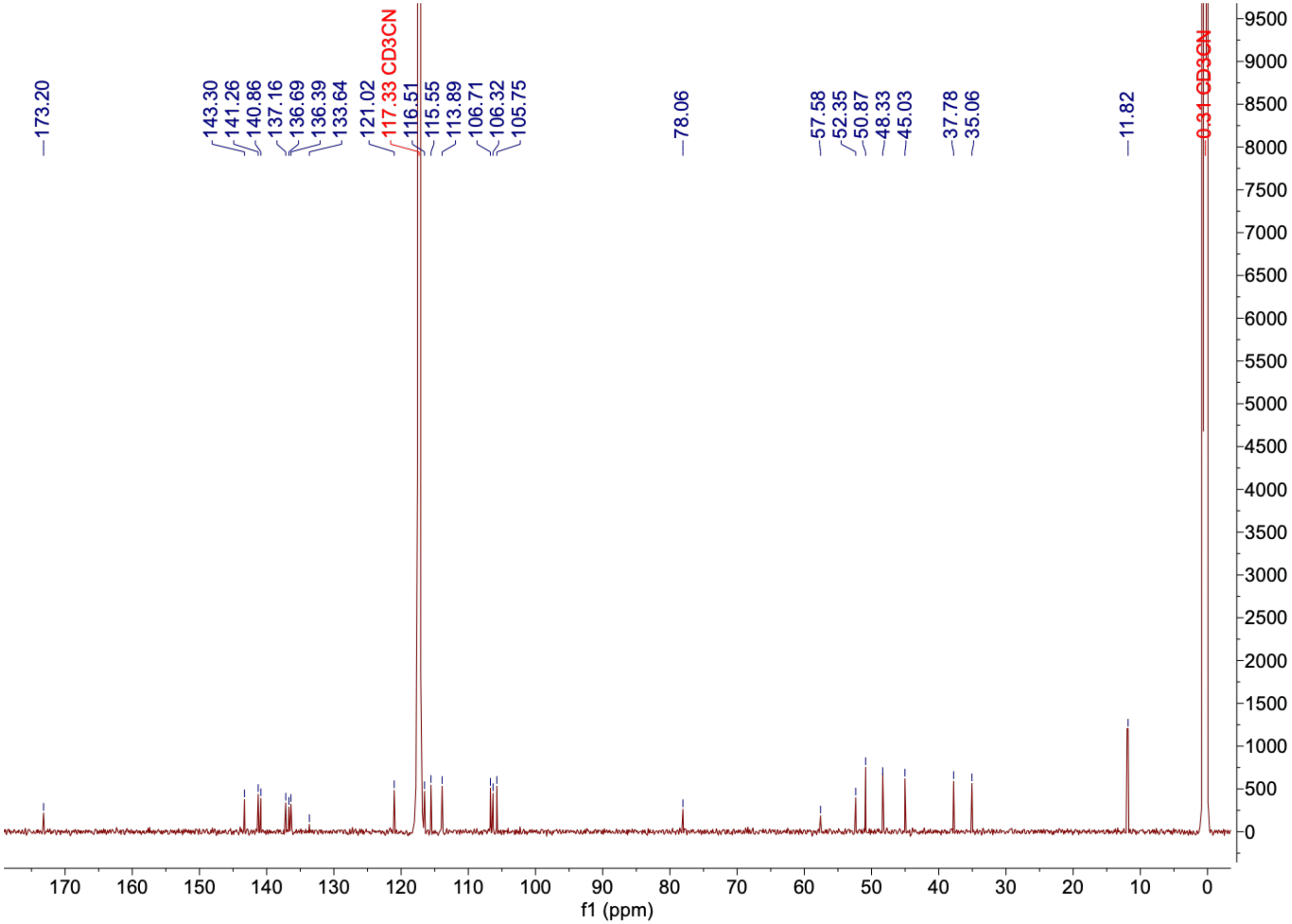

Supplementary Fig. 70: ^{13}C-NMR (CD_3CN) of Compound **32**.

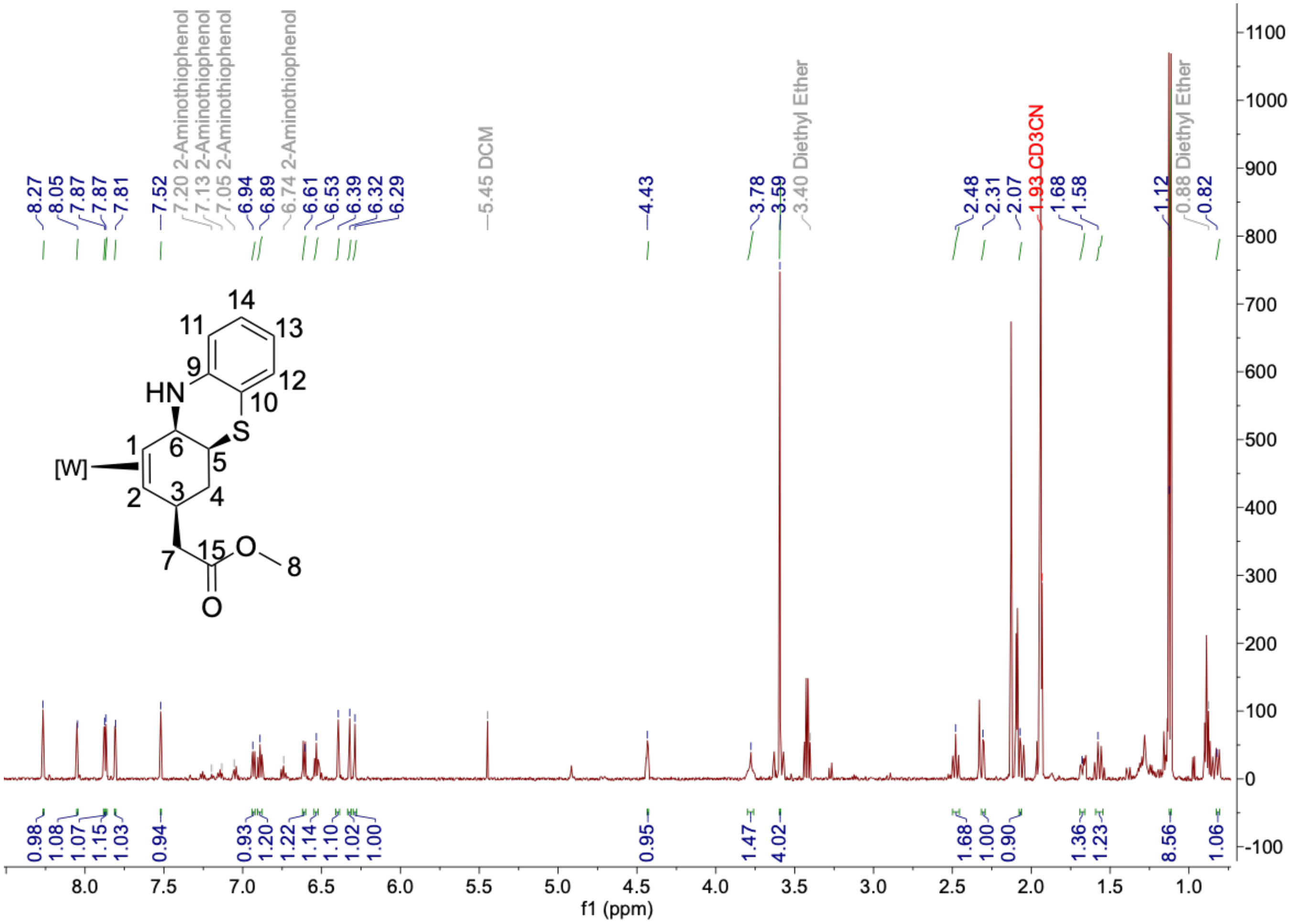

Supplementary Fig. 71: ^{1}H-NMR (CD_3CN) of Compound **33**.

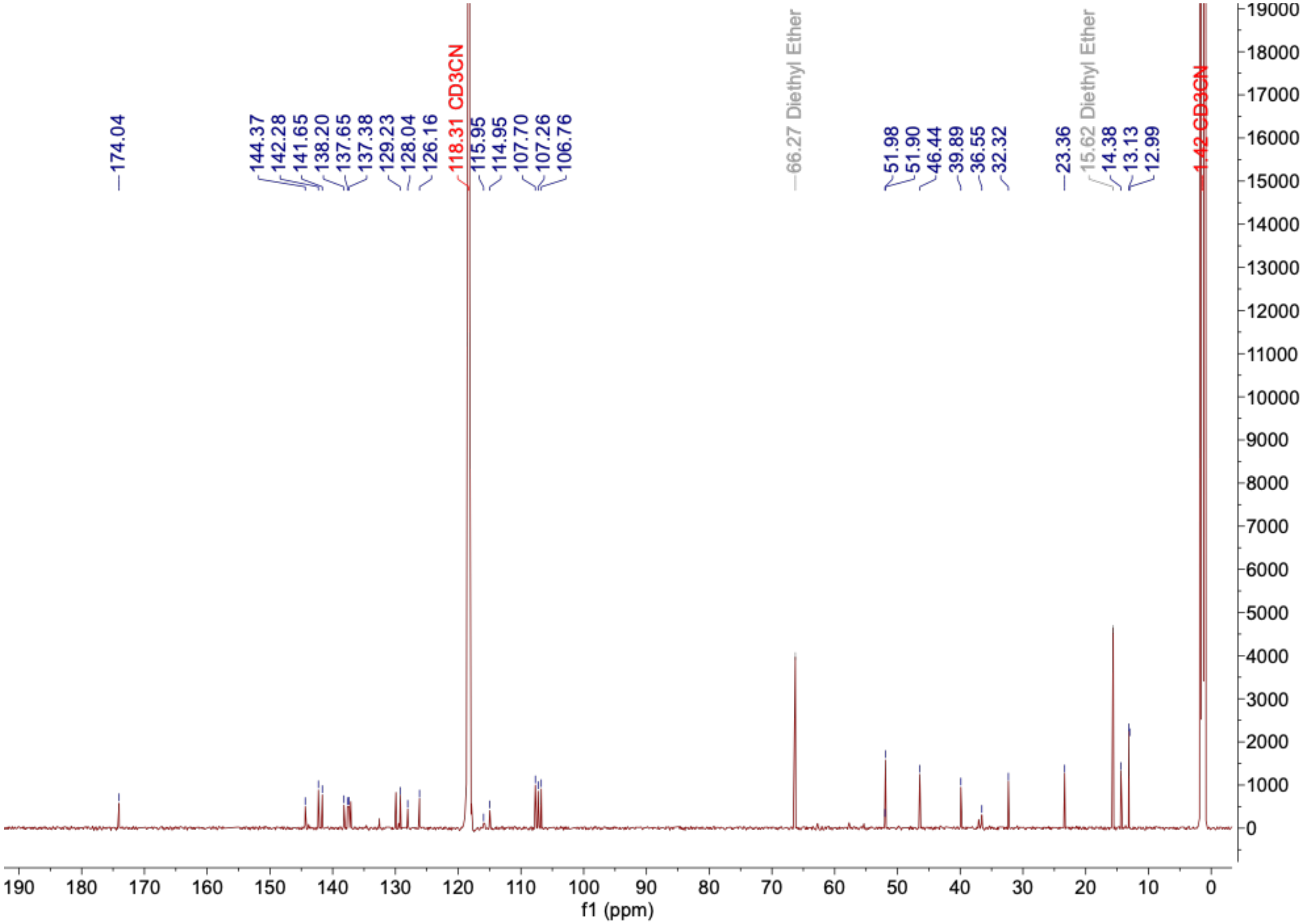

Supplementary Fig. 72: ^{13}C-NMR (CD_3CN) of Compound **33**.

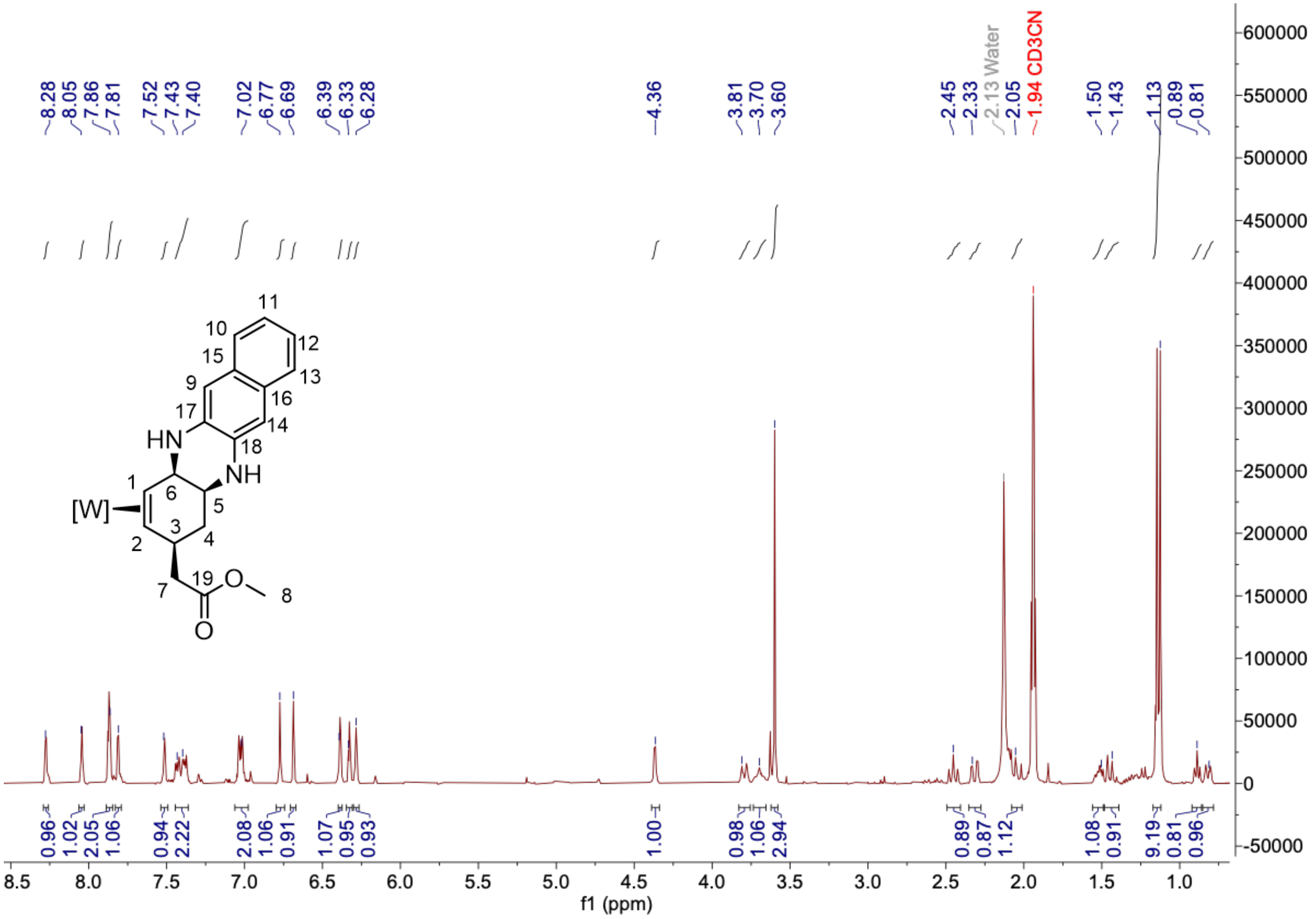

Supplementary Fig. 73: ^{1}H-NMR (CD_3CN) of Compound **34**.

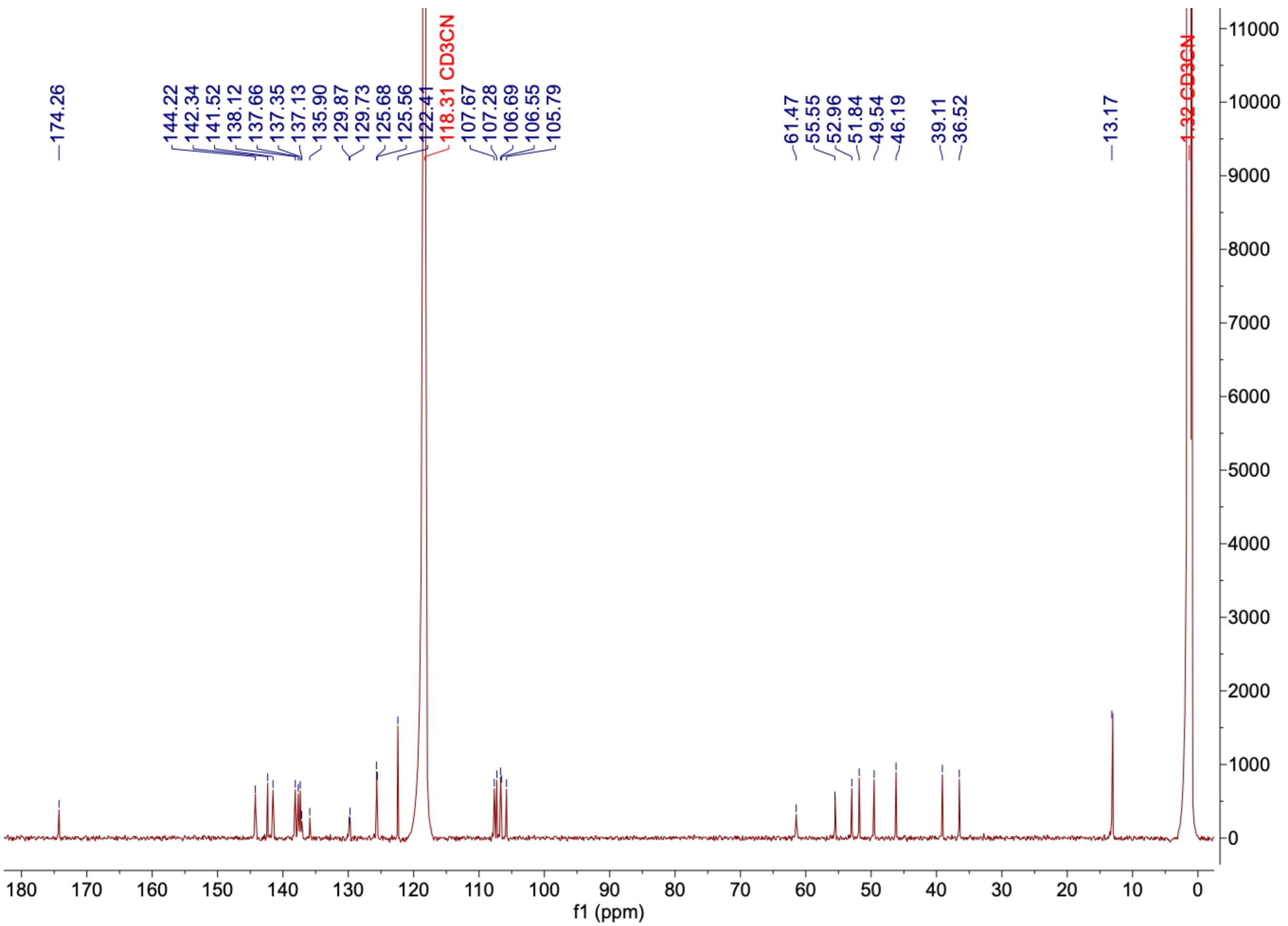

Supplementary Fig. 74: ^{13}C-NMR (CD_3CN) of Compound **34**.

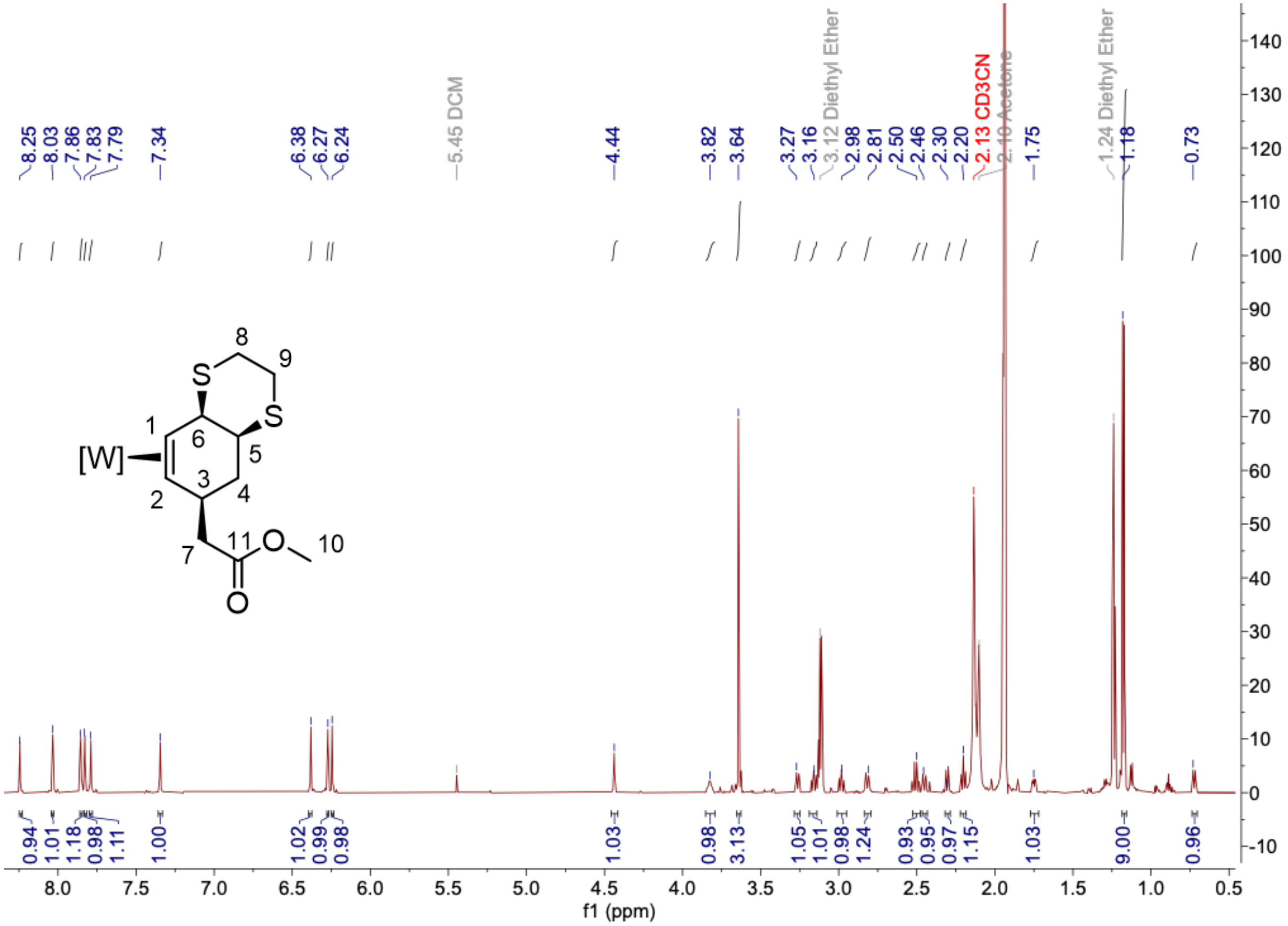

Supplementary Fig. 75: ^{1}H-NMR (CD_3CN) of Compound **35**.

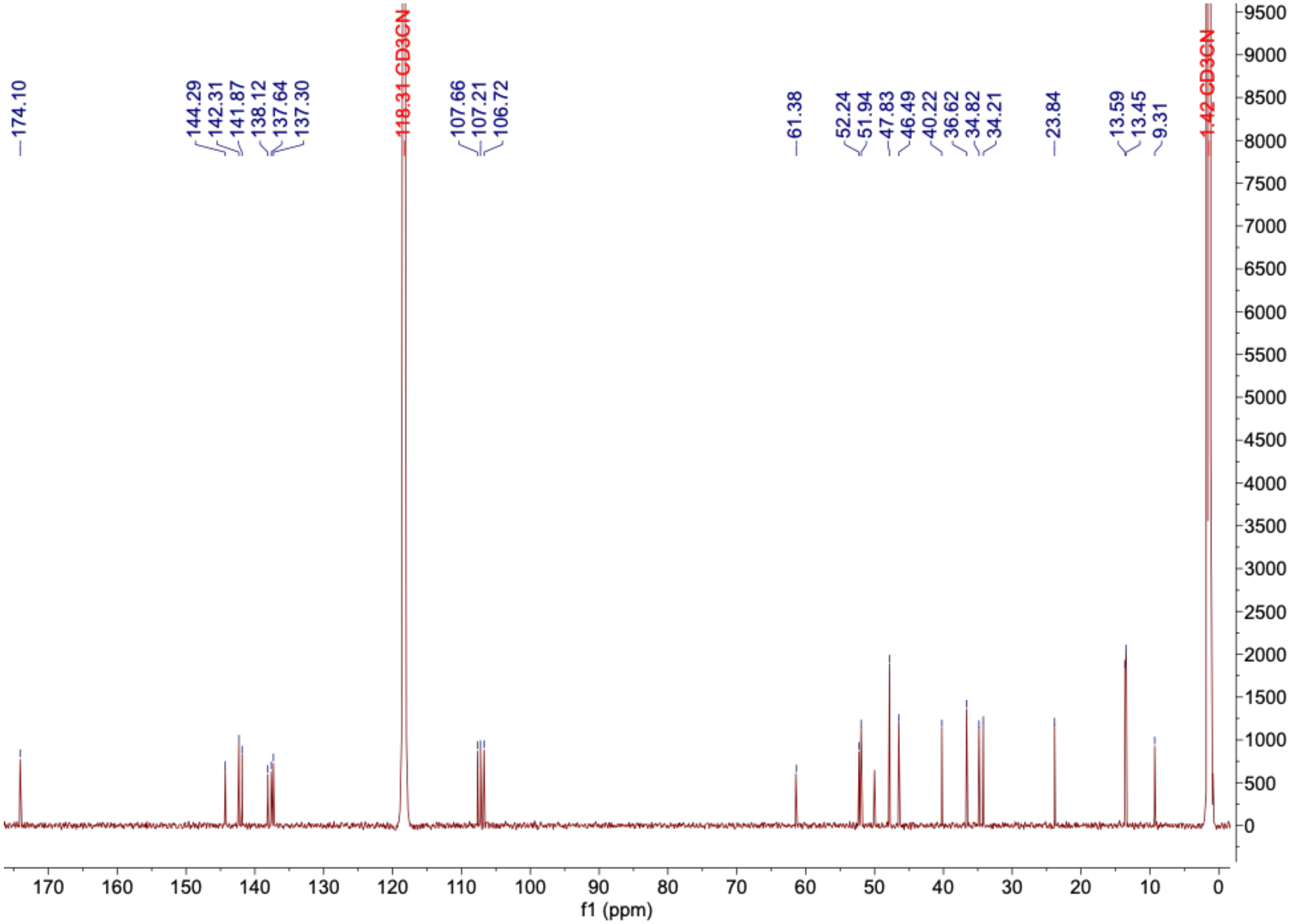

Supplementary Fig. 76: ^{13}C-NMR (CD_3CN) of Compound **35**.

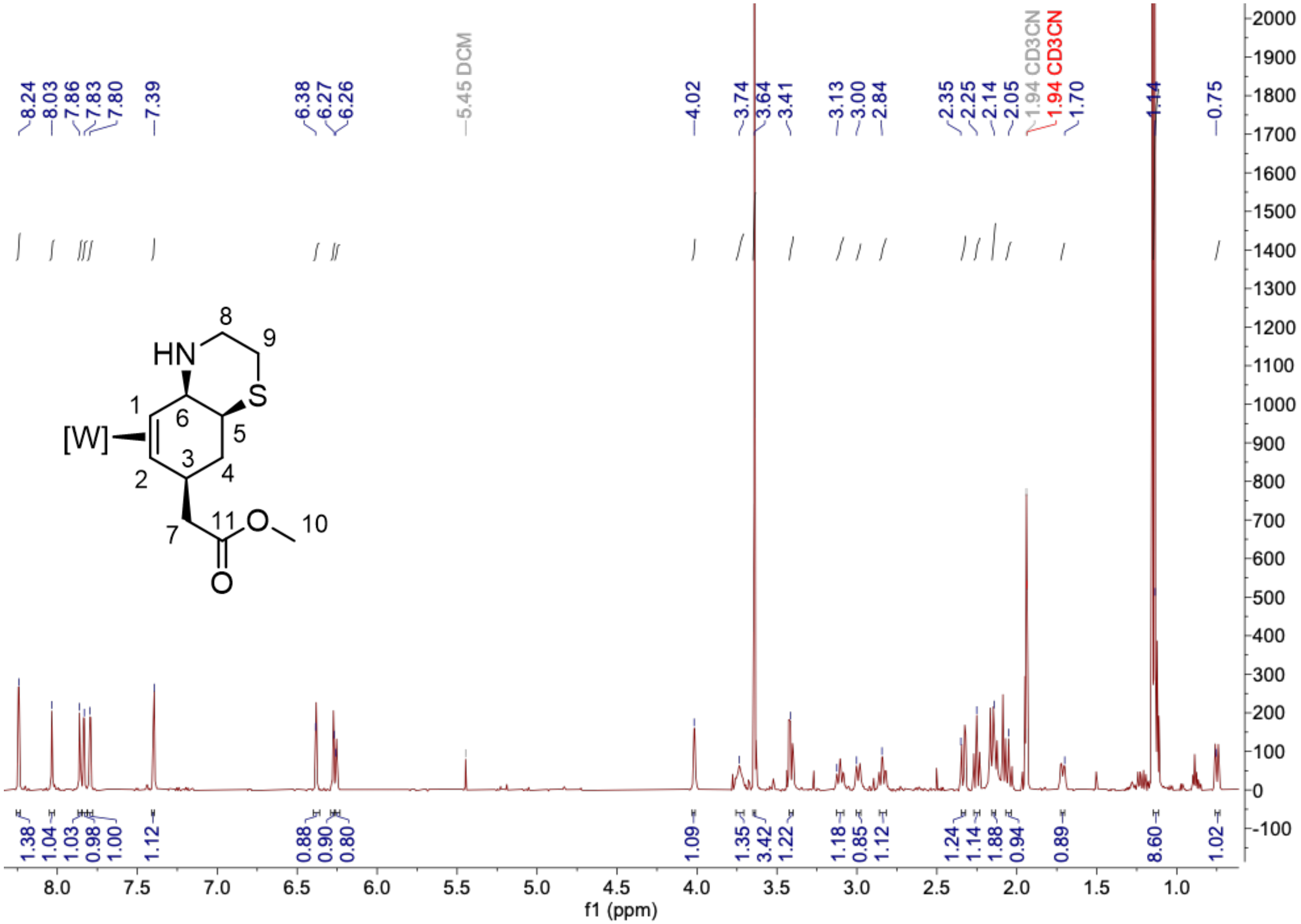

Supplementary Fig. 77: ^{1}H-NMR (CD_3CN) of Compound **36**.

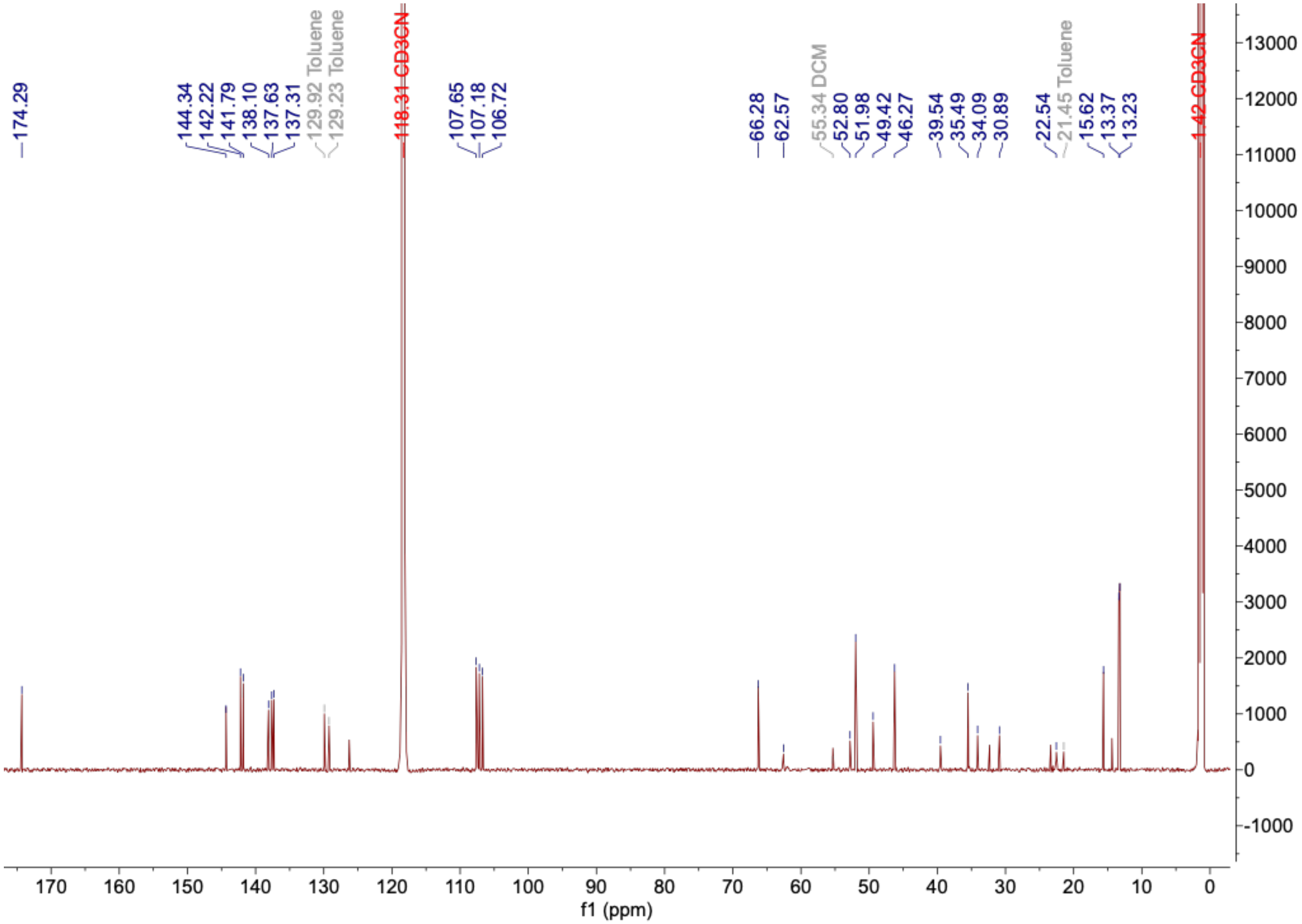

Supplementary Fig. 78: ^{13}C-NMR (CD_3CN) of Compound **36**.

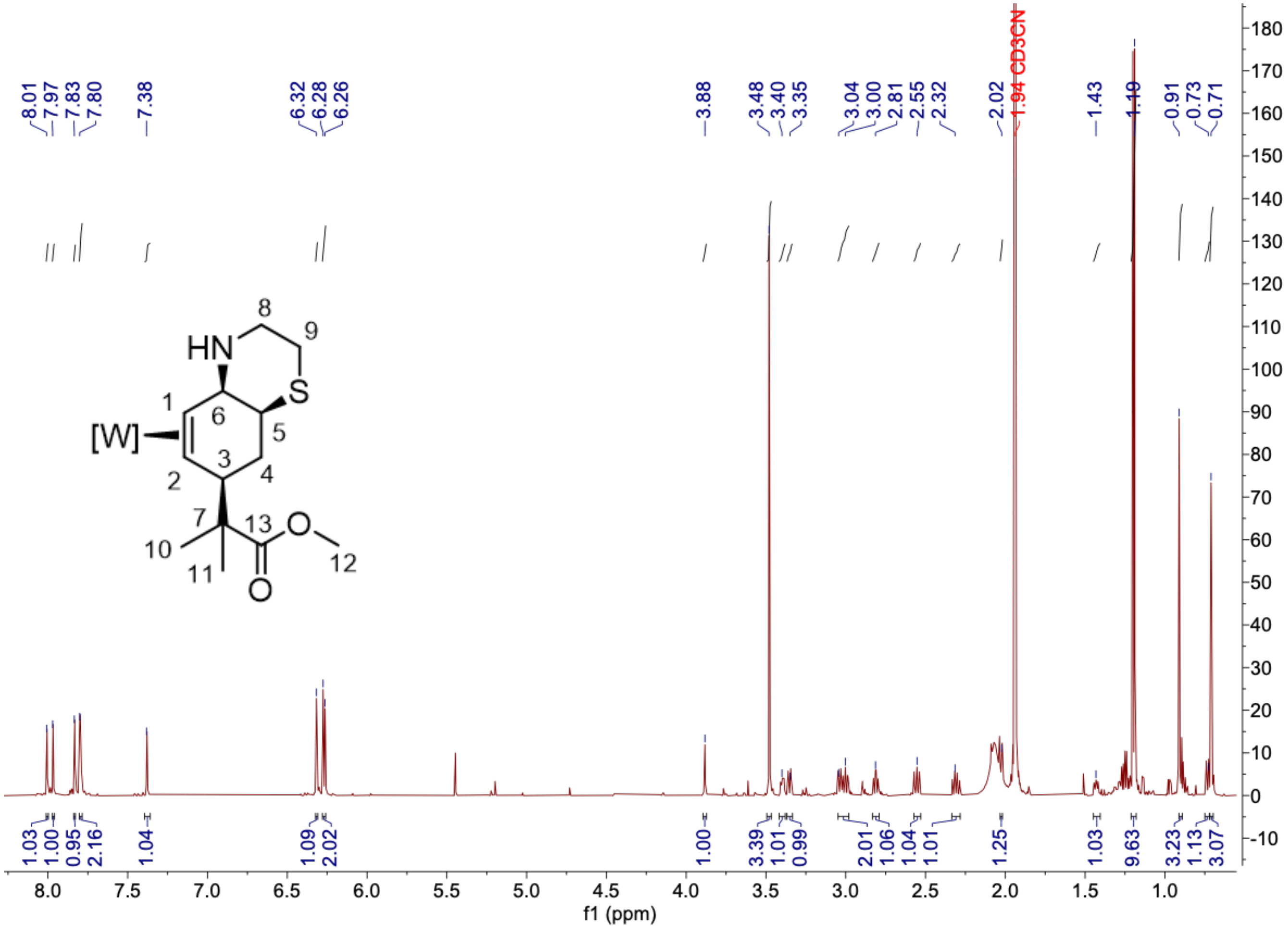

Supplementary Fig. 79: ^{1}H-NMR (CD_3CN) of Compound **37**.

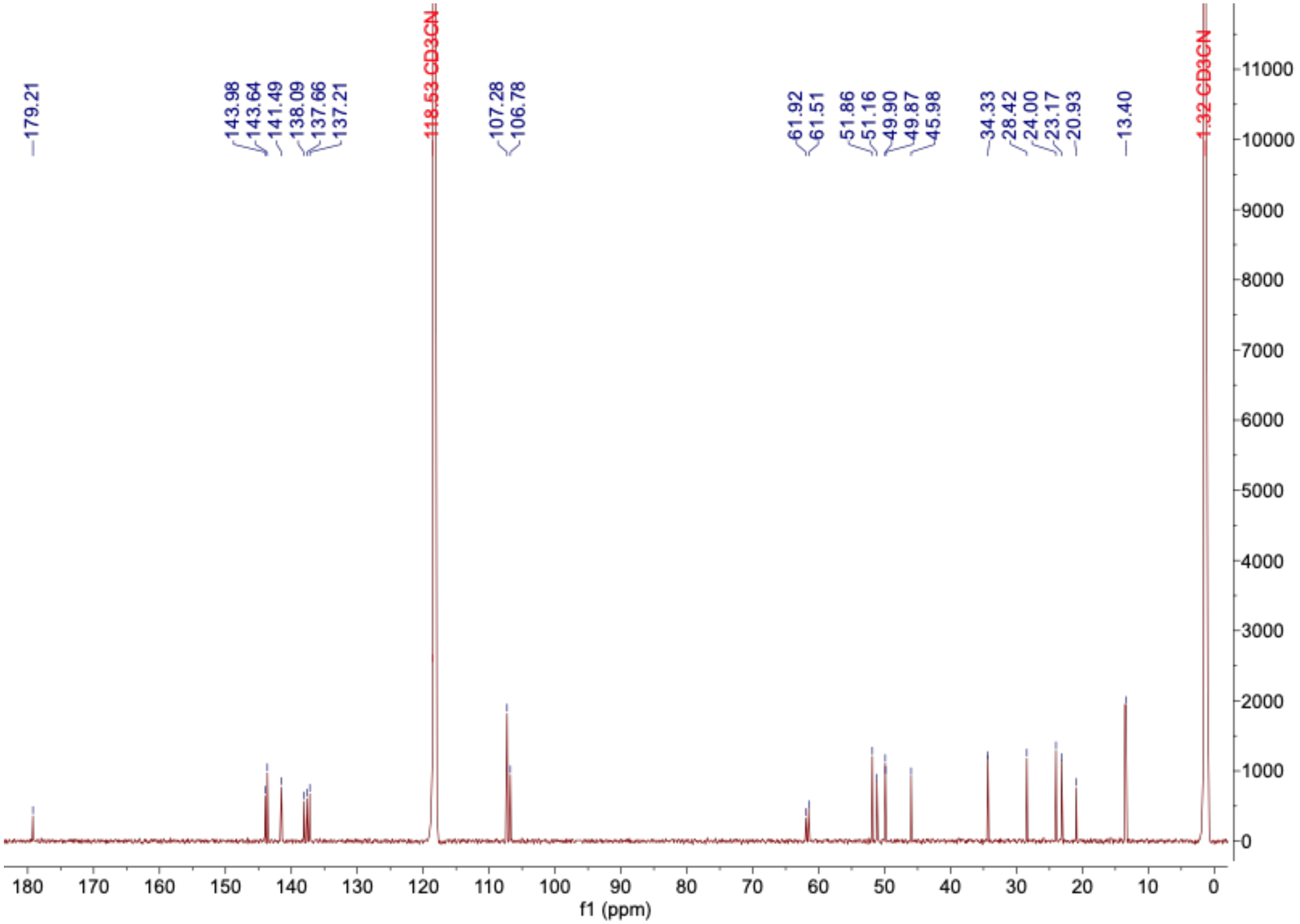

Supplementary Fig. 80: ^{13}C-NMR (CD_3CN) of Compound **37**.

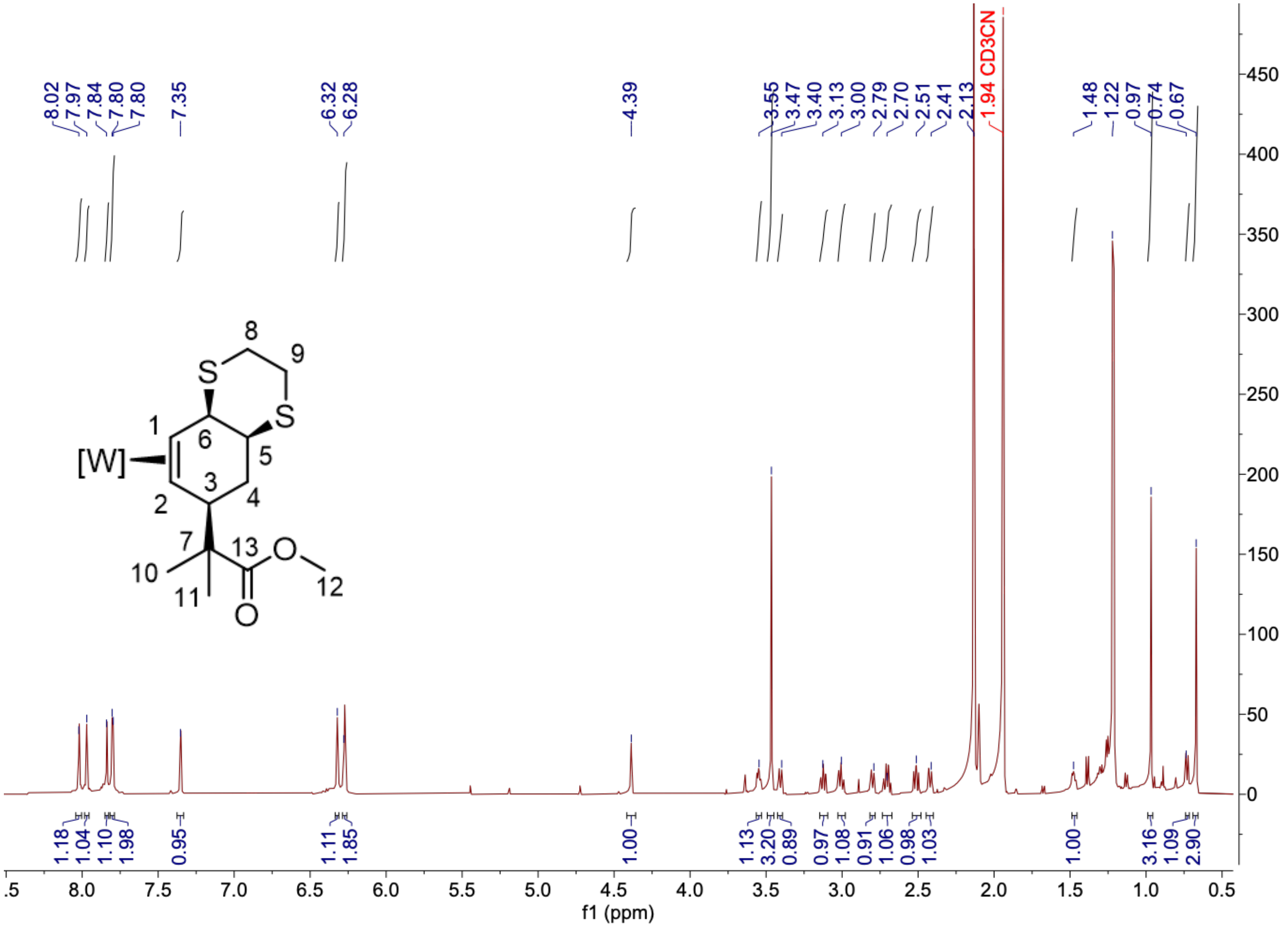

Supplementary Fig. 81: ^{1}H-NMR (CD_3CN) of Compound **38**.

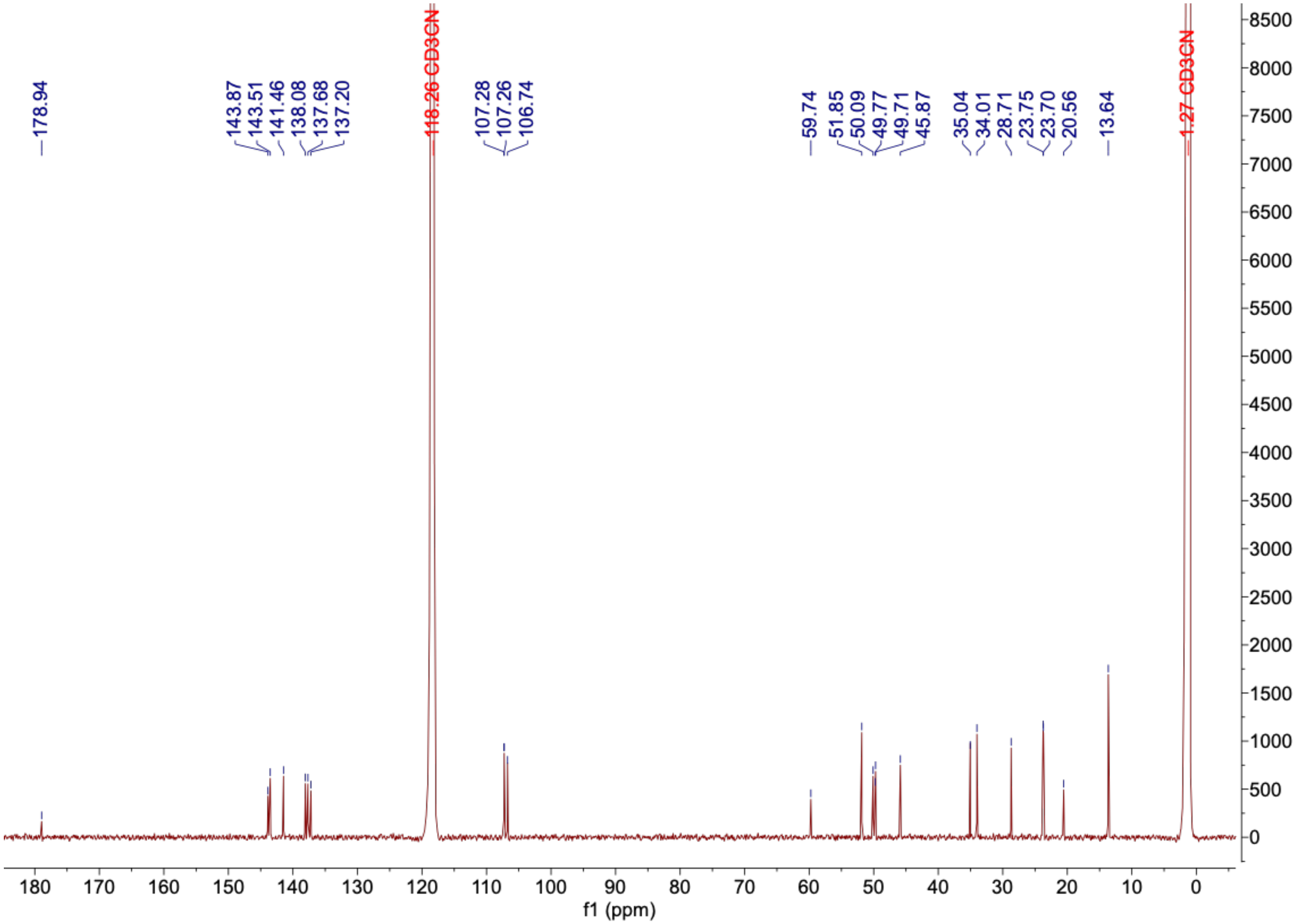

Supplementary Fig. 82: ^{13}C-NMR (CD_3CN) of Compound **38**.

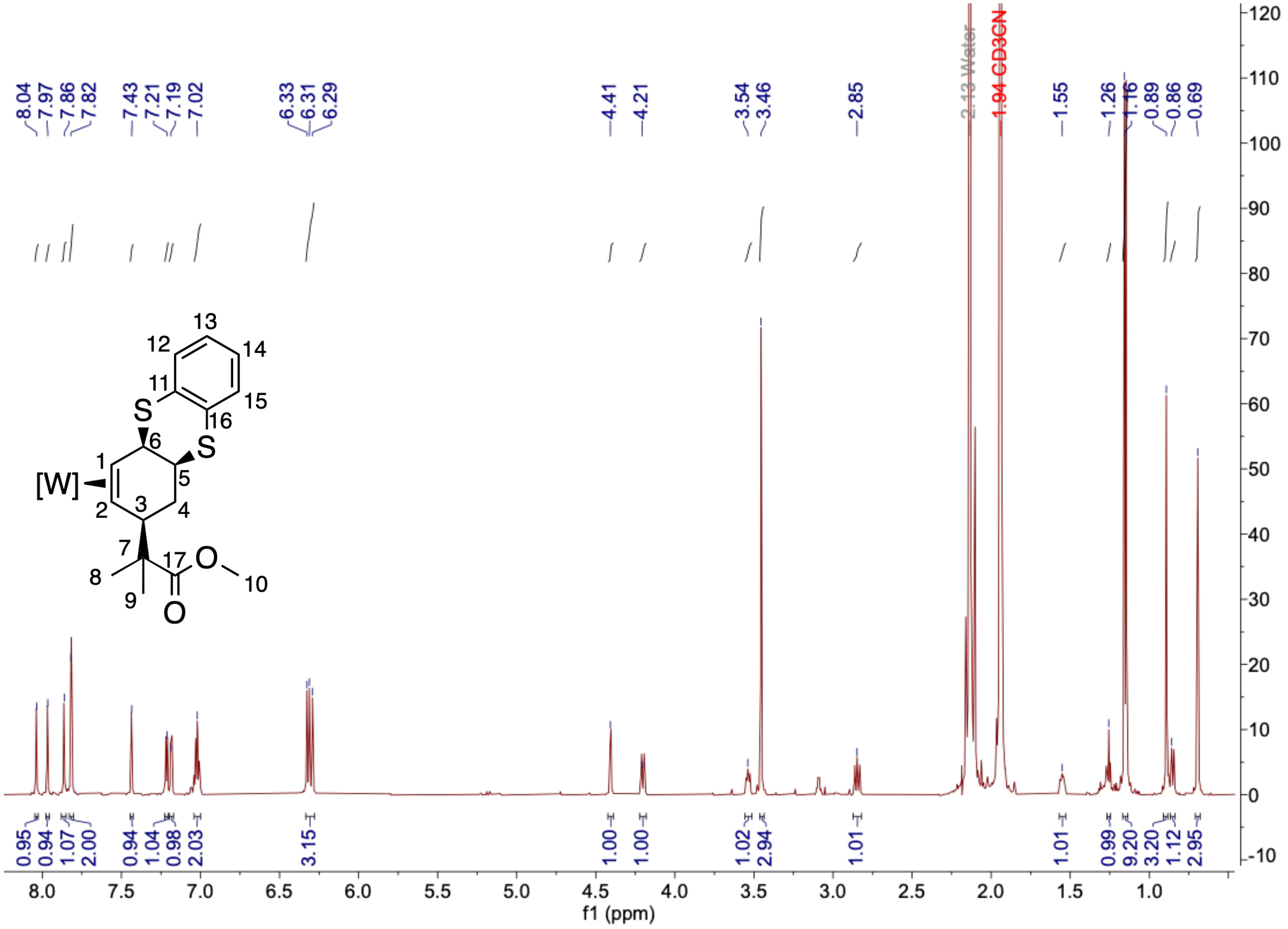

Supplementary Fig. 83: ^{1}H-NMR (CD_3CN) of Compound **39**.

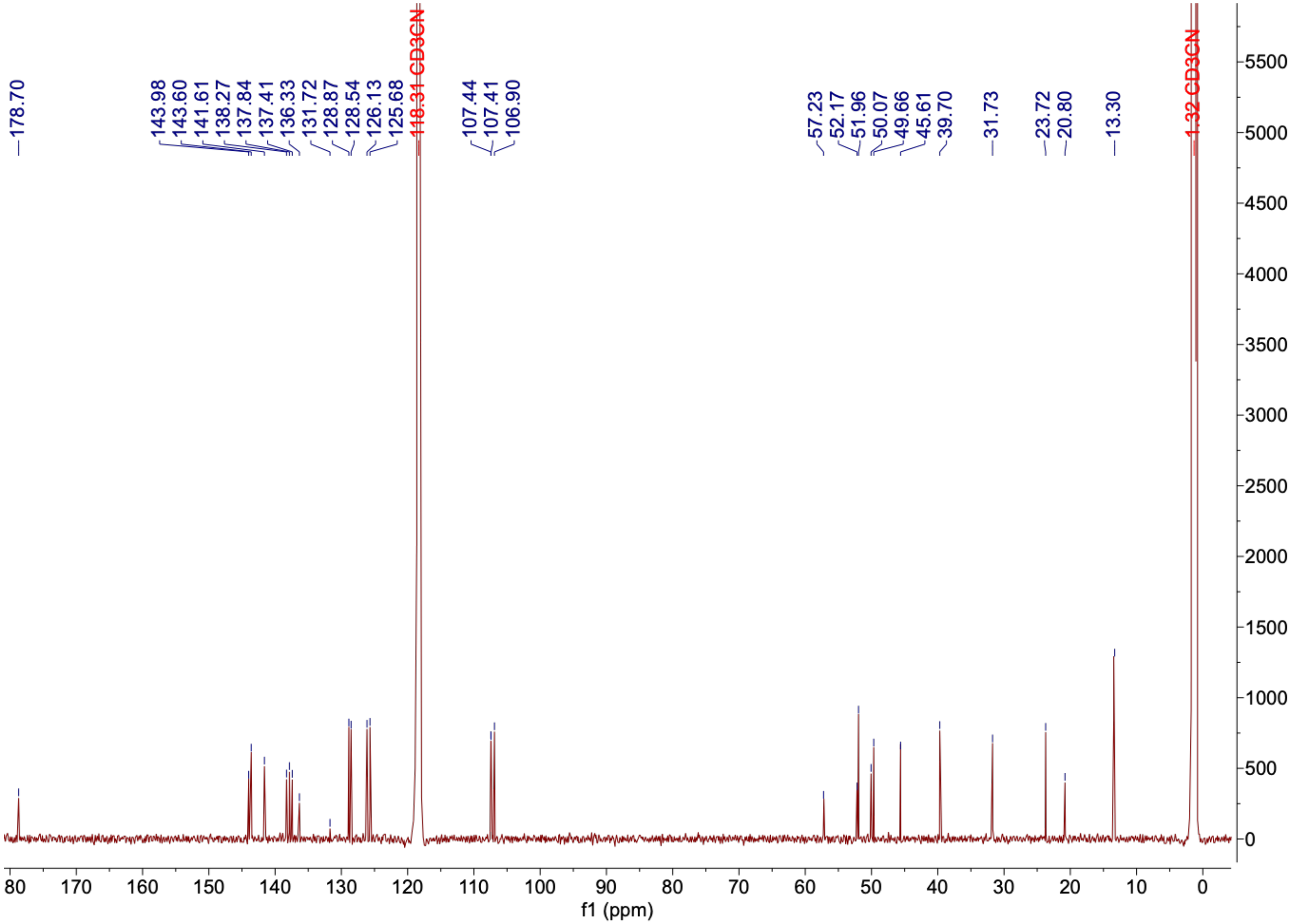

Supplementary Fig. 84: ^{13}C-NMR (CD_3CN) of Compound **39**.

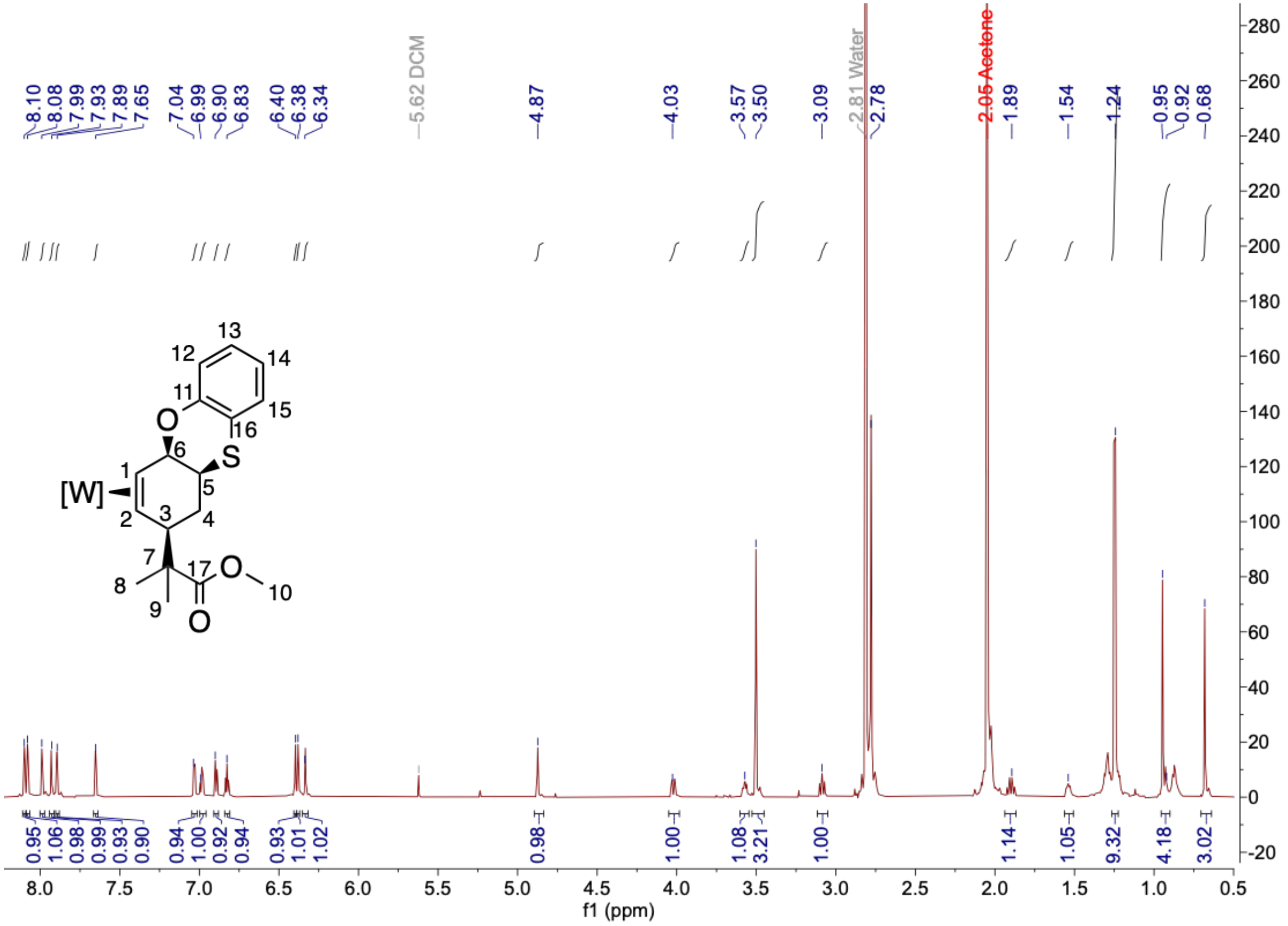

Supplementary Fig. 85: ^{1}H-NMR (CD_3CN) of Compound **40**.

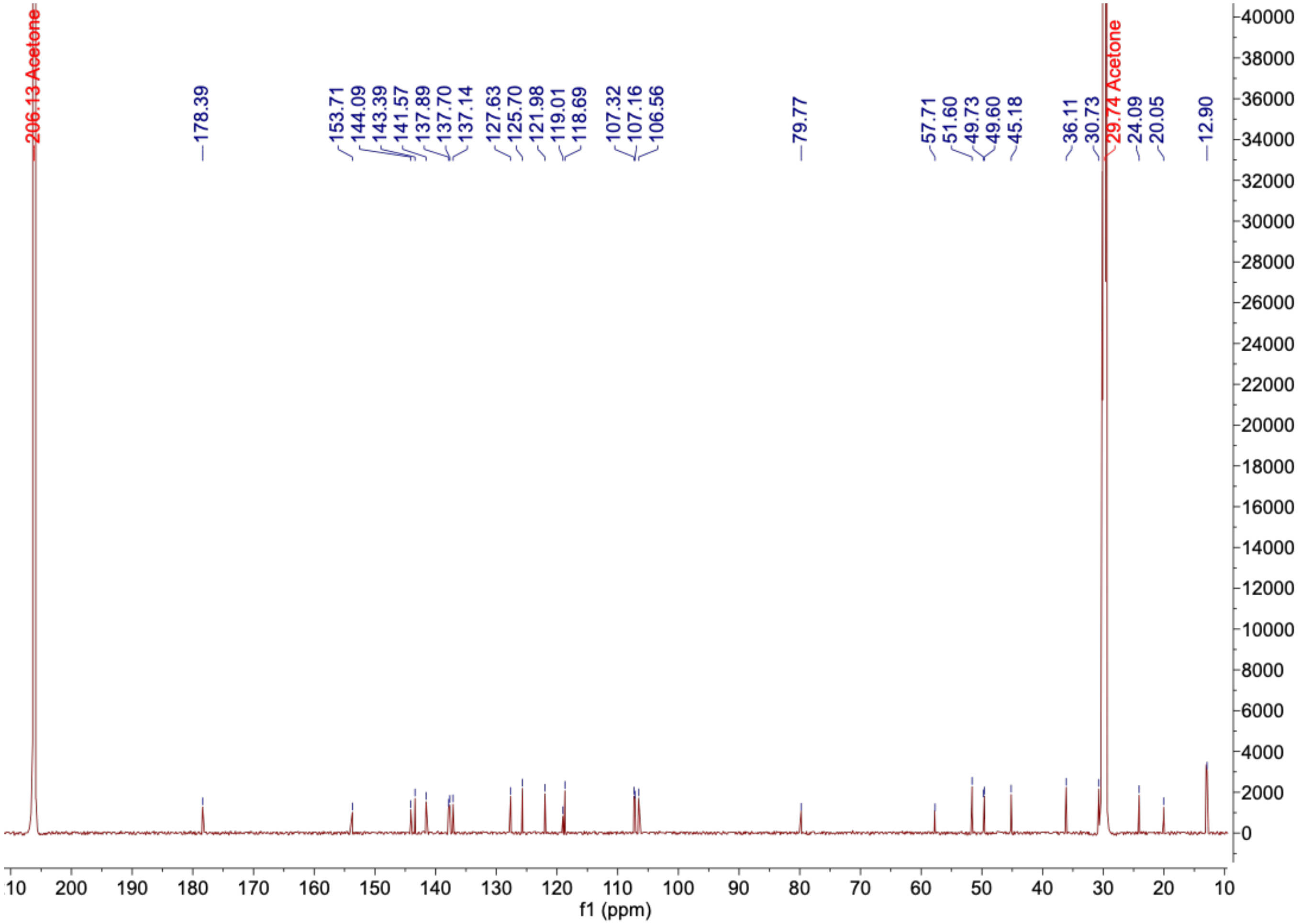

Supplementary Fig. 86: ^{13}C-NMR (CD_3CN) of Compound **40**.

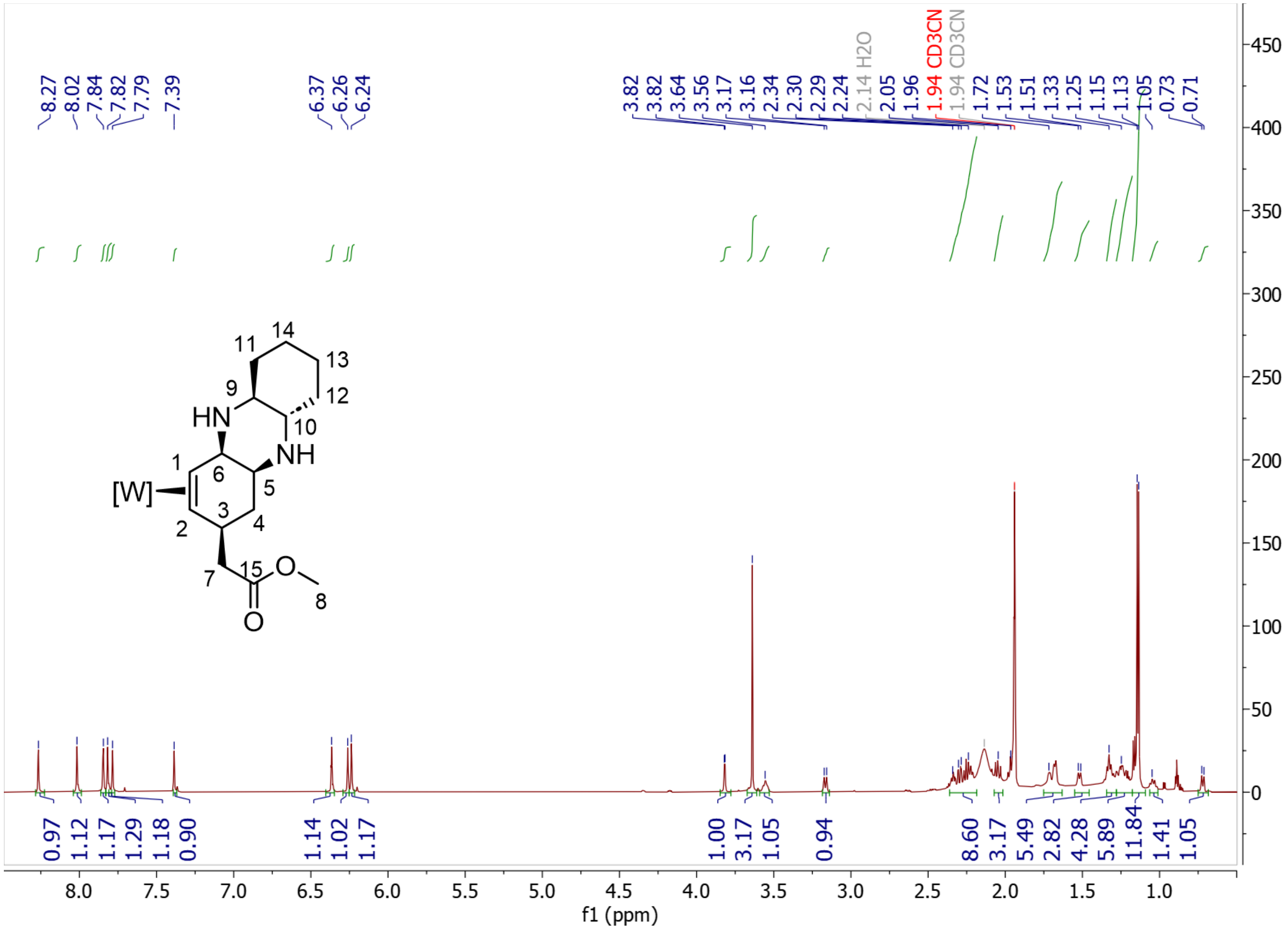

Supplementary Fig. 87: ^{1}H-NMR (CD_3CN) of Compound **41**.

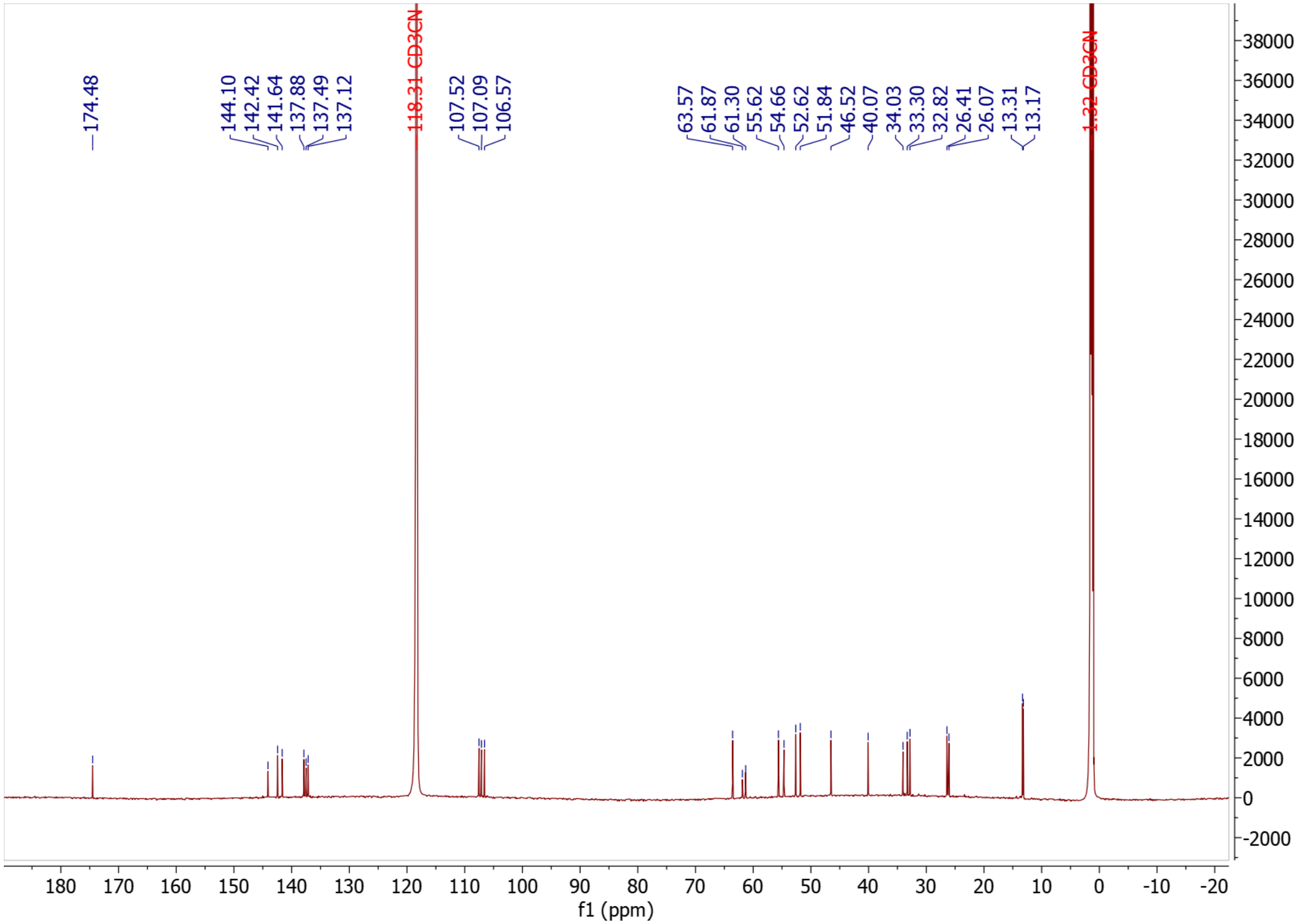

Supplementary Fig. 88: ^{13}C-NMR (CD_3CN) of Compound **41**.

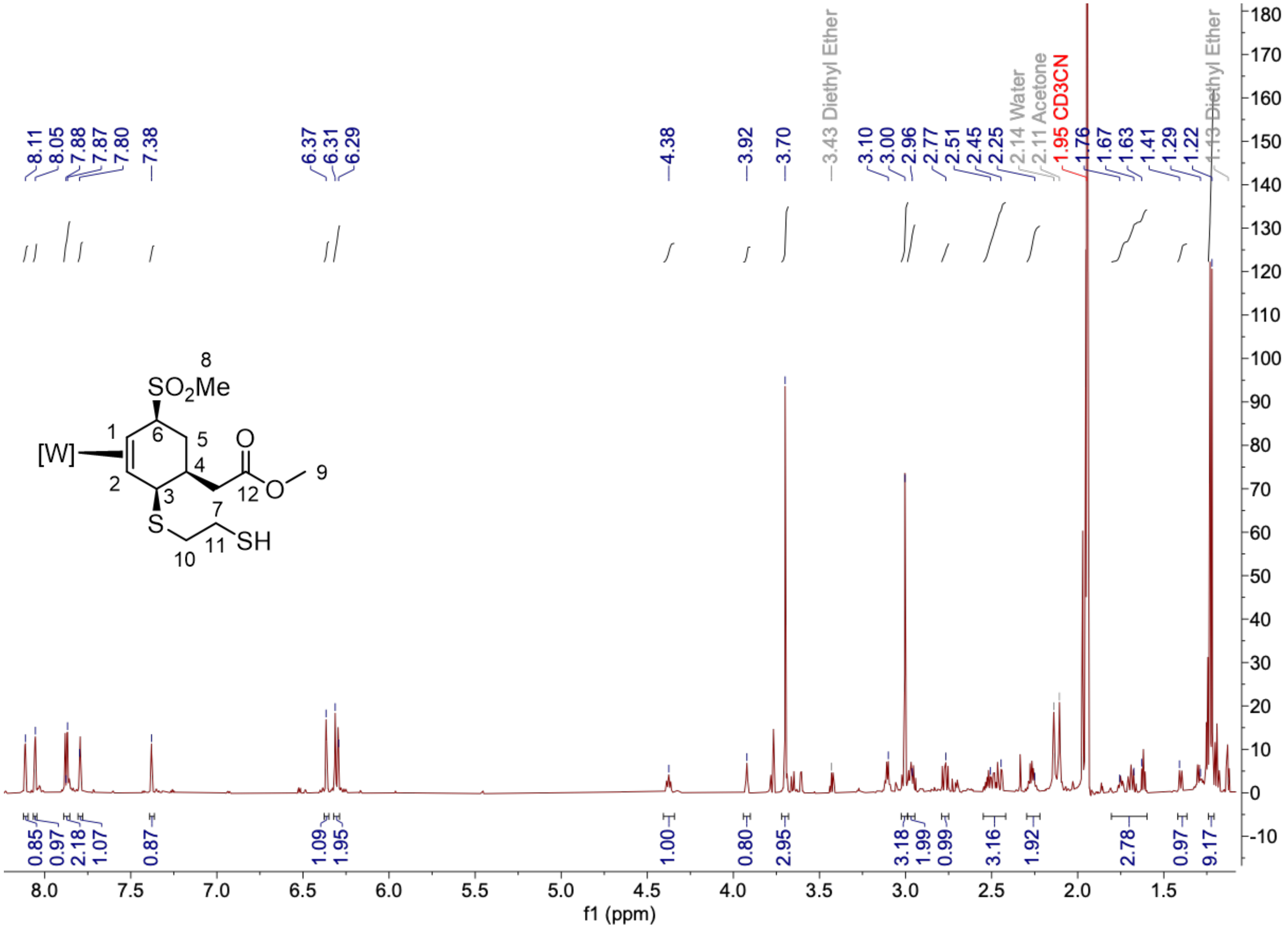

Supplementary Fig. 89: ^{1}H-NMR (CD_3CN) of Compound **42**.

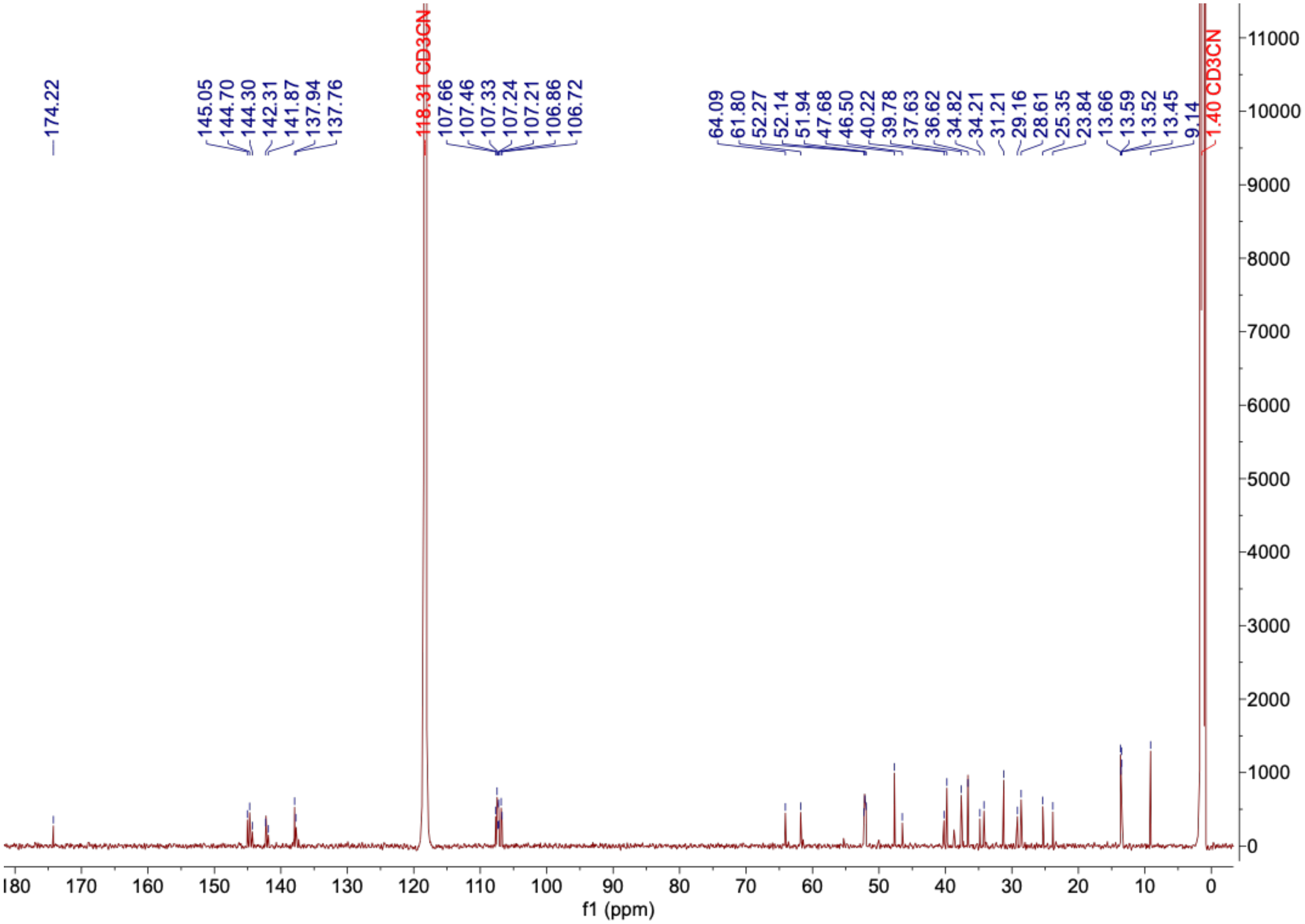

Supplementary Fig. 90: ^{13}C-NMR (CD_3CN) of Compound **42**.
Over the course of the NMR experiment 42 partially converted into **35**.

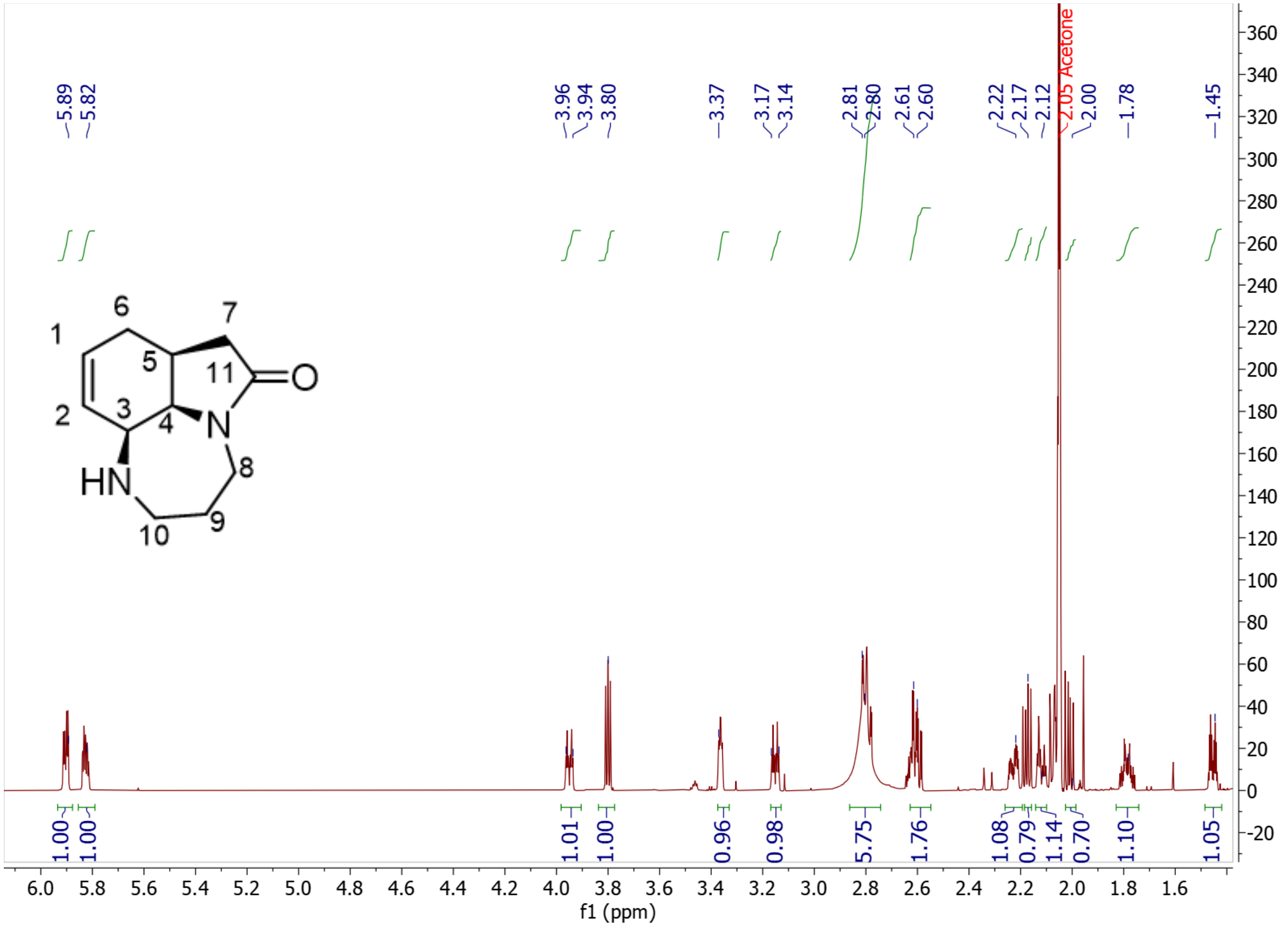

Supplementary Fig. 91: ^{1}H-NMR (CD_3CN) of Compound **43**.

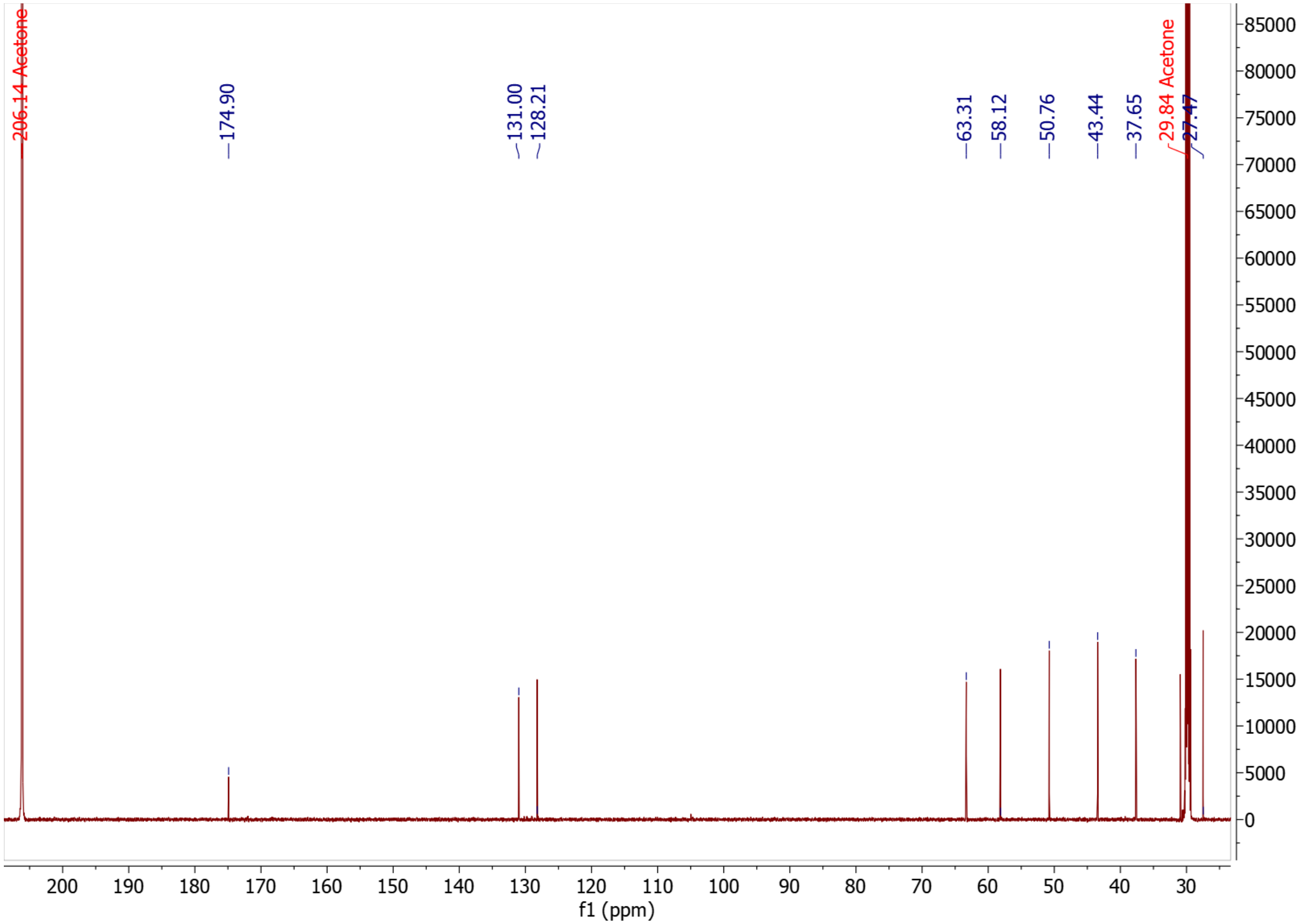

Supplementary Fig. 92: ^{13}C-NMR (CD_3CN) of Compound **43**.

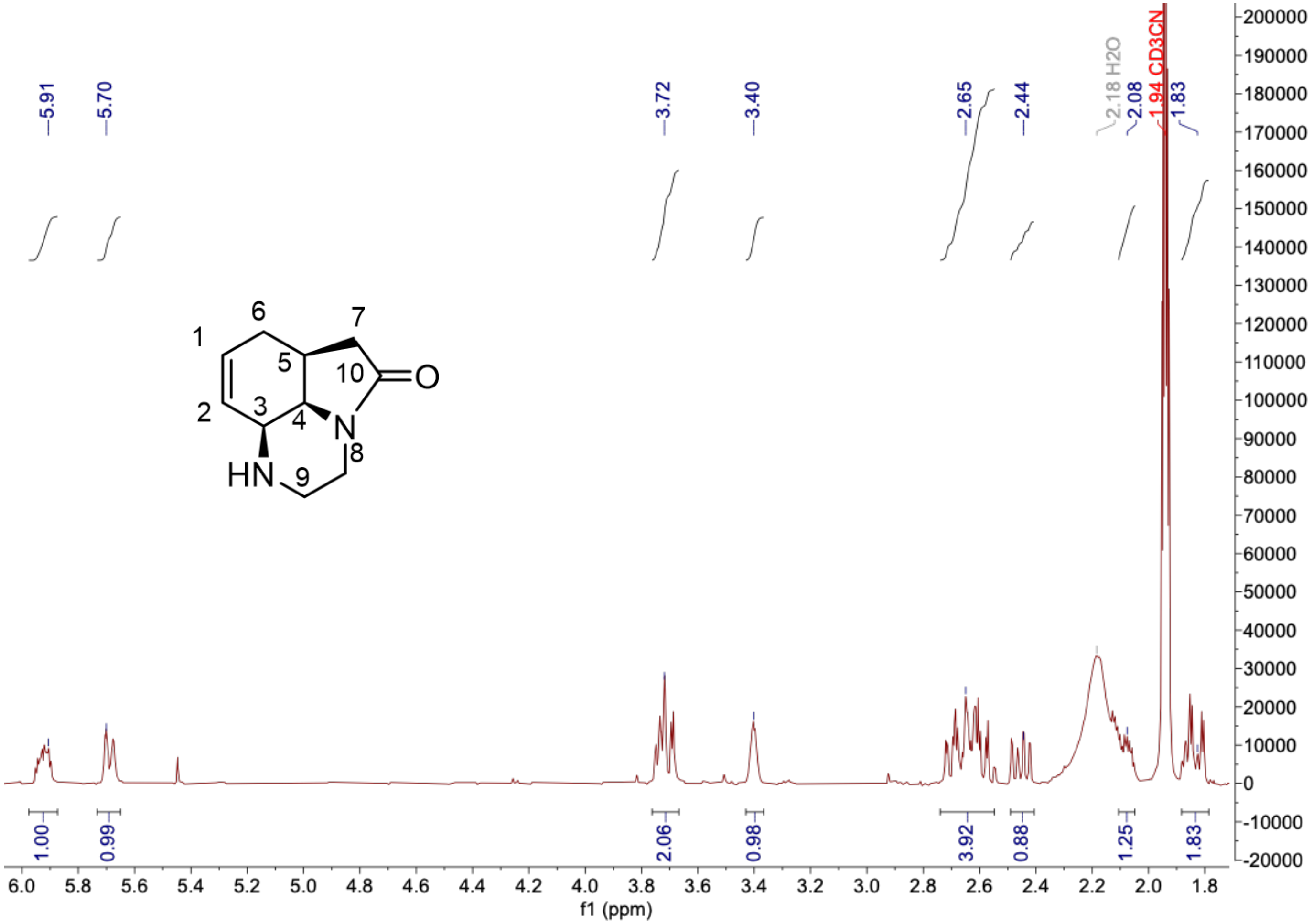

Supplementary Fig. 93: ^{1}H-NMR (CD_3CN) of Compound **44**.

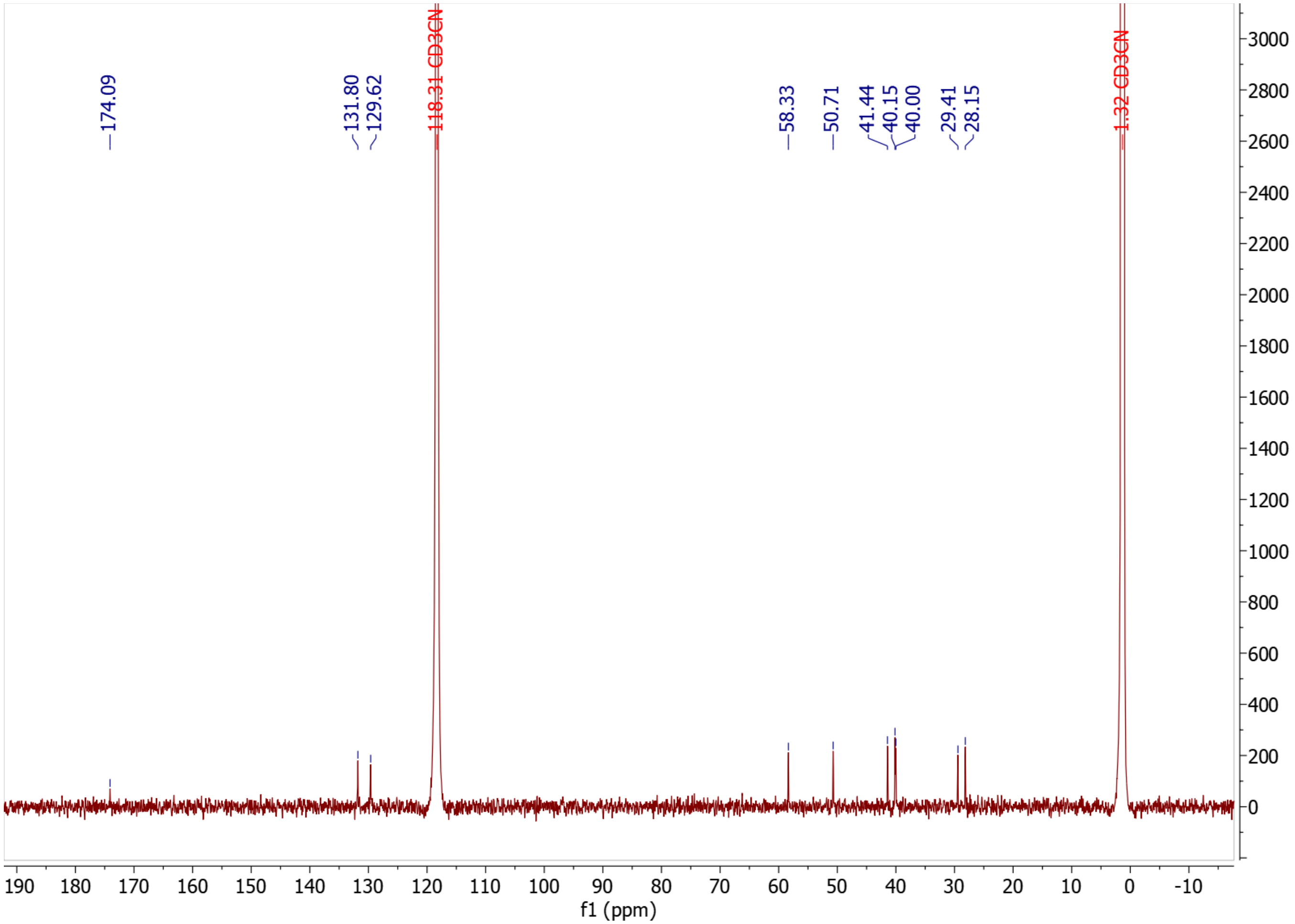

Supplementary Fig. 94: ^{13}C-NMR (CD_3CN) of Compound **44**.

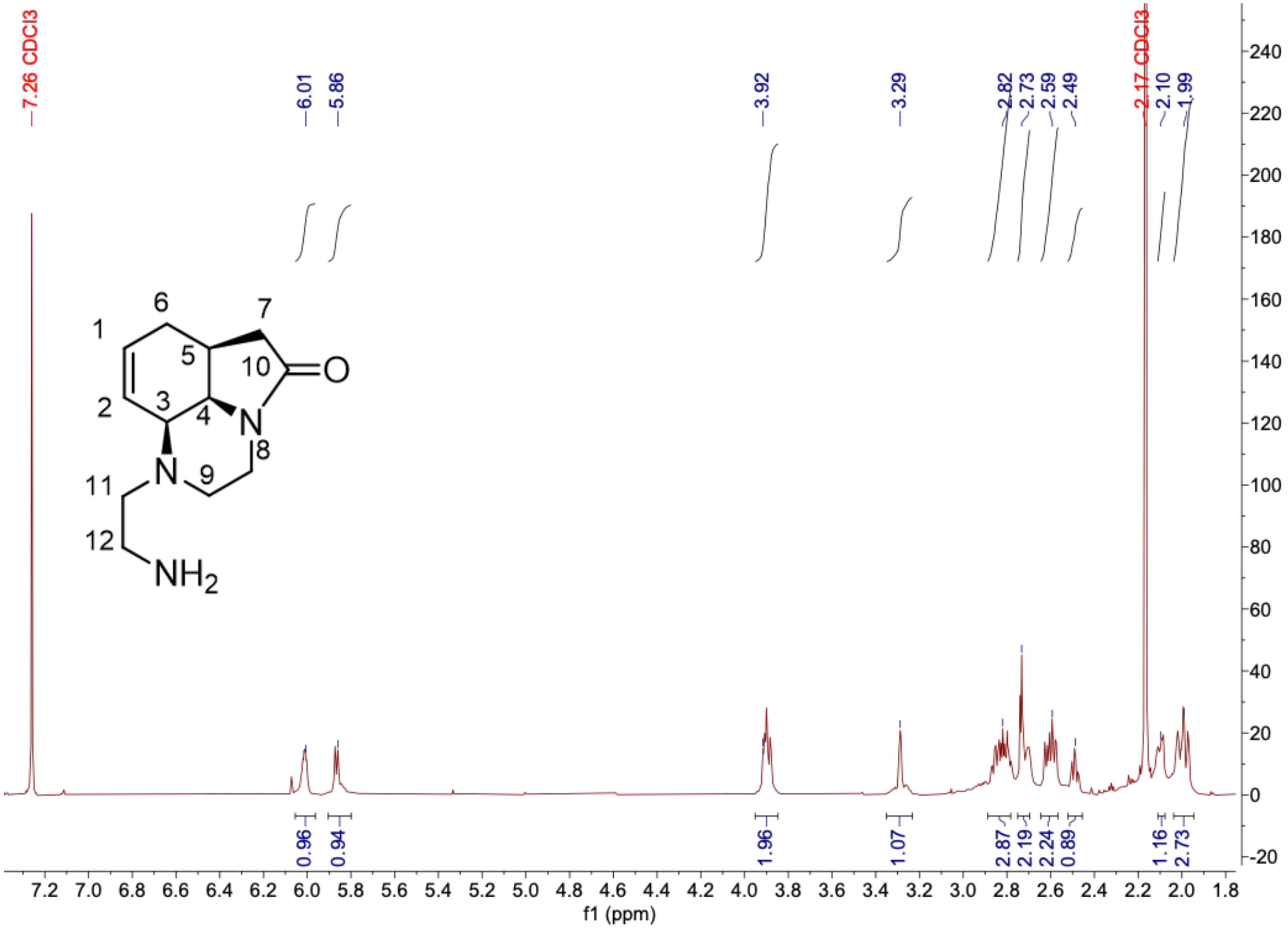

Supplementary Fig. 95: ^{1}H-NMR ($CDCl_3$) of Compound **45**.

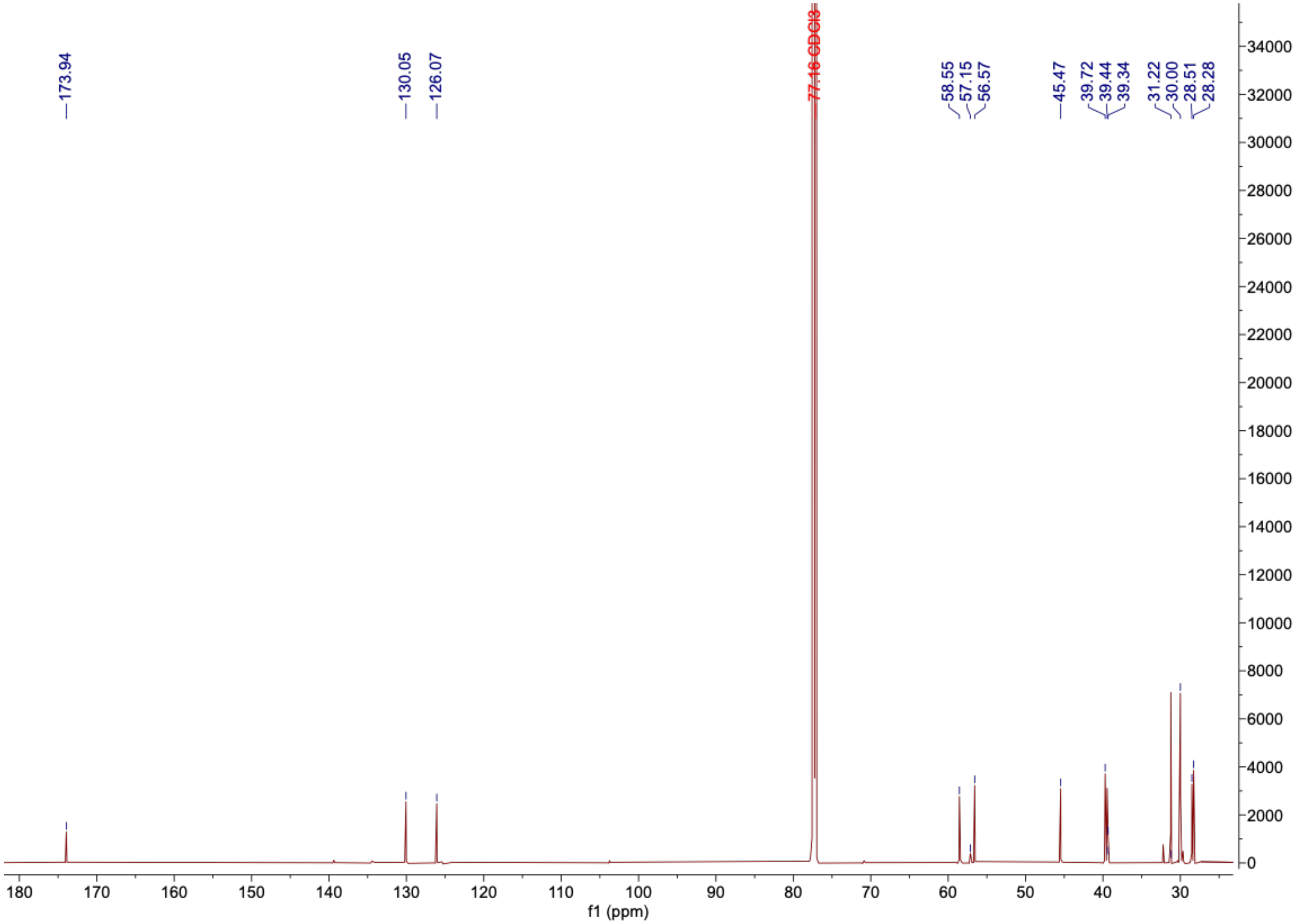

Supplementary Fig. 96: ^{13}C-NMR ($CDCl_3$) of Compound **45**.

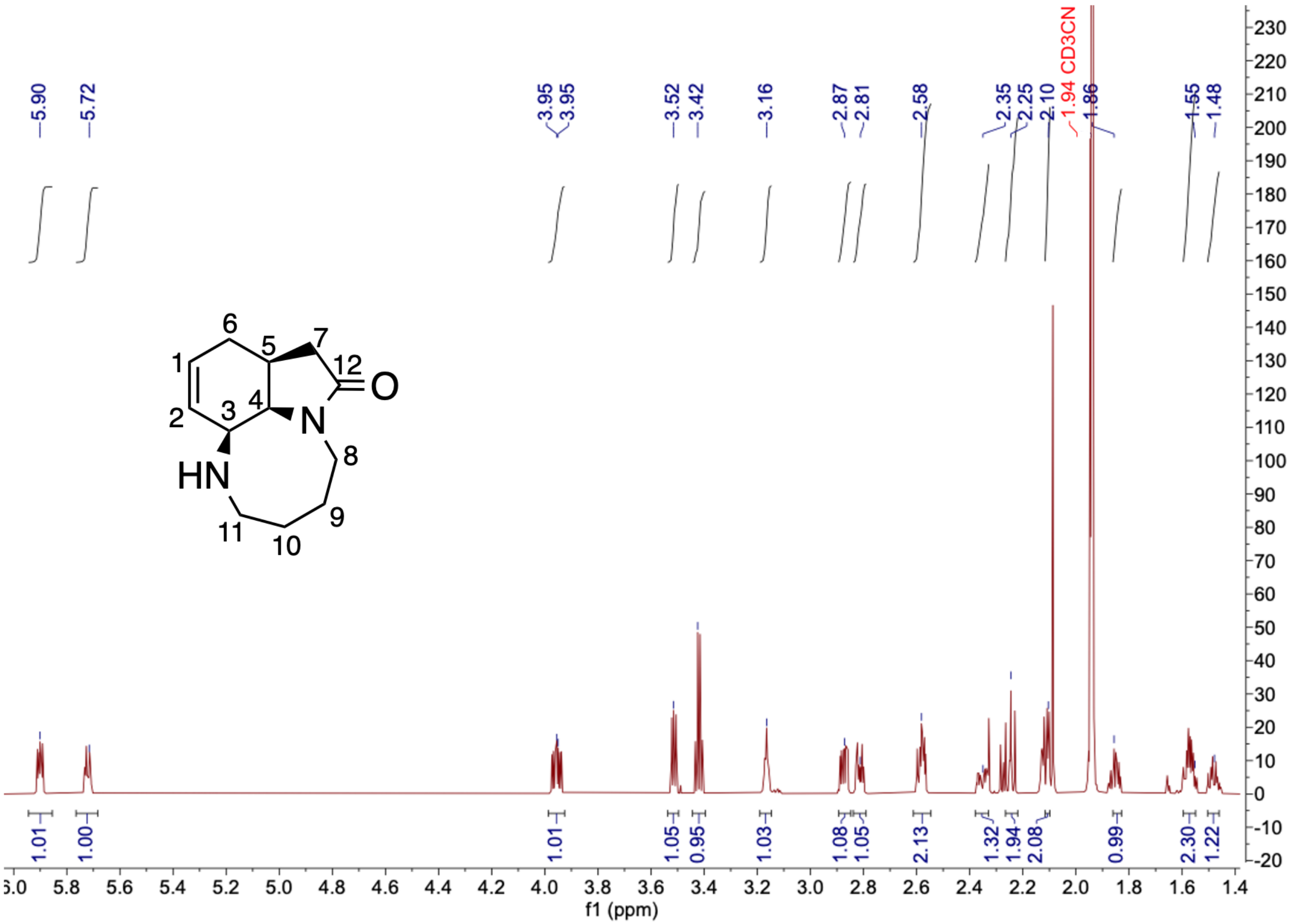

Supplementary Fig. 97: ^{1}H-NMR (CD_3CN) of Compound **46**.

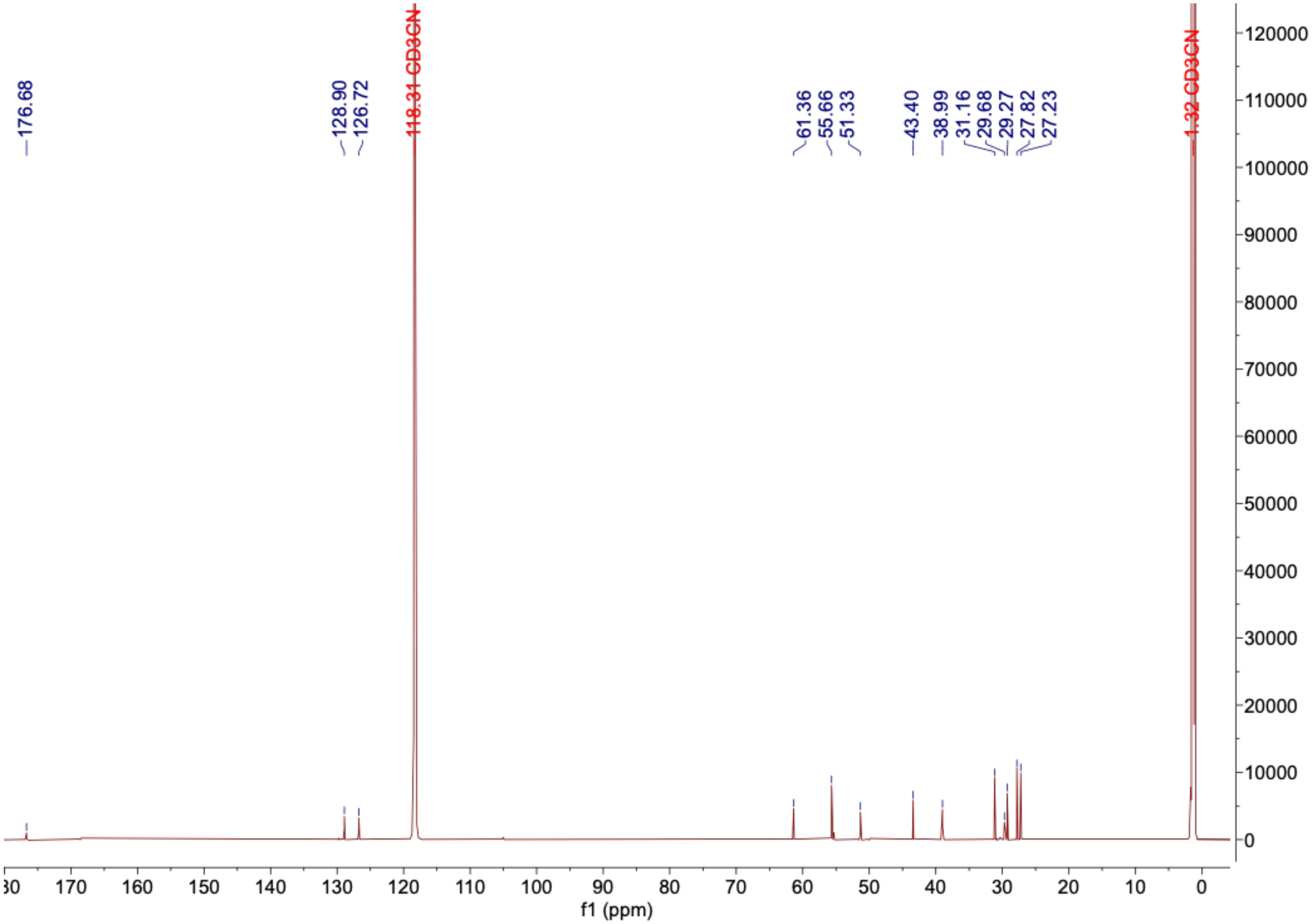

Supplementary Fig. 98: ^{13}C-NMR (CD_3CN) of Compound **46**.

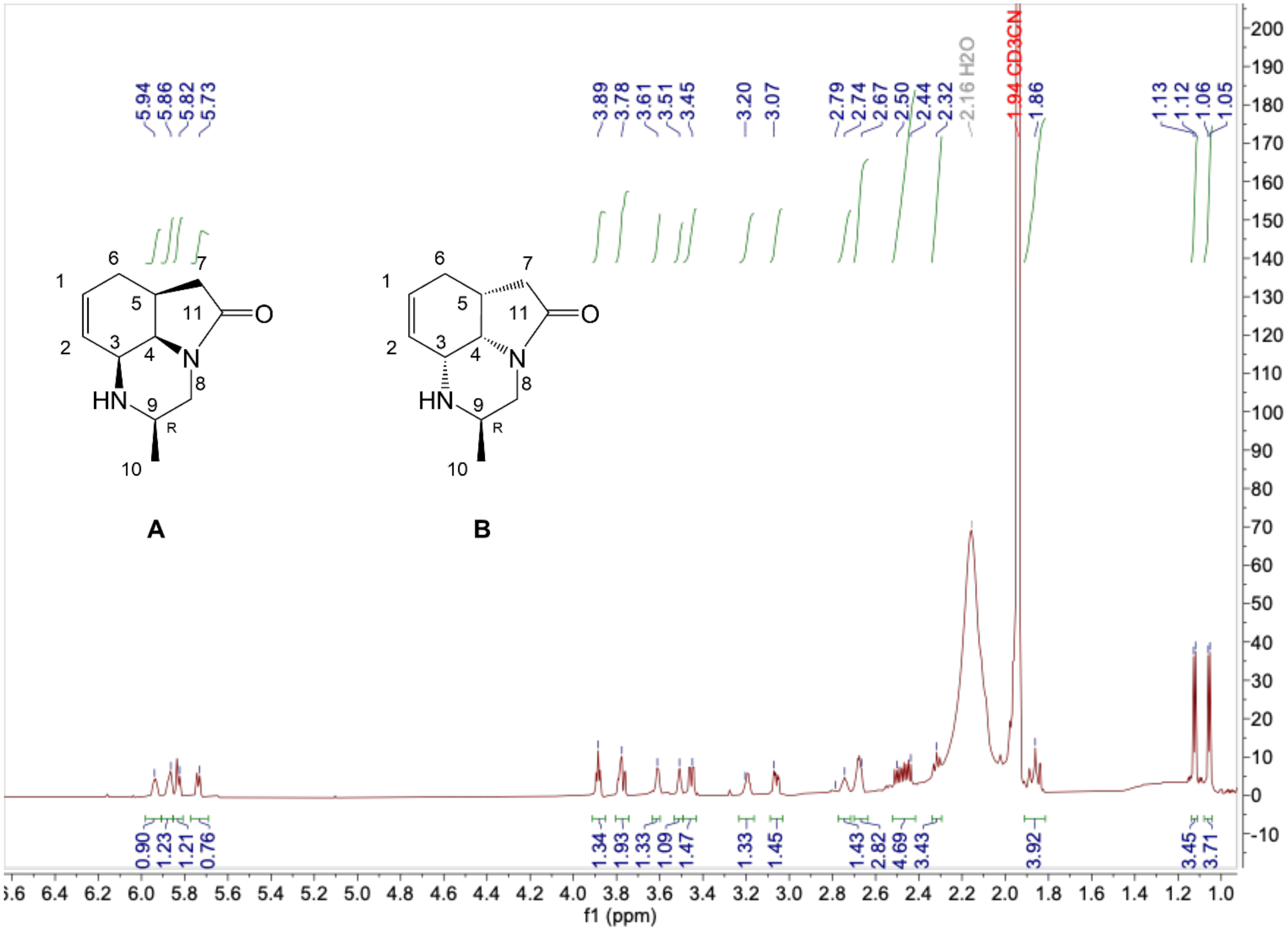

Supplementary Fig. 99: ^{1}H-NMR (CD_3CN) of Compound **47**.

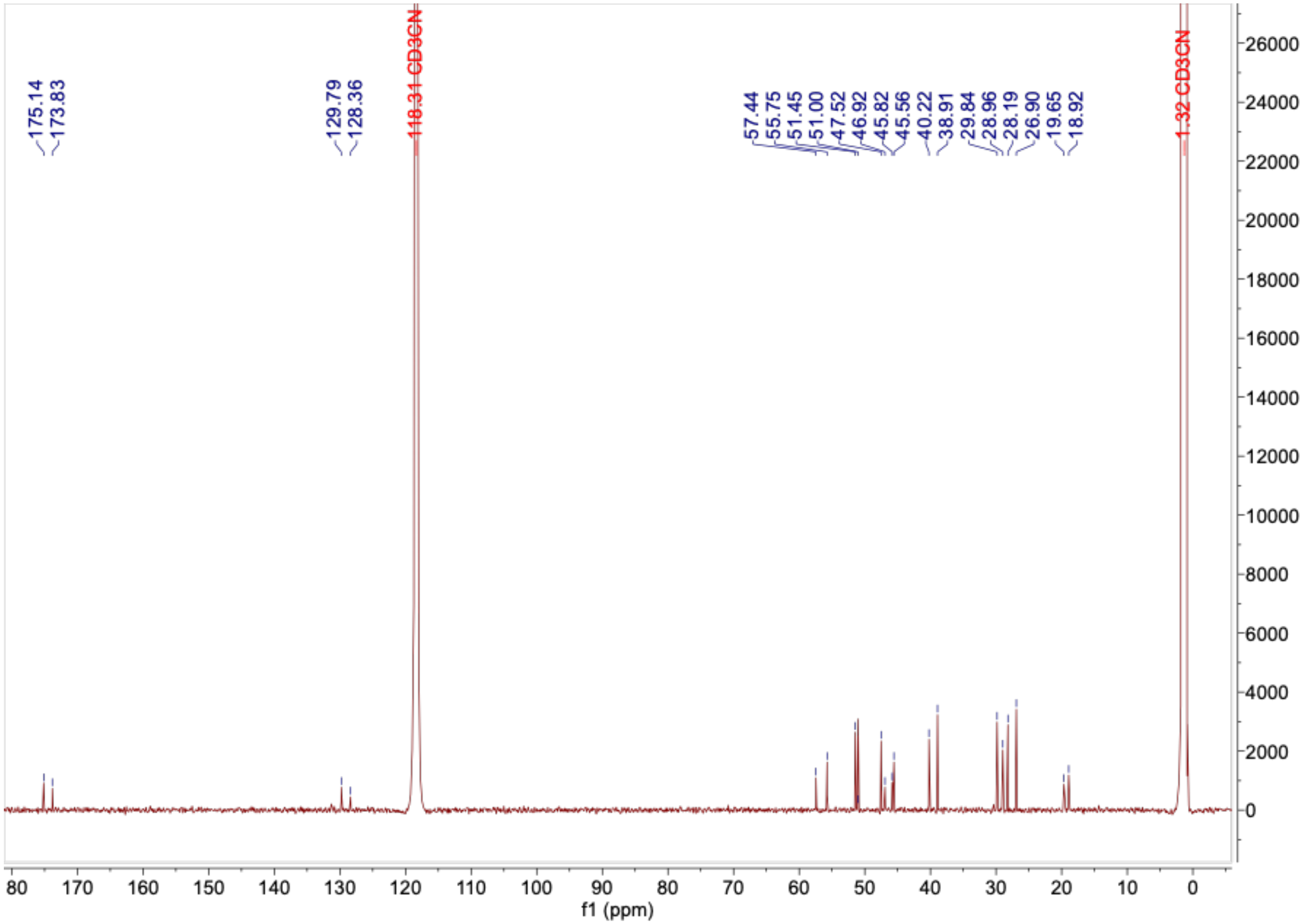

Supplementary Fig. 100: ^{13}C-NMR (CD_3CN) of Compound **47**.

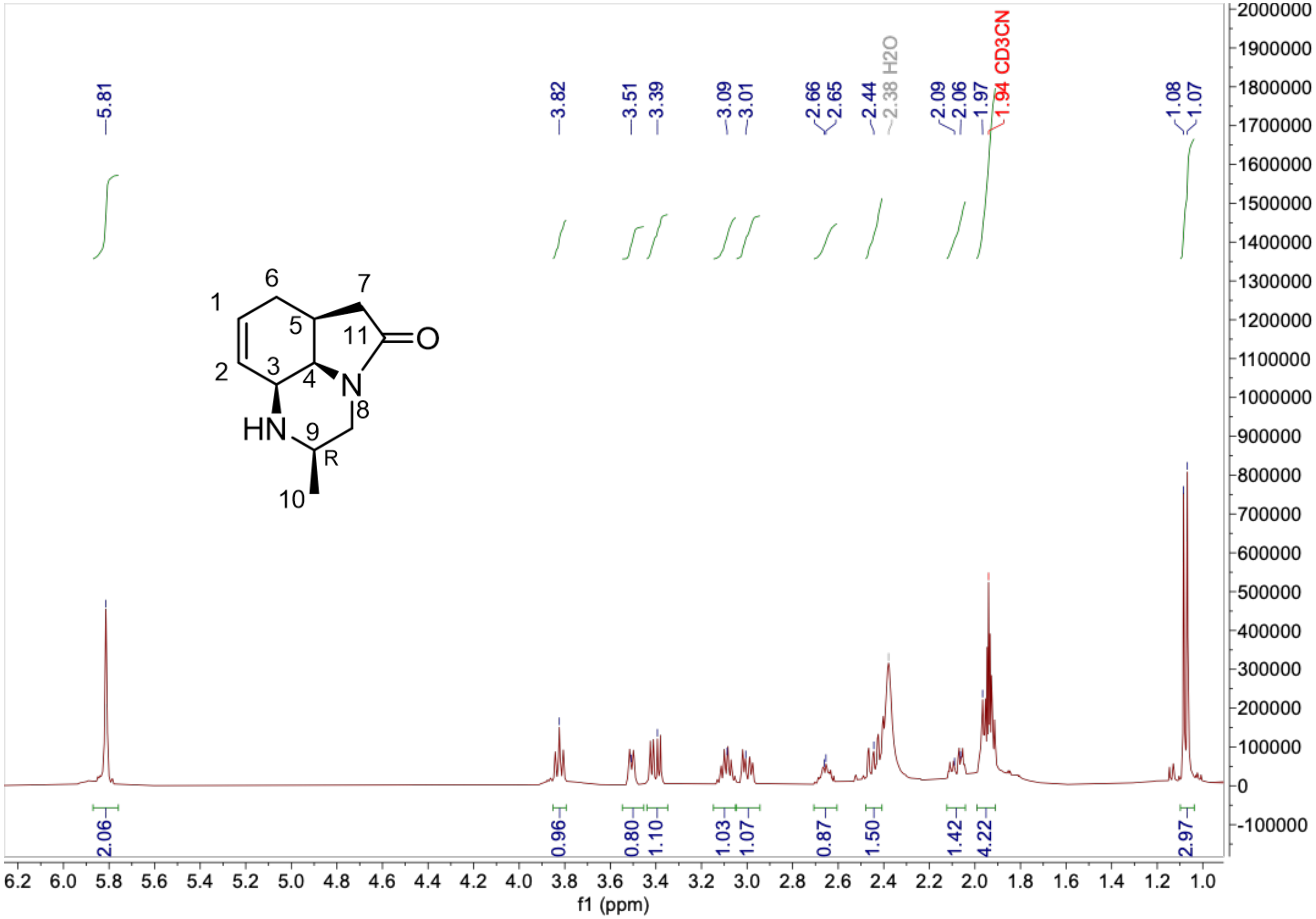

Supplementary Fig. 101: ^{1}H-NMR (CD_3CN) of Compound **48**.

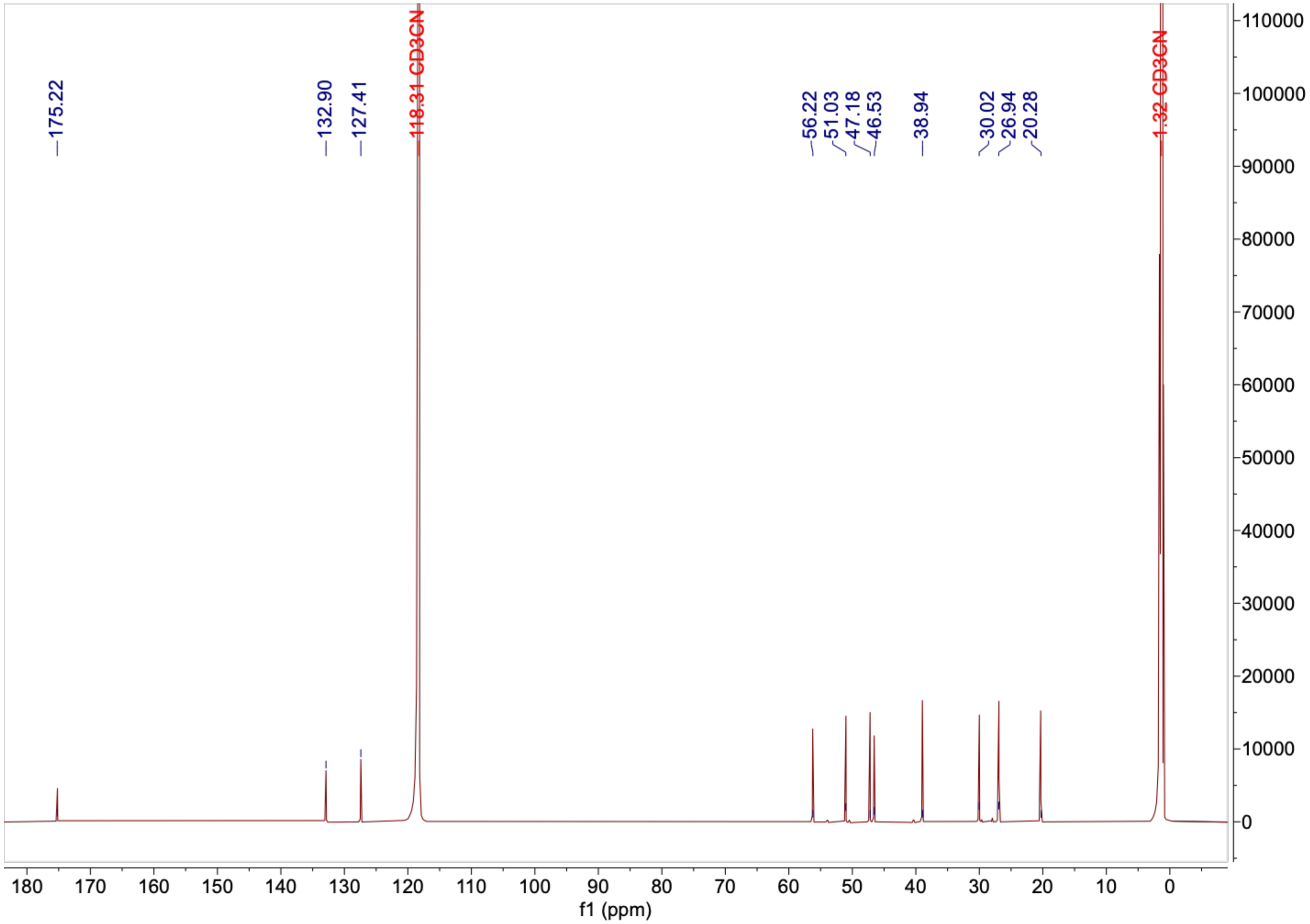

Supplementary Fig. 102: ^{13}C-NMR (CD_3CN) of Compound **48**.

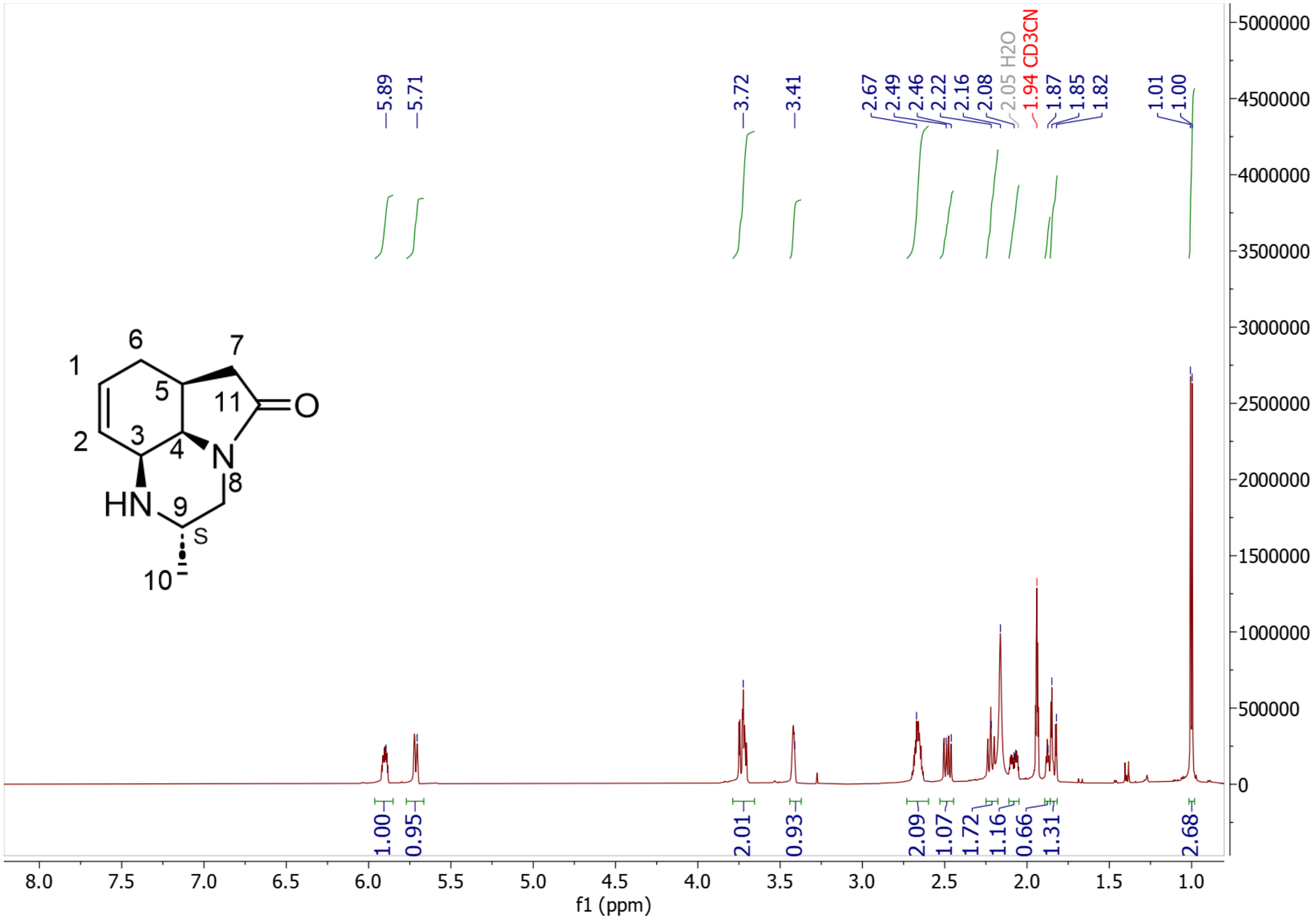

Supplementary Fig. 103: ^{1}H-NMR (CD_3CN) of Compound **49**.

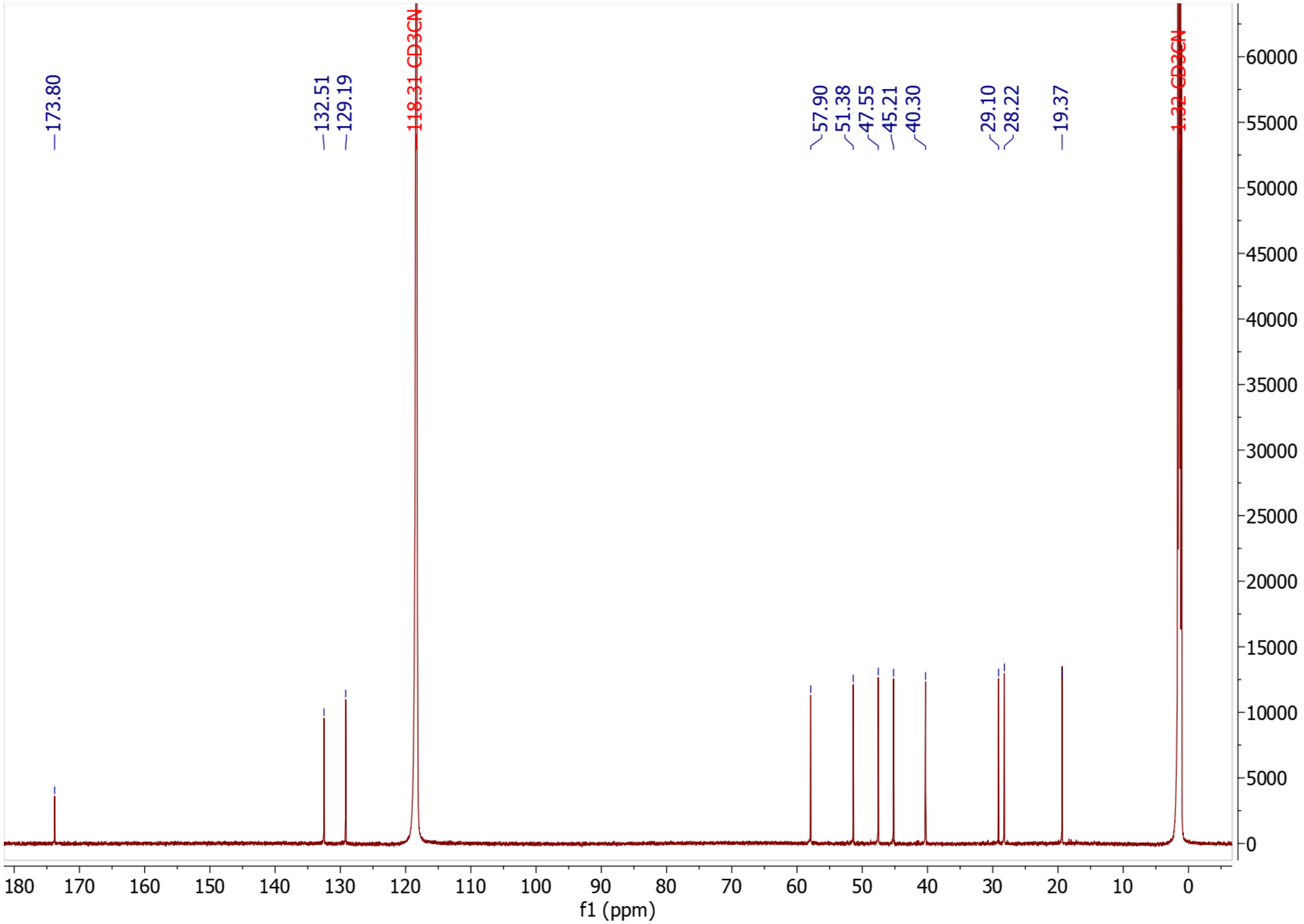

Supplementary Fig. 104: ^{13}C-NMR (CD_3CN) of Compound **49**.

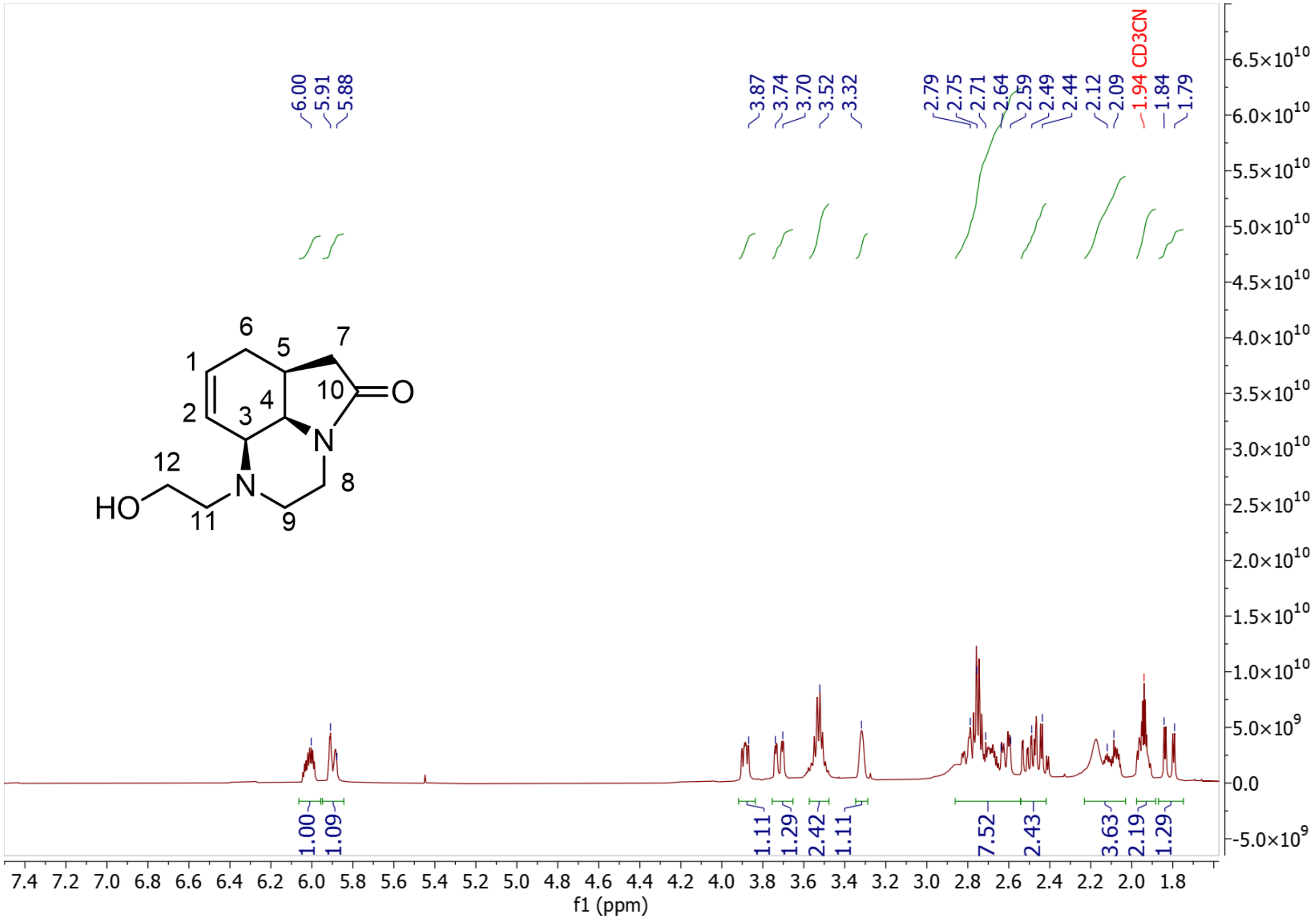

Supplementary Fig. 105: ^{1}H-NMR (CD_3CN) of Compound **50**.

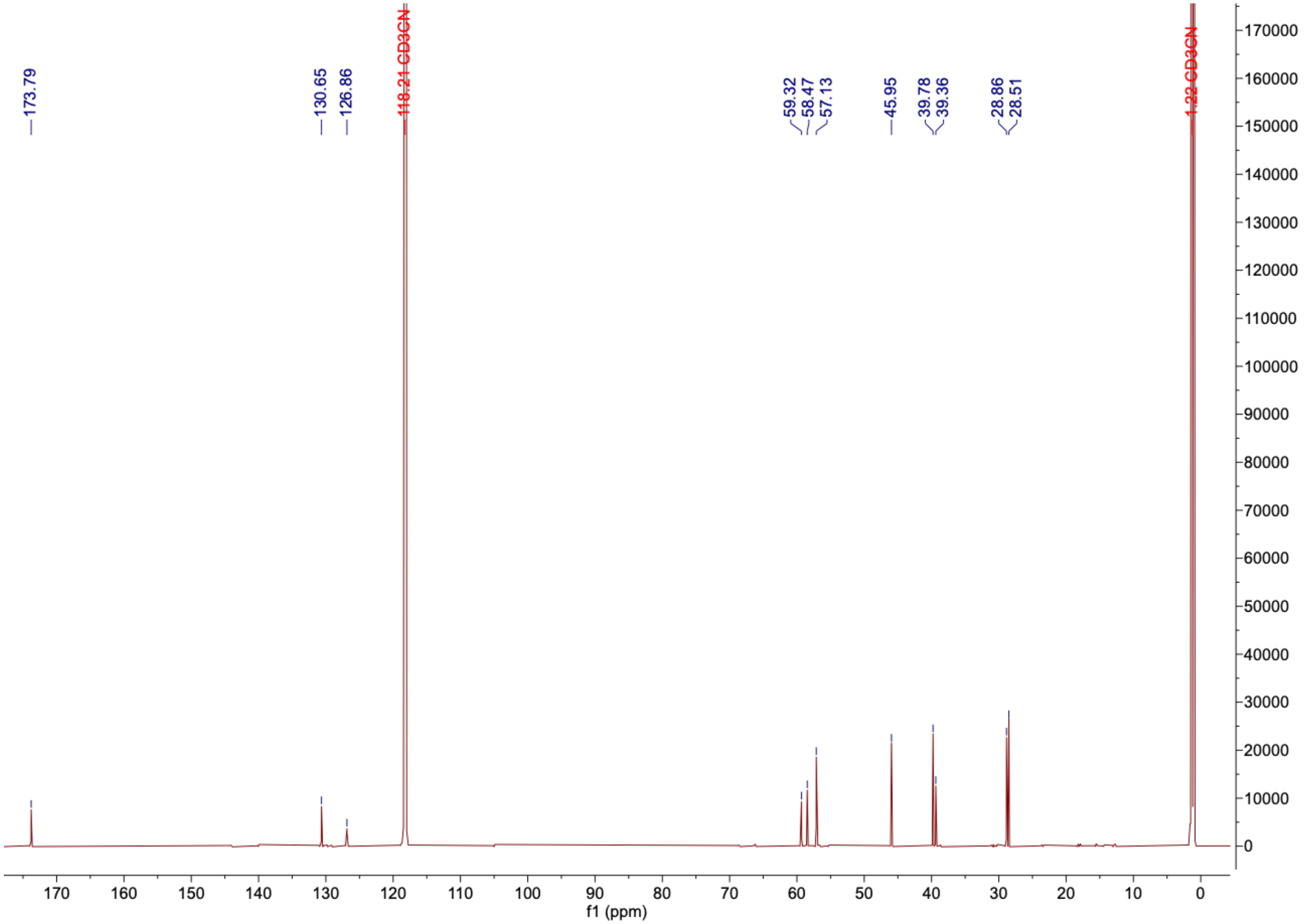

Supplementary Fig. 106: ^{13}C-NMR (CD_3CN) of Compound **50**.

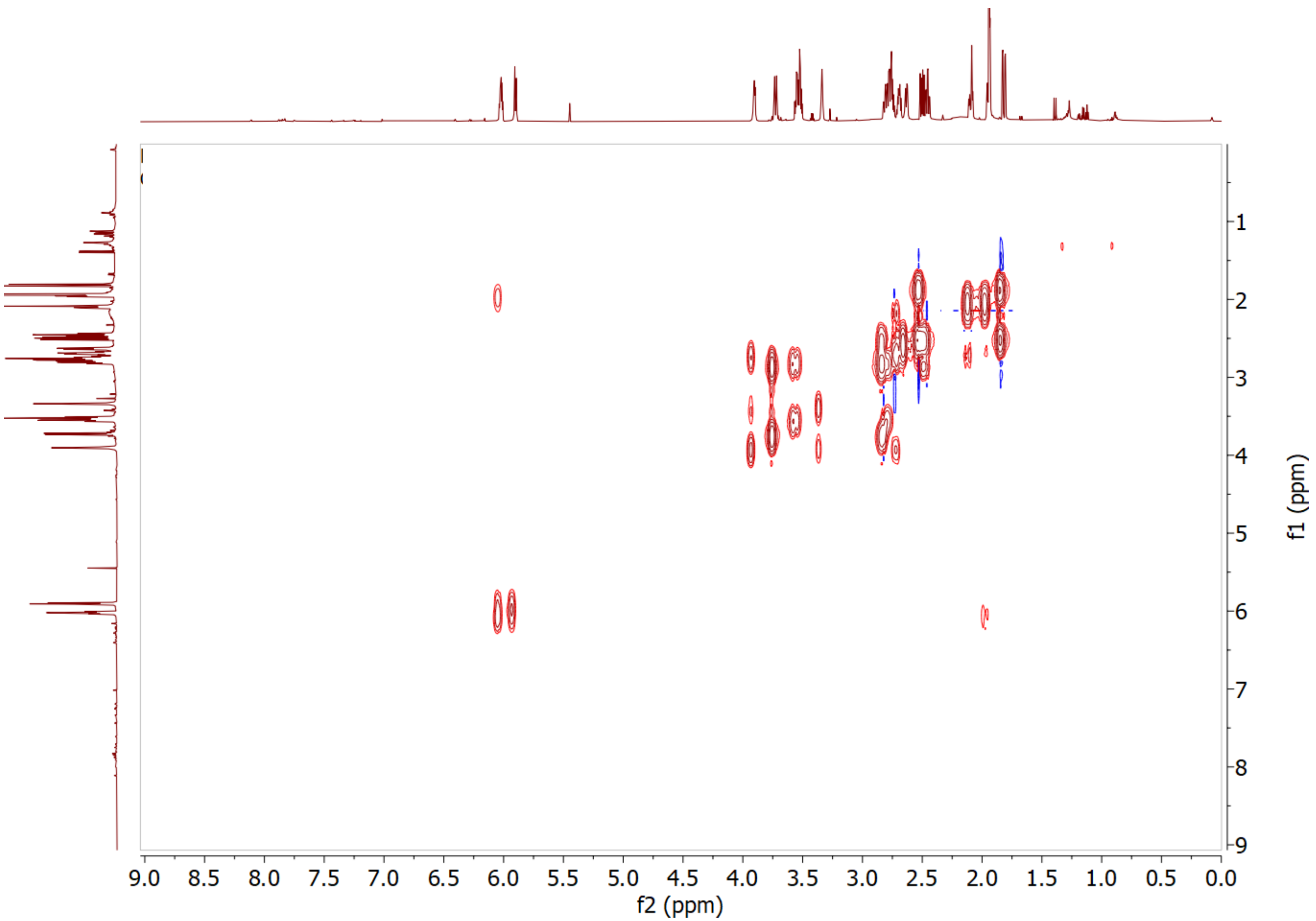

Supplementary Fig. 107: ^1H-^1H COSY (CD_3CN) of Compound **50**.

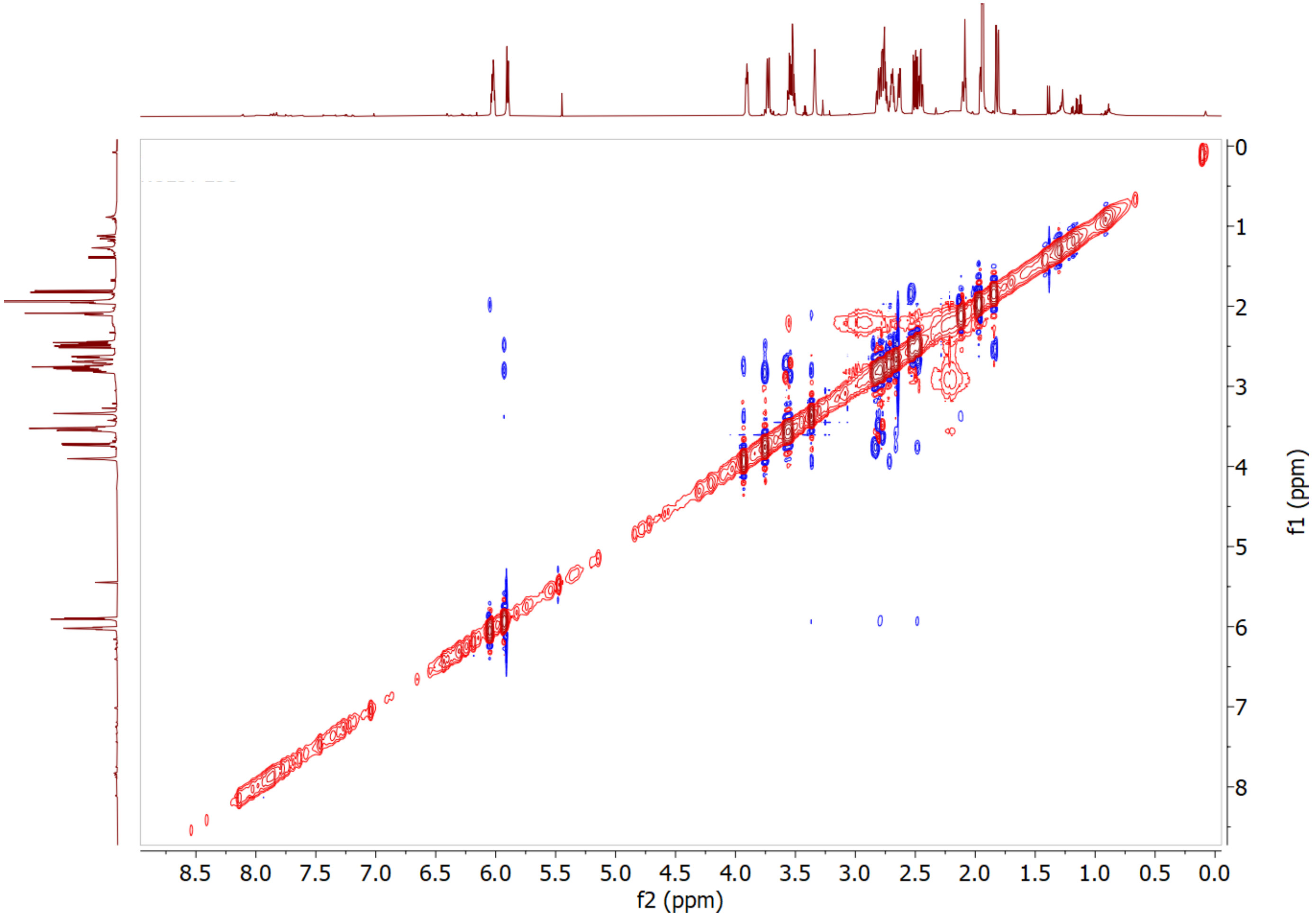

Supplementary Fig. 108: ^{1}H-^{1}H NOESY (CD_3CN) of Compound **50**.

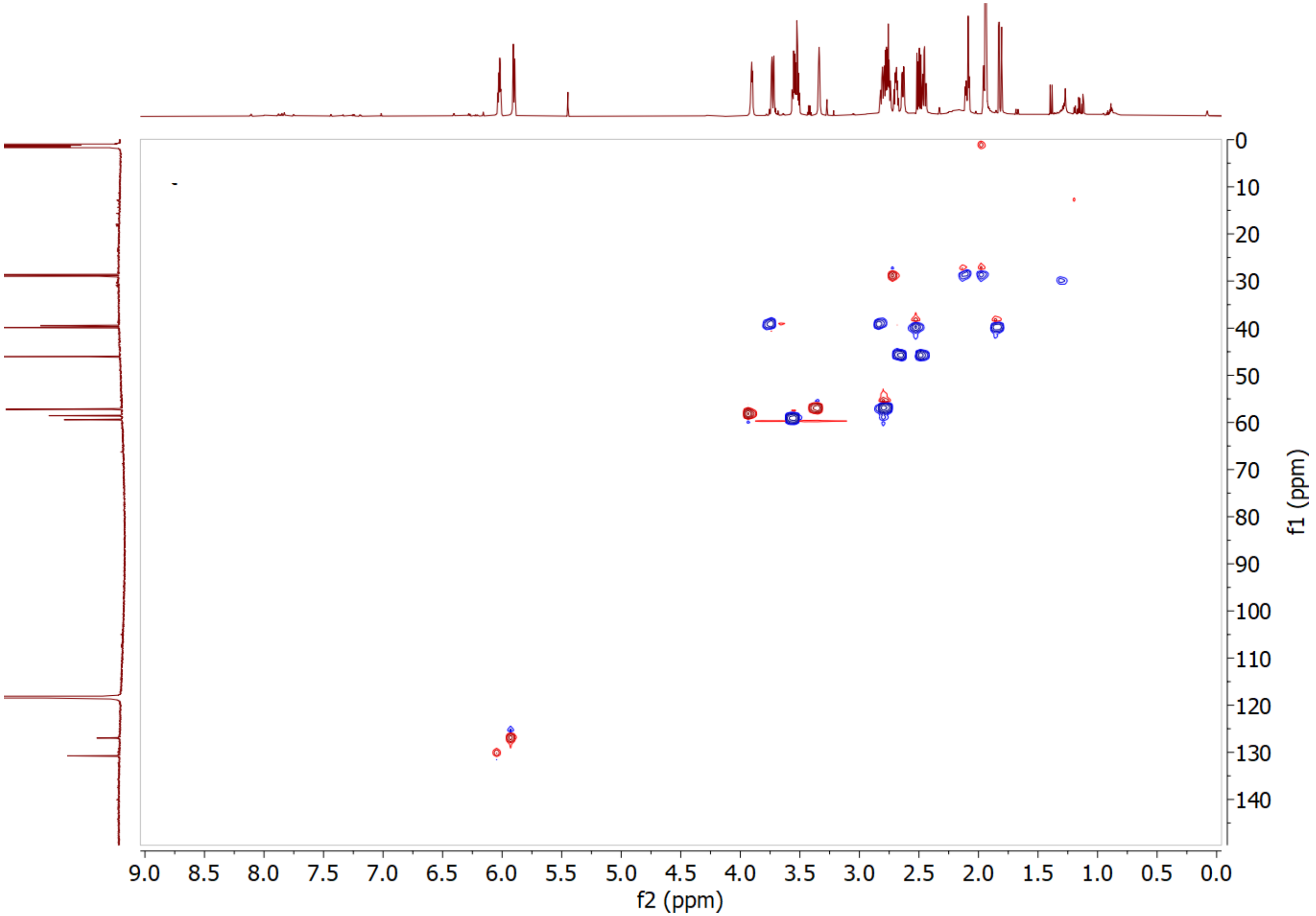

Supplementary Fig. 109: ^{1}H-^{13}C HSQC (CD_3CN) of Compound **50**.

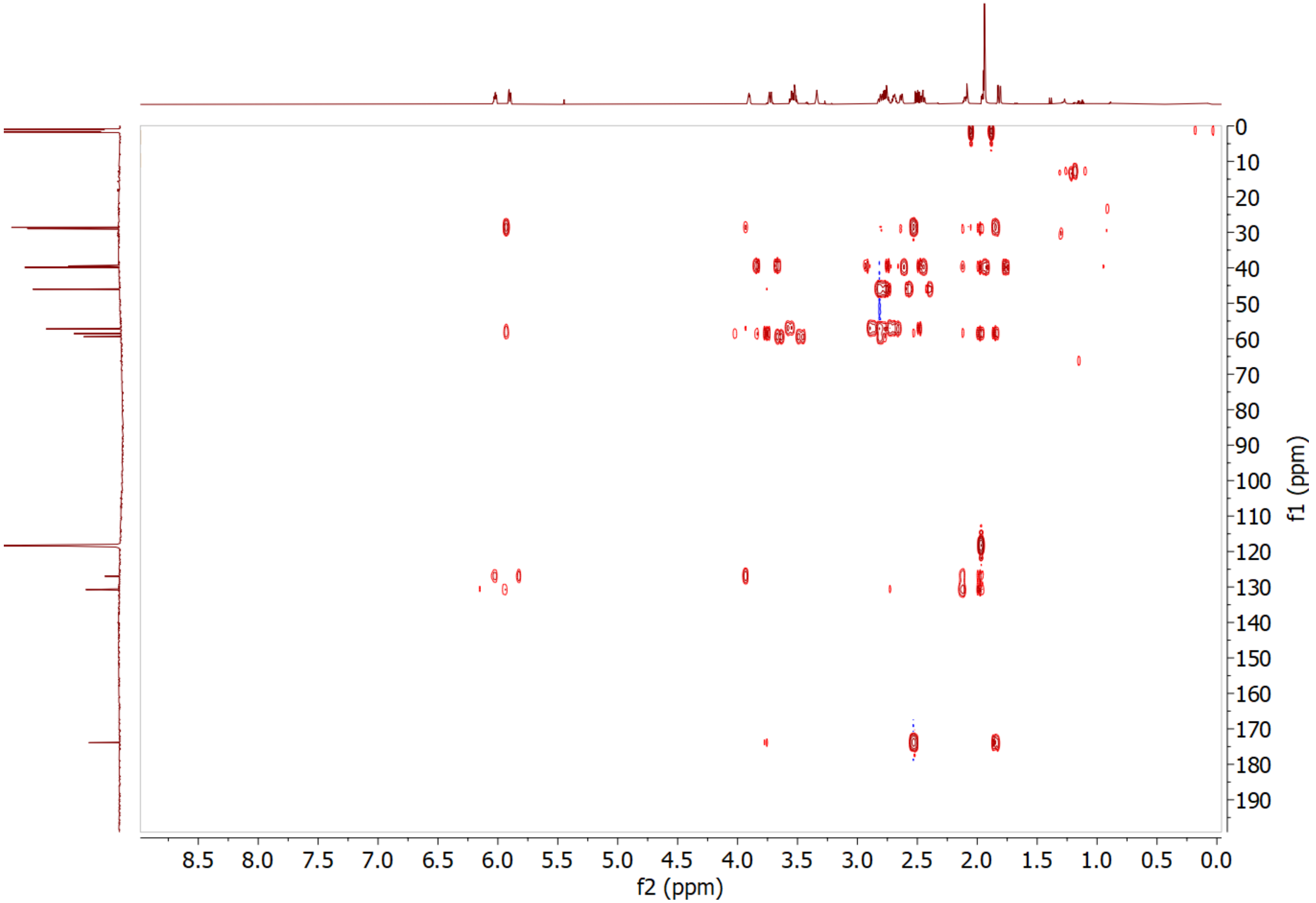

Supplementary Fig. 110: ^{1}H-^{13}C HMBC (CD_3CN) of Compound **50**.

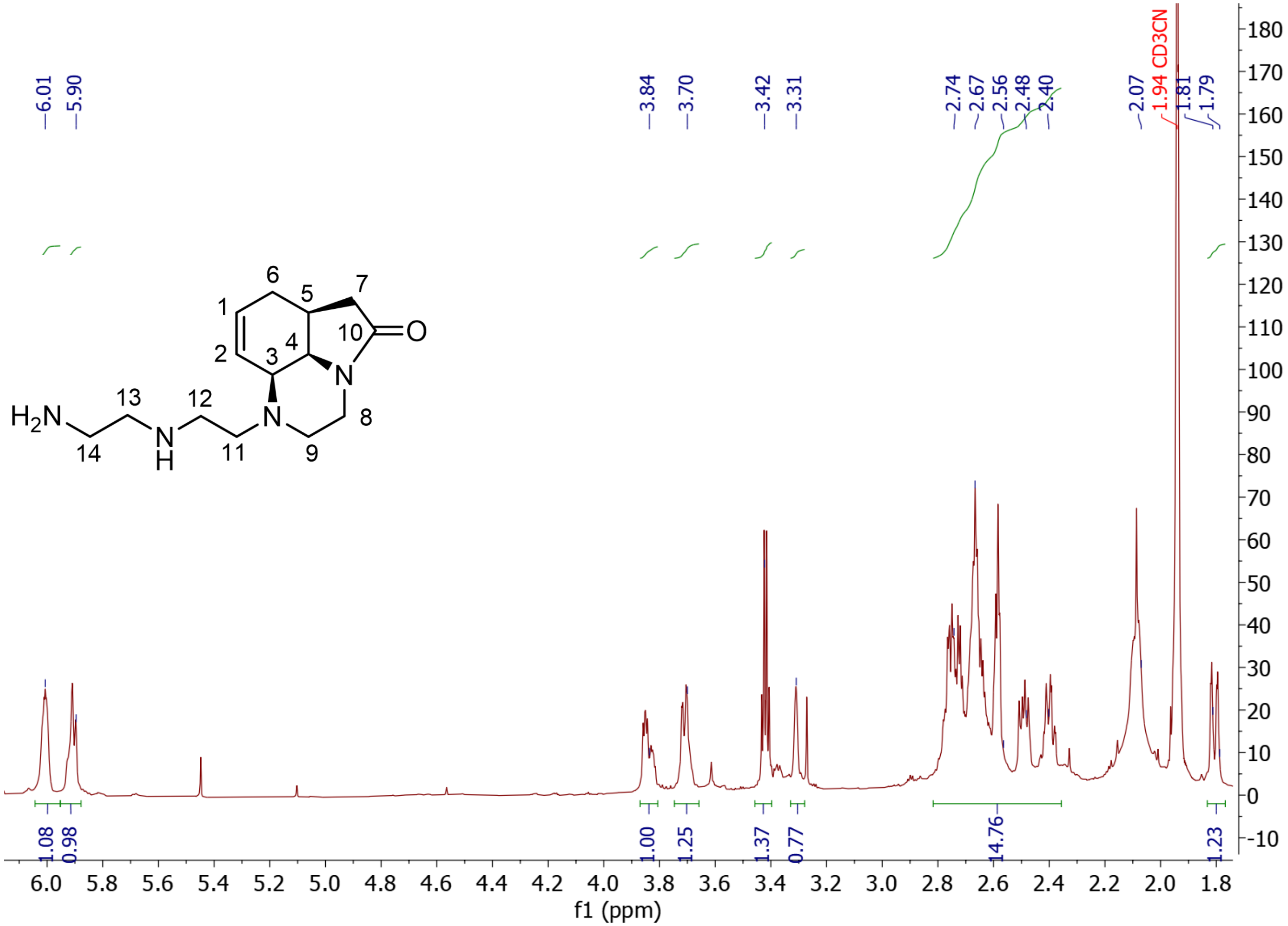

Supplementary Fig. 111: ^{1}H-NMR (CD_3CN) of Compound **51**.

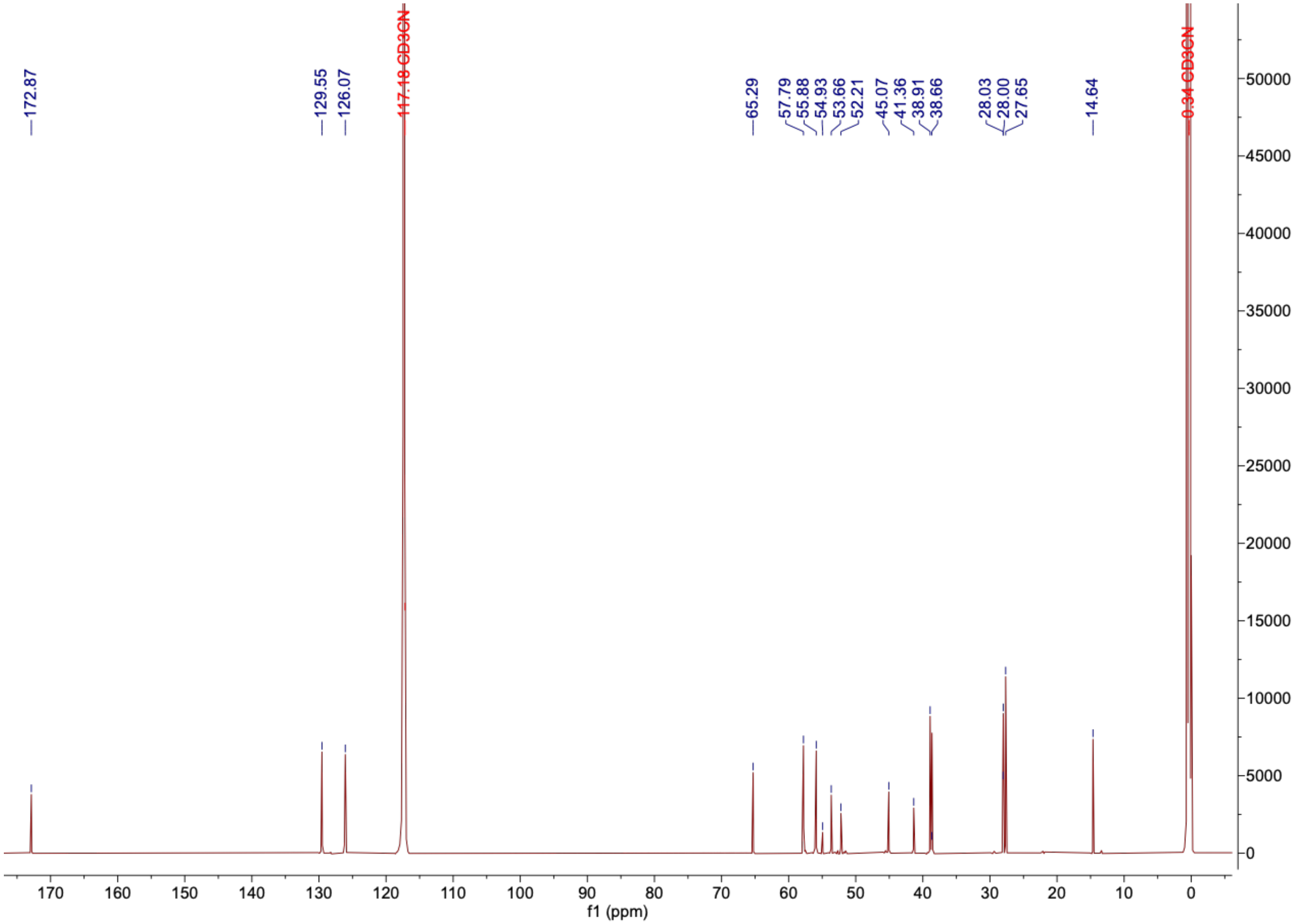

Supplementary Fig. 112: ^{13}C-NMR (CD_3CN) of Compound **51**.

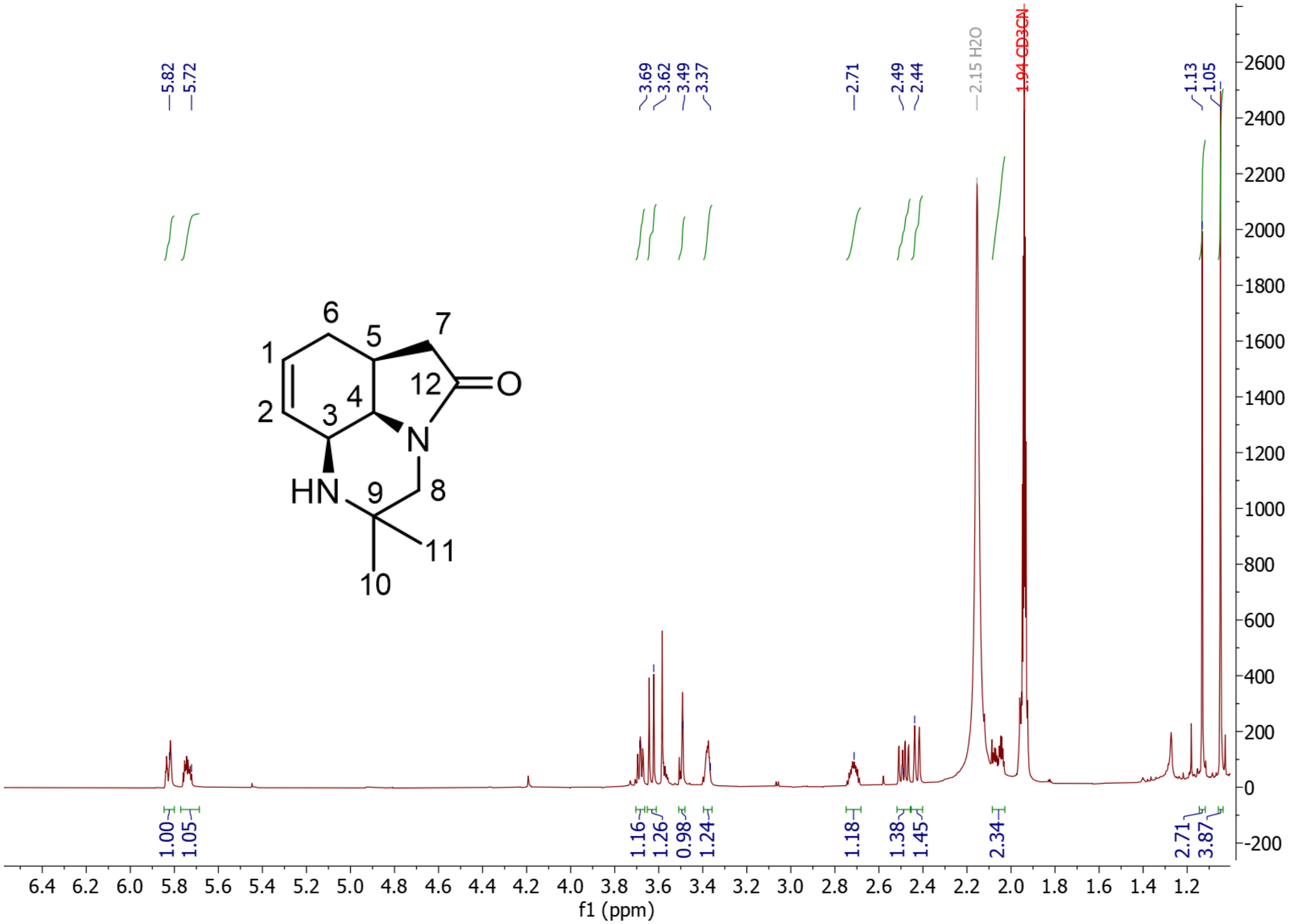

Supplementary Fig. 113: ^{1}H-NMR (CD_3CN) of Compound **52**.

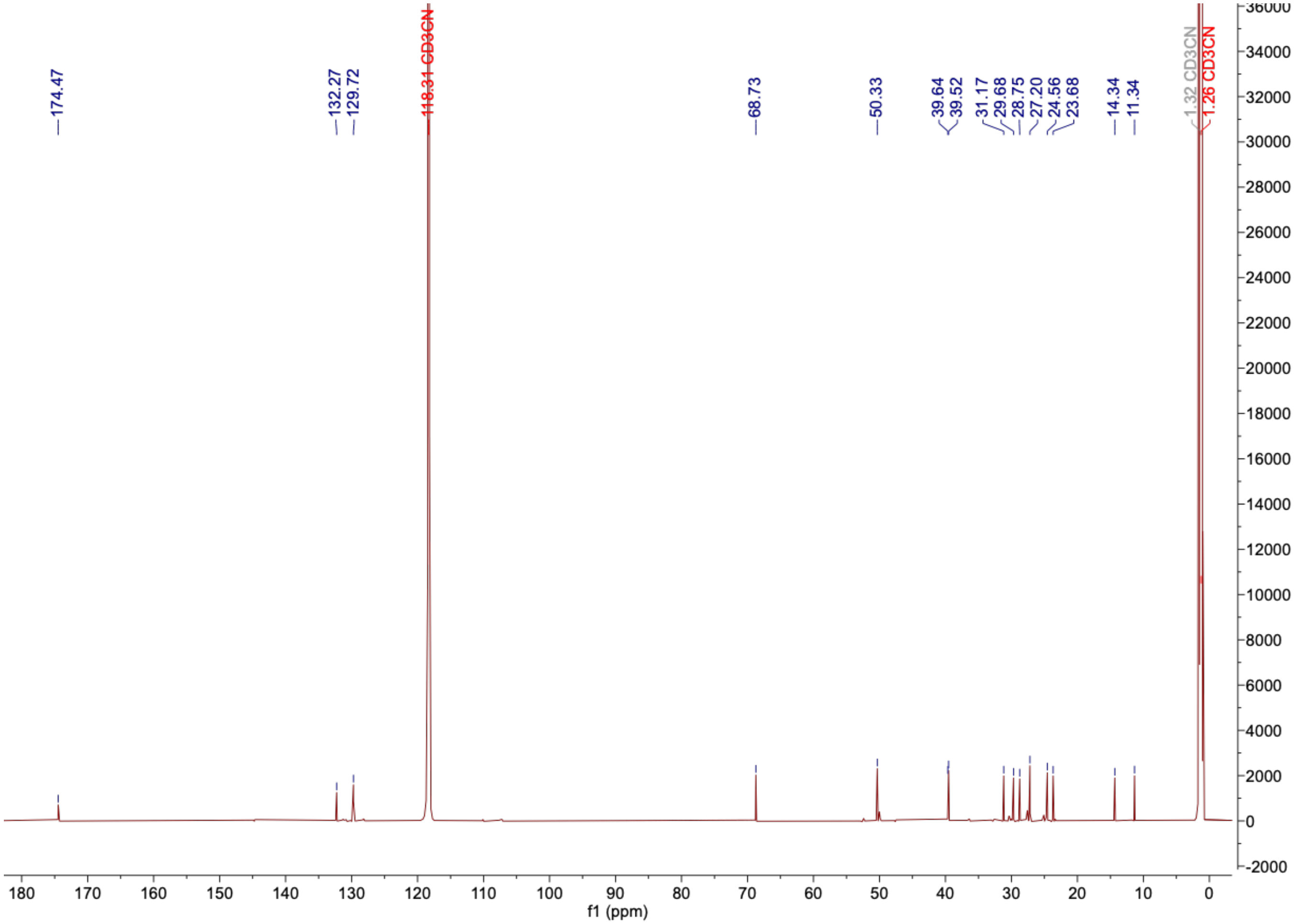

Supplementary Fig. 114: ^{13}C-NMR (CD_3CN) of Compound **52**.

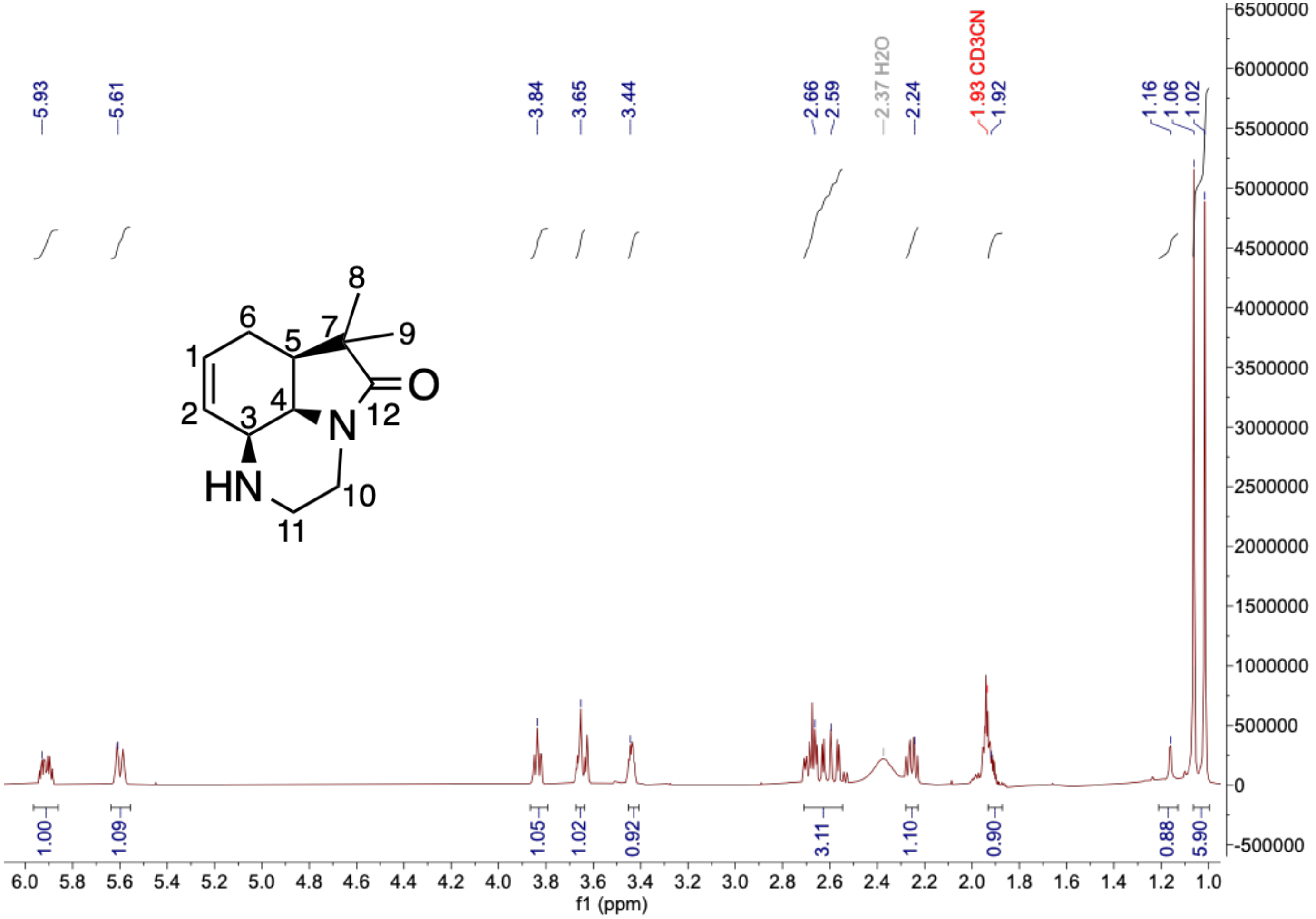

Supplementary Fig. 115: ^{1}H-NMR (CD_3CN) of Compound **53**.

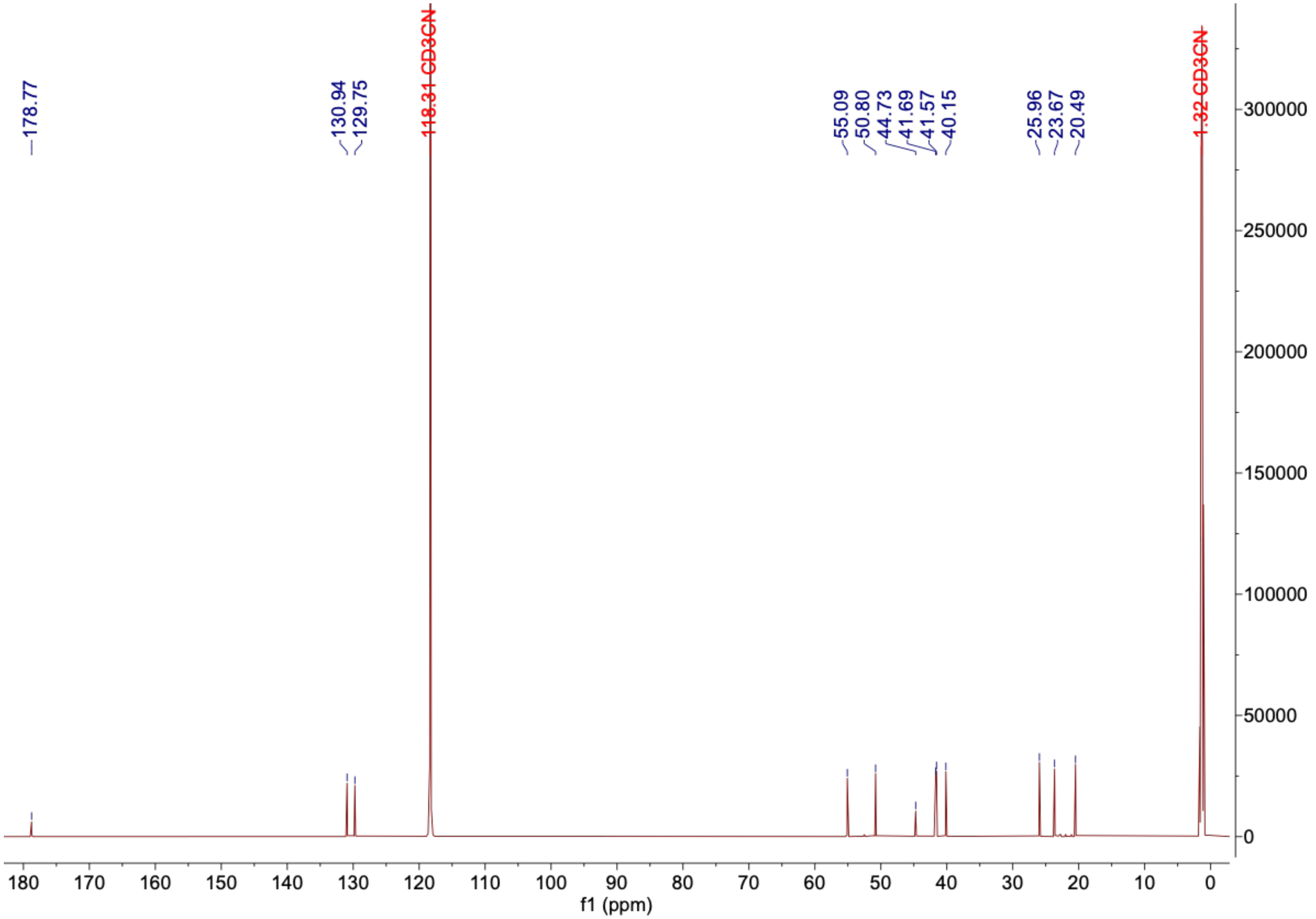

Supplementary Fig. 116: ^{13}C-NMR (CD_3CN) of Compound **53**.

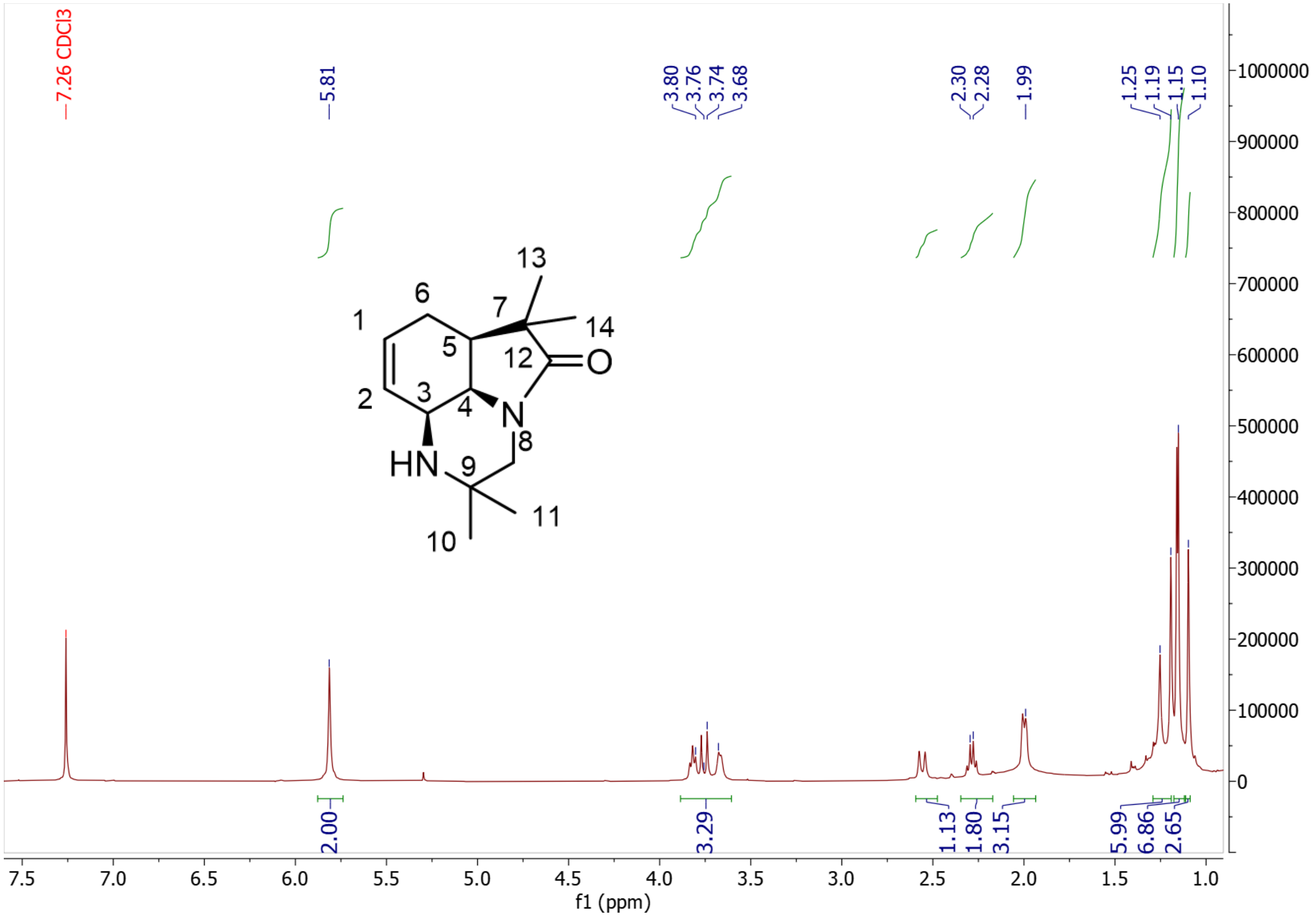

Supplementary Fig. 117: ^{1}H-NMR ($CDCl_3$) of Compound **54**.

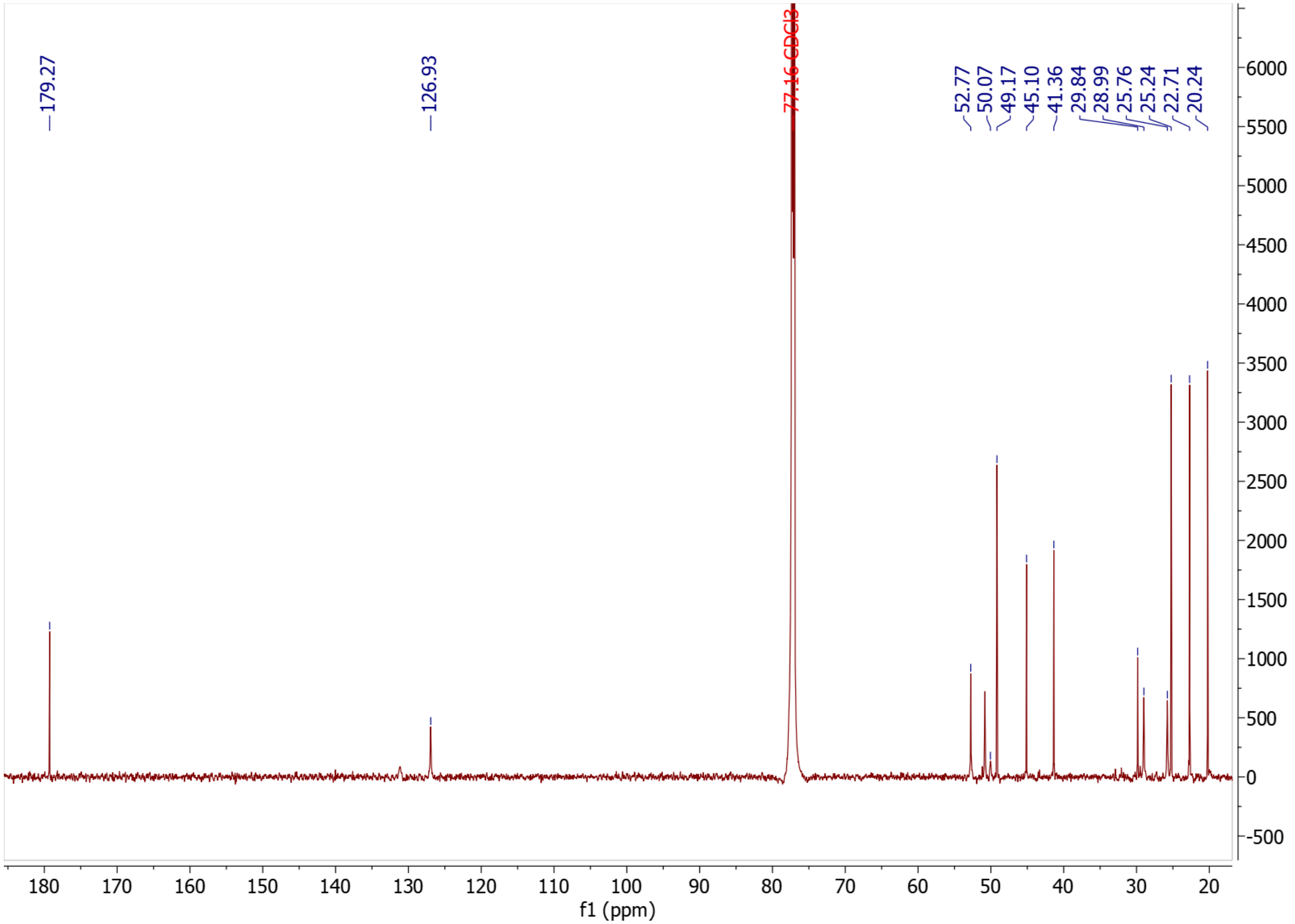

Supplementary Fig. 118: ^{13}C-NMR ($CDCl_3$) of Compound **54**.

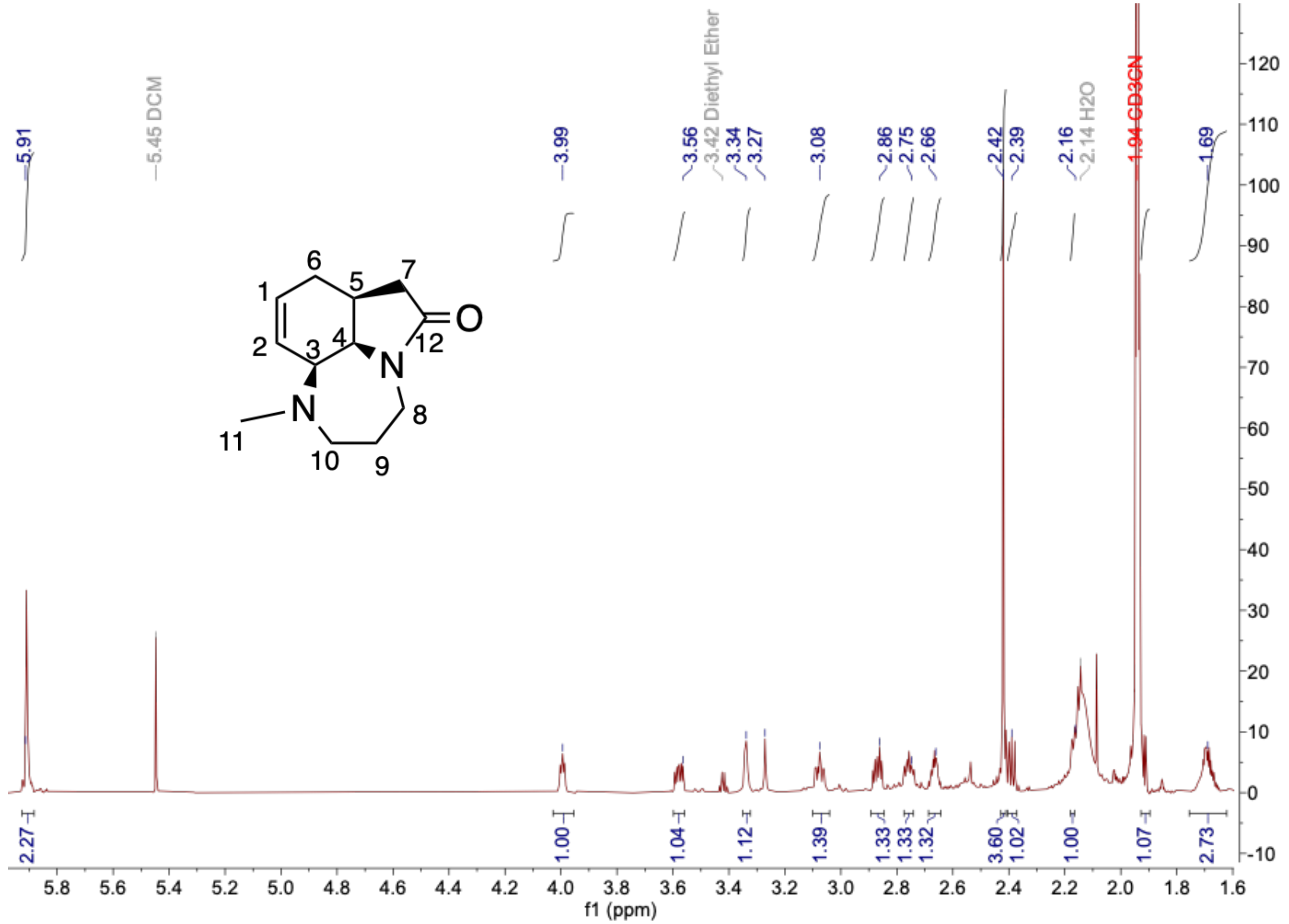

Supplementary Fig. 119: ^{1}H-NMR (CD_3CN) of Compound **55**.

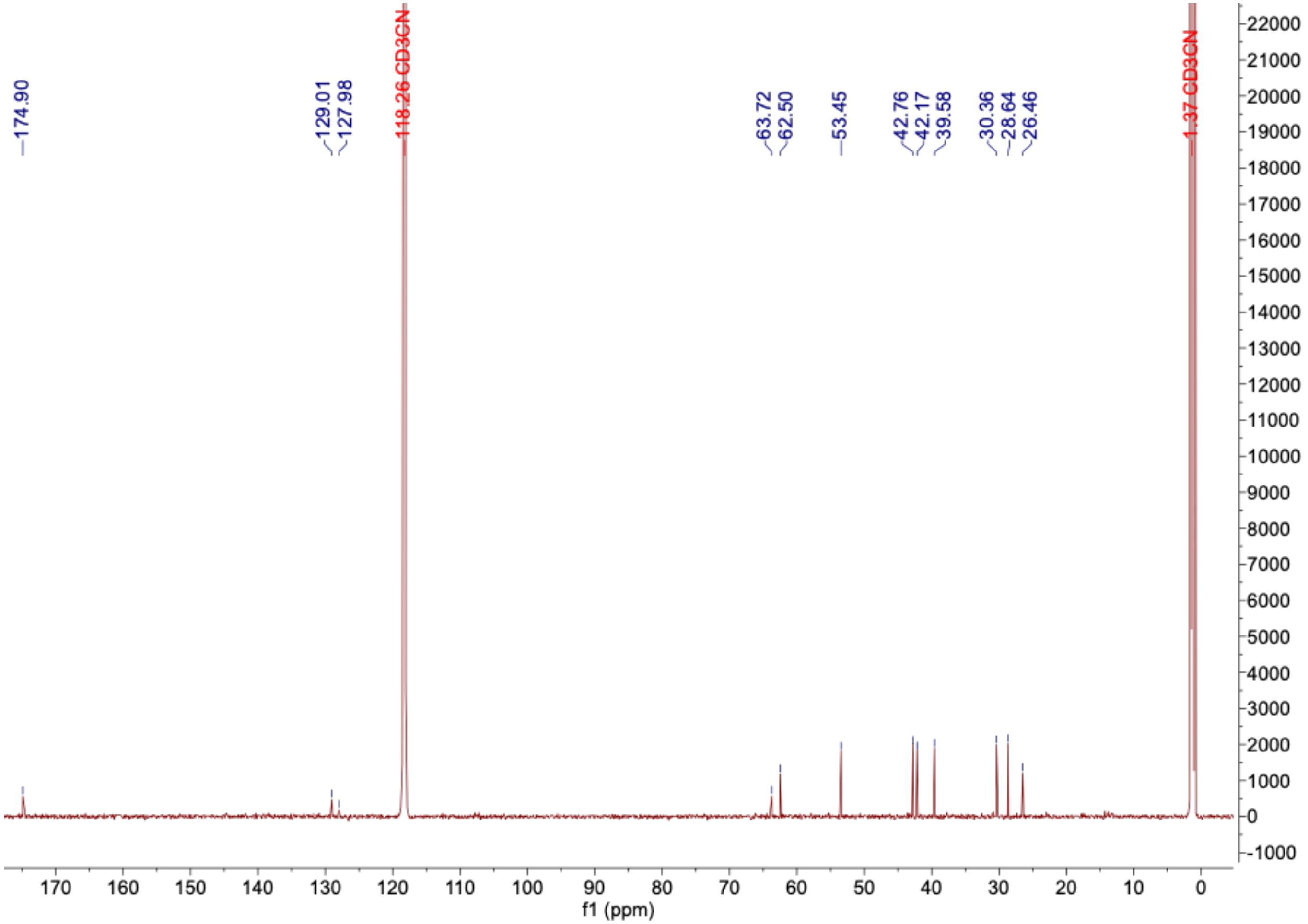

Supplementary Fig. 120: ^{13}C-NMR (CD_3CN) of Compound **55**.

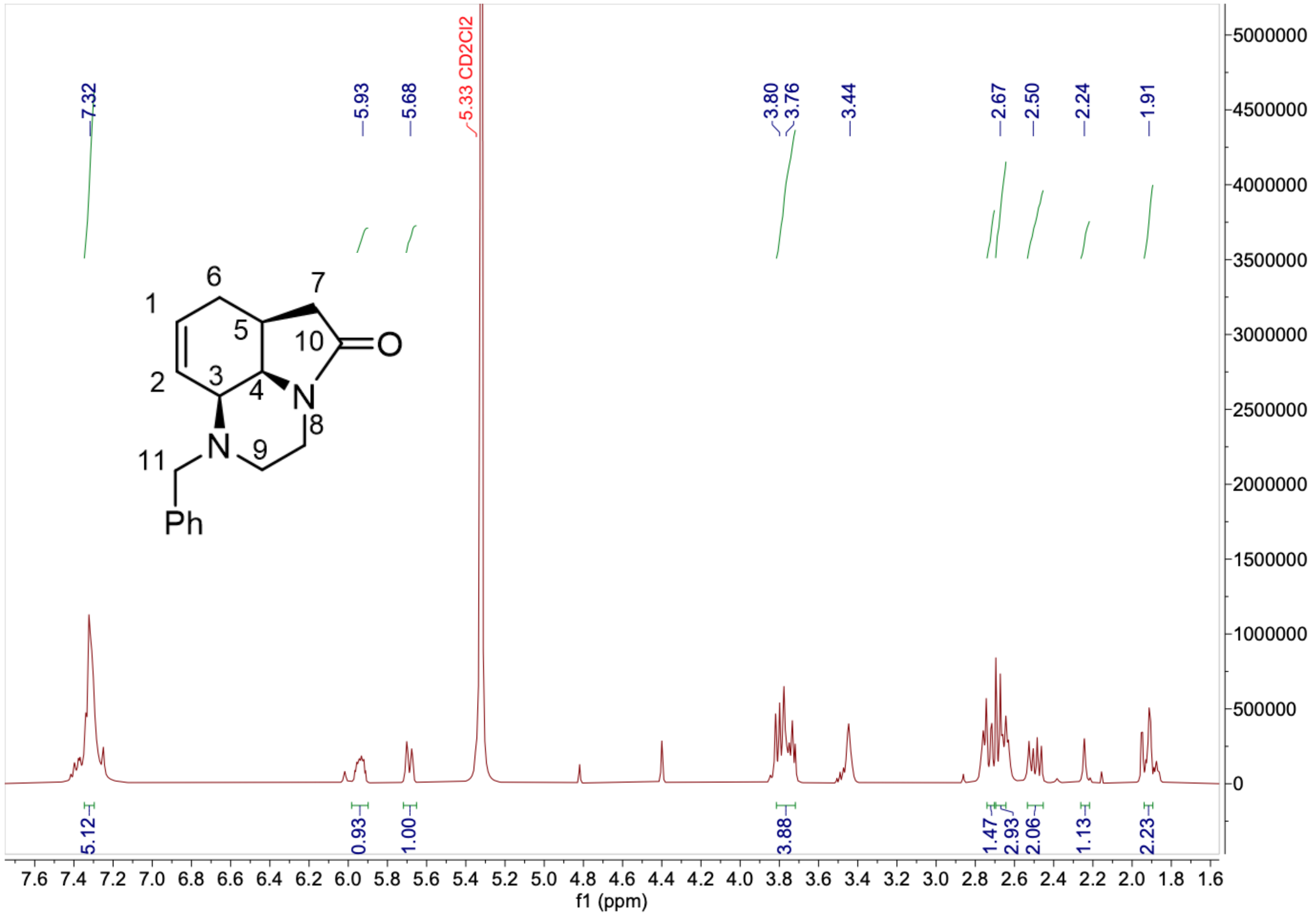

Supplementary Fig. 121: ^{1}H-NMR (CD_2Cl_2) of Compound **56**.

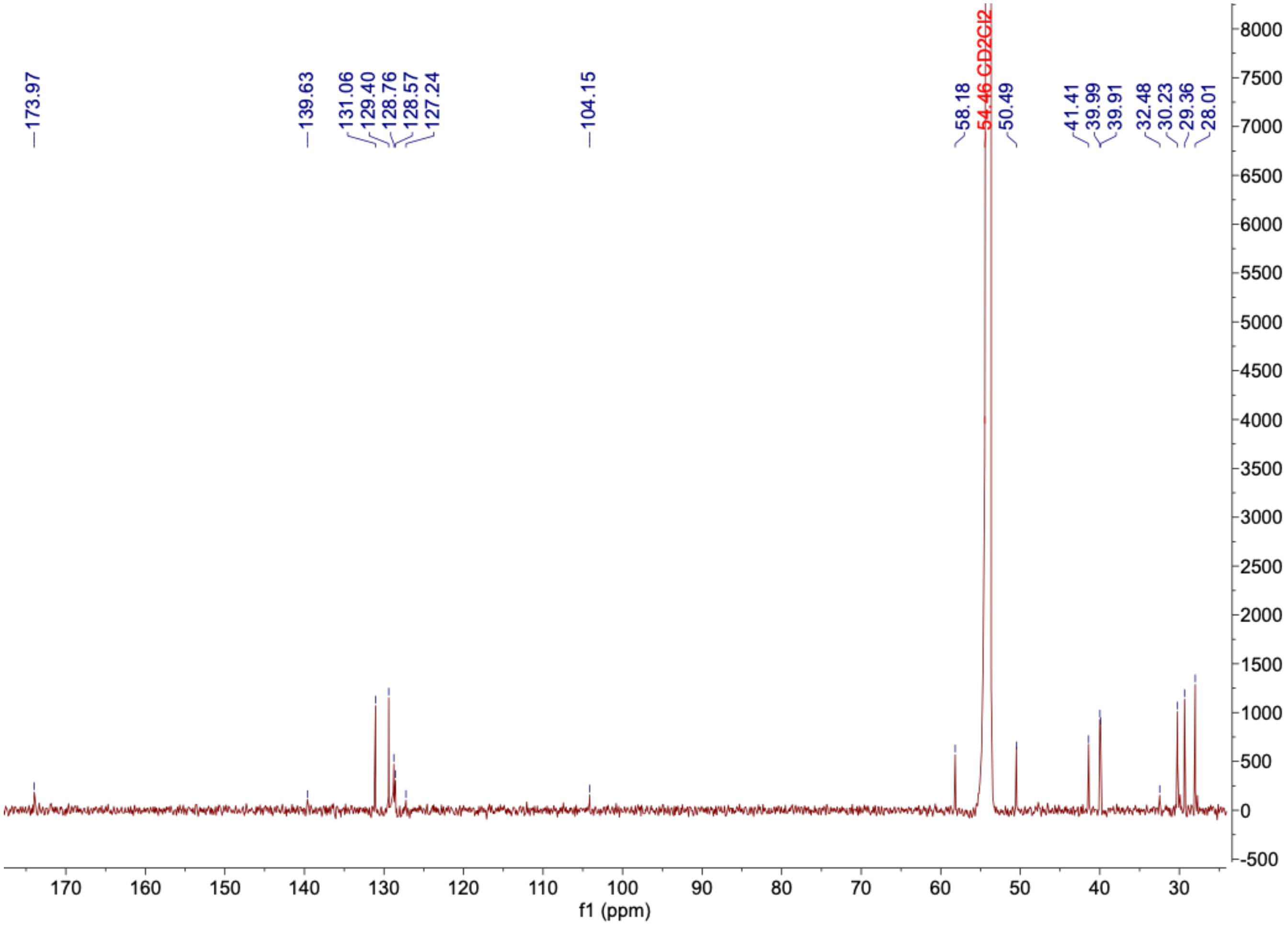

Supplementary Fig. 122: ^{13}C-NMR (CD_2Cl_2) of Compound **56**.

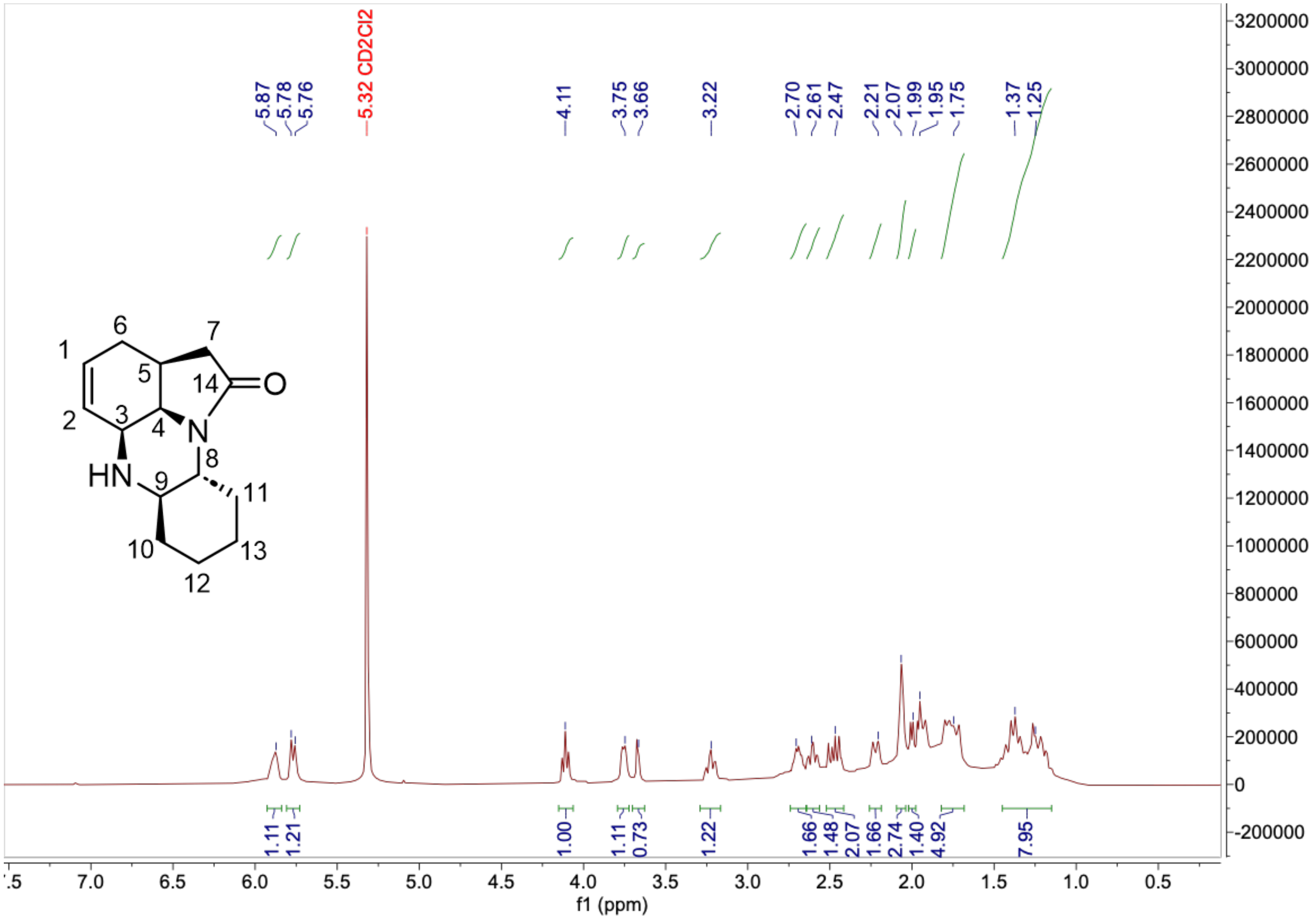

Supplementary Fig. 123: ^{1}H-NMR (CD_2Cl_2) of Compound **57**.

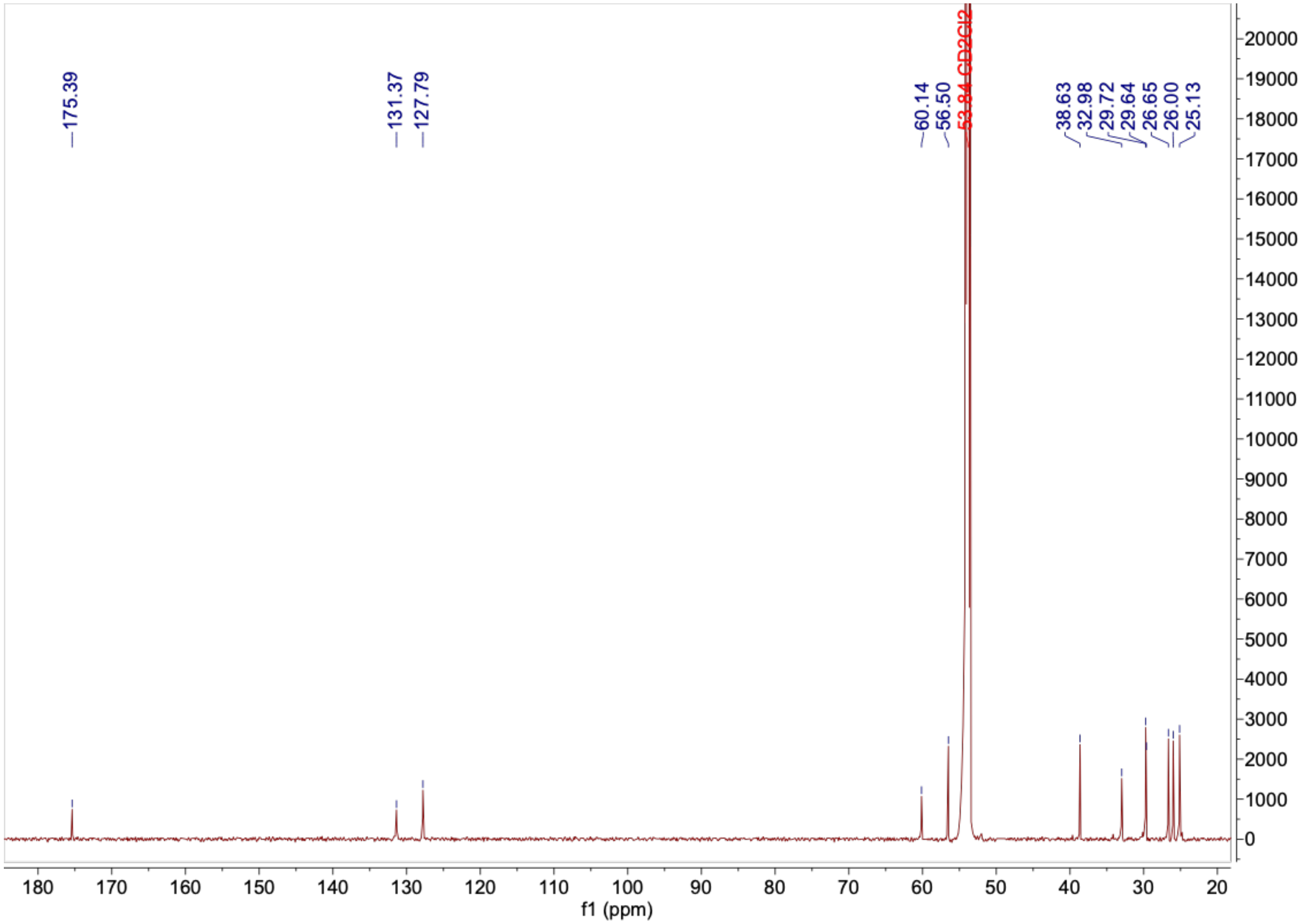

Supplementary Fig. 124: ^{13}C-NMR (CD_2Cl_2) of Compound **57**.

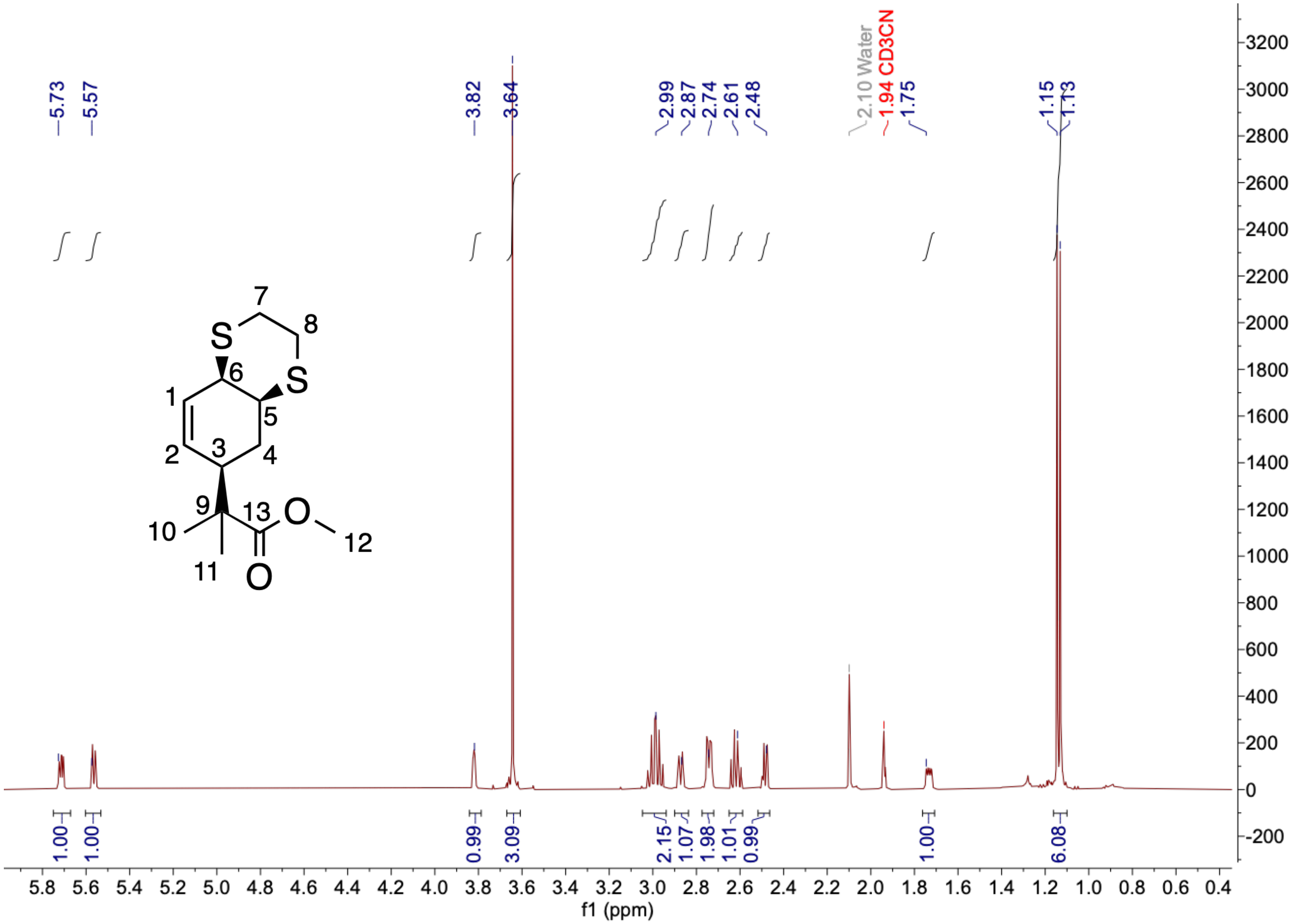

Supplementary Fig. 125: ^{1}H-NMR (CD_3CN) of Compound **58**.

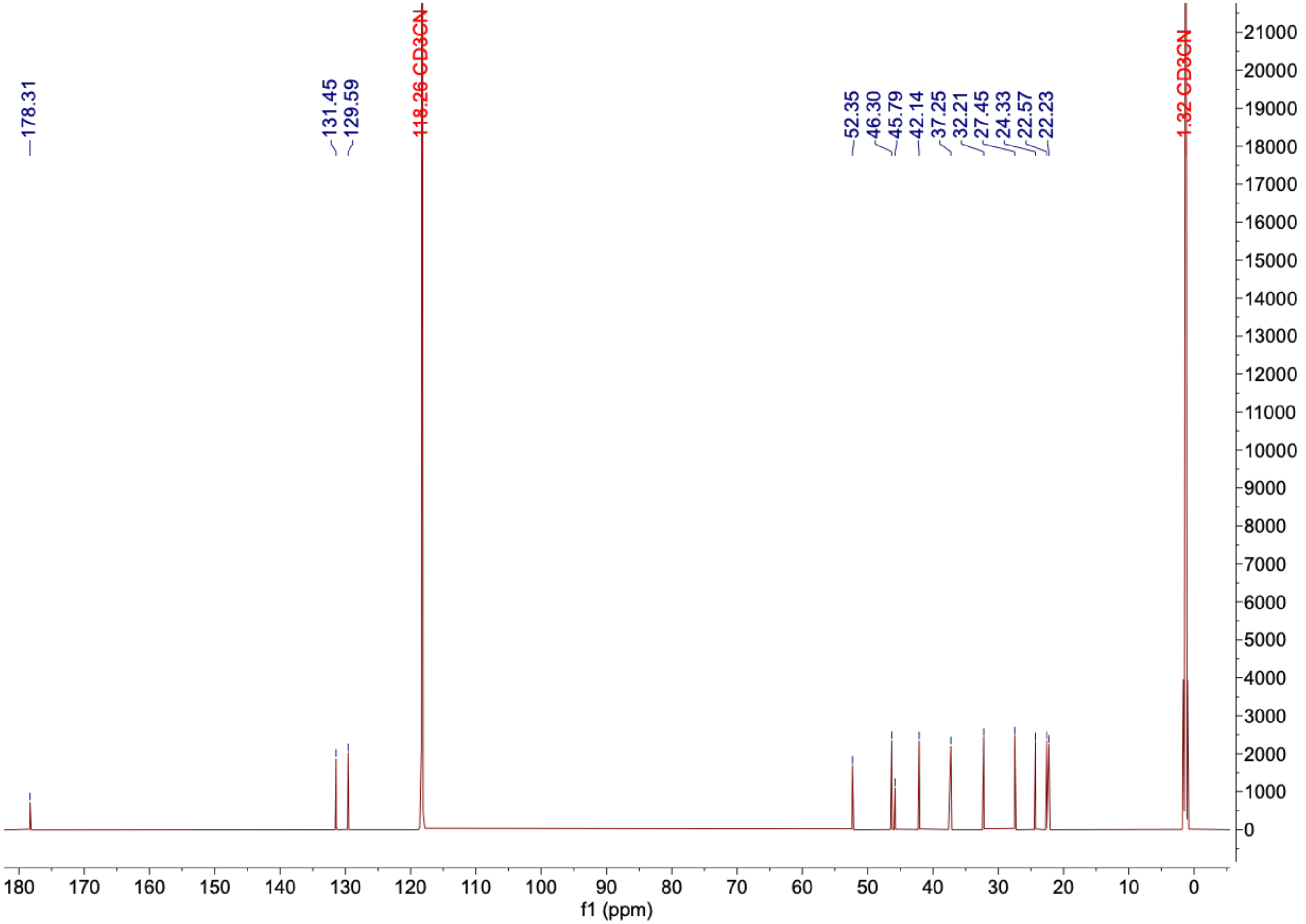

Supplementary Fig. 126: ^{13}C-NMR (CD_3CN) of Compound **58**.

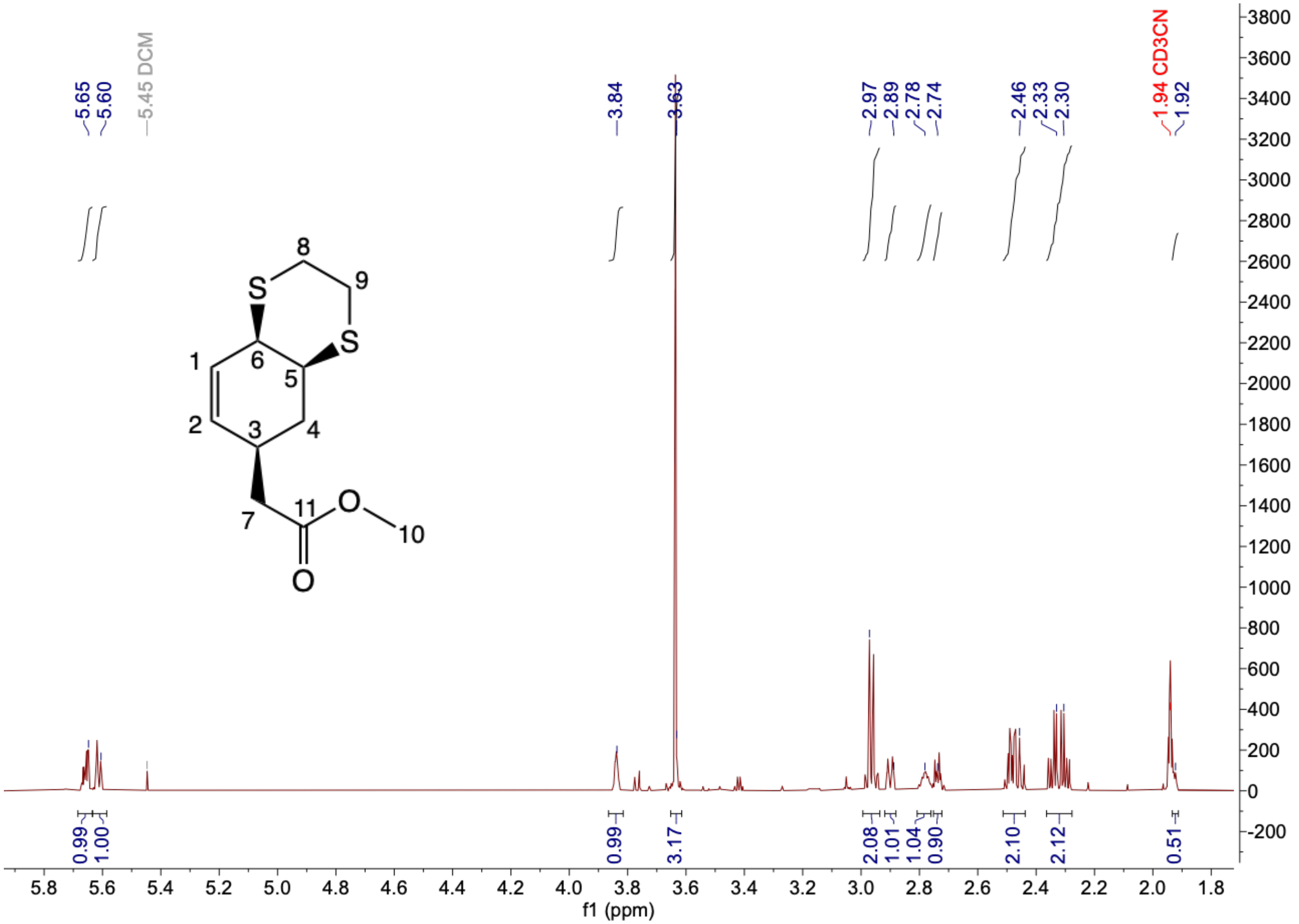

Supplementary Fig. 127: ^{1}H-NMR (CD_3CN) of Compound **59**.

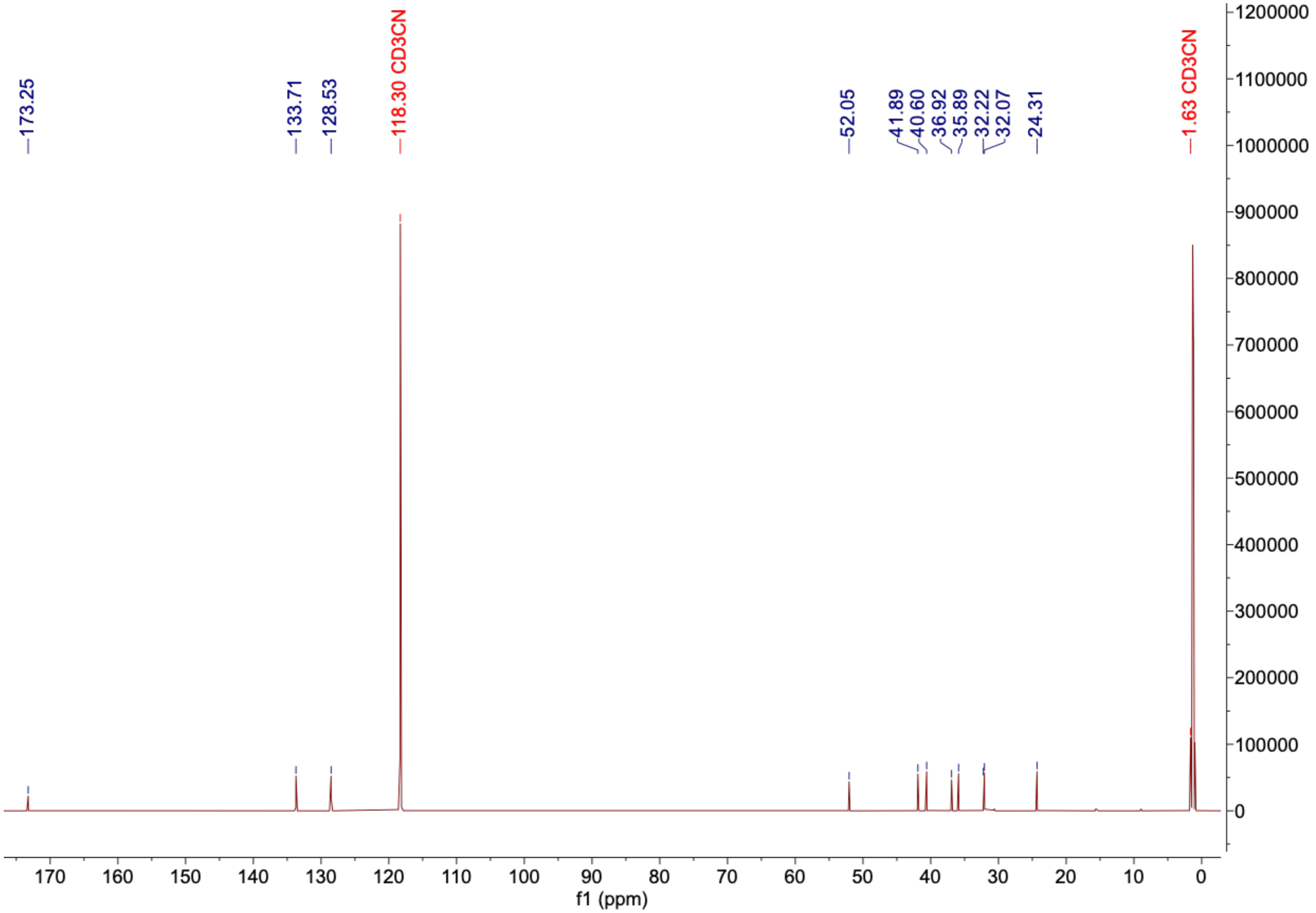

Supplementary Fig. 128: ^{13}C-NMR (CD_3CN) of Compound **59**.

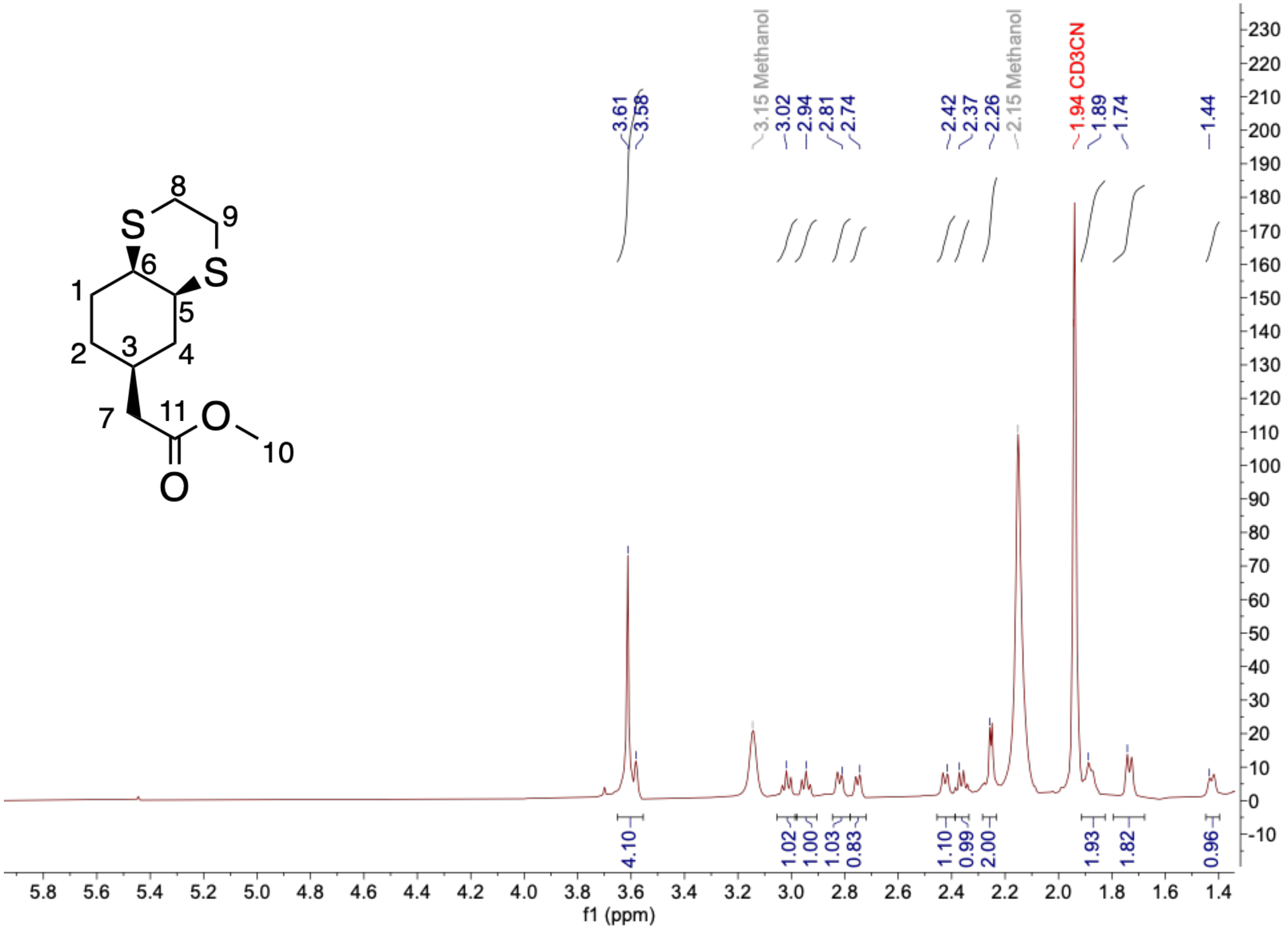

Supplementary Fig. 129: ^{1}H-NMR (CD_3CN) of Compound **60**.

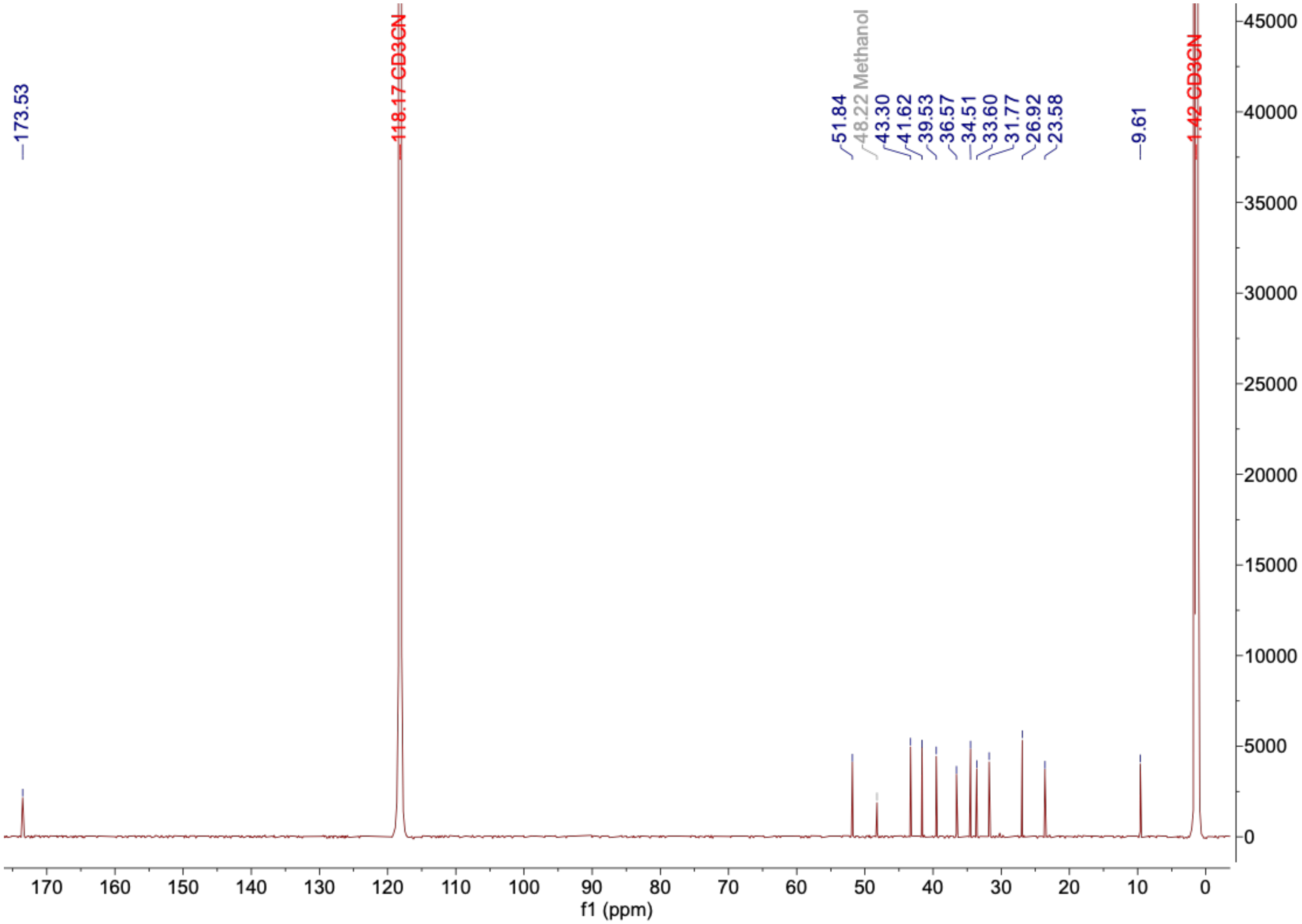

Supplementary Fig. 130: ^{13}C-NMR (CD_3CN) of Compound **60**.

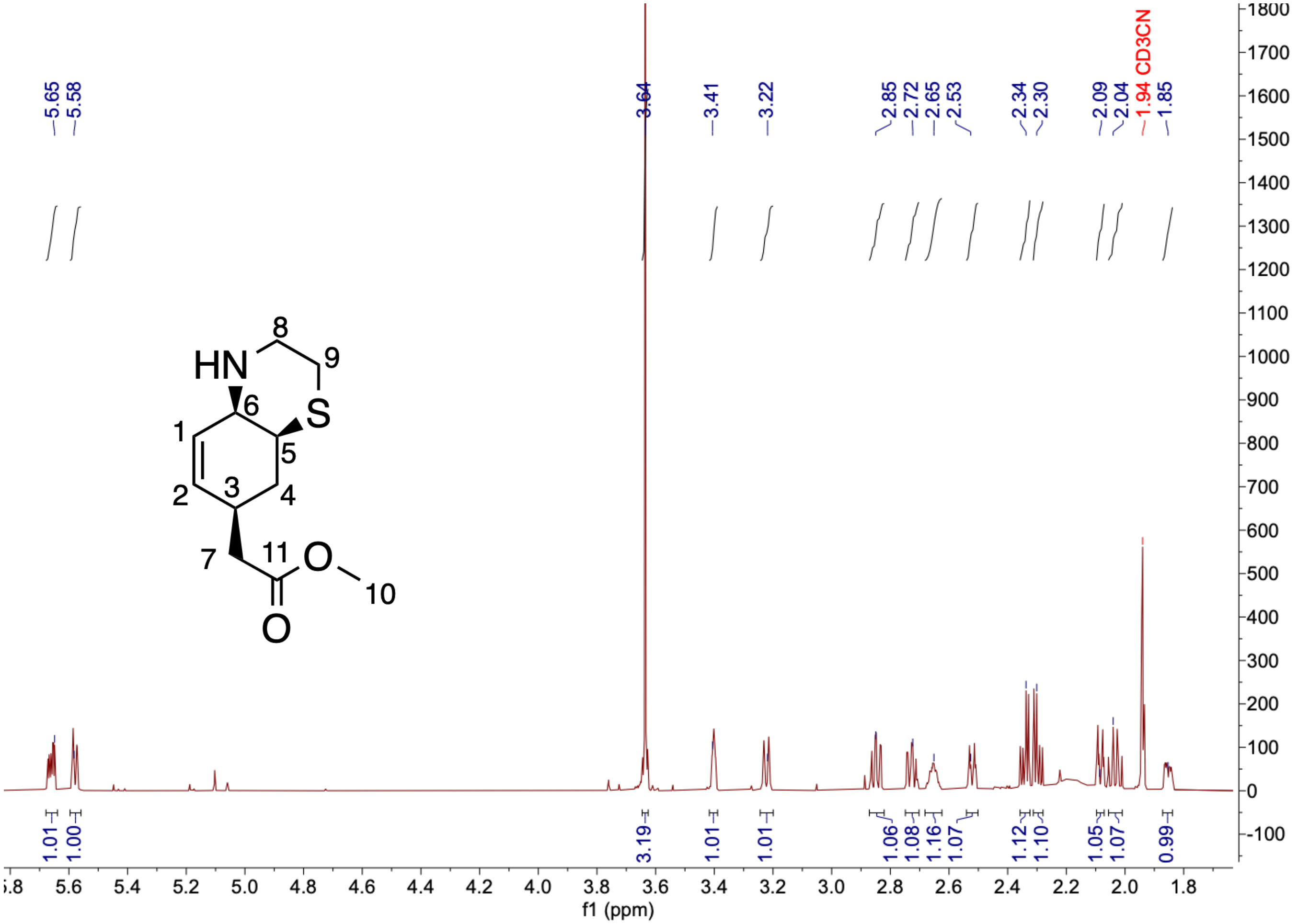

Supplementary Fig. 131: ^{1}H-NMR (CD_3CN) of Compound **61**.

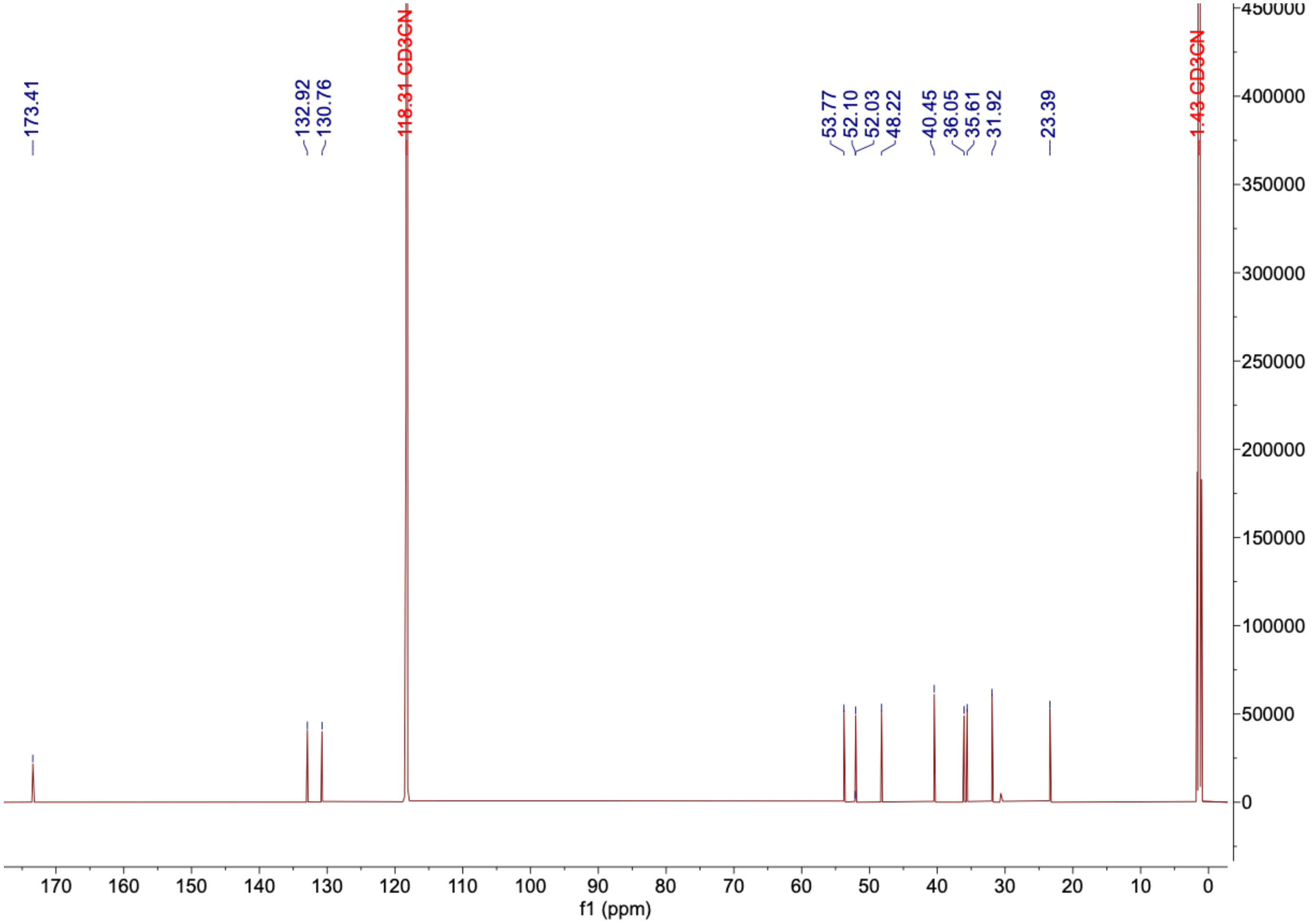

Supplementary Fig. 132: ^{13}C-NMR (CD_3CN) of Compound **61**.

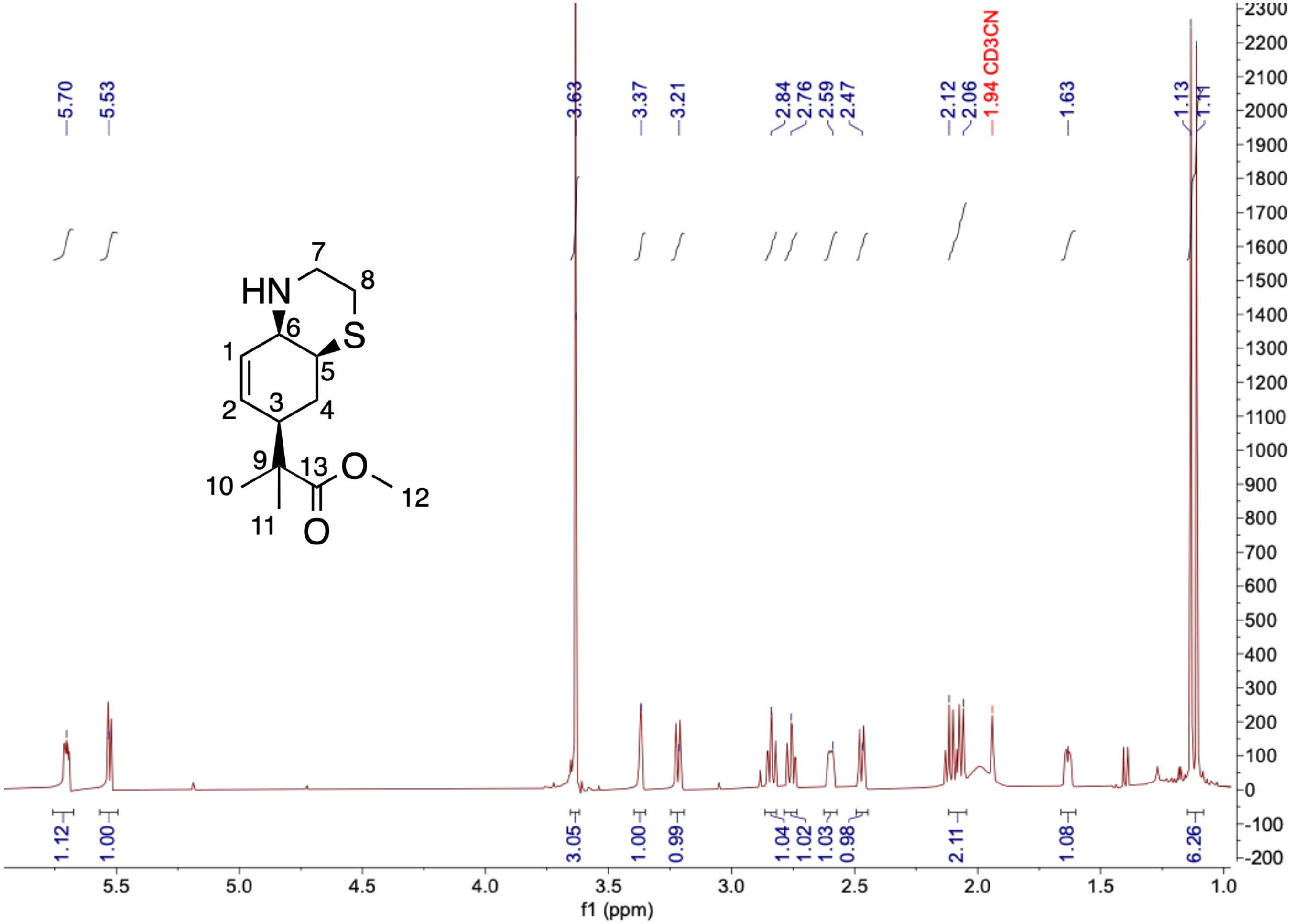

Supplementary Fig. 133: ^{1}H-NMR (CD_3CN) of Compound **62**.

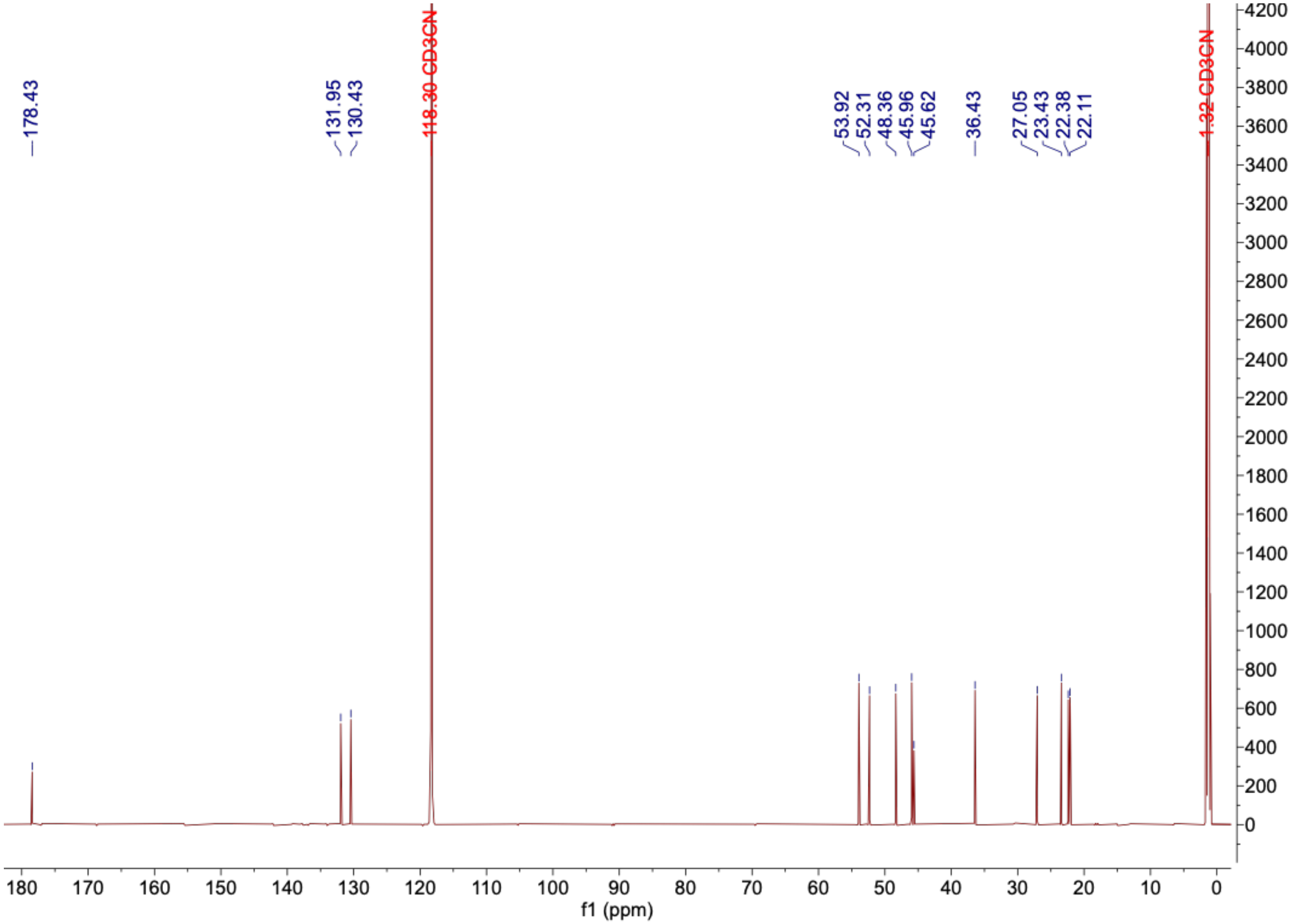

Supplementary Fig. 134: ^{13}C-NMR (CD_3CN) of Compound **62**.

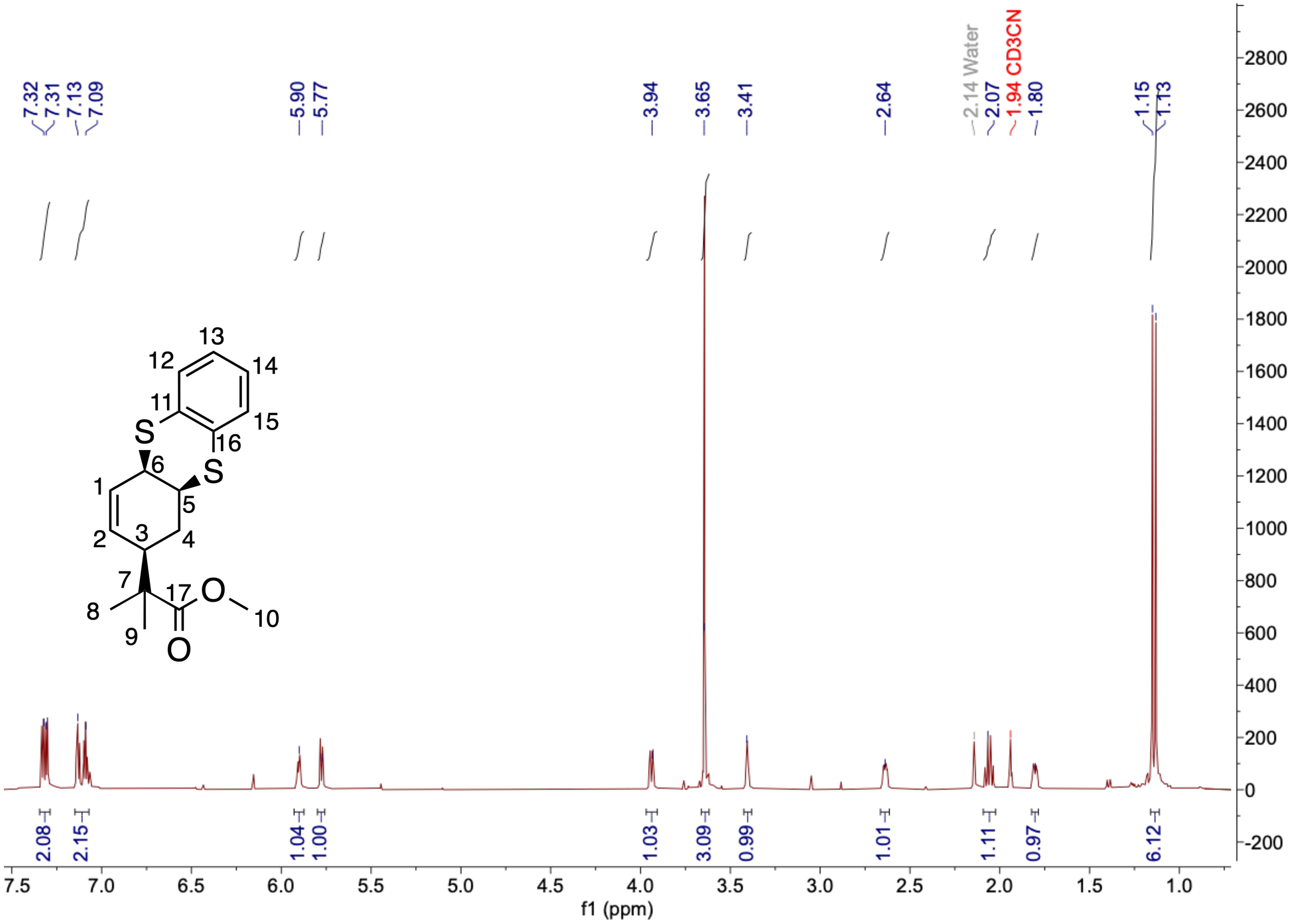

Supplementary Fig. 135: ^{1}H-NMR (CD_3CN) of Compound **63**.

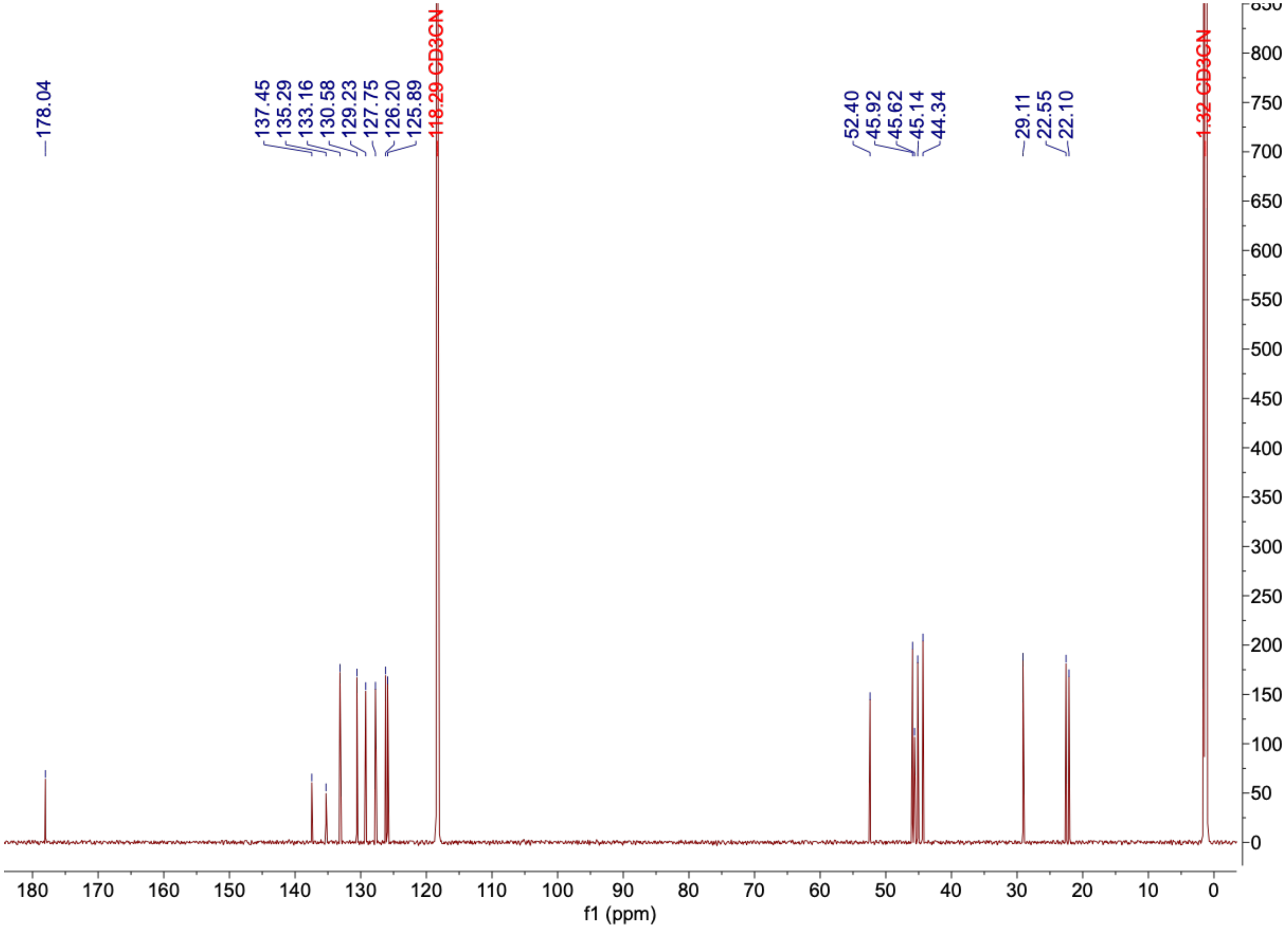

Supplementary Fig. 136: ^{13}C-NMR (CD_3CN) of Compound **63**.

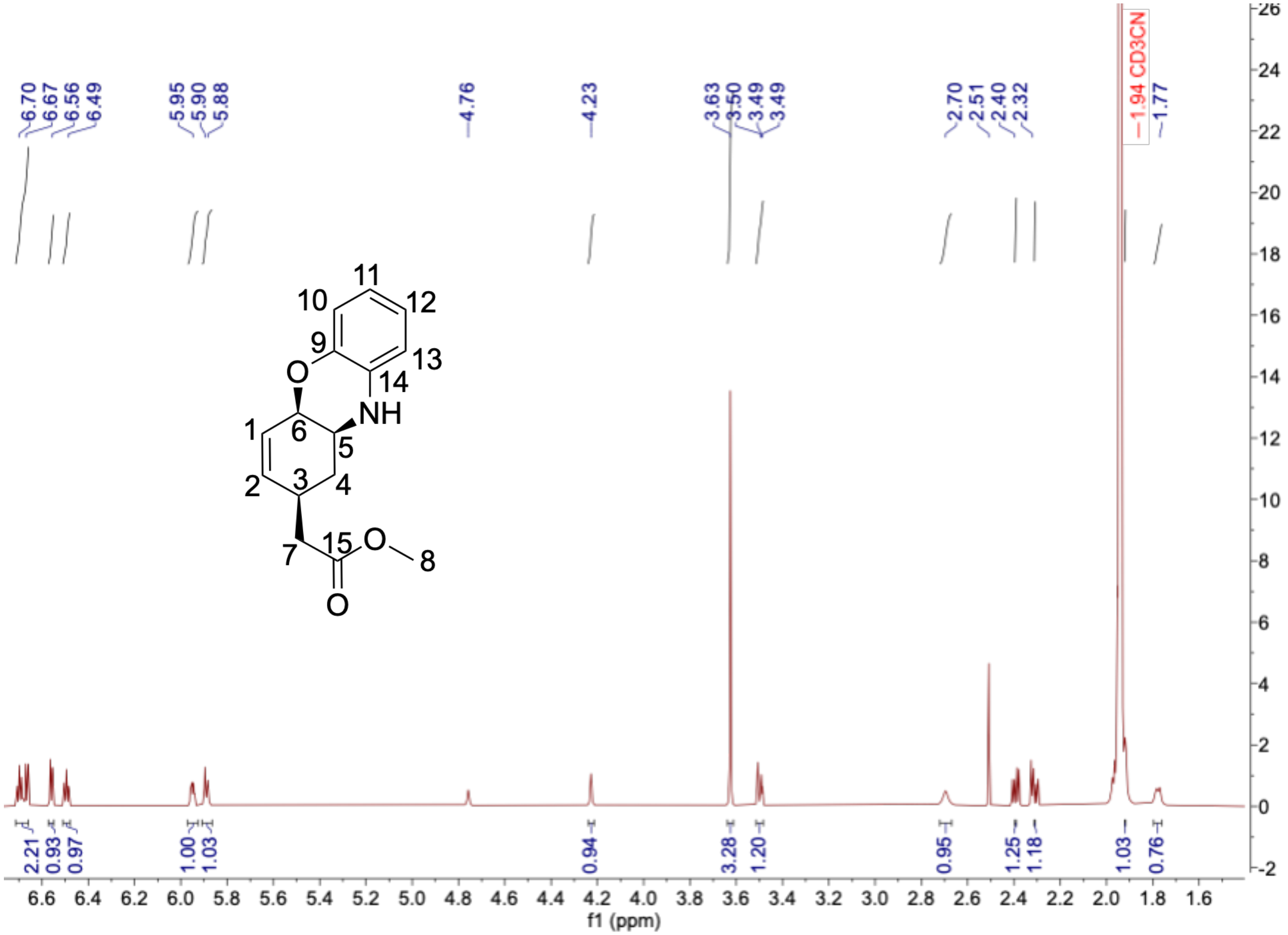

Supplementary Fig. 137: ^{1}H-NMR (CD_3CN) of Compound **64**.

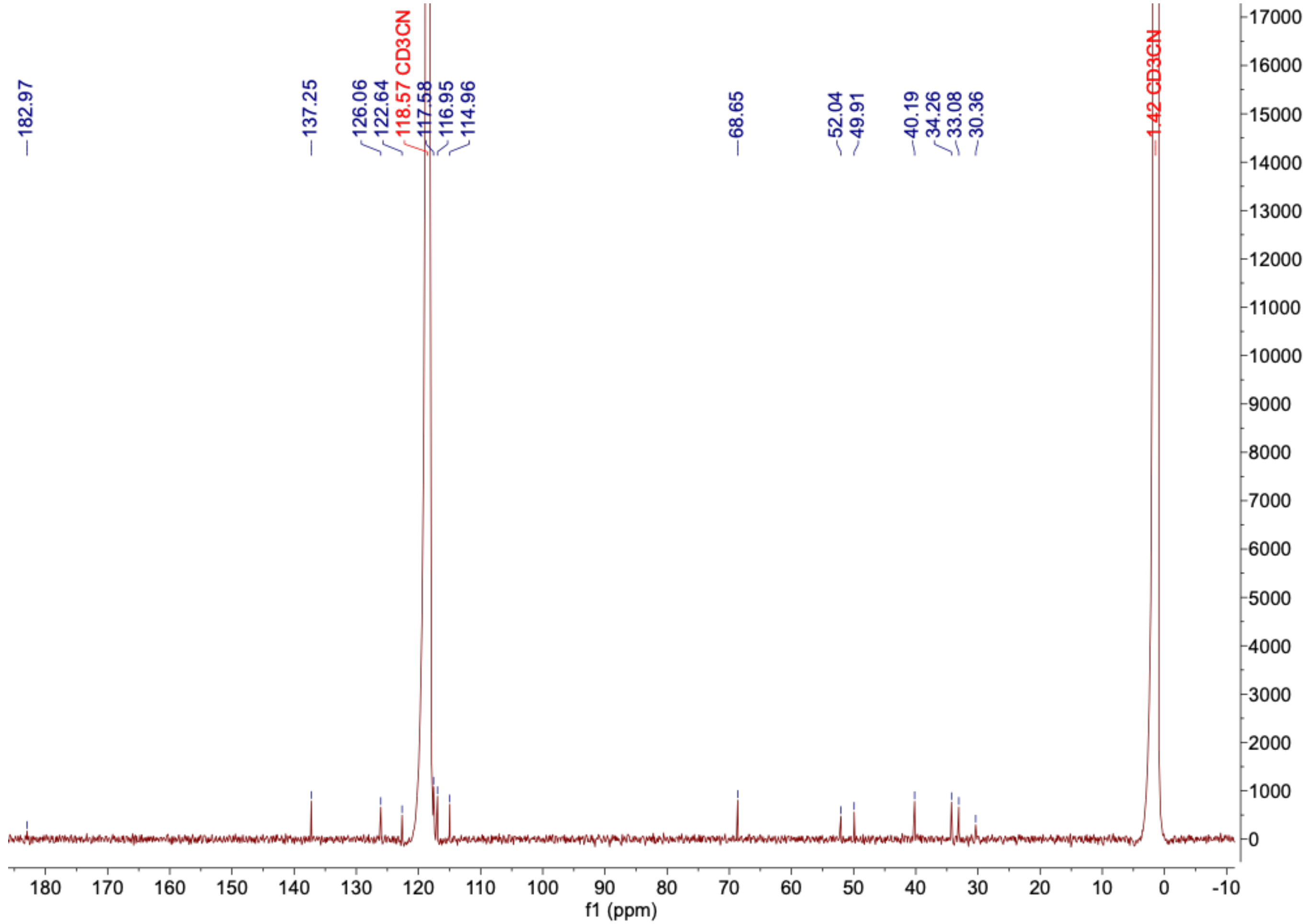

Supplementary Fig. 138: ^{13}C-NMR (CD_3CN) of Compound **64**.

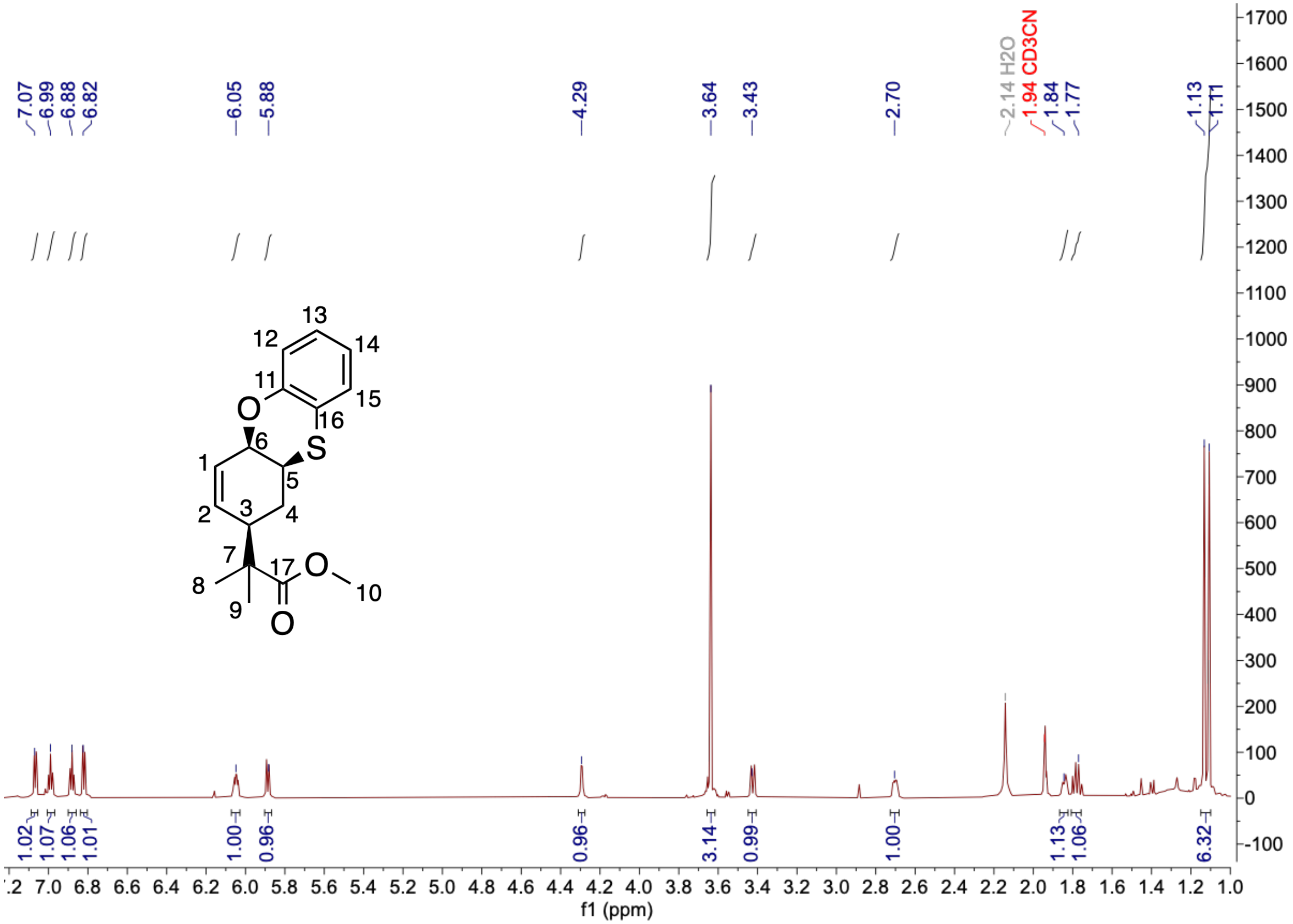

Supplementary Fig. 139: ¹H-NMR (CD_3CN) of Compound **65**.

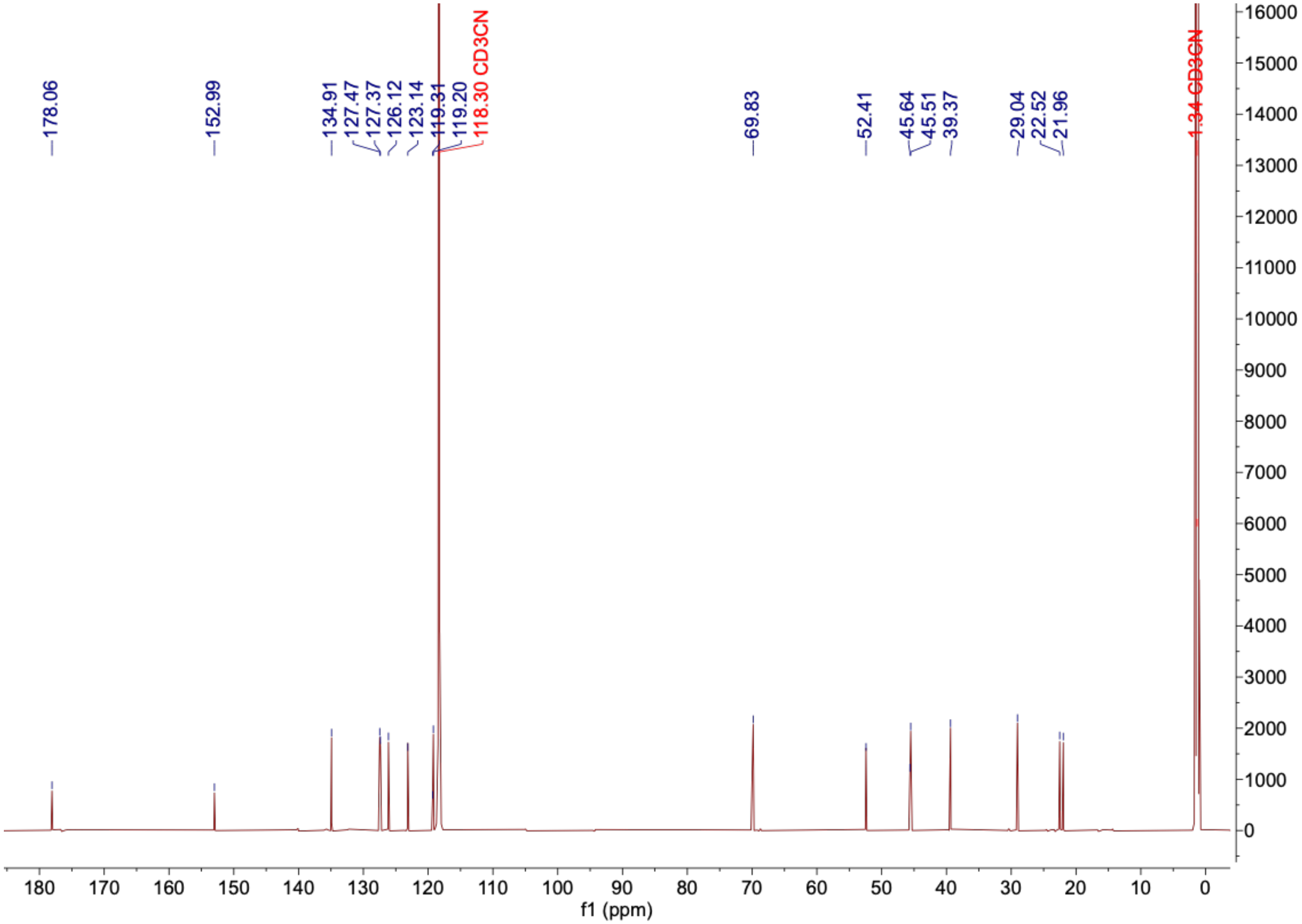

Supplementary Fig. 140: ^{13}C-NMR (CD_3CN) of Compound **65**.

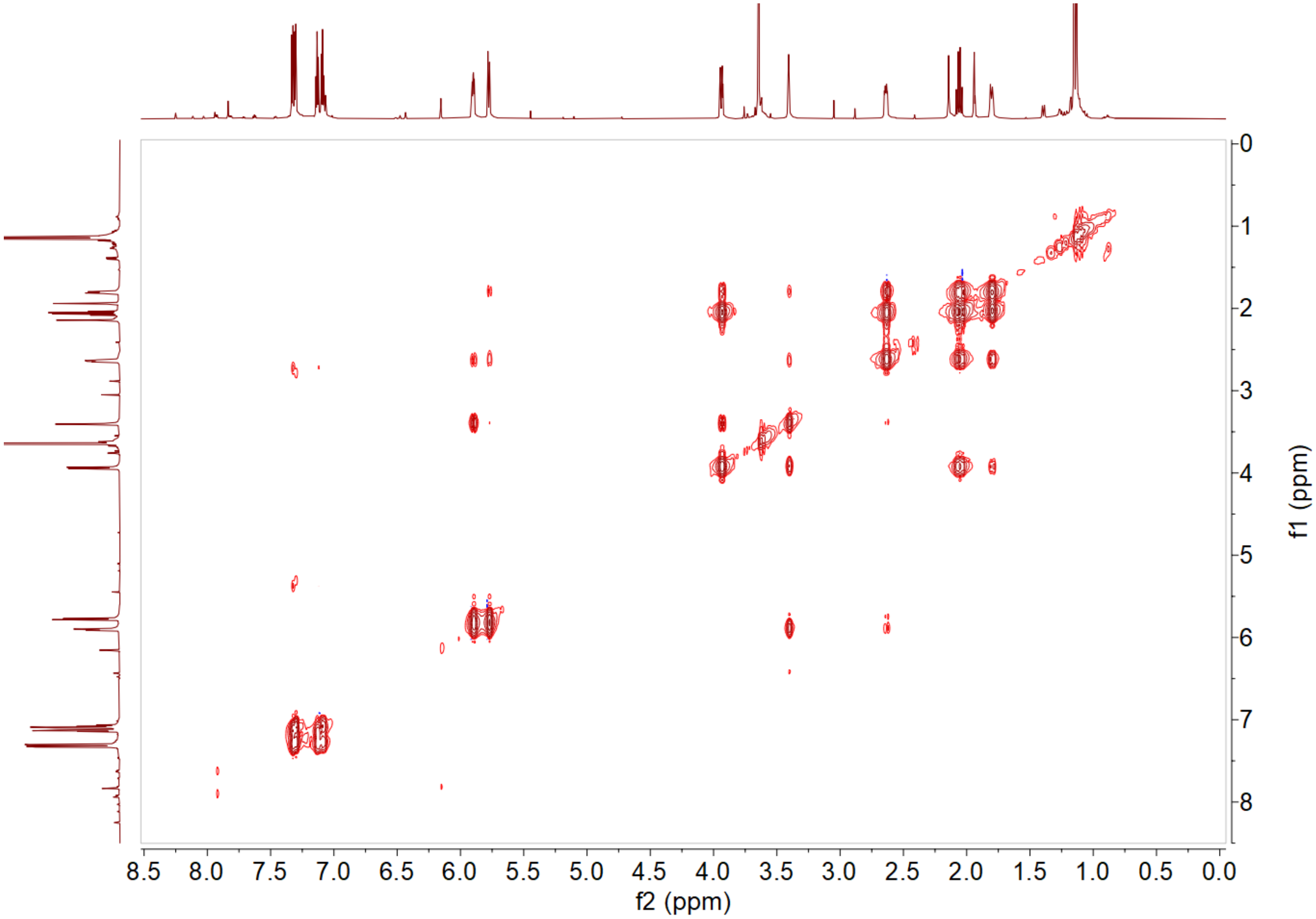

Supplementary Fig. 141: ^{1}H-^{1}H COSY (CD_3CN) of Compound **65**.

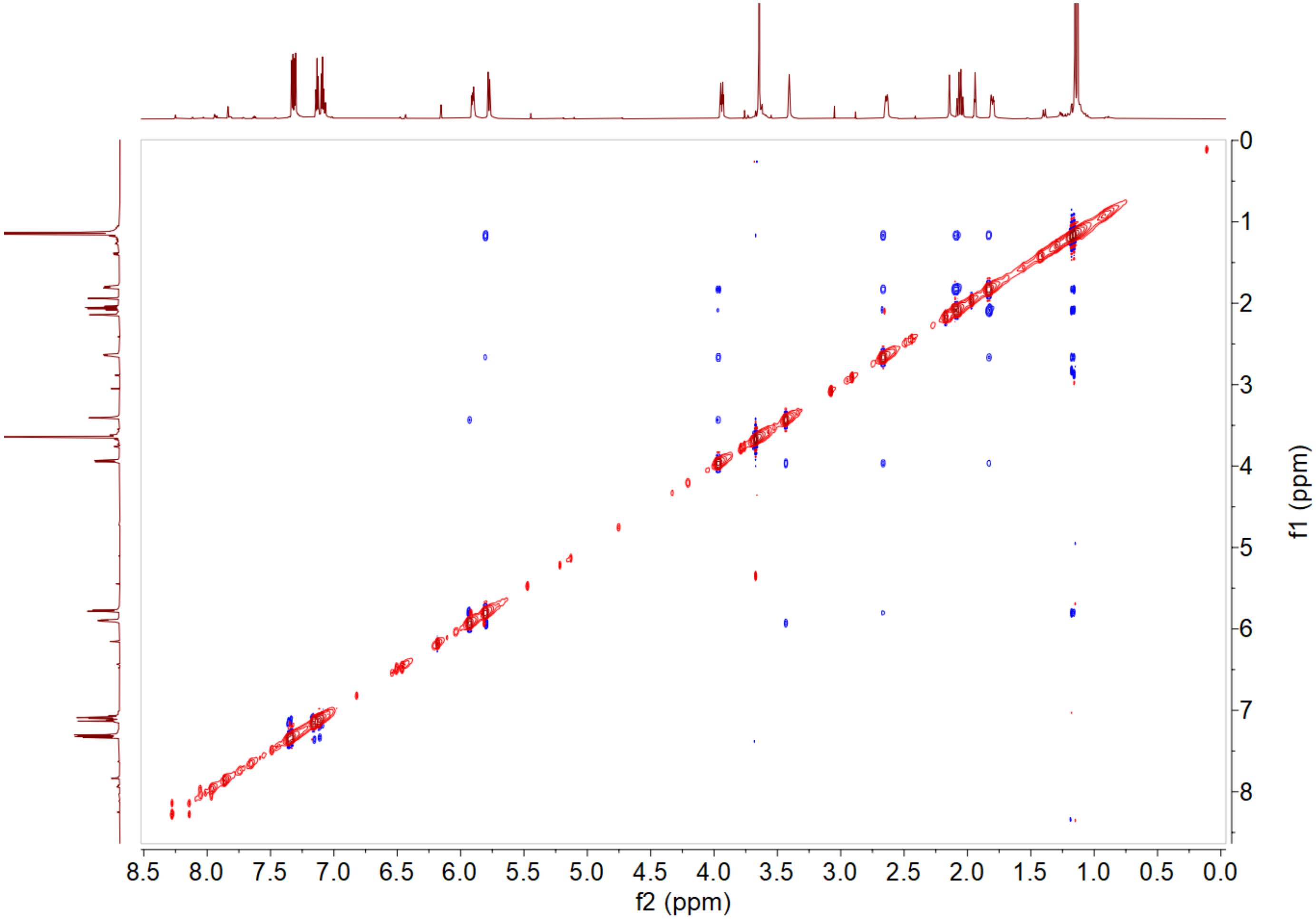

Supplementary Fig. 142: ^{1}H-^{1}H NOESY (CD_3CN) of Compound **65**.

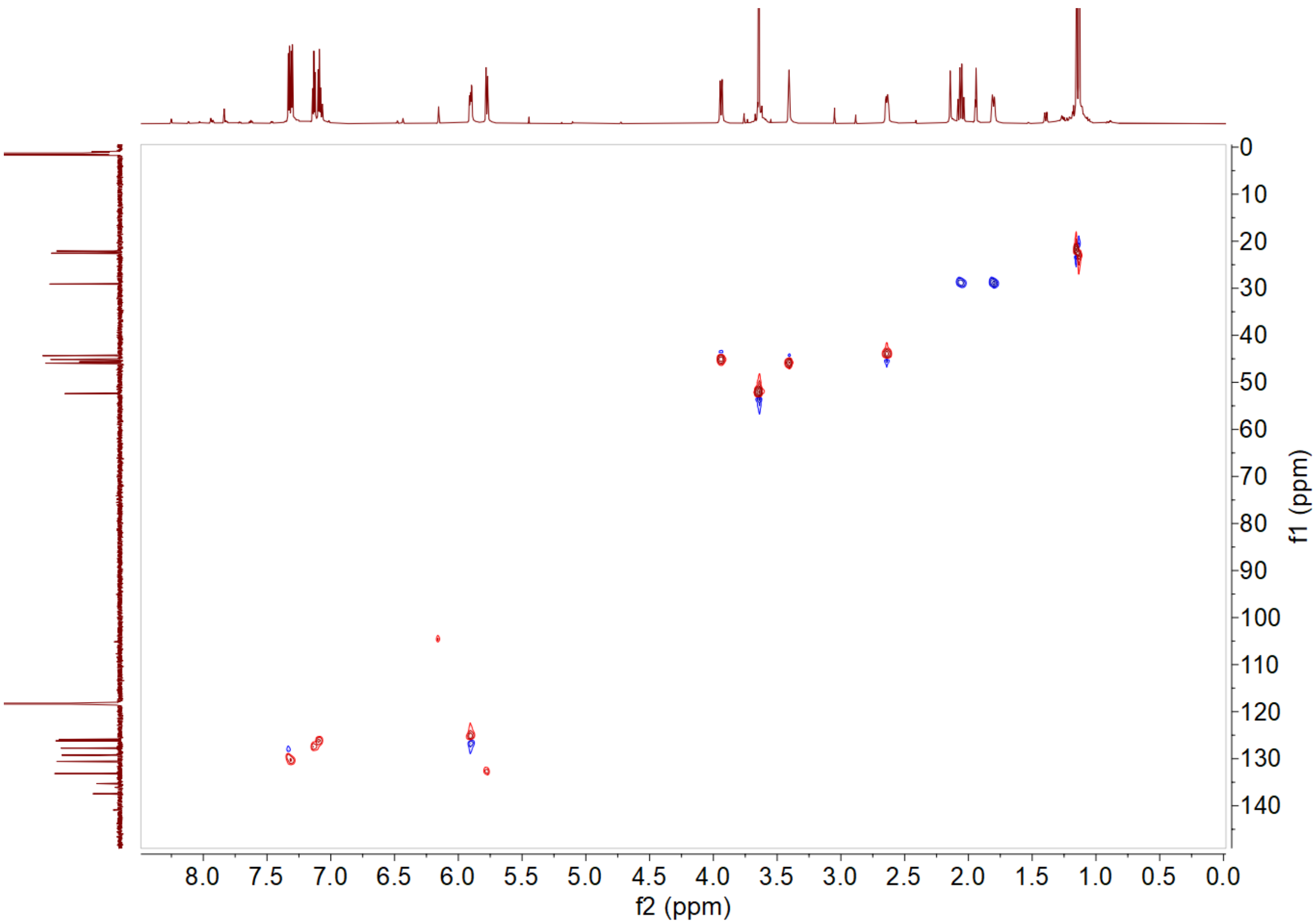

Supplementary Fig. 143: ^{1}H-^{13}C HSQC (CD_3CN) of Compound **65**.

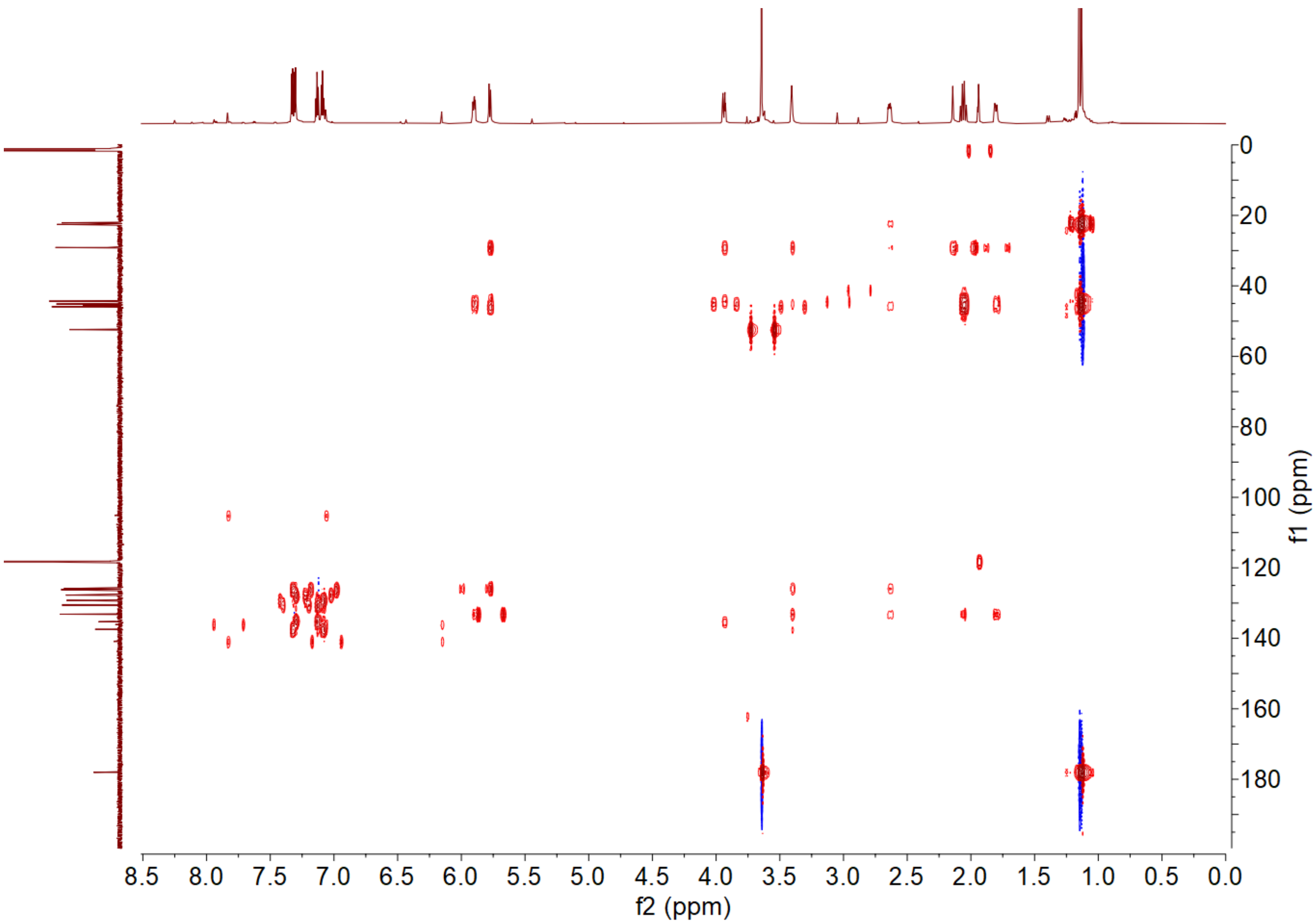

Supplementary Fig. 144: ^{1}H-^{13}C HMBC (CD_3CN) of Compound **65**.

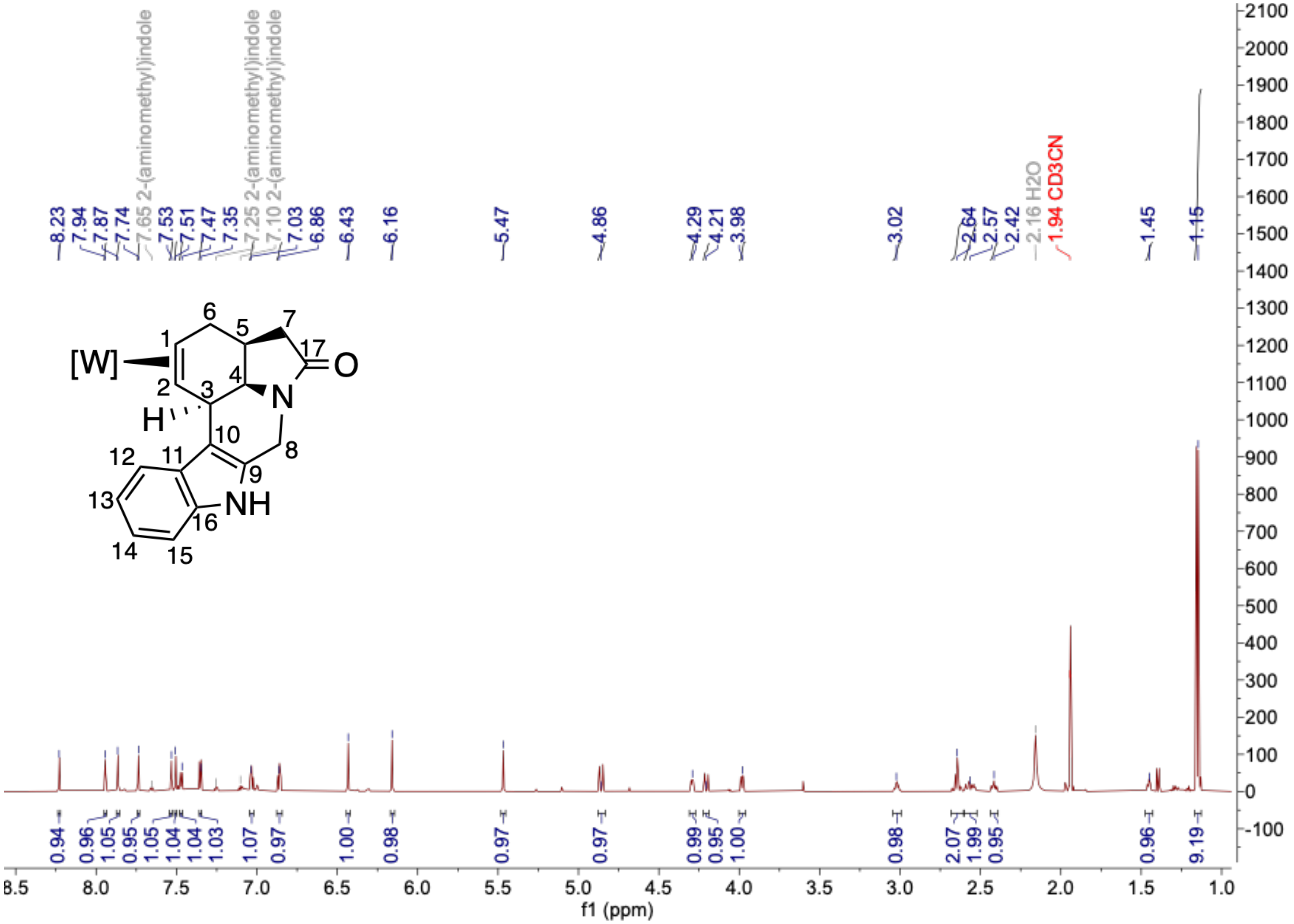

Supplementary Fig. 145: ^{1}H-NMR (CD_3CN) of Compound **67**.

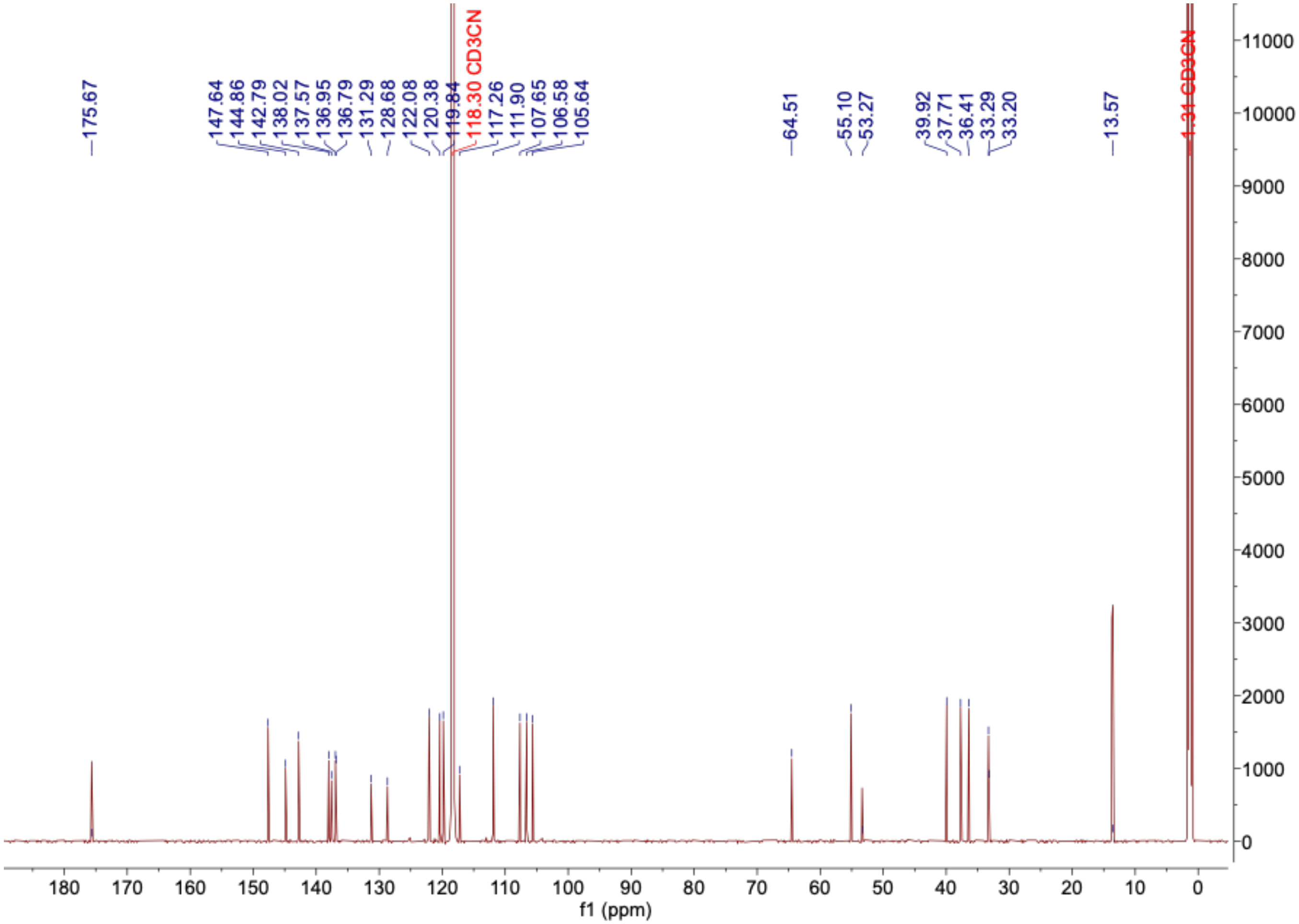

Supplementary Fig. 146: ^{13}C-NMR (CD_3CN) of Compound **67**.

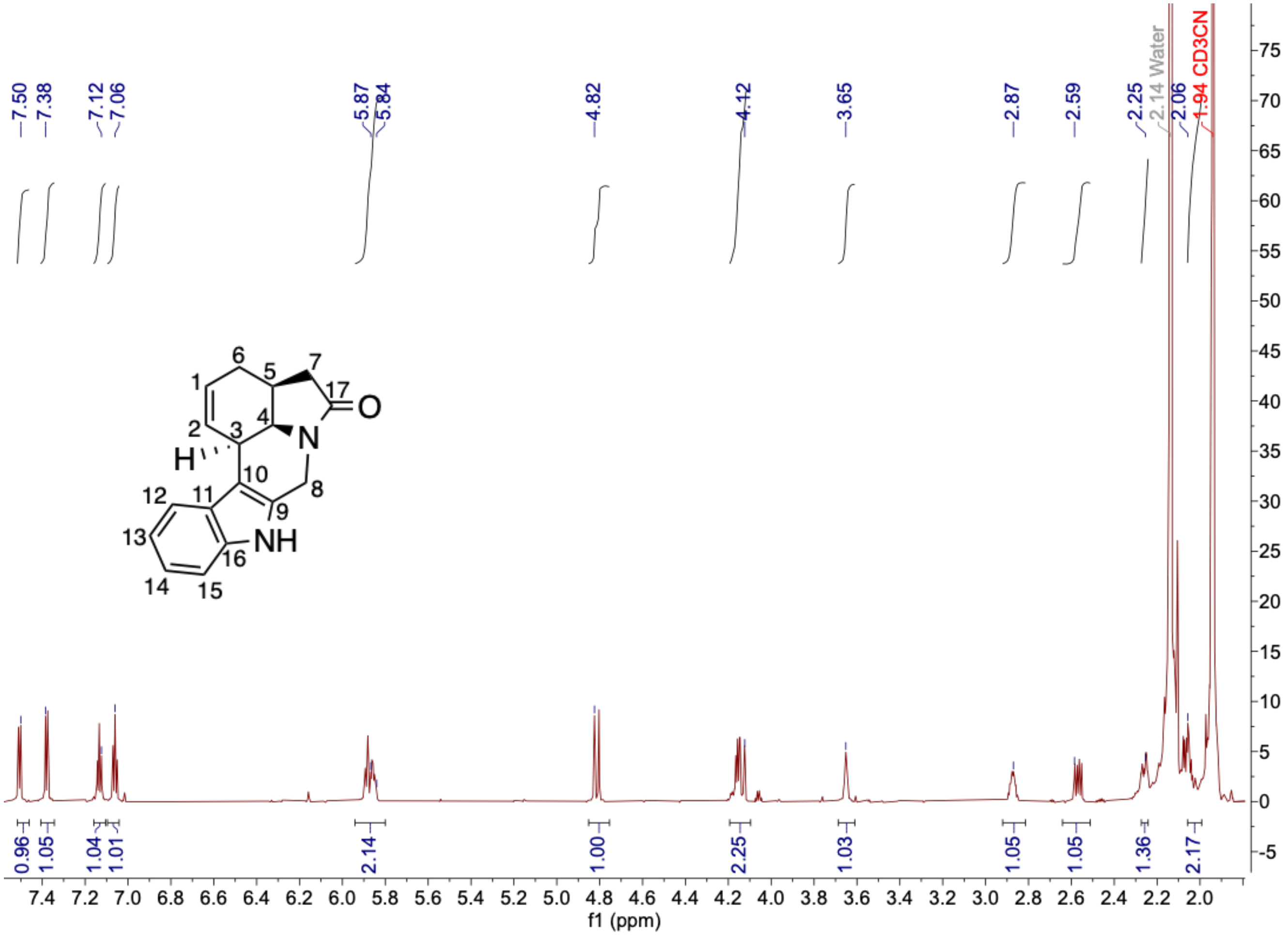

Supplementary Fig. 147: ^{1}H-NMR (CD_3CN) of Compound **68**.

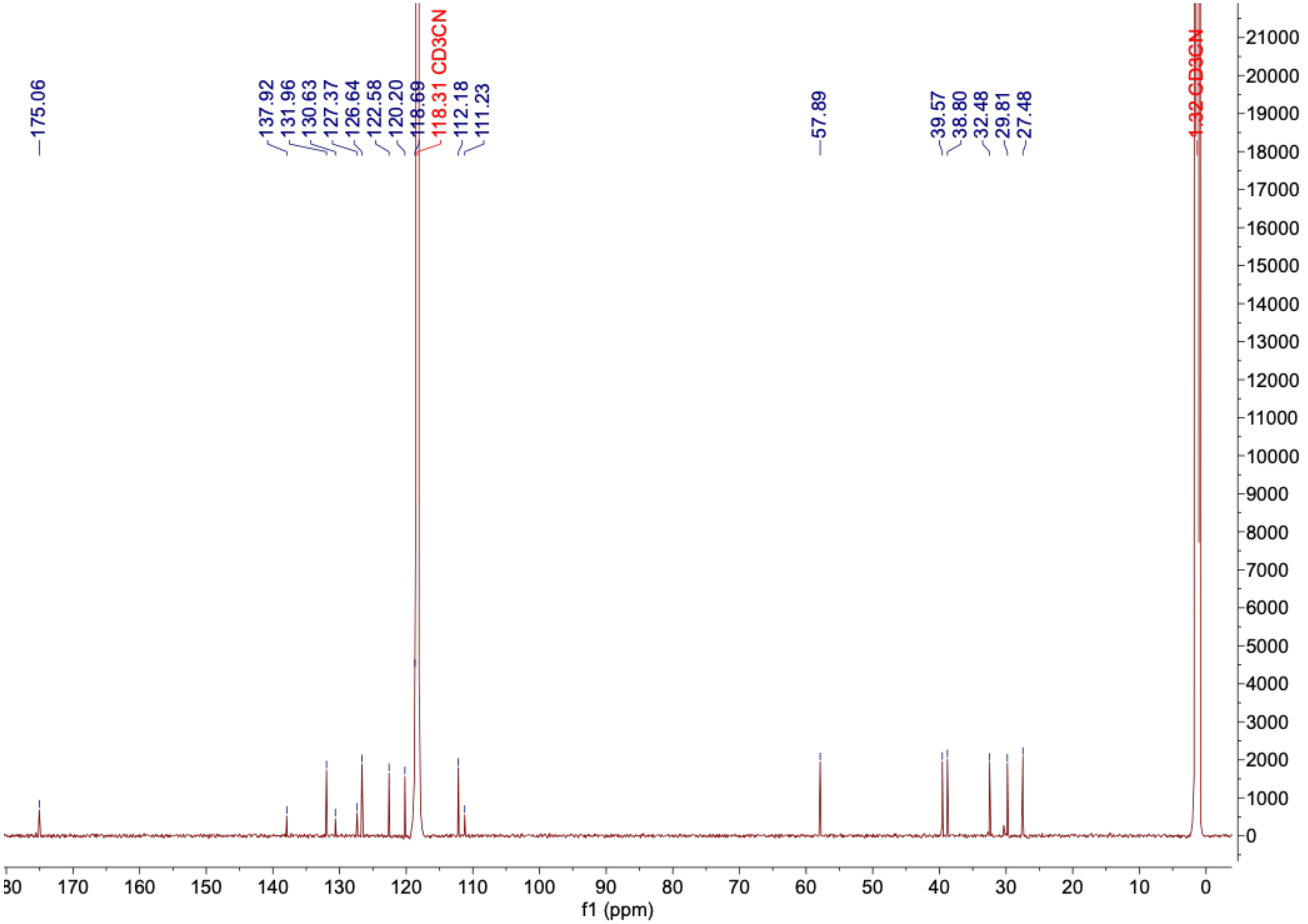

Supplementary Fig. 148: ^{13}C-NMR (CD_3CN) of Compound **68**.

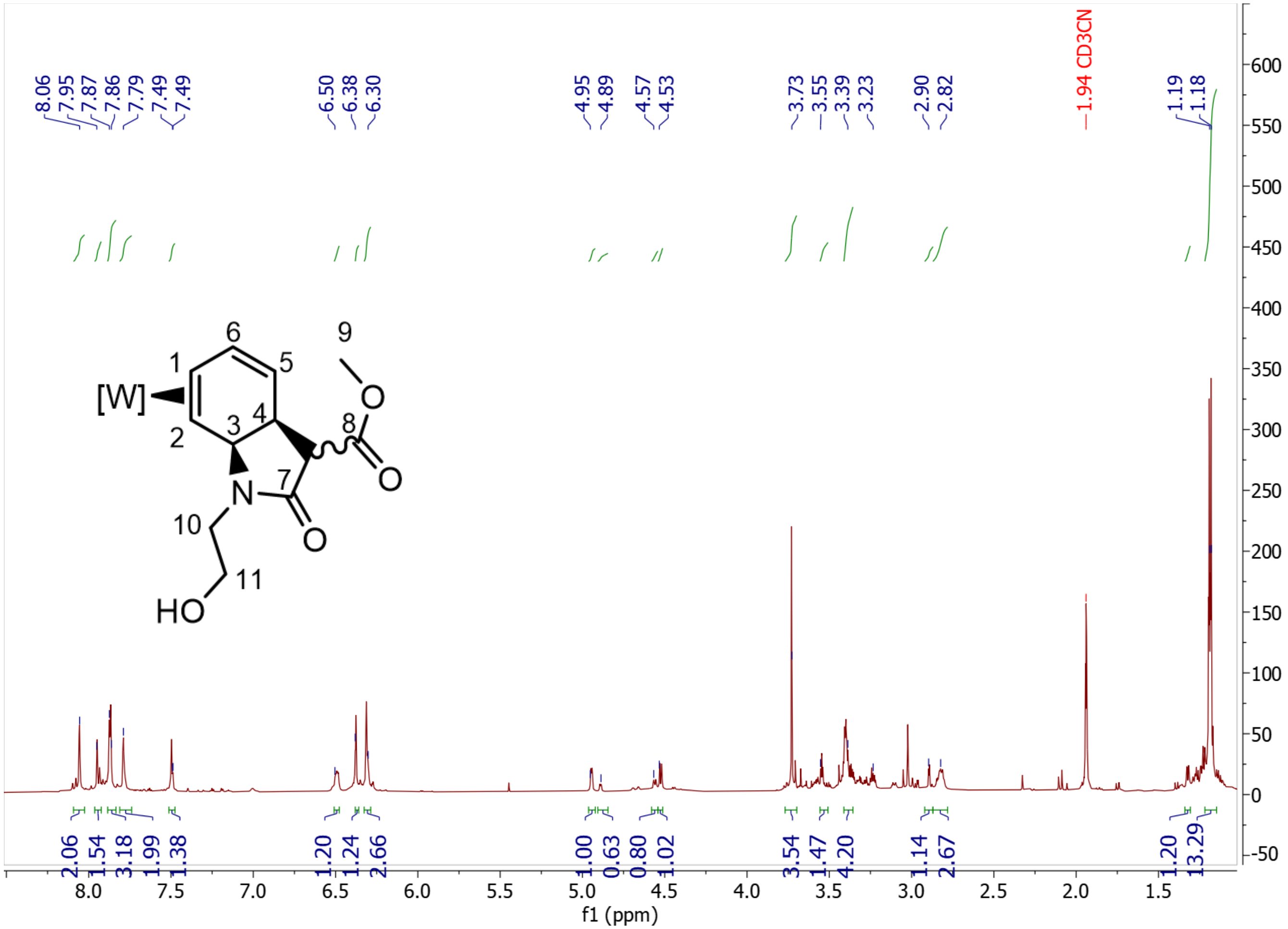

Supplementary Fig. 149: ^{1}H-NMR (CD_3CN) of Compound **69**.
Epimers are observed in the NMR spectra.

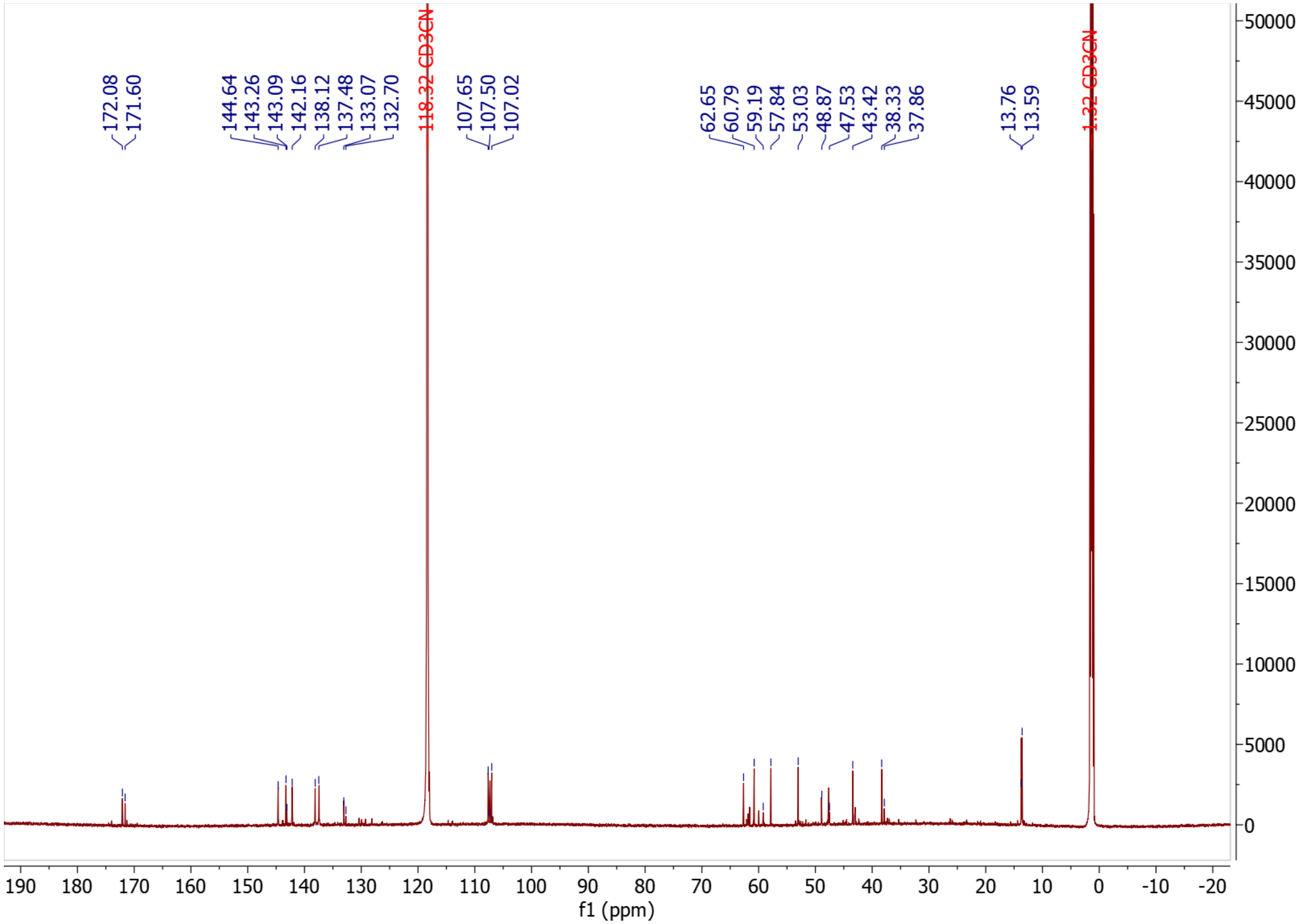

Supplementary Fig. 150: ^{13}C-NMR (CD_3CN) of Compound **69**.
Epimers are observed in the NMR spectra.

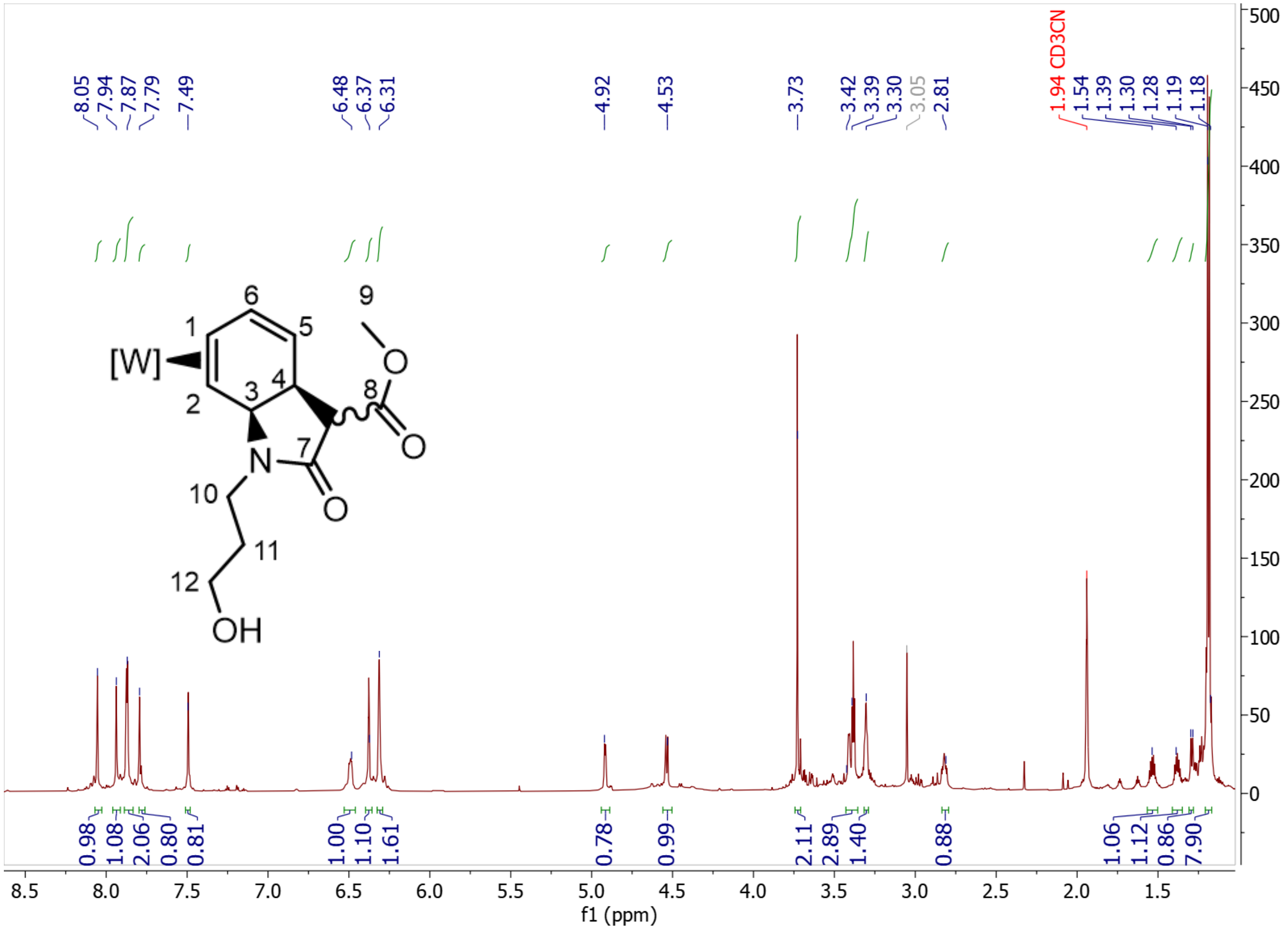

Supplementary Fig. 151: ^{1}H-NMR (CD_3CN) of Compound **70**.
Epimers are observed in the NMR spectra.

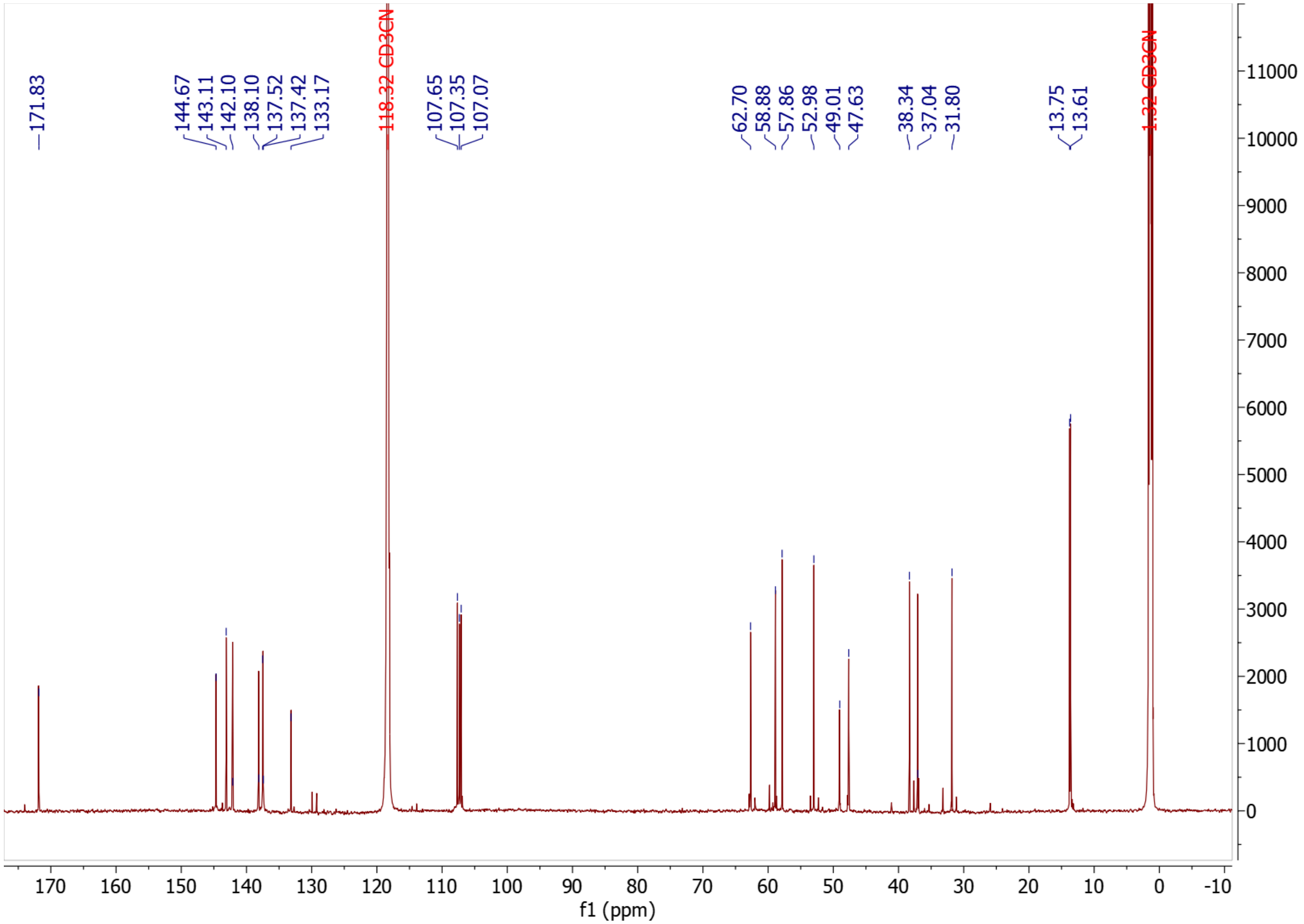

Supplementary Fig. 152: ^{13}C-NMR (CD_3CN) of Compound **70**.
Epimers are observed in the NMR spectra.

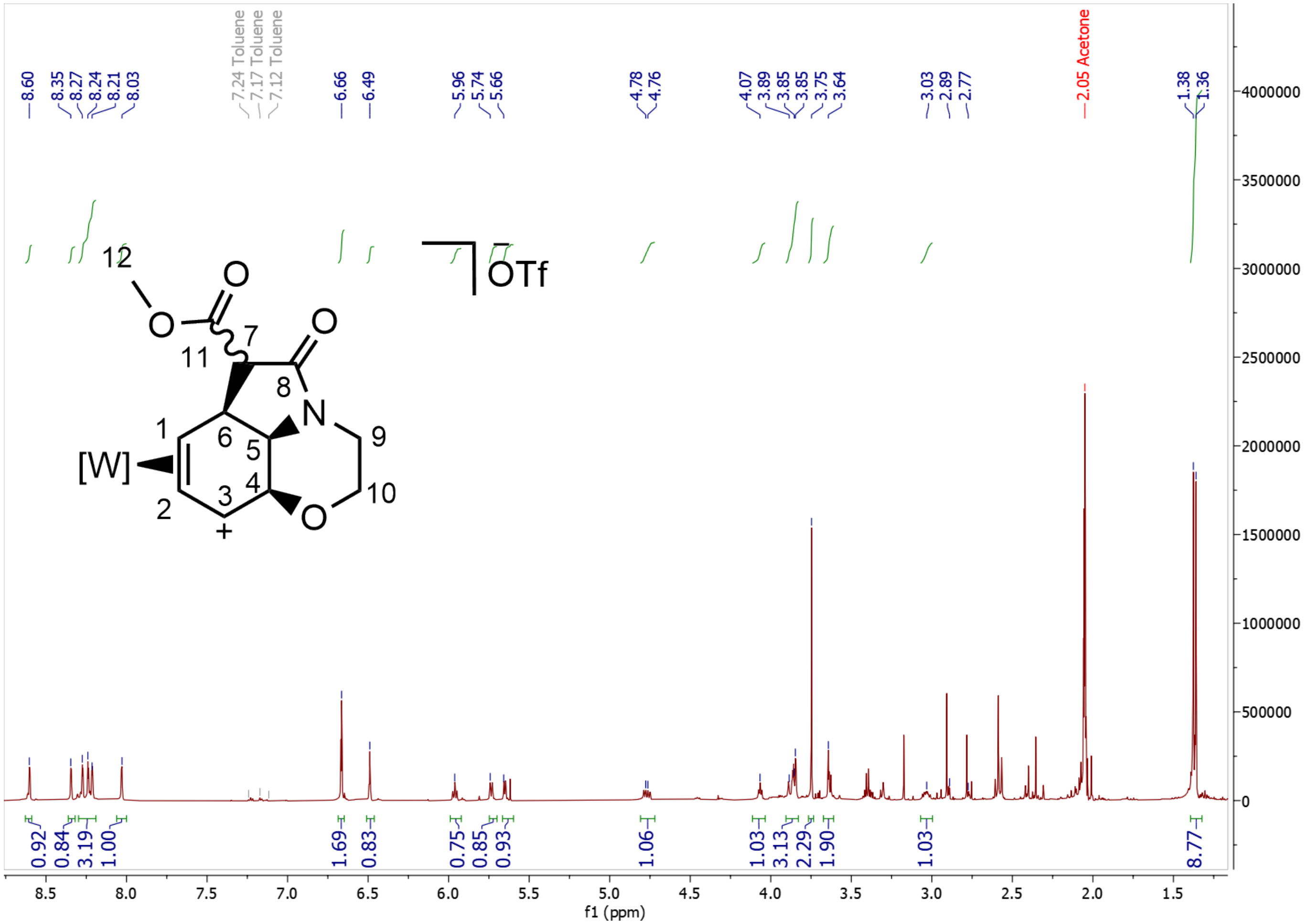

Supplementary Fig. 153: ^{1}H-NMR ($(CD_3)_2CO$) of Compound **73**.
Epimers are observed in the NMR spectra.

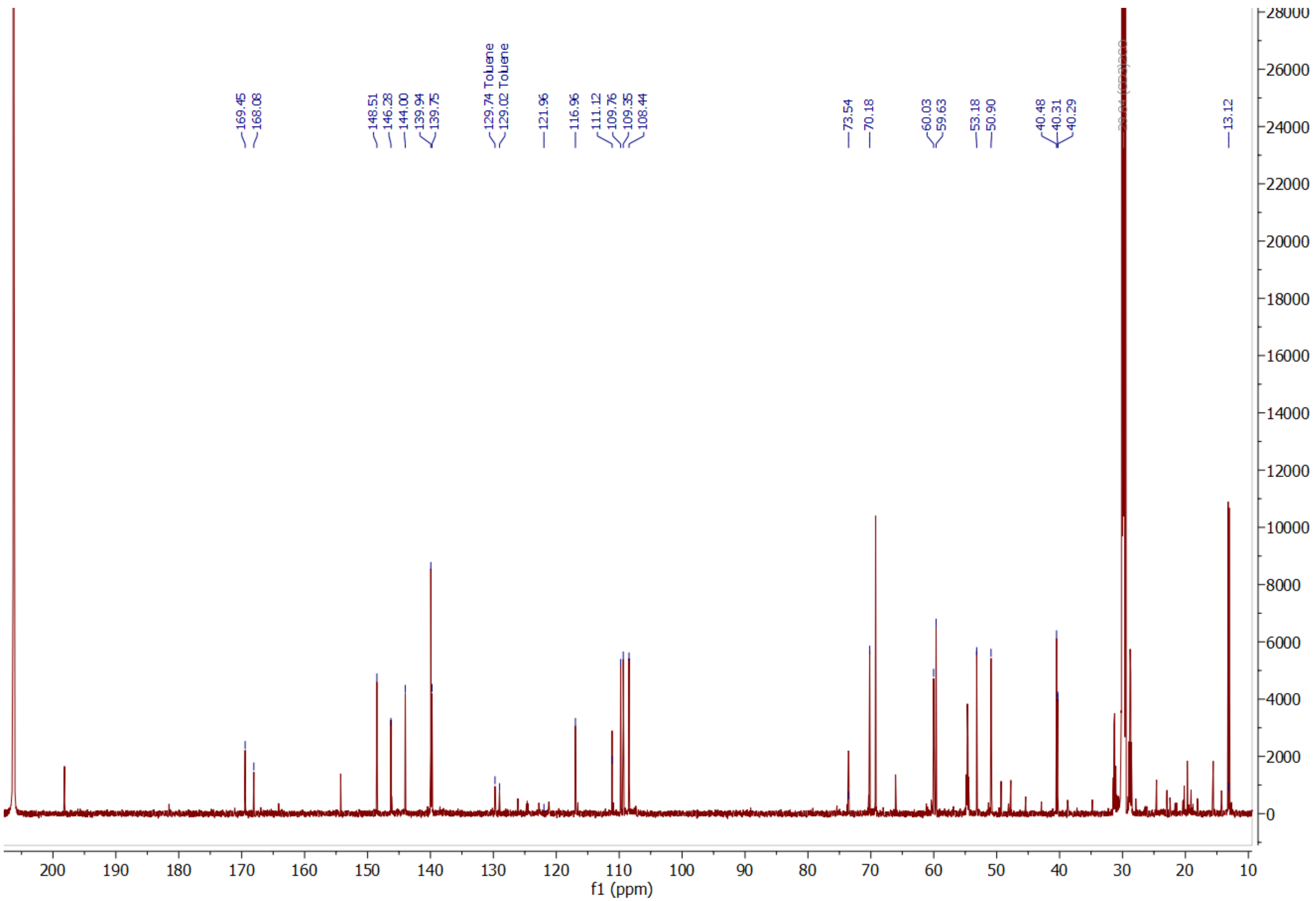

Supplementary Fig. 154: ^{13}C-NMR ($(CD_3)_2CO$) of Compound **73**.
Epimers are observed in the NMR spectra.

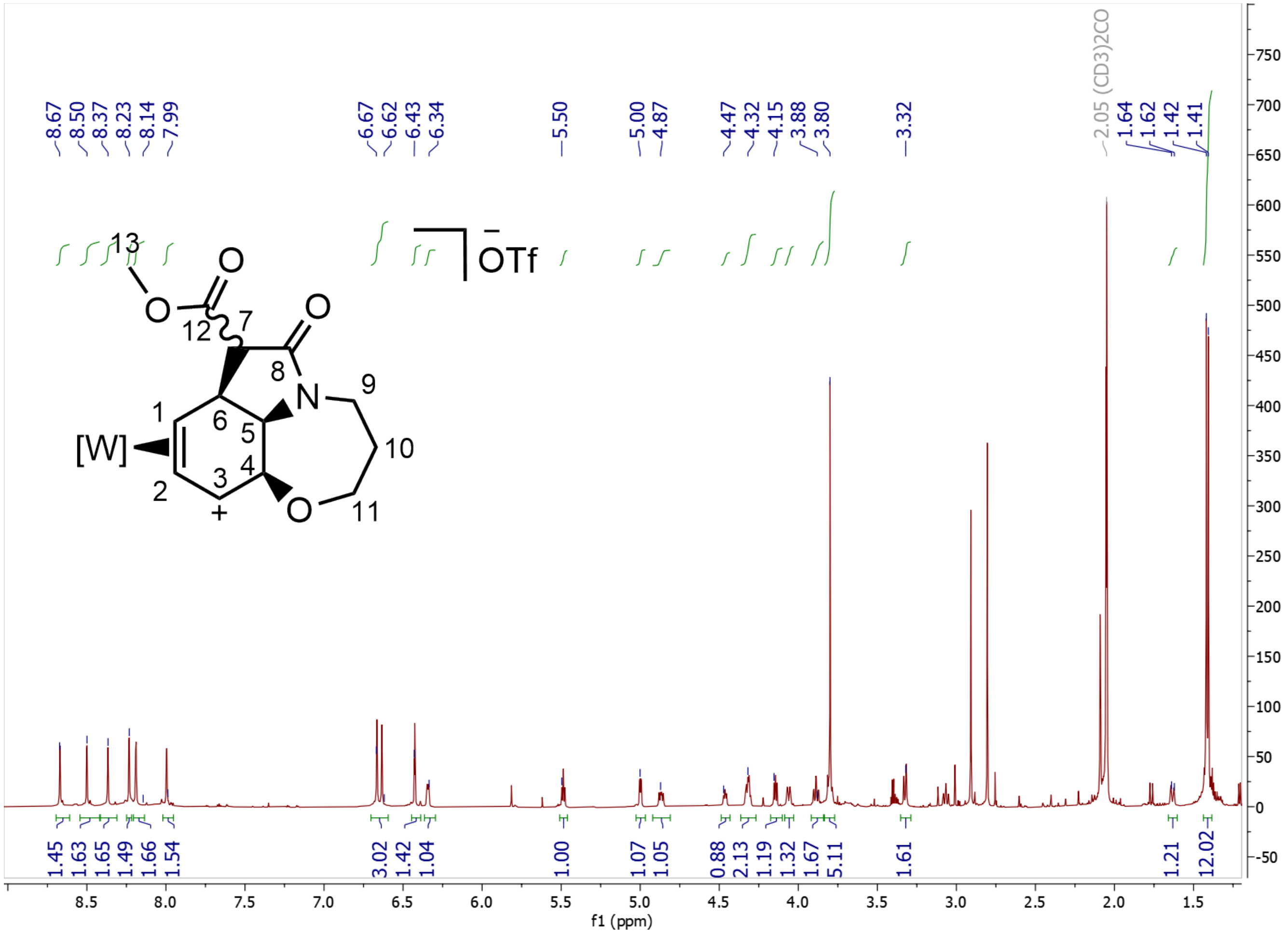

Supplementary Fig. 155: ^{1}H-NMR ($(CD_3)_2CO$) of Compound **74**.
Epimers are observed in the NMR spectra.

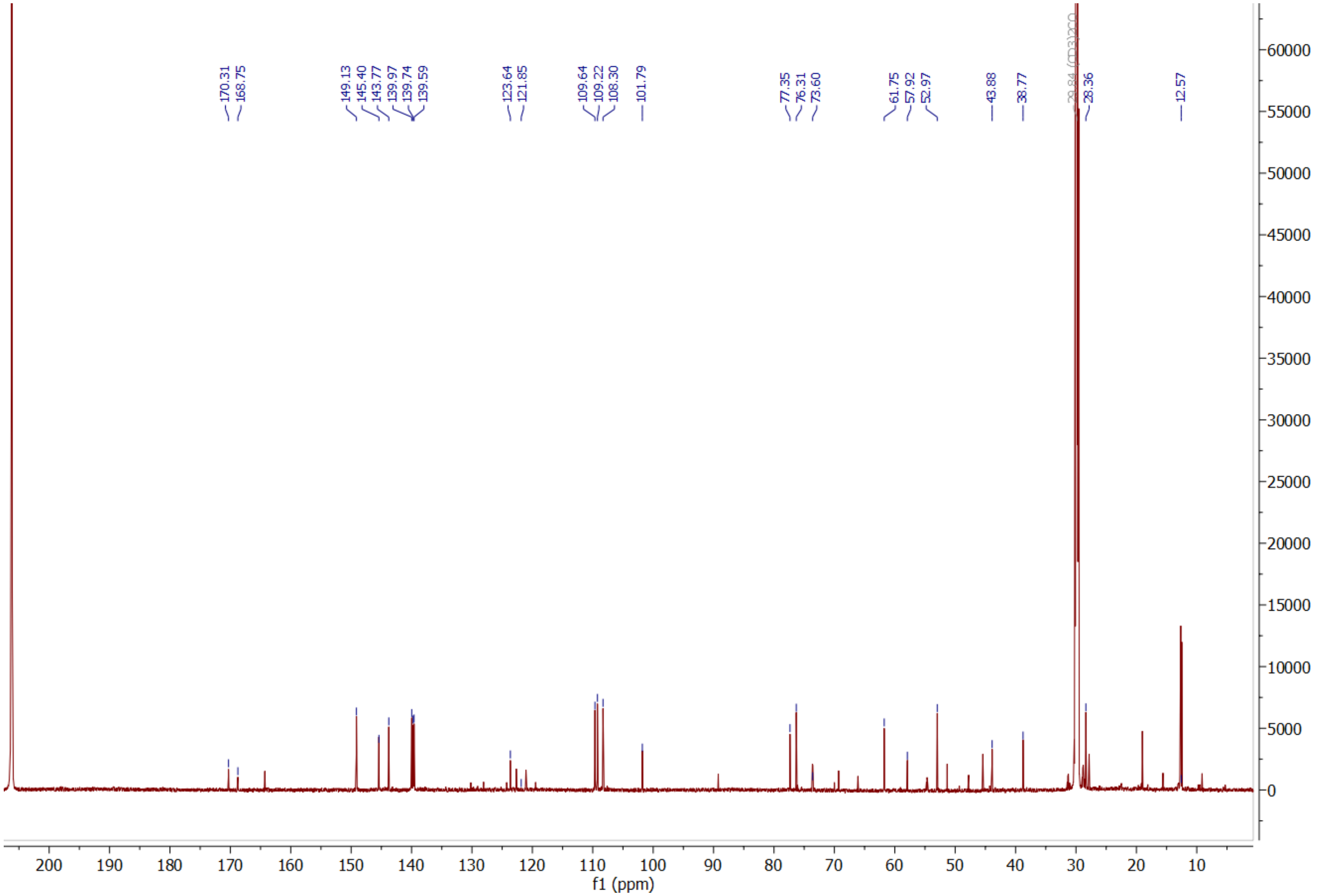

Supplementary Fig. 156: ^{13}C-NMR ($(CD_3)_2CO$) of Compound **74**.
Epimers are observed in the NMR spectra.

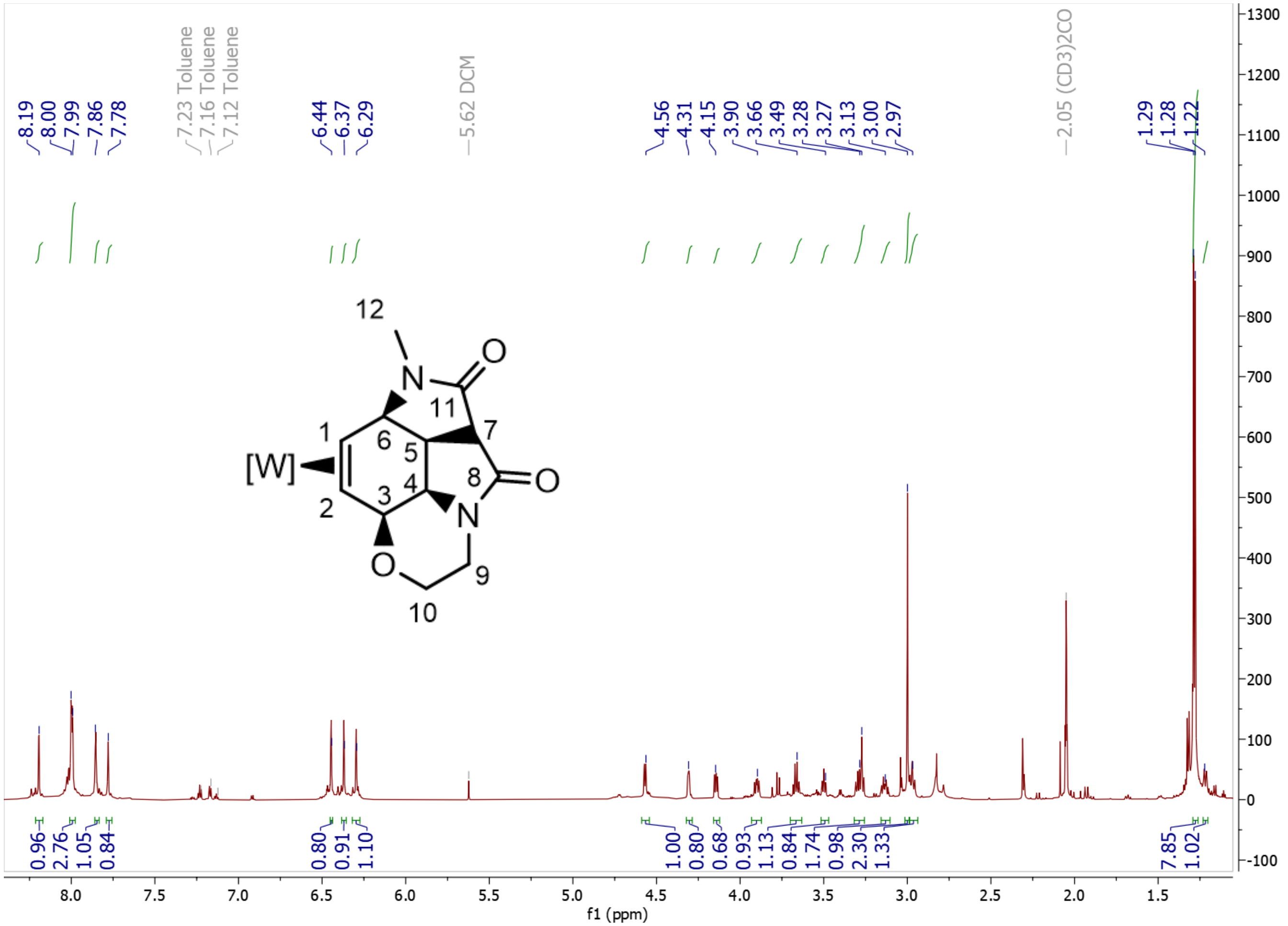

Supplementary Fig. 157: ^{1}H-NMR ($(CD_3)_2CO$) of Compound **75**.

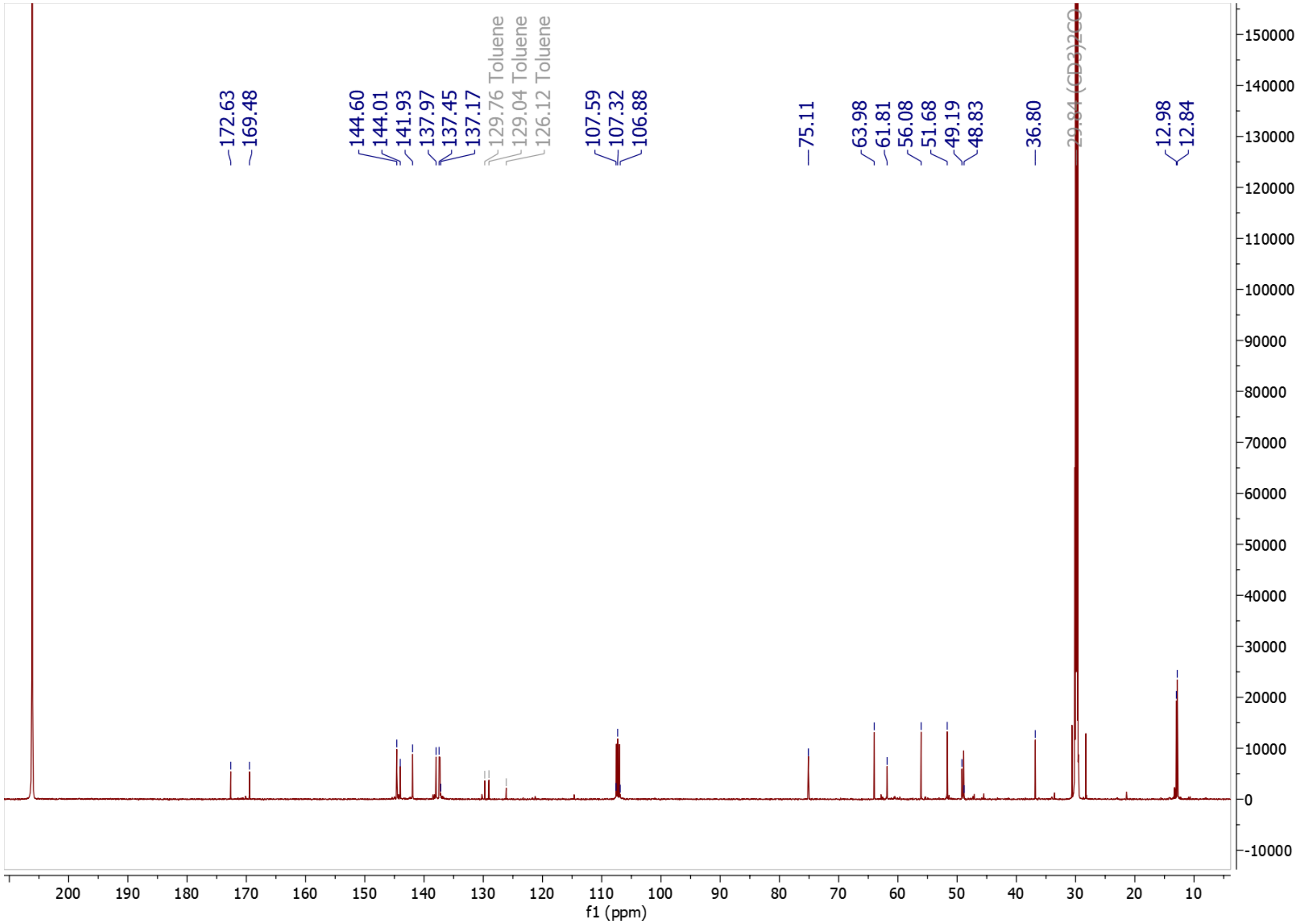

Supplementary Fig. 158: ^{1}H-NMR ($(CD_3)_2CO$) of Compound **75**.

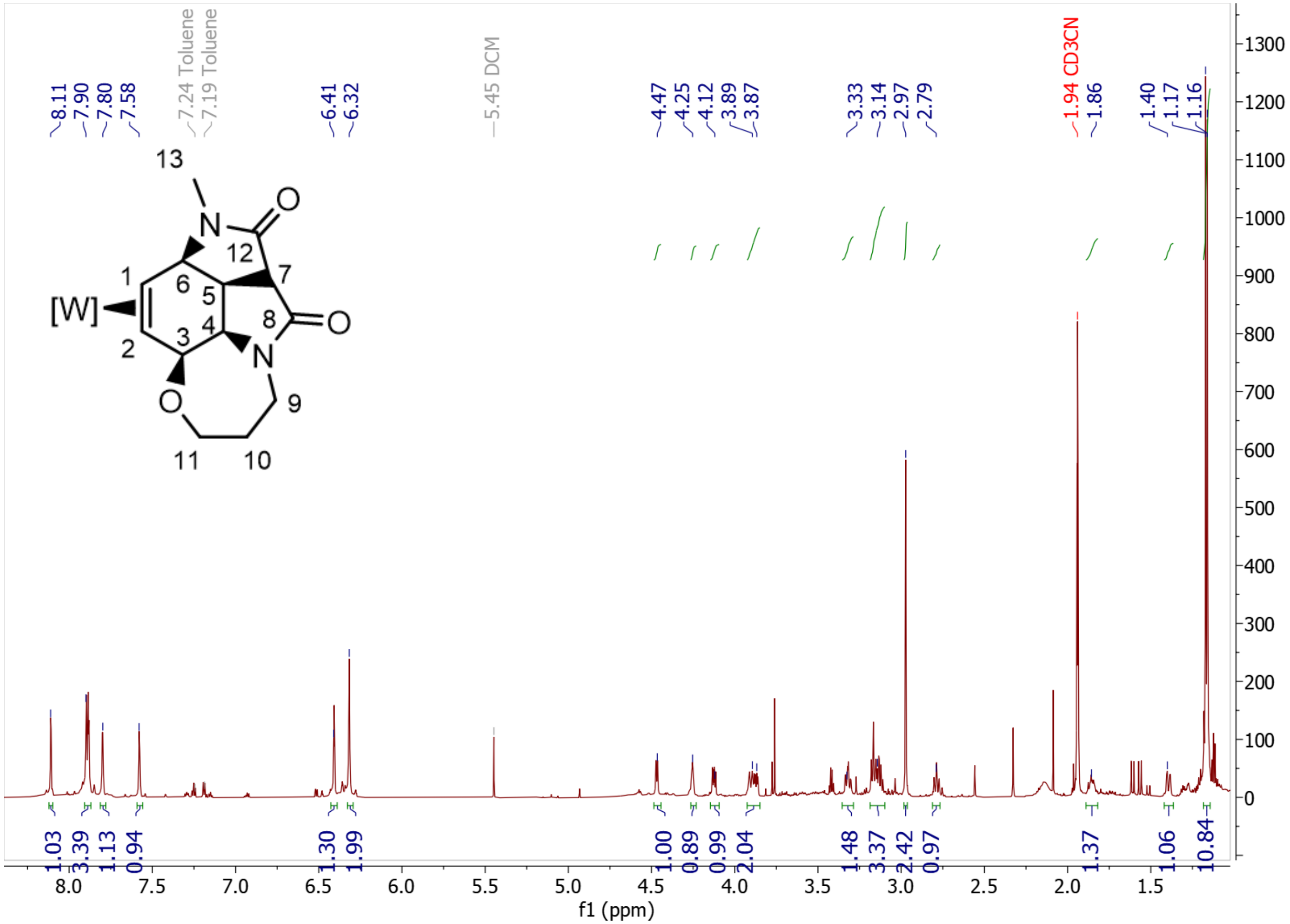

Supplementary Fig. 159: ^{1}H-NMR (CD_3CN) of Compound **76**.

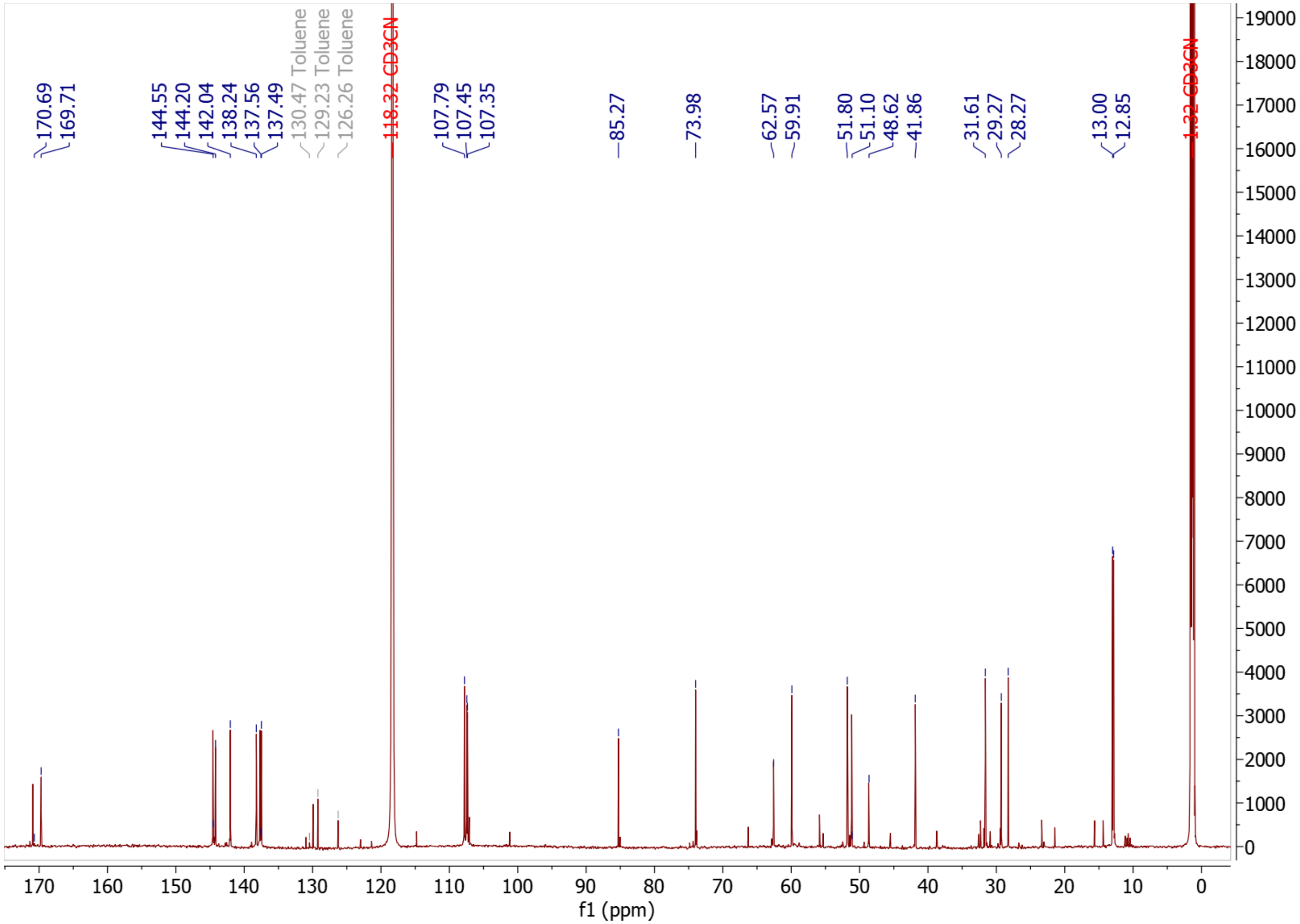

Supplementary Fig. 160: ^{13}C-NMR (CD_3CN) of Compound **76**.

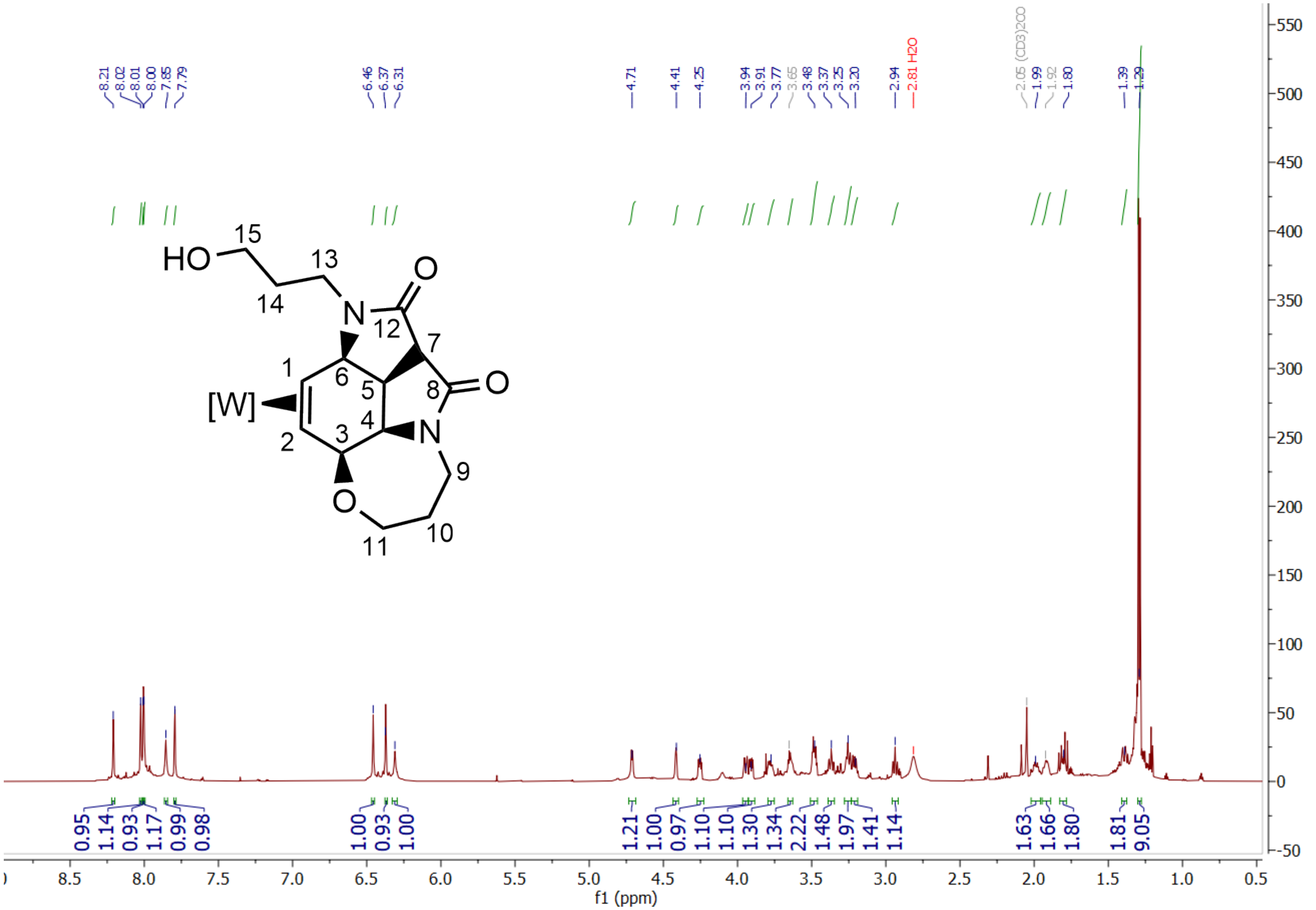

Supplementary Fig. 161: ^{1}H-NMR (CD_3CN) of Compound **77**.

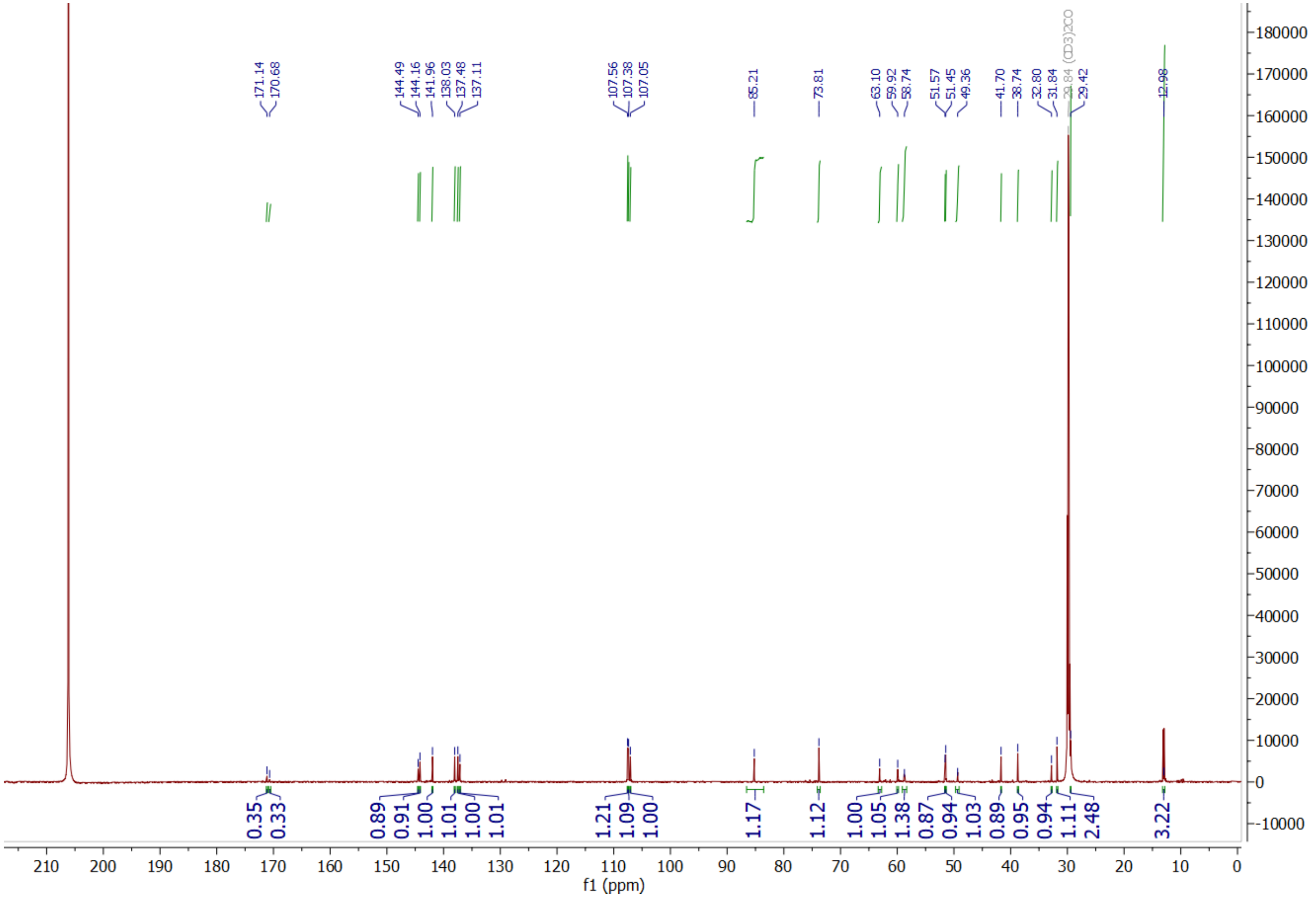

Supplementary Fig. 162: ^{13}C-NMR (CD_3CN) of Compound **77**.

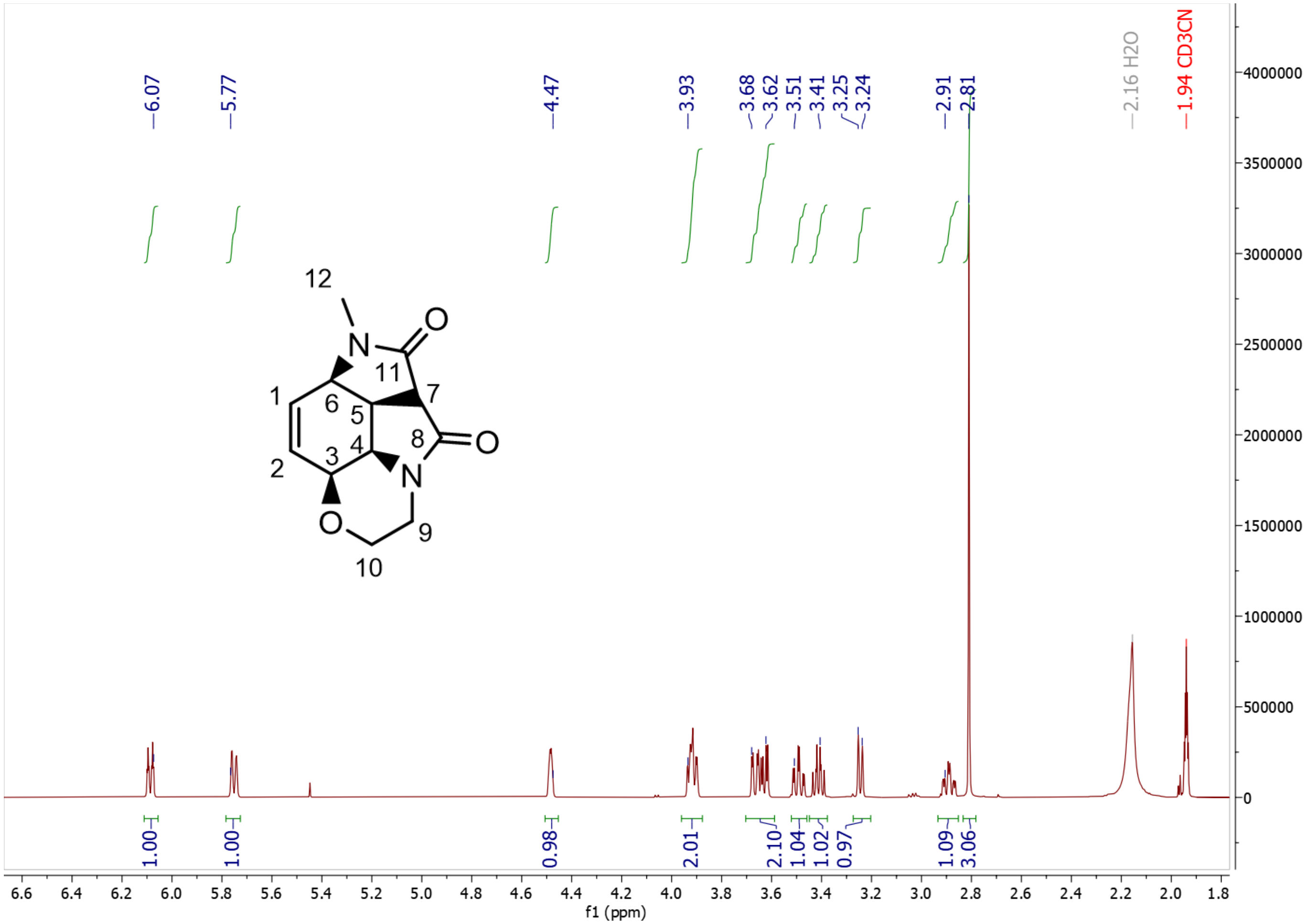

Supplementary Fig. 163: ^{1}H-NMR (CD_3CN) of Compound **78**.

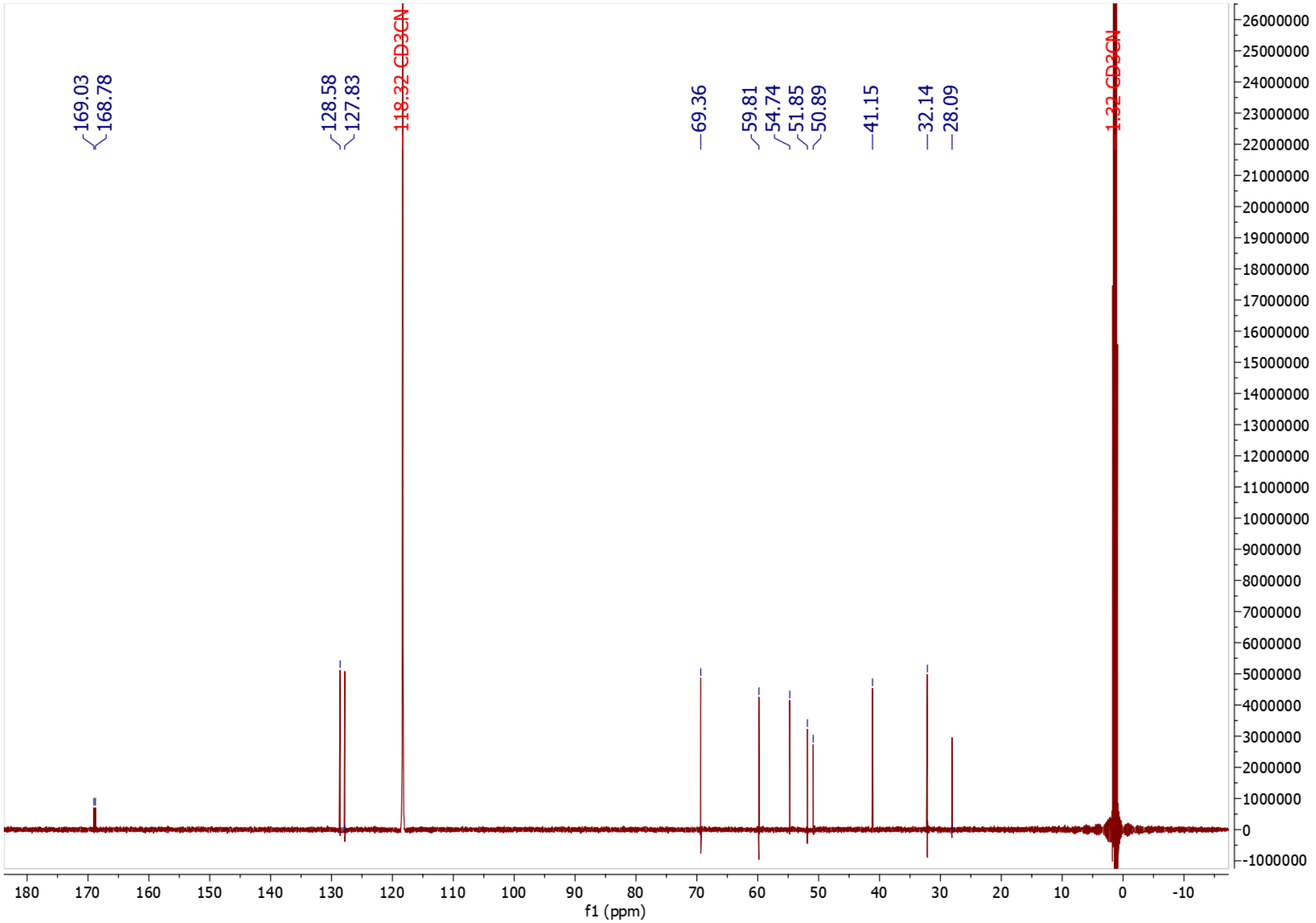

Supplementary Fig. 164: ^{13}C-NMR (CD_3CN) of Compound **78**.

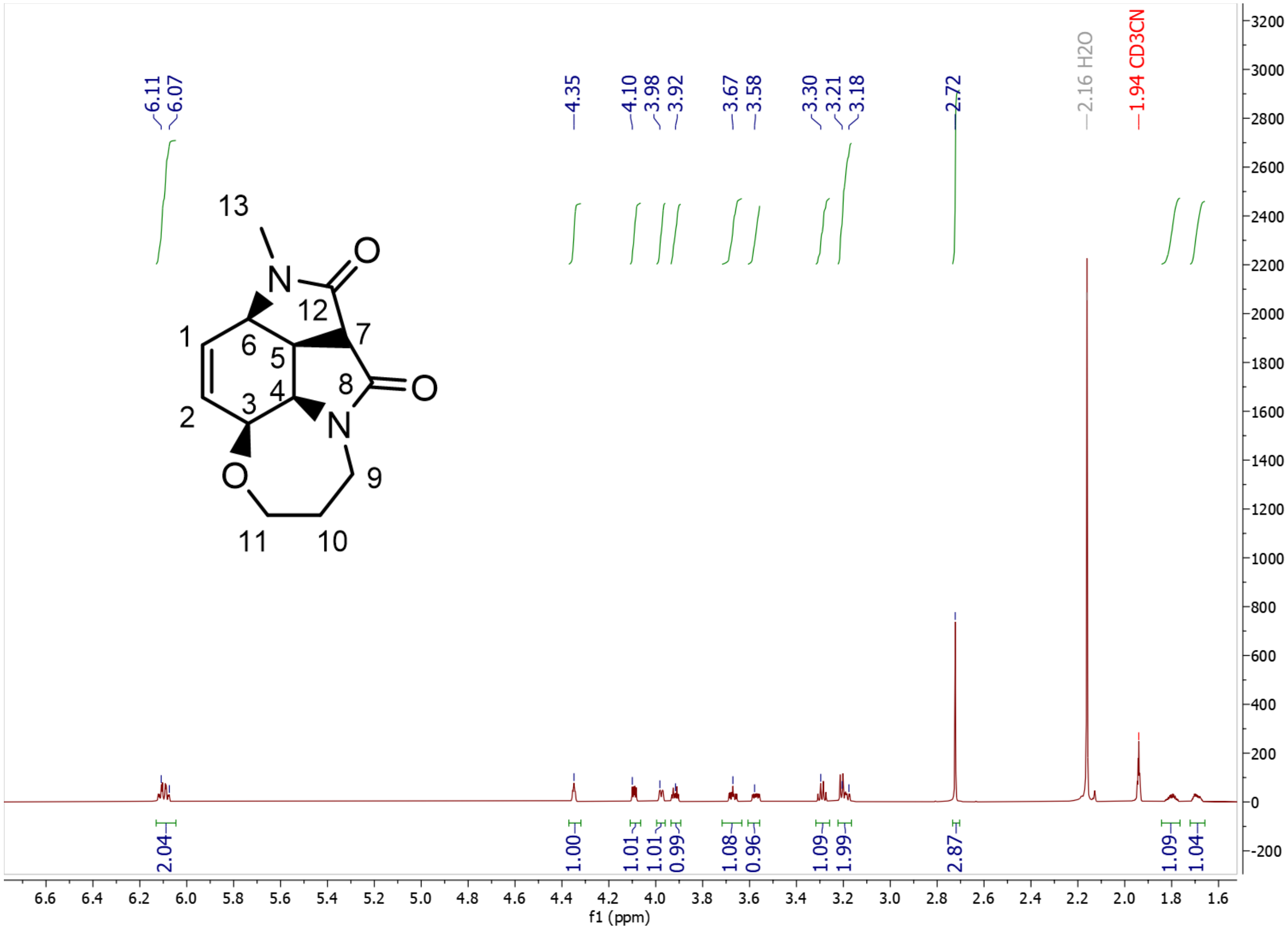

Supplementary Fig. 165: ^{1}H-NMR (CD_3CN) of Compound **79**.

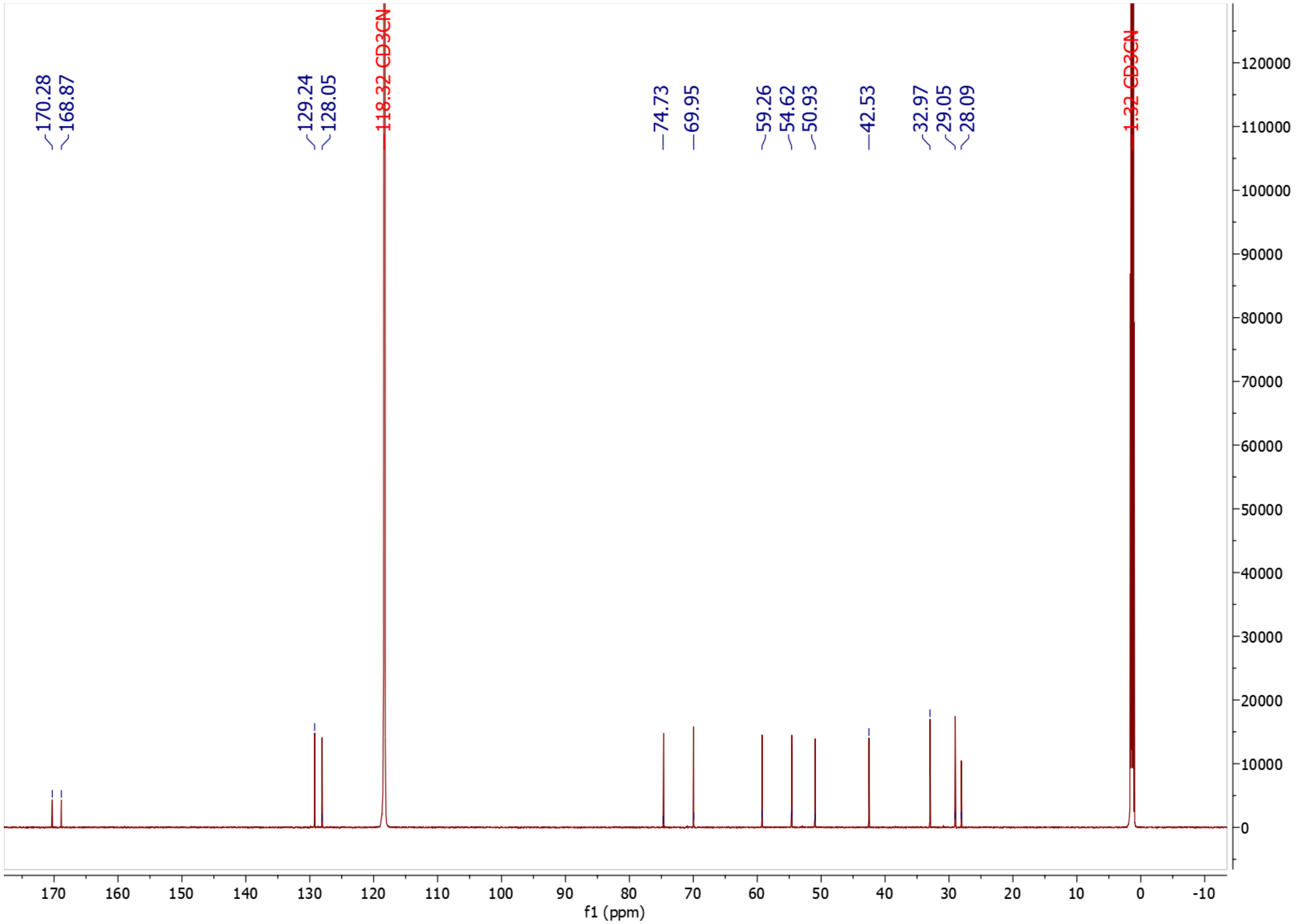

Supplementary Fig. 166: ^{13}C-NMR (CD_3CN) of Compound **79**.

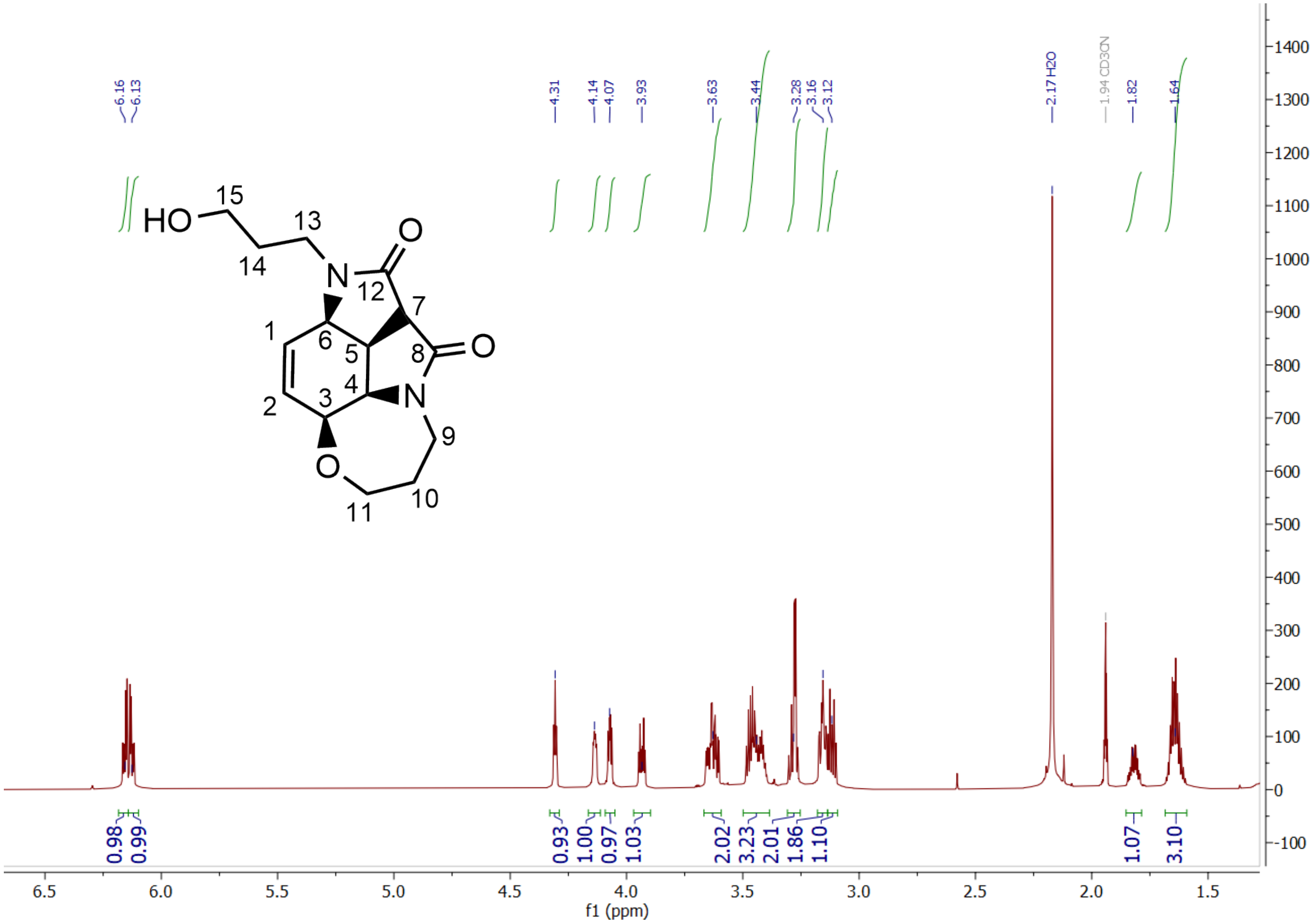

Supplementary Fig. 167: ^{1}H-NMR (CD_3CN) of Compound **80**.

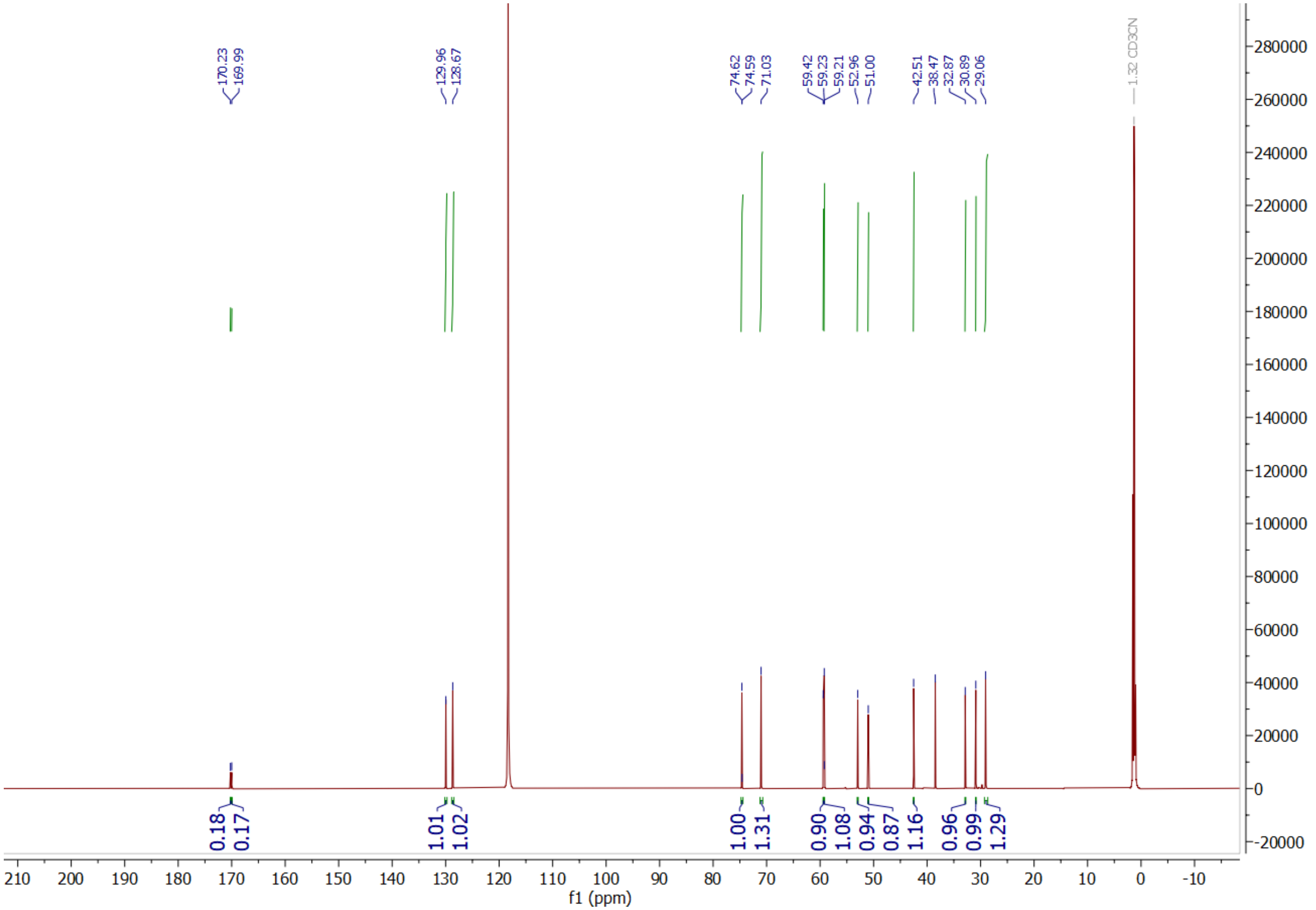

Supplementary Fig. 168: ^{13}C-NMR (CD_3CN) of Compound **80**.

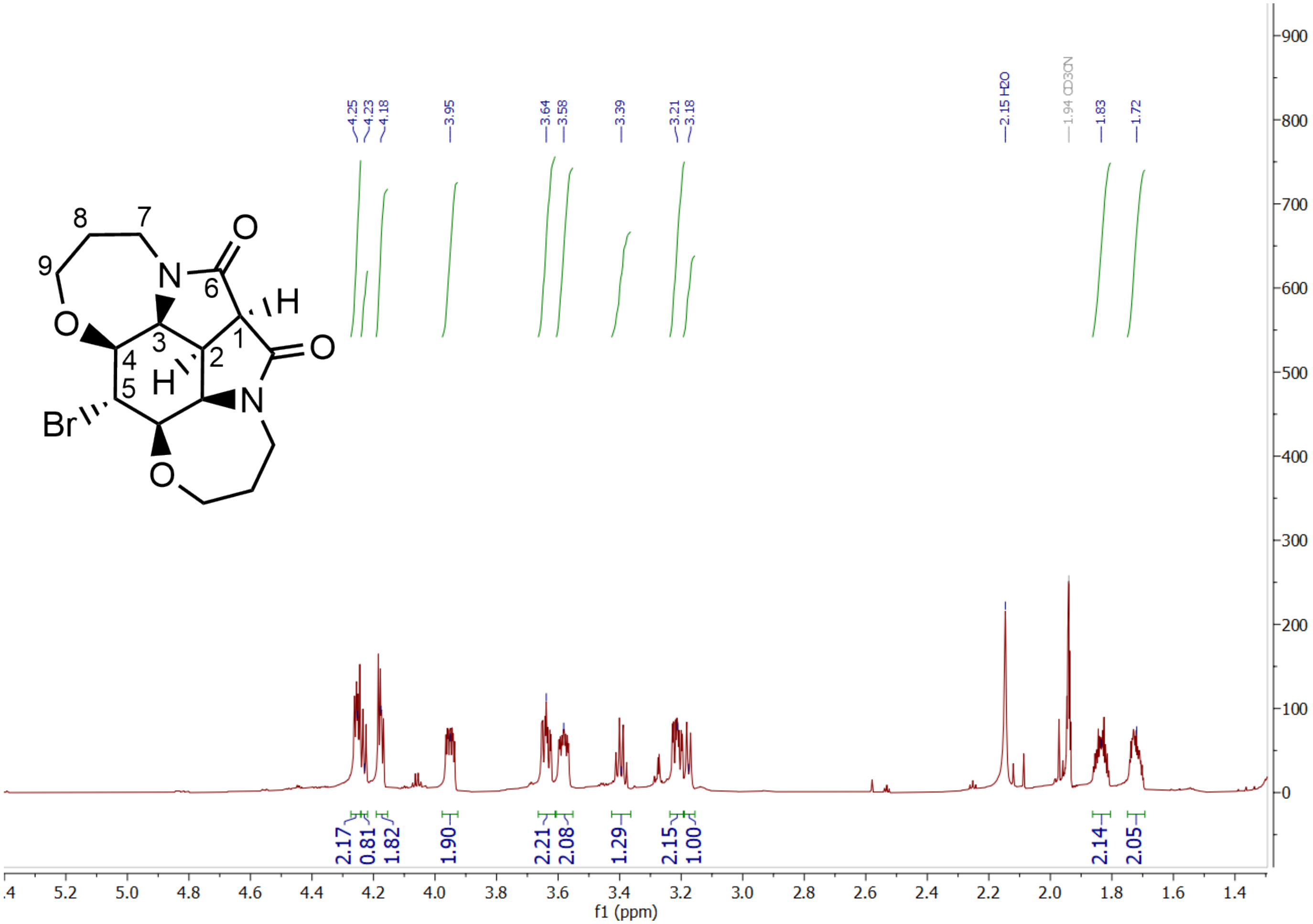

Supplementary Fig. 169: ^{1}H-NMR (CD_3CN) of Compound **81**.

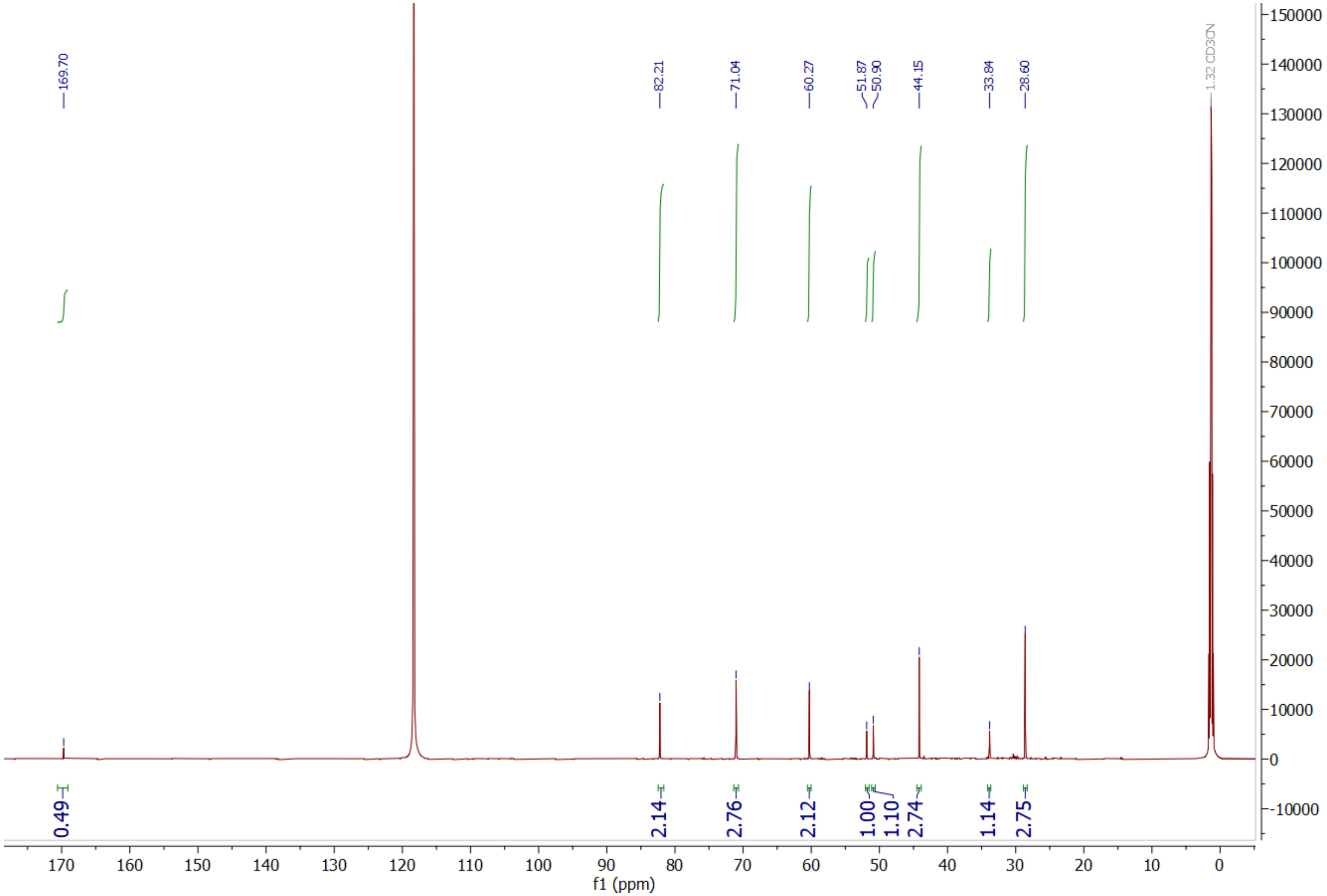

Supplementary Fig. 170: ^{13}C-NMR (CD_3CN) of Compound **81**.

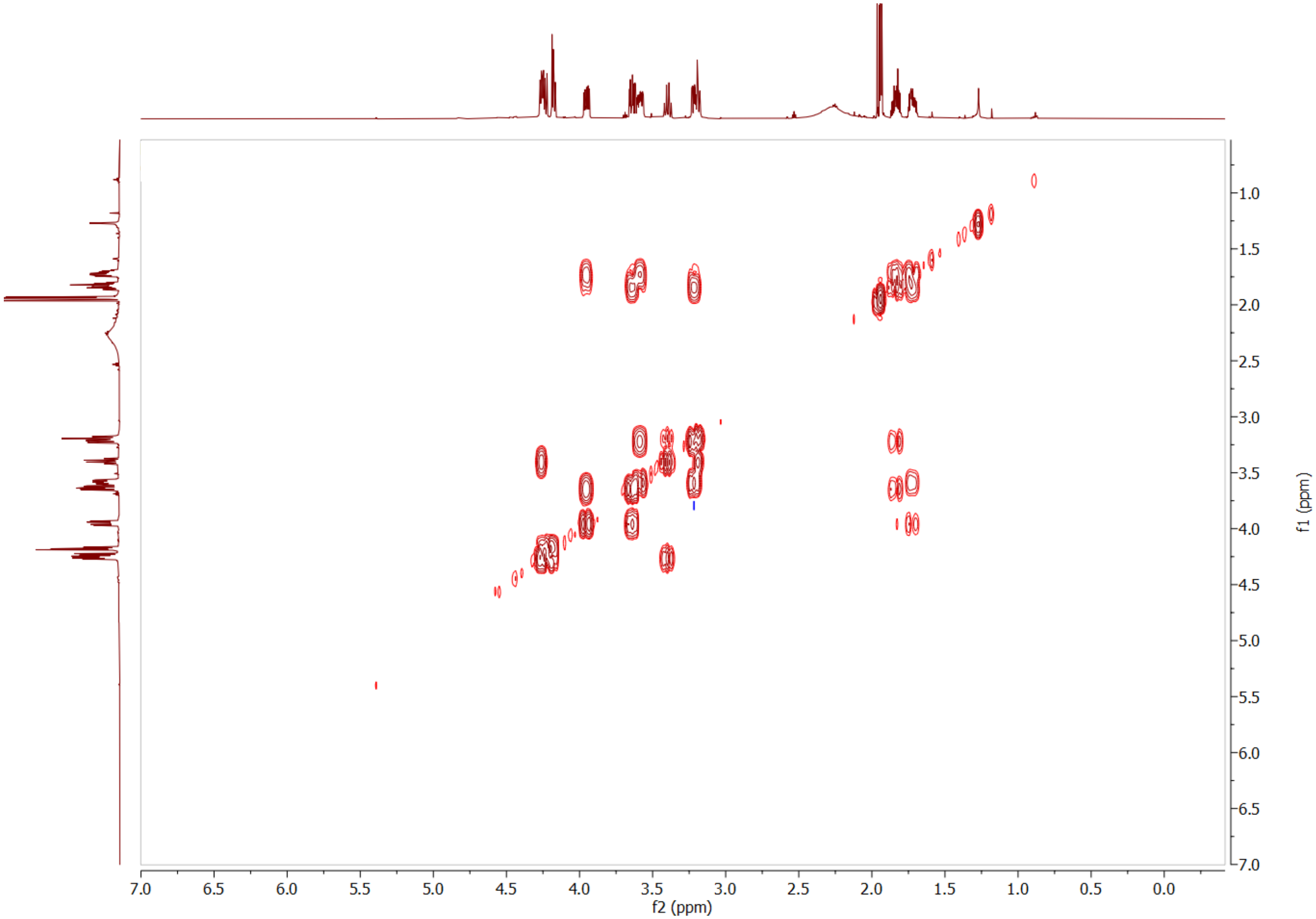

Supplementary Fig. 171: ^{1}H-1H COSY (CD_3CN) of Compound **81**.

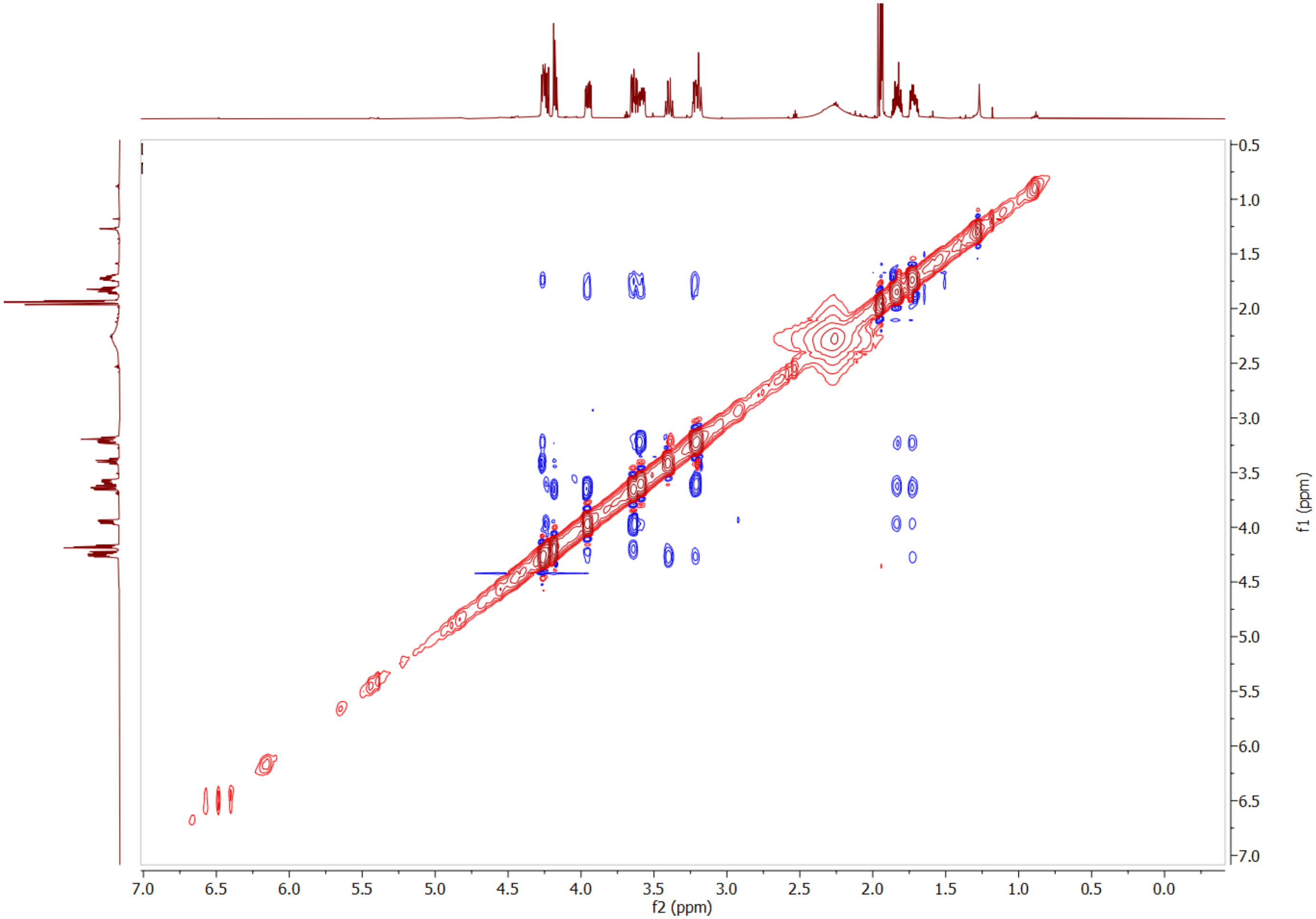

Supplementary Fig. 172: ^{1}H-^{1}H NOESY (CD_3CN) of Compound **81**.

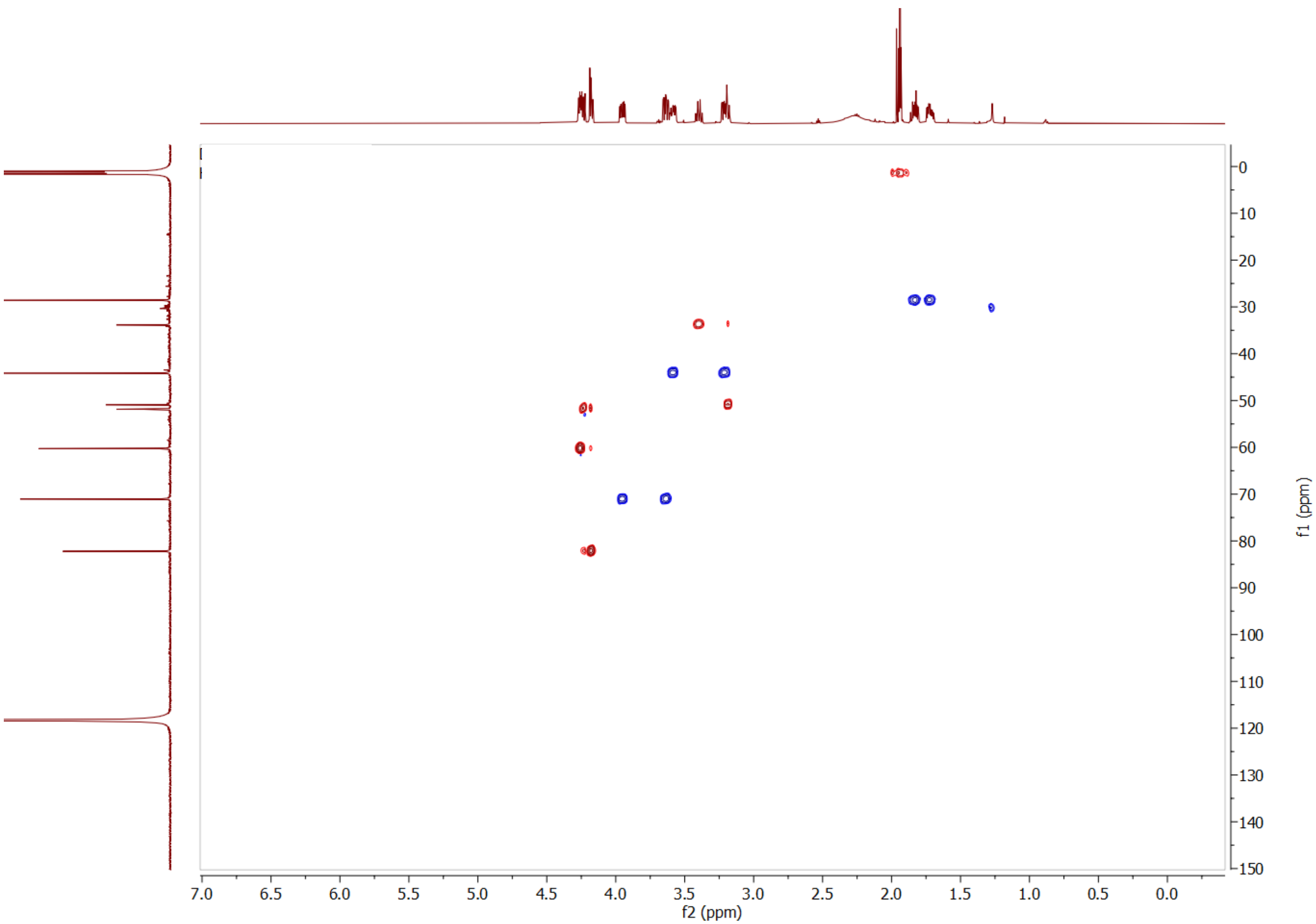

Supplementary Fig. 173: 1H-^{13}C HSQC (CD_3CN) of Compound **81**.

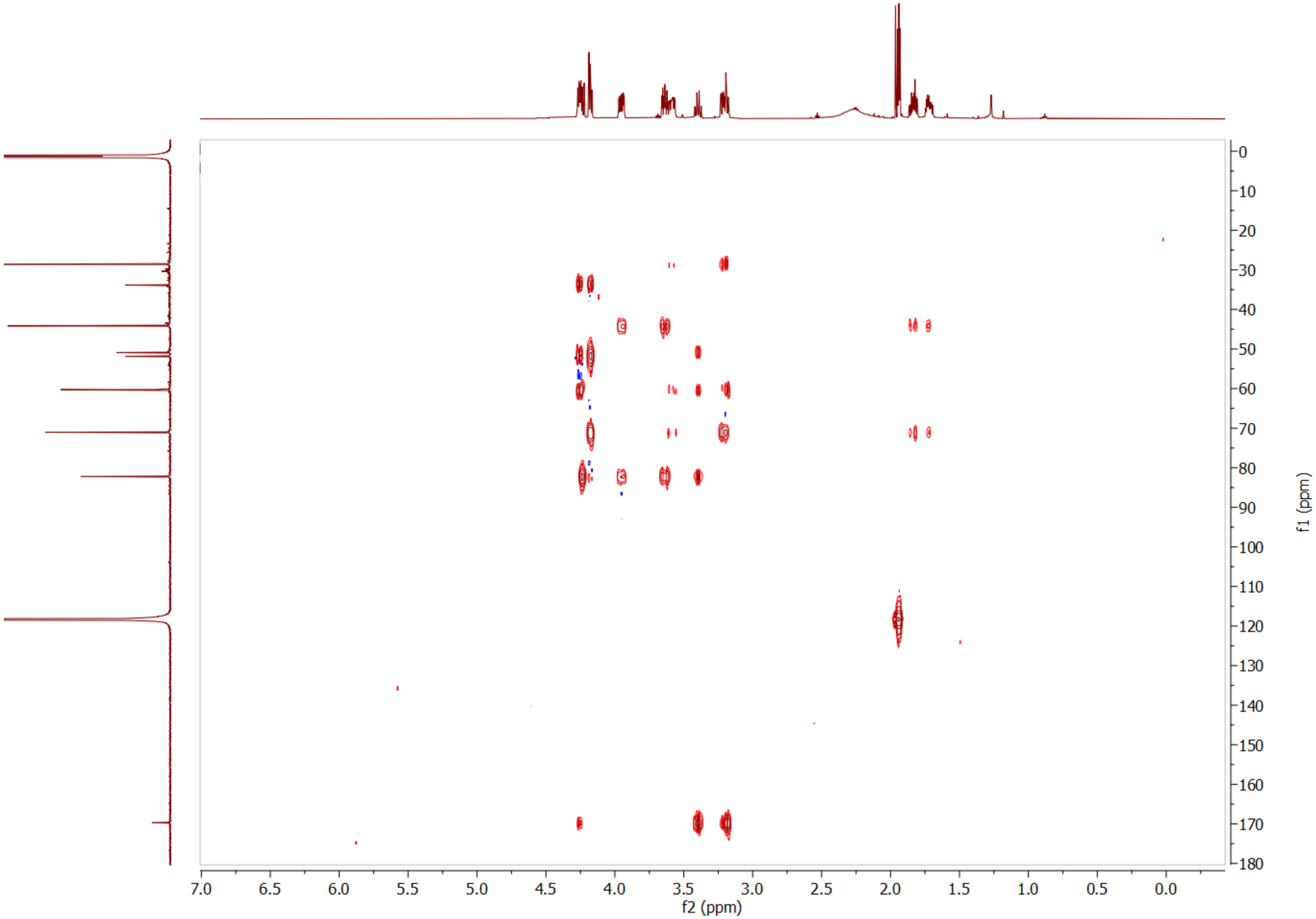

Supplementary Fig. 174: 1H-^{13}C HMBC (CD_3CN) of Compound **81**.

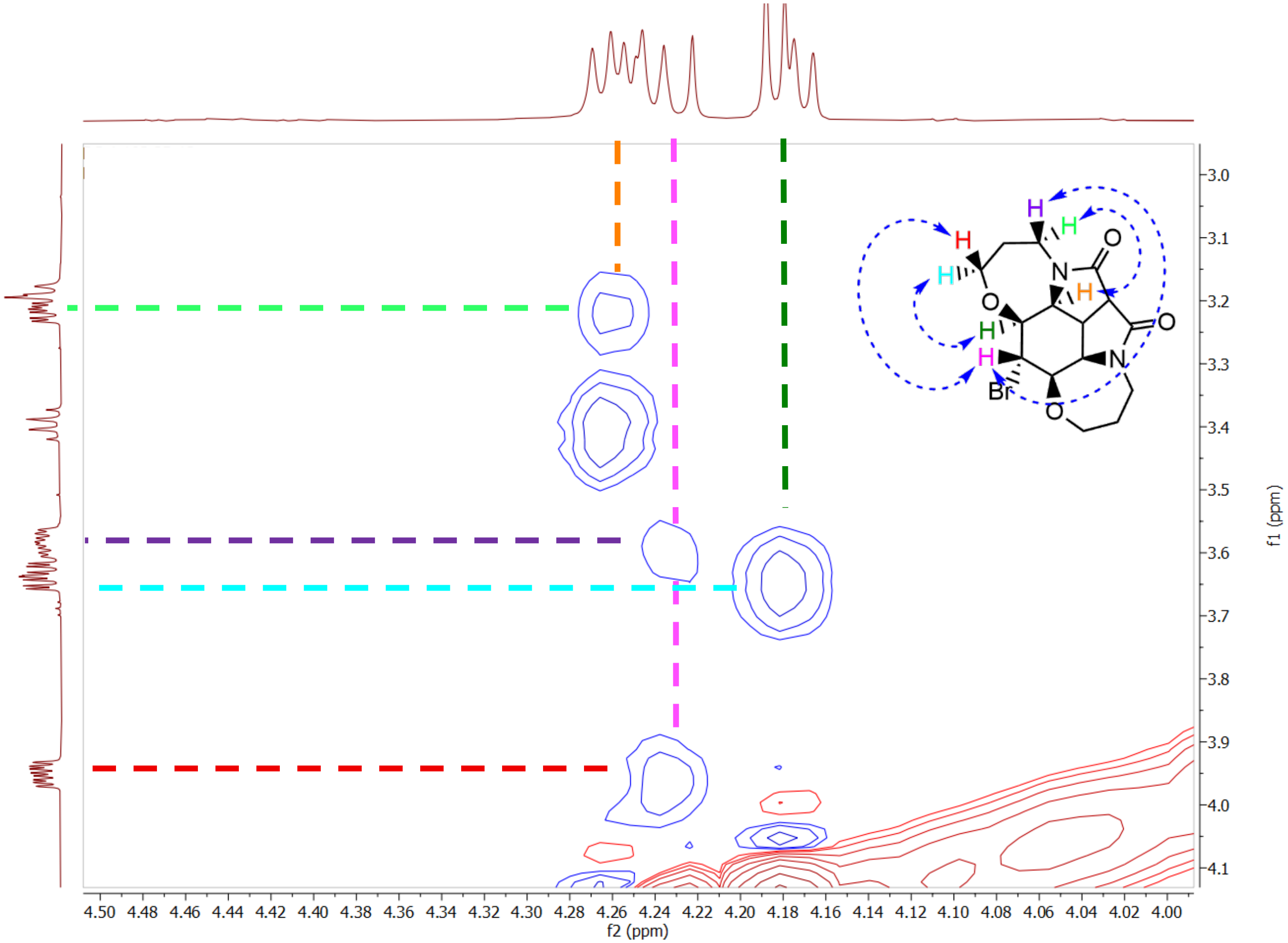

Supplementary Fig. 175: Key NOE interactions (CD_3CN) of Compound **81**.

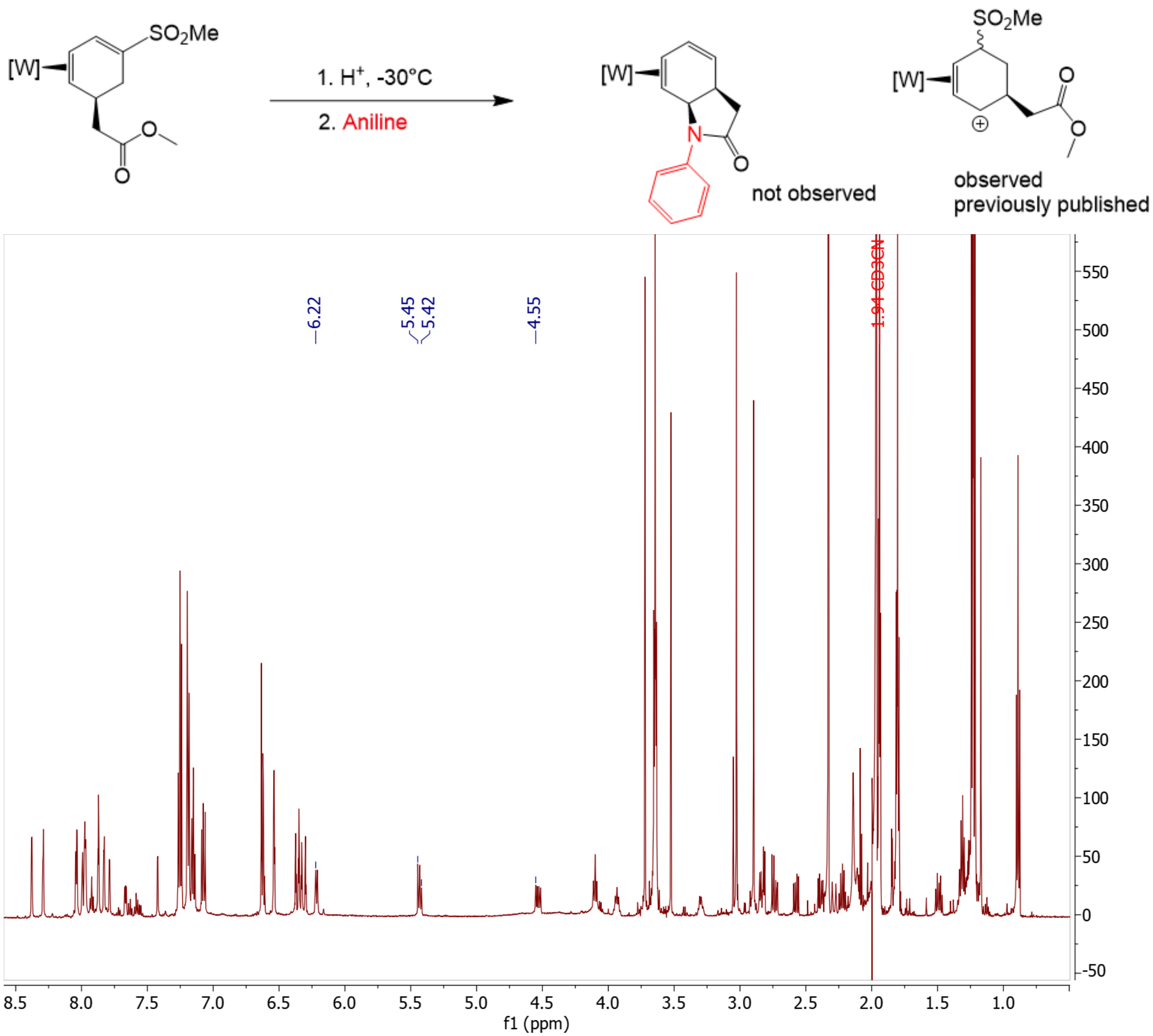

Supplementary Fig. 176: ^{1}H-NMR (CD_3CN) Reaction of 2 with aniline (Compound **82**).

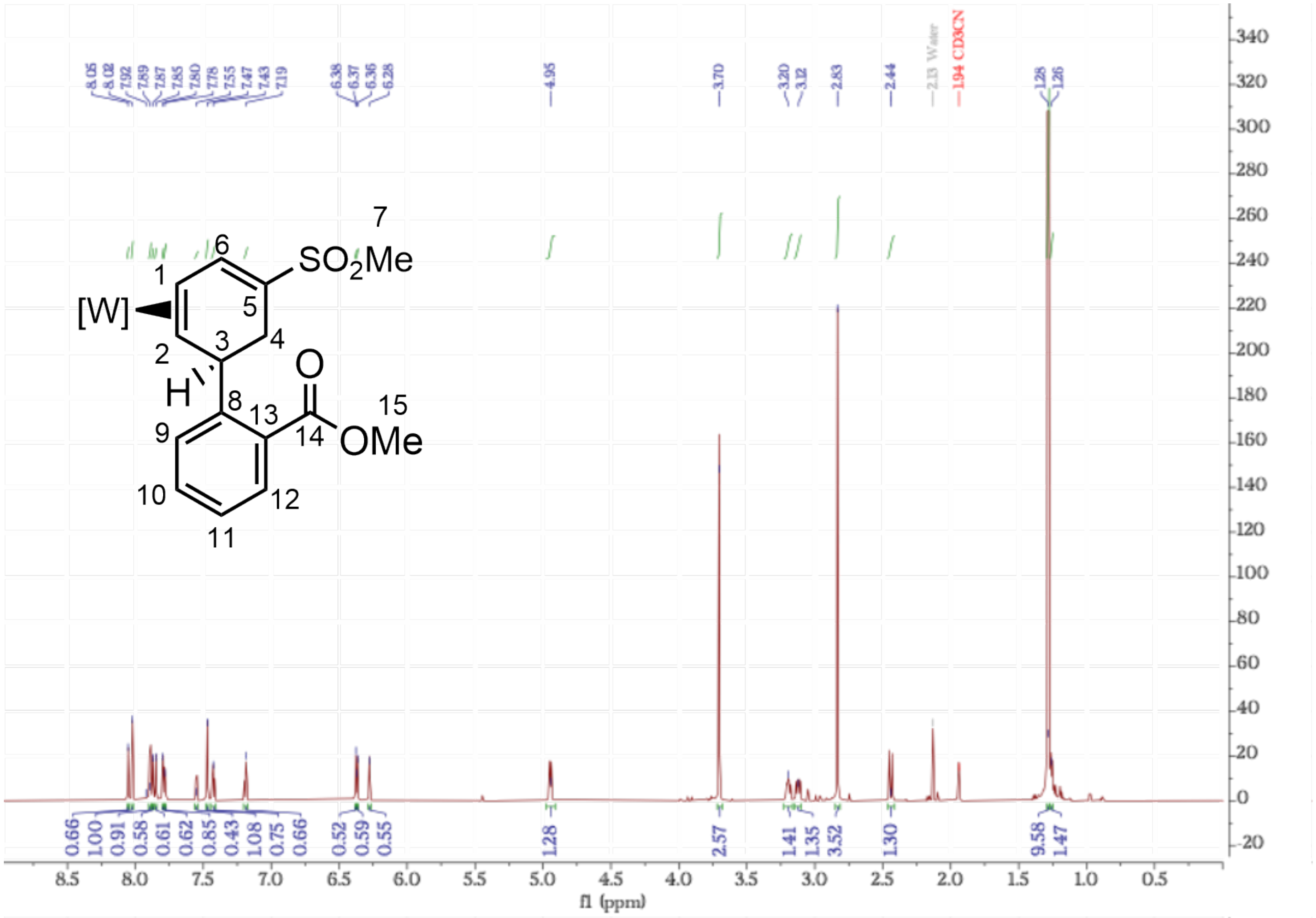

Supplementary Fig. 177: ^{1}H-NMR (CD_3CN) of Compound **83**.

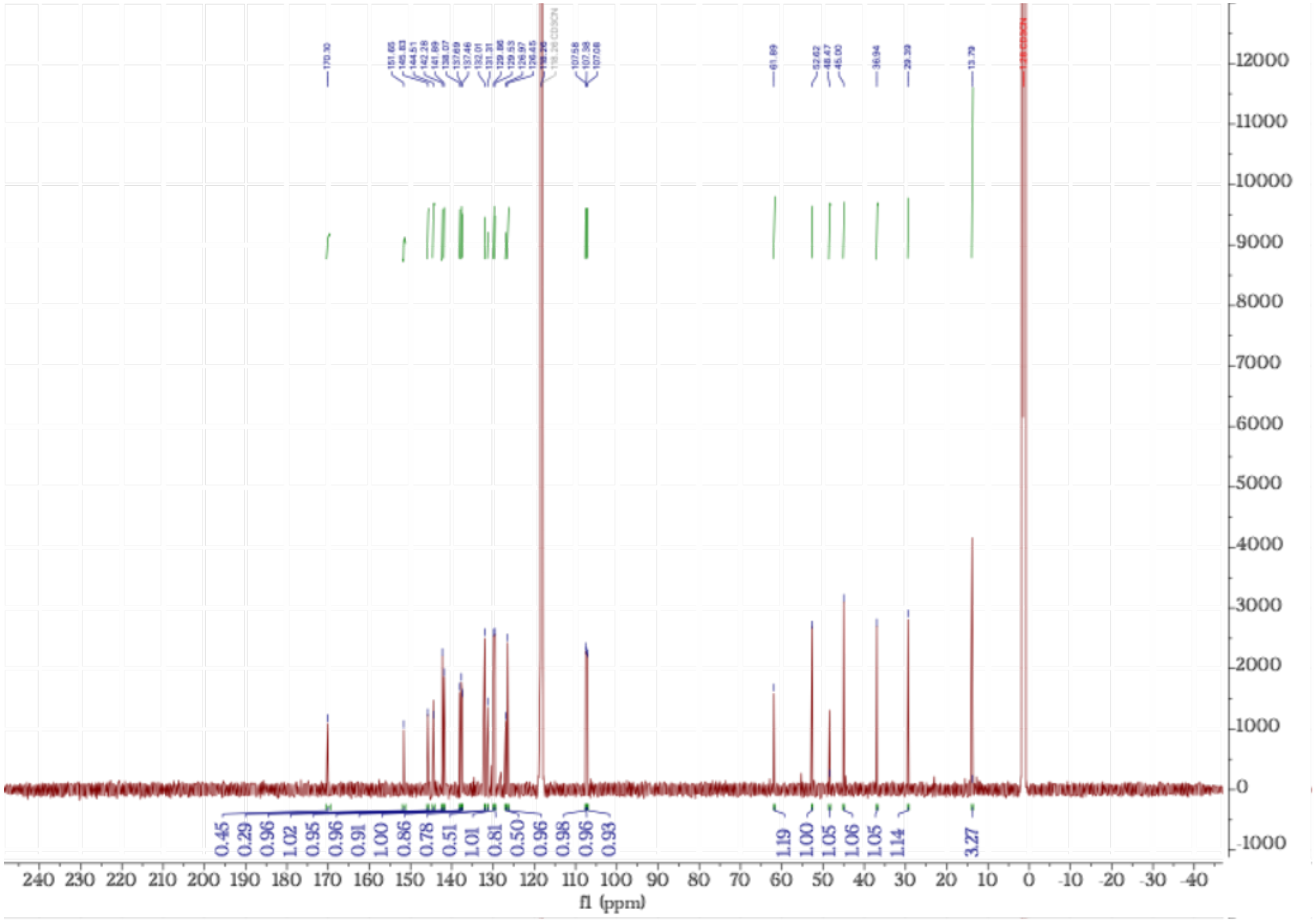

Supplementary Fig. 178: ^{13}C-NMR (CD_3CN) of Compound **83**.

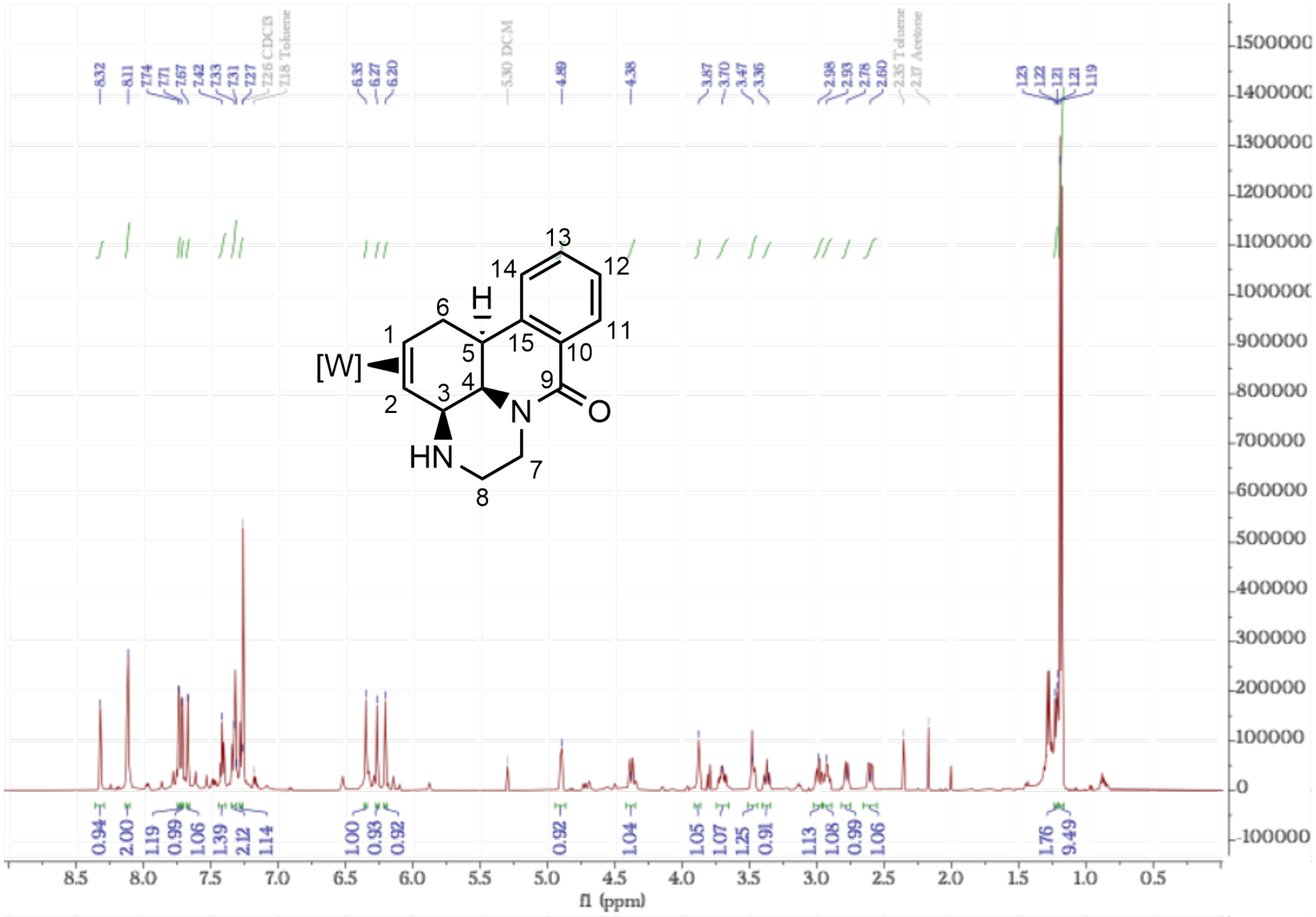

Supplementary Fig. 179: ^{1}H-NMR ($CDCl_3$) of Compound **84**.

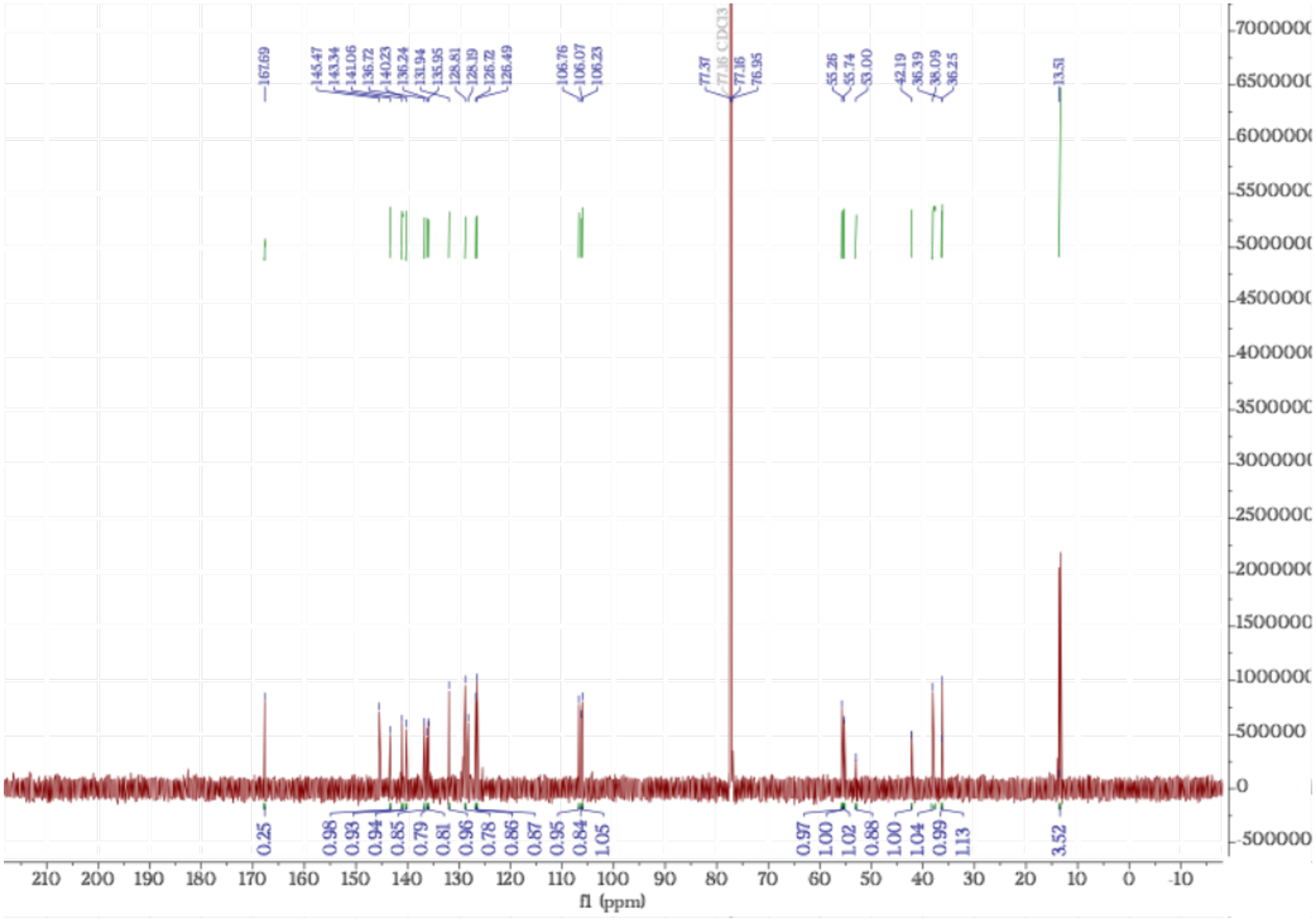

Supplementary Fig. 180: ^{13}C-NMR ($CDCl_3$) of Compound **84**.

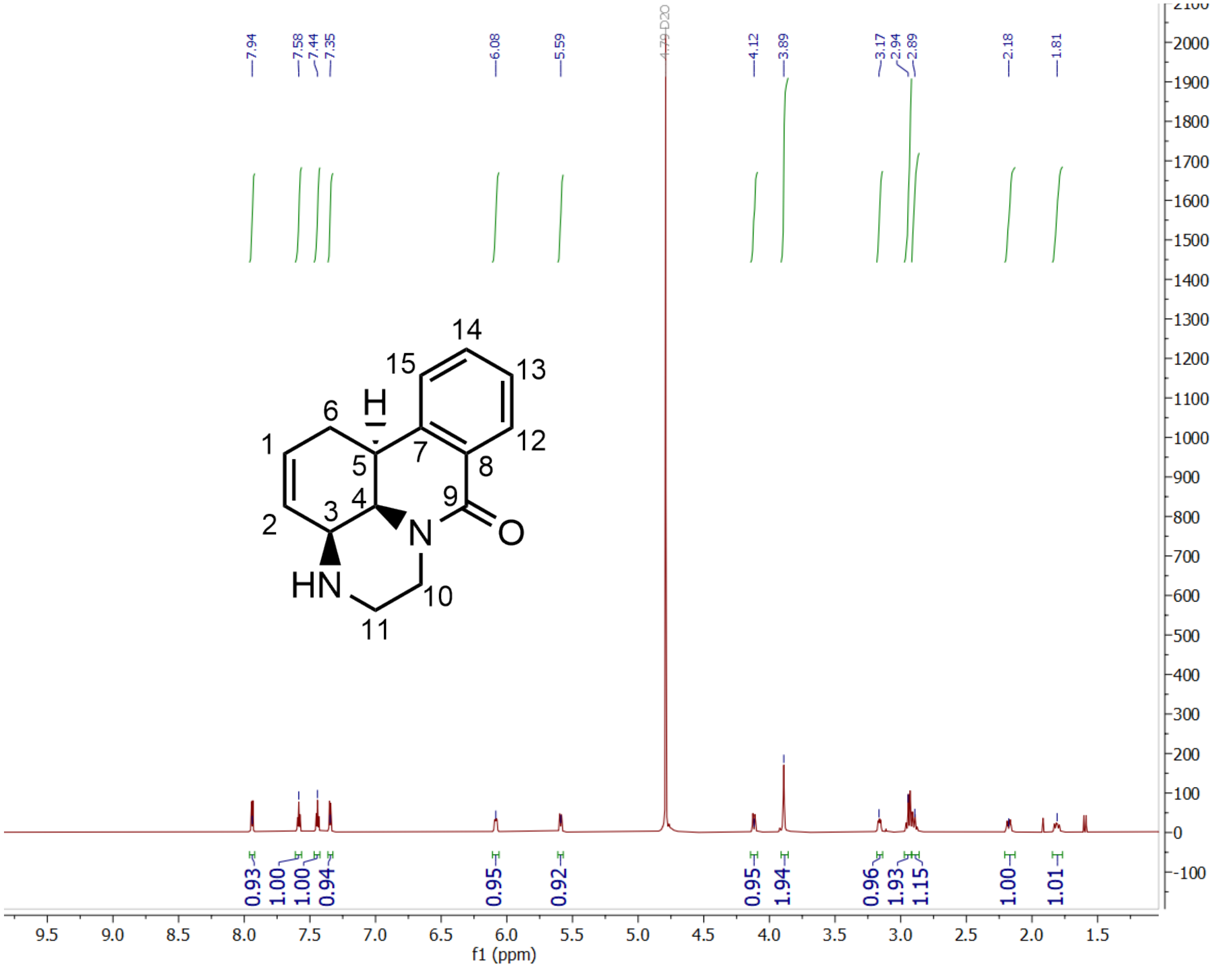

Supplementary Fig. 181: ¹H-NMR (D_2O) of Compound **85**.

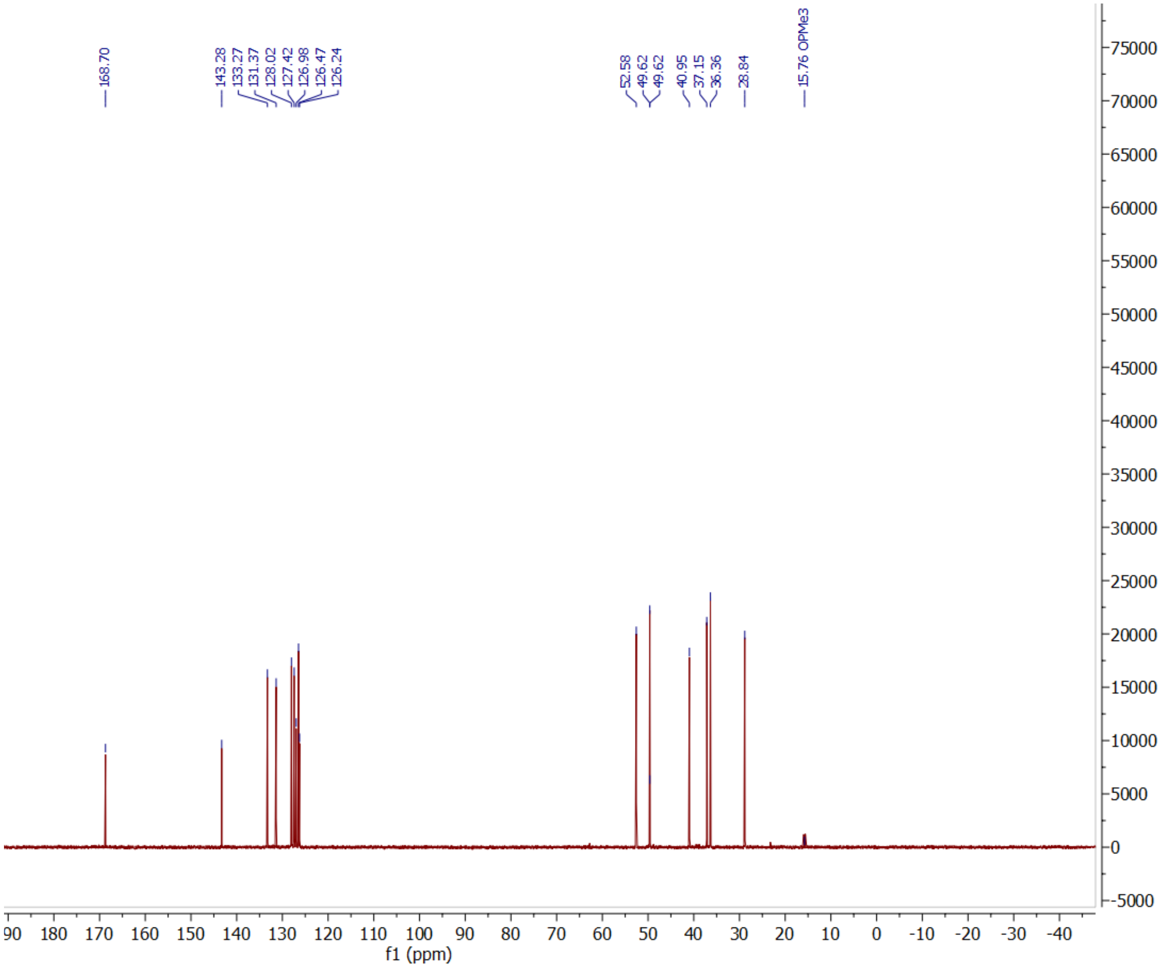

Supplementary Fig. 182: ^{13}C-NMR (D_2O) of Compound **85**.

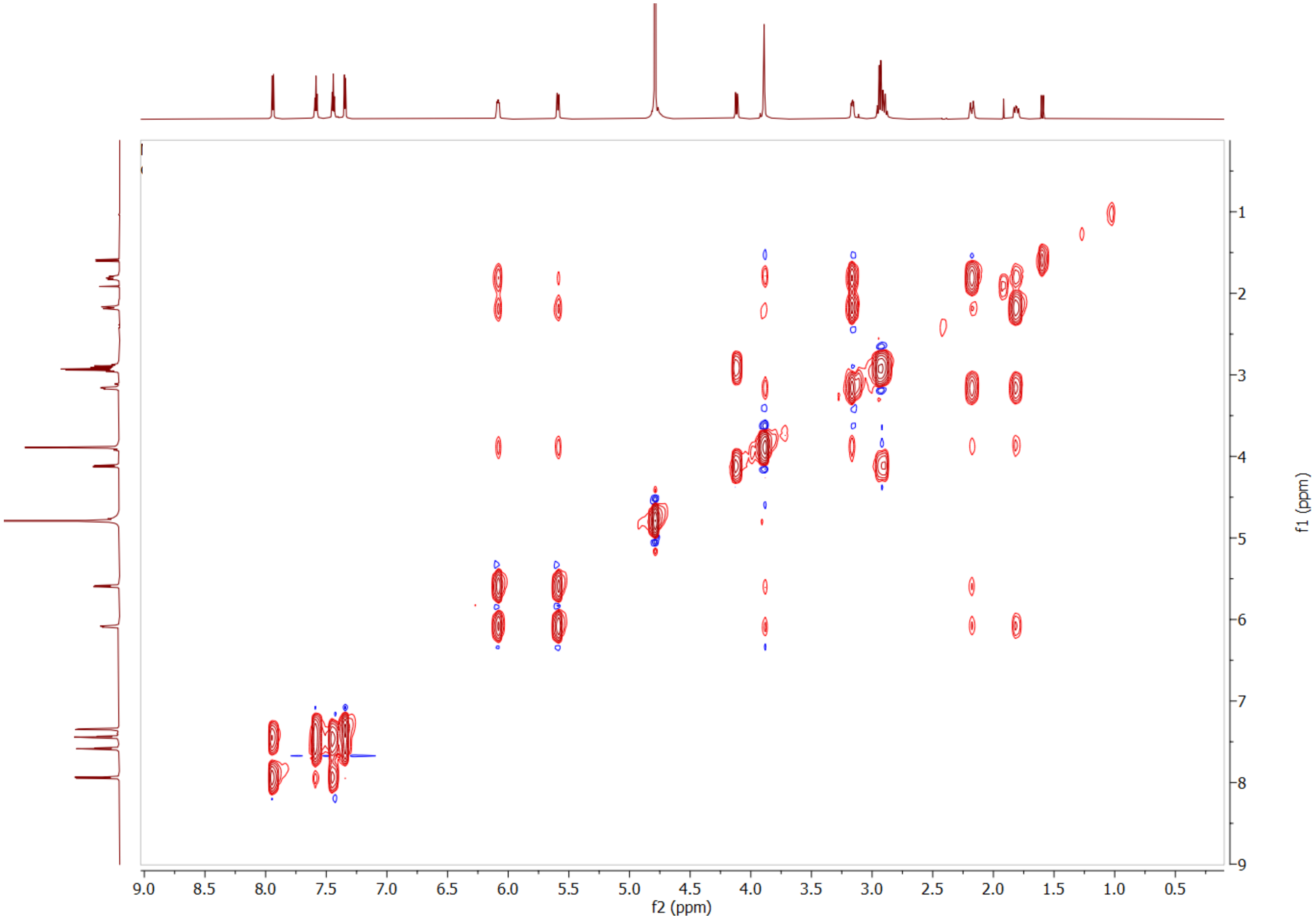

Supplementary Fig. 183: ^{1}H-^{1}H COSY (D_2O) of Compound **85**.

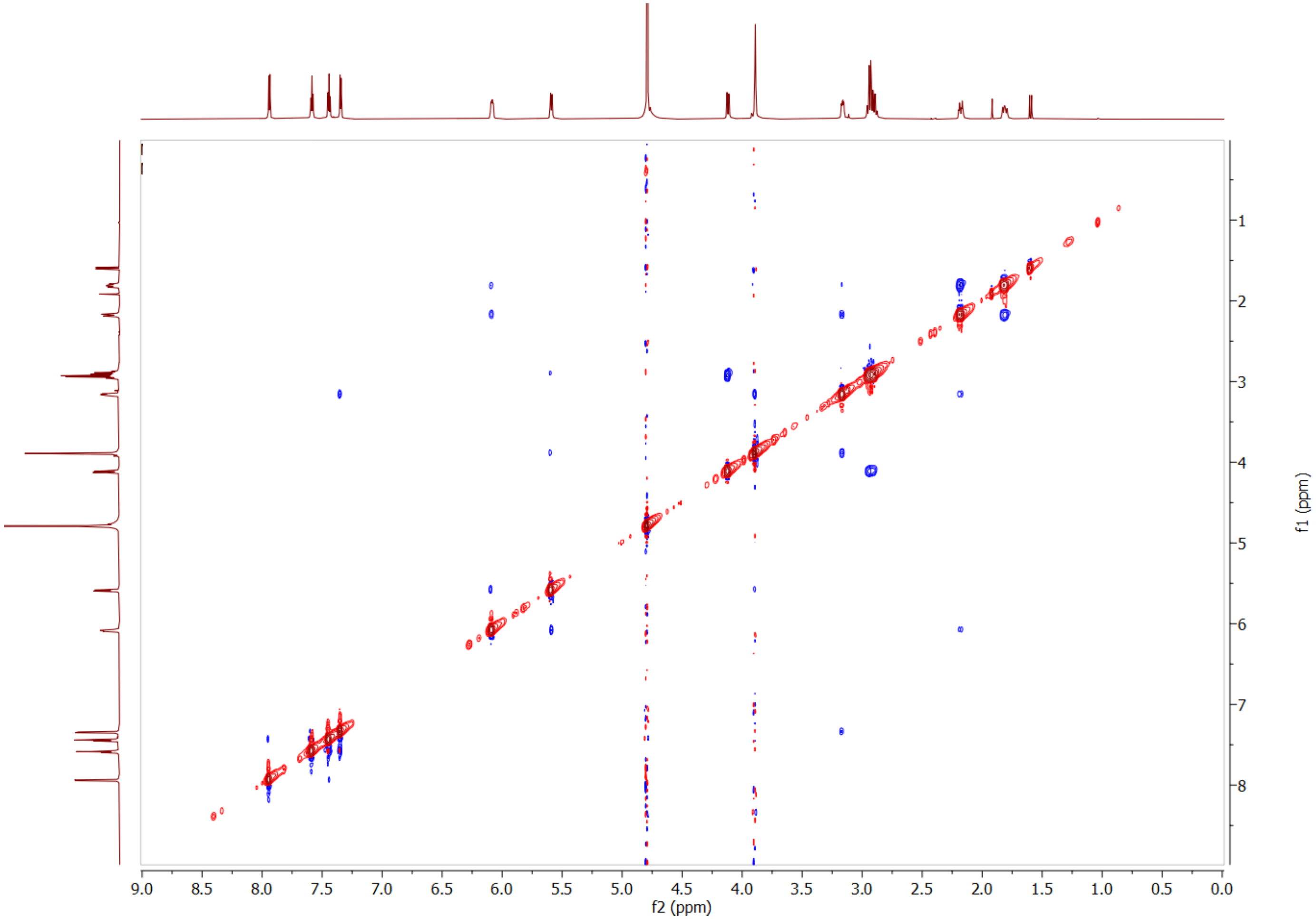

Supplementary Fig. 184: ^{1}H-1H NOESY (D_2O) of Compound **85**.

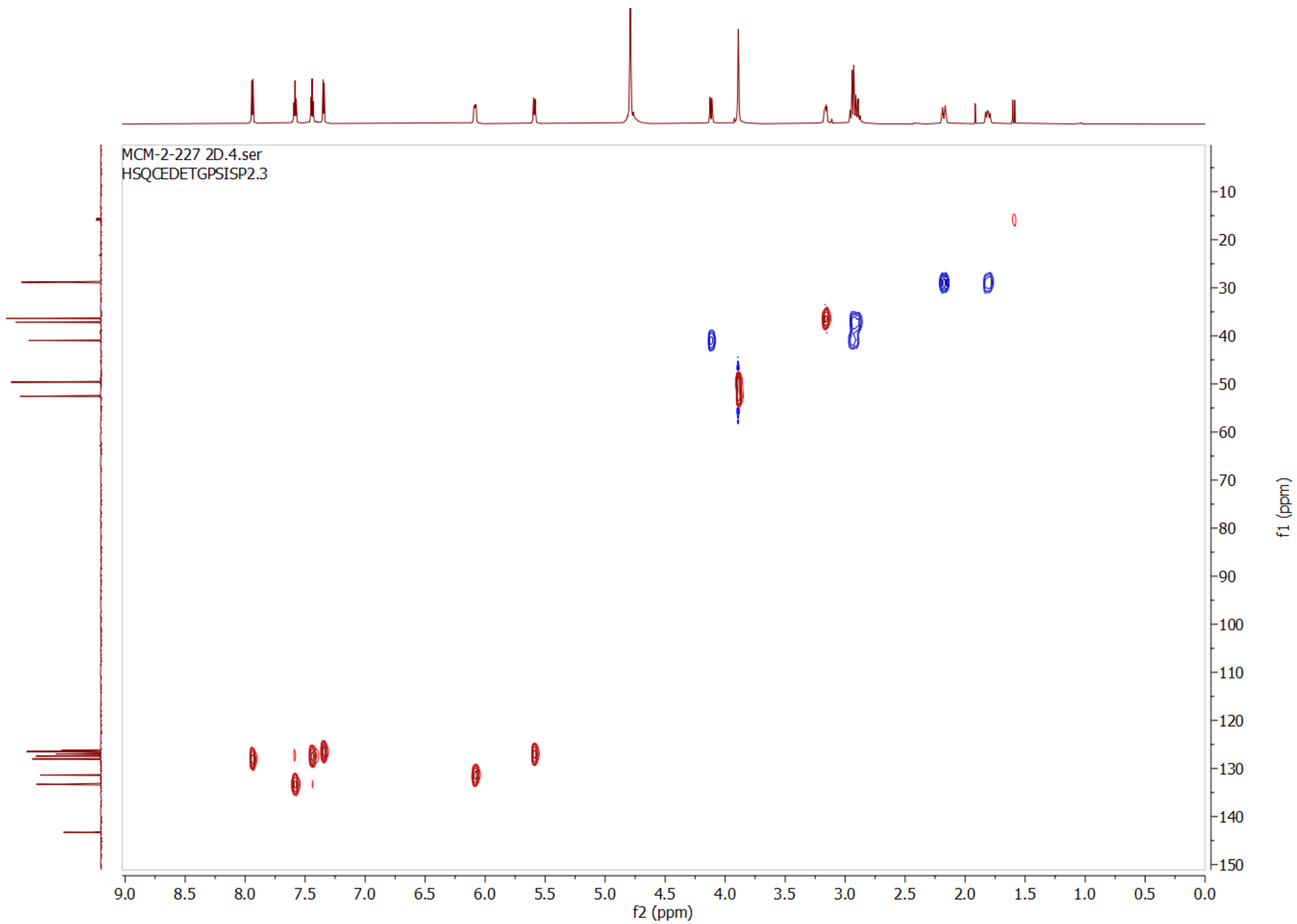

Supplementary Fig. 185: ^{1}H-^{13}C HSQC (D_2O) of Compound 85.

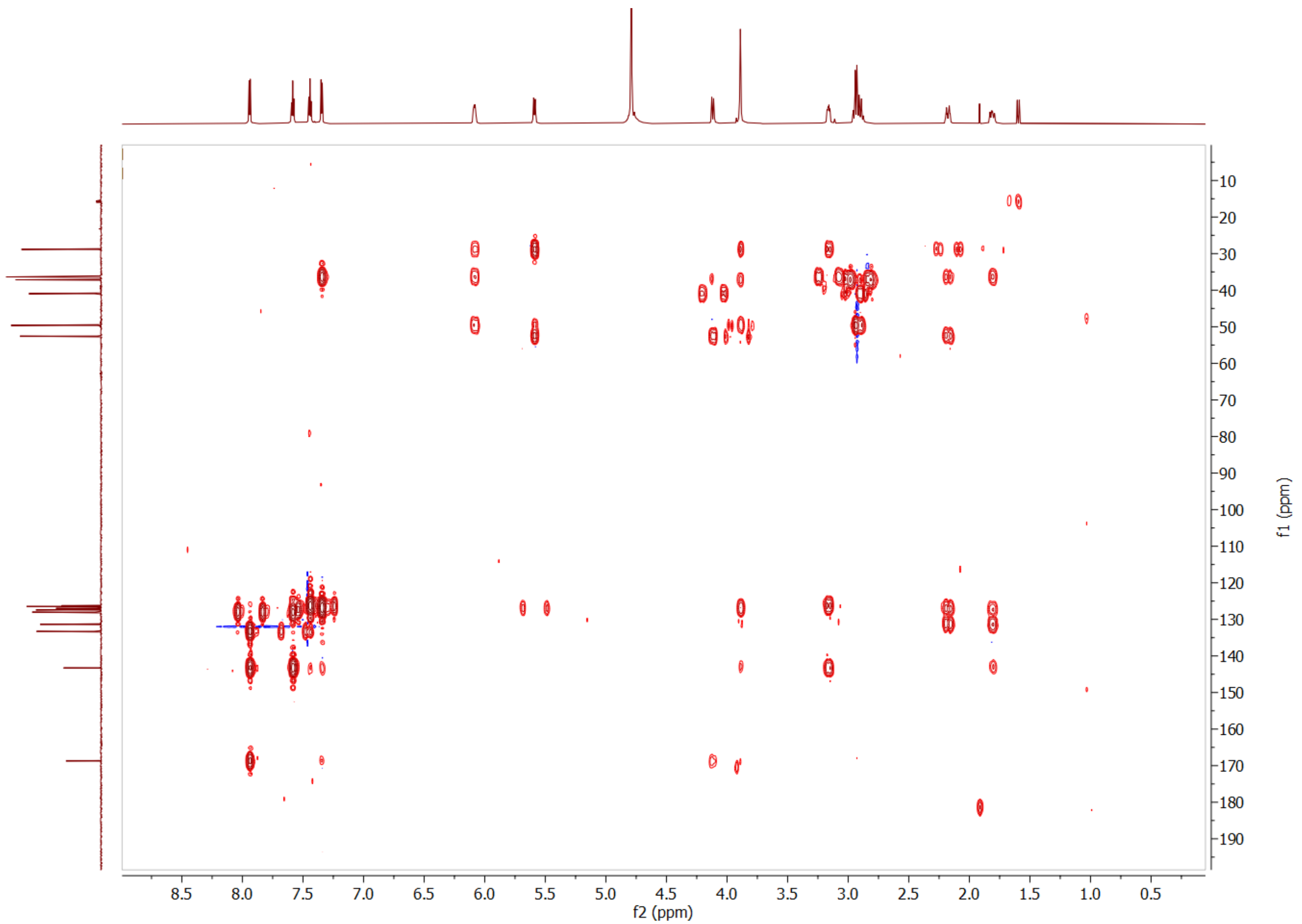

Supplementary Fig. 186: ^{1}H-13C HMBC (D_2O) of Compound **85**.

Supplementary Methods:

NMR spectra were obtained on 400, 600, or 800 MHz spectrometers. Chemical shifts are referenced to tetramethylsilane (TMS) utilizing residual ^1H signals of the deuterated solvents as internal standards. S46 Chemical Shifts are reported in ppm and coupling constants (J) are reported in hertz (Hz). Infrared spectra (IR) were recorded as a solid on a spectrometer with an ATR crystal accessory, and peaks are reported in cm^{-1}. Electrochemical experiments were performed under a nitrogen atmosphere. Most cyclic voltammetric data were recorded at ambient temperature at 100 mV/ s, unless otherwise noted, with a standard three-electrode cell from +1.8 to −1.8 V with a platinum working electrode, acetonitrile or N, N-dimethylacetamide (DMA) solvent, and tetrabutylammonium hexafluorophosphate (TBAH) electrolyte (~1.0 M). All potentials are reported versus the normal hydrogen electrode (NHE) using cobaltocenium hexafluorophosphate (E1/2 = −0.78 V, −1.75 V) or ferrocene (E1/2 = 0.55 V) as an internal standard. The peak separation of all reversible couples was less than 100 mV. All synthetic reactions were performed in a glovebox under a dry nitrogen atmosphere unless otherwise noted. All solvents were purged with nitrogen prior to use. Deuterated solvents were used as received from Cambridge Isotopes and were purged with nitrogen under an inert atmosphere. When possible, pyrazole protons of the tris(pyrazolyl)borate (Tp) ligand were uniquely assigned (e.g., “Tp3B”) using two-dimensional NMR data. If unambiguous assignments were not possible, Tp protons were labeled as “Tp3/5 or Tp4”. All J values for Tp protons are 2(±0.4) Hz. BH peaks (around 4−5 ppm) in the ^1H NMR spectra are not assigned due to their quadrupole broadening; However, confirmation of the BH group is provided by IR data (ca 2500 cm−1). Compounds **1**, **2**, **3**, **4** and **82** have been previously reported.[1] Full characterization of compounds is provided in the SI. Ground-state structures were optimized at the M06 level of theory using the 6-31G**[LANL2DZ for W] basis set in Gaussian 16.[2] Previous literature demonstrates that this functional and basis set choice accurately corroborates experimental results.[3] Vibrational frequency analysis verified that optimized structures were minima, and rigid-rotorharmonic-oscillator thermochemical chemical corrections were applied at 298 K and 1 atm utilizing Gaussian's default implementation. When solvent corrections were applied to estimate DGsolv, optimization and frequency calculations were performed using the SMD continuum solvent model with the appropriate solvent's parameters from Gaussian.

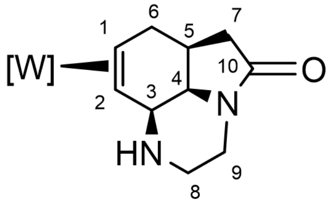

Compound 5:

Compound **2** (500 mg, 0.682 mmol) was placed in a test tube, with EtCN, and chilled to -20 °C. After 10 min, a 1 M HOTf/EtCN (1.36 mL, 1.36 mmol) solution was added to the test tube and the solution was allowed to stir at -20 °C for 60 min. In a separate test tube, ethylene diamine (0.91 mL, 13.6 mmol) in EtCN was cooled at -20 °C for 20 min. Then, the former solution was added to the latter, dropwise. The reaction stirred at -30 °C for 3 days. A 20% KO^{t}Bu/THF solution (2.06 mL, 3.41 mmol) was added dropwise to quench the reaction. The reaction was washed three times (H_2O: Na_3PO_4/DCM; 60 mL/60mL) and dried with golf-ball size Na_2SO_4. The organic layer was evaporated in vacuo. The resulting yellow film was dissolved in minimal DCM and pipetted in 150 mL of stirring pentane. Precipitation was induced twice to collect all the material. An off-white solid precipitated out and was collected on a 15 mL fine-porosity fritted disk, washed with pentane (2 × 10 mL) and desiccated overnight to yield compound **5** (396mg, 0.581 mmol, 85.3%, 50.1%-85.3%).

^{1}H-NMR (800 MHz, CD_3CN) δ: 9.26 (1H, d, TpA3), 8.07 (1H, d, TpB3), 7.89 (1H, d, TpB5), 7.84 (1H, d, TpC5), 7.71 (1H, d, TpA5), 7.45 (1H, d, TpC3), 6.40 (1H, t, TpB4), 6.26 (1H, t, TpC4), 6.20 (1H, t, TpA4), 4.17 (1H, dd, H3), 3.78 (1H, m, H8), 3.80 (1H, m, H4), 2.84 (1H, m H5), 2.68 (2H, m, H1/H8), 2.68 (1H, m, H9), 2.53 (2H, m, H7), 25349 (1H, dd, H9), 2.30 (1H, m, H6), 2.12 (1H, m, H6), 1.21 (1H, m, H2), 1.17 (9H, d, PMe3). **^{13}C-NMR (201 MHz, CD_3CN) δ**: 173.4 (1C, C10), 144.9 (1C, TpA3), 142.1 (1C, TpB3), 137.9 (1C, TpB5), 137.3 (1C, TpC5), 136.6 (1C, TpA5), 136.1 (1C, TpC3), 107.4 (1C, TpB4), 106.8 (1C, TpC4), 106.1 (1C, TpA4), 65.4 (1C, C4), 57.0 (1C, C3), 50.8 (1C, C1), 47.9 (1C, C2), 41.8 (1C, C8), 40.3 (1C, C7), 34.0 (1C, C5), 38.5 (1C, C9), 32.1 (1C, C6), 13.7 (3C, d J= 29.9 Hz, PMe_3). **CV** (DMA): $E_{p,a}$= 0.82 V (NHE). **IR (neat)**: *v*(NO) 1544 cm^{-1}, *v*(CO) 1655 cm^{-1}, *v*(BH) 2492 cm^{-1}.

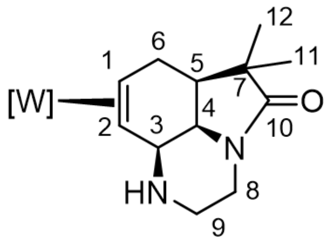

Compound 6:

Compound **3** (104 mg, 0.137 mmol) was placed in a test tube, with ACN, and chilled to -30 °C. After 10 min, a 1 M HOTf/ACN (0.274 mL, 0.274mmol) solution was added to the test tube and the solution was allowed to stir at -30 °C for 30 min. In a separate test tube, ethylene diamine (0.09 mL, 1.37 mmol) in ACN was cooled at -30 °C for 30 min. Then, the former solution was added to the latter, dropwise. The reaction stirred at -30 °C for 3 days and room temperature for 1 day. The reaction was washed three times (H_2O:Na_3PO_4/DCM; 60 mL/60mL) and dried with golf-ball size Na_2SO_4. The organic layer was evaporated in vacuo. The resulting yellow film was dissolved in minimal DCM and pipetted in 150 mL of stirring pentane. Precipitation was induced twice to collect all the material. An off-white solid precipitated out and was collected on a 15 mL fine-porosity fritted disk, washed with pentane (2 × 10 mL) and desiccated overnight to yield compound **6** (81 mg, 0.110 mmol, 83%, 72-83%).

^{1}H-NMR (800 MHz, CD_3CN) δ: 8.94 (1H, d, TpA3), 8.03 (1H, d, TpB3), 7.86 (1H, d, TpB5), 7.80 (1H, d, TpC5), 7.71 (1H, d, TpA5), 7.32 (1H, d, TpC3), 6.37 (1H, t, TpB4), 6.22 (1H, t, TpC4), 6.20 (1H, t, TpA4), 4.19 (1H, bs, H3), 3.76 (1H, d, H4), 3.73 (1H, d, H9), 2.74 (1H, m H8), 2.70 (1H, m, H9), 2.55 (2H, m, H1/H8), 2.44 (1H, m, H6), 2.35 (1H, m, H5), 2.31 (1H, m, H6), 1.33 (3H, s, H12), 1.20 (1H, m, H2), 1.10 (3H, s, H11), 1.15 (9H, d, PMe3). **^{13}C-NMR (201 MHz, CD_3CN) δ**: 179.0 (1C, C10), 146.8 (1C, TpA3), 144.8 (1C, TpB3), 142.0 (1C, TpB5), 137.9 (1C, TpC5), 137.0 (1C, TpA5), 136.7 (1C, TpC3), 107.5 (1C, TpB4), 106.9 (1C, TpC4), 106.3 (1C, TpA4), 61.4 (1C, C4), 57.5 (1C, C3), 52.2 (1C, C1), 48.5 (1C, C2), 46.2 (1C, C5), 43.9 (1C, C7), 41.2 (1C, C9), 38.4 (1C, C8), 30.3 (1C, C11), 29.3 (1C, C6), 20.8 (1C, C12), 13.7 (3C, d J= 29.4 Hz, PMe_3).

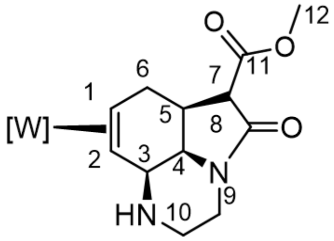

Compound 7:

Compound **4** (50 mg; 0.063 mmol) was placed in a test tube, with ACN (2 mL), and chilled to -30 °C. After 10 min, a 1 M HOTf/ACN (0.126 mL, 0.126 mmol) solution was added to the test tube and the solution was allowed to stir at -30 °C for 15 min. In a separate test tube, ethylene diamine (0.042 mL; 0.632 mmol) in ACN (2 mL) was cooled at -30 °C for 30 min. Then, the former solution was added to the latter, dropwise. The reaction stirred at -30 °C for 3 days. The reaction was washed three times (H_2O:$NaHCO_3$/DCM; 60 mL/60mL) and dried over anhydrous Na_2SO_4. The organic layer was evaporated in vacuo. The resulting yellow film was dissolved in minimal DCM and pipetted in 50 mL of stirring pentane. Precipitation was induced twice to collect all the material. An off-white solid precipitated out and was collected on a 15 mL fine-porosity fritted disk, washed with pentane (2 × 10 mL) and desiccated overnight to yield compound **7** (25 mg, 0.033 mmol, 54%).

^{1}H NMR (800 MHz, CD_3CN) δ 9.24 (1H, d, *J* = 2.1 Hz, Tp3A), 8.04 (1H, d, *J* = 2.0 Hz, Tp3B), 7.87 (1H, d, *J* = 2.1 Hz, Tp5B), 7.81 (1H, d, *J* = 2.1 Hz, Tp5C), 7.68 (1H, d, Tp5A), 7.46 (1H, d, *J* = 2.2 Hz, Tp3C), 6.38 (1H, d, *J* = 2.2 Hz, Tp4B), 6.23 (1H, d, *J* = 2.2 Hz, Tp4C), 6.18 (1H, d, Tp4A), 4.19 (1H, ddd, *J* = 11.9, 7.8, 4.2 Hz, H3), 3.82 (1H, dd, *J* = 9.6, 4.1Hz, H4), 3.79 (1H, m, H9), 3.73 (1H, d, *J* = 6.0 Hz, H7), 3.72 (3H, s, H12), 3.06 (1H, m, H5), 2.75 (1H, m, H9), 2.64 (1H, m, H10), 2.60 (1H, m, H1), 2.55 (1H, dddd, *J* = 13.4, 12.1, 4.2, 1.0 Hz, H10), 2.36 (1H, ddt, *J* = 14.4, 9.8, 4.8 Hz, H6), 2.22 (1H, dddd, *J* = 23.1, 14.0, 6.2, 2.4 Hz, H6), 1.22 (1H, m, H2), 1.13 (9H, d, *J* = 8.4 Hz, PMe_3).**^{13}C NMR (201 MHz, CD_3CN) δ** 173.3 (1C, C11), 168.7 (1C, C8), 148.0 (1C, Tp3A), 144.9 (1C, d, J = 7.8 Hz, Tp3B), 142.3 (1C, Tp3C), 138.0 (1C, Tp5C), 137.1 (1C, Tp5B), 136.6 (1C, Tp5A), 107.6 (1C, Tp4B), 106.8 (1C, Tp4C), 106.2 (1C, Tp4A), 64.2 (1C, C4), 56.6 (1C, C3), 54.2 (1C, C7), 52.9 (1C, C12), 50.4 (1C, d, J = 12.8 Hz, C1), 46.6 (1C, C2), 42.3 (1C, C9), 40.6 (1C, C5), 38.0 (1C, C10), 31.4 (1C, C6), 13.5 (3C, d, J = 28.2 Hz, PMe_3). **IR (neat):** *v*(NO) 1544 cm^{-1}, *v*(amide CO) 1674 cm^{-1}, *v*(ester CO) 1731 cm^{-1}, *v*(BH) 2438 cm^{-1}.

Minor
^{1}H NMR (800 MHz, CD_3CN) δ 9.31 (1H, d, J = 2.0 Hz, Tp3A), 8.05 (1H, d, J = 2.0 Hz, Tp3B), 7.87 (1H, d, J = 2.1 Hz, Tp5B), 7.81 (1H, d, J = 2.1 Hz, Tp5C), 7.68 (1H, d, Tp5A), 7.43 (1H, d, J = 2.1 Hz, Tp3C), 6.38 (1H, t, J = 2.2 Hz, Tp4B), 6.23 (1H, t, J = 2.2 Hz, Tp4C), 6.18 (1H, t, Tp4A), 4.19 (1H, under major, H3), 3.75 (1H, m, H4), 3.66 (1H, m, H7), 3.06 (1H, m, H5), 2.75 (1H, under major, H9), 2.64 (1H, under major H10, H9), 2.60 (1H, m, H1), 2.22 (1H, under major, H6), 1.22 (1H, under major, H2), 1.13 (9H, under major, PMe_3). **^{13}C NMR (201 MHz, CD_3CN) δ** 148.2 (1C, Tp3A), 144.9 (1C, Tp3B), 142.1 (1C, Tp3C), 138.0 (1C, Tp5C), 136.6 (1C, Tp5A), 64.2 (1C, C4), 56.6 (1C, C3), 51.0 (1C, d, J = 12.8, C1), 46.5 (1C, C2), 42.3 (1C, C9), 38.7 (1C, C5), 38.1 (1C, C10), 13.5 (3C, d, J = 28.4 Hz, PMe_3).

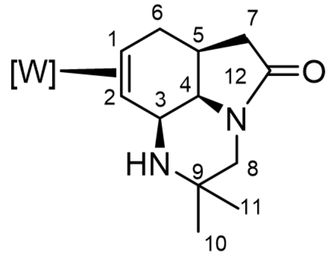

Compound 8:

Compound **2** (140 mg, 0.191 mmol) was placed in a test tube, with EtCN, and chilled to -30 °C. After 10 min, a 1 M HOTf/EtCN (0.382 mL, 0.382 mmol) solution was added to the test tube and the solution was allowed to stir at -30 °C for 30 min. In a separate test tube, 2-methylpropane-1,2-diamine (0.199 mL, 1.91 mmol) in EtCN was cooled at -30 °C for 30 min. Then, the former solution was added to the latter, dropwise. The reaction stirred at -30 °C for 24 h and at room temperature for 4 h. The reaction was washed three times (H_2O:Na_2CO_3/DCM; 60 mL/60mL) and dried with golf-ball size Na_2SO_4. The organic layer was evaporated in vacuo. The resulting yellow film was dissolved in minimal DCM and pipetted in 150 mL of stirring pentane. Precipitation was induced twice to collect all the material. An off-white solid precipitated out and was collected on a 15 mL fine-porosity fritted disk, washed with pentane (2 × 10 mL) and desiccated overnight to yield compound **8** (105 mg, 0.148 mmol, 77.5%).

^{1}H-NMR (800 MHz, CD_3CN) δ: 8.72 (1H, d, TpA3), 8.08 (1H, d, TpB3), 7.86 (1H, d, TpB5), 7.81 (1H, d, TpC5), 7.73 (1H, d, TpA5), 7.44 (1H, d, TpC3), 6.38 (1H, t, TpB4), 6.26 (1H, t, TpC4), 6.23 (1H, t, TpA4), 4.07 (1H, t, H3), 3.73 (1H, t, H4), 3.69 (1H, d, H8), 3.01 (1H, bs, H6), 2.76 (1H, m, H5), 2.60 (1H, m, H1), 2.55 (1H, m, H7), 2.50 (1H, m, H8), 2.36 (1H, m, H6), 2.30 (1H, dd, H7), 1.24 (1H, m, H2), 1.17 (9H, d, PMe3), 1.04 (3H, s, H11), 0.98 (3H, s, H10). **^{13}C-NMR (201 MHz, CD_3CN) δ**: 174.3 (1C, C12), 144.6 (1C, TpA3), 142.1 (1C, TpB3), 142.1 (1C, TpB5), 137.9 (1C, TpC5), 137.8 (1C, TpC3), 137.1 (1C, TpA5), 107.4 (1C, TpB4), 106.9 (1C, TpC4), 106.4 (1C, TpA4), 60.6 (1C, C4), 54.6 (1C, C3), 52.7 (1C, C2), 52.1 (1C, C1), 50.7 (1C, C9), 49.7 (1C, C8), 37.6 (1C, C7), 32.6 (1C, C6), 31.6 (1C, C5), 30.1 (1C, 11), 29.6 (1C, C10), 13.6 (3C, PMe_3).

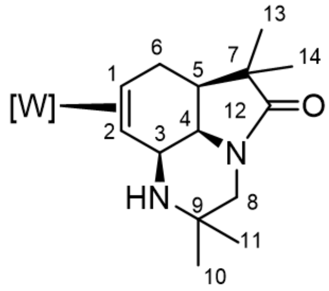

Compound 9:

Compound **3** (100 mg, 0.131 mmol) was placed in a test tube, with EtCN, and chilled to -30 °C. After 10 min, a 1 M HOTf/EtCN (0.263 mL, 0.263 mmol) solution was added to the test tube and the solution was allowed to stir at -30 °C for 30 min. In a separate test tube, 2-methylpropane-1,2-diamine (0.137 mL, 1.31 mmol) in EtCN was cooled at -30 °C for 30 min. Then, the former solution was added to the latter, dropwise. The reaction stirred at -30 °C for 24 h and at room temperature for 4 h. The reaction was washed three times (H_2O:Na_2CO_3/DCM; 60 mL/60mL) and dried with golf-ball size Na_2SO_4. The organic layer was evaporated in vacuo. The resulting yellow film was dissolved in minimal DCM and pipetted in 150 mL of stirring pentane. Precipitation was induced twice to collect all the material. An off-white solid precipitated out and was collected on a 15 mL fine-porosity fritted disk, washed with pentane (2 × 10 mL) and desiccated overnight to yield compound **9** (69 mg, 0.094 mmol, 71%).

^{1}H-NMR (800 MHz, CD_3CN) δ: 8.11 (1H, d, TpB3), 8.07 (1H, d, TpA3), 7.85 (1H, d, TpB5), 7.83 (1H, d, TpC5), 7.75 (1H, d, TpA5), 7.45 (1H, d, TpC3), 6.37 (1H, t, TpB4), 6.28 (1H, t, TpC4), 6.24 (1H, t, TpA4), 3.91 (1H, d, H3), 3.78 (1H, t, H4), 3.64 (1H, d, H8), 3.48 (1H, t, H6), 2.59 (1H, td, H1), 2.50 (1H, m, H8), 2.31 (1H, m, H5), 2.20 (1H, bs, H6), 1.23 (9H, d, PMe3), 1.18 (1H, m, H2), 1.09 (6H, d, H13/H14), 1.04 (3H, s, H10), 1.03 (3H, s, H11). **^{13}C-NMR (201 MHz, CD_3CN) δ**: 179.6 (1C, C12), 144.8 (1C, TpB3), 144.3 (1C, TpA3), 142.1 (1C, TpB5), 138.0 (1C, TpC5), 137.1 (1C, TpC3), 136.9 (1C, TpA5), 107.3 (1C, TpB4), 106.9 (2C, TpC4/TpA4), 54.7 (2C, C4/C2), 52.9 (1C, C1), 51.8 (1C, C3), 52.7 (1C, C9), 47.9 (1C, C8), 44.5 (1C, C7), 42.6 (1C, C5), 28.3 (1C, C6), 30.5 (1C, 11), 27.9 (2C, C13/C14), 27.0 (1C, C10), 13.9 (3C, PMe_3).

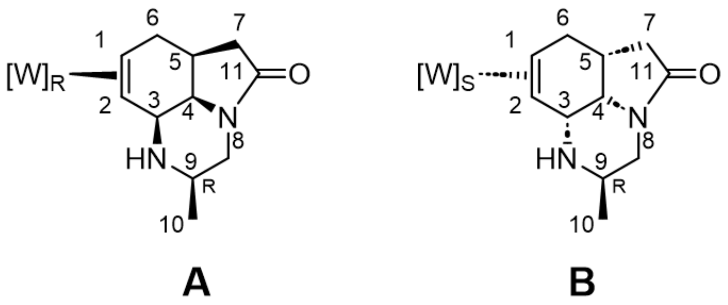

Compound 10A+10B

Compound 3 (100 mg, 0.136 mmol) was placed in a test tube, with EtCN, and chilled to -30 °C. After 10 min, a 1 M HOTf/EtCN (0.273 mL, 0.273 mmol) solution was added to the test tube and the solution was allowed to stir at -30 °C for 30 min. In a separate test tube, (*R*)-Propane-1,2-diamine (101 mg, 1.36 mmol) in EtCN was cooled at -30 °C for 30 min. Then, the former solution was added to the latter, dropwise. The reaction stirred at -30 °C for 24 h and at room temperature for 4 h. The reaction was washed three times (H_2O:Na_2CO_3/DCM; 60 mL/60mL) and dried with golf-ball size Na_2SO_4. The organic layer was evaporated in vacuo. The resulting yellow film was dissolved in minimal DCM and pipetted in 150 mL of stirring pentane. Precipitation was induced twice to collect all the material. An off-white solid precipitated out and was collected on a 15 mL fine-porosity fritted disk, washed with pentane (2 × 10 mL) and desiccated overnight to yield compound 10A + 10B (76 mg, 0.110 mmol, 80%).

10A

^{1}H-NMR (800 MHz, CD_3CN) δ: 8.46 (1H, d, TpA3), 8.06 (1H, d, TpB3), 7.86 (1H, d, TpB5), 7.82 (1H, d, TpA5), 7.73 (1H, d, TpC5), 7.41 (1H, d, TpC3), 6.37 (1H, t, TpB4), 6.25 (1H, t, TpC4), 6.24 (1H, t, TpA4), 4.11 (1H, t, H3), 3.89 (1H, t, H4), 3.23 (1H, dd, H8), 3.10 (1H, m, H9), 3.02 (1H, dd, H8), 2.85 (1H, m, H6), 2.75 (1H, m, H5), 2.61 (1H, m, H1), 2.48 (1H, m, H7), 2.33 (2H, m, H6/H7), 1.18 (9H, d, PMe3), 1.00 (3H, d, H10), 0.94 (1H, ddd, H2). **^{13}C-NMR (201 MHz, CD_3CN) δ:** 175.4 (1C, C11), 145.2 (1C, TpA3), 144.8 (1C, TpB3), 142.0 (1C, TpB5), 138.0 (1C, TpA5), 137.1 (1C, TpC5), 136.9 (1C, TpC3), 107.4 (1C, TpB4), 106.9 (1C, TpC4), 106.5 (1C, TpA4), 58.4 (1C, C4), 58.0 (1C, C3), 55.1 (1C, C2), 51.8 (1C, C1), 47.8 (1C, C9), 46.7 (1C, C8), 37.1 (1C, C7), 32.3 (1C, C6), 32.1 (1C, C5), 21.7 (1C, C10), 13.7 (3C, d J= 29.8 Hz, PMe_3).

10B

^{1}H-NMR (800 MHz, CD_3CN) δ: 9.38 (1H, d, TpA3), 8.05 (1H, d, TpB3), 7.87 (1H, d, TpA5), 7.80 (1H, d, TpB5), 7.68 (1H, d, TpC5), 7.42 (1H, d, TpC3), 6.38 (1H, t, TpB4), 6.21 (1H, t, TpC4), 6.20 (1H, t, TpA4), 4.16 (1H, dd, H3), 3.85 (1H, dd, H8), 3.68 (1H, dd, H4), 2.85 (1H, m, H5), 2.71 (1H, m, H9), 2.65 (1H, m, H1), 2.55 (2H, m, H7), 2.28 (1H, m, H8), 2.15 (2H, m, H6), 1.12 (1H, td, H2), 1.14 (9H, d, PMe3), 0.89 (3H, d, H10). **^{13}C-NMR (201 MHz, CD_3CN) δ:** 173.2 (1C, C11), 148.2 (1C, TpA3), 144.9 (1C, TpB3), 142.1 (1C, TpA5), 138.0 (1C, TpB5), 137.0 (1C, TpC5), 136.6 (1C, TpC3), 107.5 (1C, TpB4), 106.8 (1C, TpC4), 106.0 (1C, TpA4), 64.5 (1C, C4), 57.3 (1C, C3), 50.8

(1C, C1), 48.0 (1C, C8), 47.9 (1C, C2), 42.7 (1C, C9), 36.5 (1C, C7), 33.9 (1C, C5), 32.2 (1C, C6), 19.5 (1C, C10), 13.4 (3C, d J= 28.9 Hz, PMe_3).

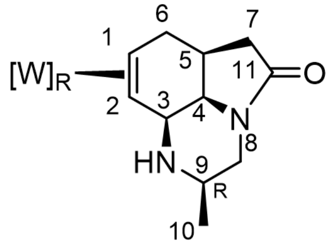

Compound 11:

Compound **3** (70 mg, 0.095 mmol) was placed in a test tube, with EtCN, and chilled to -30 °C. After 10 min, a 1 M HOTf/EtCN (0.191 mL, 0.191 mmol) solution was added to the test tube and the solution was allowed to stir at -30 °C for 30 min. In a separate test tube, (*R*)-Propane-1,2-diamine (70.8 mg, 0.955 mmol) in EtCN was cooled at -30 °C for 30 min. Then, the former solution was added to the latter, dropwise. The reaction stirred at -30 °C for 24 h and at room temperature for 4 h. The reaction was washed three times (H_2O:Na_2CO_3/DCM; 60 mL/60mL) and dried with golf-ball size Na_2SO_4. The organic layer was evaporated in vacuo. The resulting yellow film was dissolved in minimal DCM and pipetted in 150 mL of stirring pentane. Precipitation was induced twice to collect all the material. An off-white solid precipitated out and was collected on a 15 mL fine-porosity fritted disk, washed with pentane (2 × 10 mL) and desiccated overnight to yield compound **11** (55 mg, 0.079 mmol, 82.9%).

^{1}H-NMR (800 MHz, CD_3CN) δ: 8.46 (1H, d, TpA3), 8.06 (1H, d, TpB3), 7.86 (1H, d, TpB5), 7.82 (1H, d, TpA5), 7.73 (1H, d, TpC5), 7.41 (1H, d, TpC3), 6.37 (1H, t, TpB4), 6.25 (1H, t, TpC4), 6.24 (1H, t, TpA4), 4.11 (1H, t, H3), 3.89 (1H, t, H4), 3.23 (1H, dd, H8), 3.10 (1H, m, H9), 3.02 (1H, dd, H8), 2.85 (1H, m, H6), 2.75 (1H, m, H5), 2.61 (1H, m, H1), 2.48 (1H, m, H7), 2.33 (2H, m, H6/H7), 1.18 (9H, d, PMe3), 1.00 (3H, d, H10), 0.94 (1H, ddd, H2). **^{13}C-NMR (201 MHz, CD_3CN) δ**: 175.4 (1C, C11), 145.2 (1C, TpA3), 144.8 (1C, TpB3), 142.0 (1C, TpB5), 138.0 (1C, TpA5), 137.1 (1C, TpC5), 136.9 (1C, TpC3), 107.4 (1C, TpB4), 106.9 (1C, TpC4), 106.5 (1C, TpA4), 58.4 (1C, C4), 58.0 (1C, C3), 55.1 (1C, C2), 51.8 (1C, C1), 47.8 (1C, C9), 46.7 (1C, C8), 37.1 (1C, C7), 32.3 (1C, C6), 32.1 (1C, C5), 21.7 (1C, C10), 13.7 (3C, d J= 29.5 Hz, PMe_3).

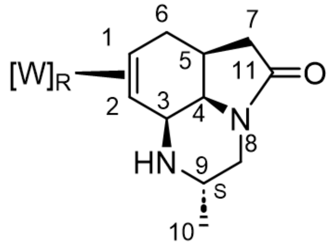

Compound 12:

Compound **3** (70 mg, 0.095 mmol) was placed in a test tube, with EtCN, and chilled to -30 °C. After 10 min, a 1 M HOTf/EtCN (0.191 mL, 0.191 mmol) solution was added to the test tube and the solution was allowed to stir at -30 °C for 30 min. In a separate test tube, (*S*)-Propane-1,2-diamine (70.8 mg, 0.955 mmol) in EtCN was cooled at -30 °C for 30 min. Then, the former solution was added to the latter, dropwise. The reaction stirred at -30 °C for 24 h and at room temperature for 4 h. The reaction was washed three times (H_2O:Na_2CO_3/DCM; 60 mL/60mL) and dried with golf-ball size Na_2SO_4. The organic layer was evaporated in vacuo. The resulting yellow film was dissolved in minimal DCM and pipetted in 150 mL of stirring pentane. Precipitation was induced twice to collect all the material. An off-white solid precipitated out and was collected on a 15 mL fine-porosity fritted disk, washed with pentane (2 × 10 mL) and desiccated overnight to yield compound **12** (52 mg, 0.075 mmol, 78%).

^{1}H-NMR (800 MHz, CD_3CN) δ: 9.38 (1H, d, TpA3), 8.05 (1H, d, TpB3), 7.87 (1H, d, TpA5), 7.80 (1H, d, TpB5), 7.68 (1H, d, TpC5), 7.42 (1H, d, TpC3), 6.38 (1H, t, TpB4), 6.21 (1H, t, TpC4), 6.20 (1H, t, TpA4), 4.16 (1H, dd, H3), 3.85 (1H, dd, H8), 3.68 (1H, dd, H4), 2.85 (1H, m, H5), 2.71 (1H, m, H9), 2.65 (1H, m, H1), 2.55 (2H, m, H7), 2.28 (1H, m, H8), 2.15 (2H, m, H6), 1.12 (1H, td, H2), 1.14 (9H, d, PMe3), 0.89 (3H, d, H10). **^{13}C-NMR (201 MHz, CD_3CN) δ**: 173.2 (1C, C11), 148.2 (1C, TpA3), 144.9 (1C, TpB3), 142.1 (1C, TpA5), 138.0 (1C, TpB5), 137.0 (1C, TpC5), 136.6 (1C, TpC3), 107.5 (1C, TpB4), 106.8 (1C, TpC4), 106.0 (1C, TpA4), 64.5 (1C, C4), 57.3 (1C, C3), 50.8 (1C, C1), 48.0 (1C, C8), 47.9 (1C, C2), 42.7 (1C, C9), 36.5 (1C, C7), 33.9 (1C, C5), 32.2 (1C, C6), 19.5 (1C, C10), 13.4 (3C, d J= 29.2 Hz, PMe_3).

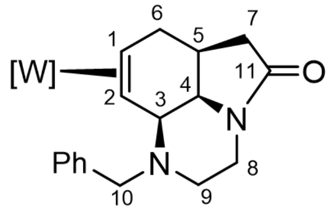

Compound 13:

Compound **2** (120 mg, 0.164 mmol) was placed in a test tube, with EtCN, and chilled to -30 °C. After 10 min, a 1 M HOTf/EtCN (0.327 mL, 0.327 mmol) solution was added to the test tube and the solution was allowed to stir at -30 °C for 30 min. In a separate test tube, *N1*-benzylethane-1,2-diamine (0.199 mL, 1.64 mmol) in EtCN was cooled at -30 °C for 30 min. Then, the former solution was added to the latter, dropwise. The reaction stirred at -30 °C for 3 days. A 20% KO^{t}Bu in THF (0.297 mL, 0.491 mmol) was added to quench the reaction. The reaction was washed three times (H_2O:Na_2CO_3/DCM; 60 mL/60mL) and dried with golf-ball size Na_2SO_4. The organic layer was evaporated in vacuo. The resulting yellow film was dissolved in minimal DCM and pipetted in 150 mL of stirring pentane. Precipitation was induced twice to collect all the material. An off-white solid precipitated out and was collected on a 15 mL fine-porosity fritted disk, washed with pentane (2 × 10 mL) and desiccated overnight to yield compound **13** (100 mg, 0.130 mmol, 79%).

^{1}H-NMR (800 MHz, CD_2Cl_2) δ: 9.41 (1H, d, TpA3), 8.08 (1H, d, TpB3), 7.79 (1H, d, TpB5), 7.70 (1H, d, TpC5), 7.55 (1H, d, TpA5), 7.44 (1H, d, TpC3), 7.32-7.24 (5H, m, Ph), 6.37 (1H, t, TpB4), 6.17 (1H, t, TpC4), 6.09 (1H, t, TpA4), 4.41 (1H, s, H3), 4.46 (1H, m, H8), 4.29 (1H, m, H8), 4.12 (1H, dd, H4), 3.57 (2H, m, H10/H9), 3.18 (1H, m, H6), 2.72 (1H, m, H1), 2.58 (2H, m, H6/H9), 2.49 (1H, d, H7), 2.40 (1H, m, H5), 2.35 (1H, d, H7), 2.17 (1H, d, H10), 1.53 (1H, td, H2), 1.14 (9H, d, PMe3). **^{13}C-NMR (201 MHz, CD_2Cl_2) δ**: 173.3 (1C, C11), 148.8 (1C, TpA3), 144.0 (1C, TpB3), 141.2 (1C, TpB5), 139.7 (1C, TpC5), 137.1 (1C, TpA5), 136.0 (1C, TpC3), 129.7, 128.6, 127.3 (6C, Ph), 106.9 (1C, TpB4), 105.9 (1C, TpC4), 105.3 (1C, TpA4), 64.4 (1C, C3), 56.7 (2C, C8/C4), 50.3 (1C, C1), 47.9 (1C, C2), 39.2 (1C, C10), 36.5 (1C, C9), 33.30 (1C, C7), 31.9 (1C, C5), 31.8 (1C, C6), 13.2 (3C, d J= 27.4 Hz, PMe_3).

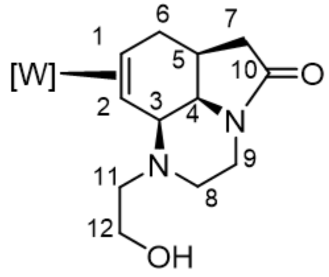

Compound 14:

Compound **2** (300 mg, 0.409 mmol) was placed in a test tube, with EtCN, and chilled to -30 °C. After 10 min, a 1 M HOTf/EtCN (0.818 mL, 0.818 mmol) solution was added to the test tube and the solution was allowed to stir at -30 °C for 30 min. In a separate test tube, N-(2-Hydroxyethyl)ethylenediamine (0.358 mL, 4.09 mmol) in EtCN was cooled at -30 °C for 30 min. Then, the former solution was added to the latter, dropwise. The reaction stirred at -30 °C for 3 days. A 20% KO^{t}Bu/THF solution (0.741 mL, 1.23 mmol) was added to quench the reaction. The reaction was washed three times (H_2O:Na_2CO_3/DCM; 60 mL/60mL) and dried with golf-ball size Na_2SO_4. The organic layer was evaporated in vacuo. The resulting yellow film was dissolved in minimal DCM and pipetted in 50 mL of stirring pentane. Precipitation was induced twice to collect all the material. An off-white solid precipitated out and was collected on a 15 mL fine-porosity fritted disk, washed with pentane (2 × 10 mL) and desiccated overnight to yield compound **14** (189 mg, 0.261 mmol, 63.7%).

^{1}H-NMR (800 MHz, CD_3CN) δ: 9.60 (1H, d, TpA3), 8.08 (1H, d, TpB3), 7.88 (1H, d, TpB5), 7.80 (1H, d, TpC5), 7.64 (1H, d, TpA5), 7.48 (1H, d, TpC3), 6.40 (1H, t, TpB4), 6.22 (1H, t, TpC4), 6.13 (1H, t, TpA4), 4.17 (1H, dd, H4), 4.06 (1H, m, H3), 3.77 (2H, m, H11), 3.52 (1H, m, H9), 3.42 (2H, m, H12), 3.14 (1H, m, H9), 3.02 (1H, m, H8), 2.83 (1H, m H5), 2.60 (1H, m, H1), 2.54 (2H, m, H7), 2.52 (1H, m, H8), 2.21 (1H, m, H6), 2.17 (1H, m, H6), 1.42 (1H, td, H2), 1.11 (9H, d, PMe3). **^{13}C-NMR (201 MHz, CD_3CN) δ:** 173.3 (1C, C10), 150.0 (1C, TpA3), 145.0 (1C, TpB3), 142.4 (1C, TpC5), 138.1 (1C, TpB5), 137.0 (1C, TpA5), 136.3 (1C, TpC3), 107.6 (1C, TpB4), 106.7 (1C, TpC4), 106.1 (1C, TpA4), 66.3 (1C, C12), 63.0 (1C, C3), 61.1 (1C, C4), 57.0 (1C, C11), 51.6 (1C, C1), 47.9 (1C, C2), 43.1 (1C, C8), 36.1 (1C, C7), 36 (1C, C9), 33.8 (1C, C5), 31.9 (1C, C6), 13.4 (3C, d J= 28.1 Hz, PMe_3).

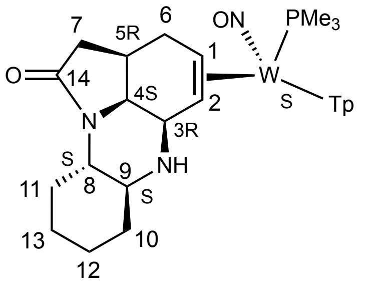

15

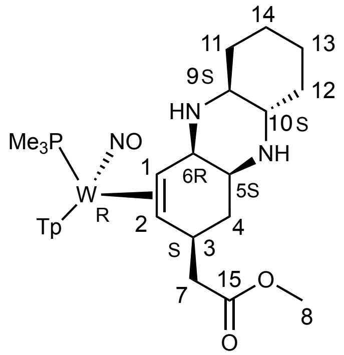

41

Compound 15 and **41:**

Compound **2** (100 mg, 0.136 mmol) was placed in a test tube, with EtCN, and chilled to -30 °C. After 10 min, a 1 M HOTf/EtCN (0.273 mL, 0.273 mmol) solution was added to the test tube and the solution was allowed to stir at -30 °C for 30 min. In a separate test tube, (1S,2S)-(+)-Cyclohexane-1,2-diamine (0.200 mL, 1.36 mmol) in EtCN was cooled at -30 °C for 30 min. Then, the former solution was added to the latter, dropwise. The reaction stirred at -30 °C for 1 d and then at 25 °C for 4 h. The reaction solution was washed three times (H_2O:Na_2CO_3/DCM; 60 mL/60mL) and dried with a golf-ball size amount of Na_2SO_4. The organic layer was evaporated in vacuo. The resulting yellow film was dissolved in minimal DCM and precipitated with pentane (50 mL), then loaded on a 60 mL medium-porosity frit filled two-thirds with basic alumina. Hexanes (150 mL) was eluted through the column, followed by diethyl ether (100 mL), ethyl acetate (150 mL) and methanol (200 mL).

The early fractions (ether and ethyl acetate) contained a mixture of isomers and were set aside. Over time, crystals of **41** spontaneously grew from the solution, which were collected (35.1 mg; 35% yield of a maximum theoretical yield of 50%). Meanwhile, the methanol portion eluted as a yellow band, which was evaporated to dryness, redissolved in minimal DCM, and then added to 50 mL of stirred pentane. An off-white solid precipitated out of the pentane, which was collected on a 15 mL fine-porosity fitted disk, washed with pentane (2 × 10 mL) and desiccated overnight to yield **15** (43 mg, 0.057 mmol, 41.9%; 42% yield out of a maximum of 50%).

Compound 15: ^{1}H-NMR (800 MHz, CD_3CN) δ: 8.69 (1H, d, TpA3), 8.01 (1H, d, TpB3), 7.85 (1H, d, TpA5), 7.81 (1H, d, TpB5), 7.70 (1H, d, TpC5), 7.36 (1H, d, TpC3), 6.36 (1H, t, TpB4), 6.23 (1H, t, TpC4), 6.20 (1H, t, TpA4), 4.35 (1H, t, H3), 4.17 (1H, m, H4), 2.98 (1H, m, H8), 2.83 (1H, m, H5), 2.72 (1H, m, H9), 2.66 (1H, m, H1), 2.63 (1H, d, H7), 2.50 (1H, d, H7), 2.23 (1H, m, H6), 2.15 (3H, m, H10/H11), 1.66 (2H, m, H12/H13), 1.26 (2H, m, H12/H13), 1.17 (9H, d, PMe3), 1.14 (1H, m, H11), 0.68 (1H, m, H2). **^{13}C-NMR (201 MHz, CD_3CN) δ**: 176.1 (1C, C14), 146.1 (1C, TpA3), 145.0 (1C, TpB3), 141.9 (1C, TpA5), 137.9 (1C, TpB5), 137.1 (1C, TpC5), 136.7 (1C, TpC3), 107.4 (1C, TpB4), 106.9 (1C, TpC4), 106.1 (1C, TpA4), 61.3 (1C, C8), 59.6 (1C, C3), 58.8 (1C, C4), 56.8 (1C, C2), 55.4 (1C, C9), 51.1 (1C, C1), 36.2 (1C, C7), 33.7 (1C, C5), 31.5 (1C, C6), 31.3 (1C, C10), 30.3 (1C, C11), 26.3 (1C, C12), 25.4 (1C, C13), 13.7 (3C, d J= 28.4 Hz, PMe_3).

Compound 41: ^{1}**H-NMR (800 MHz, CD_3CN) δ:** 8.27 (1H, d, TpA5), 8.02 (1H, d, TpB3), 7.84 (1H, d, TpB5), 7.82 (1H, d, TpC5), 7.79 (1H, d, TpA3), 7.39 (1H, d, TpC3), 6.37 (1H, t, TpB4), 6.26 (1H, t, TpC4), 6.24 (1H, t, TpA4), 3.82 (1H, d, H6), 3.64 (3H, s, H8), 3.56 (1H, m, H3), 3.16 (1H, dd, H9), 2.30 (2H, m, H7/H5), 2.24 (1H, m, H1), 2.05 (1H, t, H7), 1.96 (1H, m, H11), 1.72 (3H, m, H12/H14/H4), 1.53 (1H, m, H13), 1.33 (2H, m, H14/H3), 1.25 (2H, m, H11/H12), 1.14 (9H, d, PMe3), 1.05 (1H, m, H13), 0.72 (1H, dd, H2). ^{13}**C-NMR (201 MHz, CD_3CN) δ:** 174.5 (1C, C15), 144.1 (1C, TpA3), 142.2 (1C, TpB3), 141.6 (1C, TpB5), 137.9 (1C, TpC5), 137.5 (1C, TpA5), 137.1 (1C, TpC3), 107.5 (1C, TpB4), 107.1 (1C, TpC4), 106.6 (1C, TpA4), 63.6 (1C, C5), 61.9 (1C, C1), 61.3 (1C, C6), 55.6 (1C, C10), 54.7 (1C, C2), 52.6 (1C, C9), 51.8 (1C, C8), 46.5 (1C, C7), 40.1 (1C, C3), 34.0 (1C, C1), 33.3 (1C, C13), 32.8 (1C, C12), 26.4 (1C, C11), 26.1 (1C, C14), 13.2 (3C, d J= 28.8 Hz, PMe_3).

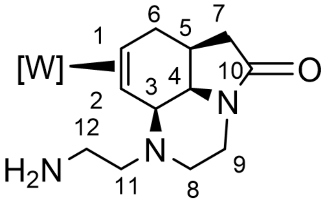

Compound 16:

Compound **2** (120 mg, 0.164 mmol) was placed in a test tube, with EtCN, and chilled to -20 °C. After 10 min, a 1 M HOTf/EtCN (0.327 mL, 0.327 mmol) solution was added to the test tube and the solution was allowed to stir at -20 °C for 60 min. In a separate test tube, N1-(aminomethyl)ethane-1,2-diamine (0.150 mL, 1.64 mmol) was cooled at -30 °C for 20 min. After the time elapsed, the former solution was added to the latter, dropwise. The reaction stirred at -30 °C for 3 days. A 20% KO^{t}Bu/THF solution (0.297 mL, 0.491 mmol) was added to quench the reaction. The reaction was washed three times (H_2O:Na_2CO_3/DCM; 30 mL/30mL) and dried with golf ball size Na_2SO_4. The organic layer was evaporated in vacuo. The resulting yellow film was dissolved in minimal DCM and pipetted in 50 mL of stirring pentane. An off-white solid precipitated out and was collected on a 15 mL fine-porosity fitted disk, washed with pentane (2 × 10 mL) and desiccated overnight to yield compound **16** (101.0 mg, 0.139 mmol, 85%).

^{1}**H-NMR (800 MHz, CD_3CN) δ**: 9.64 (1H, d, TpA3), 8.08 (1H, d, TpB3), 7.89 (1H, d, TpB5), 7.74 (1H, d, TpC5), 7.66 (1H, d, TpA5), 7.45 (1H, d, TpC3), 6.40 (1H, t, TpB4), 6.21 (1H, t, TpC4), 6.15 (1H, t, TpA4), 4.19 (1H, dd, H4), 4.04 (1H, m, H3), 3.51 (1H, m, H9), 3.12 (1H, m, H9), 3.02 (1H, m, H11), 3.01 (1H, m, H8), 2.95 (2H, m, H11/H12), 2.86 (1H, m, H12), 2.82 (1H, m H5), 2.60 (1H, m, H1), 2.54 (4H, m, H7/H8), 2.22 (1H, m, H6), 2.15 (1H, dd, H6), 1.40 (1H, m, H2), 1.10 (9H, d, PMe3). ^{13}**C-NMR (201 MHz, CD_3CN) δ**: 173.3 (1C, C10), 149.9 (1C, TpA3), 144.9 (1C, TpB3), 142.3 (1C, TpC3), 138.0 (1C, TpC5), 136.8 (1C, TpB5), 136.3 (1C, TpA5), 107.5 (1C, TpB4), 106.5 (1C, TpC4), 105.5 (1C, TpA4), 63.1 (1C, C3), 57.0 (1C, C4), 56.7 (1C, C11), 51.5 (1C, C1), 47.9 (1C, C2), 42.7 (1C, C8), 41.5 (1C, C12), 36.2 (1C, C7), 35.0 (1C, C9), 34.0 (1C, C5), 31.7 (1C, C6), 13.3 (3C, d J= 28.6 Hz, PMe_3). **CV (DMA):** $E_{p,a}$= 0.75 V (NHE). **IR (neat):** *v*(NO) 1542 cm^{-1}, *v*(CO) 1661 cm^{-1}, *v*(BH) 2486 cm^{-1}.

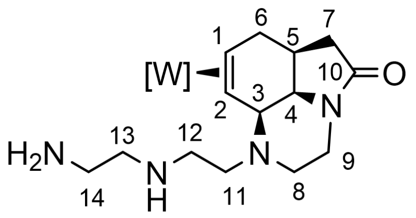

Compound 17:

Compound **2** (150 mg, 0.205 mmol) was placed in a test tube, with EtCN, and chilled to -20 °C. After 10 min, a 1 M HOTf/EtCN (0.410 mL, 0.410 mmol) solution was added to the test tube and the solution was allowed to stir at -20 °C for 60 min. In a separate test tube, N1,N1'-(ethane-1,2-diyl)bis(ethane-1,2-diamine) (0.610 mL, 4.09 mmol) was cooled at -30 °C for 20 min. After the time elapsed, the former solution was added to the latter, dropwise. The reaction stirred at -30 °C for 3 days. A 20% KO^{t}Bu/THF solution (0.354 mL, 0.614 mmol) was added to quench the reaction. The reaction was washed three times (H_2O:Na_2CO_3/DCM; 30 mL/30mL) and dried with golf ball size Na_2SO_4. The organic layer was evaporated in vacuo. The resulting yellow film was dissolved in minimal DCM and pipetted in 150 mL of stirring pentane. An off-white solid precipitated out and was collected on a 15 mL fine-porosity fitted disk, washed with pentane (2 × 10 mL) and desiccated overnight to yield compound **17** (108.0 mg, 0.141 mmol, 68.8%).

^{1}H-NMR (800 MHz, CD_2Cl_2) δ: 9.64 (1H, d, TpA3), 8.11 (1H, d, TpB3), 7.79 (1H, d, TpB5), 7.69 (1H, d, TpC5), 7.56 (1H, d, TpA5), 7.35 (1H, d, TpC3), 6.37 (1H, t, TpB4), 6.17 (1H, t, TpC4), 6.15 (1H, t, TpA4), 4.18 (1H, dd, H4), 4.10 (1H, dd, H3), 3.61 (1H, dd, H9), 3.18 (1H, m, H9), 3.15 (1H, m, H14), 3.12 (1H, m, H11), 2.90 (3H, m, H13/H5), 2.74 (3H, m, H8/H12), 2.63 (2H, m, H11/H14), 2.57 (2H, m, H7/H12), 2.55 (1H, m, H1), 2.52 (1H, m, H7), 2.28 (1H, td, H6), 2.14 (1H, dd, H6), 1.59 (1H, td, H2), 1.12 (9H, d, PMe3). **^{13}C-NMR (800 MHz, CD_2Cl_2) δ**: 173.0 (1C, C10), 149.7 (1C, TpB3), 144.0 (1C, TpA3), 141.4 (1C, TpB5), 137.1 (1C, TpC5), 136.0 (1C, TpA5), 135.4 (1C, TpC3), 106.9 (1C, TpB4), 105.8 (1C, TpC4), 105.2 (1C, TpA4), 62.6 (1C, C3), 56.8 (1C, C4), 52.7 (1C, C11), 51.3 (1C, C1), 49.5 (1C, C8), 48.8 (1C, C13), 47.8 (1C, C2), 42.2 (1C, C14), 41.9 (1C, C12), 36.2 (1C, C7), 34.8 (1C, C9), 33.3 (1C, C5), 31.6 (1C, C6), 13.2 (3C, d J= 28.5 Hz, PMe_3).

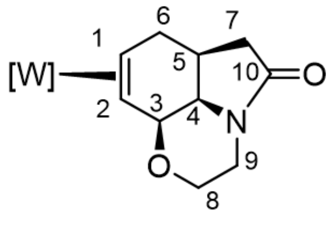

Compound 18:

Compound **23** (80.0 mg, 0.120 mmol) was placed in a test tube, with EtCN, and chilled to -20 °C. In a separate test tube, adenine (0.470 mg, 0.350 mmol) was cooled at -30 °C for 20 min with KO^{t}B$_u$ (0.140 mL, 0.203 mmol, 20% in THF). After 10 min, the former solution was added to the

latter, dropwise. The reaction was worked up after 10 min. This was washed three times (H_2O:Na_3PO_4/DCM; 30 mL/30mL) and dried with golf ball size Na_2SO_4. The organic layer was evaporated in vacuo. The resulting yellow film was dissolved in minimal DCM and pipetted in 30 mL of stirring pentane. An off-white solid precipitated out and was collected on a 15 mL fine-porosity fitted disk, washed with pentane (2 × 10 mL) and desiccated overnight to yield compound **18** (47 mg, 0.069 mmol, 59%).

^{1}H-NMR (800 MHz, CD_3CN) δ: 8.69 (1H, d, TpA3), 8.06 (1H, d, TpB3), 7.88 (1H, d, TpB5), 7.81 (1H, d, TpC5), 7.69 (1H, d, TpA5), 7.43 (1H, d, TpC3), 6.40 (1H, t, TpB4), 6.23 (1H, t, TpC4), 6.20 (1H, t, TpA4), 5.05 (1H, dt, H3), 3.74 (1H, m, H8), 3.54 (1H, m, H4), 3.33 (1H, m, H8), 3.17 (1H, m, H9), 2.93 (1H, m, H9), 2.70 (1H, , m, H1), 2.65 (1H, m, H5), 2.53 (1H, m, H6), 2.31 (1H, m, H7), 2.03 (1H, m, H6), 1.98 (1H, m, H7), 1.38 (1H, td, H2), 1.14 (9H, d, PMe3). **^{13}C-NMR (201 MHz, CD_3CN) δ**: 173.6 (1C, C10), 147.0 (1C, TpB3), 145.0 (1C, TpA3), 142.2 (1C, TpB5), 138.0 (1C, TpC5), 137.2 (1C, TpA5), 136.5 (1C, TpC3), 107.6 (1C, TpB4), 106.9 (1C, TpC4), 106.2 (1C, TpA4), 78.8 (1C, C3), 63.2 (1C, C4), 62.8 (1C, C1), 61.3 (1C, C2), 57.6 (1C, C8), 45.2 (1C, C9), 40.2 (1C, C7), 33.7 (1C, C5), 31.5 (1C, C6), 13.4 (3C, d J= 29.7 Hz, PMe_3).

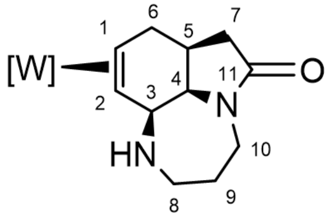

Compound 19:

Compound **2** (100 mg, 0.136 mmol) was placed in a test tube, with EtCN, and chilled to -20 °C. After 10 min, a 1 M HOTf/EtCN (0.273 mL, 0.273 mmol) solution was added to the test tube and the solution was allowed to stir at -30 °C for 60 min. In a separate test tube, diaminopropane (0.114 mL, 1.36 mmol) was cooled at -30 °C for 20 min in minimal EtCN. After the time elapsed, the former solution was added to the latter, dropwise. The reaction stirred at -30 °C for 3 days. A 2M NaO^{t}Bu/THF solution (0.205 mL, 0.409 mmol) was added to quench the reaction. The reaction was washed three times (H_2O:Na_3PO_4/DCM; 30 mL/30mL) and dried with golf ball size Na_2SO_4. The organic layer was evaporated in vacuo. The resulting yellow film was dissolved in minimal DCM and pipetted in 30 mL of stirring pentane. An off-white solid precipitated out and was collected on a 15 mL fine-porosity fitted disk, washed with pentane (2 × 10 mL) and desiccated overnight to yield compound **19** (74 mg, 0.11 mmol, 78%; 78-79%).

^{1}H-NMR (800 MHz, CD_3CN) δ: 8.11 (1H, d, TpB3), 8.09 (1H, d, TpA3), 7.86 (1H, d, TpB5), 7.86 (1H, d, TpC5), 7.76 (1H, d, TpA5), 7.43 (1H, d, TpC3), 6.39 (1H, t, TpB4), 6.29 (1H, t, TpA4), 6.26 (1H, t, TpC4), 3.96 (1H, dd, H4), 3.89 (1H, m, H10), 3.66 (2H, m, H3/H6), 3.10 (1H, m, H8), 2.92 (1H, m, H10), 2.60 (4H, m, H1/H5/H6H8), 2.56 (1H, m, H7), 1.94 (1H, m, H7), 1.71 (1H, m, H9), 1.51 (1H, m, H9), 1.21 (9H, d, PMe3), 1.17 (1H, d, H2). **^{13}C-NMR (201 MHz, CD_3CN) δ:** 175.2 (1C, C11), 145.0 (1C, TpB3), 144.6 (1C, TpA3), 142.0 (1C, TpC3), 137.3 (2C, TpB5/C5), 136.7

(1C, TpA5), 107.4 (1C, TpB4), 107.1 (1C, TpA4), 107.0 (1C, TpC4), 66.1 (1C, C3), 62.2 (1C, C4), 54.4 (1C, C2), 52.0 (1C, C1), 51.8 (1C, C8), 42.1 (1C, C10), 38.6 (2C, C6/C7), 29.2 (1C, C9), 28.2 (1C, C5), 13.7 (3C, d J= 29.9 Hz, PMe_3). **CV (DMA):** $E_{p,a}$= 0.78 V (NHE).

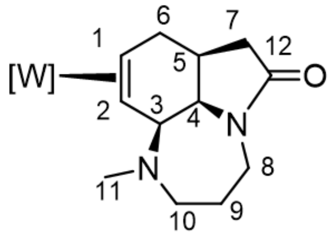

Compound 20:

Compound **2** (100 mg, 0.136 mmol) was placed in a test tube, with ACN, and chilled to -30 °C. After 10 min, a 1 M HOTf/ACN (0.273 mL, 0.273 mmol) solution was added to the test tube and the solution was allowed to stir at -30 °C for 30 min. In a separate test tube, N-Methyl-1,3-diaminopropane (0.064 mL, 061 mmol) was cooled at -30 °C for 30 min. After the time elapsed, the former solution was added to the latter, dropwise. The reaction stirred at -30 °C for 2 days and room temperature for 1 day. The reaction was washed three times (H_2O:Na_2CO_3/DCM; 30 mL/30mL) and dried with Na_2SO_4. The organic layer was evaporated in vacuo. The resulting yellow film was dissolved in minimal DCM and pipetted in 50 mL of stirring pentane. An off-white solid precipitated out and was collected on a 15 mL fine-porosity fitted disk, washed with pentane (2 × 10 mL) and desiccated overnight to yield compound **20** (82 mg, 0.12 mmol, 84%).

^{1}H NMR (800 MHz, CD_3CN) δ: 9.16 (d, 1H, TpA3), 8.07 (d, *J* = 2.0 Hz, 1H, TpB3), 7.87 (d, *J* = 2.4 Hz, 1H, TpB5), 7.80 (d, *J* = 2.3 Hz, 1H, TpC5), 7.67 (d, *J* = 2.3 Hz, 1H, TpA5), 7.42 (d, *J* = 2.2 Hz, 1H, TpC3), 6.38 (t, *J* = 2.2 Hz, 1H, TpB4), 6.24 (t, *J* = 2.2 Hz, 1H, TpC4), 6.19 (t, *J* = 2.2 Hz, 1H, TpA4), 4.41 (dd, *J* = 9.1, 3.0 Hz, 1H, H4), 3.78 (dd, *J* = 7.7, 3.0 Hz, 1H, H3), 3.27 (m, 2H, H8A/H8B), 2.89 (m, 1H, H10A), 2.71 (m, 2H, H5/H10B), 2.61 (m, 5H, H1/H7A/H11), 2.52 (m, 1H, H6A), 2.43 (m, 1H, H6B), 2.24 (dd, *J* = 17.1, 4.0 Hz, 1H, H7B), 1.88 (d, *J* = 12.6 Hz, 1H, H9A), 1.54 (m, 1H, H9B), 1.24 (m, 1H, H2), 1.12 (d, *J* = 8.3 Hz, 9H, PMe3).**^{13}C NMR (201 MHz, CD_3CN) δ**: 175.6 (1C, C12), 148.3 (1C, TpA3), 144.8 (1C, TpB3), 142.1 (1C, TpC3), 138.0 (1C, TpC5), 137.0 (1C, TpB5), 136.4 (1C, TpA5), 107.5 (1C, TpB4), 106.9 (1C, TpC4), 106.4 (1C, TpA4), 68.6 (1C, C3), 62.3 (1C, C4), 55.3 (1C, C10), 50.9 (1C, C1), 49.3 (1C, C2), 44.8 (1C, C8), 40.8 (1C, C11), 38.7 (1C, C7), 35.4 (1C, C6), 32.3 (1C, C5), 23.4 (1C, C9), 13.4 (3C, PMe3).

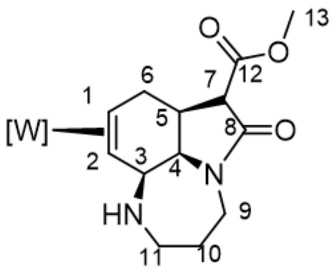

Compound 21:
Compound **4** (125 mg; 0.158 mmol) was placed in a test tube, with ACN (2 mL), and chilled to -30 °C. After 10 min, a 1 M HOTf/ACN (0.316 mL, 0.316) solution was added to the test tube and the solution was allowed to stir at -30 °C for 15 min. In a separate test tube, diaminopropane (0.133 mL; 1.58 mmol) in ACN (2 mL) was cooled at -30 °C for 30 min. Then, the former solution was added to the latter, dropwise. The reaction stirred at -30 °C for 3 days. The reaction was washed three times (H_2O:$NaHCO_3$/DCM; 60 mL/60mL) and dried over anhydrous Na_2SO_4. The organic layer was evaporated in vacuo. The resulting yellow film was dissolved in minimal DCM and pipetted in 50 mL of stirring pentane. Precipitation was induced twice to collect all the material. An off-white solid precipitated out and was collected on a 15 mL fine-porosity fritted disk, washed with pentane (2 × 10 mL) and desiccated overnight to yield compound **21** (105 mg, 0.140 mmol, 88%).

^{1}H NMR (800 MHz, CD_3CN) δ 8.12 (1H, d, J = 2.0 Hz, Tp3B), 8.09 (1H, d, J = 2.0 Hz, Tp3A), 7.87 (1H, d, J = 2.6 Hz, Tp5B), 7.84 (1H, d, J = 2.3 Hz, Tp5C), 7.76 (1H, d, J = 2.5 Hz, Tp5A), 7.43 (1H, d, J = 2.3 Hz, Tp3C), 6.38 (1H, t, J = 2.2 Hz, Tp4B), 6.29 (1H, t, J = 2.2 Hz, Tp4A), 6.26 (1H, t, J = 2.2 Hz, Tp4C), 4.04 (1H, dd, J = 9.5, 5.6 Hz, H4), 3.89 (1H, m, H9), 3.76 (1H, m, H6), 3.67 (1H, m, H3), 3.65 (3H, s, H13), 3.11 (1H, m, H11), 3.09 (1H, m, H7), 3.01 (1H, m, H9), 2.80 (1H, m, H5), 2.65 (1H, td, J = 12.9, 3.5 Hz, H11), 2.58 (1H, m, H6), 1.72 (1H, m, H10), 1.53 (1H, m, H10), 1.20 (9H, d, J = 8.4 Hz, PMe_3), 1.16 (1H, m, H2). **^{13}C NMR (201 MHz, CD_3CN) δ** 172.9 (1C, C12), 170.5 (1C, C8), 145.0 (1C, Tp3A), 144.7 (1C, Tp3B), 142.0 (Tp3C), 138.1 (1C, Tp5C), 137.3 (1C, Tp5B), 136.9 (1C, Tp5A), 107.5 (1C, Tp4B), 107.1 (1C, Tp4C), 65.7 (1C, C3), 61.3 (1C, C4), 56.9 (1C, H7), 53.9 (1C, C2), 52.7 (1C, C13), 51.7 (1C, C11), 51.3 (1C, d, J = 12.6 Hz, C1), 42.4 (1C, C9), 35.5 (1C, C6), 34.8 (1C, C5), 29.3 (1C, C10), 13.5 (3C, d, J = 28.9 Hz, PMe_3).

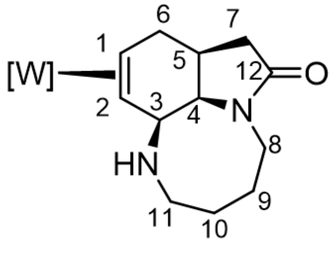

Compound 22:

Compound **2** (100 mg, 0.136 mmol) was placed in a test tube, with ACN, and chilled to -30 °C. After 10 min, a 1 M HOTf/ACN (0.273 mL, 0.273 mmol) solution was added to the test tube and the solution was allowed to stir at -30 °C for 60 min. In a separate test tube, diaminobutane (0.137 mL, 1.36 mmol) was cooled at -30 °C for 60 min in minimal ACN. After the time elapsed, the former solution was added to the latter, dropwise. The reaction stirred at -30 °C for 2 d and room temperature for 1 d. The reaction was washed three times (H_2O:Na_2CO_3/DCM; 30 mL/30mL) and dried with golf ball size Na_2SO_4. The organic layer was evaporated in vacuo. The resulting yellow film was dissolved in minimal DCM and pipetted in 30 mL of stirring pentane. An off-white solid precipitated out and was collected on a 15 mL fine-porosity fitted disk, washed with pentane (2 × 10 mL) and desiccated overnight to yield compound **22** (78 mg, 0.110 mmol, 81%).

^{1}H NMR (800 MHz, CD_3CN): δ 8.09 (s, 1H, TpB3), 7.92 (s, 1H, TpA3), 7.86 (d, 1H, TpB5), 7.83 (d, 1H, TpC5) 7.76 (s, 1H, TpA5), 7.39 (s, 1H, TpC3), 6.38 (s, 1H, TpB4), 6.31 (d, 1H, TpA4), 6.26 (d, 1H, TpC4), 3.96 (m, 1H, H8A), 3.91 (dd, 1H, H4), 3.63 (t, 1H, H6A), 3.43 (t, 1H, H3), 2.90 (m, 2H, H8B/H11A), 2.75 (dd, 1H, 6B), 2.65 (t, 1H, H1), 2.52 (m, 3H, H5/H7A/H11B), 2.09 (dd, 1H, H7B), 1.90 (m, 1H, H9A), 1.67 (m, 1H, H9B), 1.61 (m, 1H, H10A), 1.42 (m, H10B), 1.22 (d, 1H, H2), 1.19 (d, 9H, PMe3). **^{13}C NMR (201 MHz, CD_3CN):** δ 175.6 (1C, C12), 144.8 (1C, TpB3), 144.4 (1C, TpA3), 141.7 (1C, TpC3), 137.9 (1C, TpB5), 137.2 (1C, TpC5), 136.8 (1C, TpA5), 107.4 (1C, TpA4), 107.1 (1C, TpB4), 107.1 (1C, TpC4), 63.3 (1C, C4), 60.5 (1C, C3), 56.8 (1C, C2), 50.9 (1C, C1), 50.8 (1C, C11), 41.4 (1C, C8), 41.0 (1C, C7), 37.3 (1C, C6), 29.3 (1C, C10), 28.4 (1C, C5), 28.1 (1C, C9), 13.4 (3C, PMe3).

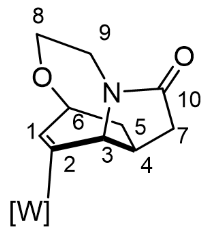

Compound 23:

Compound **2** (100 mg, 0.136 mmol) was placed in a test tube, with EtCN, and chilled to -20 °C. After 10 min, a 1 M HOTf/EtCN (0.273 mL, 0.273 mmol) solution was added to the test tube and the solution was allowed to stir at -60 °C for 60 min. In a separate test tube, 2-aminoethan-1-ol (0.165 mL, 2.73 mmol) with triethylamine (0.095 mL, 0.682 mmol) were cooled at -60 °C for 60 min. After the time elapsed, the former solution was added to the latter, dropwise. The reaction stirred at -60 °C for 3 days. A pipette-full of 20% KO^{t}Bu/THF solution was added to quench the reaction. A distinct color change was observed from yellow to light brown. The reaction was washed three times (H_2O:Na_2CO_3/DCM; 60 mL/60mL) and dried with golf ball size Na_2SO_4. The organic layer was evaporated in vacuo. The resulting yellow film was dissolved in minimal DCM and pipetted in 50 mL of stirring pentane. An off-white solid precipitated out and was collected on a 15 mL fine-porosity fitted disk, washed with pentane (2 × 10 mL) and desiccated overnight to yield compound **23** (72.0 mg, 0.110 mmol, 77.0%).

^{1}**H-NMR (800 MHz, CD_3CN) δ:** 8.06 (2H, d, TpB3/C5), 7.86 (2H, d, TpB5/C3), 7.78 (1H, d, TpA5), 7.49 (1H, d, TpA3), 6.28 (1H, t, TpB4), 6.29 (1H, t, TpA4), 6.26 (1H, t, TpC4), 4.69 (1H, d, H3), 3.60 (2H, m, H8), 3.58 (1H, t, H6), 3.17 (1H, m, H9), 3.03 (1H, m, H9), 2.61 (1H, m, H7), 2.52 (1H, m, H4), 2.47 (1H, m, H1), 2.20 (1H, d, H7), 2.05 (1H, m, H5), 1.23 (1H, m, H5), 1.19 (9H, d, PMe3), 1.15 (1H, d, H2). ^{13}**C-NMR (201 MHz, CD_3CN) δ:** 177.5 (1C, C10), 144.5 (1C, TpB3), 144.1 (1C, TpB5), 143.5 (1C, TpC5), 142.2 (1C, TpA3), 137.7 (1C, TpA5), 137.4 (1C, TpC3), 107.5 (1C, TpB4), 107.2 (1C, TpA4), 106.8 (1C, TpC4), 63.3 (1C, C3), 62.6 (1C, C8), 58.4 (1C, C6), 57.3 (1C, C1), 49.0 (1C, C2), 44.1 (1C, C9), 41.6 (1C, C7), 33.5 (1C, C5), 30.3 (1C, C4), 13.8 (3C, d J= 28.4 Hz, PMe_3). **CV (DMA)**: $E_{p,a}$= 1.2 V (NHE). **IR (neat):** *v*(NO) 1546 cm^{-1}, *v*(CO) 1649 cm^{-1}, *v*(BH) 2361 cm^{-1}.

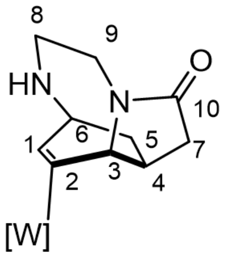

Compound 24:

Compound **2** (100 mg, 0.136 mmol) was placed in a test tube, with EtCN, and chilled to -20 °C. After 10 min, a 1 M HOTf/EtCN (0.273 mL, 0.273 mmol) solution was added to the test tube and the solution was allowed to stir at -60 °C for 60 min. In a separate test tube, ethylenediamine (0.182 mL, 2.73 mmol) in propionitrile at -60 °C for 60 min. After the time elapsed, the former solution was added to the latter, dropwise. The reaction stirred at -60 °C for 3 days. A pipette-full of 20% KO^tBu/THF solution was added to quench the reaction. A distinct color change was observed from yellow to light brown. The reaction was washed three times (H_2O:Na_2CO_3/DCM; 60 mL/60mL) and dried with golf ball size Na_2SO_4. The organic layer was evaporated in vacuo. The resulting yellow film was dissolved in minimal DCM and pipetted in 50 mL of stirring pentane. An off-white solid precipitated out and was collected on a 15 mL fine-porosity fitted disk, washed with pentane (2 × 10 mL) and desiccated overnight to yield compound **24** (37 mg, 0.054 mmol, 40%) and **5** (39 mg, 0.057 mmol, 44%) in 9:10 ratio.

Assignments for compound 24.

^{1}**H-NMR (800 MHz, CD_3CN) δ**: 8.05 (2H, d, TpB3/C5), 7.85 (2H, d, TpB5/C3), 7.81 (1H, d, TpA5), 7.42 (1H, d, TpA3), 6.37 (1H, t, TpB4), 6.29 (1H, t, TpA4), 6.22 (1H, t, TpC4), 4.72 (1H, d, H3), 3.60 (1H, bs, H8), 3.15 (1H, m, H9), 2.88 (1H, m, H9), 2.65 (1H, m, H7), 2.51 (1H, m, H4), 2.33 (1H, m, H1), 2.19 (1H, d, H7), 1.23 (1H, m, H5), 1.19 (9H, d, PMe3), 1.09 (1H, d, H2).

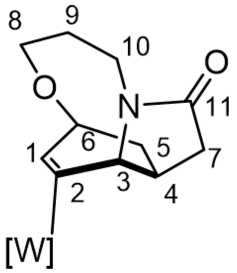

Compound 25:

Compound **2** (110 mg, 0.150 mmol) was placed in a test tube, with ACN, and chilled to -60 °C. After 10 min, a 1 M HOTf/ACN (0.300 mL, 0.300 mmol) solution was added to the test tube and the solution was allowed to stir at -60 °C for 60 min. In a separate test tube, propanolamine (0.229 mL, 3.00 mmol) with triethylamine (0.083 mL, 0.600 mmol) were cooled at -60 °C for 60 min. After the time elapsed, the former solution was added to the latter, dropwise. The reaction stirred at -60 °C for 3 days. A pipette-full of 20% KO^tBu/THF solution was added to quench the reaction. A distinct color change was observed from yellow to light brown. The reaction was washed three times (H_2O:Na_2CO_3/DCM; 60 mL/60mL) and dried with golf ball size Na_2SO_4. The organic layer was evaporated in vacuo. The resulting yellow film was dissolved in minimal DCM and pipetted in 50 mL of stirring pentane. An off-white solid precipitated out and was collected on a 15 mL fine-porosity fitted disk, washed with pentane (2 × 10 mL) and desiccated overnight to yield compound **25** (56 mg, 0.0.080 mmol, 54.0%).

^{1}H NMR (800 MHz, CD_3CN): δ 8.08 (d, 1H, TpB3), 8.05 (d, 1H, TpA3), 7.86 (d, 1H, 7.85), 7.85 (d, 1H, TpC5), 7.79 (d, 1H, TpB5), 7.46 (d, 1H, TpC3), 6.37 (t, 1H, TpA4), 6.29 (t, 1H, TpC4), 6.25 (t, 1H, TpB4), 4.70 (d, 1H, C3), 3.63 (m, 2H, H8A/H8B), 3.60 (m, 1H, H6), 3.16 (m, 1H, H10A), 3.05 (m, 1H, H10B), 2.61 (dd, 1H, H7A) , 2.50 (t, 1H, H4), 2.46 (t, 1H, H1), 2.23 (d, 1H, H7B), 1.94 (m, 1H, H5A), 1.68 (m, 2H, H9A/H9B), 1.22 (d, 9H, PMe3), 1.17 (m, 1H, H5B), 1.09 (d, 1H, H2).**^{13}C-NMR (201 MHz, CD_3CN) δ** 177.4 (1C, C11), 144.6 (1C, TpB3), 143.4 (1C, TpB5), 142.1 (1C, TpC5), 138.0 (1C, TpA3), 137.8 (1C, TpA5), 137.4 (1C, TpC3), 107,5 (1C, TpB4), 107.2 (1C, TpA4), 106.8 (1C, TpC4), 62.7 (1C, C3), 62.1 (1C, C8), 58.9 (1C, C6), 57.0 (1C, C1), 48.7 (1C, C2), 40.6 (1C, C7), 36.8 (1C, C10), 34.2 (1C, C9), 33.5 (1C, C5), 31.6 (1C, C4), 14.0 (3C, PMe3).

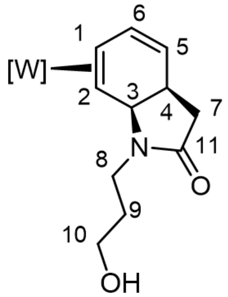

Compound 26:

Compound **2** (100mg, 0.136 mmol) was placed in a test tube with ACN, and chilled to -30 °C. After 10 min, a 1 M HOTf/ACN (0.273 mL, 0.273 mmol) solution was added to the test tube and the solution was allowed to stir at -30 °C for 30 min. In a separate test tube, propanolamine (0.104 mL, 2.73 mmol) was cooled at -30 °C for 30 min. After the time elapsed, the former solution was added to the latter, dropwise. The reaction stirred at -30 °C for 48 hrs and room temperature for 24 hrs. The reaction was washed three times (H_2O:Na_2CO_3/DCM; 30 mL/30mL) and dried with Na_2SO_4. The organic layer was evaporated in vacuo. The resulting yellow film was dissolved in minimal DCM and pipetted in 25 mL of stirring hexane. A white solid precipitated out and was collected on a 15 mL fine-porosity fitted disk, washed with hexane (2 × 10 mL) and desiccated overnight to yield compound **26** (75.0 mg, 0.108 mmol, 79.0%).

^{1}H NMR (800 MHz, CD_2Cl_2) δ: 8.06 (d, *J* = 2.0 Hz, 1H, TpB3), 7.99 (d, *J* = 2.0 Hz, 1H, TpA5), 7.78 (d, *J* = 2.3 Hz, 1H, TpC5), 7.77 (d, *J* = 2.5 Hz, 1H, TpB5), 7.69 (d, *J* = 2.4 Hz, 1H, TpA3), 7.36 (d, *J* = 2.1 Hz, 1H, TpC3), 6.44 (ddd, *J* = 9.8, 5.2, 2.4 Hz, 1H, H6), 6.35 (t, *J* = 2.2 Hz, 1H, TpB4), 6.27 (t, *J* = 2.2 Hz, 1H, TpC4), 6.26 (t, *J* = 2.2 Hz, 1H, TpA4), 4.64 (m, 2H, H3/H5), 3.50 (ddd, *J* = 14.1, 11.1, 6.7 Hz, 2H, H8A/H10A), 3.41 (d, *J* = 9.9 Hz, 1H, H10B), 3.30 (t, *J* = 7.4 Hz, 1H, H4), 3.18 (ddd, *J* = 14.3, 6.3, 4.4 Hz, 1H, H8B), 2.71 (m, 2H, H1/H7A), 2.10 (d, *J* = 16.3 Hz, 1H, H7B), 1.60 (ddt, *J* = 13.7, 9.1, 4.4 Hz, 1H, H9A), 1.45 (tdd, *J* = 8.9, 7.4, 4.1 Hz, 1H, H9B), 1.41 (d, *J* = 9.5 Hz, 1H, H2), 1.21 (d, *J* = 8.4 Hz, 9H, PMe_3). **^{13}C NMR (201 MHz, CD_2Cl_2) δ**: 177.2 (1C, C11), 143.8 (1C, TpB3), 142.7 (1C, TpA5), 140.8 (1C, TpC3), 137.2 (1C, TpC5), 136.5 (1C, TpA3), 136.3 (1C, TpB5), 131.3 (d, *J* = 3.5 Hz, 1C, C6), 120.0 (1C, C5), 106.9 (1C, TpB4), 106.5 (1C, TpC4), 106.1 (1C, TpA4), 64.1 (1C, C3), 58.2 (1C, C10), 49.6 (d, *J* = 10.1 Hz, 1C, C1), 47.9 (1C, C2), 39.9 (1C, C7), 36.4 (1C, C8), 33.0 (1C, C4), 32.1 (1C, C9), 13.8 (d, *J* = 28.2 Hz, 1C, PMe_3).

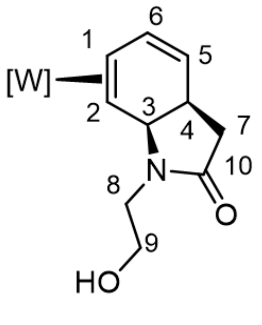

Compound 27:

Compound **2** (200mg, 0.273 mmol) was placed in a test tube with ACN, and chilled to -30 °C. After 10 min, a 1 M HOTf/ACN (0.546 mL, 0.546 mmol) solution was added to the test tube and the solution was allowed to stir at -30 °C for 30 min. In a separate test tube, ethanolamine (0.165 mL, 2.73 mmol) was cooled at -30 °C for 30 min. After the time elapsed, the former solution was added to the latter, dropwise. The reaction stirred at -30 °C for 48 hrs and room temperature for 24 hrs. The reaction was washed three times (H_2O:Na_2CO_3/DCM; 30 mL/30mL) and dried with Na_2SO_4. The organic layer was evaporated in vacuo. The resulting yellow film was dissolved in minimal DCM and pipetted in 25 mL of stirring hexane. A white solid precipitated out and was

collected on a 15 mL fine-porosity fitted disk, washed with hexane (2 × 10 mL) and desiccated overnight to yield compound **27** (157 mg, 0.230 mmol, 84.4%).

^{1}H NMR (800 MHz, CD_3CN) δ: 8.07 (dd, *J* = 5.7, 2.1 Hz, 2H, TpA5/B3), 7.86 (m, 2H, TpA3/C5), 7.78 (d, *J* = 2.3 Hz, 1H, TpB5), 7.50 (d, *J* = 2.2 Hz, 1H, TpC3), 6.46 (ddd, *J* = 9.8, 4.7, 2.2 Hz, 1H, H6), 6.38 (t, 1H, TpA4), 6.31 (t, 1H, TpC4), 6.28 (t, 1H, TpB4), 4.71 (d, *J* = 6.1 Hz, 1H, H3), 4.52 (dd, *J* = 9.7, 2.0 Hz, 1H, H5), 3.40 (t, *J* = 6.1 Hz, 2H, H9A/9B), 3.32 (dt, *J* = 14.0, 5.8 Hz, 1H, H8A), 3.25 (m, 2H, H8B, H4), 2.80 (m, 1H, H1), 2.66 (t, *J* = 5.5 Hz, 1H, H7A), 1.92 (m, 1H, H7B), 1.33 (d, *J* = 9.6 Hz, 1H, H2), 1.18 (d, *J* = 8.6 Hz, 9H, PMe_3) . **^{13}C NMR (201 MHz, CD_3CN) δ:** 176.9 (1C, C10), 144.6 (1C, TpA5), 143.7 (1C, TpB3), 142.2 (1C, TpC3), 138.1 (1C, TpC5), 137.4 (1C, TpA3), 137.3 (1C, TpB5), 132.2 (1C, C6), 120.2 (1C, C5), 107.6 (1C, TpC4), 107.3 (1C, TpA4) , 106.8 (1C, TpB4) , 63.6 (1C, C3), 61.5 (1C, C9), 49.5 (d, *J* = 9.9 Hz, 1C, C1), 48.3 (1C, C2), 43.4 (1C, C8), 40.1 (1C, C7), 33.5 (1C, C4), 13.7 (d, *J* = 28.7 Hz, 1C, PMe_3).

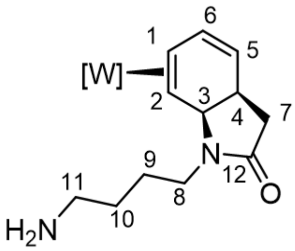

Compound 28:

Compound **2** (50 mg, 0.068 mmol) was placed in a test tube, with CH_3CN, and chilled to -30 °C. After 10 min, a 1 M HOTf/CH_3CH_2CN (0.136 mL, 0.136 mmol) solution was added to the test tube and the solution was allowed to stir at -30 °C for 10 min. In a separate test tube, 1,4-diaminobutane (0.069 mL, 0.68 mmol) was cooled at -30 °C for 10 min. After the time elapsed, the former solution was added to the latter, dropwise. The reaction was washed three times (H_2O:Na_2CO_3/DCM; 30 mL/30mL) and dried with Na_2SO_4. The clear solution was evaporated in vacuo. The resulting film was dissolved in minimal DCM and pipetted in 15 mL of stirring pentane. A tan/white solid precipitated out and was collected on a 15 mL fine-porosity fritted disk, washed with hexane (2 × 10 mL) and desiccated overnight to yield compound **28** (42.0 mg, 0.059 mmol, 87%).

^{1}H-NMR (800 MHz, $(D_3C)_2CO$) δ: 8.18 (1H, d, TpA3), 8.15 (1H, d, TpB3), 7.97 (2H, t, TpB5/C5), 7.84 (1H, d, TpA5), 7.67 (1H, d, TpC3), 6.41 (2H, m, H6/TpB4), 6.35 (1H, t, TC4), 6.30 (1H, t, TpA4), 4.75 (1H, d, H3), 4.51 (1H, dd, H5), 3.38 (1H, d, H11), 3.32 (1H, t, H4), 3.20 (1H, m, H8), 3.11 (1H, m, H11), 3.07 (1H, m, H8), 2.85 (1H, m, H1), 2.62 (1H, m, H7), 1.92 (1H, d, H7), 1.62 (2H, m, H9), 1.44 (1H, d, H2), 1.41 (2H, m, H10), 1.27 (9H, d, PMe_3).**^{13}C-NMR (201 MHz, $(D_3C)_2CO$) δ:** 174.7 (1C, C9), 144.5 (1C, TpB3) 143.4 (1C, TpA3), 142.0 (2C, TpB5/A5), 137.1 (1C, TpC3), 136.9 (1C, TpC5), 131.8 (1C, C6), 120.6 (1C, C5), 107.3 (1C, TpB4), 107.1 (1C, TpC4), 106.5 (1C, TpA4), 62.5 (1C, C3), 51.7 (1C, C8), 49.6 (1C, d, C1), 48.2 (1C, C2), 40.3 (1C, s, C7), 39.6 (1C, C11), 32.8 (1C, s, C4), 28.6 (1C, C9), 26.9 (1C, C10) 13.7 (3C, d, PMe_3).

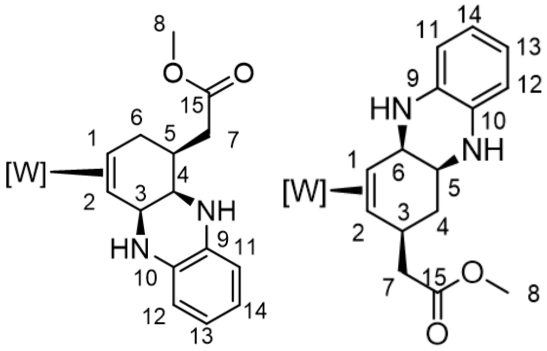

Compound 29 and 31:

Compound **2** (150 mg, 0.205 mmol) was placed in a test tube, with ACN, and chilled to -30 °C. After 10 min, a 1 M HOTf/ACN (0.409 mL, 0.409 mmol) solution was added to the test tube and the solution was allowed to stir at -30 °C for 30 min. In a separate test tube, o-Phenylenediamine (221 mg, 2.05 mmol) was cooled at -30 °C for 30 min in minimal THF. After the time elapsed, the former solution was added to the latter, dropwise. The reaction stirred at -30 °C for 72 hours. The reaction was washed three times (H_2O:Na_3PO_4/DCM; 30 mL/30mL) and dried with golf ball size Na_2SO_4. The organic layer was evaporated in vacuo. The resulting yellow film was dissolved in minimal DCM and pipetted in 20 mL of stirring pentane. An off-white solid precipitated out and was collected on a 15 mL fine-porosity fitted disk, washed with pentane (2 × 10 mL) and desiccated overnight to yield compounds **29** and **31** in a 0.25:1 ratio (112 mg, 0.147 mmol, 72%).

Assignments given for the major isomer (**31**). **^{1}H-NMR (800 MHz, CD_3CN) δ**: 8.27 (1H, d, TpA5), 8.03 (1H, d, TpB3), 7.86 (1H, d, TpB5), 7.85 (1H, d, TpC5), 7.80 (1H, d, TpA3), 7.49 (1H, d, TpC3), 6.47 (1H, m, H11), 6.42 (2H, m, H12/H13), 6.40 (1H, m, H14), 6.38 (1H, t, TpB4), 6.30 (1H, t, TpC4), 6.27 (1H, t, TpA4), 4.22 (1H, d, H6), 3.67 (2H, m, H3/H5), 3.61 (3H, s, H8), 2.40 (1H, m, H1), 2.29 (1H, m, H7), 2.03 (1H, m, H7), 1.39 (2H, m, H4), 1.12 (9H, d, PMe3), 0.78 (1H, m, H2). **^{13}C-NMR (201 MHz, CD_3CN) δ:** 174.3 (1C, C15), 144.3 (1C, TpB3), 142.2 (1C, TpA5), 141.7 (1C, TpC3), 138.2 (1C, TpB5), 137.6 (1C, TpC5), 137.4 (1C, TpA3), 118.4 (2C, C13/C14), 114.2 (C10/C12), 113.9 (2C, C9/C11), 107.7 (1C, TpB4), 107.1 (1C, TpC4), 106.8 (1C, TpA4), 62.1 (1C, C1), 55.7 (1C, C4), 53.1 (1C, C2), 52.1 (1C, C8), 49.5 (1C, C3), 46.3 (1C, C7), 39.5 (1C, C5), 36.6 (1C, C6), 13.7 (3C, d J= 29.9 Hz, PMe_3). **CV (DMA):** $E_{p,a}$= 0.74 V (NHE). **IR (neat):** *v*(NO) 1558 cm^{-1}, *v*(CO) 1732 cm^{-1}, *v*(BH) 2503 cm^{-1}.

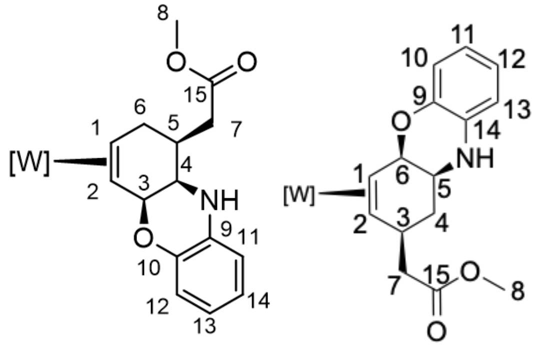

Compound 30 and 32:

Compound **2** (150mg, 0.205 mmol) was placed in a test tube with ACN, and chilled to -30 °C. After 10 min, a 1 M HOTf/ACN (0.410 mL, 0.410 mmol) solution was added to the test tube and the solution was allowed to stir at -30 °C for 30 min. In a separate test tube, 2-Aminophenol (224 mg, 2.05 mmol) was cooled at -30 °C for 30 min in THF. After the time elapsed, the former solution was added to the latter, dropwise. The reaction stirred at -30 °C for 72 hrs. The reaction was washed three times (H_2O:Na_2CO_3/DCM; 30 mL/30mL) and dried with Na_2SO_4. The organic layer was evaporated in vacuo. The resulting brown film was desiccated for 1hr then dissolved in minimal acetonitrile. After 24 hrs, crystals were removed from the solution via vacuum filtration through a 15ml fine-porosity fitted-disk. The filtrate was evaporated to dryness, dissolved in minimal DCM and pipetted in 25 mL of stirring hexane. An off-white solid precipitated out and was collected on a 15 mL fine-porosity fitted disk, washed with hexane (2 × 10 mL) and desiccated overnight to yield compound **30** and **32** in a 0.25:1 ratio (96.0 mg, 0.130 mmol, 61%).

Assignments given for the major isomer (**32**). **^{1}H NMR (800 MHz, CD_3CN) δ**: 8.25 (d, *J* = 2.1 Hz, 1H, TpA3), 8.04 (d, *J* = 2.0 Hz, 1H, TpB3), 7.88 (d, *J* = 2.3 Hz, 1H, TpC5), 7.86 (d, *J* = 2.3 Hz, 1H, TpB5), 7.82 (d, *J* = 2.3 Hz, 1H, TpA5), 7.50 (d, *J* = 2.2 Hz, 1H, TpC3), 6.73 (dd, *J* = 7.8, 1.4 Hz, 1H, H11), 6.39 (t, *J* = 2.2 Hz, 1H, TpB4), 6.29 (dt, *J* = 4.1, 2.2 Hz, 2H, TpA4/C4), 6.73 (d, 1H, H10), 6.68 (t, 1H, H12), 6.54 (M, 2H, H11/H13), 4.79 (d, *J* = 2.5 Hz, 1H, H6), 3.83 (d, *J* = 12.4 Hz, 1H, H5), 3.65 (m, 1H, H3), 3.61 (s, 3H, H8), 2.71 (t, *J* = 11.1 Hz, 1H, H1), 2.31 (dd, *J* = 14.7, 3.4 Hz, 1H, H7A), 2.06 (m, 1H, H7B), 1.54 (dt, *J* = 11.6, 4.0 Hz, 1H, H4A), 1.41 (m, 1H, H4B), 1.12 (d, *J* = 8.4 Hz, 9H, PMe_3), 0.84 (ddd, *J* = 11.3, 3.1, 2.3 Hz, 1H, H2). **^{13}C NMR (201 MHz, CD_3CN) δ:** 174.2 (1C, C15), 143.3 (1C, TpB3), 141.3 (1C, TpA3), 140.9 (1C, TpC3), 137.2 (1C, TpA5), 136.7 (1C, TpC5), 136.4 (1C, TpB5), 133.8 (1C, C14), 121.0 (1C, C12), 116.5 (1C, C11), 115.6 (1C, C13), 113.9 (1C, C10), 79.0 (d, *J* = 4.5 Hz, 1C, C6), 58.5 (d, *J* = 10.8 Hz, 1C, C1), 53.3, (1C, C2) , 51.9 (1C, C8) , 49.3 (1C, C5), 46.0 (1C, C7), 38.8 (1C, C3), 36.1 (1C, C4), 12.9 (d, *J* = 28.8 Hz, 3C, PMe_3).

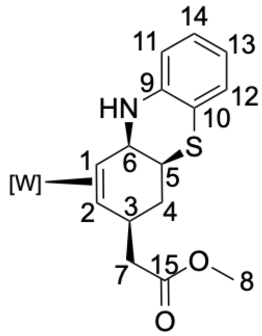

Compound 33:

Compound **2** (120 mg, 0.164 mmol) was placed in a test tube, with ACN, and chilled to -30 °C. After 10 min, a 1 M HOTf/ACN (0.327 mL, 0.327 mmol) solution was added to the test tube and the solution was allowed to stir at -30 °C for 30 min. In a separate test tube, 2-aminothiophenol (0.175 mL, 1.64 mmol) was cooled at -30 °C for 30 min in THF. After the time elapsed, the former solution was added to the latter, dropwise. The reaction stirred at -30 °C for 3 days. The reaction was washed three times (H_2O:Na_2CO_3/DCM; 30 mL/30mL) and dried with $MgSO_4$. The solution was evaporated in vacuo. The resulting film was dissolved in minimal DCM and pipetted in 15 mL of stirring hexane. An off-white solid precipitated out and was collected on a 15 mL fine-porosity fritted disk, washed with hexane (2 × 10 mL) and desiccated overnight to yield compound **33** (74.0 mg, 0.095 mmol, 58.0%).

^{1}H NMR (800 MHz, CD_3CN) δ: 8.26 (d, J = 2.1 Hz, 1H, TpA3), 8.05 (d, J = 2.0 Hz, 1H, TpB3), 7.88 (d, J = 2.3 Hz, 1H, TpB5), 7.86 (d, J = 2.3 Hz, 1H, TpC5), 7.81 (d, J = 2.4 Hz, 1H, TpA5), 7.52 (d, J = 2.2 Hz, 1H, TpC3), 6.94 (dd, J = 7.7, 1.5 Hz, 1H, H11), 6.89 (m, 1H, H13), 6.60 (dd, J = 8.1, 1.5 Hz, 1H, H14), 6.53 (m, 1H, H12), 6.40 (t, J = 2.2 Hz, 1H, TpB4), 6.33 (t, J = 2.2 Hz, 1H, TpC4), 6.29 (t, J = 2.2 Hz, 1H, TpA4), 4.43 (d, J = 3.7 Hz, 1H, H6), 3.78 (s, 1H, H3), 3.59 (s, 4H, H8/H5), 2.48 (t, J = 11.0 Hz, 1H, H1), 2.31 (d, J = 3.5 Hz, 1H, H7A), 2.07 (d, J = 3.1 Hz, 1H, H7B), 1.68 (m, 1H, H4A), 1.58 (q, J = 12.1 Hz, 1H, H4B), 1.12 (d, J = 8.4 Hz, 9H, PMe3), 0.82 (dt, J = 11.2, 2.5 Hz, 1H, H2). **^{13}C NMR (201 MHz, CD_3CN) δ**: 174.0 (1C, C15), 144.4 (1C, TpB3), 142.3 (1C, TpA3), 141.7 (1C, TpC3), 138.2 (1C, C10), 137.7 (1C, TpA5), 137.4 (1C, TpC5), 137.1 (1C, TpB5), 128.0 (1C, C11), 126.2 (1C, C13), 118.3 (1C, C12), 115.0 (1C, C14), 107.7 (1C, TpA4), 107.3 (1C, TpB4), 106.8 (1C, TpC4), 66.3 (1C, C1), 55.3 (1C, C6), 52.0 (1C,C2), 51.9 (1C, C8), 46.4 (1C, C7), 39.9 (1C, C3), 36.6 (2C, C5/4), 13.1 (d, J = 28.6 Hz, 3C, PMe3).

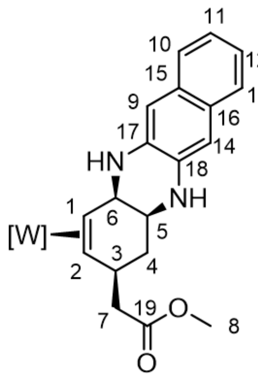

Compound 34:

Compound **2** (150 mg, 0.205 mmol) was placed in a test tube, with ACN, and chilled to -30 °C. After 10 min, a 1 M HOTf/ACN (0.409 mL, 0.409 mmol) solution was added to the test tube and the solution was allowed to stir at -30 °C for 30 min. In a separate test tube, naphthalene-2,3-diamine (324 mg, 2.05 mmol) in MeOH (2 mL) was cooled at -30 °C for 30 min. Then, the former solution was added to the latter, dropwise. The reaction stirred at -30 °C for 3 days and at room temperature for a day. The reaction was removed from the glovebox and loaded onto a silica column with 2ml DCM. The column was eluted with 100ml hexanes, 400ml diethyl ether and 200ml ethyl acetate. The ethyl acetate portion was evaporated in vacuo. The resulting yellow film was dissolved in minimal DCM and pipetted in 50 mL of stirring pentane. Precipitation was induced twice to collect all the material. An off-white solid precipitated out and was collected on a 15 mL fine-porosity fritted disk, washed with pentane (2 × 10 mL) and desiccated overnight to yield compound **34** (67.0 mg, 0.083 mmol, 40.0%).

^{1}H-NMR (800 MHz, CD_3CN) δ: 8.28 (1H, d, TpA3), 8.05 (1H, d, TpB3), 7.87 (1H, d, TpB5), 7.86 (1H, d, TpC5), 7.81 (1H, d, TpA3), 7.73 (1H, dd, H10), 7.64 (1H, d, H13), 7.52 (1H, d, TpC3), 7.43 (1H, dd, H12), 7.2408 (1H, dd, H11), 6.77 (1H, s, H9), 6.68 (1H, s, H14), 6.39 (1H, t, TpB4), 6.33 (1H, t, TpC4), 6.28 (1H, t, TpA4), 4.36 (1H, d, H6), 3.81 (1H, m, H5), 3.70 (1H, dd, H3), 3.60 (3H, s, H8), 2.45 (1H, t, H1), 2.33 (1H, m, H7), 2.05 (1H, d, H7), 1.55 (1H, m, H4), 1.45 (1H, m, H4), 1.16 (9H, d, PMe3), 0.82 (1H, m, H2). **^{13}C-NMR (201 MHz, CD_3CN) δ:** 174.3 (1C, C19), 142.2 (1C, TpB3), 142.3 (1C, TpA5), 140.5 (1C, TpC3), 137.1 (1C, TpB5), 136.4 (1C, TpC5), 137.4 (1C, TpA3), 136.7 (1C, C16), 136.1 (1C, C15), 128.7 (1C, C18), 128.1 (1C, C17), 126.9 (1C, C13), 125.7 (1C, C12), 124.6 (1C, C11), 128.0 (1C, C10), 107.8 (1C, TpB4), 107.2 (1C, TpC4), 106.7 (1C, TpA4), 106.5 (1C, C9), 105.5 (1C, C14), 61.2 (1C, C1), 53.7 (1C, C6), 52.8 (1C, C2), 49.1 (1C, C5), 46.0 (1C, C7), 39.1(1C, C3), 36.5 (1C, C4), 13.7 (3C, d J= 29.6 Hz, PMe_3).

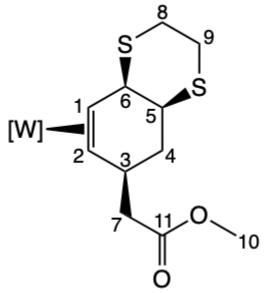

Compound 35:

Compound **2** (100 mg, 0.136 mmol) was placed in a test tube with ACN, and chilled to -30 °C. After 10 min, a 1 M HOTf/ACN (0.273 mL, 0.273 mmol) solution was added to the test tube and the solution was allowed to stir at -30 °C for 30 min. In a separate test tube, Ethane-1,2-dithiol (0.115 ml, 1.37 mmol) and Triethylamine (0.076 ml, 0.545 mmol) were cooled at -30 °C for 30 min in MeOH. After the time elapsed, the former solution was added to the latter, dropwise. The reaction stirred at -30 °C for 5 hrs and room temperature for 24 hrs. The reaction was washed three times (H_2O:Na_2CO_3/DCM; 30mL/30mL) and dried with Na_2SO_4. The organic layer was evaporated in vacuo. The resulting yellow film was dissolved in minimal DCM and pipetted in 25 mL of stirring hexane. An off-white solid precipitated out and was collected on a 15 mL fine-porosity fitted disk, washed with hexane (2 × 10 mL) and desiccated overnight to yield compound **35** (49.0 mg, 0.066 mmol, 48%, 41-48%).

^{1}H NMR (800 MHz, CD_3CN) δ: 8.25 (d, *J* = 2.1 Hz, 1H, TpA3), 8.03 (d, *J* = 2.0 Hz, 1H, TpB3), 7.86 (d, *J* = 2.4 Hz, 1H, TpB5), 7.83 (d, *J* = 2.3 Hz, 1H, TpC5), 7.79 (d, *J* = 2.4 Hz, 1H, TpA5), 7.34 (d, *J* = 2.2 Hz, 1H, TpC3), 6.38 (t, *J* = 2.2 Hz, 1H, TpB4), 6.27 (t, *J* = 2.3 Hz, 1H, TpA4), 6.24 (t, *J* = 2.3 Hz, 1H, TpC4), 4.44 (d, *J* = 3.0 Hz, 1H, H6), 3.82 (s, 1H, H3), 3.64 (s, 3H, H10), 3.27 (d, *J* = 12.5 Hz, 1H, H5), 3.16 (m, 1H, H8A), 2.99 (ddd, *J* = 14.3, 12.2, 2.4 Hz, 1H, H9A), 2.83 (d, *J* = 13.6 Hz, 1H, H8B), 2.50 (q, *J* = 12.0 Hz, 1H, H4A), 2.46 (ddd, *J* = 13.8, 4.1, 2.4 Hz, 1H, H9B), 2.31 (dd, *J* = 14.6, 3.5 Hz, 1H, H7A), 2.20 (t, *J* = 11.2 Hz, 1H, H1), 2.13 (Hidden, 1H, H7B), 1.74 (dt, *J* = 10.8, 3.2 Hz, 1H, H4B), 1.17 (d, *J* = 8.4 Hz, 9H, PMe3), 0.72 (dt, *J* = 11.3, 2.6 Hz, 1H, H2).**^{13}C NMR (201 MHz, CD_3CN) δ:** 174.1 (1C, C11), 144.3 (1C, TpB3), 142.3 (1C, TpA3), 141.9 (1C, TpC3), 138.1 (1C, TpC5), 137.6 (1C, TpA5), 137.3 (1C, TpB5), 107.7 (1C, TpB4), 107.2 (1C, TpA4), 106.7 (1C, TpC4), 61.4 (d, *J* = 10.9 Hz, 1C, C1), 52.3 (1C, C2), 51.9 (1C, C9), 47.8 (1C, C6), 46.5 (1C, C7), 40.2 (1C, C3), 36.6 (1C, C5), 34.8 (1C, C4), 34.2 (1C, C8), 23.8 (1C, C9), 13.5 (d, *J* = 28.2 Hz, 3C, PMe3).

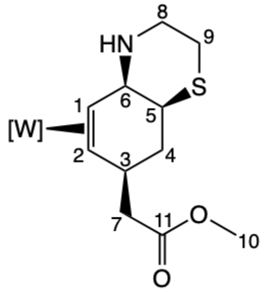

Compound 36:

Compound **2** (125 mg, 0.170 mmol) was placed in a test tube, with ACN, and chilled to -30 °C. After 10 min, a 1 M HOTf/ACN (0.341 mL, 0.341 mmol) solution was added to the test tube and the solution was allowed to stir at -30 °C for 30 min. In a separate test tube, cysteamine (132 mg, 1.70 mmol) in MeOH was cooled at -30 °C for 30 min. After the time elapsed, the former solution was added to the latter, dropwise. The reaction stirred at -30 °C for 2 days and at room temperature for 1 day. The reaction was washed three times (H_2O:Na_2CO_3/DCM; 30 mL/30mL) and dried with $MgSO_4$. The solution was evaporated in vacuo. The resulting film was dissolved in minimal DCM and pipetted in 15 mL of stirring hexane. An off-white solid precipitated out and was collected on a 15 mL fine-porosity fritted disk, washed with hexane (2 × 10 mL) and desiccated overnight to yield compound **36** (52.0 mg, 0.071mmol, 42%).

^{1}H NMR (600 MHz, CD_3CN) δ: 8.24 (d, *J* = 2.1 Hz, 1H, TpA3), 8.03 (d, *J* = 2.0 Hz, 1H, TpB3), 7.86 (d, *J* = 2.4 Hz, 1H, TpB5), 7.83 (d, *J* = 2.3 Hz, 1H, TpC5), 7.79 (d, *J* = 2.4 Hz, 1H, TpA5), 7.39 (d, *J* = 2.2 Hz, 1H, TpC3), 6.38 (t, *J* = 2.2 Hz, 1H, TpB4), 6.27 (t, *J* = 2.3 Hz, 1H, TpA4), 6.25 (t, *J* = 2.3 Hz, 1H, TpC4), 4.02 (s, 1H, H6), 3.74 (s, 1H, H3), 3.64 (s, 3H, H10), 3.42 (m, 1H, H8A), 3.11 (t, *J* = 12.5 Hz, 1H, H8B), 2.98 (d, *J* = 13.4 Hz, 1H, H5), 2.84 (m, 1H, H9A), 2.33 (m, 1H, H7A), 2.25 (t, *J* = 11.1 Hz, 1H, H1), 2.15 (dd, *J* = 14.6, 11.2 Hz, 2H, H7B/H9B), 2.05 (m, 1H, H4A), 1.71 (m, 1H, H4B), 1.15 (d, *J* = 8.3 Hz, 9H, PMe3), 0.75 (dt, *J* = 11.4, 2.6 Hz, 1H, H2). **^{13}C NMR (201 MHz, CD_3CN) δ**: 174.3 (1C, C11), 144.3 (1C, TpB3), 142.2 (1C, TpA3), 141.8 (1C, TpC3), 138.1 (1C, TpA5), 137.6 (1C, TpC5), 137.3 (1C, TpB5), 107.7 (1C, TpB4), 107.2 (1C, TpA4), 106.7 (1C, TpC4), 66.3 (1C, C1), 62.6 (1C, C6), 52.8 (1C, C2), 51.9 (1C, C10), 49.4 (1C, C8), 46.3 (1C, C7), 39.5 (1C, C3), 35.5 (1C, C5), 34.1 (1C, C4), 22.5 (1C, C9), 13.3 (d, *J* = 28.2 Hz, 3C, PMe3).

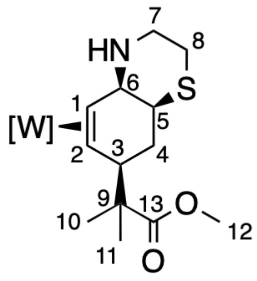

Compound 37:

Compound **3** (125 mg, 0.164 mmol) was placed in a test tube with ACN, and chilled to -30 °C. After 10 min, a 1 M HOTf/ACN (0.328 mL, 0.328 mmol) solution was added to the test tube and the solution was allowed to stir at -30 °C for 30 min. In a separate test tube, Cysteamine (0.127 ml, 1.64 mmol) was cooled at -30 °C for 30 min in MeOH. After the time elapsed, the former solution was added to the latter, dropwise. The reaction stirred at -30 °C for 5 hrs and room temperature for 24 hrs. The reaction was washed three times (H_2O:Na_2CO_3/DCM; 30mL/30mL) and dried with Na_2SO_4. The organic layer was evaporated in vacuo. The resulting yellow film was dissolved in minimal DCM and pipetted in 25 mL of stirring hexane. An off-white solid precipitated

out and was collected on a 15 mL fine-porosity fitted disk, washed with hexane (2 × 10 mL) and desiccated overnight to yield compound **37** (100 mg, 0.132 mmol, 80%).

^{1}H NMR (800 MHz, CD_3CN) δ: 8.01 (d, *J* = 2.1 Hz, 1H, TpA3), 7.97 (d, *J* = 2.0 Hz, 1H, TpB3), 7.83 (d, *J* = 2.4 Hz, 1H, TpB5), 7.80 (d, *J* = 2.3 Hz, 1H, TpC5), 7.79 (d, *J* = 2.4 Hz, 1H, TpA5), 7.36 (d, *J* = 2.2 Hz, 1H, TpC3), 6.32 (t, *J* = 2.2 Hz, 1H, TpB4), 6.28 (t, *J* = 2.3 Hz, 1H, TpA4), 6.26 (t, *J* = 2.3 Hz, 1H, TpC4), 3.88 (d, 1H, H6), 3.48 (s, 3H, H12), 3.40 (m, 1H, H3), 3.35 (d, 1H, H7), 3.04 (m, 1H, H5), 3.00 (m, 1H, H7), 2.81 (d, 1H, H8), 2.55 (m, 1H, H1), 2.33 (m, 1H, H4), 2.04 (m, 1H, H8), 1.43 (m, 1H, H4), 1.10 (d, *J* = 8.1 Hz, 9H, PMe3), 0.91 (s, 3H, H2) 0.72 (m, 1H, H2), 0.71 (s, 3H, H11).**^{13}C NMR (201 MHz, CD_3CN) δ**: 179.2 (1C, C13), 144.0 (1C, TpB3), 143.6 (1C, TpA3), 141.5 (1C, TpC3), 138.1 (1C, TpC5), 137.5 (1C, TpA5), 137.2 (1C, TpB5), 107.3 (1C, TpB4), 107.3 (1C, TpA4), 106.8 (1C, TpC4), 61.8 (1C, C2), 61.5 (1C, C6), 51.9 (1C, C12), 51.2 (1C, C1), 49.8 (1C, C7), 46.0 (1C, C3), 34.4 (1C, C5), 28.4 (1C, C4), 24.0 (1C, C8), 23.1 (1C, C11), 20.9 (1C, C10), 13.40 (d, *J* = 28.1 Hz, 3C, PMe3).

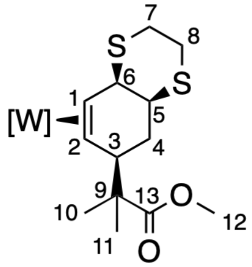

Compound 38:

Compound **3** (100 mg, 0.144 mmol) was placed in a test tube with ACN, and chilled to -30 °C. After 10 min, a 1 M HOTf/ACN (0.289 mL, 0.289 mmol) solution was added to the test tube and the solution was allowed to stir at -30 °C for 30 min. In a separate test tube, Ethane-1,2-dithiol (0.122 ml, 1.45 mmol) and Triethylamine (0.806 ml, 0.578 mmol) were cooled at -30 °C for 30 min in MeOH. After the time elapsed, the former solution was added to the latter, dropwise. The reaction stirred at -30 °C for 5 hrs and room temperature for 24 hrs. The reaction was washed three times (H_2O:Na_2CO_3/DCM; 30mL/30mL) and dried with Na_2SO_4. The organic layer was evaporated in vacuo. The resulting yellow film was dissolved in minimal DCM and pipetted in 25 mL of stirring hexane. An off-white solid precipitated out and was collected on a 15 mL fine-porosity fitted disk, washed with hexane (2 × 10 mL) and desiccated overnight to yield compound **38** (77.0 mg, 0.990 mmol, 69%).

^{1}H NMR (800 MHz, CD_3CN) δ: 8.02 (d, *J* = 2.1 Hz, 1H, TpA3), 7.97 (d, *J* = 2.1 Hz, 1H, TpB3), 7.84 (d, *J* = 2.3 Hz, 1H, TpC5), 7.80 (d, *J* = 2.4 Hz, 1H, TpB5), 7.80 (d, *J* = 2.5 Hz, 1H, TpA5), 7.35 (d, *J* = 2.3 Hz, 1H, TpC3), 6.32 (t, *J* = 2.2 Hz, 1H, TpB4), 6.28 (dt, *J* = 4.4, 2.1 Hz, 2H, TpA4/TpC4), 4.39 (d, *J* = 3.0 Hz, 1H, H6), 3.55 (m, 1H, H3), 3.47 (s, 3H, H12), 3.40 (d, *J* = 13.1 Hz, 1H, H5), 3.13 (ddd, *J* = 13.4, 12.3, 2.4 Hz, 1H, H7A), 3.00 (ddd, *J* = 14.3, 12.2, 2.4 Hz, 1H, H8A), 2.79 (m, 1H, H7B), 2.70 (td, *J* = 12.8, 11.0 Hz, 1H, H4A), 2.51 (dd, *J* = 14.0, 11.6 Hz, 1H, H1), 2.41 (ddd, *J* = 13.7, 4.1, 2.4 Hz, 1H, H8B), 1.48 (ddd, *J* = 12.3, 7.7, 3.4 Hz, 1H, H4B), 1.22

(d, J = 8.3 Hz, 9H, PMe3), 0.97 (s, 3H, H10), 0.74 (dt, J = 11.7, 1.7 Hz, 1H, H2), 0.67 (s, 3H, H11).**^{13}C NMR (201 MHz, CD_3CN) δ:** 178.9 (1C, C13), 143.9 (1C, TpB3), 143.5 (1C, TpA3), 141.5 (1C, TpC3), 138.1 (1C, TpB5), 137.7 (1C, TpA5), 137.2 (1C, TpC5), 107.3 (2C, TpA4/TpB4), 106.7 (1C, TpC4), 59.7 (1C, C1), 51.9 (1C, C12), 50.1 (1C, C2), 49.8 (1C, C9), 49.7 (1C, C6), 45.9 (1C, C3), 35.0 (1C, C5), 34.0 (1C, C7), 28.7 (1C, C4), 23.8 (1C, C11), 23.7 (1C, C8), 20.6 (1C, C10), 13.6 (3C, PMe3).

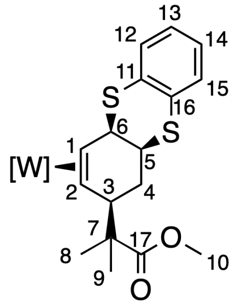

Compound 39:

Compound **3** (100 mg, 0.131 mmol) was placed in a test tube with ACN, and chilled to -30 °C. After 15 min, a 1 M HOTf/ACN (0.262 mL, 0.262mmol) solution was added to the test tube and the solution was allowed to stir at -30 °C for 30 min. In a separate test tube, 1,2-benzenedithiol (187 mg, 1.31 mmol) and triethylamine (0.073 mL, 0.525 mmol) were cooled at -30 °C for 30 min in MeOH. After the time elapsed, the former solution was added to the latter, dropwise. The reaction stirred at -30 °C for 5 hrs and for 24 hrs at RT. The reaction was washed three times (H_2O:NaOH/DCM; 30 mL/30mL) and dried with Na_2SO_4. The organic layer was evaporated in vacuo. The resulting brown film was dissolved in minimal DCM and pipetted in 50 mL of stirring pentane. A white solid precipitated out and was collected on a 15 mL fine-porosity fitted disk, washed with hexane (2 × 10 mL) and desiccated overnight to yield compound **39** (85.0 mg, 0.099 mmol, 79.0%).

^{1}H NMR (600 MHz, CD_3CN) δ: 8.04 (d, J = 2.1 Hz, 1H, TpA3), 7.97 (d, J = 2.0 Hz, 1H, TpB3), 7.86 (d, J = 2.4 Hz, 1H, TpB5), 7.82 (d, J = 2.3 Hz, 1H, TpC5), 7.81 (d, J = 2.4 Hz, 1H, TpA5), 7.43 (d, J = 2.2 Hz, 1H, TpC3), 7.21 (m, 1H, H11), 7.18 (m, 1H, H15), 7.01 (m, 1H, H12/H13), 6.32 (t, J = 2.2 Hz, 1H, TpB4), 6.31 (t, J = 2.3 Hz, 1H, TpA4), 6.28 (t, J = 2.3 Hz, 1H, TpC4), 4.41 (s, 1H, H6), 4.21 (s, 1H, H5), 3.54 (s, 3H, H3), 3.45 (s, 3H, H10), 2.85 (m, 1H, H1), 2.10 (m, 1H, H4), 1.55 (m, 1H, H4), 1.22 (d, J = 8.1 Hz, 9H, PMe3), 0.89 (s, 3H, H8), 0.85 (d, 1H, H2), 0.69 (s, 3H, H9).**^{13}C NMR (201 MHz, CD_3CN) δ**: 178.7 (1C, C17), 144.0 (1C, TpB3), 143.6 (1C, TpA3), 141.6 (1C, TpC3), 138.3 (1C, TpA5), 137.8 (1C, TpC5), 137.3 (1C, TpB5), 136.4 (1C, C16), 131.8 (1C, C15), 128.9 (1C, C14), 128.6 (1C, C11), 126.2 (1C, C13), 125.6 (1C, C12), 107.4 (1C, TpB4), 107.3 (1C, TpA4), 106.8 (1C, TpC4), 57.2 (1C, C2), 52.2 (1C, C6), 52.0 (1C, C10), 50.1 (1C, C7), 49.6 (1C, C1), 45.6 (1C, C3), 39.8 (1C, C5), 31.7 (1C, C4), 23.7 (1C, C9), 20.9 (1C, C8),13.4 (d, J = 27.3 Hz, 3C, PMe3).

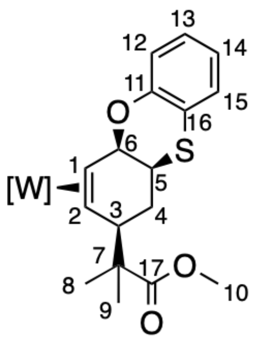

Compound 40:

Compound **3** (70.0 mg, 0.092 mmol) was placed in a test tube with ACN, and chilled to -30 °C. After 10 min, a 1 M HOTf/ACN (0.180 mL, 0.180 mmol) solution was added to the test tube and the solution was allowed to stir at -30 °C for 30 min. In a separate test tube, 2-mercaptophenol (0.10 mL, 0.92 mmol) and triethylamine (0.051 mL, 0.37 mmol) were cooled at -30 °C for 30 min in MeOH. After the time elapsed, the former solution was added to the latter, dropwise. The reaction stirred at -30 °C for 5 hrs and for 24 hrs at RT. The reaction was washed three times (H_2O:NaOH/DCM; 30 mL/30mL) and dried with Na_2SO_4. The organic layer was evaporated in vacuo. The resulting brown film was dissolved in minimal DCM and pipetted in 50 mL of stirring pentane. A white solid precipitated out and was collected on a 15 mL fine-porosity fitted disk, washed with hexane (2 × 10 mL) and desiccated overnight to yield compound **40** (60.0 mg, 0.074 mmol, 81.0%).

^{1}H NMR (600 MHz, $(CD_3)_2CO$) δ: 8.10 (d, J = 2.1 Hz, 1H, TpA3), 8.08 (d, J = 2.0 Hz, 1H, TpB3), 7.99 (d, J = 2.4 Hz, 1H, TpB5), 7.93 (d, J = 2.3 Hz, 1H, TpC5), 7.89 (d, J = 2.4 Hz, 1H, TpA5), 7.65 (d, J = 2.2 Hz, 1H, TpC3), 7.04 (m, 1H, H15), 6.99 (m, 1H, H13), 6.90 (m, 1H, H12), 6.82 (m, 1H, H14), 6.40 (t, J = 2.2 Hz, 1H, TpB4), 6.38 (t, J = 2.3 Hz, 1H, TpA4), 6.32 (t, J = 2.3 Hz, 1H, TpC4), 4.86 (s, 1H, H6), 4.03 (s, 1H, H5), 3.56 (s, 3H, H3), 3.49 (s, 3H, H10), 3.09 (m, 1H, H1), 1.92 (m, 1H, H4), 1.55 (m, 1H, H4), 1.24(d, J = 8.3 Hz, 9H, PMe3), 0.95 (d, 1H, H2), 0.94 (s, 3H, H8), 0.68 (s, 3H, H9). **^{13}C NMR (201 MHz, $(CD_3)_2CO$) δ:** 178.4 (1C, C11), 153.7 (1C, C11), 144.1 (1C, TpB3), 143.3 (1C, TpA3), 141.9 (1C, TpC3), 137.9 (1C, TpA5), 137.6 (1C, TpC5), 137.0 (1C, TpB5), 127.7 (1C, C15), 125.7 (1C, C13), 121.9 (1C, C14), 119.1 (1C, C16), 118.7 (1C, C12), 107.3 (1C, TpB4), 107.1 (1C, TpA4), 106.5 (1C, TpC4), 79.8 (1C, C6), 57.8 (1C, C1), 51.6 (1C, C10), 49.6 (1C, C2), 49.5 (1C, C7), 45.3 (1C, C3), 36.3 (1C, C5), 30.6 (1C, C4), 24.0 (1C, C9), 20.2 (1C, C8), 13.28 (d, J = 28.9 Hz, 3C, PMe3).

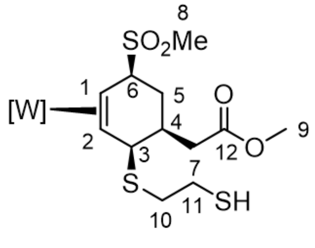

Compound 42:

Compound **2** (75.0 mg, 0.10 mmol) was placed in a test tube with ACN, and chilled to -30 °C. After 10 min, a 1 M HOTf/ACN (0.200 mL, 0.200 mmol) solution was added to the test tube and the solution was allowed to stir at -30 °C for 30 min. In a separate test tube, ethane-1,2-dithiol (0.0690 mL, 0.820 mmol) and triethylamine (0.057 mL, 0.41 mmol) were cooled at -30 °C for 30 min in THF. After the time elapsed, the former solution was added to the latter, dropwise. The reaction stirred at -30 °C for 12 hrs. A pipette-full of 20% KO^{t}Bu/THF solution was added to quench the reaction. A distinct color change was observed from yellow to light brown. The reaction was washed three times (H_2O:NaOH/DCM; 30 mL/30mL) and dried with Na_2SO_4. The organic layer was evaporated in vacuo. The resulting brown film was dissolved in minimal DCM and pipetted in 50 mL of stirring pentane. A white solid precipitated out and was collected on a 15 mL fine-porosity fitted disk, washed with hexane (2 × 10 mL) and desiccated overnight to yield compound **42** (53.0 mg, 0.064 mmol, 63%).

^{1}H NMR (800 MHz, CD_3CN) δ: 8.11 (m, 1H), 8.05 (m, 1H), 7.88 (m, 1H), 7.86 (d, *J* = 2.4 Hz, 1H), 7.79 (d, *J* = 2.5 Hz, 1H), 7.37 (m, 1H), 6.36 (dd, *J* = 2.4, 1.9 Hz, 1H), 6.31 (t, *J* = 2.3 Hz, 1H), 6.29 (t, *J* = 2.3 Hz, 1H), 4.37 (t, *J* = 9.3 Hz, 1H, H6), 3.92 (s, 1H, H3), 3.69 (s, 3H, H9), 3.00 (m, 3H, H8), 2.95 (m, 1H, H1), 2.77 (m, 1H, H7A), 2.50 (m, 2H, H10A/H10B), 2.45 (m, 1H, H7B), 2.25 (m, 2H, H11A/H11B), 1.74 (m, 1H, H5A), 1.68 (m, 1H, H5B), 1.39 (m, 1H, H2), 1.21 (d, *J* = 8.8 Hz, 9H, PMe3).

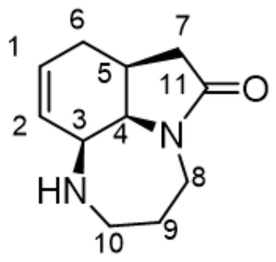

Compound 43:

Compound **19** (70 mg; 0.101 mmol) was placed in a test tube with ACN (2 ml). The solution was cooled to -30°C for 10 minutes. A solution of Br_2 in DCE was added (0.121 mL; 0.121 mmol, 1M). The reaction was stirred at -30°C for 10 minutes and then at room temperature for 10 min. The solution was extracted with an aqueous $Na_2S_2O_3$ (10% w/w) and DCM (3x 30ml). To the aqueous layer from this first extraction was added 30ml saturated sodium hydroxide solution. Another extraction with DCM was carried out (3x40ml DCM). The layers from the second extraction were dried over anhydrous Na_2SO_4 and were evaporated in vacuo to yield a yellow oil 10 mg, 0.052 mmol, 52%).

^{1}H NMR (800 MHz, $(CD_3)_2CO$) δ: 5.90 (1H, ddt, *J* = 9.8, 4.3, 1.8 Hz, H2), 5.83 (1H, dddd, *J* = 9.9, 5.3, 3.9, 1.7 Hz, H1), 3.95 (1H, dtd, *J* = 13.7, 3.8, 1.5 Hz, H9), 3.80 (1H, dd, *J* = 8.1, 6.8 Hz, H4), 3.36 (1H, m, H3), 3.15 (1H, dddd, *J* = 13.6, 5.0, 3.6, 1.5 Hz, H11), 2.80 (1H, m, H9), 2.62 (2H, m, H5/H11), 2.23 (1H, dddd, *J* = 17.0, 8.8, 4.0, 2.0 Hz, H6), 2.18 (1H, dd, *J* = 16.1, 8.8 Hz, H7), 2.12 (1H, m, H6), 2.01 (1H, dd, *J* = 16.1, 9.2 Hz, H7), 1.79 (1H, m, H10), 1.45 (1H, dp, *J* = 13.5, 3.3

Hz, H10). **^{13}C NMR (201 MHz, $(CD_3)_2CO$) δ:** 174.9 (1C, C8), 131.0 (1C, C2), 128.2 (1C, C1), 63.3 (1C, C4), 58.1 (1C, C3), 50.8 (1C, C11), 43.4 (1C, C9), 37.7 (1C, C7), 30.9 (1C, C5), 29.4 (1C, C10), 27.5 (1C, C6). **HRMS (ESI$^+$, deconvoluted):** [M] calcd. for $C_{11}H_{16}N_2O$, 192.1263; found, 192.1263

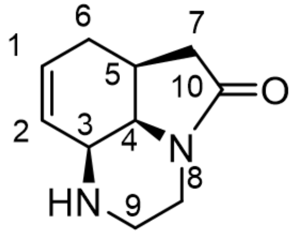

Compound 44:

Compound **5** (70 mg, 0.103 mmol) was placed in a test tube with ACN (2 ml). The solution was cooled to -30°C for 10 minutes. A solution of Br_2 in DCE was added (123 mL, 0.123 mmol, 1M). The reaction was stirred at -30°C for 10 minutes and then at room temperature for 10 min. The solution was extracted with an aqueous $Na_2S_2O_3$ (10% w/w) and DCM (3x 30ml). To the aqueous layer from this first extraction was added 30ml saturated sodium hydroxide solution. Another extraction with DCM was carried out (3x40ml DCM). The layers from the second extraction were dried over anhydrous Na_2SO_4 and were evaporated in vacuo to yield a yellow oil (11 mg, 0.062 mmol, 60%).

^{1}H-NMR (800 MHz, CD_3CN) δ: 5.95 (1H, m, H1), 5.70 (1H, m, H2), 3.92 (1H, m, H9a), 3.79 (1H, td, H4), 3.48 (1H, m, H3), 2.80 (1H, m, H8a), 2.75 (2H, m, H9b, H8a), 2.68 (1H, m, H5), 2.57 (1H, m, H7a), 2.12 (1H, m, H6a), 2.02 (1H, dd, H7b), 1.94 (1H, dt, H6b). **^{13}C-NMR (201 MHz, CD_3CN) δ:** 174.1 (1C, s, C10), 130.4 (1C, s, C2), 129.3 (1C, s, C1), 57.9 (1C, s, C4), 50.0 (1C, s, C3), 41.0 (1C, s, C9), 39.7 (1C, s, C7), 39.4 (1C, s, C8), 28.9 (1C, s, C5), 27.6 (1C, s, C6). **HRMS (APCI$^+$, deconvoluted):** [M] calcd. for $C_{10}H_{14}N_2O$, 178.1106; found, 178.1106.

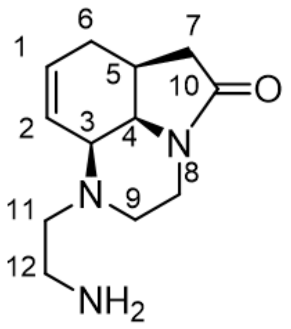

Compound 45:

Compound **16** (120 mg, 0.207 mmol) was placed in a test tube with ACN (2 mL). The solution was cooled to -30°C for 10 minutes. A solution of Br_2 in DCE was added (0.249 mL. 0.249 mmol, 1M). The reaction was stirred at -30°C for 10 minutes and then at room temperature for 10 min. The solution was extracted with an aqueous $Na_2S_2O_3$ (10% w/w) and DCM (3x 30ml). To the aqueous layer from this first extraction was added 30ml saturated sodium hydroxide solution. Another extraction with DCM was carried out (3x40ml DCM). The layers from the second extraction were dried over anhydrous Na_2SO_4 and were evaporated in vacuo to yield a yellow oil (31 mg, 0.140 mmol, 68%).

^{1}H-NMR (800 MHz, $CDCl_3$) δ: 6.01 (1H, m, H1), 5.86 (1H, m, H2), 3.90 (1H, m, H4), 3.88 (1H, m, H8), 3.29 (1H, m, H3), 2.81 (3H, m, H9, H8), 2.73 (3H, m, H12, H5), 2.58 (1H, m, H11, H7), 2.48 (1H, t, H11), 2.10 (1H, m, H6), 1.99 (2H, t, H7, H6). **^{13}C-NMR (800 MHz, $CDCl_3$) δ**: 173.9 (1C, C10), 130.1 (1C, C1), 126.0 (1C, C2), 58.5 (1C, C4), 56.7 (1C, C3), 57.1 (1C, C12), 45.5 (1C, C11), 39.8 (1C, C8), 39.5 (1C, C7), 39.4 (1C, C9), 30.1 (1C, C5), 28.5 (1C, C6). **HRMS (APCI$^+$, deconvoluted):** [M] calcd. for $C_{12}H_{19}N_3O$, 221.1528; found, 221.1563.

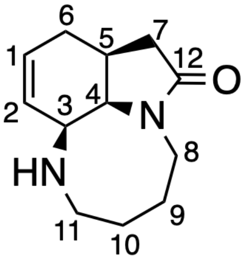

Compound 46:

Compound **22** (105 mg, 0.148 mmol) was placed in a test tube with ACN (5 mL). A solution of Br_2 in DCE was added (0.185 mL, 0.185 mmol, 1M). The reaction was stirred at room temperature for 10 min. The solution was extracted with an aqueous $Na_2S_2O_3$ (10% w/w) and DCM (3x 30ml). To the aqueous layer from this first extraction a 10% NaOH solution was added (30ml). Another extraction with DCM was carried out (3x40ml DCM). The layers from the second extraction were dried over anhydrous Na_2SO_4 and were evaporated in vacuo to yield a yellow oil (19 mg, 0.92 mmol, 62%).

^{1}H NMR (800 MHz, CD_3CN) δ: 5.90 (ddt, *J* = 9.8, 5.6, 2.1 Hz, 1H, H1), 5.72 (dtd, *J* = 9.6, 3.9, 1.4 Hz, 1H, H2), 3.96 (ddd, *J* = 13.8, 10.0, 4.9 Hz, 1H, H8A), 3.52 (dd, *J* = 7.6, 5.7 Hz, 1H, H4), 3.42 (q, *J* = 7.0 Hz, 1H, H11A), 3.16 (t, *J* = 5.8 Hz, 1H, H3), 2.86 (ddd, *J* = 12.9, 6.4, 3.8 Hz, 1H, H6A), 2.81 (dt, *J* = 13.8, 4.8 Hz, 1H, H8B), 2.58 (dddd, *J* = 13.6, 7.5, 5.6, 2.7 Hz, 2H, H5/H11B), 2.25 (m, 1H, H7A), 2.10 (m, 2H, H6B/H7B), 1.85 (ttd, *J* = 9.9, 5.0, 1.2 Hz, 1H, H9A), 1.57 (m, 2H, H9B/H10A), 1.50 (m, 1H, H10B). **^{13}C NMR (201 MHz, CD_3CN) δ** 176.7 (1C, C12), 128.9 (1C, C2), 126.7 (1C, C1), 61.4 (1C, C4), 55.7 (1C, C3), 51.3 (1C, C11), 43.4 (1C, C8), 38.9 (1C, C7), 31.2 (1C, C5), 29.3 (1C, C10), 27.8 (1C, C9), 27.2 (1C, C6). **HRMS (APCI$^+$, deconvoluted):** [M] calcd. for $C_{12}H_{18}N_2O$, 206.1419; found, 206.1422.

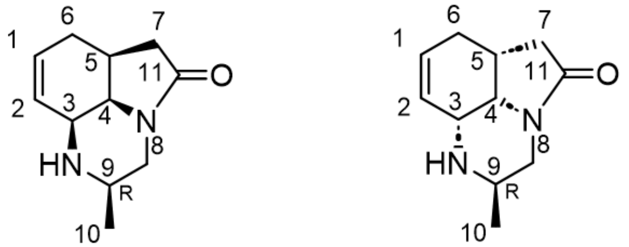

Compound 47A+47B:

Compounds **10A + 10B** (60 mg, 0.110 mmol) were placed in a test tube with ACN (2 mL). A solution of Br_2 in DCE was added (0.100 mL, 0.1300 mmol, 1M). The reaction was stirred at room temperature for 10 min. The solution was extracted with an aqueous $Na_2S_2O_3$ (10% w/w) and DCM (3x 30ml). To the aqueous layer from this first extraction a 10% NaOH solution was added (30ml). Another extraction with DCM was carried out (3x40ml DCM). The layers from the second extraction were dried over anhydrous Na_2SO_4 and were evaporated in vacuo to yield a yellow oil (10 mg, 0.052 mmol, 60%).

47A: ^{1}H-NMR (800 MHz, CD_3CN) δ: 5.83 (2H, s, H1/H2), 3.89 (1H, t, H4), 3.61 (1H, d, H3), 3.51 (1H, dd, H8), 3.20 (1H, m, H9), 3.07 (1H, dd, H8), 2.67 (1H, m, H5), 2.44 (1H, m, H7), 2.08 (1H, m, H6), 1.97 (2H, m, H6/H7), 1.06 (3H, d, H10). **^{13}C-NMR (201 MHz, CD_3CN) δ:** 175.2 (1C, C11), 129.8 (1C, C1), 128.4 (1C, C2), 55.6 (1C, C4), 51.0 (1C, C3), 47.5 (1C, C9), 46.9 (1C, C8), 38.9 (1C, C7), 29.8 (1C, C5), 26.9 (1C, C6), 19.7 (1C, C10).

47B: ^{1}H-NMR (800 MHz, CD_3CN) δ: 5.94 (1H, m, H2), 5.73 (1H, d, H1), 3.76 (2H, m, H8/H4), 3.45 (1H, bs, H3), 2.79 (1H, m, H5/H9), 2.50 (1H, m, H7), 2.32 (1H, t, H8), 2.08 (1H, m, H6), 1.86 (1H, m, H6/H7), 1.00 (3H, d, H10). **^{13}C-NMR (201 MHz, CD_3CN) δ:** 173.8 (1C, C11), 129.8 (1C, C1), 128.4 (1C, C2), 57.4 (1C, C4), 51.5 (1C, C3), 47.5 (1C, C9), 45.8 (1C, C8), 40.2 (1C, C7), 29.0 (1C, C5), 28.2 (1C, C6), 18.9 (1C, C10).

47A+47B: HRMS ($APCI^+$, deconvoluted): [M] calcd. for $C_{11}H_{16}N_2O$, 192.1263; found, 192.1259.

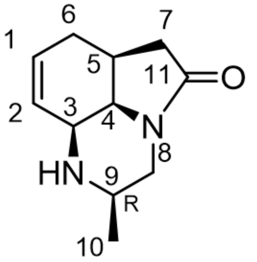

Compound 48:

Compound **11** (75 mg, 0.110 mmol) was placed in a test tube with ACN (2 mL). A solution of Br_2 in DCE was added (0.130 mL, 0.130 mmol, 1M). The reaction was stirred at room temperature for 10 min. The solution was extracted with an aqueous $Na_2S_2O_3$ (10% w/w) and DCM (3x 30ml). To the aqueous layer from this first extraction a 10% NaOH solution was added (30ml). Another extraction with DCM was carried out (3x40ml DCM). The layers from the second extraction were

dried over anhydrous Na_2SO_4 and were evaporated in vacuo to yield a yellow oil (16 mg, 0.083 mmol, 77%).

^{1}H-NMR (800 MHz, CD_3CN) δ: 5.81 (2H, s, H1/H2), 3.82 (1H, t, H4), 3.51 (1H, d, H3), 3.39 (1H, dd, H8), 3.09 (1H, m, H9), 3.01 (1H, dd, H8), 2.66 (1H, m, H5), 2.44 (1H, m, H7), 2.08 (1H, m, H6), 1.97 (2H, m, H6/H7), 1.08 (3H, d, H10). **^{13}C-NMR (201 MHz, CD_3CN) δ:** 175.2 (1C, C11), 132.9 (1C, C1), 127.4 (1C, C2), 56.2 (1C, C4), 51.0 (1C, C3), 47.2 (1C, C9), 46.5 (1C, C8), 38.9 (1C, C7), 30.0 (1C, C5), 26.9 (1C, C6), 20.3 (1C, C10). **HRMS (APCI$^+$, deconvoluted):** [M] calcd. for $C_{11}H_{16}N_2O$, 192.1263; found, 192.1262.

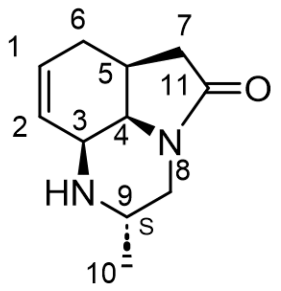

Compound 49:
Compound **12** (75 mg, 0.110 mmol) was placed in a test tube with ACN (2 mL). A solution of Br_2 in DCE was added (0.130 mL, 0.130 mmol, 1M). The reaction was stirred at room temperature for 10 min. The solution was extracted with an aqueous $Na_2S_2O_3$ (10% w/w) and DCM (3x 30ml). To the aqueous layer from this first extraction a 10% NaOH solution was added (30ml). Another extraction with DCM was carried out (3x40ml DCM). The layers from the second extraction were dried over anhydrous Na_2SO_4 and were evaporated in vacuo to yield a yellow oil (15 mg, 0.078 mmol, 72%).

^{1}H-NMR (800 MHz, CD_3CN) δ: 5.89 (1H, m, H2), 5.71 (1H, d, H1), 3.72 (2H, m, H8/H4), 3.41 (1H, bs, H3), 2.67 (1H, m, H5/H9), 2.49 (1H, m, H7), 2.22 (1H, t, H8), 2.08 (1H, m, H6), 1.87 (1H, m, H6), 1.85 (1H, m, H7), 1.00 (3H, d, H10). **^{13}C-NMR (201 MHz, CD_3CN) δ:** 173.8 (1C, C11), 132.5 (1C, C1), 129.2 (1C, C2), 57.9 (1C, C4), 51.4 (1C, C3), 47.6 (1C, C9), 45.2 (1C, C8), 40.3 (1C, C7), 29.1 (1C, C5), 28.2 (1C, C6), 19.4 (1C, C10). **HRMS (APCI$^+$, deconvoluted):** [M] calcd. for $C_{11}H_{16}N_2O$, 192.1263; found, 192.1262.

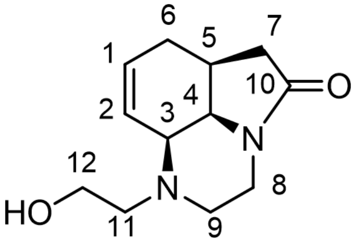

Compound 50:

Compound **14** (150 mg, 0.207 mmol) was placed in a test tube with ACN (5 mL). A solution of Br_2 in DCE was added (0.248 mL. 0.248 mmol, 1M). The reaction was stirred at room temperature for 10 min. The solution was extracted with an aqueous $Na_2S_2O_3$ (10% w/w) and DCM (3x 30ml). To the aqueous layer from this first extraction a 10% NaOH solution was added (30ml). Another extraction with DCM was carried out (3x40ml DCM). The layers from the second extraction were dried over anhydrous Na_2SO_4 and were evaporated in vacuo to yield a yellow oil (33 mg, 0.150 mmol, 73%).

^{1}H NMR (800 MHz, CD_3CN) δ: 6.00 (1H, ddd, H1), 5.91 (1H, m, H2), 3.87 (1H, m, H4), 3.74 (1H, dd, H8), 3.52 (2H, m, H12), 3.32 (1H, bs, H3), 2.79 (1H, m, H8), 2.75 (2H, m, H11), 2.72 (1H, m, H5), 2.65 (1H, m, H9), 2.51 (1H, m, H5), 2.48 (1H, m, H9), 2.11 (1H, m, H6), 1.98 (1H, m, H7), 1.84 (1H, dd, H7).**^{13}C NMR (201 MHz, CD_3CN) δ:** 173.8 (1C, C10), 130.6 (1C, C1), 126.9 (1C, C2), 59.3 (1C, C12), 58.5 (1C, C4), 57.1 (1C, C11), 57.0 (1C, C3), 45.9 (1C, C9), 39.8 (1C, C7), 39.4 (1C, C8), 28.8 (1C, C5), 28.5 (1C, C6). **HRMS (APCI$^+$, deconvoluted):** [M] calcd. for $C_{12}H_{18}N_2O_2$, 222.1368; found 222.1370.

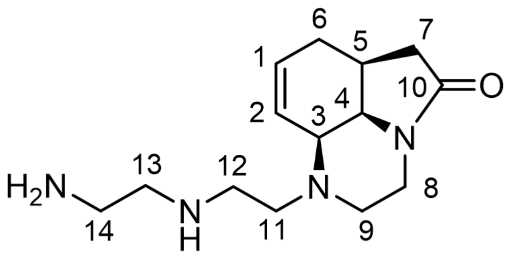

Compound 51:

Compound **17** (80 mg, 0.10 mmol) was placed in a test tube with ACN (2 mL). The solution was cooled to -30°C for 10 minutes. A solution of Br_2 in DCE was added (0.13 mL. 0.13 mmol, 1 molar). The reaction was stirred at room temperature for 10 min. The solution was extracted with an aqueous $Na_2S_2O_3$ (10% w/w) and DCM (3x 30ml). To the aqueous layer from this first extraction a 10% NaOH solution was added (30ml). Another extraction with DCM was carried out (3x40ml DCM). The layers from the second extraction were dried over anhydrous Na_2SO_4 and were evaporated in vacuo to yield a yellow oil (16 mg, 0.061 mmol, 58%).

^{1}H NMR (800 MHz, CD_3CN) δ: 6.01 (1H, m, H1), 5.90 (1H, d, H2), 3.84 (1H, m, H4), 3.70 (1H, d, H8), 3.42 (2H, m, H14), 3.31 (1H, bs, H3), 2.74 (1H, m, H8), 2.74 (4H, m, H13/H11), 2.70 (1H, m, H5), 2.68 (1H, m, H9), 2.60 (2H, m, H12), 2.52 (1H, m, H7), 2.41 (1H, m, H9), 2.11 (1H, m, H6), 1.95 (1H, m, H6), 1.83 (1H, dd, H7).**^{13}C NMR (201 MHz, CD_3CN) δ:** 172.9 (1C, C10), 129.5 (1C, C1), 126.1 (1C, C2), 65.3 (1C, C14), 57.3 (1C, C4), 55.6 (1C, C3), 53.8 (1C, C13), 53.6 (1C, C11), 52.1 (1C, C12), 45.0 (1C, C9), 38.9 (1C, C7), 38.6 (1C, C8), 27.9 (2C, C6/C5). **HRMS (APCI$^+$, deconvoluted):** [M] calcd. for $C_{14}H_{24}N_4O$, 264.1950; found 264.1954.

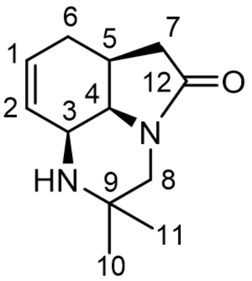

Compound 52:
Compound **8** (100 mg, 0.141 mmol) was placed in a test tube with ACN (2 mL). The solution was cooled to -30°C for 10 minutes. A solution of Br_2 in DCE was added (0.176 mL. 0.176 mmol, 1M). The reaction was stirred at room temperature for 10 min. The solution was extracted with an aqueous $Na_2S_2O_3$ (10% w/w) and DCM (3x 30ml). To the aqueous layer from this first extraction a 10% NaOH solution was added (30ml). Another extraction with DCM was carried out (3x40ml DCM). The layers from the second extraction were dried over anhydrous Na_2SO_4 and were evaporated in vacuo to yield a yellow oil (21 mg, 0.10 mmol, 72%).

^{1}H NMR (800 MHz, CD_3CN) δ: 5.82 (1H, m, H1), 5.72 (1H, m, H2), 3.69 (1H, m, H4), 3.62 (1H, m, H8), 3.49 (1H, m, H3), 3.37 (1H, m, H5), 2.71 (1H, d, H8), 2.49 (1H, m, H7), 2.44 (1H, m, H6), 2.01 (1H, m, H6), 1.98 (1H, m, H7), 1.13 (3H, s, H10), 1.05 (3H, s, H11). **^{13}C NMR (201 MHz, CD_3CN) δ:** 174.5 (1C, C12), 132.3 (1C, C1), 129.7 (1C, C2), 55.2 (1C, C4), 49.4 (1C, C8), 49.3 (1C, C3), 49.0 (1C, C9), 38.8 (1C, C7), 28.6 (1C, C5), 26.7 (1C, C6), 13.3 (1C, C10), 10.4 (1C, C11). **HRMS (APCI$^+$, deconvoluted):** [M] calcd. for $C_{12}H_{18}N_2O$, 206.1419; found, 206.1421

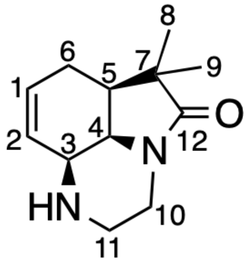

Compound 53:
Compound **6** (98 mg, 0.14 mmol) was placed in a test tube with ACN (5 mL). A solution of Br_2 in DCE was added (0.18 mL. 0.18 mmol, 1M). The reaction was stirred at room temperature for 10 min. The solution was extracted with an aqueous $Na_2S_2O_3$ (10% w/w) and DCM (3x 30ml). To the aqueous layer from this first extraction a 10% NaOH solution was added (30ml). Another extraction with DCM was carried out (3x40ml DCM). The layers from the second extraction were dried over anhydrous Na_2SO_4 and were evaporated in vacuo to yield a yellow oil (19 mg, 0.093 mmol, 67%).

^{1}H-NMR (800 MHz, CD_3CN) δ: 5.93 (1H, m, H1), 5.61 (1H, m, H2), 3.84 (1H, m, H10), 3.65 (1H, td, H4), 3.44 (1H, m, H3), 2.68 (1H, m, H10), 2.60 (2H, m, H11), 2.37 (1H, m, H5), 2.24 (1H, m, H6), 1.92 (1H, m, H6), 1.16 (s, 3H, H8), 1.08 (s, 3H, H9). **^{13}C-NMR (201 MHz, CD_3CN) δ:** 178.9

(1C, C12), 130.9 (1C, C2), 129.8 (1C, C1), 55.0 (1C, C4), 50.8 (1C, C3), 44.7 (1C, C7), 41.7 (1C, C11), 41.6 (1C, C10), 40.15 (1C, C5), 26.0 (1C, C6), 23.7 (1C, C8), 20.5 (1C, C9). **HRMS (APCI$^+$, deconvoluted):** [M] calcd. for $C_{12}H_{18}N_2O$, 206.1419; found, 206.1423

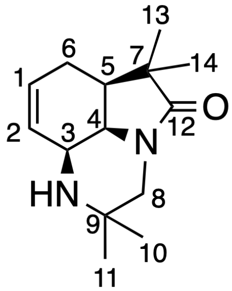

Compound 54:
Compound **9** (70 mg, 0.095 mmol) was placed in a test tube with ACN (2 mL). The solution was cooled to -30°C for 10 minutes. A solution of Br_2 in DCE was added (0.114 mL. 0.114 mmol, 1M). The reaction was stirred at room temperature for 10 min. The solution was extracted with an aqueous $Na_2S_2O_3$ (10% w/w) and DCM (3x 30ml). To the aqueous layer from this first extraction a 10% NaOH solution was added (30ml). Another extraction with DCM was carried out (3x40ml DCM). The layers from the second extraction were dried over anhydrous Na_2SO_4 and were evaporated in vacuo to yield a yellow oil (14 mg, 0.060 mmol, 63%).

^{1}H NMR (800 MHz, $CDCl_3$) δ: 5.82 (2H, m, H1/H2), 3.80 (1H, m, H4), 3.76 (1H, d, H8), 3.74 (1H, m, H3), 2.30 (1H, d, H8), 2.28 (2H, q, H5/H6), 1.99 (1H, m, H6), 1.25 (3H, s, H10), 1.19 (3H, s, H11), 1.15 (3H, s, H13), 1.10 (3H, s, H14).**^{13}C NMR (201 MHz, $CDCl_3$) δ:** 179.3 (1C, C12), 126.9 (2C, C1/C2), 52.8 (1C, C4), 50.1 (1C, C8), 49.2 (1C, C3), 45.1 (1C, C9), 41.4 (1C, C7), 29.4 (1C, C5), 29.0 (1C, C6), 25.7 (1C, C10), 25.2 (1C, C11) 22.7 (1C, C13), 20.2 (1C, C14). **HRMS (APCI$^+$, deconvoluted):** [M] calcd. for $C_{14}H_{22}N_2O$, 234.1732; found, 234.1737.

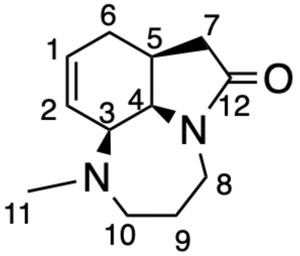

Compound 55:
Compound **20** (112 mg, 0.158 mmol) was placed in a test tube with ACN (2 mL). A solution of Br_2 in DCE was added (0.205 mL, 0.205 mmol, 1M). The reaction was stirred at room temperature for 10 min. The solution was extracted with an aqueous $Na_2S_2O_3$ (10% w/w) and DCM (3x 30ml). To the aqueous layer from this first extraction a 10% NaOH solution was added (30ml). Another extraction with DCM was carried out (3x40ml DCM). The layers from the second extraction were

dried over anhydrous Na_2SO_4 and were evaporated in vacuo to yield a yellow oil (22 mg, 0.11 mmol, 67%).

^{1}H NMR (800 MHz, CD_3CN): δ 5.91 (q, *J* = 2.4 Hz, 2H, H1/H2), 3.99 (dd, *J* = 7.4, 5.1 Hz, 1H, H4), 3.59 (ddd, *J* = 13.6, 6.6, 4.0 Hz, 1H, H8A), 3.34 (s, 1H, H3), 3.08 (ddd, *J* = 13.2, 9.0, 3.7 Hz, 1H, H8B), 2.86 (dt, *J* = 13.6, 5.9 Hz, 1H, H10A), 2.75 (ddd, *J* = 13.4, 7.9, 5.2 Hz, 1H, H10B), 2.65 (m, 1H, H5), 2.42 (s, 3H, H11), 2.39 (m, 1H, H7A), 2.16 (m, 1H, H6A), 1.91 (s, 2H, H6B/H7B), 1.69 (m, 2H, H9). **^{13}C NMR (201 MHz, CD_3CN):** δ 174.9 (1C, C12), 129.0 (1C, C2), 128.0 (1C, C1), 63.7 (1C, C4), 62.5 (1C, C3), 53.5 (1C, C10), 42.8 (1C, C11), 42.2 (1C, C8), 39.6 (1C, C7), 30.4 (1C, C5), 28.6 (1C, C6), 26.5 (1C, C9). **HRMS (APCI$^+$, deconvoluted):** [M] calcd. for $C_{12}H_{18}N_2O$, 206.1419; found, 206.1423.

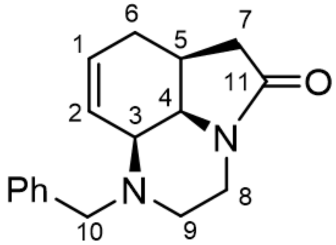

Compound 56:

Compound **13** (70 mg, 0.091 mmol) was placed in a test tube with ACN (2 mL). A solution of Br_2 in DCE was added (0.109 mL, 0.109 mmol, 1M). The reaction was stirred at room temperature for 10 min. The solution was extracted with an aqueous $Na_2S_2O_3$ (10% w/w) and DCM (3x 30ml). To the aqueous layer from this first extraction a 10% NaOH solution was added (30ml). Another extraction with DCM was carried out (3x40ml DCM). The layers from the second extraction were dried over anhydrous Na_2SO_4 and were evaporated in vacuo to yield a yellow oil (15 mg, 0.056 mmol, 62%).

^{1}H NMR (800 MHz, CD_2Cl_2) δ: 7.34-7.23 (5H, Ph), 5.95 (1H, m, H1), 5.68 (1H, dt, H2), 3.80 (4H, m, H3/H4/H8/H10), 2.74 (1H, m, H8), 2.68 (3H, m, H9/H10), 2.49 (2H, m, H5/H7), 2.24 (1H, m, H6), 2.09 (1H, m, H7), 1.16 (1H, m, H6). **^{13}C NMR (201 MHz, CD_2Cl_2) δ**: 173.8 (1C, C11), 130.9 (2C, Ph), 129.3 (2C, Ph), 128.6 (1C, C2), 128.4 (1C, C1), 104.0 (1C, Ph), 41.3 (1C, C4), 39.4 (1C, C3), 39.9 (1C, C9), 39.8 (1C, C8), 32.3 (1C, C7), 30.1 (1C, C5), 29.2 (1C, C6) 27.9 (1C, C10). **HRMS (APCI$^+$, deconvoluted):** [M] calcd. for $C_{17}H_{20}N_2O$, 268.1576; found, 268.1577.

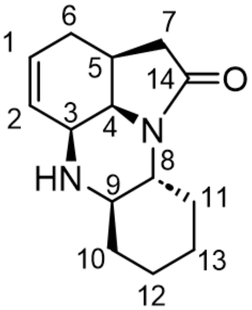

Compound 57:

Compound **15** (63 mg, 0.086 mmol) was placed in a test tube with ACN (2 mL). A solution of Br_2 in DCE was added (0.103 mL, 0.103 mmol, 1M). The reaction was stirred at room temperature for 10 min. The solution was extracted with an aqueous $Na_2S_2O_3$ (10% w/w) and DCM (3x 30ml). To the aqueous layer from this first extraction a 10% NaOH solution was added (30ml). Another extraction with DCM was carried out (3x40ml DCM). The layers from the second extraction were dried over anhydrous Na_2SO_4 and were evaporated in vacuo to yield a yellow oil (12 mg, 0.052 mmol, 60%).

^{1}H-NMR (800 MHz, CD_2Cl_2) δ: 5.87 (1H, m, H1), 5.77 (1H, d, H2), 4.11 (1H, t, H4), 3.75 (1H, d, H3), 3.22 (1H, t, H8), 2.70 (1H, m, H5), 2.61 (1H, t, H9), 2.47 (1H, dd, H7), 2.21 (1H, m, H11), 1.99 (3H, m, H6/H7), 1.95 (1H, m, H10), 1.75 (1H, m, H10), 1.37 (2H, m, H12/H13), 1.25 (3H, H11/H12/H13). **^{13}C-NMR (201 MHz, CD_2Cl_2) δ:** 175.4 (1C, C14), 131.4 (1C, C2), 127.8 (1C, C1), 61.1 (1C, C8), 56.5 (1C, C9), 54.0 (2C, C4/C3), 39.6 (1C, C7), 33.0 (1C, C11), 29.7 (1C, C5), 29.6 (1C, C10), 26.7 (1C, C6), 26.0 (1C, C12), 25.1 (1C, C13). **HRMS (APCI$^+$, deconvoluted):** [M] calcd. for $C_{14}H_{20}N_2O$, 232.1576; found, 232.1578.

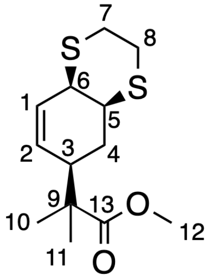

Compound 58:

Compound **38** (77 mg, 0.099 mmol) was placed in a test tube with ACN (2 mL). 2,3-Dichloro-5,6-dicyano-1,4-benzoquinone (45 mg, 0.20 mmol) in ACN (2 mL) was added dropwise, then the reaction allowed to stir at room temperature for 10 minutes The reaction was washed three times (H_2O:$NaHCO_3$/DCM; 30mL/30mL) and dried over anhydrous Na_2SO_4. The organic layer was evaporated in vacuo. The resulting yellow film was dissolved in minimal DCM and pipetted in 100 mL of stirring hexane. A brown solid precipitated out and was collected on a 15 mL fine-porosity fitted disk and washed two times with diethyl ether (5ml). The resulting filtrate was evaporated to dryness to yield a clear oil (20 mg, 0.073 mmol, 74%).

^{1}H NMR (800 MHz, CD_3CN) δ 5.73 (ddd, *J* = 10.0, 5.5, 2.6 Hz, 1H, H1), 5.57 (ddt, *J* = 9.9, 2.4, 1.3 Hz, 1H, H2), 3.82 (ddq, *J* = 6.3, 3.1, 1.6 Hz, 1H, H6), 3.64 (s, 3H, H12), 2.99 (m, 2H, H7A/H8A), 2.87 (dtd, *J* = 12.8, 3.2, 1.4 Hz, 1H, H5), 2.74 (ddt, *J* = 13.1, 4.4, 2.4 Hz, 2H, H3/H7B), 2.61 (td, *J* = 12.6, 11.2 Hz, 1H, H4A), 2.48(m, 1H, H8B), 1.75 (m, 1H, H4B), 1.15 (s, 3H, H10), 1.13 (s, 3H, H11). **^{13}C NMR (201 MHz, CD_3CN) δ:** 178.3 (1C, C13), 131.5 (1C, C2), 129.6 (1C, C1), 52.4 (1C, C12), 46.3 (1C, C3), 45.8 (1C, C9), 42.1 (1C, C6), 37.3 (1C, C5), 32.2 (1C, C7), 27.5 (1C, C4), 24.3 (1C, C8), 22.6 (1C, C10), 22.2 (1C, C11). **HRMS (APCI$^+$, deconvoluted):** [M] calcd. for $C_{13}H_{20}O_2S_2$, 272.0905; found, 272.0895.

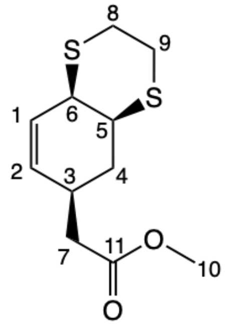

Compound 59:

Compound **35** (60 mg, 0.08 mmol) was placed in a test tube with ACN (2 mL). 2,3-Dichloro-5,6-dicyano-1,4-benzoquinone (36 mg, 0.16 mmol) in ACN (2 mL) was added dropwise, then the reaction allowed to stir at room temperature over 10 minutes. The reaction was washed three times (H_2O:$NaHCO_3$/DCM; 30mL/30mL) and dried over anhydrous Na_2SO_4. The organic layer was evaporated in vacuo. The resulting yellow film was dissolved in minimal DCM and pipetted in 25 mL of stirring hexane. A brown solid precipitated out and was collected on a 15 mL fine-porosity fitted disk and washed two times with diethyl ether (5ml). The resulting filtrate was evaporated to dryness to yield a clear oil (14 mg, 0.057 mmol, 72%).

^{1}H NMR (800 MHz, CD_3CN) δ: 5.65 (ddd, *J* = 9.9, 5.3, 2.5 Hz, 1H, H1), 5.60 (ddt, *J* = 9.8, 2.3, 1.2 Hz, 1H, H2), 3.84 (ddd, *J* = 6.4, 3.1, 1.4 Hz, 1H, H6), 3.63 (s, 3H, H10), 2.97 (m, 1H, H8A), 2.96 (m, 1H, H9A), 2.89 (dtd, *J* = 12.7, 2.8, 1.2 Hz, 1H, H5), 2.78 (m, 1H, H3), 2.74 (m, 1H, H8B), 2.46 (m, 2H, H4A/H9B), 2.32 (dd, *J* = 15.5, 7.1 Hz, 1H, H7A), 2.30 (dd, *J* = 15.5, 7.7 Hz, 1H, H7B), 1.92 (ddd, *J* = 5.6, 2.7, 1.4 Hz, 1H, H4B).**^{13}C NMR (201 MHz, CD_3CN) δ:** 173.3 (1C, C11), 133.7 (1C, C2), 128.5 (1C, C1), 52.1 (1C, C10), 41.9 (1C, C6), 40.6 (1C, C7), 36.9 (1C, C5), 35.9 (1C, C3), 32.2 (1C, C4), 32.1 (1C, C8), 24.3 (1C, C9). **HRMS (APCI$^+$, deconvoluted):** [M] calcd. for $C_{11}H_{16}O_2S_2$, 244.0592; found, 244.0594.

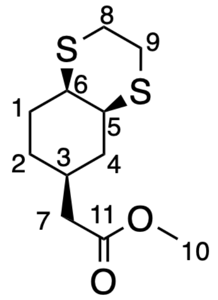

Compound 60:
Compound **59** was dissolved in methanol (2 mL) and added to a 4-dram vial. This solution was then circulated through a ThalesNano H-Cube flow hydrogenator for 4 h. The temperature was set to 50 °C and the H2 pressure to 25 bars. The catalyst cartridge used contained 5%Pd on carbon. After circulation, the solution was collected and reduced to dryness (12 mg, 0.049 mmol, 85%).

^{1}H NMR (800 MHz, CD_3CN) δ: 3.61 (s, 2H, H10), 3.58 (s, 1H, H6), 3.02 (t, 1H, H8), 2.95 (t, 1H, H9), 2.81 (d, 1H, H8), 2.74 (d, 1H, H5), 2.42 (d, 1H, H9), 2.37 (q, 1H, H4), 2.26 (m, 2H, H7), 1.93 (m, 1H, H1), 1.87 (m, 1H, H3), 1.74 (m, 3H, H4/H1), 1.44 (m, 1H, H2), 1.23 (m, 1H, H2).**^{13}C NMR (201 MHz, CD_3CN) δ:** 173.5 (1C, C11), 51.8 (1C, C10), 43.3 (1C, C6), 41.6 (1C, C7), 39.5 (1C, C5), 36.8 (1C, C3), 34.6 (1C, C4), 33.7 (1C, C1), 31.3 (1C, C8), 26.4 (1C, C2), 23.6 (1C, C9). **HRMS (APCI$^+$, deconvoluted):** [M] calcd. for $C_{11}H_{18}O_2S_2$, 246.0748; found, 246.0749.

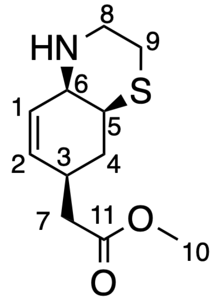

Compound 61:
Compound **36** (75 mg, 0.099 mmol) was placed in a test tube with ACN (2 mL). 2,3-Dichloro-5,6-dicyano-1,4-benzoquinone (45 mg, 0.21 mmol) in ACN (2 mL) was added dropwise, then the reaction allowed to stir at room temperature for 10 minutes The reaction was washed three times (H_2O:$NaHCO_3$/DCM; 30mL/30mL) and dried over anhydrous Na_2SO_4. The organic layer was evaporated in vacuo. The resulting yellow film was dissolved in minimal DCM and pipetted in 100 mL of stirring hexane. A brown solid precipitated out and was collected on a 15 mL fine-porosity fitted disk and washed two times with diethyl ether (5ml). The resulting filtrate was evaporated to dryness to yield a clear oil (16 mg, 0.069 mmol, 67%).

^{1}H NMR (800 MHz, CD_3CN) δ: 5.65 (m, 1H, H1), 5.58 (d, 1H, H2), 3.64 (s, 3H, H10), 3.41 (d, 1H, H6), 3.22 (m, 1H, H8), 2.85 (t, 1H, H8), 2.72 (t, 1H, H9), 2.64 (m, 1H, H3), 2.53 (dd, 1H, H5), 2.33 (m, 1H, H7), 2.30 (m, 1H, H7), 2.09 (m, 1H, H9), 2.03 (m, 1H, H4), 1.86 (m, 1H, H4). **^{13}C NMR**

(201 MHz, CD_3CN) δ: 173.4 (1C, C11), 132.4 (1C, C2), 130.7 (1C, C1), 53.8 (1C, C6), 52.0 (1C, C12), 48.2 (1C, C8), 40.4 (1C, C7), 36.2 (1C, C5), 35.5 (1C, C3), 32.1 (1C, C4), 23.3 (1C, C9). **HRMS (APCI$^+$, deconvoluted):** [M] calcd. for $C_{11}H_{17}NO_2S$, 227.0980; found, 227.0983.

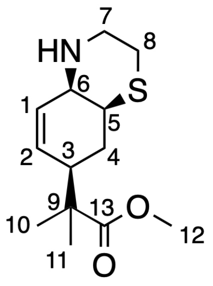

Compound 62:

Compound **37** (75 mg, 0.099 mmol) was placed in a test tube with ACN (2 mL). 2,3-Dichloro-5,6-dicyano-1,4-benzoquinone (45 mg, 0.20 mmol) in ACN (2 mL) was added dropwise, then the reaction allowed to stir at room temperature for 10 minutes The reaction was washed three times (H_2O:$NaHCO_3$/DCM; 30mL/30mL) and dried over anhydrous Na_2SO_4. The organic layer was evaporated in vacuo. The resulting yellow film was dissolved in minimal DCM and pipetted in 100 mL of stirring hexane. A brown solid precipitated out and was collected on a 15 mL fine-porosity fitted disk and washed two times with diethyl ether (5ml). The resulting filtrate was evaporated to dryness to yield a clear oil (17 mg, 0.067 mmol, 67%).

^{1}H NMR (800 MHz, CD_3CN) δ: 55.70 (m, 1H, H1), 5.53 (d, 1H, H2), 3.63 (s, 3H, H12), 3.37 (d, 1H, H6), 3.21 (m, 1H, H7), 2.84 (t, 1H, H7), 2.76 (t, 1H, H4), 2.58 (d, 1H, H5), 2.47 (d, 1H, H3), 2.12 (d, 1H, H8), 2.06 (d, 1H, H4), 1.63 (t, 1H, H8), 1.13 (s, 3H, H10), 1.11 (s, 3H, H11).**^{13}C NMR (201 MHz, CD_3CN) δ:** 178.4 (1C, C13), 132.0 (1C, C1), 130.4 (1C, C2), 53.9 (1C, C6), 52.3 (1C, C12), 48.4 (1C, C7), 46.0 (1C, C9), 45.6 (1C, C5), 36.4 (1C, C3), 27.1 (1C, C8), 23.4 (1C, C4), 22.4 (1C, C10), 22.1 (1C, C11). **HRMS (ESI$^+$):** [M+H] calcd. for $C_{13}H_{22}NO_2S^+$, 256.1366; found, 256.1361.

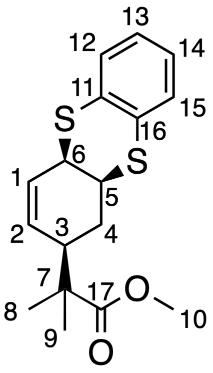

Compound 63:

Compound **39** (85 mg, 0.10 mmol) was placed in a test tube with ACN (2 mL). 2,3-Dichloro-5,6-dicyano-1,4-benzoquinone (47 mg, 0.21 mmol) in ACN (2 mL) was added dropwise, then the reaction allowed to stir at room temperature for 10 minutes The reaction was washed three times (H_2O:$NaHCO_3$/DCM; 30mL/30mL) and dried over anhydrous Na_2SO_4. The organic layer was evaporated in vacuo. The resulting yellow film was dissolved in minimal DCM and pipetted in 100 mL of stirring hexane. A brown solid precipitated out and was collected on a 15 mL fine-porosity fitted disk and washed two times with diethyl ether (5ml). The resulting filtrate was evaporated to dryness to yield a clear oil (19 mg, 0.059 mmol, 57%).

^{1}H NMR (800 MHz, CD_3CN) δ: 7.32 (dd, *J* = 7.8, 1.5 Hz, 1H, H13), 7.31 (dd, *J* = 7.7, 1.5 Hz, 1H, H12), 7.13 (td, *J* = 7.5, 1.4 Hz, 1H, H11), 7.09 (td, *J* = 7.5, 1.5 Hz, 1H, H14), 5.90 (ddd, *J* = 9.9, 5.1, 2.7 Hz, 1H, H1), 5.77 (dq, *J* = 10.0, 1.8 Hz, 1H, H2), 3.94 (m, 1H, H6), 3.65 (d, *J* = 0.9 Hz, 3H, H10), 3.41 (dtd, *J* = 6.4, 3.3, 1.4 Hz, 1H, H5), 2.64 (ddt, *J* = 12.2, 5.4, 2.1 Hz, 1H, H3), 2.07 (q, *J* = 12.5 Hz, 1H, H4A), 1.80 (dddt, *J* = 12.8, 5.6, 3.1, 1.5 Hz, 1H, H4B), 1.15 (s, 3H, H8), 1.13 (d, *J* = 2.1 Hz, 3H, H9). **^{13}C NMR (201 MHz, CD_3CN) δ:** 178.0 (1C, C17), 137.5 (1C, C15), 135.3 (1C, C16), 133.2 (1C, C2), 130.6 (1C, C13), 129.2 (1C, C12), 127.8 (1C, C11), 126.2 (1C, C14), 125.9 (1C, C1), 52.4 (1C, C10), 45.9 (1C, C5), 45.6 (1C, C7), 45.1 (1C, C6), 44.3 (1C, C3), 29.1 (1C, C4), 22.6 (1C, C8), 22.1 (1C, C9).

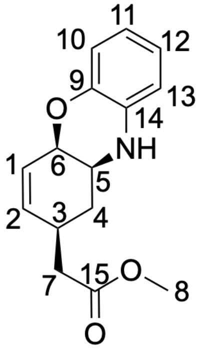

Compound 64:

Compound **32** (96 mg, 0.13 mmol) was dissolved in ACN (10 mL) and left to stir at room temperature for 1 week. The solution was then evaporated in vacuo. The resulting brown oil was dissolved in minimal DCM and pipetted in 100ml of stirring hexanes. A brown solid precipitated out and was collected on a 15 mL fine-porosity fitted disk and washed two times with diethyl ether (5ml). The resulting filtrate was evaporated to dryness to yield a clear oil (16 mg, 0.048 mmol, 47%).

^{1}H NMR (800 MHz, CD_3CN) δ 6.70 (td, *J* = 7.6, 1.5 Hz, 1H, H13), 6.67 (dd, *J* = 8.0, 1.4 Hz, 1H, H11), 6.56 (dd, *J* = 7.8, 1.6 Hz, 1H, H12), 6.49 (m, 1H, H10), 5.95 (ddd, *J* = 9.9, 5.0, 2.6 Hz, 1H, H2), 5.90 (d, *J* = 10.0 Hz, 1H, H1), 4.23 (s, 1H, H6), 3.63 (s, 3H, H8), 3.50 (dt, *J* = 12.4, 3.2 Hz, 1H, H5), 2.40 (dd, *J* = 16.0, 7.2 Hz, 1H, H7A), 2.32 (dd, *J* = 15.9, 7.7 Hz, 1H, H7B), 1.92 (m, 1H, H4A), 1.77 (m, 1H, H4B). **NMR (201 MHz, CD_3CN):** δ 183.0 (1C, C15), 137.3 (1C, C1), 126.1 (1C, C2), 122.6 (1C, C12), 117.6 (1C, C11), 117.0 (1C, C13), 115.0 (1C, C10), 68.7 (1C, C6),

52.0 (1C, C8), 49.9 (1C, C5), 40.2 (1C, C7), 34.3 (1C, C3), 33.1 (1C, C4). **HRMS (APCI$^+$, deconvoluted):** [M] calcd. for $C_{15}H_{17}NO_3$, 259.1208; found, 259.1211.

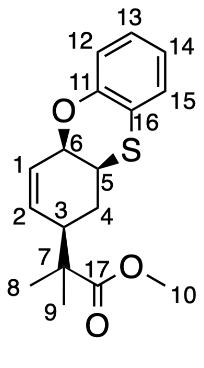

Compound 65:

Compound **40** (75 mg, 0.093 mmol) was placed in a test tube with ACN (2 mL). 2,3-Dichloro-5,6-dicyano-1,4-benzoquinone (42 mg, 0.19 mmol) in ACN (2 mL) was added dropwise, then the reaction allowed to stir at room temperature for 10 minutes The reaction was washed three times (H_2O:$NaHCO_3$/DCM; 30mL/30mL) and dried over anhydrous Na_2SO_4. The organic layer was evaporated in vacuo. The resulting yellow film was dissolved in minimal DCM and pipetted in 100 mL of stirring hexane. A brown solid precipitated out and was collected on a 15 mL fine-porosity fitted disk and washed two times with diethyl ether (5ml). The resulting filtrate was evaporated to dryness to yield a clear oil (17 mg, 0.056 mmol, 60%).

^{1}H NMR (800 MHz, CD_3CN) δ: 7.07 (dd, J = 7.8, 1.8 Hz, 1H, H15), 6.99 (tdd, J = 7.3, 1.9, 0.7 Hz, 1H, H13), 6.88 (td, J = 7.5, 1.4 Hz, 1H, H14), 6.82 (dd, J = 8.0, 1.4 Hz, 1H, H12), 6.05 (ddd, J = 10.2, 5.3, 2.7 Hz, 1H, H1), 5.88 (dt, J = 10.2, 1.9 Hz, 1H, H2), 4.29 (m, 1H, H6), 3.64 (s, 3H, H10), 3.43 (dt, J = 13.2, 3.0 Hz, 1H, H5), 2.70 (m, 1H, H3), 1.84 (m, 1H, H4A), 1.77 (td, J = 12.9, 11.3 Hz, 1H, H4B), 1.13 (s, 3H, H8), 1.11 (s, 3H, H9). **^{13}C NMR (201 MHz, CD_3CN): δ** 178.1 (1C, C17), 153.0 (1C, C11), 134.9 (1C, C2), 127.5 (1C, C1), 127.4 (1C, C15), 126.1 (1C, C13), 123.1 (1C, C14), 119.3 (1C, C16), 119.2 (1C, C12), 69.9 (1C, C6), 52.4 (1C, C10), 45.6 (1C, C7), 45.5 (1C, C3), 39.4 (1C, C5), 29.0 (1C, C4), 22.5 (1C, C8), 22.0 (1C, C9). **HRMS (APCI$^+$, deconvoluted):** [M] calcd. for $C_{17}H_{20}O_3S$, 304.1133; found, 304.1138.

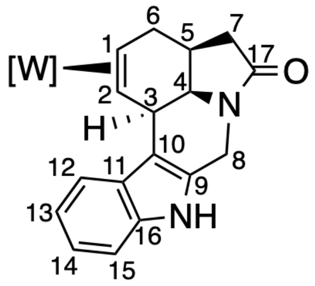

Compound 67:
Compound **2** (170 mg, 0.232 mmol) was placed in a test tube with ACN (2 mL), and chilled to -30 °C. After 10 min, a 1 M HOTf/ACN (0.464 mL, 0.464 mmol) solution was added to the test tube and the solution was allowed to stir at -30 °C for 30 min. In a separate test tube, 2-(aminomethyl)indole (339 mg, 2.32 mmol) in ACN (2 mL) was cooled at -30 °C for 30 min. After the time elapsed, the former solution was added to the latter, dropwise. The reaction stirred at -30 °C for 1 day and room temperature for 1 day. The reaction was washed three times (H_2O:Na_2CO_3/DCM; 30mL/30mL) and dried over anhydrous Na_2SO_4. The organic layer was evaporated in vacuo. The resulting yellow film was dissolved in minimal DCM and loaded on a basic alumina column that was gradient eluded with hexane/ethyl acetate followed by ethyl acetate/Methanol. The band at 70:30 Ethyl acetate/methanol was evaporated to dryness, dissolved in minimal DCM and pipetted in 25 mL of stirring hexane. An off-white solid precipitated out and was collected on a 15 mL fine-porosity fitted disk, washed with hexane (2 × 10 mL) and desiccated overnight to yield compound **59** (105 mg, 0.137 mmol, 61%).

^{1}H NMR (800 MHz, CD_3CN) δ: 8.23 (d, *J* = 2.4 Hz, 1H, TpB3), 7.94 (d, *J* = 2.4 Hz, 1H, TpA3), 7.87 (d, *J* = 2.5 Hz, 1H, TpB5), 7.74 (d, *J* = 2.4 Hz, 1H, TpC5), 7.53 (d, *J* = 2.3 Hz, 1H, TpC3), 7.51 (d, *J* = 2.4 Hz, 1H, TpA5), 7.47 (d, *J* = 7.8 Hz, 1H, H12), 7.35 (d, *J* = 8.2 Hz, 1H, H15), 7.03 (ddd, *J* = 8.2, 7.0, 1.3 Hz, 1H, H14), 6.86 (ddd, *J* = 8.0, 7.0, 1.0 Hz, 1H, H13), 6.43 (t, *J* = 2.2 Hz, 1H, TpB4), 6.16 (t, *J* = 2.3 Hz, 1H, TpC4), 5.47 (t, *J* = 2.3 Hz, 1H, TpA4), 4.86 (d, *J* = 17.1 Hz, 1H, H8A), 4.29 (dd, *J* = 7.0, 3.8 Hz, 1H, H3), 4.21 (dd, *J* = 17.0, 1.2 Hz, 1H, H8B), 3.98 (dd, *J* = 8.4, 3.9 Hz, 1H, H4), 3.02 (ddq, *J* = 13.8, 9.4, 4.2 Hz, 1H, H5), 2.64 (m, 2H, H7A/H7B), 2.57 (m, 2H, H1/H6A), 2.42 (ddd, *J* = 12.9, 8.3, 4.5 Hz, 1H, H6B), 1.45 (ddd, *J* = 9.8, 7.0, 2.9 Hz, 1H, H2), 1.15 (d, *J* = 8.4 Hz, 9H, PMe3).**^{13}C NMR (201 MHz, CD_3CN) δ:** 175.67 (1C, C17), 147.64 (1C, TpA3), 144.86 (1C, TpB3), 142.79 (1C, TpC3), 138.02 (1C, C16), 137.57 (1C, TpC5), 136.95 (1C, TpB5), 136.79 (1C, TpA5), 131.29 (1C, C9), 128.68 (1C, C11), 122.08 (1C, C14), 120.38 (1C, C12), 119.84 (1C, C13), 117.26 (1C, C10), 111.90 (1C, C15), 107.65 (1C, TpB4), 106.58 (1C, TpC4), 105.64 (1C, TpA4), 64.51 (1C, C4), 55.10 (1C, C2), 53.27 (1C, C1), 39.92 (1C, C8), 37.71 (1C, C7), 36.41 (1C, C3), 33.29 (1C, C6), 33.20 (1C, C5), 13.57 (3C, PMe3). **HRMS (APCI$^+$, deconvoluted):** [M] calcd. for $C_{29}H_{35}BN_9O_2PW$, 767.2254; found, 767.2242.

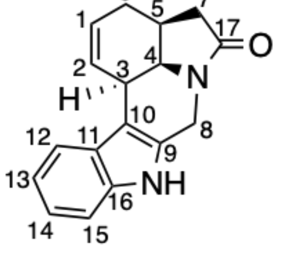

Compound 68:
Compound **67** (105 mg, 0.137 mmol) was dissolved in ACN (10 mL) and left to stir at room temperature for 1 week. The solution was then evaporated in vacuo. The resulting brown oil was dissolved in minimal DCM and pipetted in 100ml of stirring hexanes. A brown solid precipitated

out and was collected on a 15 mL fine-porosity fitted disk and washed two times with diethyl ether (5ml). The resulting filtrate was evaporated to dryness to yield a clear oil (20 mg, 0.137 mmol, 55%).

^{1}H NMR (800 MHz, CD_3CN) δ: 7.51 (d, *J* = 7.8 Hz, 1H, H15), 7.37 (dt, *J* = 8.2, 0.9 Hz, 1H, H12), 7.14 (ddd, *J* = 8.2, 7.0, 1.2 Hz, 1H, H13), 7.06 (ddd, *J* = 7.9, 7.0, 1.1 Hz, 1H, H14), 5.89 (m, 1H, H1), 5.85 (m, 1H, H2), 4.80 (d, *J* = 16.8 Hz, 1H, H8A), 4.15 (m, 2H, H3/H8B), 3.65 (s, 1H, H4), 2.88 (m, 1H, H5), 2.55 (ddd, *J* = 16.7, 9.0, 1.4 Hz, 1H, H7A), 2.26 (m, 1H, H6A), 2.05 (m, 2H, H6B/H7B). **^{13}C NMR (201 MHz, CD_3CN) δ:** 175.06 (1C, C17), 137.92 (1C, C16), 131.96 (1C, C2), 130.63 (1C, C9), 127.37 (1C, C11), 126.64 (1C, C1), 122.58 (1C, C14), 120.20 (1C, C13), 118.69 (1C, C12), 112.18 (1C, C15), 111.23 (1C, C10), 57.89 (1C, C4), 39.57 (1C, C3), 38.80 (1C, C8), 32.48 (1C, C7), 29.81 (1C, C6), 27.48 (1C, C5). **HRMS (APCI$^+$):** [M+H] calcd. for $C_{17}H_{17}N_2O^+$, 265.1335; found, 265.1339.

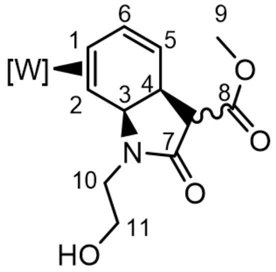

Compound 69:

Compound **4** (300mg, 0.379 mmol) was placed in a test tube with ACN (2 mL), and chilled to -30 °C. After 10 min, a 1 M HOTf/ACN (0.758 mL, 0.758 mmol) solution was added to the test tube and the solution was allowed to stir at -30 °C for 30 min. In a separate test tube, ethanolamine (0.229 mL, 3.79 mmol) in ACN (2 mL) was cooled at -30 °C for 30 min. After the time elapsed, the former solution was added to the latter, dropwise. The reaction stirred at -30 °C for 1 day and room temperature for 1 day. The reaction was washed three times (H_2O:$NaHCO_3$/DCM; 30 mL/30mL) and dried over anhydrous Na_2SO_4. The organic layer was evaporated in vacuo. The resulting yellow film was dissolved in minimal DCM and pipetted in 25 mL of stirring hexane. A white solid precipitated out and was collected on a 15 mL fine-porosity fitted disk, washed with hexane (2 × 10 mL) and desiccated overnight to yield compound **69** (238 mg, 0.322 mmol, 85%).

^{1}H NMR (800 MHz, CD_3CN) δ: 8.08 (d, J = 2.1 Hz, 2H), 7.98 (d, J = 2.1 Hz, 1H), 7.90 (d, J = 2.3 Hz, 1H), 7.90 (d, J = 2.4 Hz, 2H), 7.82 (d, J = 2.4 Hz, 1H), 7.53 (d, J = 2.2 Hz, 1H), 6.52 (ddt, J = 10.0, 4.8, 2.3 Hz, 1H), 6.41 (t, J = 2.1 Hz, 2H), 6.34 (m, 3H), 4.97 (d, J = 6.1 Hz, 1H), 4.55 (dd, J = 9.7, 2.1 Hz, 1H), 3.76 (s, 2H), 3.43 (m, 4H), 3.39 (m, 1H), 3.26 (m, 1H), 3.05 (s, 1H), 2.85 (m, 3H), 1.35 (d, J = 9.4 Hz, 1H), 1.22 (dd, J = 8.5, 3.8 Hz, 15H). **^{13}C NMR (201 MHz, CD_3CN) δ:** 172.1, 171.6, 144.6, 143.3, 142.2 (d, J = 4.1 Hz), 138.1 (d, J = 7.5 Hz), 137.4 (m), 133.1 (d, J = 3.4 Hz), 118.0, 107.6, 107.3 (d, J = 8.0 Hz), 107.0, 62.7, 60.8, 57.8, 53.0, 48.9 (d, J = 9.5 Hz), 47.7, 43.4, 38.3, 13.7 (dd, J = 29.1, 5.5 Hz). **HRMS (APCI$^+$, deconvoluted):** [M] calcd. for $C_{24}H_{34}BN_8O_5PW$, 740.1998; found, 740.1974

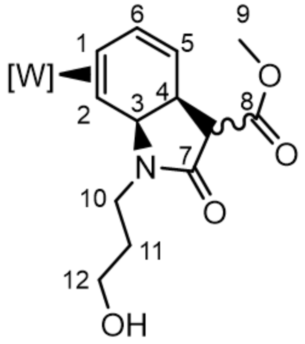

Compound 70:

Compound **4** (379 mg, 0.479 mmol) was placed in a test tube with ACN (2 mL), and chilled to -30 °C. After 10 min, a 1 M HOTf/ACN (0.958 mL, 0.958 mmol) solution was added to the test tube and the solution was allowed to stir at -30 °C for 30 min. In a separate test tube, propanolamine (0.366 mL, 4.79 mmol) in ACN (2 mL) was cooled at -30 °C for 30 min. After the time elapsed, the former solution was added to the latter, dropwise. The reaction stirred at -30 °C for 1 day and room temperature for 1 day. The reaction was washed three times (H_2O:$NaHCO_3$/DCM; 30 mL/30mL) and dried over anhydrous Na_2SO_4. The organic layer was evaporated in vacuo. The resulting yellow film was dissolved in minimal DCM and pipetted in 25 mL of stirring hexane. A white solid precipitated out and was collected on a 15 mL fine-porosity fitted disk, washed with hexane (2 × 10 mL) and desiccated overnight to yield compound **70** (320 mg, 0.420 mmol, 89.0%).

^{1}H NMR (800 MHz, CD_3CN) δ: 8.06 (1H, d, J = 2.0 Hz, Tp3B), 7.94 (1H, d, J = 2.0 Hz, Tp3A), 7.88 (1H, d, J = 2.1 Hz, Tp5C), 7.87 (1H, d, J = 2.3 Hz, Tp5B), 7.80 (1H, d, J = 2.3 Hz, Tp5A), 7.49 (1H, d, J = 2.0 Hz, Tp3C), 6.49 (1H, m, H6), 6.38 (1H, t, J = 2.1 Hz, Tp4B), 6.31 (2H, m, Tp4A/Tp4C), 4.92 (1H, d, J = 6.1 Hz, H3), 4.54 (1H, d, J = 9.7 Hz, H5), 3.73 (3H, s, H13), 3.41 (1H, m, H4), 3.38 (2H, t, J = 6.0 Hz, H11), 3.30 (2H, m, H9), 3.05 (1H, s, H7), 2.82 (1H, m, H1), 1.54 (1H, m, H10), 1.38 (1H, m, H10), 1.29 (1H, d, J = 9.5 Hz, H2), 1.19 (9H, d, J = 8.8 Hz, PMe_3). **^{13}C NMR (201 MHz, CD_3CN) δ:** 171.9 (1C, H8/H12), 171.8 (1C, H8/H12), 144.7 (1C, Tp3B), 143.1 (1C, Tp3A), 142.1 (1C, Tp3C), 138.1 (1C, Tp5C)), 137.5 (1C, Tp5B), 137.5 (1C, Tp5A), 133.2 (1C, d, J = 3.8 Hz, C6), 118.0 (1C, C5), 107.6 (1C, Tp4B), 107.3 (1C, Tp4C), 107.1 (1C, Tp4A), 62.7 (1C, C3), 58.9 (1C, C11), 57.9 (1C, C7), 53.0 (1C, C13), 49.0 (1C, d, J = 10.0 Hz, C1), 47.6 (1C, C2), 38.3 (1C, C4), 37.1 (1C, C9), 31.8 (1C, C10), 13.7 (3C, d, J = 29.1 Hz, PMe_3). **HRMS (APCI$^+$, deconvoluted):** [M] calcd. for $C_{25}H_{36}BN_8O_5PW$, 754.2154; found, 754.2151. **IR (neat):** *v*(NO) 1560 cm^{-1}, *v*(CO amide) 1661 cm^{-1}, *v*(CO ester) 1731 cm^{-1}, *v*(BH) 2491 cm^{-1}, *v*(OH) 3419 cm^{-1}.

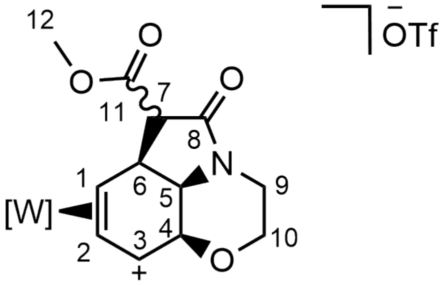

Compound 73:

Compound **69** (238 mg, 0.322 mmol) was placed in a test tube with DCM/MeOH (5 mL: 5 mL). In a separate test tube, Selectfluor™ (137 mg, 0.386 mmol) and Na_2CO_3 (102 mg, 0.965 mmol) were combined in ACN (2 mL). Then, the former solution was added to the latter, dropwise. The reaction stirred at room temperature for 2 h. The reaction was washed three times (H_2O:$NaHCO_3$/DCM; 30 mL/30mL) and dried over anhydrous Na_2SO_4. The organic layer was evaporated in vacuo to yield **71** as a red oil. This was carried forward without further purification. Et_2O (90 mL) was chilled to -30°C and added to a 50 mL Erlenmeyer flask charged with a stir pea. A 1 M HOTf/ACN (1.61 mL, 1.61 mmol) solution was added to the Erlenmeyer flask and the solution allowed to stir for 1 min. Compound **71** in ACN (2 mL) was then added to the Erlenmeyer flask dropwise and the solution allowed to stir for 1 min. A white solid precipitated out and was collected on a 15 mL fine-porosity fitted disk, washed with cold ether (2 × 10 mL) and desiccated overnight to yield compound **73** (211 mg, 0.238 mmol, 74 % over two steps).

^{1}H NMR (600 MHz, $(CD_3)_2CO$) δ: 8.60 (1H, d, J = 2.3 Hz, Tp3B), 8.34 (1H, d, J = 2.4 Hz, Tp3C), 8.27 (1H, d, J = 2.3 Hz, Tp3A), 8.23 (1H, d, J = 2.4 Hz, Tp5C), 8.21 (1H, dt, J = 2.4, 0.8 Hz, Tp5B), 8.02 (1H, dt, J = 2.5, 0.8 Hz, Tp5A), 6.66 (2H, q, J = 2.2 Hz, Tp4B/Tp4C), 6.48 (1H, t, J = 2.4 Hz, Tp4A), 5.95 (1H, td, J = 7.8, 1.5 Hz, H2), 5.73 (1H, d, J = 8.0 Hz, H3), 5.64 (1H, d, J = 6.0 Hz, H4), 4.76 (1H, ddd, J = 15.4, 7.5, 1.8 Hz, H1), 4.06 (1H, t, J = 5.9 Hz, H5), 3.86 (3H, m, H9/H10), 3.74 (3H, s, H12), 3.62 (2H, m, H6/H7), 3.03 (1H, ddd, J = 13.1, 9.4, 6.3 Hz, H9), 1.36 (9H, d, J = 10.0 Hz, PMe_3). **^{13}C NMR (201 MHz, $(CD_3)_2CO$) δ:** 169.5 (1C, C11), 168.1 (1C, C8), 148.5 (1C, Tp3A), 146.3 (1C, Tp3B), 144.0 (1C, Tp3C), 139.9 (1C, Tp5B/Tp5C), 139.8 (1C, Tp5A), 122.0 (1C, q, J = 322.4, CF_3), 117.0 (1C, C3), 111.1 (1C, d, J = 3.7 Hz, C2), 109.8 (1C, Tp4B), 109.4 (1C, Tp4C), 108.4 (1C, Tp4A), 73.5 (1C, d, J = 10.9 Hz, C1), 70.2 (1C, H4), 60.0 (1C, C7), 59.6 (1C, C10), 53.2 (1C, C12), 50.9 (1C, C5), 40.5 (1C, C9), 40.3 (1C, d, J = 2.8 Hz, C6), 13.1 (3C, d, J = 33.3 Hz, PMe_3). **HRMS (APCI$^+$, deconvoluted):** [M] calcd. for $C_{24}H_{33}BN_8O_5PW^+$, 739.1914; found, 739.1913.

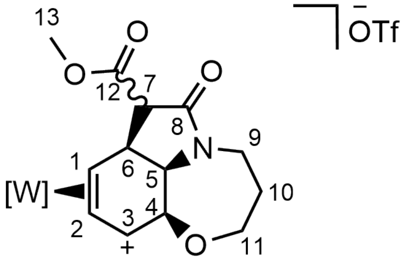

Compound 74:

Compound **70** (241 mg, 0.319 mmol) was placed in a test tube with DCM/MeOH (5 mL: 5 mL). In a separate test tube, Selectfluor™ (136 mg, 0.383 mmol) and Na_2CO_3 (102 mg, 0.959 mmol) were combined in ACN (2 mL). Then, the former solution was added to the latter, dropwise. The reaction stirred at room temperature for 2 h. The reaction was washed three times (H_2O:$NaHCO_3$/DCM; 30 mL/30mL) and dried over anhydrous Na_2SO_4. The organic layer was evaporated in vacuo to yield **72** as a red oil. This was carried forward without further purification. Et_2O (90 mL) was chilled to -30°C and added to a 50 mL Erlenmeyer flask charged with a stir pea. A 1 M HOTf/ACN (1.60 mL, 1.60 mmol) solution was added to the Erlenmeyer flask and the solution allowed to stir for 1 min. Compound **72** in ACN (2 mL) was then added to the Erlenmeyer flask dropwise and the solution allowed to stir for 1 min. A white solid precipitated out and was collected on a 15 mL fine-porosity fitted disk, washed with cold ether (2 × 10 mL) and desiccated overnight to yield compound **74** (232 mg, 0.257 mmol, 80 % over two steps).

^{1}H NMR (800 MHz, $(CD_3)_2CO$) δ: 8.67 (1H, d, J = 2.3 Hz, Tp3B), 8.50 (1H, d, J = 2.3 Hz, Tp3A), 8.37 (1H, d, J = 2.4 Hz, Tp3C), 8.23 (1H, d, J = 2.4 Hz, Tp5C), 8.19 (1H, d, J = 2.6 Hz, Tp5B), 8.00 (1H, d, J = 2.5 Hz, Tp5A), 6.67 (1H, t, J = 2.3 Hz, Tp4B), 6.63 (1H, t, J = 2.3 Hz, Tp4C), 6.43 (1H, t, J = 2.4 Hz, Tp4A), 6.34 (1H, m, H3), 5.49 (1H, t, J = 7.7 Hz, H2), 5.00 (1H, dd, J = 6.8, 3.1 Hz, H4), 4.87 (1H, m, H1), 4.32 (1H, m, H11), 4.14 (1H, t, J = 7.7 Hz, H5), 4.06 (1H, m, H9), 3.89 (1H, td, J = 12.4, 2.6 Hz, H11), 3.80 (4H, m, H6/H13), 3.33 (1H, d, J = 10.9 Hz, H7), 3.07 (1H, ddd, J = 14.4, 12.2, 2.6 Hz, H9), 2.07 (1H, under $(CH_3)_2CO$, 1H), 1.64 (1H, dq, J = 14.5, 2.7 Hz, H10), 1.41 (9H, d, J = 10.1 Hz, PMe_3). **^{13}C NMR (201 MHz, $(CD_3)_2CO$) δ:** 170.3 (1C, C12), 168.8 (1C, C8), 149.1 (1C, Tp3A), 145.4 (1C, Tp3B), 143.8 (1C, Tp3C), 140.0 (1C, Tp5C), 139.7 (1C, Tp5A), 139.6 (1C, Tp5B), 123.6 (1C, C3), 121.9 (1C, q, J = 321.1 Hz, CF_3) 109.6 (1C, Tp4B), 109.2 (1C, Tp4C), 108.3 (1C, Tp4A), 101.8 (1C, C2), 77.3 (1C, C4), 76.3 (1C, C11), 73.6 (1C, d, J = 13.0 Hz, C1), 61.8 (1C, C7), 57.9 (1C, C5), 53.0 (1C, C13), 43.9 (1C, C9), 38.8 (1C, C6), 28.4 (1C, C10), 12.6 (3C, d, J = 33.4 Hz, PMe_3). **HRMS ($APCI^+$, deconvoluted):** [M] calcd. for $C_{25}H_{35}BN_8O_5PW^+$, 753.2070; found, 753.2080.

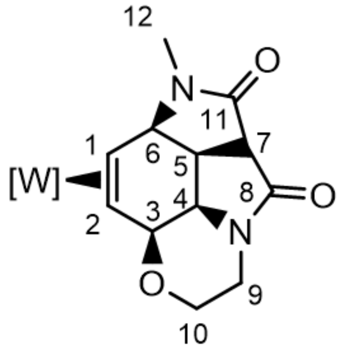

Compound 75:

Compound **73** (347 mg; 0.391 mmol) taken in 15 mL ACN and cooled to -30°C. To this 2M $MeNH_2$ in THF (1.95 mL) was added. The reaction was stirred at -30°C for 2 days, then warmed to 50°C for 1.5 hr. The reaction was quenched with saturated $NaHCO_3$ solution and extracted with DCM.

The organic layer was dried over Na_2SO_4 and evaporated to dryness. The oil was then dissolved in minimal DCM, precipitated into 100 mL of stirring 4:1 Hexanes: Et_2O, and collected on a fine porosity fritted disc as a white powder **75** (254 mg, 0.345 mmol, 88%).

^{1}H NMR (800 MHz, $(CD_3)_2CO$) δ: 8.19 (1H, d, J = 2.0 Hz, Tp3B), 8.00 (3H, m, Tp3A/Tp5B/Tp5C), 7.85 (1H, d, J = 2.5 Hz, Tp5A), 7.78 (1H, d, J = 2.2 Hz, Tp3C), 6.45 (1H, t, J = 2.2 Hz, Tp4B), 6.37 (1H, t, J = 2.2 Hz, Tp4C), 6.30 (1H, t, J = 2.2 Hz, Tp4A), 4.57 (1H, d, J = 7.3 Hz, H6), 4.31 (1H, ddd, J = 5.2, 3.0, 1.5 Hz, H3), 4.14 (1H, dd, J = 9.1, 5.6 Hz, H4), 3.90 (1H, ddd, J = 13.3, 8.9, 1.9 Hz, H9), 3.67 (1H, q, J = 9.4 Hz, H10), 3.50 (1H, ddd, J = 9.8, 7.7, 1.9 Hz, H10), 3.29 (2H, m, H5/H7), 3.14 (1H, m, H9), 3.00 (3H, s, H12), 2.97 (1H, m, H1), 1.28 (9H, d, J = 8.5 Hz, PMe_3), 1.22 (1H, m, H2). **^{13}C NMR (201 MHz, $(CD_3)_2CO$) δ:** 172.6 (1C, C8), 169.5 (1C, C11), 144.6 (1C, Tp3A), 144.0 (1C, Tp3B), 141.9 (1C, Tp3C), 138.0 (1C, Tp5C), 137.5 (1C, Tp5B), 137.3 (1C, Tp5A), 107.5 (1C, Tp4B), 107.3 (1C, Tp4C), 107.0 (1C, Tp4A), 75.1 (1C, C3), 64.0 (1C, C10), 61.8 (1C, d, J = 3.9 Hz, C6), 56.1 (1C, C4), 51.7 (1C, C7), 49.2 (1C, d, J = 11.1 Hz, C1), 48.9 (1C, C2), 36.8 (1C, C9), 30.6 (1C, C5), 28.3 (1C, C12), 12.9 (3C, d, J = 28.6 Hz, PMe_3). **IR (neat):** *v*(NO) 1548 cm^{-1}, *v*(CO) 1699 cm^{-1}, *v*(BH) 2488 cm^{-1}

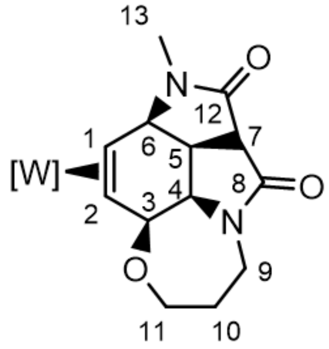

Compound 76:

Compound **74** (260 mg, 0.287 mmol) was placed in a test tube, with ACN (5 mL) and chilled to -30°C. After 10 min, 2 M $MeNH_2$ in THF (0.717 mL, 1.44 mmol) solution was added to the test tube followed by 3 drops of 1M HOTf in ACN and the solution was allowed to stir at -30 °C for 4 days then at room temperature for 3 days. The reaction was washed three times (H_2O:$NaHCO_3$/DCM; 60 mL/60mL) and dried over anhydrous Na_2SO_4. The organic layer was evaporated in vacuo. The resulting yellow film was dissolved in minimal DCM and pipetted in 110 mL of stirring pentane. Precipitation was induced twice to collect all the material. An off-white solid precipitated out and was collected on a 15 mL fine-porosity fritted disk, washed pentane (2 × 10 mL) desiccated overnight to yield compound **76** (175 mg, 0.233 mmol, 81%).

^{1}H NMR (800 MHz, CD_3CN) δ: 8.11 (1H, d, J = 2.0 Hz, Tp3B), 7.90 (1H, d, J = 2.4 Hz, Tp5B), 7.89 (1H, d, J = 2.3 Hz, Tp5C), 7.88 (1H, d, J = 2.1 Hz, Tp3A), 7.80 (1H, d, J = 2.5 Hz, Tp5A), 7.58 (1H, d, J = 2.2 Hz, Tp3C), 6.41 (1H, t, J = 2.2 Hz, Tp4B), 6.32 (2H, t, J = 2.3 Hz, Tp4A, Tp4C), 4.47 (1H, d, J = 7.1 Hz, H6), 4.25 (1H, m, H3), 4.12 (1H, dd, J = 9.8, 5.6 Hz, H4), 3.90 (1H, m, H9), 3.87 (1H, dd, J = 12.8, 5.7 Hz, H11), 3.32 (1H, m, H11), 3.14 (3H, m, H5, H7, H9), 2.97 (3H, s, H13), 2.79 (1H, ddd, J = 12.5, 10.6, 2.0 Hz, H1), 1.85 (1H, m, H10), 1.39 (1H, d, J = 13.2, H10), 1.17 (10H, d, J = 8.5 Hz, H2, PMe_3). **^{13}C NMR (201 MHz, CD_3CN) δ:** 170.9 (1C, C8),

169.7 (1C, C12), 144.6 (1C, Tp3A), 144.2 (1C, Tp3B), 142.0 (1C, Tp3C), 138.2 (1C, Tp5C), 137.7 (1C, Tp5B), 137.5 (1C, Tp5A), 107.8 (1C, Tp4B), 107.5 & 107.4 (2C, Tp4A/Tp4C) , 85.3 (1C, C3), 74.0 (1C, C11), 62.6 (1C, d, J = 3.6 Hz, C6), 59.9 (1C, C4), 51.8 (1C, C7), 51.2 (1C, C2), 48.6 (1C, d, J = 11.2 Hz, C1), 41.9 (1C, C9), 31.6 (1C, C5), 29.3 (1C, C10), 28.3 (1C, C13), 12.9 (1C, d, J = 29.0 Hz, PMe_3).

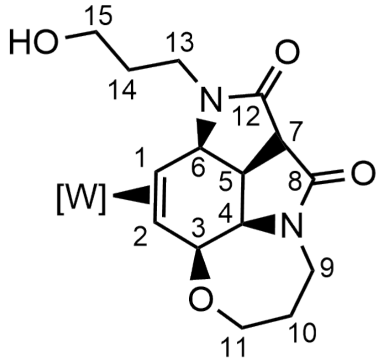

Compound 77:

Compound **74** (200 mg, 0.222 mmol) was placed in a test tube, with MeCN (5 mL) and chilled to -30°C. After 10 min, 3-amino-1-propanol (85 µL, 1.11 mmol) was added to the test tube followed by 2 drops of 1M HOTf in ACN and the solution was allowed to stir at -30 °C for 4 days then at room temperature for 2 days. The reaction was washed three times (H_2O:$NaHCO_3$/DCM; 60 mL/60mL) and dried over anhydrous Na_2SO_4. The organic layer was evaporated in vacuo. The resulting yellow film was dissolved in minimal DCM and pipetted in 110 mL of stirring pentane. An off-white solid precipitated out and was collected on a 15 mL fine-porosity fritted disk, washed pentane (2 × 5 mL) desiccated overnight to yield compound **77** (137 mg, 0.172 mmol, 78%).

^{1}H NMR (800 MHz, Acetone) δ 8.21 (1H, d, J = 2.1 Hz, Tp3B), 8.02 (1H, d, J = 2.1 Hz, Tp3A), 8.01 (1H, d, J = 2.2 Hz, Tp5C), 8.00 (1H, d, *J* = 2.4 Hz, Tp5B), 7.85 (1H, bs, Tp5A), 7.79 (1H, d, J = 2.2 Hz, Tp3C), 6.46 (1H, t, J = 2.3 Hz, Tp4B), 6.37 (1H, t, J = 2.2 Hz, Tp4C), 6.31 (1H, bs, Tp4A), 4.71 (1H, d, J = 7.2 Hz, H6), 4.41 (1H, d, J = 5.0 Hz, H3), 4.25 (1H, dd, J = 10.0, 5.6 Hz, H4), 3.94 (1H, d, J = 14.2 Hz, H9), 3.91 (1H, dd, J = 12.7, 5.6 Hz, H11), 3.77 (1H, m, H13), 3.65 (1H, m, H15), 3.48 (2H, dt, J = 14.2, 5.5 Hz, H13/H15), 3.37 (1H, td, J = 12.7, 2.8 Hz, H11), 3.25 (2H, t, J = 11.0 Hz, H7/H9), 3.20 (1H, m, H5), 2.94 (1H, m, H1), 1.99 (1H, dddd, J = 21.5, 17.5, 10.7, 5.6 Hz, H10), 1.92 (1H, m, H14), 1.80 (1H, m, H14), 1.39 (1H, dt, J = 20.9, 7.5 Hz, H10), 1.29 (9H, d, J = 8.4 Hz, PMe_3). **^{13}C NMR (201 MHz, Acetone) δ** 171.1 (1C, C12), 170.7 (1C, C8), 144.5 (1C,Tp3A), 144.2 (1C, Tp3B), 142.0 (1C, Tp3C), 138.0 (1C, Tp5C), 137.5 (1C, Tp5B), 137.1 (1C,Tp5A), 107.6 (1C, Tp4B), 107.4 (1C,Tp4C), 107.1 (1C, Tp4A), 85.2 (1C, C3), 73.8 (1C, C11), 63.1 (1C,C6), 59.9 (1C, C4), 58.7 (1C, d, J = 26.1 Hz, C15), 51.6 (1C, C7), 51.5 (1C, C2), 49.4 (1C, d, J = 11.6 Hz, C1), 41.7 (1C, C9), 38.7 (1C, C13), 32.8 (1C, C14), 31.8 (1C, C5), 29.4 (1C, C10), 13.0 (3C, d, J = 28.5 Hz, PMe_3).

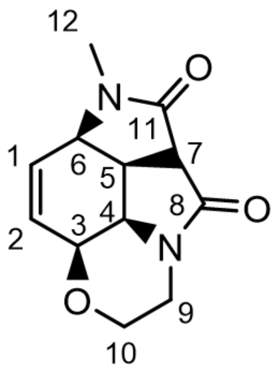

Compound 78:

Compound **75** (100 mg; 0.136 mmol) was taken in ACN (10 mL) followed by $AgNO_3$ (92.0 mg; 0.540 mmol) and stirred in darkness for 24 hrs. The reaction was diluted with DCM and extracted 3x with water. The aqueous layers were collected and dried in vacuo. The resulting crude mixture was then purified by preparative TLC (5% MeOH/DCM). The product (Rf = 0.35) was visualized as a burn spot with $KMnO_4$ and the corresponding silica band was scraped from the plate and loaded in a fritted disc. The product was eluted off with excess acetone and evaporated to an oil. The product was then picked up with HPLC water and filtered through a 0.22 µm PTFE syringe filter into a weighed vial, then evaporated to an oil (16 mg, 0.068 mmol, 50%).

^{1}H NMR (600 MHz, CD_3CN) δ: 6.09 (1H, dt, J = 10.7, 2.4 Hz, H1), 5.75 (1H, dq, J = 10.7, 1.8 Hz, H2), 4.48 (1H, dq, J = 4.5, 2.2 Hz, H3), 3.92 (2H, m, H4/H6), 3.67 (1H, dd, J = 13.1, 3.2 Hz, H9), 3.63 (1H, dd, J = 11.8, 4.3 Hz, H10), 3.49 (1H, td, J = 12.0, 3.1 Hz, H10), 3.41 (1H, td, J = 9.9, 7.5 Hz, H5), 3.24 (1H, d, J = 9.7 Hz, H7), 2.89 (1H, dddd, J = 13.4, 12.0, 4.3, 1.6 Hz, H9), 2.81 (3H, s, H12). **^{13}C NMR (151 MHz, CD_3CN) δ:** 169.0 (1C, C8), 168.8 (1C, 11), 128.6 (1C, C2), 127.8 (1C, C1), 69.4 (1C, C3), 59.8 (1C, C10), 54.7 (1C, C6), 51.8 (1C, H4), 50.9 (1C, C7), 41.1 (1C, C9), 32.1 (1C, C5), 28.1 (1C,C12). **HRMS (APCI$^+$, deconvoluted):** [M] calcd. for $C_{12}H_{14}N_2O_3$, 234.1004; found, 234.1003.

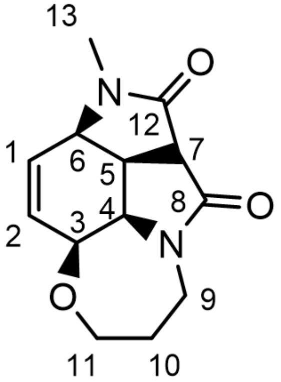

Compound 79:

Compound **76** (110 mg; 0.146 mmol) was taken in ACN (12 mL) followed by $AgNO_3$ (100 mg; 0.586 mmol) and stirred in darkness for 6 days. The reaction filtered through celite and dried in vacuo. The resulting crude mixture was then purified by preparative TLC (5% MeOH/acetone). The product (Rf = 0.3) was visualized as a burn spot with $KMnO_4$ and the corresponding silica band was scraped from the plate and loaded in a fritted disc. The product was eluted off with excess 5% MeOH/acetone and evaporated to an oil (13 mg, 0.052 mmol, 36%).

1H NMR (800 MHz, CD_3CN) δ: 6.11 (1H, dd, J = 10.2, 4.0 Hz, H2), 6.08 (1H, ddd, J = 10.2, 3.5, 1.5 Hz, H1), 4.35 (1H, ddt, J = 5.7, 3.7, 1.6 Hz, H3), 4.09 (1H, dd, J = 8.5, 4.7 Hz, H4), 3.98 (1H, ddt, J = 9.1, 3.1, 1.4 Hz, H6), 3.92 (1H, dt, J = 12.7, 4.8 Hz, H11), 3.67 (1H, ddd, J = 12.9, 9.4, 3.8 Hz, H11), 3.57 (1H, ddd, J = 13.9, 7.4, 3.3 Hz, H9), 3.29 (1H, m, H5), 3.19 (2H, m, H7/H9), 2.72 (3H, s, H13), 1.80 (1H, m, H10), 1.68 (1H, m, H10). **^{13}C NMR (201 MHz, CD_3CN) δ:** 170.3 (1C, C8), 168.9 (1C, C12), 129.2 (1C, C2), 128.1 (1C, C1), 74.6 (1C, C3), 70.0 (1C, C11), 59.2 (1C, C4), 54.6 (1C, C6), 51.0 (1C, C7), 42.5 (1C, C9), 33.0 (1C, C5), 29.0 (1C, C10), 28.1 (1C, C13). **IR (neat):** *v*(CO) 1700 cm^{-1}, *v*(CH) 2949 cm^{-1}.

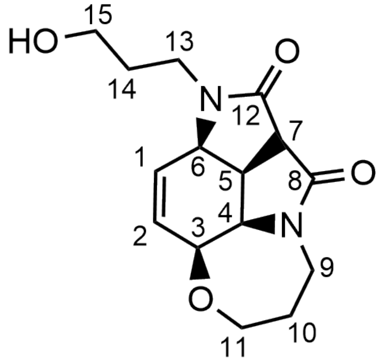

Compound 80:

Compound **77** (110 mg; 0.138 mmol) was taken in ACN (10 mL) followed by $AgNO_3$ (94 mg; 0.553 mmol) and stirred in darkness for 9 days. The reaction filtered through celite and dried in vacuo. The resulting crude mixture was then purified by preparative TLC (5% MeOH/acetone). The product (Rf = 0.3) was visualized as a burn spot with $KMnO_4$ and the corresponding silica band was scraped from the plate and loaded in a fritted disc. The product was eluted off with excess 5% MeOH/acetone and evaporated to an oil (10 mg, 0.034 mmol, 25%).

^{1}H NMR (800 MHz, CD3CN) δ 6.16 (1H, dd, J = 10.2, 4.4 Hz, H2), 6.13 (1H, ddd, J = 10.1, 4.0, 1.2 Hz, H1), 4.31 (1H, tt, J = 4.7, 1.3 Hz, H3), 4.14 (1H, m, H6), 4.07 (1H, m, H4), 3.93 (1H, m, H11), 3.63 (2H, dddd, J = 16.3, 13.8, 8.4, 3.4 Hz, H9), 3.44 (3H, m, H13/H15), 3.28 (2H, m, H5/H7), 3.16 (1H, m, H9), 3.12 (1H, m, H13), 1.82 (1H, dtdd, J = 14.9, 10.1, 4.9, 3.3 Hz, 10H), 1.64 (3H, m, H10/14). **^{13}C NMR (201 MHz, CD3CN) δ** 170.2 (1C, C8/12), 170.0 (1C, C8/12), 130.0 (1C, C2), 128.7 (1C, C1) , 74.6 (1C, C3), 71.0 (1C, C11), 59.4 (1C, C4), 59.2 (1C, C15), 53.0 (1C, C6), 51.0 (1C, C7), 42.5 (1C, C9), 38.5 (1C, C13), 32.9 (1C, C5), 30.9 (1C, C14), 29.1 (1C, C10). **HRMS (APCI$^+$, deconvoluted):** [M] calcd. for $C_{15}H_{20}N_2O_4$, 292.1423; found, 292.1428. **IR (neat):** *v*(CO) 1701 cm^{-1}, *v*(C-H) 2849 and 2917 cm^{-1}, *v*(OH) 3400 cm^{-1}.

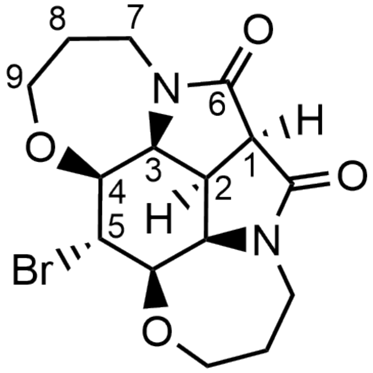

Compound 81:

Compound **80** (22 mg, 0.075 mmol) was taken in a test tube with 6 mL MeCN and cooled to -10°C. Solid Na_2CO_3 (160 mg, 1.50 mmol) was added to the tube followed by Br_2 (108 mg, 0.675 mmol). The reaction was then allowed to warm to 0°C over 30 min before being placed at room temperature. The reaction was then immediately quenched with saturated $Na_2S_2O_3$ dropwise until colorless and then diluted with 30 mL of DCM. The DCM was dried over Na_2SO_4 and evaporated in vacuo to a crude oil. The product was further purified by preparative TLC (4% MeOH/DCM) and the corresponding silica band (Rf = 0.35) was collected and loaded on a fritted disc. The product was eluted off with excess 5% MeOH/DCM and evaporated to an oil (7 mg, 0.019 mmol, 25%).

^{1}H NMR (800 MHz, CD3CN) δ 4.25 (2H, dd, J = 8.9, 5.2 Hz, H3), 4.23 (1H, d, J = 8.1 Hz, H5), 4.18 (2H, dd, J = 8.1, 5.2 Hz, H4), 3.95 (2H, ddd, J = 12.9, 6.3, 3.7 Hz, H9a), 3.64 (2H, ddd, J = 12.6, 8.5, 2.9 Hz, H9b), 3.58 (2H, ddd, J = 14.0, 7.9, 3.5 Hz, H7a), 3.39 (1H, m, H2), 3.21 (2H, ddd, J = 14.1, 8.6, 3.0 Hz, H7b), 3.18 (1H, d, J = 10.0 Hz, H1), 1.83 (2H, dtt, J = 15.4, 8.4, 3.6 Hz, H8a), 1.72 (2H, m, H8b). **^{13}C NMR (201 MHz, CD3CN) δ** 169.7 (1C, C6), 82.2 (2C, C4), 71.0 (2C, C9), 60.3 (2C, C3), 51.9 (1C, C5), 50.9 (1C, C1), 44.2 (2C, C7), 33.8 (1C, C2), 28.6 (2C, C9). **HRMS (APCI$^+$, deconvoluted):** [M] calcd. for $C_{15}H_{19}BrN_2O_4$, 370.0528 and 372.0508; found, 370.0532 and 372.0512. **IR (neat):** *v*(C-Br) 698 cm^{-1}, *v*(CO) 1697 cm^{-1}, *v*(C-H) 2854 and 2923 cm^{-1}.

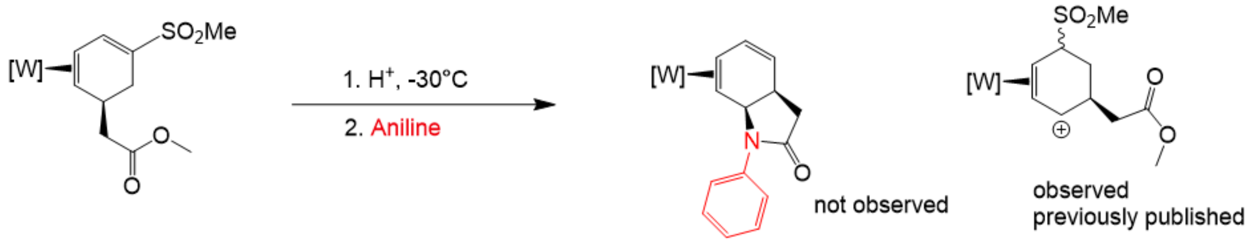

Compound 82:

Compound **2** (100 mg, 0.136 mmol) was placed in a test tube with ACN (2 mL), and chilled to -30 °C. After 10 min, a 1 M HOTf/ACN (0.273 mL, 0.273 mmol) solution was added to the test tube and the solution was allowed to stir at -30 °C for 30 min. In a separate test tube, aniline (0.123 mL, 1.360 mmol) with ACN (2 mL) was cooled at -30 °C for 20 min. After the time elapsed, the former solution was added to the latter, dropwise. The reaction stirred at -30 °C for 48 h and room temperature for 5 h. The organic layer was evaporated in vacuo and concentrated to a thin yellow film. The resulting film was dissolved in minimal DCM and pipetted into 25 mL of stirring hexane. An off-white solid precipitated out and was collected on a 15 mL fine porosity fitted disk, washed with hexane (2 × 10 mL) and desiccated overnight to yield compound **82** (97 mg, 0.116 mmol, 85%).

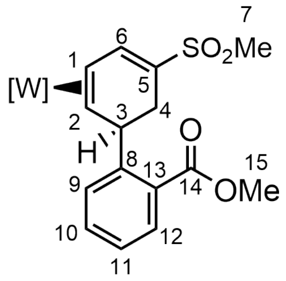

Compound 83:

Methyl 2-Iodobenzoate (0.424 mL, 2.88 mmol) was transferred to a small oven-dried test tube to which 1 mL of dry THF was added. The solution was cooled to -30° C for 5 minutes. To this solution, 3M isopropyl magnesium bromide in 2-methyl THF (0.910 mL, 2.73 mmol) was added and the resulting solution was stirred for an hour. A yellow\brown precipitate slowly formed. While this solution stirred, compound **1** (1.00 g, 1.517 mmol) was taken into a large test tube and placed in an ice bath to cool for 5 minutes. To this test tube, 1M HOTf in acetonitrile (1.82 mL, 1.821 mmol) was added. The solution was stirred until a bronze-colored precipitate formed. The test tube was then cooled to -30° C for 20 minutes. While this test tube cooled, a third test tube containing CuCN (272 mg, 3.03 mmol) dissolved in minimal dry THF was prepared and then cooled to -30° C for 5 minutes. This solution was then added to the original test tube containing 3M isopropyl magnesium bromide and methyl 2-Iodobenzoate and the resulting solution was stirred for 15 minutes at -30°C. Finally, this solution was added to the large test tube containing the bronze-colored precipitate. The mixture was then left to stir overnight at -30° C. The next day, the reaction was taken out of the cold bath and ~10 mL of saturated Na_2CO_3 solution was added followed by ~10 mL of dichloromethane. The DCM layer was extracted and dried using Na_2SO_4 and the extraction\drying process was repeated twice more. The extracted organic layers were combined and evaporated until a dark orange film was formed. The film was then redissolved in the minimum amount of DCM possible and transferred onto a silica column pre-wet with hexanes. 100 mL of hexanes, 50 mL of diethyl ether, and 200 mL of ethyl acetate was pulled through the column. The ethyl acetate fraction was collected and evaporated until a light-yellow film formed. The resulting film was taken into the minimum amount of DCM and precipitated into ~125 mL of

stirring hexanes. The precipitate was then collected on a 15 mL fine-porosity fritted disc and desiccated overnight yielding compound **83** (605 mg, 0.761 mmol, 50%).

^{1}H NMR (800 MHz, CD_3CN) δ: 8.05 (1H, d, Tp3B), 8.02 (1H, d, Tp3A), 7.89 (1H, d, H9) 7.87 (1H, d Tp5B), 7.85 (1H, d, Tp5C), 7.79 (1H, d Tp5A), 7.78 (1H, dd, H6), 7.55 (1H, d, H12) 7.47 (1H, d, Tp3C), 7.43 (1H, t, H10), 7.19 (1H, t, H11), 6.38 (1H, t, Tp4B), 6.36 (1H, t, Tp4A), 6.28 (1H, t, Tp4C), 4.95 (1H, d, H3), 3.70 (3H, s, H15), 3.20 (1H, m, H1), 3.12 (1H, dd, H4a), 2.83 (3H, s, H7), 2.44 (1H, d, H4b), 1.29 (9H, d, PMe_3)1.26 (1H, d, H2). **^{13}C-NMR (201 MHz, CD_3CN) δ:** 170.1 (1C, C14), 151.6 (1C, C5), 145.8 (1C, C6), 144.5 (1C, Tp3A), 142.3 (1C, Tp3B), 141.9 (1C, Tp3C), 138.0 (1C, Tp5C), 137.7 (1C, Tp5B), 137.5 (1C, Tp5A), 132.0 (1C, C10), 131.3 (1C, C8), 129.9 (1C, C9), 129.5 (1C, C12), 127.0 (1C, C13), 126.4 (1C, C11), 107.6 (1C, Tp4B), 107.4 (1C, Tp4A), 107.1 (1C, Tp4C), 61.9 (1C, C2), 52.6 (1C, C15), 48.5 (1C, d, C1), 45.0 (1C, C7), 36.9 (1C, C3), 29.4 (1C, C4), 13.8 (3C, d, PMe_3). **IR (neat):** *v*(NO) 1548 cm^{-1}, *v*(CO) 1717 cm^{-1}, *v*(BH) 2484 cm^{-1}

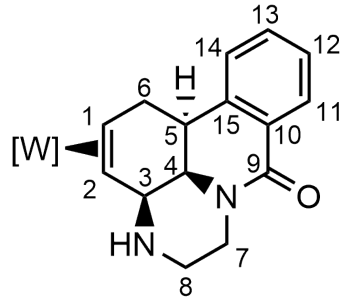

Compound 84:

Compound **83** (160 mg, 0.201 mmol) was taken into a test tube along with ~5 mL of acetonitrile. The solution was chilled to -30°C for 5 minutes. 1 M HOTf (0.402 mL, 0.402 mmol) was added to the solution. After 15 minutes, ethylene diamine (0.134 mL, 2.012 mmol) was added to the solution. This mixture was stirred at -30°C for 2 days, and then 4 hours at room temperature. Then, ~5 mL of saturated Na_2CO_3 solution was added followed by ~5 mL of dichloromethane. The DCM layer was extracted and dried using Na_2SO_4 and the extraction\drying process was repeated twice more. The extracted organic layers were combined and evaporated until a film was formed. The film was then redissolved in the minimum amount of DCM possible and precipitated into ~75 mL of stirring pentanes. The precipitate was then collected on a 15 mL fine-porosity fritted disc and desiccated overnight yielding compound **85** (126 mg, 170 mmol, 84%).

^{1}H NMR (800 MHz, $CDCl_3$) δ: 8.32 (1H, d, Tp3A), 8.12 (1H, d, H11), 8.11 (1H, d, Tp3B), 7.74 (1H, d, Tp5B), 7.71 (1H, d, Tp5C), 7.67 (1H, d Tp5A), 7.42 (1H, t, H13), 7.33 (1H, t, H12), 7.32 (1H, d, Tp3C), 7.28 (1H, d, H14), 6.35 (1H, t, Tp4B), 6.27 (1H, t, Tp4A), 6.20 (1H, t, Tp4C), 4.90 (1H, d, H3), 4.38 (1H, d, H8a), 3.88 (1H, t, H4), 3.70 (1H, td, H6a), 3.47 (1H, m, H5), 3.37 (1H, td, H7a), 2.98 (1H, td, H8b), 2.92 (1H, m, H1), 2.78 (1H, dd, H7b), 2.61 (1H, dd, H6b), 1.21 (1H, d, H2), 1.19 (9H, d, PMe_3) **^{13}C-NMR (201 MHz, $CDCl_3$) δ:** 167.5 (1C, C9), 145.5 (1C, C10), 143.3

(1C, Tp3B), 141.1 (1C, Tp3A), 136.7 (1C, Tp5C), 136.2 (1C, Tp5A), 136.0 (1C, Tp5B), 131.9 (1C, C13), 128.8 (1C, C11), 128.2 (1C, C15), 126.7 (1C, C12), 126.5 (1C, C14), 106.8 (1C, Tp4B), 106.2 (1C, Tp4A), 106.1 (1C, Tp4C), 55.7 (1C, C3), 55.6 (1C, d, C1), 55.3 (1C, C4), 53.0 (1C, C2), 42.2 (1C, C8), 38.0 (1C, C7), 36.4 (1C, C6), 36.2 (1C, C5), 13.4 (3C, d, PMe_3).

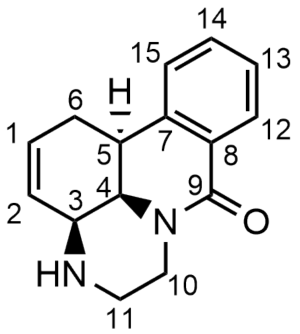

Compound 85:

Compound **84** (186 mg, 0.251 mmol) was taken in a test tube with ~5 mL MeCN. To this, DDQ (125 mg, 0.552 mmol) was added and the reaction stirred for 5 min. The crude mixture was then added to a separatory funnel with 50 mL of 0.5 M HCl and extracted with DCM (3x15 mL). NaOH (20% wt) was then added to the aqueous layer until the pH was >10, and the aqueous solution was re-extracted with DCM (3x15 mL). The combined organic layers were dried over Na_2SO_4 and evaporated to a clear oil in vacuo yielding compound **89** (13 mg, 0.054 mmol, 22%).

^{1}H NMR (800 MHz, D_2O) δ: 7.94 (1H, d, J = 7.9, H12), 7.58 (1H, t, J = 7.4 Hz, H14), 7.44 (1H, t, J = 7.6 H13), 7.35 (1H, d, J = 7.5 Hz, H15), 6.08 (1H, dd, J = 10.7, 5.0 Hz, H1), 5.59 (1H, dq, J = 10.2, 1.6 Hz, H2), 4.12 (1H, m, H10a), 3.89 (2H, s, H3/H4), 3.17 (1H, dt, J = 10.7, 4.2 Hz, H5), 2.92 (3H, m, H10b/H11a/H11b), 2.18 (1H, dt, J = 18.5, 5.8 Hz, H6a), 1.81 (1H, ddd, J = 18.6, 11.1, 2.7 Hz, H6b). **^{13}C NMR (201 MHz, D_2O) δ:** 168.7 (1C, C9), 143.3 (1C, C7), 133.3 (1C, C14), 131.4 (1C, C1), 128.0 (1C, C12), 127.4 (1C, C13), 127.0 (1C, C2), 126.5 (1C, C15), 126.2 (1C, C8), 52.6 (1C, C4), 49.6 (1C, C3), 41.0 (1C, C10), 37.2 (1C, C11), 36.4 (1C, C5), 28.8 (1C, C6). *Referenced to $OPMe_3$ 15.8 (d, J = 70.1 Hz).*

DFT Analysis:

Ground-state structures were optimized at the M06 level of theory using the 6-31G** [LANL2DZ for W] basis set in Gaussian 16.[56] Previous literature demonstrates that this functional and basis set choice accurately corroborates experimental results.[57] Solvent effects of acetonitrile were modeled using SMD. Gaussian's default criteria were used for optimization, vibrational frequency analysis verified that all structures contain no imaginary frequencies, and thermal free energy corrections were applied.

Supplementary Fig. 187: Summary of DFT calculations.

Structure	Electronic Energy (Hartree)	Relative Free Energy (kcal/mol)
ID	-2221.027764	0.0
IP	-2221.020150	+4.8
IIID	-1839.943947	0.0
IIIP	-1839.943133	+0.5
VII	-1934.062356	0.0
VI	-1934.061306	+0.7
VIII	-1934.043481	+11.9
V	-1934.061200	+0.7
IX	-1934.055962	+4.0
32	-2221.859302	0.0
30	-2221.855662	+2.3
32'	-2221.858350	0.0
30'	-2221.857058	+0.8
31	-2201.980776	0.0
29	-2201.976988	+2.4
18	-1953.940675	0.0
27	-1953.931793	+5.6
22	-2012.565689	+1.9
28	-2012.568747	0.0

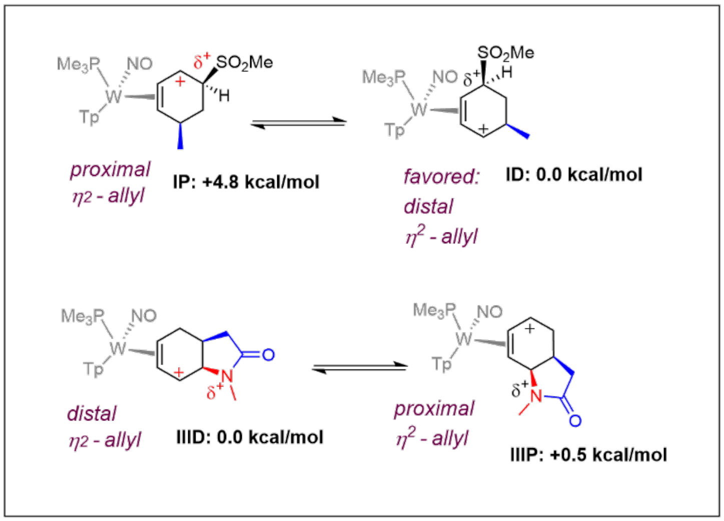

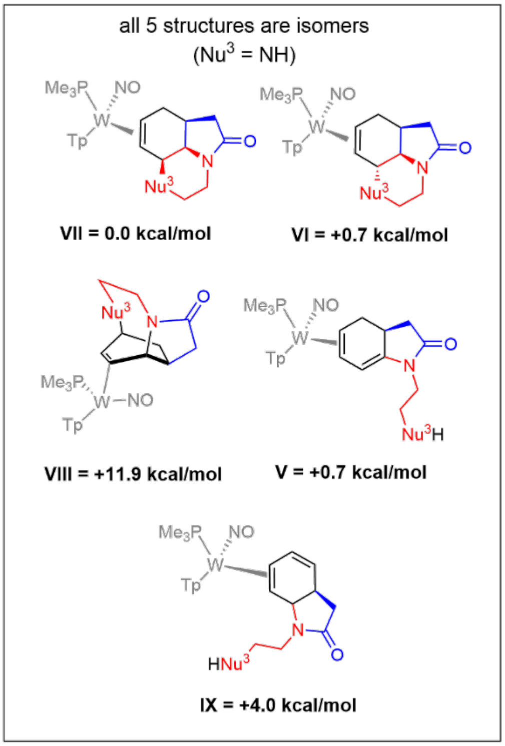

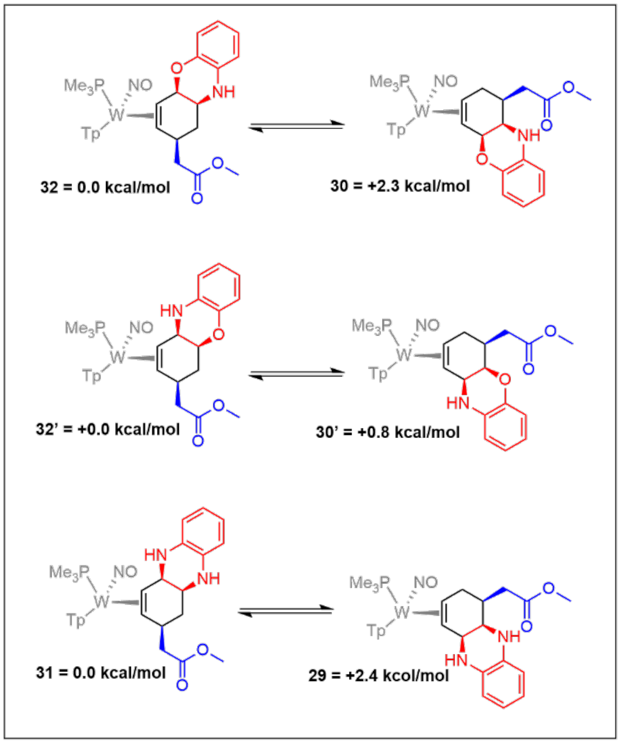

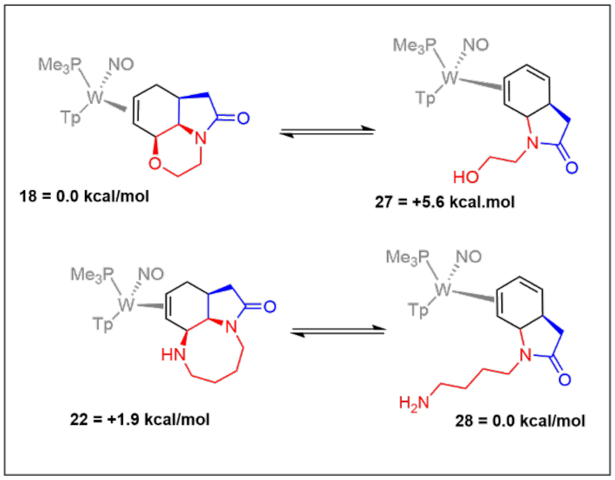

Crystallographic Data:

A single crystal of each molecule listed in tables S1-S6 was coated with Paratone oil and mounted on a MiTeGen MicroLoop. The X-ray intensity data were measured on a Bruker D8 Venture dual wavelength Mo/Cu Kappa four-circle diffractometer equipped with a PHOTON III detector and an Oxford Cryostream 800 or 800Plus low-temperature device. An Incoatec IμS 3.0 microfocus sealed X-ray tube (Cu K_α, λ=1.54178 Å) and either a HELIOS MX or HELIOS EF double bounce multilayer mirror monochromator were used for **16, 33, 39, 40, 79**. An Incoatec IμS 3.0 micro-focus sealed X-ray tube (Mo K_α, λ = 0.71073 Å) and a HELIOS double bounce multilayer mirror monochromator for were used for all others. All frames were integrated with the Bruker SAINT software package[4] using a narrow-frame algorithm. Data were corrected for absorption effects using the Multi-Scan method (SADABS[5] or TWINABS (**6, 7, 33**).[6] Each structure was solved using the Bruker SHELXT Software Package[7] within either APEX4/APEX5[4] or OLEX2.[8] For the structure of **85**, all atoms including hydrogen were refined anisotropically using olex2.refine and NoSpherA2,[9] an implementation of NOn-SPHERical Atom-form-factors in Olex2 using the ORCA 5.0 quantum mechanical package[10, 11] with the def2-TZVP basis set and PBE method.

For all other structures, non-hydrogen atoms were refined anisotropically using SHELXL.[12] Hydrogen atoms were placed in geometrically calculated positions with U_{iso} = 1.2U_{equiv} of the parent atom (U_{iso} = 1.5U_{equiv} for methyl), with the following exceptions that were located in the electron density map and refined isotropically: the B-H hydrogen atoms for all W complexes except **7, 15·HOTf, 16** and **33**; the hydrogen atoms on the carbons directly bound to W in **2, 6, 8, 8·2CH$_2$Cl$_2$, 10, 12-14, 18, 19, 27, 30, 34, 35, 38, 39, 41, 75, 76, 77, 83,** and **84**; the N-H and/or O-H hydrogen atoms in **6**, **8-10, 12, 14, 19, 21, 26, 27, 30, 34, 41,** and **77**. Most CIF files were prepared for publication using FinalCif.[13] Additional refinement details are given below.

The structure of **3** was refined as an inversion twin, with the BASF parameter of the twin domains refining to 0.47975. The structures of **6, 7,** and **33** were each refined as 2-domain twins on HKLF 5 data. The BASF parameter for the twin domains refined to 0.4908 for **6**, 0.1321 for **7**, and 0.51261 for **33**. The relative occupancy of each set of disordered positions in **3, 8, 14, 16, 26, 27, 41, 75, 77,** and **83** was freely refined. Constraints were used on the anisotropic displacement parameters of most of the disordered atoms and restraints were used on most of the disordered bonds. Additionally, a global RIGU restraint was used in **16** due to the weak diffraction data.

In the structures of **9, 16**, **21, 34,** and **39,** there was severely disordered co-crystallized solvent that could not be adequately modeled with or without constraints. These solvents were accounted for using Platon SQUEEZE.[14] A void space of 327 A^3 containing 15 electrons was found in **9**. This corresponds to a partially occupied pentane or CH_2Cl_2 molecule. A void space of 2228 A^3 containing 1003 electrons was found in **16**. This corresponds to a mixture of CH_2Cl_2 and Et_2O. A void space of 282 A^3 containing 65 electrons was found in **21**. This corresponds to 3-4 molecules of methanol. A void space of 948 Å^3 containing 211 electrons was found in **34**. This corresponds to a mixture of acetone and pentanes. A void space of 843 Å^3 containing 167 electrons was found in **39**. This corresponds to a mixture of acetone and pentane.

Supplementary Table 1: Crystal data for **2, 3,** and **5-8.**

	**2**	**3**	**5**	**6**	**7**	**8**	**8·2CH$_2$Cl$_2$**
CCDC number	2432199	2432200	2432201	2432202	2432203	2432204	2432205
Formula	$C_{25}H_{39}BN_7O_6PSW$	$C_{27}H_{43}BN_7O_6PSW$	$C_{22}H_{33}BN_9O_2PW$	$C_{24}H_{37}BN_9O_2PW$	$C_{24}H_{35}BN_9O_4PW$	$C_{24}H_{37}BN_9O_2PW$	$C_{26}H_{41}BCl_4N_9O_2PW$
FW (g/mol)	791.32	819.37	681.20	709.25	739.24	709.25	879.11
Temp (K)	100(2)	100(2)	100(2)	100(2)	100(2)	300(2)	100(2)
λ (Å)	0.71073	0.71073	0.71073	0.71073	0.71073	0.71073	0.71073
Size (mm)	0.051 x 0.060 x 0.147	0.04 × 0.044 × 0.073	0.01 × 0.02 × 0.04	0.04 × 0.059 × 0.169	0.038 × 0.045 × 0.229	0.098 × 0.081 × 0.032	0.023 x 0.037 x 0.074
Crystal habit	colourless block	colourless plate	colourless plate	colourless needle	colourless needle	colourless plate	colourless needle
Crystal system	monoclinic	triclinic	monoclinic	monoclinic	monoclinic	triclinic	triclinic
Space group	P 2_1/n	P 1	P 2_1/n	P 2_1/n	P 2_1/c	P -1	P -1
a (Å)	14.2707(7)	11.7294(4)	10.2784(5)	10.3764(3)	10.0172(8)	9.9748(3)	9.8458(4)

b(Å)	15.3139(7)	12.2675(5)	17.4183(10)	22.9111(7)	18.882(2)	12.9649(5)	13.1319(7)
c (Å)	15.7164(9)	12.9211(6)	14.9222(8)	11.7392(3)	15.3182(15)	13.1464(5)	14.4525(8)
α (°)	90	110.907(2)	90	90	90	60.4930(10)	109.749(2)
β (°)	113.803(2)	103.760(2)	106.847(2)	95.6140(10)	108.376(3)	84.9820(10)	100.603(2)
γ (°)	90	94.8740(10)	90	90	90	74.5230(10)	94.134(2)
Volume ($Å^3$)	3142.5(3)	1657.15(12)	2556.9(2)	2777.43(14)	2749.6(5)	1424.00(9)	1710.38(15)
Z	4	2	4	4	4	2	2
Density (g/cm^3)	1.673	1.642	1.770	1.696	1.786	1.654	1.707
μ (mm^{-1})	3.843	3.647	4.620	4.257	4.309	4.151	3.776
F(000)	1584	824	1352	1416	1472	708	876
θ range (°)	1.94 to 27.10	1.809 to 28.325	2.147 to 28.289	2.163 to 27.489	2.142 to 28.472	2.121 to 26.375	1.82 to 28.36
Index ranges	-18 ≤ h ≤ 18<br>-19 ≤ k ≤ 19<br>-20 ≤ l ≤ 20	−15 ≤ h ≤ 15<br>−16 ≤ k ≤ 16<br>−17 ≤ l ≤ 17	−13 ≤ h ≤ 13<br>−23 ≤ k ≤ 23<br>−19 ≤ l ≤ 18	−13 ≤ h ≤ 13<br>0 ≤ k ≤ 29<br>0 ≤ l ≤ 15	−13 ≤ h ≤ 12<br>0 ≤ k ≤ 25<br>0 ≤ l ≤ 20	−12 ≤ h ≤ 12<br>−16 ≤ k ≤ 16<br>−16 ≤ l ≤ 16	-11 ≤ h ≤ 13<br>-17 ≤ k ≤ 17<br>-19 ≤ l ≤ 19
Reflns collected	40643	119521	36954	10868	14095	56583	71345
Independent reflns	6926 [R_{int} = 0.0845]	16484 [R_{int} = 0.0711]	6337 [R_{int} = 0.0905]	6407 [R_{int} = 0.0701]	6975 [R_{int} = 0.0774]	5833 [R_{int} = 0.0903]	8515 [R_{int} = 0.0849]
Data / restraints /parameters	6926 / 0 / 397	16484 / 10 / 816	6337 / 1 / 335	6407 / 0 / 364	6975 / 0 / 369	5833/97/426	8515 / 0 / 417
GOF on F^2	1.016	1.026	1.000	1.042	1.035	1.017	1.047
R_1 (I>2σ(I))	0.0340	0.0284	0.0345	0.0313	0.0448	0.0283	0.0332
wR_2 (all data)	0.0721	0.0598	0.0706	0.0679	0.1055	0.0622	0.0763

Supplementary Table 2. Crystal data for **9, 10,** and **12-16.**

	**9**	**10**	**12**	**13**	**14**	**15·HOTf**	**16**
CCDC number	2432217	2432218	2432219	2432220	2432221	2432222	2432223
Formula	$C_{26}H_{41}BN_9O_2$ PW	$C_{23}H_{35}BN_9O_2$ PW	$C_{23}H_{35}BN_9O_2$ PW	$C_{29}H_{39}BN_9O_2$ PW	$C_{24}H_{37}BN_9O_3$ PW	$C_{27}H_{40}BF_3N_9O_5$ PSW	$C_{24}H_{38}BN_{10}O_2$ PW
FW (g/mol)	737.31	695.23	695.23	771.32	725.25	885.37	724.27
Temp (K)	100(2)	100(2)	100(2)	100(2)	100(2)	100(2)	100(2)
λ (Å)	0.71073	0.71073	0.71073	0.71073	0.71073	0.71073	1.54178
Size (mm)	0.026 × 0.042 × 0.107	0.052 × 0.096 × 0.115	0.032 × 0.065 × 0.364	0.048 x 0.068 x 0.094	0.029 x 0.092 x 0.145	0.046 × 0.079 × 0.109	0.007 x 0.073 x 0.108
Crystal habit	colourless needle	colourless needle	colourless needle	colourless plate	colourless plate	colourless plate	colourless needle
Crystal system	monoclinic	monoclinic	monoclinic	monoclinic	monoclinic	monoclinic	monoclinic
Space group	P 2_1/c	P 2_1	P 2_1	P 2_1/n	P 2_1/c	P n	I 2
a (Å)	12.3484(9)	8.5048(4)	10.5222(8)	9.8388	14.5623	12.2532(6)	22.7017(8)
b(Å)	25.111(2)	28.6885(10)	13.6741(9)	13.1493(5)	12.1588(4)	7.4647(4)	14.4788(4)
c (Å)	20.0032(18)	11.0890(3)	19.3241(15)	23.9472(11)	15.6697(4)	17.8039(9)	22.8425(14)

α (°)	90	90	90	90	90	90	90
β (°)	91.104(2)	94.1170(10)	103.811(2)	94.483(2)	102.0340(10)	100.779(2)	93.093(2)
γ (°)	90	90	90	90	90	90	90
Volume (Å^3)	6201.5(9)	2698.62(17)	2700.0(3)	3088.7(2)	2713.51(14)	1599.73(14)	7497.2(6)
Z	8	4	4	4	4	2	8
Density (g/cm^3)	1.579	1.711	1.710	1.659	1.775	1.838	1.283
μ (mm^{-1})	3.816	4.379	4.377	3.835	4.362	3.798	6.375
F(000)	2960	1384	1384	1544	1448	884	2896
θ range (°)	2.09 to 25.05	1.973 to 28.336	1.843 to 28.293	2.18 to 29.60	2.41 to 28.31	2.23 to 26.38	2.67 to 68.45
Index ranges	−14 ≤ h ≤ 13<br>−29 ≤ k ≤ 29<br>−23 ≤ l ≤ 23	−11 ≤ h ≤ 11<br>−38 ≤ k ≤ 38<br>−14 ≤ l ≤ 14	−14 ≤ h ≤ 14<br>−18 ≤ k ≤ 18<br>−25 ≤ l ≤ 25	-13 ≤ h ≤ 13<br>-18 ≤ k ≤ 17<br>-33 ≤ l ≤ 32	-19 ≤ h ≤ 19<br>-16 ≤ k ≤ 14<br>-17 ≤ l ≤ 20	−15 ≤ h ≤ 15<br>−9 ≤ k ≤ 9<br>−22 ≤ l ≤ 22	-27 ≤ h ≤ 27<br>-17 ≤ k ≤ 17<br>-27 ≤ l ≤ 26
Reflns collected	74891	86623	82479	58132	49232	37253	57045
Independent reflns	10958 [R_{int} = 0.1215]	13427 [R_{int} = 0.0691]	13362 [R_{int} = 0.0541]	8652 [R_{int} = 0.0888]	6751 [R_{int} = 0.0832]	6536 [R_{int} = 0.0374]	13688 [R_{int} = 0.1117]
Data / restraints /parameters	10958 / 0 / 751	13427 / 1 / 705	13362 / 1 / 705	8652 / 0 / 400	6751 / 161 / 423	6536 / 2 / 436	13688 / 755 / 716
GOF on F^2	1.021	1.025	1.031	1.010	1.036	1.080	1.035
R_1 (I>2σ(I))	0.0444	0.0319	0.0273	0.0370	0.0344	0.0313	0.0535
wR_2 (all data)	0.1075	0.0702	0.0616	0.0772	0.0798	0.0773	0.1486

Supplementary Table 3: Crystal data for **18-21, 26,** and **27.**

	**18**	**19**	**20**	**21**	**26**	**27**
CCDC number	2432224	2432225	2432226	2432227	2432228	2432229
Formula	$C_{24}H_{35}BN_9O_3PW$	$C_{25}H_{38}BN_{10}O_2PW$	$C_{25}H_{39}BCl_2N_9O_2PW$	$C_{25}H_{37}BN_9O_4PW$	$C_{26}H_{40}BN_8O_4PW$	$C_{22}H_{32}BN_8O_3PW$
FW (g/mol)	723.24	736.28	794.18	753.26	754.29	682.18
Temp (K)	100(2)	100(2)	100(2)	100(2)	100(2)	100(2)
λ (Å)	0.71073	0.71073	0.71073	0.71073	0.71073	0.71073
Size (mm)	0.058 x 0.091 x 0.117	0.043 × 0.061 × 0.132	0.04 × 0.091 × 0.166	0.07 4× 0.103 × 0.115	0.055 x 0.082 x 0.179	0.044 x 0.087 x 0.134
Crystal habit	colourless plate	colourless rod	colourless needle	colourless plate	colorless block	colorless plate
Crystal system	monoclinic	triclinic	triclinic	monoclinic	monoclinic	monoclinic
Space group	P 2_1/c	P -1	P -1	P 2_1/c	P 2_1/c	P 2_1/c
a (Å)	11.6632(6)	9.7805(3)	10.2091(6)	13.2192(9)	21.1853(9)	13.2055(5)
b(Å)	15.0807(6)	12.4416(4)	12.4991(7)	12.1097(7)	15.0978(6)	17.2321(5)
c (Å)	15.8438(7)	12.9471(5)	12.5035(6)	19.8806(13)	20.4456(8)	23.6985(8)
α (°)	90	96.8520(10)	106.427(2)	90	90	90
β (°)	95.127(2)	106.2720(10)	101.778(2)	106.418(2)	111.7050(10)	100.4770(10)
γ (°)	90	104.6560(10)	94.072(2)	90	90	90

Volume (Å^3)	2775.6(2)	1432.14(8)	1484.01(14)	3052.7(3)	6075.9(4)	5302.9(3)
Z	4	2	2	4	8	8
Density (g/cm^3)	1.731	1.707	1.777	1.639	1.649	1.709
μ (mm^{-1})	4.264	4.132	4.168	3.883	3.901	4.457
F(000)	1440	736	792	1504	3024	2704
θ range (°)	1.87 to 27.49	2.15 to 28.29	2.04 to 26.38	1.99 to 25.37	2.01 to 27.56	1.96 to 28.28
Index ranges	-14 ≤ h ≤ 15<br>-19 ≤ k ≤ 18<br>-20 ≤ l ≤ 20	−10 ≤ h ≤ 13<br>−16 ≤ k ≤ 16<br>−17 ≤ l ≤ 17	−12 ≤ h ≤ 12<br>−15 ≤ k ≤ 15<br>−14 ≤ l ≤ 15	−15 ≤ h ≤ 15<br>−13 ≤ k ≤ 14<br>−23 ≤ l ≤ 23	-27 ≤ h ≤ 27<br>-19 ≤ k ≤ 19<br>-25 ≤ l ≤ 26	-17 ≤ h ≤ 17<br>-22 ≤ k ≤ 18<br>-31 ≤ l ≤ 31
Reflns collected	37138	43181	44707	37010	89355	114708
Independent reflns	6358 [R_{int} = 0.0967]	7101 [R_{int} = 0.0559]	6011 [R_{int} = 0.0416]	5585 [R_{int} = 0.1052]	13963 [R_{int} = 0.0704]	13137 [R_{int} = 0.0625]
Data / restraints /parameters	6358 / 0 / 365	7101 / 0 / 379	6011 / 0 / 378	5585 / 0 / 382	13963 / 114 / 805	13137 / 3 / 699
GOF on F^2	1.033	1.075	1.105	1.019	1.025	1.026
R_1 (I>2σ(I))	0.0395	0.0251	0.0336	0.0441	0.0346	0.0291
wR_2 (all data)	0.0849	0.0582	0.0998	0.1038	0.0742	0.0681

Supplementary Table 4: Crystal data for **30, 33-35, 38** and **39.**

	**30**	**33**	**34**	**35**	**38**	**39**
CCDC number	2432206	2432207	2432208	2432209	2432210	2432211
Formula	$C_{29}H_{39}BN_9O_4PW$	$C_{27}H_{36}BN_8O_3PSW$	$C_{31}H_{39}BN_9O_3PW$	$C_{23}H_{35}BN_7O_3PS_2W$	$C_{25}H_{39}BN_7O_3PS_2W$	$C_{29}H_{39}BN_7O_3PS_2W$
FW (g/mol)	803.32	778.33	811.34	747.33	775.38	823.42
Temp (K)	100(2)	100(2)	100(2)	100(2)	100(2)	100(2)
λ (Å)	0.71073	1.54178	0.71073	0.71073	1.54178	1.54178
Size (mm)	0.032 × 0.078 × 0.086	0.032 x 0.033 x 0.136	0.067 × 0.07 × 0.134	0.091 × 0.143 × 0.293	0.046 × 0.061 × 0.079	0.035 × 0.053 × 0.067
Crystal habit	colourless block	colorless needle	colourless needle	colourless block	colourless plate	colourless plate
Crystal system	triclinic	triclinic	monoclinic	triclinic	triclinic	orthorhombic
Space group	P 1	P -1	P 2_1/c	P -1	P -1	P bca
a (Å)	8.3069(3)	7.6301(4)	17.0566(9)	10.9448(5)	8.5618(2)	16.6203(5)
b(Å)	8.9205(3)	13.8917(6)	17.6280(11)	11.7132(6)	11.0656(4)	13.9359(4)
c (Å)	12.2685(4)	29.8378(13)	14.1011(7)	12.6725(6)	17.2112(5)	30.5898(10)
α (°)	109.8177(12)	88.409(2)	90	113.7540(10)	89.714(2)	90
β (°)	97.1550(12)	89.470(2)	111.951(2)	101.3110(10)	76.786(2)	90
γ (°)	105.0904(12)	88.564(2)	90	94.751(2)	74.931(2)	90
Volume (Å^3)	802.80(5)	3160.3(3)	3932.5(4)	1434.19(12)	1530.28(8)	7085.2(4)
Z	1	4	4	2	2	8
Density (g/cm^3)	1.662	1.636	1.370	1.731	1.683	1.544
μ (mm^{-1})	3.697	8.219	3.018	4.267	9.091	7.894
F(000)	402	1552	1624	744	776	3296
θ range (°)	2.50 to 28.29	2.96 to 68.38	1.94 to 28.29	1.81 to 29.60	2.64 to 68.54	2.89 to 70.94

Index ranges	$-11 \le h \le 11$<br>$-11 \le k \le 11$<br>$-16 \le l \le 16$	$-9 \le h \le 9$<br>$-16 \le k \le 16$<br>$0 \le l \le 35$	$-22 \le h \le 21$<br>$-23 \le k \le 23$<br>$-18 \le l \le 17$	$-15 \le h \le 15$<br>$-16 \le k \le 16$<br>$-17 \le l \le 17$	$-10 \le h \le 9$<br>$-13 \le k \le 13$<br>$-20 \le l \le 20$	$-20 \le h \le 19$<br>$-17 \le k \le 16$<br>$-36 \le l \le 37$
Reflns collected	49695	121269	60914	47593	31042	71009
Independent reflns	7958 [R_{int} = 0.0486]	11577 [R_{int} = 0.1035]	9741 [R_{int} = 0.0553]	8034 [R_{int} = 0.0312]	5623 [R_{int} = 0.1001]	6778 [R_{int} = 0.1019]
Data / restraints /parameters	7958 / 3 / 415	11577 / 0 / 766	9741 / 0 / 439	8034 / 0 / 359	5623 / 0 / 379	6778 / 0 / 415
GOF on F^2	1.063	1.064	1.020	1.067	1.044	1.047
R_1 (I>2σ(I))	0.0218	0.0875	0.0236	0.0155	0.0458	0.0379
wR_2 (all data)	0.0485	0.2439	0.0524	0.0343	0.1109	0.0910

Supplementary Table 5: Crystal data for **40, 41, 63, 75** and **76.**

	**40**	**41**	**63**	**75**	**76**
CCDC number	2432212	2432213	2432214	2432215	2432216
Formula	$C_{29}H_{39}BN_7O_4PSW$	$C_{27}H_{45}BN_9O_4PW$	$C_{17}H_{20}O_2S_2$	$C_{51}H_{72}B_2N_{18}O_9P_2W_2$	$C_{27}H_{38}BN_{10}O_4PW$
FW (g/mol)	807.36	785.35	320.45	1532.52	792.30
Temp (K)	100(2)	100(2)	100(2)	100(2)	100(2)
λ (Å)	1.54178	0.71073	0.71073	0.71073	0.71073
Size (mm)	0.044 × 0.044 × 0.081	0.044 × 0.079 × 0.352	0.157 × 0.201 × 0.371	0.035 × 0.065 × 0.079	0.044 × 0.061 × 0.121
Crystal habit	colourless prism	colourless needle	colourless plate	colourless plate	colourless plate
Crystal system	monoclinic	orthorhombic	monoclinic	monoclinic	monoclinic
Space group	$P\ 2_1/n$	$P\ 2_12_12_1$	$P\ 2_1/c$	$P\ 2_1/c$	$P\ 2_1/c$
a (Å)	18.5399(9)	7.8278(2)	13.5314(7)	12.6385(4)	12.7192(5)
b(Å)	7.9462(3)	17.0738(6)	10.2355(4)	14.9862(4)	14.9872(6)
c (Å)	22.9289(11)	23.1515(8)	12.7533(5)	15.5750(4)	16.0415(6)
α (°)	90	90	90	90	90
β (°)	107.818(3)	90	117.304(2)	94.2870(10)	97.3100(10)
γ (°)	90	90	90	90	90
Volume ($Å^3$)	3215.9(3)	3094.20(17)	1569.54(12)	2941.70(14)	3033.1(2)
Z	4	4	4	2	4
Density (g/cm^3)	1.668	1.686	1.356	1.730	1.735
μ (mm^{-1})	8.116	3.834	0.341	4.032	3.914
F(000)	1616	1584	680	1528	1584
θ range (°)	2.70 to 68.74	2.126 to 28.294	2.61 to 28.29		1.87 to 26.36
Index ranges	$-22 \le h \le 22$<br>$-9 \le k \le 9$<br>$-27 \le l \le 27$	$-9 \le h \le 10$<br>$-22 \le k \le 22$<br>$-29 \le l \le 30$	$-18 \le h \le 18$<br>$-13 \le k \le 11$<br>$-16 \le l \le 17$	$-15 \le h \le 15$<br>$-18 \le k \le 18$<br>$-19 \le l \le 19$	$-15 \le h \le 15$<br>$-18 \le k \le 18$<br>$-19 \le l \le 20$
Reflns collected	33065	54423	23870	38248	44662
Independent reflns	5936 [R_{int} = 0.1169]	7680 [R_{int} = 0.0428]	3885 [R_{int} = 0.0757]	6012 [R_{int} = 0.00463]	6191 [R_{int} = 0.1030]
Data / restraints /parameters	5936 / 0 / 407	7680 / 3 / 474	3885 / 0 / 193	6012 / 11 / 410	6191 / 0 / 418
GOF on F^2	1.040	1.100	1.068	1.047	0.998
R_1 (I>2σ(I))	0.0610	0.0211	0.0416	0.0254	0.0341
wR_2 (all data)	0.1773	0.0409	0.1013	0.0644	0.0747

Supplementary Table 6: Crystal data for **77**, **79**, and **83-85**

	**77**	**79**	**83**	**84**	**85**

CCDC number	2432194	2432195	2432196	2342197	2342198
Formula	$C_{27}H_{39}BN_9O_5PW$	$C_{13}H_{16}N_2O_3$	$C_{27}H_{35}BN_7O_5PSW$	$C_{29}H_{38}BN_{10}O_2PW$	$C_{15}H_{16}N_2O$
FW (g/mol)	795.28	248.28	795.31	784.32	240.307
Temp (K)	100(2)	100(2)	100(2)	100(2)	100(2)
λ (Å)	0.71073	1.54178	0.71073	0.71073	0.71073
Size (mm)	0.032 × 0.171 × 0.198	0.04 × 0.06 × 0.084	0.035 × 0.05 × 0.107	0.039 × 0.073 × 0.103	0.17 × 0.244 × 0.354
Crystal habit	colourless plate	colourless block	colourless plate	colourless plate	colourless plate
Crystal system	monoclinic	orthorhombic	monoclinic	orthorhombic	monoclinic
Space group	$P\ 2_1/c$	$P\ na2_1$	$P\ 2_1/c$	P bca	$P\ 2_1/c$
a (Å)	12.7135(4)	17.2449(6)	11.8010(7)	11.9107(6)	7.9614(4)
b(Å)	14.9815(4)	8.5757(3)	17.6901(10)	20.7835(10)	17.8055(8)
c (Å)	16.0666(4)	7.6352(3)	15.3327(8)	25.0122(15)	8.2254(4)
α (°)	90	90	90	90	90
β (°)	98.1810(10)	90	106.162(2)	90	97.080(2)
γ (°)	90	90	90	90	90
Volume ($Å^3$)	3029.02(15)	1129.15(7)	3074.4(3)	6191.7(6)	1157.11(10)
Z	4	4	4	8	4
Density (g/cm^3)	1.744	1.460	1.718	1.683	1.379
μ (mm^{-1})	3.921	0.863	3.927	3.829	0.088
F(000)	1592	528	1584	3136	512.305
θ range (°)	2.114 to 28.306	5.13 to 68.51	2.134 to 25.706	2.122 to 26.410	2.75 to 29.59
Index ranges	$-16 \le h \le 16$<br>$-18 \le k \le 19$<br>$-21 \le l \le 21$	$-20 \le h \le 20$<br>$-10 \le k \le 10$<br>$-9 \le l \le 9$	$-13 \le h \le 14$<br>$-21 \le k \le 21$<br>$-18 \le l \le 18$	$-14 \le h \le 14$<br>$-24 \le k \le 25$<br>$-31 \le l \le 31$	$-11 \le h \le 11$<br>$-19 \le k \le 24$<br>$-11 \le l \le 11$
Reflns collected	49229	9086	36299	64751	32867
Independent reflns	7530 [R_{int} = 0.0425]	2052 [R_{int} = 0.0691]	5859 [R_{int} = 0.1060]	5691 [R_{int} = 0.1373]	3243 [R_{int} = 0.0396]
Data / restraints /parameters	7530 / 5 / 418	2052 / 1 / 164	5859 / 18 / 438	5691 / 0 / 416	3243 / 0 / 307
GOF on F^2	1.035	1.023	0.999	1.006	1.1342
R_1 (I>2σ(I))	0.0286	0.0354	0.0364	0.0380	0.0337
wR_2 (all data)	0.0746	0.0849	0.0648	0.0858	0.0654

Supplementary References:

1 Simpson, S. R. *et al.* Phenyl Sulfones: A Route to a Diverse Family of Trisubstituted Cyclohexenes from Three Independent Nucleophilic Additions. *J. Am. Chem. Soc.* **144**, 9489-9499 (2022). https://doi.org/10.1021/jacs.2c03529

2 Gaussian 16 Rev. C.01 (Wallingford, CT, 2016).

3 Smith, J. A. *et al.* Experiments and Direct Dynamics Simulations That Probe η2-Arene/Aryl Hydride Equilibria of Tungsten Benzene Complexes. *J. Am. Chem. Soc.* **142**, 16437-16454 (2020). https://doi.org/10.1021/jacs.0c08032

4 APEX4; Apex5; Saint (Bruker AXS, Inc., Madison, WI, USA, 2019).

5 Krause, L., Herbst-Irmer, R., Sheldrick, G. M. & Stalke, D. Comparison of silver and molybdenum microfocus X-ray sources for single-crystal structure determination. *J. Appl. Crystallogr.* **48**, 3-10 (2015). https://doi.org/10.1107/S1600576714022985

6 Sevvana, M., Ruf, M., Usón, I., Sheldrick, G. M. & Herbst-Irmer, R. Non-merohedral twinning: from minerals to proteins. *Acta Crystallogr. Sect. D: Struct. Biol.* **75**, 1040-1050 (2019). https://doi.org/10.1107/S2059798319010179

7 Sheldrick, G. SHELXT - Integrated space-group and crystal-structure determination. *Acta Crystallogr. Sect. A: Found. Adv.* **71**, 3-8 (2015). https://doi.org/10.1107/S2053273314026370

8 Dolomanov, O. V., Bourhis, L. J., Gildea, R. J., Howard, J. A. K. & Puschmann, H. OLEX2: a complete structure solution, refinement and analysis program. *J. Appl. Crystallogr.* **42**, 339-341 (2009). https://doi.org/10.1107/S0021889808042726

9 Kleemiss, F. *et al.* Accurate crystal structures and chemical properties from NoSpherA2. *Chem. Sci.* **12**, 1675-1692 (2021). https://doi.org/10.1039/d0sc05526c

10 Neese, F., Wennmohs, F., Becker, U. & Riplinger, C. The ORCA quantum chemistry program package. *J. Chem. Phys.* **152**, 224108 (2020). https://doi.org/10.1063/5.0004608

11 Neese, F. Software update: The ORCA program system—Version 5.0. *WIREs Computational Molecular Science* **12** (2022). https://doi.org/10.1002/wcms.1606

12 Sheldrick, G. M. Crystal structure refinement with SHELXL. *Acta Crystallogr. Sect. C; Struct. Chem.* **71**, 3-8 (2015). https://doi.org/10.1107/S2053229614024218

13 Kratzert, D. *FinalCIF*, https://dkratzert.de/finalcif.html.

14 Spek, A. L. PLATON SQUEEZE: a tool for the calculation of the disordered solvent contribution to the calculated structure factors. *Acta Crystallogr. Sect. C: Cryst. Struct. Commun.* **71**, 9-18 (2015). https://doi.org/10.1107/S2053229614024929
